# CK2 alpha prime and alpha-synuclein pathogenic functional interaction mediates synaptic dysregulation in huntington’s disease

**DOI:** 10.1186/s40478-022-01379-8

**Published:** 2022-06-03

**Authors:** Dahyun Yu, Nicole Zarate, Angel White, De’jah Coates, Wei Tsai, Carmen Nanclares, Francesco Cuccu, Johnny S. Yue, Taylor G. Brown, Rachel H. Mansky, Kevin Jiang, Hyuck Kim, Tessa Nichols-Meade, Sarah N. Larson, Katherine Gundry, Ying Zhang, Cristina Tomas-Zapico, Jose J. Lucas, Michael Benneyworth, Gülin Öz, Marija Cvetanovic, Alfonso Araque, Rocio Gomez-Pastor

**Affiliations:** 1 Department of Neuroscience, School of Medicine, University of Minnesota, 321 Church St. SE, Jackson Hall Room 6-145, Minneapolis, MN USA; 2 Center for Magnetic Resonance Research. Department of Radiology, School of Medicine, University of Minnesota, Minneapolis, MN USA; 3 Department of Life and Environment Sciences, University of Cagliari, Cagliari, Italy; 4 Mounds View High School, Arden Hills, MN USA; 5 Minnesota Supercomputing Institute, University of Minnesota, Minneapolis, MN USA; 6 Centro de Biología Molecular ‘Severo Ochoa’ (CBMSO) CSIC/UAM, Madrid, Spain; 7 Networking Research Center On Neurodegenerative Diseases (CIBERNED), Instituto de Salud Carlos III, Madrid, Spain; 8 Present Address: HK, MEPSGEN, Seoul, 05836 South Korea; 9 Present Address: CTZ Department of Functional Biology, Physiology, University of Oviedo, 33006 Asturias, Spain; 10 Present Address: Health Research Institute of the Principality of Asturias (ISPA), 33011 Asturias, Spain

**Keywords:** Huntington’s disease, Polyglutamine, CK2 alpha prime, Alpha-synuclein, Neuroinflammation, Protein aggregation

## Abstract

Huntington’s disease (HD) is a neurodegenerative disorder caused by a CAG trinucleotide repeat expansion in the *HTT* gene for which no therapies are available. *HTT* mutation causes protein misfolding and aggregation, preferentially affecting medium spiny neurons (MSNs) of the basal ganglia. Transcriptional perturbations in synaptic genes and neuroinflammation are key processes that precede MSN dysfunction and motor symptom onset. Understanding the interplay between these processes is crucial to develop effective therapeutic strategies to treat HD. We investigated the role of protein kinase CK2α’, a kinase upregulated in MSNs in HD and previously associated with Parkinson’s disease (PD), in the regulation of neuroinflammation and synaptic function in HD. We used the heterozygous knock-in zQ175 HD mouse model and compared that to zQ175 mice lacking one allele of CK2α’ (zQ175:CK2α’^(±)^). CK2α’ haploinsufficiency in zQ175 mice resulted in decreased levels of pro-inflammatory cytokines, HTT aggregation, astrogliosis and transcriptional alterations of synaptic genes related to glutamatergic signaling. zQ175:CK2α’^(±)^ mice also presented increased frequency of striatal miniature excitatory postsynaptic currents (mEPSCs), an indicator of synaptic activity, and improved motor coordination compared to zQ175 mice. Neuropathological and phenotypic changes mediated by CK2α’ were connected to alpha-synuclein (α-syn) dysregulation and correlated with differences in α-syn serine 129 phosphorylation (pS129-α-syn), a post-translational modification involved in α-synucleinopathy and shown to be regulated by CK2 in PD. pS129-α-syn was increased in the nuclei of MSNs in zQ175 mice and in the striatum of patients with HD, and it decreased in zQ175:CK2α’^(±)^ mice. Collectively, our data established a novel connection between CK2α’, neuroinflammation and synaptic gene dysregulation with synucleinopathy in HD and suggested common molecular mechanisms of neurodegeneration between HD and PD. Our results also support CK2α’ inhibition as a potential therapeutic strategy to modulate neuronal function and neuroprotection in HD.

**Supplementary Information:**

The online version contains supplementary material available at 10.1186/s40478-022-01379-8.

## Introduction

Huntington’s disease (HD) is a neurodegenerative disorder that manifests with progressive motor, cognitive, and psychiatric deficits for which there is no cure. HD is caused by a poly-glutamine (polyQ) expansion in exon 1 of the Huntingtin (*HTT*) gene. This mutation results in progressive misfolding and aggregation of mutant HTT protein (mtHTT) and preferentially affects GABAergic medium spiny neurons (MSNs) in the striatum [1, 25, 30]. Transcriptional perturbations in synaptic genes and neuroinflammation are key processes that precede MSN death and motor symptom onset [19]. However, our understanding of the interplay between these processes, mtHTT aggregation, and their contributions to MSN susceptibility in HD is still incomplete.

Protein kinase CK2 is at the crossroads between neuroinflammation, protein aggregation, and synaptic activity, and has recently emerged as a potential therapeutic target of neurodegeneration [7, 11, 62]. CK2 is a highly conserved serine/threonine kinase composed of two regulatory beta (CK2β) subunits and two catalytic subunits, alpha (CK2α) and alpha prime (CK2α’) [53, 60]. The two catalytic subunits share high structural homology, but they differ in their tissue distribution and their ability to phosphorylate different substrates [6, 13]. Increased CK2 activity was reported in polyQ-HTT expressing cells and in the YAC128 HD mouse model [28]. We previously showed CK2α’, but not CK2α, is induced in cell and mouse models of HD, in human iPSC-MSN like cells derived from patients with HD and in postmortem striatum from patients with HD [37]. CK2α’ genetic knockdown in different HD cell models resulted in decreased HTT aggregation and increased cell viability [37] suggesting a role of CK2α’ in the dysregulation of protein quality control systems and HTT aggregation in HD [37, 38]. However, pharmacological studies conducted in vitro have suggested a protective role of CK2 in HD via HTT phosphorylation [4, 28], imposing the necessity to clarify the specific involvement of CK2α’ in HD pathogenesis and its potential as a therapeutic target.

CK2 is involved in the phosphorylation and aggregation of other pathological proteins like microtubule associated protein tau (MAPT) and α-syn, proteins involved in Alzheimer’s (AD) and Parkinson’s disease (PD) [39, 73]. Phosphorylation of Tau and α-syn contribute to the activation of neuroinflammatory processes, transcriptional dysregulation, and synaptic deficits in AD and PD [46, 55]. Alterations in these proteins have also been associated with HD pathology [29, 54, 70]. In particular, increased levels of α-syn were observed in the plasma of patients with HD [9] and its deletion in R6/1 mice resulted in amelioration of motor deficits [17, 70]. However, the mechanisms by which these proteins are altered in HD and the extent to which they contribute to HD pathophysiology are still unknown.

In this study, we characterized the role of CK2α’ in HD in vivo by using the heterozygous zQ175 HD mouse lacking one allele of CK2α’. CK2α’ haploinsufficiency decreased the levels of pro-inflammatory cytokines and ameliorated the expression of genes related to astrocyte dysfunction. zQ175:CK2α’^(±)^ mice also showed restored expression of genes related to glutamatergic signaling, increased the frequency of mEPSCs, and improved motor behavior compared to zQ175 mice. These neuropathological and phenotypic changes correlated with alterations in α-syn serine 129 phosphorylation (pS129-α-syn) in the striatum, a post-translational modification (PTM) involved in α-synucleinopathy, establishing a novel connection between CK2α’ function and synucleinopathy in HD. Collectively, our data demonstrates that CK2α’ plays a negative role in HD and indicates the therapeutic potential of modulating CK2α’ to achieve enhanced neuronal function and neuroprotection.

## Materials and methods

See Additional File 18 for complete methods.

### Cell lines

Mammalian cell lines used in this study were the mouse-derived striatal cells ST*Hdh*^*Q7*^ and ST*Hdh*^*Q111*^ (Coriell Cell Repositories). Cells were grown at 33ºC in Dulbecco’s modified Eagle’s medium (DMEM, Genesee) supplemented with 10% fetal bovine serum (FBS), 100 U ml^−1^ penicillin/streptomycin and 100 ug ml^−1^ G418 (Gibco), as previously described [37].

### Mouse strains

For this study we used a full-length knock-in mouse model of HD known as zQ175 on the C57BL/6 J background (Stock No. 027410). Tail DNA from a subset of zQ175 mice (n = 6 mice) was used for CAG repeat length analysis at Laragen, Inc. (Culver City, CA) and showed an average CAG repeat of 165.5 ± 7.47 (SD). CK2α′ heterozygous mice (CK2α′^(+/−)^) on the 129/SvEv-C57BL/6 J background (Taconic Biosciences TF3062) were originally obtained from Dr. Seldin (Boston University) [75]. All mice were housed under standard SPF conditions. We also used 5-month WT (mixed background CBA x C57BL/6), R6/1, SNCA^KO^, and R6/1SNCA^KO^ obtained from Dr. Lucas. All animal care and sacrifice procedures were approved by the University of Minnesota Institutional Animal Care and Use Committee (IACUC) in compliance with the National Institutes of Health guidelines for the care and use of laboratory animals under the approved animal protocol 2007-38316A.

### siRNA transfection, RNA preparation and RT-qPCR

For CK2α’ knockdown, ST*Hdh* cells were transfected with FlexiTube siRNA (5 nmol) from Qiagen (Mm_Csnk2a2; SI00961051; SI00961058; SI00961065; SI00961072) using DharmaFECT1 per manufacturer’s guidelines. As a negative control, ON-TARGETplus control Non-targeting pool siRNA (Dharmacon) was used. Cells were collected 24 h after transfection. RNA was extracted from ST*Hdh* cells and mouse striatal tissues by using the RNeasy extraction kit (Qiagen) according to the manufacturer’s instructions. cDNA for all was prepared using the Superscript First Strand Synthesis System (Invitrogen). SYBR green based PCR was performed with SYBR mix (Roche). The qPCR amplification was performed using the LightCycler 480 System (Roche). Each sample was tested in triplicate and normalized to GAPDH levels.

### Immunoblot analysis

Sample preparation and immunoblotting were performed as previously described [37]. Striatum protein extracts from one hemisphere of mice were prepared in cell lysis buffer (25 mM Tris pH 7.4, 150 mM NaCl, 1 mM EDTA, 1% Triton-X100 and 0.1% SDS). Primary antibodies were anti-CK2α’ (Novus NB100-379 and Proteintech 10,606–1-AP), anti-Iba1 (FUJIFILM Wako 019–19,741), α-syn (Biolegend 834,303 clone 4D6), pS129-α-syn (Millipore MABN826, clone 81A and Abcam ab51253, EP1536Y), GAPDH (Santacruz sc-365062). Quantitative analyses were performed using ImageJ software and normalized to GAPDH controls.

### Immunohistochemistry

Sample preparation was performed as previously described [37]. Fluorescent images were acquired on an epi-fluorescent microscope (Echo Revolve) or confocal microscope (Olympus FV1000). Primary antibodies used are as follows: α-syn (Biolegend 834,303), pS129-α-syn (Millipore MABN826 and Cell signaling technology 23076S, D1R1R), CK2α’ (Proteintech 10,606–1-AP), Ctip2 (Abcam ab18465), GS (BD Biosciences 610,517 and Abcam 49,873), HTT (Millipore, clone mEM48 Mab5374, and Abcam ab109115), Iba1 (FUJIFILM Wako 019–19,741), NeuN (Millipore MAB377), IL-6 (Santa Cruz Bio sc-32296). For cell number (Ctip, GS, NeuN, Iba1, DAPI), the Cell counter plugin from ImageJ software was used and normalized to the image area (300μm^2^). EM48^+^ and α-syn puncta were counted using annotations in the Echo Revolve software and using the Puncta Analyzer plugin in ImageJ.

### Nuclear/Cytoplasm fractionation

Frozen striatum samples (~ 20 mg) were fractionated using the Minute™ Cytosolic and Nuclear Extraction Kit for Frozen/Fresh tissues (Invent Biotechnologies INC, Cat NT-032) as per Manufacturer’s instructions.

### Electrophysiological analyses

Acute dorsolateral striatum coronal slices (350 μm thick) were obtained from 12 months old mice using a vibratome, and processed as previously described [12]. Researchers were blind to the mouse genotype. The brain was quickly removed after decapitation and placed in ice-cold artificial cerebrospinal fluid (ACSF) containing (in mM): NaCl 124, KCl 2.69, KH_2_PO_4_ 1.25, MgSO_4_ 2, NaHCO_3_ 26, CaCl_2_ 2, ascorbic acid 0.4, and glucose 10, and continuously bubbled with carbogen (95% O_2_ and 5% CO_2_) (pH 7.4). For excitatory postsynaptic currents (EPSCs) picrotoxin (50 µM) and CGP54626 (1 µM) were added. Whole-cell electrophysiological recordings were obtained using patch electrodes (3–10 MΩ) filled with an internal solution containing (in mM): KMeSO_4_ 135, KCl 10, HEPES-K 10, NaCl 5, ATP-Mg 2.5, GTP-Na 0.3 (pH 7.3). Membrane potentials were held at − 70 mV. For EPSCs, theta capillaries filled with ACSF were used for bipolar stimulation. Input–output curves of EPSCs were made by increasing stimulus intensities from 0 to 100 μA. Paired-pulse facilitation was done by applying paired pulses (2 ms duration) with 25, 50, 75, 100, 200, 300, and 500 ms inter-pulse intervals. The paired-pulse ratio was calculated by dividing the amplitude of the second EPSC by the first (PPR = EPSC-2/EPSC-1). Synaptic fatigue was assessed by applying 30 consecutive stimuli in 15 ms intervals. For miniature EPSCs (mEPSCs) tetrodotoxin (TTX; 1 μM) was added to the solution.

### Behavioral assays

Sample sizes were calculated using GraphPad Prism 9.0 and GPower 3.1 to detect differences between WT versus zQ175 groups with a power of ≥ 0.8. Researchers at the Mouse Behavioral core at University of Minnesota were blinded to the genotypes of the mice during testing. See Additional file 18 for a complete description of all behavioral tests conducted in the study. *Beam Walk*: 19-mm (medium-round) or 10-mm (small-round) diameter and 16-mm (medium-Square) or 10-mm (small-Square) width of 3 feet long wood beams (made in house) were used. Each mouse was placed on the beam at one end and allowed to walk to the goal box. Foot slips were recorded manually when the hind paws slipped off the beam. Testing included 3 training days and 1 test day with 4 consecutive trials each. *Rotarod*: Mice were tested over 3 consecutive days. Each daily session included 3 consecutive accelerating trials of 5 min on the rotarod apparatus (Ugo Basile) with the rotarod speed changing from 5 to 50 RPM over 300 s, with an inter-trial interval of at least 15 min.

### RNA-Seq analyses

Gene expression analysis was carried out using the CHURP pipeline (https://doi.org/10.1145/3332186.3333156) using n = 5 mice/genotype for WT, zQ175, and zQ175:CK2α’^(±)^ and n = 3 mice for CK2α’^(±)^, with a female (F)/male (M) ratio: 4F/1 M WT, 1F/2 M CK2α’^(±)^, 2F/3 M zQ175, 4F/1 M zQ175:CK2α’^(±)^. Differential gene expression was determined with DESeq2 using default setting (PMID: 25,516,281). Genes with a q < 0.1 were considered significant. Outliers’ identification was performed using Cook’s distance (DESeq2). Driver factors of gene expression variance (genotype and/or sex) were evaluated using R package variance Partition. Pathway and clustering analysis were completed with IPA (Ingenuity Systems: RRID: SCR_008653) and gProfiler2 (PMID: 31,066,453). Data visualization was done using various R graphic packages, including ggplo2, ggraph, and DESeq2 visualization functions. The RNA-seq data set generated in this manuscript has been deposited at GEO (accession number GSE160586). Reviewer token “**gpqrigisbxgprqf”.**

### WGCNA analysis

The count-based gene expressions were first transformed using a variance stabilizing method via DESeq2 vst function [50]. The WGCNA R package (v1.69) was used to construct an unsigned gene co-expression network with a soft threshold power [beta] of 6. We used a non-parametric Kruskal–Wallis test (*p* value < 0.05) to identify modules that differed significantly among different genotypes. Data for the Greenyellow module was exported using a Cytoscape format for visualization. Network figures are limited to the top 15% of genes with the strongest network connections. The size of the circles is scaled by the absolute value of the mean log2 fold change between zQ175 and zQ175:CK2α’^(±)^ mice.

### Quantification and statistical analyses

Data are expressed as Mean ± SEM, Mean ± SD, or percentage, analyzed for statistical significance, and displayed by Prism 8 software (GraphPad, San Diego, CA) or Excel software (Microsoft). Datasets involving multiple measurements per animal (i.e. immunofluorescence experiments with multiple images captured per animal) were averaged to generate one mean per animal and analyses were conducted using cluster-based summary statistics. Detailed number of images analyzed per animal can be found in figure legends. Pearson correlation tests were applied to test the normal distribution of experimental data. Normal distributions were compared with Student t-test (two-tailed or one-tailed), Welch’s t-test or ANOVA with appropriate post-hoc tests (Sidak’s, Dunn’s, or Tukey’s) for multiple comparisons. The accepted level of significance was *p* ≤ 0.05. Statistical analyses for electrophysiological experiments were performed with SigmaPlot 13.0 software. No statistical methods were used to predetermine sample sizes, but sample sizes were chosen to be similar to those reported in previous publications (11).

## Results

### Increased CK2α’ levels in the striatum of zQ175 mice parallel progressive HTT aggregation and NeuN depletion

**Fig. 1:**
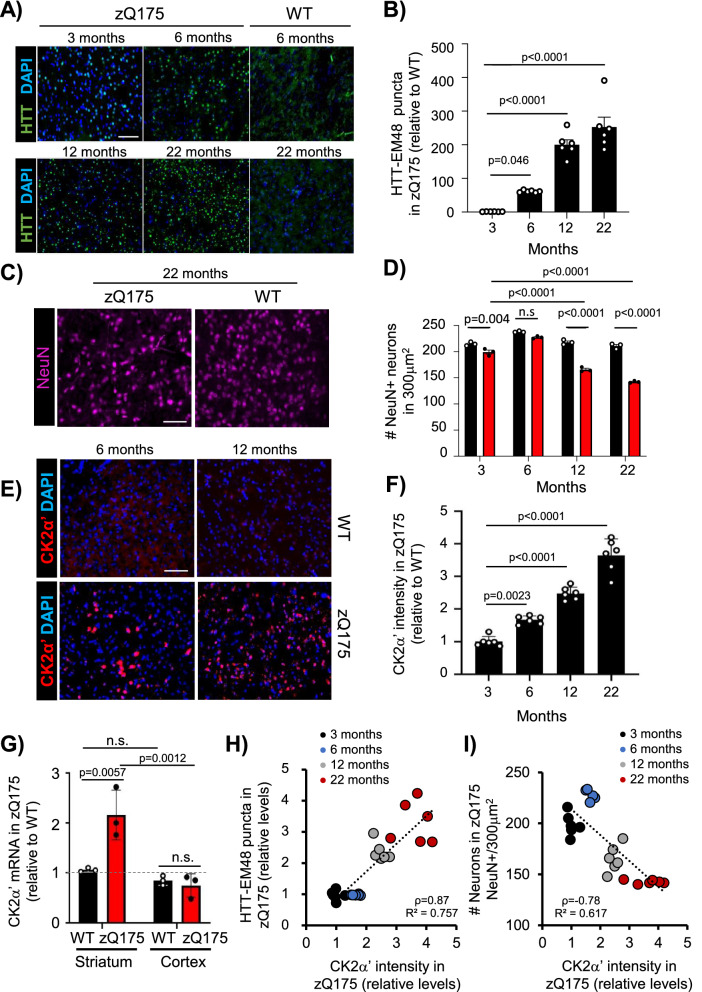
CK2α’ levels progressively increase in striatum of zQ175 and correlate with HTT aggregation and NeuN depletion. **A**–**F,** Immunostaining and quantification of HTT puncta detected with anti-HTT EM48 antibody (*n* = 6 mice/genotype, 6 images averaged/mouse) (**A**, **B**), NeuN^+^ cells (*n* = 3 mice/genotype, 15 images averaged/mouse) (**C, D**) and CK2α’ levels (*n* = 6 mice/genotype, 6 images averaged/mouse) (**E**, **F**) in zQ175 compared with WT mice at 3, 6, 12 and 22 months. **G,** CK2α’ mRNA levels analyzed by RT-qPCR in striatum and cortex of 6-month-old mice. Data was normalized to GAPDH and WT striatum (n = 3 mice/genotype,). **H**, Linear regression analysis between CK2α’ levels and HTT puncta, and ***I***, between CK2α’ levels and number of NeuN^+^ cells in zQ175 mice. The Pearson correlation coefficient (ρ) and *R*^*2*^ are indicated. Data are mean ± SEM with significance determined by one-way ANOVA with Dunnett’s post-hoc test in **B**, mean ± SD with significance determined by one-way ANOVA Dunnett’s post-hoc test in **F** and two-way ANOVA withTukey’s post-hoc test in **D** and **G**. *p*-values < 0.05 are indicated. n.s = not significant. Scale bar, 50 µm

Increased CK2 activity has been associated with detrimental effects in protein homeostasis and neuroinflammation in different neurodegenerative diseases, but its role in HD is still controversial [4, 28, 37]. To determine whether CK2α’ plays a negative role during HD pathogenesis, we first evaluated the relationship between HTT aggregation, neuronal loss, and CK2α’ levels in the striatum over time for the heterozygous zQ175 mouse model at 3 (pre-symptomatic), 6 (early symptomatic), 12 (symptomatic), and 22 months (late-stage disease) of age [40, 56]. We observed an age-dependent increase of HTT aggregates (EM48^+^ puncta) and fewer NeuN^+^ neurons (pan-neuronal marker) in the striatum of zQ175 mice (Fig. 1a–d, Additional file 1a–c). Increased HTT aggregates were also seen over time in the cortex of zQ175 mice, but they were delayed and significantly lower than in the striatum (Additional file 1a, b), as previously described [10]. We confirmed that the depletion of NeuN^+^ cells correlated with decreased Ctip2^+^ neurons (MSN marker) [3] (Additional file 1d, e). However, we did not observe a significant difference in the total number of neurons, measured by cresyl violet (Additional file 1f-h), or in striatum volume (Additional file 1i, j), suggesting that changes in NeuN and Ctip2 reactivity may reflect transcriptional dysregulation and/or neuronal dysfunction rather than neuronal loss.

Due to the differences observed in the timing and level of HTT aggregation between striatum and cortex (Additional file 1a, b), we hypothesized that specific up-regulation of CK2α’ in the striatum contributes to the enhanced accumulation of HTT aggregates in the striatum. The levels of CK2α’ increased over time in zQ175 mice in the striatum but not in the cortex (Fig. 1e–g), coinciding with the timing of HTT aggregation and preceding robust NeuN depletion in the striatum. Regression analysis demonstrated that CK2α’ levels had a significant positive relationship with HTT aggregation (Pearson r(22) = 0.87, *p* value < 0.001) (Fig. 1h) and a significant negative relationship with the number of NeuN^+^ cells (Pearson r(22) =  − 0.78, *p* value < 0.001) (Fig. 1i).

### Depletion of CK2α’ improves neuronal function and motor coordination

**Fig. 2:**
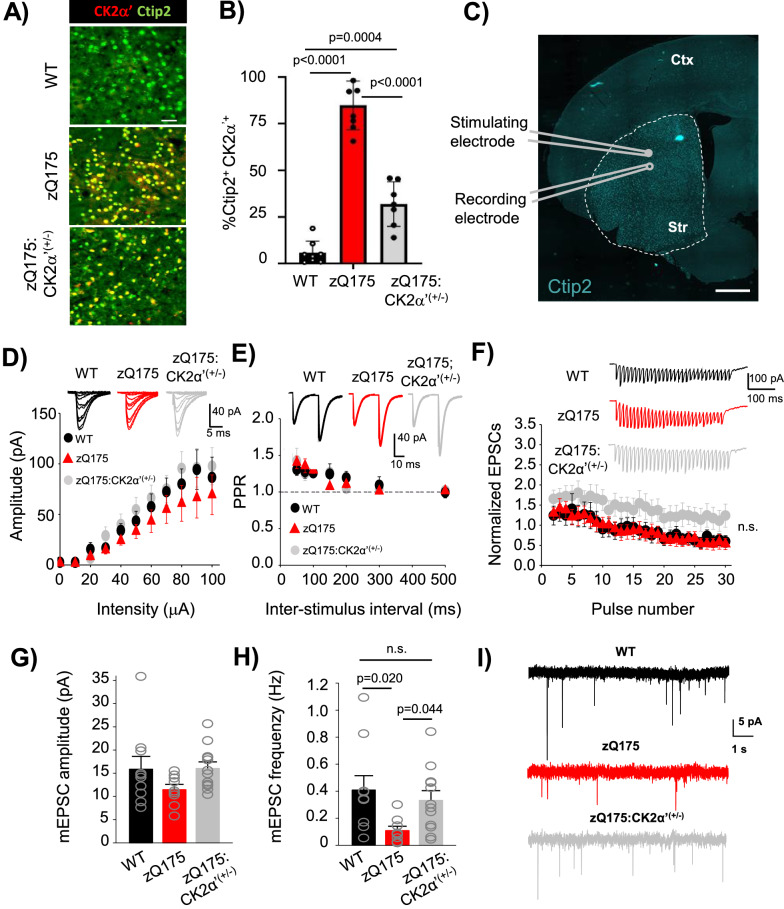
CK2α’ haploinsufficiency increased frequency of striatal AMPA-mediated miniature excitatory postsynaptic currents (mEPSC) in zQ175 mice. a, b, Representative images show the labeling (**A**) and quantification (**B**) of CK2α’ in striatal MSNs immunostained for Ctip2, a specific MSN marker in WT, zQ175 and zQ175:CK2α’ ^(±)^ mice at 12 months of age (*n* = 7–8 mice/genotype, 6 images averaged/mouse). Scale bar, 50 µm. **C**, Image shows whole-cell patch-clamp recording diagram in acute dorsolateral striatum slices, where Ctip2 labeled MSNs from 12-month-old mice. Scale bar 500 µm, Ctr: Cortex; Str: Striatum. **D**, Input–output curve (WT, n = 8 cells from 3 mice; zQ175, n = 9 cells from 4 mice; zQ175:CK2α’^(±)^ n = 13 cells from 4 mice). Representative traces are shown in the top inset. **E**, Short-term potentiation measured via paired-pulse facilitation (WT, n = 8 cells from 3 mice; zQ175, n = 9 cells from 4 mice; zQ175:CK2α’^(±)^ n = 11 cells from 4 mice). Representative traces of two consecutive stimuli delivered at 25 ms time intervals are shown in the top inset. **F**, Short-term depression analyzed through synaptic fatigue (WT, n = 7 cells from 3 mice; zQ175, n = 9 cells from 4 mice; zQ175:CK2α’^(±)^ n = 12 cells from 4 mice). Representative traces are shown in the top inset. Values were analyzed using two-way ANOVA with Tukey’s post-hoc analysis. **G,** **H**, Recordings of mini excitatory postsynaptic currents (mEPSCs). Amplitude (in pA; left panel) (**G**) and frequency (in Hz; right panel) (**h**) were analyzed (WT, n = 10 cells from 3 mice; zQ175, n = 9 cells from 4 mice; zQ175:CK2α’^(±)^ n = 12 cells from 4 mice). **I**, Representative mEPSC traces. Values were analyzed using one-way ANOVA with Dunn´s post-hoc analysis. p values < 0.05 are indicated. Error bars represent mean ± SEM

CK2 has been involved in the regulation of glutamate receptor trafficking via phosphorylation of receptor subunits as well as scaffolding proteins, suggesting a role of CK2 in neuronal signaling [15, 65]. In addition, upregulation of the CK2α’ subunit in HD has been associated with alterations in MSN spine maturation and striatal synapse density in HD mice [37]. Based on this evidence we decided to explore the functional extent of CK2α’ in HD by using a zQ175 mouse model lacking one allele of CK2α’ (zQ175:CK2α’^(±)^) [37] (Fig. 2a, b). We first assessed MSN abundance and striatal synaptic proteins expression (Additional file 2a–c). CK2α’ haploinsufficiency in zQ175 mice did not alter the number of MSNs (Ctip2^+^ cells) or the mRNA levels of the MSN markers (Drd1 and Drd2), but increased the levels of synaptic gene expression like the scaffold protein Dlg4 (PSD-95) and Ppp1r1b (dopamine- and cAMP-regulated neuronal phosphoprotein DARPP-32), a key regulator of the electrophysiological responses in striatal neurons [31, 71] (Additional file 2a–c).

We then assessed the impact of CK2α’ depletion in AMPA-mediated excitatory transmission by conducting whole-cell patch clamp recordings from acute dorsolateral striatum coronal slices at 12 months (Fig. 2c). MSNs from all genotypes showed similar profiles in the analysis of basal synaptic transmission, including input/output curves, paired-pulse facilitation, and synaptic fatigue (Fig. 2d–f). We observed a trend towards increased normalized excitatory postsynaptic currents (EPSCs) in zQ175:CK2α’^(±)^ mice compared to the other two genotypes, but the data did not reach statistical significance (Fig. 2f). Spontaneous neurotransmitter release and synaptic activity via miniature EPSC (mEPSC) recordings showed that mEPSC amplitude, reflecting postsynaptic AMPA receptor function, was comparable among the 3 genotypes (Fig. 2g). However, mEPSC frequency, which reflects the probability of neurotransmitter release from presynaptic vesicles and also correlates with the number of synapses, was reduced in zQ175 mice (Fig. 2h, i), as previously reported [44], and rescued in zQ175:CK2α’^(±)^ mice. These data supported the role of CK2α’ in the dysregulation of striatal synaptic activity in HD mice.

**Fig. 3:**
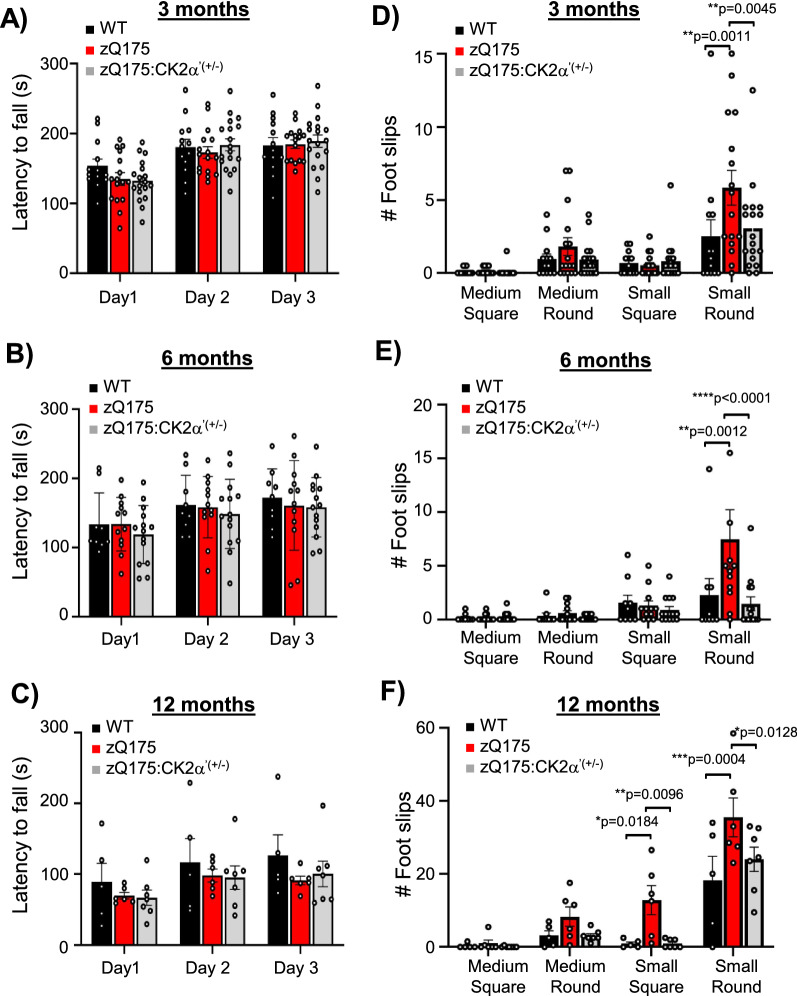
Genetic deletion of CK2α’ improved motor coordination in zQ175. **A**–**C**, Latency to fall off the rod (Rotarod test) for three consecutive days. **D**–**F**, Number of foot slips recorded while walking on four different types of beams with different degrees of difficulty from less to more challenging: medium-square, medium round, small-square and small-round (Beam test). Analyses were performed at 3, 6 and 12 months of age (n = 16–18 mice/genotype in 3 months, n = 12–14 for 6 months and n = 5–6 for 12 months). Error bars denote mean ± SEM, values were analyzed by two-way ANOVA with *Sidak’s* post-hoc test. *p*-values < 0.05 are indicated, n.s = not significant

Glutamatergic synaptic transmission is often related to motor and cognitive function in HD mouse models [67, 71]. We conducted a series of motor tests including accelerating rotarod and beam walk in WT, zQ175, and zQ175:CK2α’^(±)^ mice at 3, 6, and 12 months (Fig. 3). We also conducted cylinder and open field assessments on a different cohort at 12 months comparing zQ175 and zQ175:CK2α’^(±)^ (Additional file 3). We did not observe significant differences between WT and zQ175 or between zQ175 and zQ175:CK2α’^(±)^ at any tested age in the accelerating rotarod test (Fig. 3a–c), open field, or cylinder test (Additional file 3). However, when we evaluated fine motor coordination and whole-body balance in the beam test, we observed a significant increase in foot slips of zQ175 mice compared to WT at 3 months, but only with the most challenging beam (small round), indicating early subtle motor deficits (Fig. 3d). At 12 months, zQ175 mice showed increased foot slips in both the small round and small square beams compared to WT, highlighting a worsening motor deficit (Fig. 3f). zQ175:CK2α’^(±)^ mice showed a significant reduction in foot slips compared to zQ175 mice at all tested ages and no significant differences compared to WT.

zQ175 mice also manifest cognitive deficits within 6–12 months of age, coinciding with disease progression, which is also seen in patients with HD [20, 21, 56, 57]. We performed tests to evaluate associative learning (fear conditioning), spatial learning and memory (Barnes maze, BM), cognitive flexibility (BM reversal), and spatial working memory (Y radial arm maze) by comparing zQ175 and zQ175:CK2α’^(±)^ mice at 12 months of age, but no significant differences were observed between the two groups (Additional file 4). This observation suggests that the positive effects of CK2α’ depletion on motor behavior may not additionally translate to improved cognitive functions.

### CK2α’ depletion rescued transcriptional dysregulation of genes involved in glutamatergic signaling

**Fig. 4:**
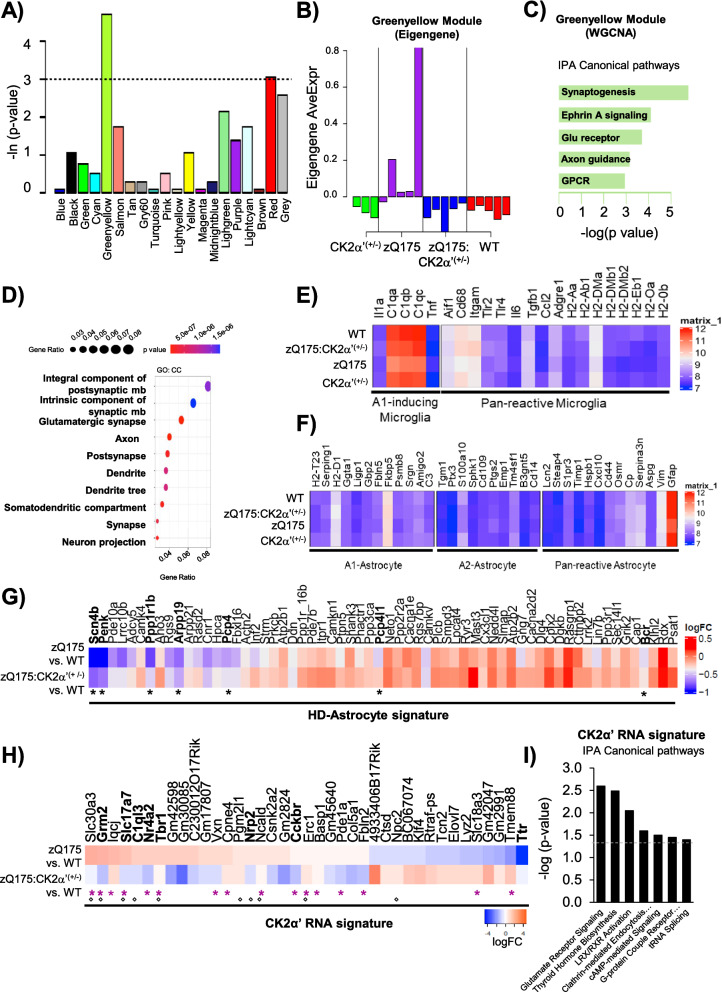
Depletion of CK2α’ restored synaptic gene expression associated with α-syn-dependent regulation in zQ175 mice. **A**, Kruskal–Wallis test of module expressions between zQ175 (HD) mice and zQ175:CK2α’^(±)^ mice. The y-axis is the negative log transformed *p*-values. **B**, Expressions of module “Greenyellow” in each mouse sample. **C**, IPA canonical pathway analysis, **D**, enrichment analysis of GO terms in CC (cellular component). ***E–F***, Gene expression for microglia marker genes; A1-inducing and pan-reactive microglia genes (40) (**E**) and astrocyte markers representative of A1, A2 and pan-reactive astrocytes genes (40) (**F**) in WT, zQ175, CK2α’^(±)^ and zQ175:CK2α’^(±)^ mice. **G**, **H**, Mean log2 fold change between zQ175 and zQ175:CK2α’^(±)^ mice compared to WT for genes representative of the HD-astrocyte molecular signature (41) (**G**), and the CK2α’-mediated RNA signature (**H**). Purple asterisk (*) indicates synaptic function, (◊) indicates genes present in the Greenyellow module. **i**, IPA canonical pathway analysis for the CK2α’-mediated RNA signature. FDR < 0.1 was used for all gene expression analyses

We sought to determine whether depletion of CK2α’ levels had any influence in the overall transcriptional dysregulation characteristic of HD and whether those changes could be associated with the functional improvement observed in zQ175:CK2α’^(±)^ mice. We performed RNA-seq in the striatum of 12–14 month old mice, followed by Weighted Gene Co-Expression Network Analysis (WGCNA) to investigate which molecular pathways are affected by CK2α’ using n = 5 mice/genotype for WT, zQ175, and zQ175:CK2α’^(±)^ and n = 3 mice for CK2α’^(±)^. We found that the mouse transcriptome could be clustered into 20 gene co-expression modules (FDR < 0.1) (Additional file 5, 6). Nine modules showed a significant difference in eigengene expression between zQ175 and WT in a Kruskal–Wallis test (p value < 0.05) (Additional file 7, 8a) and two modules (Greenyellow: 255 genes, and Red: 639 genes) were significantly different between zQ175 and zQ175:CK2α’^(±)^ mice (p value < 0.05) (Fig. 4a, b, Additional file 7). Cook’s distance (DESeq2) analyses revealed that these differences were not due to the presence of outliers in our data set (Additional file 8b). We focused our analyses on the Greenyellow module due to its higher significance. Ingenuity pathway analysis (IPA) indicated that the five most significant pathways in the Greenyellow module were signaling pathways for synaptogenesis (*p*-value 1.68E-06), Ephrin A (*p*-value 7.84E-05), glutamate receptor (p-value 1.98E-04), axonal guidance (*p*-value 7.13E-04), and G-protein coupled receptor (GPCR) (*p*-value 1.14E-03) (Fig. 4c), all of which are pathways previously shown to be dysregulated in HD [49]. IPA in the Red module also revealed synaptic signaling related pathways among their five most significant pathways (Additional file 8c). Additional Gene Ontology (GO) annotation of cellular components of the Greenyellow indicated that genes were enriched in synaptic components (Fig. 4d).

Connectivity analyses (Additional file 8d) revealed that the two most connected genes within the hub were Slit1 (Slit Guidance Ligand 1), associated with “poor” behavior and a worse prognosis in the R6/1 mouse mode [34], and Ncald (Neurocalcin delta), which regulates multiple endocytosis-dependent neuronal functions and is situated on a locus that has been associated with earlier clinical onset of HD [16, 61]. Differential Gene Expression (DGE) between WT and zQ175 mice confirmed a large transcriptional dysregulation (n = 885 genes, FDR < 0.1) (Additional file 8e, f, 9), as previously reported [49] while the DGE between zQ175:CK2α’^(±)^ and WT mice only reported 123 genes (Additional file 8 g, h). R package variance Partition confirmed that these expression changes were driven only by genotype and not by differences in sex distribution among our groups (Additional file 8i).

CK2 has been previously associated with neuroinflammatory processes [62, 66], which was supported by the amelioration in the levels of inflammatory cytokines upon reduction of CK2α’ in both HD cells and mice (Additional file 10a-d). Therefore, we examined our data set for microglial and astrocytic inflammatory RNA signatures [52] but did not observe significant changes in the expression of these gene signatures across genotypes (Fig. 4e, f, Additional file 11, 12). Immunoblotting analyses of the microglial marker Iba1 (Ionized calcium binding adaptor molecule), considered a reactive marker of microgliosis, indicated an increase in total Iba1 protein levels between WT and the HD groups but immunohistological analyses of Iba1 showed no differences in the number or area size of Iba1^+^ cells across all genotypes (Additional file 10e-h), in line with the results obtained by RNA-seq (Fig. 4e). The discrepancy between changes in protein levels of inflammatory cytokines and the absence of an inflammatory transcriptional signature could be related to post-translational events potentially regulated by CK2α’. In addition, no changes in the RNA signature characteristic of reactive neurotoxic A1 astrocytes were seen across genotypes in our data set (Fig. 4f). The absence of robust microglial and astrocytic inflammatory RNA signatures in zQ175 and other HD models has previously been demonstrated [24]. However, we recapitulated some transcriptional changes for the so-called ‘*HD-associated astrocyte molecular signature*’ (Fig. 4g), which represents a group of 62 genes specifically altered in HD astrocytes and associated with astrocyte dysfunction [2, 24]. zQ175 mice showed significant decreased expression of Scn4b, Penk, Ppp1r1b, Arpp19, Pcp4, Pcp4l1 and Bcr, compared to WT mice, which are among the 15 top-most dysregulated genes in the HD-associated astrocyte molecular signature (Fig. 4g). Notably, these changes were ameliorated when comparing zQ175:CK2α’^(±)^ and WT mice. Some of these genes are also expressed in neurons (https://www.proteinatlas.org/). Although we cannot rule out a neuronal contribution to the observed changes in these genes in our dataset, we speculate that changes in Scn4b, Penk, Ppp1r1b, Arpp19, Pcp4, Pcp4l1 and Bcr may be related to a diminished astrocytic pathology upon reduction of CK2α’ levels. This hypothesis is supported by the amelioration of astrogliosis (Additional file 13a, b) and the reduction in the astroglia marker myo-inositol, measured by in vivo proton magnetic resonance spectroscopy (^1^H-MRS) [40, 59, 69], when comparing zQ175:CK2α’^(±)^ and zQ175 mice (Additional file 13c-f).

The DGEs analyzed between zQ175 and zQ175:CK2α’^(±)^ revealed 39 specific and significant genes (FDR < 0.1) (CK2α’ RNA signature) (Fig. 4h, Additional file 14), which included Csnk2a2 (CK2α’ gene) as a positive control. Three genes (Ncald, Nrp2, and Slc30a3) were also among the 15% most highly connected members of the Greenyellow module (Additional file 8i). At least 40% of the DGEs (n = 16) were related to synaptic functions (Additional file 14). IPA on the 39 genes showed that the most significant canonical pathway was for glutamate receptor signaling (p-value 2.59E-03) (Fig. 4i), confirming the contribution of CK2α’ to the dysregulation of genes related to excitatory synaptic transmission in HD. However, the available information for the regulation of the 39 gene set did not provide a direct connection between any of these hits and CK2α’, therefore suggesting additional regulators implicated in the CK2α’-mediated RNA signature.

### α-syn participates in CK2α’-mediated synaptic gene dysregulation

**Fig. 5:**
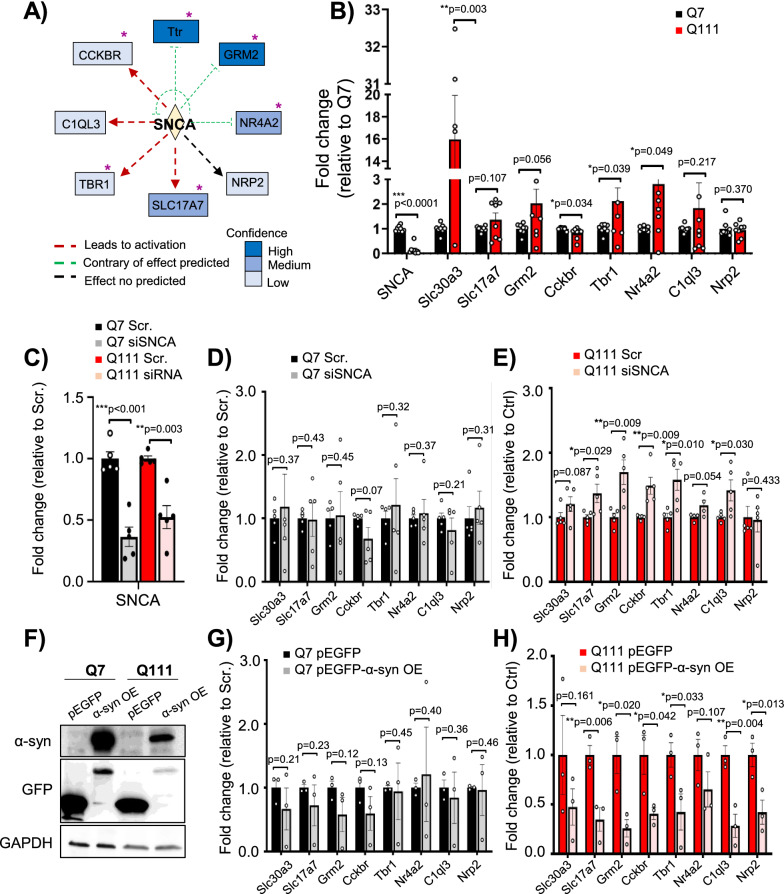
SNCA regulates the expression of genes identified in the CK2α’ mediated RNA signature. **A,** SNCA and DGEs connection by IPA network analysis. Purple asterisks denote synaptic function. **B**, RT-qPCR in Q7 and Q111 cells (*n* = 6–8 experiments). **C**–**E,** siRNA knockdown of SNCA (siSNCA) for 24 h in ST*Hdh Q7 (D)* and Q111 cells (**E**), and RT-qPCR for SNCA (**C**) and SNCA putative gene targets (**D**, **E**). Data were normalized with GAPDH and relativized to non-targeting control siRNA-treated cells (Scr.), (*n* = 5 experiments). Four data points fell in the axis break for Slc30a3. **F**, Immunoblotting for α-syn, GFP and GAPDH in Q7 and Q111 cells after plasmid transfections. Q7 and Q111 cells were transfected with pEGFP (control) or α-syn-GFP overexpression (OE) and harvested 24 h after transfection. **G**, **H**, RT-qPCR for Q7 (**G**) and Q111 cells (**h**) transfected with control or a-syn-GFP. Data were normalized with GAPDH and relativized to non-targeting control pEGFP-treated cells, (*n* = 3 experiments). Data are mean ± SEM with significance determined by Welch’s t-test

When looking at the most significant upstream regulators identified by IPA of both the Greenyellow module and the 39 gene set identified by DGE, we found SNCA (α-syn) (p-value 9.10E-11 and 1.03E-07, respectively). α-syn regulates multiple processes including synaptic vesicle trafficking, neurotransmitter release and transcription [22, 23], and has been previously connected with CK2 [51, 73]. IPA connected α-syn with some of the most differentially dysregulated genes by CK2α’ including Ttr (Transthyretin), Grm2 (Glutamate Metabotropic Receptor 2), Slc17a7 (Solute Carrier Family 17 Member 7; alias VGlut1), C1ql3 (Complement Component 1, Q Subcomponent-Like), Cckbr (cholecystokinin B receptor), Nrp2 (Neuropilin 2), and the transcription factors Tbr1 (T-Box Brain Transcription Factor 1) and Nr4a2 (Nuclear Receptor Subfamily 4 Group A Member 2; alias Nurr1) (Fig. 5a).

To determine the extent to which α-syn participates in the regulation of genes identified in the CK2α’-mediated RNA signature by IPA, we silenced or overexpressed SNCA in the murine striatal cell models Q7 (control) and Q111 (HD) cells. We first validated that Q111 cells presented similar gene expression alterations to those observed in zQ175 mice for the putative SNCA targets when compared to Q7 cells (Fig. 5b). Slc30a3 was included as a non-SNCA target control. Ttr expression was not detected in either Q7 or Q111. RT-qPCR showed a significant increase in Slc30a3, Slc17a7, Grm2, Cckbr, Tbr1 and Nr4a2 in Q111 compared to Q7 as observed in zQ175 mice when compared with WT. Interestingly, SNCA transcripts in Q111 cells were significantly lower compared to Q7 (Fig. 5b). Silencing SNCA in Q111 cells significantly increased the expression of several putative SNCA targets; Slc17a7, Grm2, Cckbr, Tbr1 and C1ql3, but not the non-SNCA targeted control gene Slc30a3. No significant effects on Q7 cells were observed (Fig. 5c–e). On the contrary, α-syn overexpression (OE) in Q111 cells had opposite effects on the same SNCA target genes with no effect on Q7 cells. We also conducted analyses in R6/1 and R6/1:SNCA^KO^ mice compared to WT (Additional file 15a, b) [70]. Although R6/1 mice did not show a similar transcriptional alteration for the SNCA target genes to that observed in zQ175, possibly due to disease severity differences between these two mouse models, we observed significantly decreased SNCA transcripts in R6/1 mice compared to WT, as observed in Q111 compared to Q7 cells (Additional file 15b). We also observed that SNCA^KO^ significantly altered the expression of Grm2 in the R6/1 background but not in the WT background (Additional file 15b). Altogether, the effects mediated by SNCA manipulations suggested that transcriptional alterations of some synaptic genes in HD could be mediated by α-syn dysregulation.

### Striatal synucleinopathy is found in zQ175 mice and is reduced by CK2α’ depletion

**Fig. 6:**
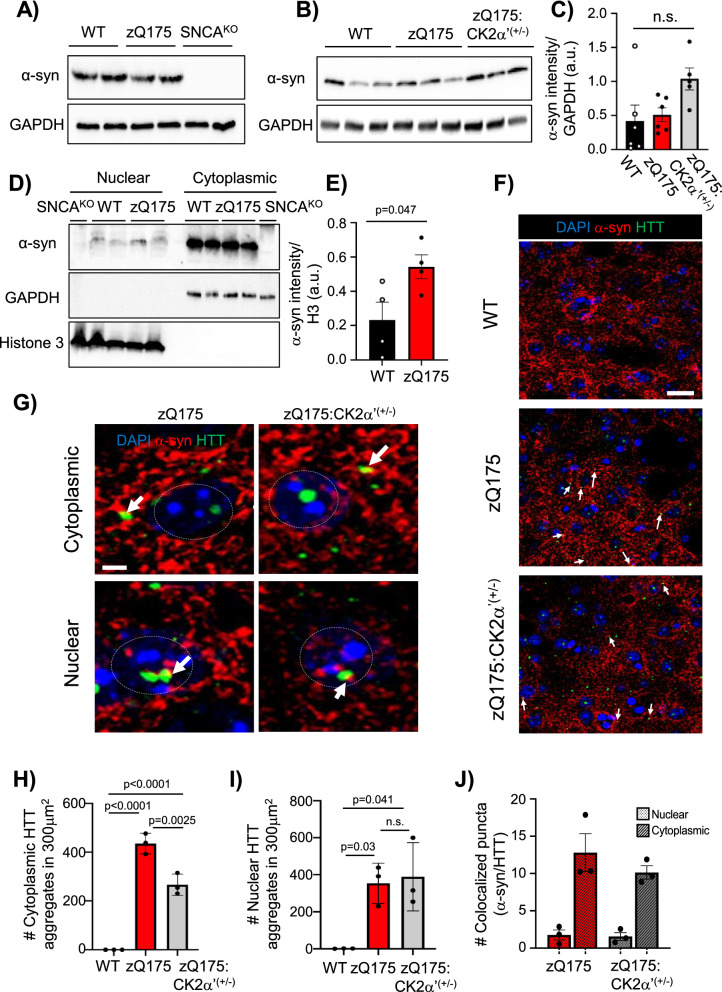
α-syn differentially accumulates in the nucleus of symptomatic zQ175 mice and colocalized with mtHTT. **A,** α-syn (4D6 antibody) IB in the striatum of WT, zQ175 and SNCA^KO^ and **B** in WT, zQ175 and zQ175:CK2α’^(±)^ mice at 12 months old. GAPDH used as loading control. **C,** α-syn protein levels analyzed by Image J from IB analyses (n = 5–6 mice/genotype). **D,** Nuclear/cytoplasmic fractionation of striatum samples from 12-month-old WT, zQ175 and SNCA^KO^ mice. **E**, Quantification of nuclear α-syn from images in D (n = 4 mice/genotype, at least 3 images averaged/mouse). **F**, α-syn and HTT (EM48 antibody) IF images of dorsal striatum sections from 12 month old WT, zQ175 and zQ175:CK2α’^(±)^ (n = 3 mice/genotype). White arrows indicate α-syn/HTT colocalization. Scale bar, 10 μm. **G,** Magnification of images from F. Scale bar, 2 μm. Grey circles represent nuclei. **H** Number of cytoplasmic and ***i*** nuclear EM48^+^ puncta (n = 3 mice/ genotype, 9 images averaged/mouse). **J**, Number of colocalized α-syn and EM48^+^ puncta calculated using Image J Puncta analysis plugin (n = 3 mice/genotype, 6–9 images averaged/mouse). Error bars denote mean ± SEM, values were analyzed by Student’s t-test

We next explored whether CK2α’ was involved in the regulation of α-syn in HD. We observed the total amount of α-syn was similar between WT and zQ175 (Fig. 6a–c) mice. zQ175:CK2α’^(±)^ mice showed a trend towards increased α-syn, but did not reach statistical significance (Fig. 6b, c). Nuclear and cytoplasmic fractionation confirmed the presence of α-syn in nuclear fractions from striatum samples [22, 63], and showed a modest but significant increase in nuclear α-syn in zQ175 mice (Fig. 6d, e). IF analyses for α-syn and HTT (EM48) also confirmed the colocalization between these two proteins (Fig. 6f, g), as previously shown in patients with HD and other HD mouse models [14, 41, 70]. This is also consistent with the annotation of α-syn as a component of the human HTT protein interactome ((http://www.interactome-atlas.org). To determine if there was a difference in the number and distribution of co-localized α-syn/HTT, we first analyzed the number of EM48^+^ puncta in both the nucleus and cytoplasm between zQ175 and zQ175:CK2α’^(±)^ mice. Cytoplasmic HTT aggregates were reduced in zQ175:CK2α’^(±)^ compared to zQ175 mice, consistent with previous studies [37], although no significant differences were observed in the number of nuclear HTT aggregates (Fig. 6h, i). Despite the decrease in cytoplasmic HTT aggregates in zQ175:CK2α’^(±)^ mice, no significant differences were observed in the number of nuclear and/or cytoplasmic α-syn/HTT colocalized puncta between zQ175 and zQ175:CK2α’^(±)^ mice (Fig. 6j).

**Fig. 7:**
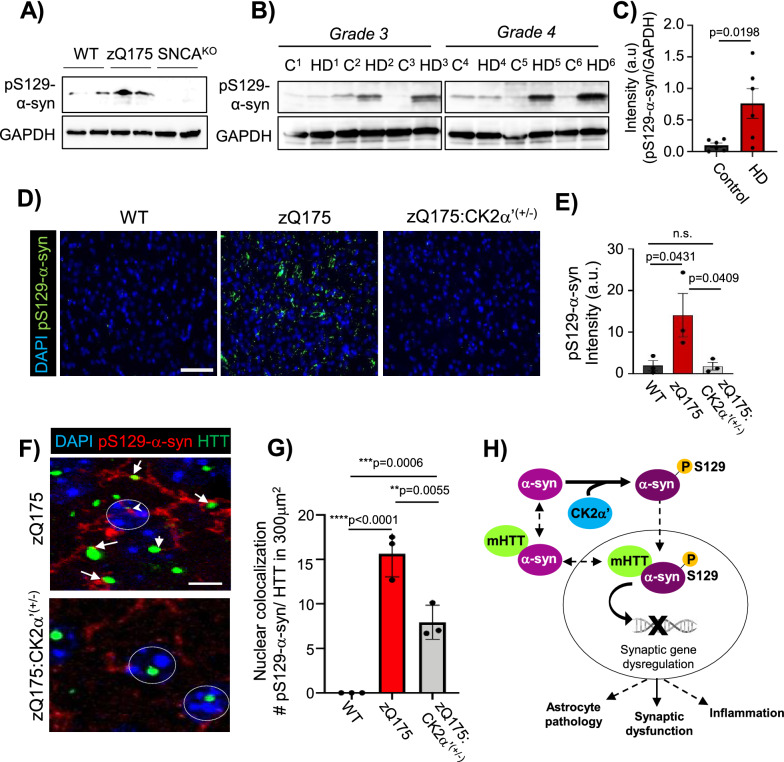
CK2α’ regulates phosphorylation of S129-α-syn and nuclear accumulation in symptomatic zQ175 mice. **A**, pS129-α-syn (EP1536Y antibody) IB in the striatum of 12-month-old WT, zQ175 and SNCA^KO^ (n = 4 mice/genotype). **B**, pS129-α-syn (81A antibody) IB in the striatum of patients with HD (Vonsattel grade 3 and 4, Harvard Brain Tissue Resource Center) compared to age and sex matched controls. GAPDH is used as loading control. ***C***, pS129-α-syn protein levels (combined grades 3 and 4) analyzed by Image J from images in (**B**). **D**, pS129-α-syn IF (81A antibody) in the dorsal striatum of 12-month-old WT, zQ175 and zQ175:CK2α’^(±)^ (n = 3 mice/genotype), Scale bar, 20 μm. **E**, pS129-α-syn fluorescence signal was calculated using Image J from images in D (n = 3 mice/genotype, at least 3 images averaged/mouse). **F**, Magnification of images in D, pS129-α-syn and EM48 colocalization in zQ175 and zQ175:CK2α’^(±)^. **G**, Quantification of pS129-α-syn and EM48 colocalized puncta using Image J puncta plug in (n = 3 mice/group, at least 3 images averaged/mouse). All data are mean ± SEM. Statistical analyses were conducted by one-way ANOVA. **H**, Working model for the role of CK2α’ in the regulation of pS129-α-syn and HD-like phenotype

We then evaluated whether pS129-α-syn, a marker of synucleinopathy [33, 58], was altered in HD and whether CK2α’ could influence its levels. We observed that the levels of pS129-α-syn increased in the striatum of zQ175 mice at 12 months compared to WT (tested with 3 different pS129-α-syn antibodies: 81A, EP1536Y and D1R1R), and in the striatum of patients with HD (Fig. 7a–e, Additional file 16a), indicating signs of synucleinopathy. The levels of pS129-α-syn were significantly reduced in zQ175:CK2α’^(±)^ mice compared to zQ175, while no significant differences were observed when compared with WT mice (Fig. 7d, e, Additional file 16a–c). pS129-α-syn was detected in both the cytoplasm and the nucleus of zQ175 striatal cells, while no nuclear presence was detected in zQ175:CK2α’^(±)^ mice (Fig. 7f, g). In addition, we observed that pS129-α-syn colocalized with both cytoplasmic and nuclear HTT puncta in zQ175 mice, while only cytoplasmic colocalization was observed in zQ175:CK2α’^(±)^ mice (Fig. 7f, g, Additional file 17).

## Discussion

Increased protein kinase CK2 activity has been associated with detrimental effects in protein homeostasis and neuroinflammation in different neurodegenerative diseases, including AD and PD [11]. However, the role of CK2 in HD remained unclear [4, 28, 37]. Here we have demonstrated the adverse effects of CK2α’ catalytic subunit on several HD-related phenotypes including transcriptional dysregulation, pro-inflammatory cytokine levels and astrocyte pathology, HTT aggregation, AMPA-mediated synaptic transmission, and motor coordination in the zQ175 HD mouse model and consolidated the detrimental contribution of CK2α’ to HD pathogenesis. We found CK2α’ contribution to HD was mediated, at least in part, by the ability of CK2α’ to influence α-syn phosphorylation, striatal synucleinopathy and synaptic gene dysregulation (Fig. 7h).

CK2 has been widely associated with the activation of neuroinflammatory processes [26, 36, 66]. Studies in AD demonstrated that pharmacological inhibition of CK2 reduced pro-inflammatory cytokine secretion by human AD astrocytes and increased cell viability [62]. One of the proposed mechanisms is the participation of CK2 in the phosphorylation of components of the IKK (IκB kinase)/NFκB pathway that results in production of proinflammatory cytokines [26]. Although the role of CK2 in inflammation is mostly attributed to the CK2α subunit [66], we showed that CK2α’ haploinsufficiency reduced the levels of several proinflammatory cytokines and diminished astrocyte pathology, assessed by decreased levels of myo-inositol and density of GS + astrocytes in the striatum of zQ175 mice.

Astrocyte pathology and pro-inflammatory cytokines in HD contribute to reduced striatal glutamatergic transmission, spine density, and MSN excitability, ultimately affecting motor behavior [43, 48, 74]. This is supported by recent studies in zQ175 mice expressing a truncated EAAT2-S506X (astrocytic glutamate transporter GLT1 lacking the C-terminal domain), which decreased abnormal protein–protein interactions between astrocytic proteins and GLT-1 and ameliorated deficits in astrocyte glutamate uptake and motor symptoms [42]. Moreover, conditional deletion of mtHTT in astrocytes of BACHD mice also improved astrocyte function, rescued the expression of synaptic proteins like PSD-95 and improved striatal synaptic activity, motor, and psychiatric-like phenotypes [74]. We showed that CK2α’ depletion increased the expression of synaptic genes (PSD-95 and Darpp-32), improved the frequency of mEPSCs (a parameter of AMPA-mediated synaptic transmission) and ameliorated motor deficits. These effects mediated by reduction of CK2α’ could be influenced by improved astrocyte pathophysiology and decreased pro-inflammatory cytokines.

Despite the beneficial effects on neuroinflammation and motor behavior observed by reducing the levels of CK2α’ in zQ175, our transcriptomic analyses did not reveal a neuroinflammatory transcriptional response in either zQ175 or zQ175:CK2α’^(±)^ mice. Similar results were previously reported in zQ175 and other HD mouse models [24]. Instead, we observed transcriptional changes associated with the ‘HD astrocyte molecular signature’, which encompasses a subset of genes identified in astrocyte-specific purified RNA from various HD mouse models representative of astrocyte dysfunction [24]. Our own bulk RNA-seq data, while not cell-specific, showed downregulation of several genes associated with the HD astrocyte molecular signature [24] (Scn4b, Penk, Ppp1r1b and Arpp19) that were rescued in zQ175:CK2α’^(±)^ mice. These results suggest CK2α’ may influence astrocyte pathology in HD. How exactly neuronal CK2α’ influences astrocyte pathophysiology in HD and whether decreased astrocytic pathology contributes to improved neuropathological markers and motor symptoms in zQ175:CK2α’^(±)^ mice is yet to be determined.

In contrast to CK2α, which is an essential protein with hundreds of targets, CK2α’ has very few identified substrates [6, 32]. Studies using CK2 inhibitors proposed that CK2 participates in HTT phosphorylation [4, 8, 28], a PTM that modulates HTT aggregation and toxicity. Therefore, it is possible that CK2α’-mediated changes in HTT aggregation in zQ175 mice could be related to HTT phosphorylation. However, genetic evidence for the direct role of either CK2α or CK2α’ in HTT phosphorylation are lacking and no CK2 consensus sequences (SxxE/D) have been found in HTT [6, 32]. A recently described direct target of CK2α’ is the stress protective transcription factor HSF1, which regulates protein homeostasis [38]. CK2α’ phosphorylates HSF1 in HD signaling the protein for proteasomal degradation, which ultimately alters chaperone expression [37]. Our RNA-seq analysis showed increased expression of chaperones like Hsp70 and Hsp25 in zQ175:CK2α’^(±)^ mice, consistent with previous findings [37]. Therefore, changes in the expression of these chaperones could explain the decrease in HTT aggregation observed in zQ175:CK2α’^(±)^ mice.

Interestingly, WGCNA and DGE did not reveal global changes in transcriptional pathways associated with protein quality control networks in zQ175:CK2α’^(±)^ mice, but instead showed a unique CK2α’-mediated RNA signature related to synaptogenesis and glutamate receptor signaling. These data fit well with the improved frequency of striatal mEPSCs observed by reducing CK2α’ levels and supports previous findings showing increased MSN maturation and striatal excitatory synapse density in zQ175:CK2α’^(±)^ mice [37, 76]. IPA revealed α-syn as one of the top putative upstream regulators of the CK2α’-mediated transcriptional changes. α-syn is a neuronal protein preferentially located in the cytoplasm and was not considered a transcription factor [5]. Recent studies showed α-syn is also present in the nucleus and support a role of α-syn in modulating transcription by either regulating the expression of transcription factors like Nurr1 [22, 23], which is differentially expressed between zQ175 and zQ175:CK2α’^(±)^, or by inducing epigenetic modifications in the DNA [64]. Glutamate receptor signaling genes seem to be selectively altered in mice expressing human α-syn at both the epigenetic and transcriptional level [64]. In aged mice, increased nuclear accumulation of pS129-α-syn in cortical neurons has been correlated with the dysregulation of vesicular glutamate transporter *SLC17A7* [68]. We showed that manipulation of CK2a’ levels in zQ175 mice or α-syn levels in ST*Hdh* Q111 cells significantly altered the expression of several genes involved in glutamatergic signaling including *SLC17A7*. It is unclear how SLC17A7 transcripts can be found in striatal tissues, due to its association with glutamatergic neurons, thought to be absent in the striatum. Recently, it was demonstrated that striatal astrocytes lead to the generation of SLC17A7 expressing glutamatergic neurons in response to injury or aging, that functionally integrate into the adult striatal microcircuitry and contribute to an internal glutamatergic transmission [27]. Overall, our data suggested that CK2α’-mediated expression changes in genes associated with synaptogenesis and glutamatergic signaling alterations in zQ175 mice are α-syn dependent, although additional contributions mediated by astrocyte pathophysiology and/or pro-inflammatory cytokines cannot be ruled out.

α-Syn participates in HD pathogenesis since α-syn KO mice decreased mtHTT aggregation and attenuated body weight loss and motor symptoms in R6/1 mice [18, 70]. However, α-syn’s specific mechanism of action in HD was not established. Similarly, CK2α’ haploinsufficiency attenuated body weight loss [37] and motor symptoms in zQ175 mice. Mice constitutively expressing α-syn in the nucleus exhibited age-dependent motor deficits and decreased protein levels of Darpp32 [35], a protein highly dysregulated in HD and whose expression levels were rescued in zQ175:CK2α’^(±)^ mice. Here we showed that pS129-α-syn levels were increased in the striatum of symptomatic HD mice and patients with HD as well as increased pS129-α-syn localization in the MSN nucleus. pS129-α-syn promotes α-syn nuclear accumulation, aggregation and synucleinopathy and was linked to CK2-dependent phosphorylation in PD, although this site is also the target of other protein kinases [45, 51]. We showed that both nuclear and cytoplasmic pS129-α-syn was decreased when reducing the levels of CK2α’ in zQ175 mice. Considering all the evidence, it is reasonable to hypothesize that CK2α’-mediated increase of pS129-α-syn in the brains of zQ175 mice could contribute to glutamate signaling dysregulation by altering (directly or indirectly) the expression of genes related to those processes and ultimately affecting several HD-like phenotypes evaluated in our study.

HD and PD are both diseases of the basal ganglia, and although they differ in their etiology and specificity of the affected cells, they also share some common motor symptoms including rigidity and involuntary movements (chorea and tremors, respectively), as well as cognitive deficits [47, 72]. Our work here suggests that activation of CK2 and phosphorylation of α-syn could represent a common underlying molecular mechanism of neurodegeneration between these two diseases. Further experiments will be necessary to decipher the mechanism by which CK2α’-mediated α-synucleinopathy contributes to HD and to tease apart the differential contribution of HTT aggregation and α-syn pathology to the symptomatology, onset, and progression of HD.

## Conclusions

Protein kinase CK2α’ is induced in HD. Increased CK2α’ contributes to transcriptional dysregulation of synaptic genes and neuroinflammation in zQ175 HD mice and its depletion improved several HD-like phenotypes in this mouse model. These effects are related to an increased phosphorylation of α-syn (pS129-α-syn) in the striatum of zQ175, a PTM associated with synucleinopathy and PD. We propose that CK2α’ participates (directly or indirectly) in phosphorylating α-syn in HD and such modification could exacerbate some of the phenotypes associated with HD. Our study highlights a potential convergent mechanism of neurodegeneration between HD and PD and suggests targeting CK2α’ as a therapeutic strategy to treat HD and perhaps other neurodegenerative diseases.

## Supplementary Information

**Additional file 1.** Depletion of MSN marker expression does not reflect neuronal loss in zQ175 mice. a, Representative images of EM48 immunostaining in the dorsal striatum and cortex of zQ175 at 3, 6, 12 and 22 months (n=6 mice/genotype, 6-9 images averaged/mouse). b, Quantification of the number of EM48 aggregates in the cortex of zQ175 and its comparison with striatum. c, Representative images of NeuN immunostaining in the dorsal striatum of WT and zQ175 at 3, 6, 12 and 22 months (n=3 mice/genotype). d, e, Representative images of Ctip2 immunostaining in the dorsal striatum of 12 months old WT and zQ175 and quantitative analysis of Ctip2+ cells (n = 3 WT; 3 zQ175, 9 images averaged/mouse). Scale bar, 50 µm. DAPI is used for nuclear staining. f, Cresyl violet staining in the dorsal striatum of 22 months old WT and zQ175 mice. Magnified image represents neurons (white arrow) and glial (black arrow) cells. g, Quantification of neurons from cresyl violet analyses at 12 and h, 22 months old WT and zQ175 mice (3 mice/genotype, 3 images averaged/mouse). i, Dotted line on magnetic resonance images displays the manually traced striatum region of representative mice of each genotype at 22 months of age. j, Striatum volume analyzed in 22-month-old mice from images in G (n=4 mice/genotype, at least 3 images averaged/mouse). Error bars denote mean ± SD. Student’s t-test, p-values <0.05 are indicated. n.s = not significant.

**Additional file 2.** Deficiency in CK2α’ expression does not affect MSN abundance but does influence synaptic genes. a, b, Representative images show the labeling of Ctip2 (a) and quantitative analysis (b) from striatum of zQ175 and zQ175:CK2α’(+/-) mice (n = 3 mice/genotype, at least 3 images averaged/mouse). Scale bar, 50 µm. c, mRNA levels assessed by RT-qPCR of Drd1 and Drd2 (striatal MSN markers), Darpp32 and PSD95 in the striatum of 12 months old mice (n=4 mice/genotype). Error bars denote mean ± SD, values were analyzed by Student’s t-test. p-values <0.05 for differences between groups are indicated in each graph. n.s = not significant.

**Additional file 3.** Genetic deletion of CK2α’ does not ameliorate anxiety-like behaviors or locomotor asymmetry of zQ175 HD at 12 months of age. a-e, Gross motor performance, exploratory behavior, and anxiety-like behavior were assessed during 30 mins in an open field test. a, distance traveled, b, time spent at the center of the field, c, average locomotion velocity and d, time in the outer/inner zone of the field between zQ175 and zQ175:CK2α’ (+/-). e, Representative tracing images show the total distance traveled by the subject. f, Parameters of the spontaneous motor activity (number of rears) evaluated by a cylinder test. Error bars denote mean ± SEM, values were analyzed by two-way ANOVA with Sidak’s post-hoc test. n.s = not significant.

**Additional file 4.** CK2α’ does not alter cognitive behavior in symptomatic HD mice. a, b, Freezing time in the cued (a) and contextual fear conditioning test (b). c-e, Performance on the Barnes maze (BM) task measured by latency to reach the escape hole (c), the mean distance from the target location (d), and total distance until escape in training sessions (e). f-h, Results of BM reversal test are similar to those in BM test, measured by latency to first entry (f), total distance traveled (g) and the mean distance from the target location (h). i, working memory, percent of spontaneous alternation measured by Y maze. Tests were conducted in 12 month old zQ175 and zQ175:CK2α’(+/-).Error bars represent mean ± SEM. Statistical analyses were conducted using ANOVA with Sidak’s post-hoc test (n = 6 mice/genotype). n.s = not significant.

**Additional file 5.** Expressions of all identified co-expression gene modules from WGCNA studies for each mouse sample. A total of 20 different modules were identified when comparing all the genotypes.

**Additional file 6.** WGCNA modules genes assignment.

**Additional file 7.** WGCNA module names and number of genes per module.

**Additional file 8.** RNA-Seq comparison between samples and with Langfelder et al., 2016 [49]. a, Kruskal-Wallis test of module expressions between zQ175 mice and WT mice. b IPA canonical pathway analysis of module genes for module “Red”. c, Cook’s distance (DESeq2), which is a measure of how much a single sample is influencing the fitted coefficients for a gene, for all tested samples (HET = CK2α’(+/-), KI = zQ175, KIHET = zQ175:CK2α’(+/-)). d, Network visualization of top 15% connected genes for module genes of module “Greenyellow”. The size of the circles was scaled by the absolute value of the mean log2-fold change between zQ175 and zQ175:CK2α’(+/-) mice. e, MA-plot of differential gene expression between HD (zQ175) and WT mice. f, Venn Diagram of differentially expressed genes between HD (zQ175) and WT mice in comparison to Langfelder 2016 data. g, MA-plot of differential gene expression between zQ175:CK2α’(+/-) and WT mice. h, Venn Diagram of differentially expressed genes between zQ175, WT, zQ175:CK2α’(+/-) and CK2α’(+/-) mice. i, Driver factors of gene expression variance (genotype and/or gender) for the DGEs identified between zQ175 and zQ175:CK2α’(+/-) were evaluated using R package variance Partition.

**Additional file 9.** Differential Gene Expression analyses across the four different genotypes WT, zQ175, CK2α’(+/-) and zQ175:CK2α’(+/-).

**Additional file 10.** Alteration in CK2α’ expression changes cytokine profiles but not microglia. a, RT-qPCR analysis for CK2α’ and IL-6 in STHdhQ7/Q7 (control) and STHdhQ111/Q111 (HD) cell (n = 5 independent experiments). Data were normalized to GAPDH and control cells. b, siRNA knockdown of CK2α’ for 24 h in STHdhQ111/Q111 cells and RT-qPCR. Data were normalized with GAPDH and relativized to non-targeting control siRNA-treated cells (scramble), (n = 6 independent experiments). c, d, Representative images of mouse cytokine array panels from striatum extracts of WT, zQ175, and zQ175:CK2α’(+/-) at 12-14 months of age (n = 6 mice/genotype). e, f, Iba1 immunoblotting in the striatum of 12 months old WT, zQ175 and zQ175:CK2α’, quantification was measured by image analyses using Image J software, GAPDH is used as a loading marker. g-i, Representative images show the labeling of Iba1 in the dorsal striatum of WT, zQ175 and zQ175:CK2α’(+/-) at 3, 6 and 12 months old (g). Images were analyzed using the Image J software. Scale bar, 100 µm. Iba1+ cell body area in 12 months old mice (n = 3-4 mice/genotype, 18 images averaged/mouse) (h) and percent of Iba1+ cells in 300mm2 (n=3-4 mice/genotype, at least 9 images averaged/mouse) (i). Error bars denote mean ± SEM, values were analyzed by Student's t test in d, and one-way ANOVA and Tukey post-hoc test in a, b, f, h, i. *p represent p-values comparing zQ175 and WT, #p are p-values comparing zQ175 and zQ175:CK2α’(+/-).

**Additional file 11.** Expression analyses for astrocyte markers of A1, A2 and pan-reactive (from Liddelow et al., 2017 (40)) in WT, zQ175, CK2α’(+/-) and zQ175:CK2α’(+/-).

**Additional file 12.** Expression analyses for microglia markers of A1 inducing and reactive microglia (from Liddelow et al., 2017 (40)) in WT, zQ175, CK2α’(+/-) and zQ175:CK2α’(+/-).

**Additional file 13.** Decreased CK2α’ ameliorated astrogliosis in zQ175 mice. a, b, Representative images show the labeling (a) and quantification (b) of Glutamine synthetase (GS, an astrocytic marker) and CK2α’ of striatum sections from 12-month-old mice in WT, zQ175 and zQ175:CK2α’(+/-) (n = 5 mice/genotype, at least 3 images averaged/mouse). DAPI is used for nuclear staining. Scale bars, 50 µm. c, Coronal images of brain scans in 9.4T magnet showing the striatum voxel (green box) for MRS acquisition from each genotype at 22 months. d, Localized proton magnetic resonance spectra [LASER sequence, TE = 15 ms, TR= 5 s, 256 transients, 9.4T] obtained at 22 months of age from the striatum of WT, zQ175, and zQ175:CK2α’(+/-) mice (n = 4 WT; 4 HD; 6 HD: CK2α’ (+/-)). Differences in myo-inositol (Ins) between WT and zQ175 mice and between zQ175 and zQ175:CK2α’(+/-) mice are shown with arrows. e, f, Mean concentrations of myo-inositol (Ins) (e) and other reliably quantified metabolites (f) in the striatum of WT (black), zQ175 (red), and zQ175:CK2α’(+/-) (gray) mice. Asc: Ascorbate/vitamin C; tCr: total creatine + phosphocreatine; GABA: gamma-aminobutyric acid; Glc: glucose; Gln: glutamine; Glu: glutamate; tCho: total phosphocholine + glycerophosphocholine; GSH: glutathione; lns: myo-inositol; Lac: lactate; NAA: N-acetylaspartate; NAAG: N-acetylaspartylglutamate; PE: phosphoethanolamine; Tau: taurine. Error bars denote mean ± SEM, values were analyzed by one-way ANOVA with Tukey’s post-hoc test. p-values < 0.05 are indicated.

**Additional file 14.** Marker genes and their mean log2 fold change between zQ175 and zQ175:CK2α’(+/-) mice compared to WT.

**Additional file 15.** SNCA regulates the expression of synaptic genes identified in IPA analysis in R6/1 mice. a, IB for α-syn (4D6 antibody) and mtHTT (EM48 antibody) in the striatum of 5 months old WT, SNCAKO, R6/1 and R6/1:SNCAKO (n=3 mice/genotype). b, RT-qPCR analyses for SNCA and genes identified in the IPA analyses to be connected to SNCA; (Ttr, Grm2, Slc17a7, Slc30a3, Cckbr, Nrp2, Tbr1 and Nr4a2) (n = 4-5 mice/genotype). Error bars represent mean ± SEM. Statistical analyses were conducted by Student’s t-test. p-values <0.05 are indicated. We also indicated p-values <0.09 for those genes that showed a trend toward decreased expression.

**Additional file 16.** CK2a’ haploinsufficiency decreased the levels of pS129-α-syn. a, Representative pS129-α-syn IF images (D1R1R antibody) in the dorsal striatum of 12 months old WT, zQ175 and zQ175:CK2α’(+/-), Scale bar, 20 μm. b, pS129-α-syn and CK2α’ immunoblotting of striatum samples from 12-month-old zQ175 and zQ175:CK2α’(+/-) mice (n = 3 mice/group). c, Levels of pS129-α-syn and CK2α’ were calculated using Image J from immunoblotting images in a and showed a parallel decrease of pS129-α-syn and CK2α’ levels in zQ175:CK2α’(+/-) compared to zQ175 mice. Error bars represent mean ± SEM. Statistical analyses were conducted by Student’s t-test. p-values <0.05 are indicated (n=3 mice/genotype).

**Additional file 17.** Video showing pS129-α-syn nuclear localization (red) in the striatum of 12-month-old zQ175 mice. DAPI (blue) stains nuclei.

**Additional file 18.** Supplementary Methods.

## Data Availability

RNA-seq data set generated in this manuscript is accessible at GEO (accession number GSE160586). All other data generated or analyzed during this study are included in this published article (and its supplementary information files).

## Abbreviations

α-syn: Alpha synuclein
pS129-α-syn: Phosphoserine 129 alpha synuclein
HD: Huntington’s disease
PD: Parkinson’s’ disease
AD: Alzheimer’s disease
CK2α’: Protein kinase CK2 alpha prime
HTT: Huntingtin
mtHTT: Mutant huntingtin
MSN: Medium spiny neuron
polyQ: Poly-glutamine
MAPT: Microtubule associated protein tau
TNF: Tumor necrosis factor
EPSC: Excitatory postsynaptic currents
IPA: Ingenuity pathway analyses
DGE: Differential gene expression
WGCNA: Weighted gene co-expression network analysis
iPSC: Induced pluripotent stem cells
Scn4b: Sodium channel β4 auxiliary subunit
Penk: Proenkephalin
Ppp1r1b: Protein phosphatase 1 regulatory subunit 1B
Arpp19: CAMP regulated phosphoprotein 19
Slc17a7: Solute carrier family 17 member 7
Grm2: Glutamate metabotropic receptor 2
Cckbr: Cholecystokinin B receptor
Tbr1: T-box brain 1
C1ql3: Complement C1q-Like protein 3
SNCA: Alpha-synuclein
Slc30a3: Solute carrier family 30 member 3
Nrp2: Neuropilin 2
Nr4a2: Nuclear receptor subfamily 4 group A member 2
