# Correction to: CK2 alpha prime and alpha-synuclein pathogenic functional interaction mediates synaptic dysregulation in Huntington’s disease

**DOI:** 10.1186/s40478-022-01397-6

**Published:** 2022-06-22

**Authors:** Dahyun Yu, Nicole Zarate, Angel White, De’jah Coates, Wei Tsai, Carmen Nanclares, Francesco Cuccu, Johnny S. Yue, Taylor G. Brown, Rachel H. Mansky, Kevin Jiang, Hyuck Kim, Tessa Nichols‑Meade, Sarah N. Larson, Katherine Gundry, Ying Zhang, Cristina Tomas‑Zapico, Jose J. Lucas, Michael Benneyworth, Gülin Öz, Marija Cvetanovic, Alfonso Araque, Rocio Gomez‑Pastor

**Affiliations:** 1 Department of Neuroscience, School of Medicine, University of Minnesota, 321 Church St. SE, Jackson Hall Room 6‑145, Minneapolis, MN USA; 2 Center for Magnetic Resonance Research, Department of Radiology, School of Medicine, University of Minnesota, Minneapolis, MN USA; 3 Department of Life and Environment Sciences, University of Cagliari, Cagliari, Italy; 4 Mounds View High School, Arden Hills, MN USA; 5 Minnesota Supercomputing Institute, University of Minnesota, Minneapolis, MN USA; 6 Centro de Biología Molecular ‘Severo Ochoa’ (CBMSO) CSIC/UAM, Madrid, Spain; 7 Networking Research Center on Neurodegenerative Diseases (CIBERNED), Instituto de Salud Carlos III, Madrid, Spain; 8 Present Address: HK, MEPSGEN, Seoul, 05836 South Korea; 9 Present Address: CTZ Department of Functional Biology, Physiology, University of Oviedo, 33006 Asturias, Spain; 10 Present Address: Health Research Institute of the Principality of Asturias (ISPA), 33011 Asturias, Spain

## Correction to: Acta Neuropathologica Communications (2022) 10:83 https://doi.org/10.1186/s40478-022-01379-8

Following publication of the original article [1], an error was identified in the corresponding authorship and equal contributions in the author group due to a typesetting mistake: the third author, Angel White, should have been indicated as equal contributor, instead of as corresponding author.

The author group has been updated above and the original article [1] has been corrected. The publisher apologises to the authors and readers for the inconvenience caused by the error.
