# Supplementary figures and images for: CK2 alpha prime and alpha-synuclein pathogenic functional interaction mediates synaptic dysregulation in huntington’s disease
Source: Acta Neuropathol Commun. 2022 Jun 3;10:83. doi: 10.1186/s40478-022-01379-8 (PMC9164558; doi:10.1186/s40478-022-01379-8)

# Additional file 1

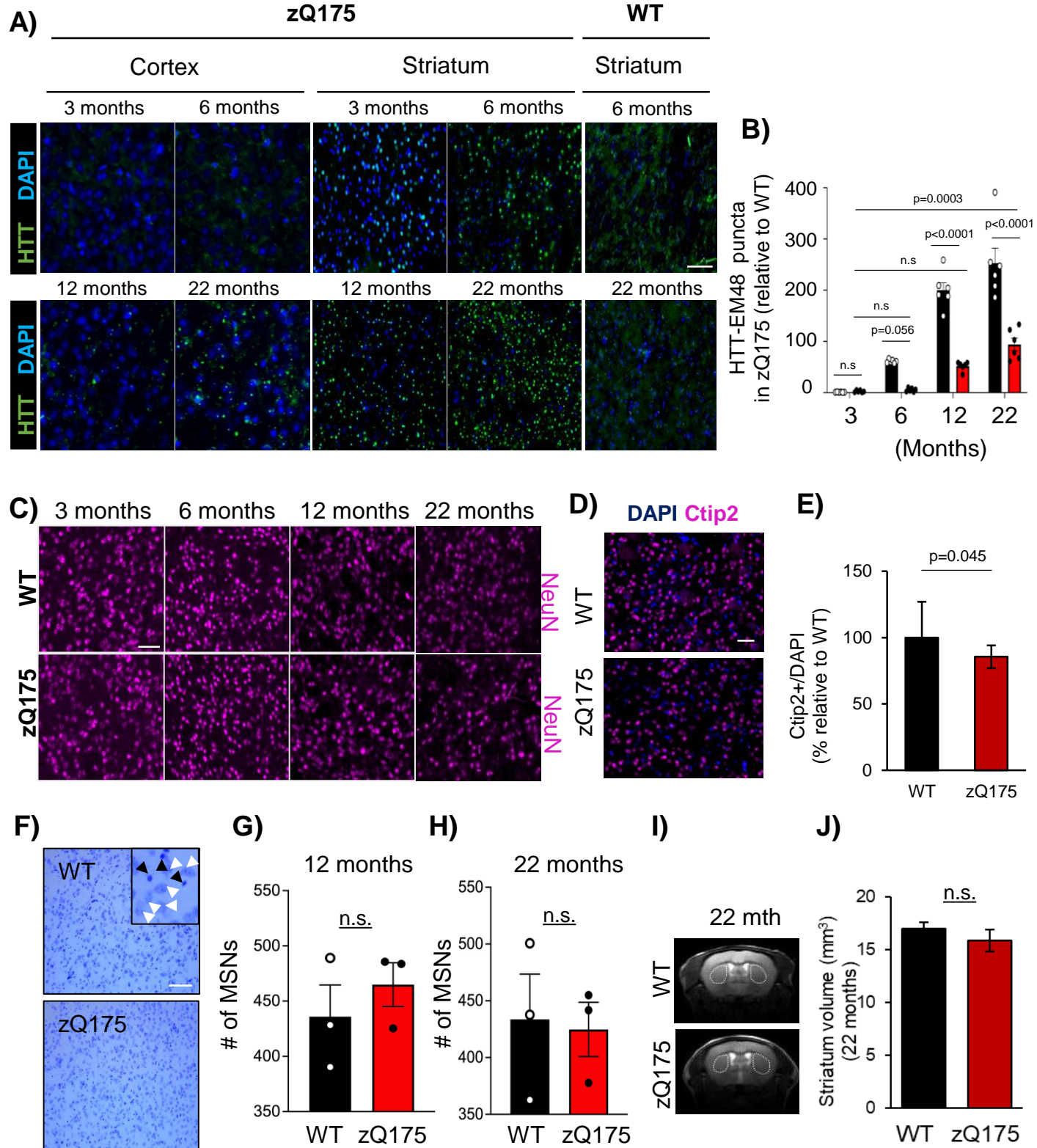

Supplement: Supplementary file 1 — Additional file 1. Depletion of MSN marker expression does not reflect neuronal loss in zQ175 mice. a, Representative images of EM48 immunostaining in the dorsal striatum and cortex of zQ175 at 3, 6, 12 and 22 months (n=6 mice/genotype, 6-9 images averaged/mouse). b, Quantification of the number of EM48 aggregates in the cortex of zQ175 and its comparison with striatum. c, Representative images of NeuN immunostaining in the dorsal striatum of WT and zQ175 at 3, 6, 12 and 22 months (n=3 mice/genotype). d, e, Representative images of Ctip2 immunostaining in the dorsal striatum of 12 months old WT and zQ175 and quantitative analysis of Ctip2+ cells (n = 3 WT; 3 zQ175, 9 images averaged/mouse). Scale bar, 50 µm. DAPI is used for nuclear staining. f, Cresyl violet staining in the dorsal striatum of 22 months old WT and zQ175 mice. Magnified image represents neurons (white arrow) and glial (black arrow) cells. g, Quantification of neurons from cresyl violet analyses at 12 and h, 22 months old WT and zQ175 mice (3 mice/genotype, 3 images averaged/mouse). i, Dotted line on magnetic resonance images displays the manually traced striatum region of representative mice of each genotype at 22 months of age. j, Striatum volume analyzed in 22-month-old mice from images in G (n=4 mice/genotype, at least 3 images averaged/mouse). Error bars denote mean ± SD. Student’s t-test, p-values <0.05 are indicated. n.s = not significant. [file 40478_2022_1379_MOESM1_ESM.pdf]

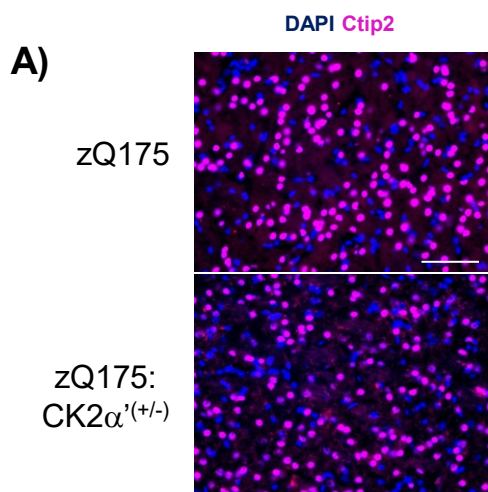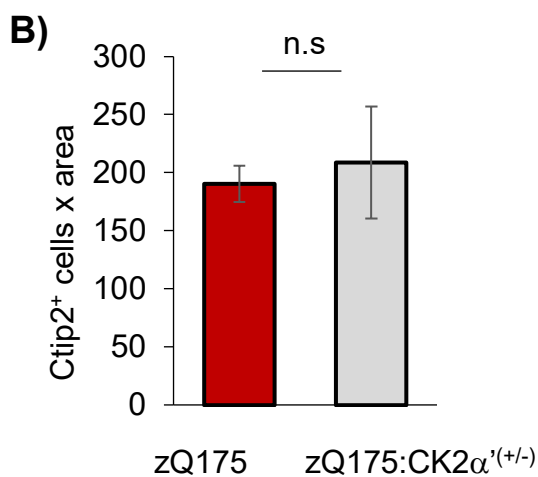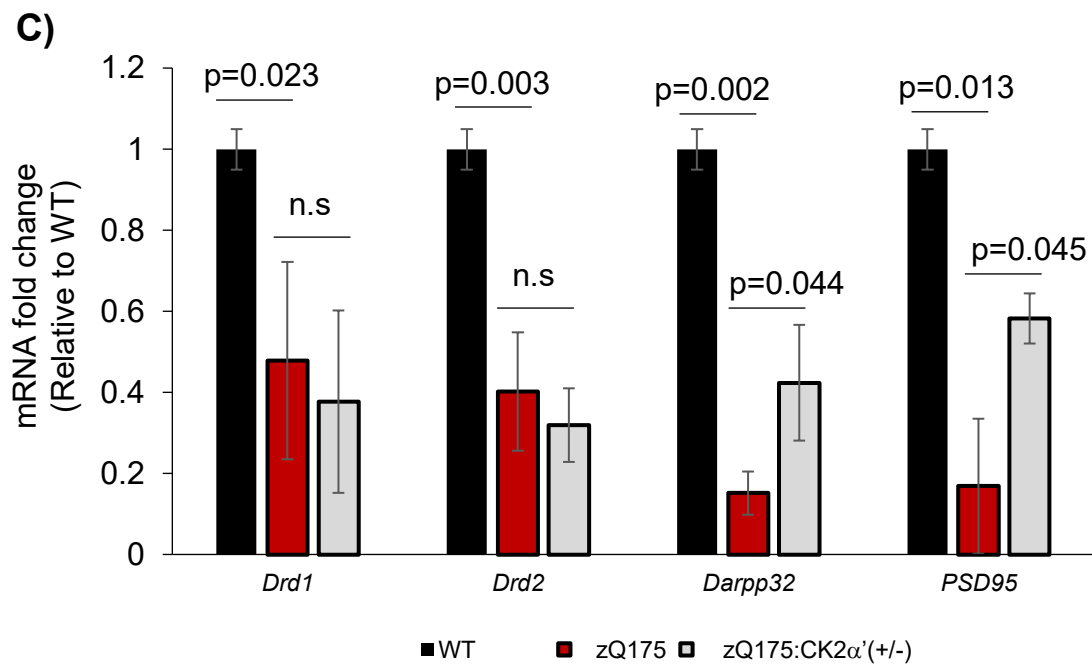

Supplement: Supplementary file 2 — Additional file 2. Deficiency in CK2α’ expression does not affect MSN abundance but does influence synaptic genes. a, b, Representative images show the labeling of Ctip2 (a) and quantitative analysis (b) from striatum of zQ175 and zQ175:CK2α’(+/-) mice (n = 3 mice/genotype, at least 3 images averaged/mouse). Scale bar, 50 µm. c, mRNA levels assessed by RT-qPCR of Drd1 and Drd2 (striatal MSN markers), Darpp32 and PSD95 in the striatum of 12 months old mice (n=4 mice/genotype). Error bars denote mean ± SD, values were analyzed by Student’s t-test. p-values <0.05 for differences between groups are indicated in each graph. n.s = not significant. [file 40478_2022_1379_MOESM2_ESM.pdf]

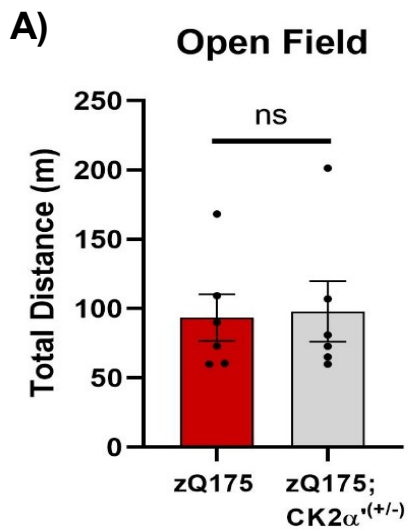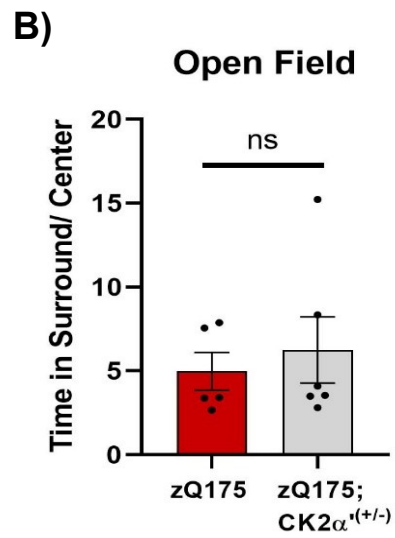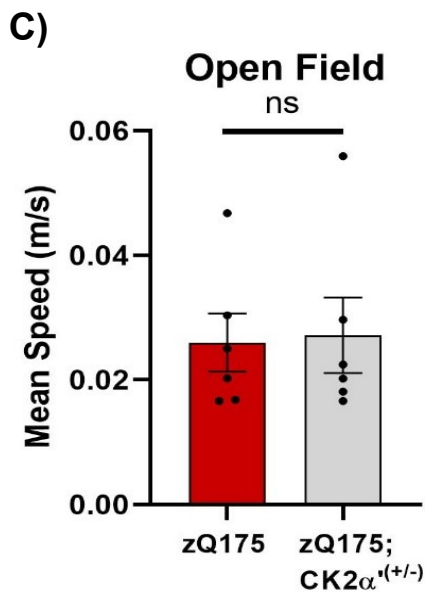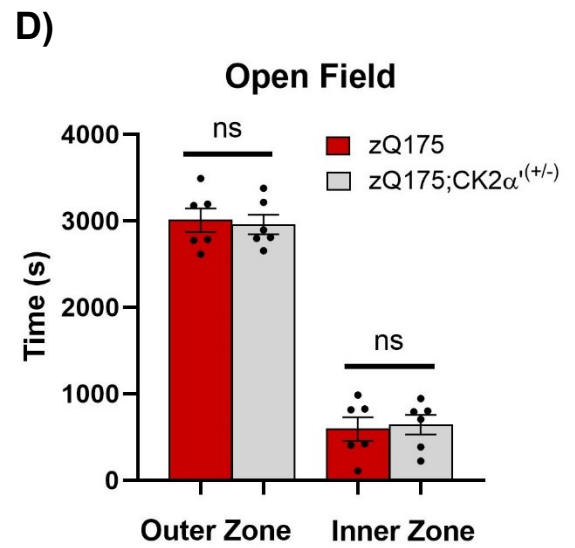

**E)**

Trace Maps

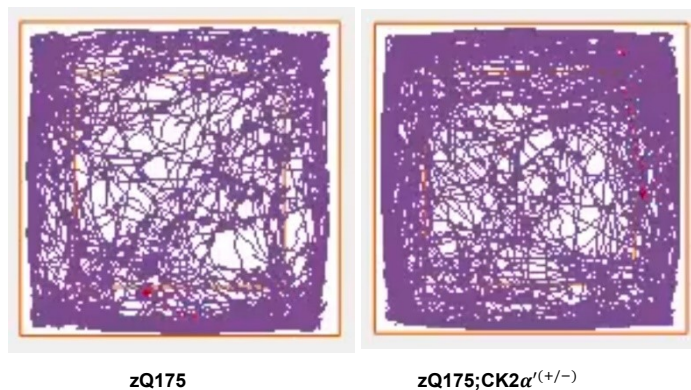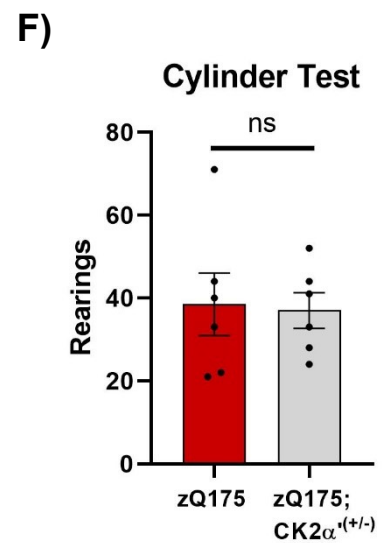

Supplement: Supplementary file 3 — Additional file 3. Genetic deletion of CK2α’ does not ameliorate anxiety-like behaviors or locomotor asymmetry of zQ175 HD at 12 months of age. a-e, Gross motor performance, exploratory behavior, and anxiety-like behavior were assessed during 30 mins in an open field test. a, distance traveled, b, time spent at the center of the field, c, average locomotion velocity and d, time in the outer/inner zone of the field between zQ175 and zQ175:CK2α’ (+/-). e, Representative tracing images show the total distance traveled by the subject. f, Parameters of the spontaneous motor activity (number of rears) evaluated by a cylinder test. Error bars denote mean ± SEM, values were analyzed by two-way ANOVA with Sidak’s post-hoc test. n.s = not significant. [file 40478_2022_1379_MOESM3_ESM.pdf]

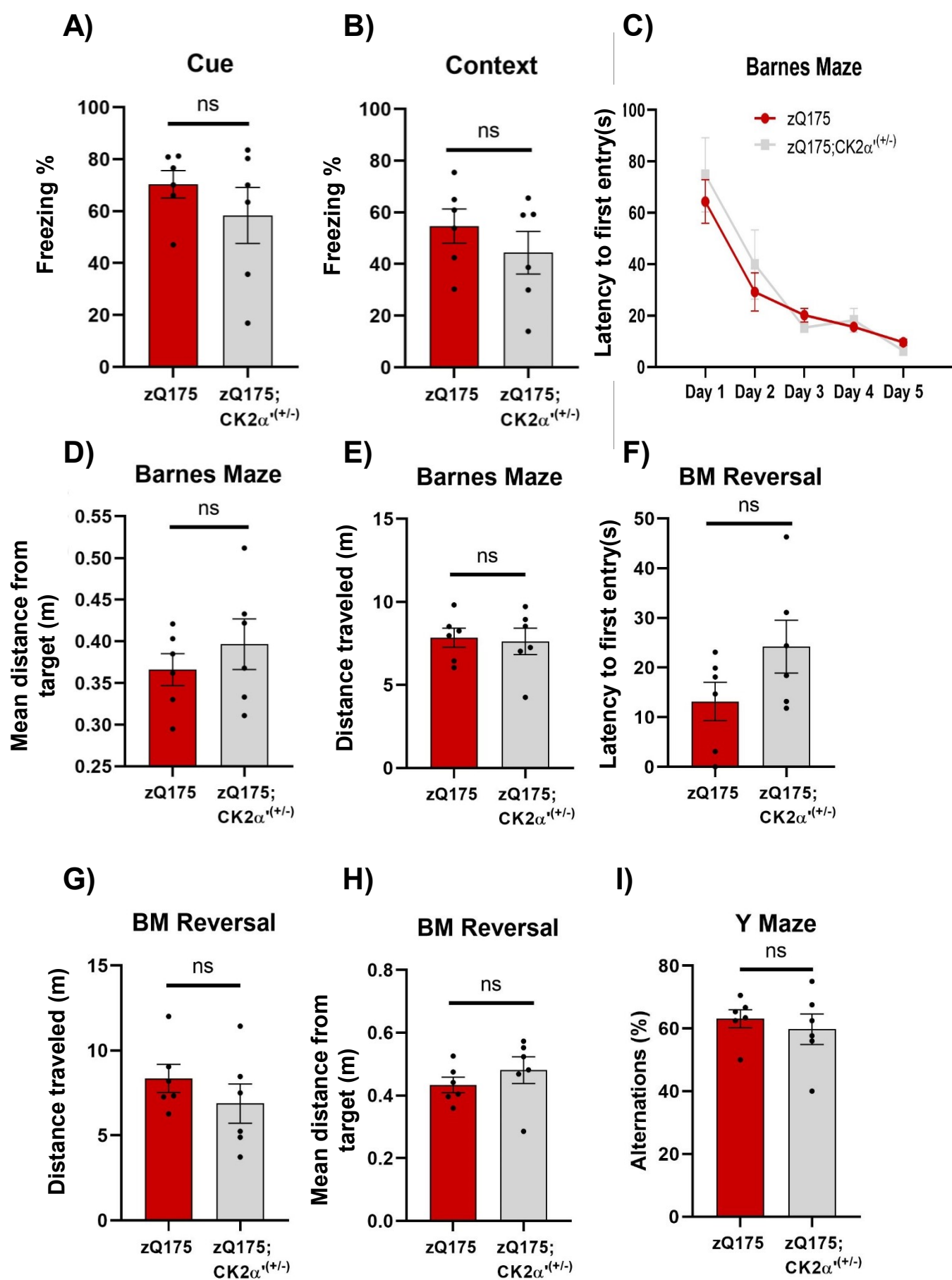

Supplement: Supplementary file 4 — Additional file 4. CK2α’ does not alter cognitive behavior in symptomatic HD mice. a, b, Freezing time in the cued (a) and contextual fear conditioning test (b). c-e, Performance on the Barnes maze (BM) task measured by latency to reach the escape hole (c), the mean distance from the target location (d), and total distance until escape in training sessions (e). f-h, Results of BM reversal test are similar to those in BM test, measured by latency to first entry (f), total distance traveled (g) and the mean distance from the target location (h). i, working memory, percent of spontaneous alternation measured by Y maze. Tests were conducted in 12 month old zQ175 and zQ175:CK2α’(+/-).Error bars represent mean ± SEM. Statistical analyses were conducted using ANOVA with Sidak’s post-hoc test (n = 6 mice/genotype). n.s = not significant. [file 40478_2022_1379_MOESM4_ESM.pdf]

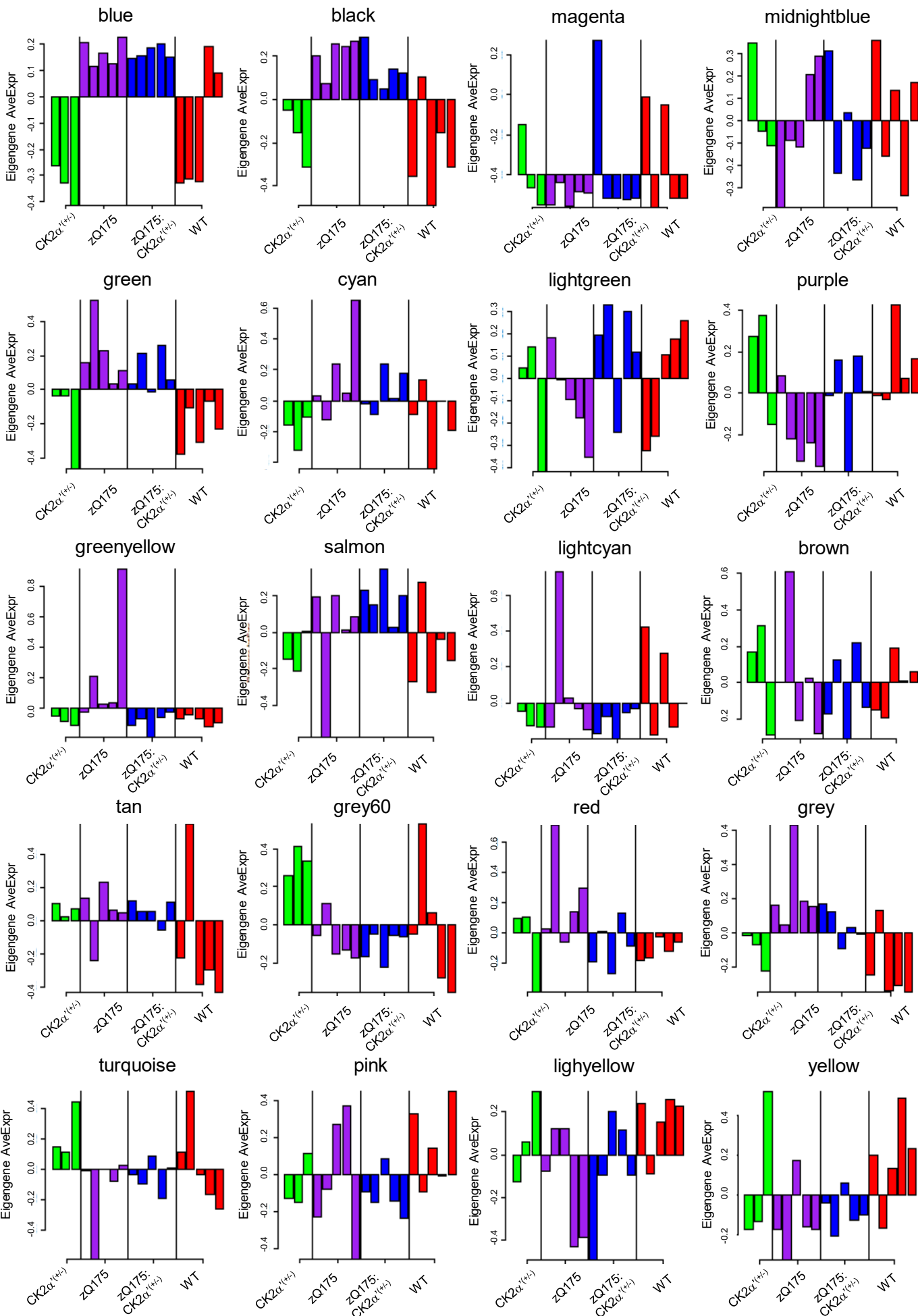

**Additional file 5**

Supplement: Supplementary file 5 — Additional file 5. Expressions of all identified co-expression gene modules from WGCNA studies for each mouse sample. A total of 20 different modules were identified when comparing all the genotypes. [file 40478_2022_1379_MOESM5_ESM.pdf]

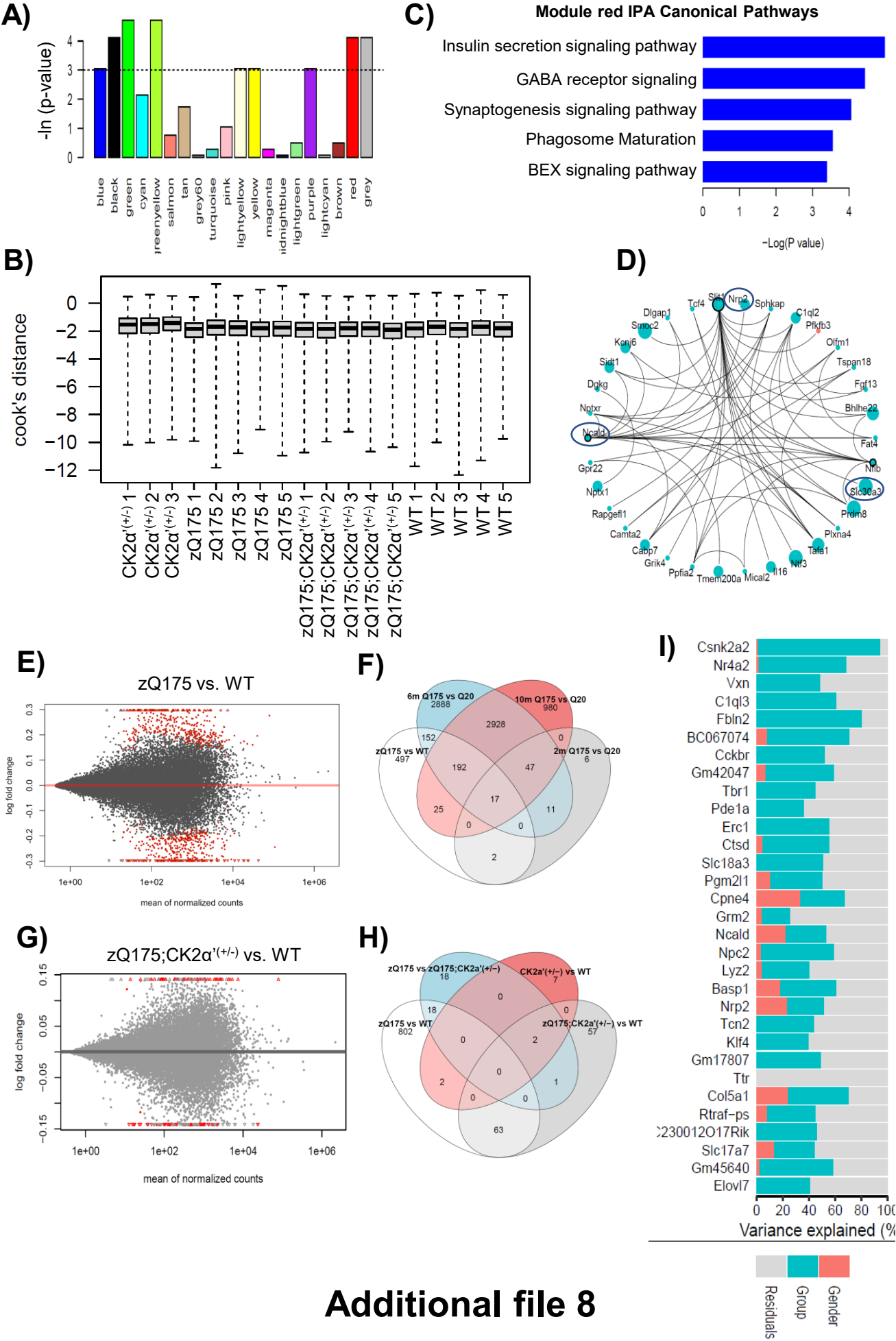

Supplement: Supplementary file 8 — Additional file 8. RNA-Seq comparison between samples and with Langfelder et al., 2016 [49]. a, Kruskal-Wallis test of module expressions between zQ175 mice and WT mice. b IPA canonical pathway analysis of module genes for module “Red”. c, Cook’s distance (DESeq2), which is a measure of how much a single sample is influencing the fitted coefficients for a gene, for all tested samples (HET = CK2α’(+/-), KI = zQ175, KIHET = zQ175:CK2α’(+/-)). d, Network visualization of top 15% connected genes for module genes of module “Greenyellow”. The size of the circles was scaled by the absolute value of the mean log2-fold change between zQ175 and zQ175:CK2α’(+/-) mice. e, MA-plot of differential gene expression between HD (zQ175) and WT mice. f, Venn Diagram of differentially expressed genes between HD (zQ175) and WT mice in comparison to Langfelder 2016 data. g, MA-plot of differential gene expression between zQ175:CK2α’(+/-) and WT mice. h, Venn Diagram of differentially expressed genes between zQ175, WT, zQ175:CK2α’(+/-) and CK2α’(+/-) mice. i, Driver factors of gene expression variance (genotype and/or gender) for the DGEs identified between zQ175 and zQ175:CK2α’(+/-) were evaluated using R package variance Partition. [file 40478_2022_1379_MOESM8_ESM.pdf]

# Additional file 10

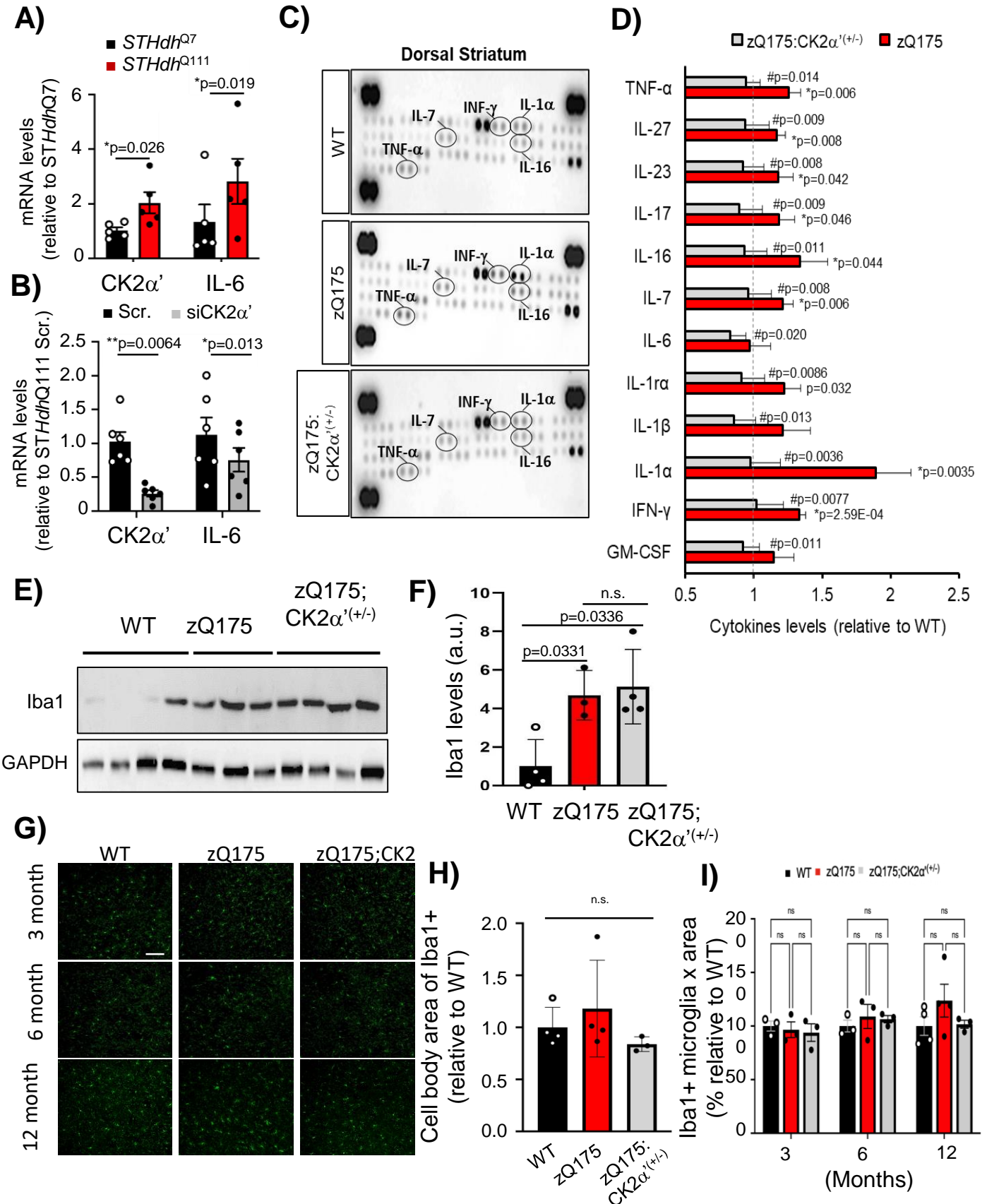

Supplement: Supplementary file 10 — Additional file 10. Alteration in CK2α’ expression changes cytokine profiles but not microglia. a, RT-qPCR analysis for CK2α’ and IL-6 in STHdhQ7/Q7 (control) and STHdhQ111/Q111 (HD) cell (n = 5 independent experiments). Data were normalized to GAPDH and control cells. b, siRNA knockdown of CK2α’ for 24 h in STHdhQ111/Q111 cells and RT-qPCR. Data were normalized with GAPDH and relativized to non-targeting control siRNA-treated cells (scramble), (n = 6 independent experiments). c, d, Representative images of mouse cytokine array panels from striatum extracts of WT, zQ175, and zQ175:CK2α’(+/-) at 12-14 months of age (n = 6 mice/genotype). e, f, Iba1 immunoblotting in the striatum of 12 months old WT, zQ175 and zQ175:CK2α’, quantification was measured by image analyses using Image J software, GAPDH is used as a loading marker. g-i, Representative images show the labeling of Iba1 in the dorsal striatum of WT, zQ175 and zQ175:CK2α’(+/-) at 3, 6 and 12 months old (g). Images were analyzed using the Image J software. Scale bar, 100 µm. Iba1+ cell body area in 12 months old mice (n = 3-4 mice/genotype, 18 images averaged/mouse) (h) and percent of Iba1+ cells in 300mm2 (n=3-4 mice/genotype, at least 9 images averaged/mouse) (i). Error bars denote mean ± SEM, values were analyzed by Student's t test in d, and one-way ANOVA and Tukey post-hoc test in a, b, f, h, i. *p represent p-values comparing zQ175 and WT, #p are p-values comparing zQ175 and zQ175:CK2α’(+/-). [file 40478_2022_1379_MOESM10_ESM.pdf]

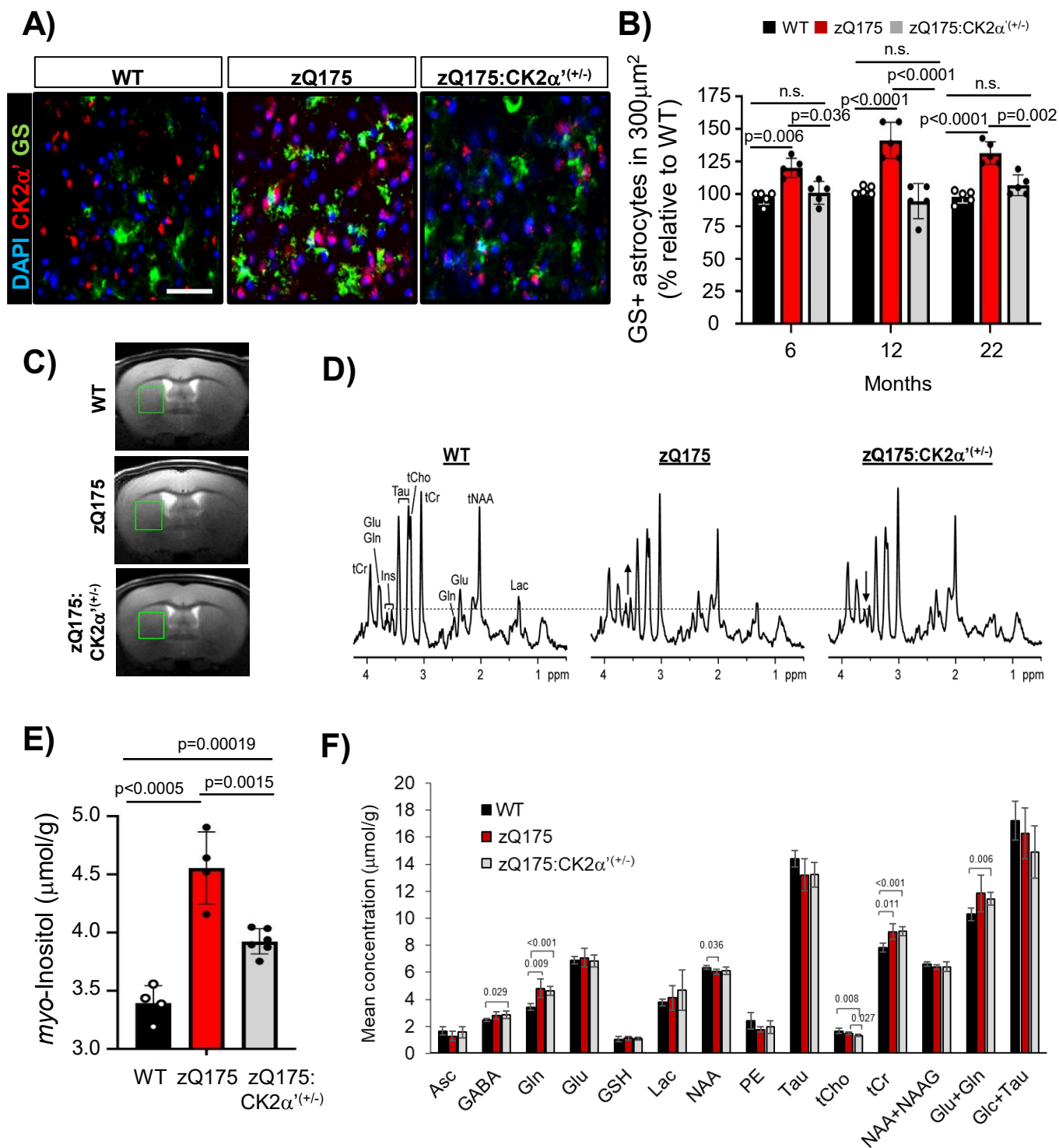

Supplement: Supplementary file 13 — Additional file 13. Decreased CK2α’ ameliorated astrogliosis in zQ175 mice. a, b, Representative images show the labeling (a) and quantification (b) of Glutamine synthetase (GS, an astrocytic marker) and CK2α’ of striatum sections from 12-month-old mice in WT, zQ175 and zQ175:CK2α’(+/-) (n = 5 mice/genotype, at least 3 images averaged/mouse). DAPI is used for nuclear staining. Scale bars, 50 µm. c, Coronal images of brain scans in 9.4T magnet showing the striatum voxel (green box) for MRS acquisition from each genotype at 22 months. d, Localized proton magnetic resonance spectra [LASER sequence, TE = 15 ms, TR= 5 s, 256 transients, 9.4T] obtained at 22 months of age from the striatum of WT, zQ175, and zQ175:CK2α’(+/-) mice (n = 4 WT; 4 HD; 6 HD: CK2α’ (+/-)). Differences in myo-inositol (Ins) between WT and zQ175 mice and between zQ175 and zQ175:CK2α’(+/-) mice are shown with arrows. e, f, Mean concentrations of myo-inositol (Ins) (e) and other reliably quantified metabolites (f) in the striatum of WT (black), zQ175 (red), and zQ175:CK2α’(+/-) (gray) mice. Asc: Ascorbate/vitamin C; tCr: total creatine + phosphocreatine; GABA: gamma-aminobutyric acid; Glc: glucose; Gln: glutamine; Glu: glutamate; tCho: total phosphocholine + glycerophosphocholine; GSH: glutathione; lns: myo-inositol; Lac: lactate; NAA: N-acetylaspartate; NAAG: N-acetylaspartylglutamate; PE: phosphoethanolamine; Tau: taurine. Error bars denote mean ± SEM, values were analyzed by one-way ANOVA with Tukey’s post-hoc test. p-values < 0.05 are indicated. [file 40478_2022_1379_MOESM13_ESM.pdf]

A)

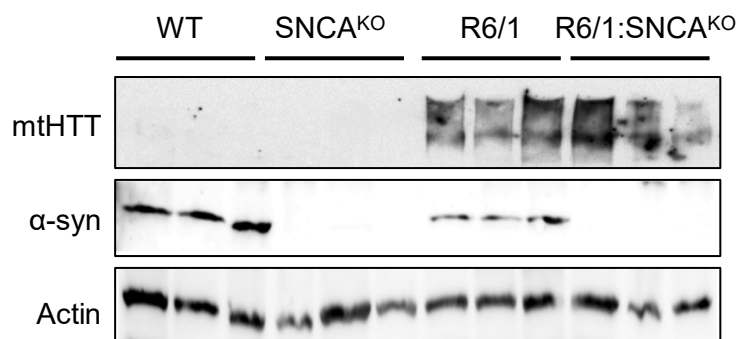

B)

SNCA

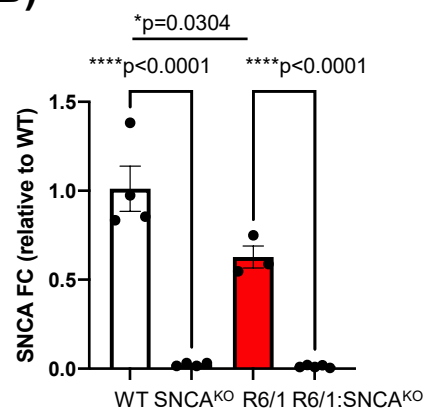

NRP2

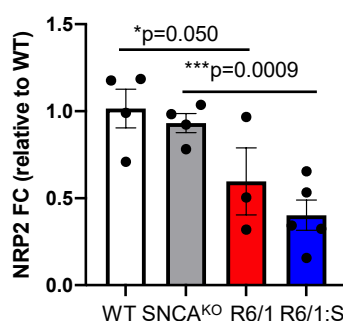

Grm2

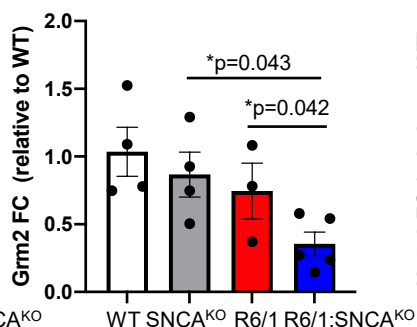

Nr4a2

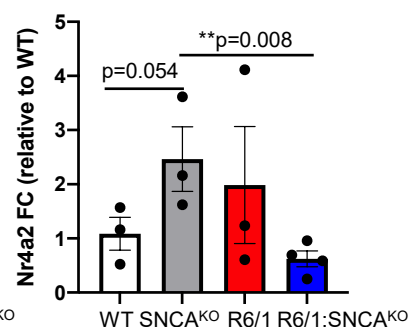

Slc30a3

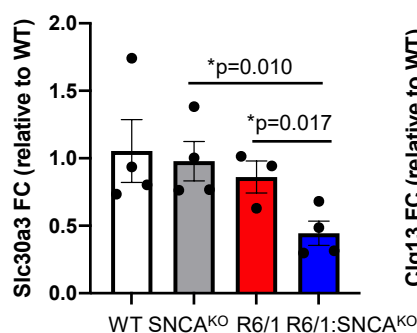

C1ql3

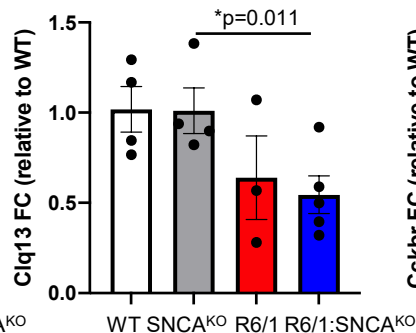

cckbr

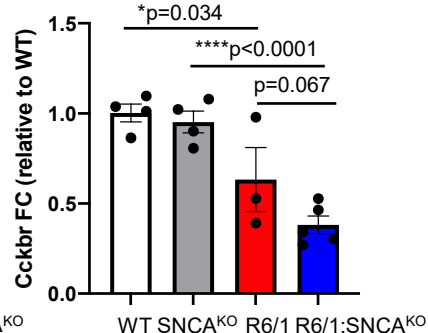

Ttr

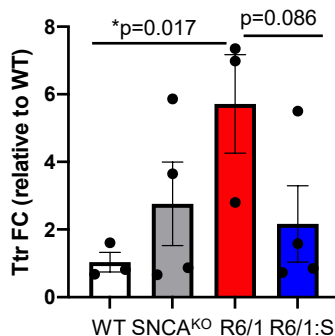

tbr

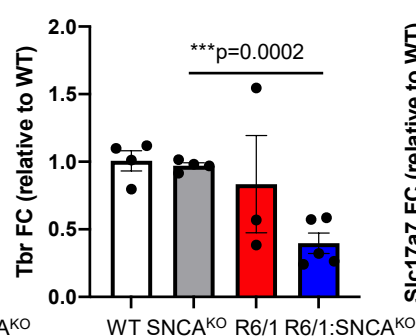

slc17a7

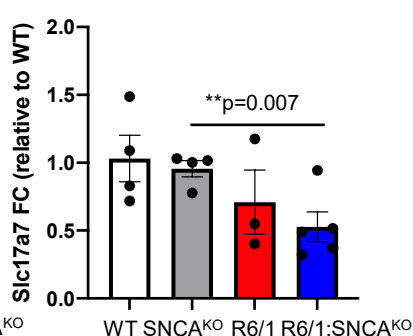

Supplement: Supplementary file 15 — Additional file 15. SNCA regulates the expression of synaptic genes identified in IPA analysis in R6/1 mice. a, IB for α-syn (4D6 antibody) and mtHTT (EM48 antibody) in the striatum of 5 months old WT, SNCAKO, R6/1 and R6/1:SNCAKO (n=3 mice/genotype). b, RT-qPCR analyses for SNCA and genes identified in the IPA analyses to be connected to SNCA; (Ttr, Grm2, Slc17a7, Slc30a3, Cckbr, Nrp2, Tbr1 and Nr4a2) (n = 4-5 mice/genotype). Error bars represent mean ± SEM. Statistical analyses were conducted by Student’s t-test. p-values <0.05 are indicated. We also indicated p-values <0.09 for those genes that showed a trend toward decreased expression. [file 40478_2022_1379_MOESM15_ESM.pdf]

# Additional file 16

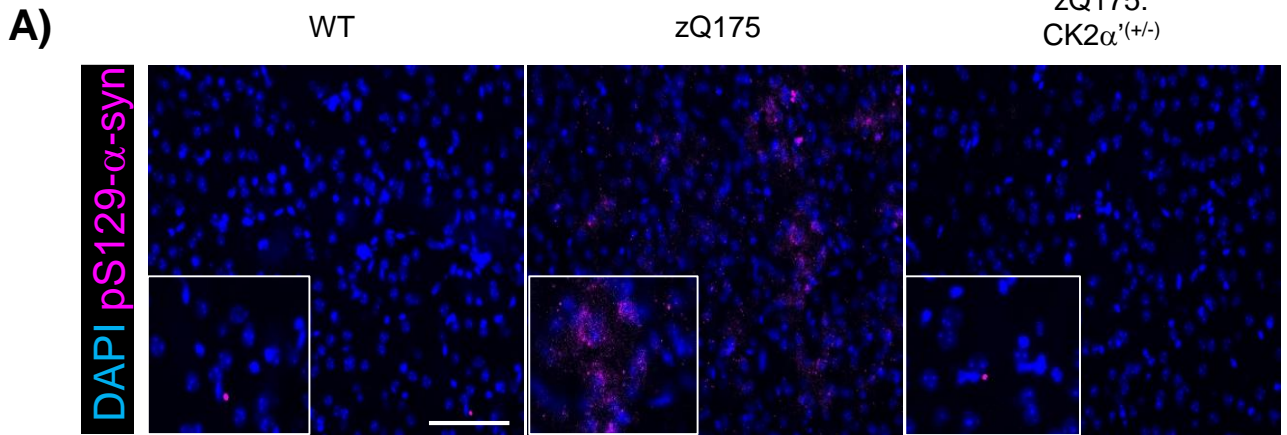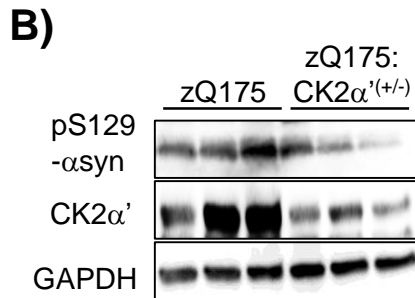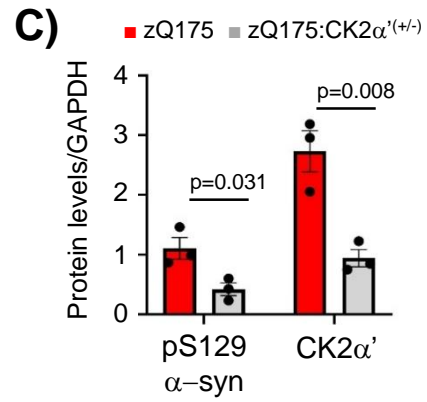

Supplement: Supplementary file 16 — Additional file 16. CK2a’ haploinsufficiency decreased the levels of pS129-α-syn. a, Representative pS129-α-syn IF images (D1R1R antibody) in the dorsal striatum of 12 months old WT, zQ175 and zQ175:CK2α’(+/-), Scale bar, 20 μm. b, pS129-α-syn and CK2α’ immunoblotting of striatum samples from 12-month-old zQ175 and zQ175:CK2α’(+/-) mice (n = 3 mice/group). c, Levels of pS129-α-syn and CK2α’ were calculated using Image J from immunoblotting images in a and showed a parallel decrease of pS129-α-syn and CK2α’ levels in zQ175:CK2α’(+/-) compared to zQ175 mice. Error bars represent mean ± SEM. Statistical analyses were conducted by Student’s t-test. p-values <0.05 are indicated (n=3 mice/genotype). [file 40478_2022_1379_MOESM16_ESM.pdf]
