# Supplementary material for: CK2 alpha prime and alpha-synuclein pathogenic functional interaction mediates synaptic dysregulation in huntington’s disease
Source: Acta Neuropathol Commun. 2022 Jun 3;10:83. doi: 10.1186/s40478-022-01379-8 (PMC9164558; doi:10.1186/s40478-022-01379-8)
Supplement: Supplementary file 6 — Additional file 6. WGCNA modules genes assignment. [file 40478_2022_1379_MOESM6_ESM.pdf]

Table S1. WGCNA Modules Genes						
EnsemblID	GeneSymbol	WGCNAmodule		EnsemblID	GeneSymbol	WGCNAmodu
ENSMUSG00000036469	43891	19		ENSMUSG00000047557	Lxn	20
ENSMUSG00000073481	43892	19		ENSMUSG00000075602	Ly6a	1
ENSMUSG00000079557	43892	9		ENSMUSG00000079018	Ly6c1	1
ENSMUSG00000032656	43893	9		ENSMUSG00000022587	Ly6e	5
ENSMUSG00000039372	43894	9		ENSMUSG00000073413	Ly6g6d	20
ENSMUSG00000039100	43896	2		ENSMUSG00000034923	Ly6g6f	3
ENSMUSG00000026977	43897	4		ENSMUSG00000022577	Ly6h	20
ENSMUSG00000025702	43898	9		ENSMUSG00000026980	Ly75	20
ENSMUSG00000040502	43899	20		ENSMUSG00000021423	Ly86	18
ENSMUSG00000078627	43900	20		ENSMUSG00000004707	Ly9	20
ENSMUSG00000022269	43901	18		ENSMUSG00000025779	Ly96	20
ENSMUSG00000000486	44075	20		ENSMUSG00000067367	Lyar	20
ENSMUSG00000026276	44076	20		ENSMUSG00000034041	Lyl1	12
ENSMUSG00000022456	44077	19		ENSMUSG00000042228	Lyn	20
ENSMUSG00000020486	44078	16		ENSMUSG00000022594	Lynx1	18
ENSMUSG00000072214	44079	9		ENSMUSG00000026344	Lypd1	20
ENSMUSG00000050379	44080	19		ENSMUSG00000050447	Lypd6	20
ENSMUSG00000001833	44081	1		ENSMUSG00000026765	Lypd6b	17
ENSMUSG00000018398	44082	20		ENSMUSG00000025903	Lypla1	9
ENSMUSG00000059248	44083	20		ENSMUSG00000028670	Lypla2	16
ENSMUSG00000019917	44084	20		ENSMUSG00000039246	Lyplal1	20
ENSMUSG00000058013	44085	9		ENSMUSG00000030922	Lyrm1	20
ENSMUSG00000007777	0610009B22Rik	20		ENSMUSG00000045854	Lyrm2	20
ENSMUSG00000086714	0610009E02Rik	20		ENSMUSG00000046573	Lyrm4	9
ENSMUSG00000043644	0610009L18Rik	20		ENSMUSG00000020268	Lyrm7	1
ENSMUSG00000042208	0610010F05Rik	9		ENSMUSG00000072640	Lyrm9	20
ENSMUSG00000020831	0610010K14Rik	20		ENSMUSG00000053769	Lysmd1	20
ENSMUSG00000107002	0610012G03Rik	12		ENSMUSG00000032184	Lysmd2	20
ENSMUSG00000097882	0610038B21Rik	20		ENSMUSG00000035840	Lysmd3	20
ENSMUSG00000089889	0610040B10Rik	20		ENSMUSG00000043831	Lysmd4	20
ENSMUSG00000087341	0610040F04Rik	20		ENSMUSG00000019726	Lyst	9
ENSMUSG00000060512	0610040J01Rik	20		ENSMUSG00000030787	Lyve1	20
ENSMUSG00000087361	0610043K17Rik	20		ENSMUSG00000069516	Lyz2	20
ENSMUSG00000090066	1110002E22Rik	5		ENSMUSG00000032530	Lyzl4	20
ENSMUSG00000097134	1110002J07Rik	20		ENSMUSG00000028990	Lzic	1
ENSMUSG00000071456	1110002L01Rik	19		ENSMUSG00000025245	Lztfl1	2
ENSMUSG00000102531	1110002O04Rik	20		ENSMUSG00000022761	Lztr1	20
ENSMUSG00000106291	1110003F10Rik	20		ENSMUSG00000036306	Lzts1	9
ENSMUSG00000030663	1110004F10Rik	3		ENSMUSG00000035342	Lzts2	20
ENSMUSG00000039195	1110008P14Rik	12		ENSMUSG00000037703	Lzts3	9
ENSMUSG00000045237	1110012L19Rik	20		ENSMUSG00000055125	M5C1000I18R	5
ENSMUSG00000116164	1110013H19Rik	20		ENSMUSG00000007458	M6pr	20
ENSMUSG00000098659	1110015O18Rik	20		ENSMUSG00000080832	M6pr-ps	20
ENSMUSG00000028441	1110017D15Rik	12		ENSMUSG00000022805	Maats1	14
ENSMUSG00000085151	1110018N20Rik	6		ENSMUSG00000056947	Mab21l1	6
ENSMUSG00000097616	1110019D14Rik	19		ENSMUSG00000028649	Macf1	19
ENSMUSG00000097047	1110020A21Rik	12		ENSMUSG00000028826	Maco1	20
ENSMUSG00000103520	1110025M09Rik	20		ENSMUSG00000036278	Macrod1	20
ENSMUSG00000085972	1110028F11Rik	20		ENSMUSG00000068205	Macrod2	12
ENSMUSG00000037971	1110032A03Rik	20		ENSMUSG00000074763	Macrod2os2	20
ENSMUSG00000046999	1110032F04Rik	20		ENSMUSG00000029554	Mad1l1	20
ENSMUSG00000092203	1110038B12Rik	10		ENSMUSG00000029910	Mad2l1	12

ENSMUSG00000063236	1110038F14Rik	19		ENSMUSG00000034509	Mad2l1bp	20
ENSMUSG00000085457	1110046J04Rik	20		ENSMUSG00000029003	Mad2l2	20
ENSMUSG00000040591	1110051M20Rik	20		ENSMUSG00000040687	Madd	20
ENSMUSG00000035171	1110059E24Rik	20		ENSMUSG00000079562	Maea	20
ENSMUSG00000032551	1110059G10Rik	19		ENSMUSG00000055435	Maf	20
ENSMUSG00000078570	1110065P20Rik	20		ENSMUSG00000022553	Maf1	20
ENSMUSG00000043687	1190005I06Rik	3		ENSMUSG00000047591	Mafa	9
ENSMUSG00000063320	1190007I07Rik	10		ENSMUSG00000074622	Mafb	20
ENSMUSG00000097290	1300002E11Rik	20		ENSMUSG00000042622	Maff	20
ENSMUSG00000112545	1300014J16Rik	20		ENSMUSG00000051510	Mafg	20
ENSMUSG00000096221	1500002C15Rik	20		ENSMUSG00000018143	Mafk	20
ENSMUSG00000097595	1500002F19Rik	20		ENSMUSG00000036634	Mag	16
ENSMUSG00000098912	1500004A13Rik	20		ENSMUSG00000082106	Mageb16-ps1	20
ENSMUSG00000068099	1500009C09Rik	20		ENSMUSG00000025151	Maged1	9
ENSMUSG00000087651	1500009L16Rik	9		ENSMUSG00000025268	Maged2	9
ENSMUSG00000072694	1500011B03Rik	19		ENSMUSG00000031227	Magee1	19
ENSMUSG00000098702	1500015A07Rik	20		ENSMUSG00000031224	Magee2	20
ENSMUSG00000094732	1500015L24Rik	20		ENSMUSG00000116632	Magef1	20
ENSMUSG00000097383	1500026H17Rik	20		ENSMUSG00000047238	Mageh1	20
ENSMUSG00000059631	1500035N22Rik	1		ENSMUSG00000056972	Magel2	20
ENSMUSG00000035595	1600002K03Rik	20		ENSMUSG00000045095	Magi1	20
ENSMUSG00000050088	1600012H06Rik	13		ENSMUSG00000040003	Magi2	6
ENSMUSG00000054676	1600014C10Rik	12		ENSMUSG00000052539	Magi3	3
ENSMUSG00000097048	1600020E01Rik	18		ENSMUSG00000028609	Magoh	12
ENSMUSG00000104986	1600023N17Rik	18		ENSMUSG00000030188	Magohb	12
ENSMUSG00000057818	1600029O15Rik	2		ENSMUSG00000031232	Magt1	20
ENSMUSG00000085984	1700001G11Rik	20		ENSMUSG00000025971	Maip1	20
ENSMUSG00000075511	1700001L05Rik	20		ENSMUSG00000021363	Mak	20
ENSMUSG00000021534	1700001L19Rik	20		ENSMUSG00000031578	Mak16	9
ENSMUSG00000044320	1700001O22Rik	20		ENSMUSG00000027375	Mal	16
ENSMUSG00000085944	1700003D09Rik	9		ENSMUSG00000024479	Mal2	20
ENSMUSG00000030030	1700003E16Rik	20		ENSMUSG00000092341	Malat1	9
ENSMUSG00000038523	1700003F12Rik	20		ENSMUSG00000027377	Mall	20
ENSMUSG00000085389	1700003M07Rik	20		ENSMUSG00000075520	Malrd1	20
ENSMUSG00000100274	1700006F04Rik	20		ENSMUSG00000029815	Malsu1	2
ENSMUSG00000101234	1700007E05Rik	20		ENSMUSG00000032688	Malt1	20
ENSMUSG00000100666	1700007F19Rik	20		ENSMUSG00000033207	Mamdc2	20
ENSMUSG00000026831	1700007K13Rik	13		ENSMUSG00000026941	Mamdc4	20
ENSMUSG00000097318	1700007L15Rik	20		ENSMUSG00000050567	Maml1	20
ENSMUSG00000103452	1700008A23Rik	20		ENSMUSG00000031925	Maml2	20
ENSMUSG00000101225	1700008J07Rik	17		ENSMUSG00000061143	Maml3	1
ENSMUSG00000008028	1700008O03Rik	20		ENSMUSG00000059401	Mamld1	1
ENSMUSG00000096573	1700009J07Rik	20		ENSMUSG00000042918	Mamstr	20
ENSMUSG00000023873	1700010I14Rik	9		ENSMUSG00000003746	Man1a	9
ENSMUSG00000031927	1700012B09Rik	14		ENSMUSG00000008763	Man1a2	20
ENSMUSG00000110424	1700012D14Rik	20		ENSMUSG00000036646	Man1b1	20
ENSMUSG00000105023	1700012D16Rik	20		ENSMUSG00000037306	Man1c1	20
ENSMUSG00000027886	1700013F07Rik	20		ENSMUSG00000024085	Man2a1	20
ENSMUSG00000051054	1700014D04Rik	20		ENSMUSG00000038886	Man2a2	9
ENSMUSG00000104908	1700015C17Rik	20		ENSMUSG00000005142	Man2b1	20
ENSMUSG00000102737	1700016A09Rik	20		ENSMUSG00000029119	Man2b2	12
ENSMUSG00000108979	1700016B15Rik	20		ENSMUSG00000032295	Man2c1	20
ENSMUSG00000053783	1700016K19Rik	14		ENSMUSG00000086728	Man2c1os	1
ENSMUSG00000085609	1700016P03Rik	20		ENSMUSG00000028164	Manba	20

ENSMUSG00000032300	1700017B05Rik	20		ENSMUSG00000063019	Manbal	1
ENSMUSG00000038408	1700018A04Rik	20		ENSMUSG00000040520	Manea	20
ENSMUSG00000100075	1700018L02Rik	20		ENSMUSG00000042763	Maneal	20
ENSMUSG00000060715	1700019A02Rik	20		ENSMUSG00000032575	Manf	20
ENSMUSG00000043629	1700019D03Rik	18		ENSMUSG00000032718	Mansc1	20
ENSMUSG00000100937	1700020D05Rik	20		ENSMUSG00000025037	Maoa	18
ENSMUSG00000035085	1700020L24Rik	20		ENSMUSG00000040147	Maob	20
ENSMUSG00000019797	1700021F05Rik	20		ENSMUSG00000050930	Map10	20
ENSMUSG00000117050	1700023B13Rik	20		ENSMUSG00000036948	Map11	20
ENSMUSG00000020940	1700023F06Rik	20		ENSMUSG00000027254	Map1a	20
ENSMUSG00000032666	1700025G04Rik	1		ENSMUSG00000052727	Map1b	19
ENSMUSG00000097321	1700028E10Rik	20		ENSMUSG00000027602	Map1lc3a	5
ENSMUSG00000038782	1700028J19Rik	20		ENSMUSG00000031812	Map1lc3b	2
ENSMUSG00000089798	1700028K03Rik	20		ENSMUSG00000019261	Map1s	20
ENSMUSG00000078635	1700028N14Rik	20		ENSMUSG00000015222	Map2	4
ENSMUSG00000033053	1700028P14Rik	14		ENSMUSG00000004936	Map2k1	18
ENSMUSG00000031452	1700029H14Rik	2		ENSMUSG00000035027	Map2k2	20
ENSMUSG00000044916	1700029I15Rik	13		ENSMUSG00000018932	Map2k3	20
ENSMUSG00000071103	1700029J07Rik	9		ENSMUSG00000042549	Map2k3os	20
ENSMUSG00000099759	1700030C10Rik	20		ENSMUSG00000033352	Map2k4	19
ENSMUSG00000091071	1700030C10Rik	20		ENSMUSG00000058444	Map2k5	20
ENSMUSG00000099869	1700030F04Rik	20		ENSMUSG00000020623	Map2k6	20
ENSMUSG00000031847	1700030J22Rik	9		ENSMUSG00000002948	Map2k7	20
ENSMUSG00000052794	1700030K09Rik	20		ENSMUSG00000021754	Map3k1	18
ENSMUSG00000097893	1700034P13Rik	20		ENSMUSG00000040390	Map3k10	9
ENSMUSG00000095956	1700036A12Rik	20		ENSMUSG00000004054	Map3k11	20
ENSMUSG00000005983	1700037C18Rik	20		ENSMUSG00000023050	Map3k12	20
ENSMUSG00000027327	1700037H04Rik	3		ENSMUSG00000033618	Map3k13	20
ENSMUSG00000097515	1700040D17Rik	20		ENSMUSG00000020941	Map3k14	9
ENSMUSG00000054822	1700041G16Rik	20		ENSMUSG00000031303	Map3k15	13
ENSMUSG00000094103	1700047I17Rik2	20		ENSMUSG00000051590	Map3k19	13
ENSMUSG00000100147	1700047M11Rik	20		ENSMUSG00000024383	Map3k2	2
ENSMUSG00000043773	1700048O20Rik	9		ENSMUSG00000004085	Map3k20	19
ENSMUSG00000099681	1700052K11Rik	20		ENSMUSG00000031853	Map3k21	9
ENSMUSG00000070997	1700055D18Rik	20		ENSMUSG00000020700	Map3k3	9
ENSMUSG00000084792	1700056N10Rik	20		ENSMUSG00000014426	Map3k4	20
ENSMUSG00000100837	1700063D05Rik	20		ENSMUSG00000071369	Map3k5	18
ENSMUSG00000099266	1700063H06Rik	20		ENSMUSG00000028862	Map3k6	20
ENSMUSG00000038323	1700066M21Rik	1		ENSMUSG00000028284	Map3k7	1
ENSMUSG00000080980	1700071K01Rik	20		ENSMUSG00000025610	Map3k7cl	20
ENSMUSG00000084859	1700080N15Rik	20		ENSMUSG00000024235	Map3k8	20
ENSMUSG00000113209	1700081N11Rik	3		ENSMUSG00000042724	Map3k9	18
ENSMUSG00000086016	1700084C06Rik	20		ENSMUSG00000032479	Map4	9
ENSMUSG00000100747	1700084E18Rik	20		ENSMUSG00000037337	Map4k1	2
ENSMUSG00000020548	1700086D15Rik	19		ENSMUSG00000024242	Map4k3	5
ENSMUSG00000071265	1700086L19Rik	9		ENSMUSG00000026074	Map4k4	20
ENSMUSG00000097080	1700086O06Rik	18		ENSMUSG00000034761	Map4k5	20
ENSMUSG00000115099	1700087I21Rik	18		ENSMUSG00000055407	Map6	18
ENSMUSG00000033029	1700088E04Rik	18		ENSMUSG00000041205	Map6d1	16
ENSMUSG00000117289	1700093J21Rik	20		ENSMUSG00000019996	Map7	9
ENSMUSG00000020286	1700093K21Rik	20		ENSMUSG00000028849	Map7d1	9
ENSMUSG00000118506	1700094D03Rik	20		ENSMUSG00000041020	Map7d2	9
ENSMUSG00000105337	1700094M23Rik	20		ENSMUSG00000033900	Map9	20
ENSMUSG00000085407	1700095J03Rik	20		ENSMUSG00000063358	Mapk1	9

ENSMUSG00000101856	1700096K18Rik	20		ENSMUSG00000046709	Mapk10	3
ENSMUSG00000097675	1700101I11Rik	20		ENSMUSG00000053137	Mapk11	19
ENSMUSG00000116852	1700102H20Rik	20		ENSMUSG00000022610	Mapk12	6
ENSMUSG00000032611	1700102P08Rik	20		ENSMUSG00000053436	Mapk14	20
ENSMUSG00000099923	1700105P06Rik	20		ENSMUSG00000063704	Mapk15	14
ENSMUSG00000008307	1700109H08Rik	20		ENSMUSG00000041775	Mapk1ip1	9
ENSMUSG00000099384	1700110C19Rik	20		ENSMUSG00000021840	Mapk1ip1l	20
ENSMUSG00000091329	1700112D23Rik	20		ENSMUSG00000063065	Mapk3	2
ENSMUSG00000097527	1700112J16Rik	20		ENSMUSG00000024558	Mapk4	9
ENSMUSG00000096935	1700113A16Rik	20		ENSMUSG00000042688	Mapk6	20
ENSMUSG00000082488	1700119I11Rik	20		ENSMUSG00000001034	Mapk7	20
ENSMUSG00000100599	1700120C14Rik	13		ENSMUSG00000021936	Mapk8	3
ENSMUSG00000086275	1700121C08Rik	1		ENSMUSG00000027223	Mapk8ip1	20
ENSMUSG00000085614	1700123M08Rik	20		ENSMUSG00000022619	Mapk8ip2	9
ENSMUSG00000040822	1700123O20Rik	1		ENSMUSG00000024163	Mapk8ip3	19
ENSMUSG00000101481	1700123O21Rik	20		ENSMUSG00000020366	Mapk9	3
ENSMUSG00000099354	1700124L16Rik	20		ENSMUSG00000038696	Mapkap1	9
ENSMUSG00000107802	1700126G02Rik	20		ENSMUSG00000016528	Mapkapk2	3
ENSMUSG00000071653	1810009A15Rik	20		ENSMUSG00000032577	Mapkapk3	20
ENSMUSG00000078607	1810010H24Rik	20		ENSMUSG00000029454	Mapkapk5	20
ENSMUSG00000022507	1810013L24Rik	3		ENSMUSG00000033902	Mapkbp1	9
ENSMUSG00000097412	1810014B01Rik	20		ENSMUSG00000027479	Mapre1	1
ENSMUSG00000097115	1810019N24Rik	20		ENSMUSG00000024277	Mapre2	20
ENSMUSG00000089997	1810020O05Rik	1		ENSMUSG00000029166	Mapre3	20
ENSMUSG00000087331	1810021B22Rik	20		ENSMUSG00000018411	Mapt	20
ENSMUSG00000044145	1810024B03Rik	20		ENSMUSG00000069662	Marcks	20
ENSMUSG00000101970	1810026B05Rik	20		ENSMUSG00000047945	Marcksl1	9
ENSMUSG00000044148	1810030O07Rik	20		ENSMUSG00000060657	Marf1	18
ENSMUSG00000054091	1810037I17Rik	3		ENSMUSG00000026620	Mark1	9
ENSMUSG00000100680	1810044D09Rik	20		ENSMUSG00000024969	Mark2	9
ENSMUSG00000035372	1810055G02Rik	20		ENSMUSG00000007411	Mark3	6
ENSMUSG00000073155	1810058I24Rik	9		ENSMUSG00000030397	Mark4	9
ENSMUSG00000108207	1810059H22Rik	20		ENSMUSG00000040354	Mars	20
ENSMUSG00000084925	1810062O18Rik	20		ENSMUSG00000046994	Mars2	3
ENSMUSG00000087165	2010001A14Rik	20		ENSMUSG00000044345	Marveld1	19
ENSMUSG00000051606	2010001K21Rik	14		ENSMUSG00000021636	Marveld2	20
ENSMUSG00000044694	2010007H06Rik	20		ENSMUSG00000001672	Marveld3	19
ENSMUSG00000029384	2010109A12Rik	20		ENSMUSG00000068037	Mas1	20
ENSMUSG00000063018	2010204K13Rik	20		ENSMUSG00000117942	Maskbp3	20
ENSMUSG00000026090	2010300C02Rik	9		ENSMUSG00000022887	Masp1	20
ENSMUSG00000101268	2010310C07Rik	20		ENSMUSG00000028979	Masp2	20
ENSMUSG00000074217	2210011C24Rik	12		ENSMUSG00000053693	Mast1	19
ENSMUSG00000021550	2210016F16Rik	20		ENSMUSG00000003810	Mast2	9
ENSMUSG00000029559	2210016L21Rik	20		ENSMUSG00000031833	Mast3	1
ENSMUSG00000039563	2210406O10Rik	20		ENSMUSG00000034751	Mast4	18
ENSMUSG00000087380	2210408F21Rik	20		ENSMUSG00000026779	Mastl	1
ENSMUSG00000071252	2210408I21Rik	20		ENSMUSG00000053907	Mat2a	20
ENSMUSG00000085563	2210411M09Rik	20		ENSMUSG00000042032	Mat2b	9
ENSMUSG00000107216	2210412B16Rik	10		ENSMUSG00000004933	Matk	20
ENSMUSG00000078894	2210418O10Rik	1		ENSMUSG00000040533	Matn1	20
ENSMUSG00000032403	2300009A05Rik	20		ENSMUSG00000022324	Matn2	18
ENSMUSG00000097354	2310001H17Rik	20		ENSMUSG00000016995	Matn4	20
ENSMUSG00000098332	2310009A05Rik	1		ENSMUSG00000037236	Matr3	2
ENSMUSG00000079283	2310009B15Rik	20		ENSMUSG00000031858	Mau2	20

ENSMUSG00000097162	2310010J17Rik	18		ENSMUSG00000037523	Mavs	20
ENSMUSG00000020133	2310011J03Rik	20		ENSMUSG00000059436	Max	18
ENSMUSG00000085945	2310014F06Rik	20		ENSMUSG00000030678	Maz	20
ENSMUSG00000097729	2310015A10Rik	20		ENSMUSG00000051065	Mb21d2	18
ENSMUSG00000049643	2310022A10Rik	20		ENSMUSG00000024561	Mbd1	20
ENSMUSG00000031983	2310022B05Rik	20		ENSMUSG00000024513	Mbd2	9
ENSMUSG00000097710	2310026I22Rik	20		ENSMUSG00000035478	Mbd3	3
ENSMUSG00000032062	2310030G06Rik	1		ENSMUSG00000030322	Mbd4	3
ENSMUSG00000020441	2310033P09Rik	20		ENSMUSG00000036792	Mbd5	7
ENSMUSG00000099655	2310034G01Rik	20		ENSMUSG00000025409	Mbd6	20
ENSMUSG00000062619	2310039H08Rik	20		ENSMUSG00000021028	Mbip	20
ENSMUSG00000072852	2310040G07Rik	20		ENSMUSG00000049285	Mblac1	20
ENSMUSG00000108732	2310043P16Rik	20		ENSMUSG00000051098	Mblac2	20
ENSMUSG00000040177	2310057M21Rik	19		ENSMUSG00000027763	Mbnl1	7
ENSMUSG00000085379	2310058D17Rik	20		ENSMUSG00000022139	Mbnl2	9
ENSMUSG00000050705	2310061I04Rik	9		ENSMUSG00000038732	Mboat1	20
ENSMUSG00000100084	2310068J16Rik	9		ENSMUSG00000020646	Mboat2	9
ENSMUSG00000089718	2310075C17Rik	1		ENSMUSG00000035596	Mboat7	20
ENSMUSG00000045411	2410002F23Rik	20		ENSMUSG00000041607	Mbp	16
ENSMUSG00000036873	2410004B18Rik	20		ENSMUSG00000059474	Mbtd1	20
ENSMUSG00000071398	2410004P03Rik	14		ENSMUSG00000031835	Mbtps1	9
ENSMUSG00000086841	2410006H16Rik	12		ENSMUSG00000046873	Mbtps2	20
ENSMUSG00000073164	2410018L13Rik	20		ENSMUSG00000038537	Mc3r	20
ENSMUSG00000100017	2410022M11Rik	20		ENSMUSG00000047259	Mc4r	20
ENSMUSG00000032840	2410131K14Rik	20		ENSMUSG00000032135	Mcam	16
ENSMUSG00000096917	2500002B13Rik	20		ENSMUSG00000048755	Mcat	20
ENSMUSG00000073236	2500004C02Rik	3		ENSMUSG00000071856	Mcc	19
ENSMUSG00000071632	2510002D24Rik	2		ENSMUSG00000027709	Mccc1	20
ENSMUSG00000043391	2510009E07Rik	9		ENSMUSG00000086392	Mccc1os	9
ENSMUSG00000106825	2510016D11Rik	1		ENSMUSG00000021646	Mccc2	20
ENSMUSG00000104891	2510017J16Rik	20		ENSMUSG00000033429	Mcee	20
ENSMUSG00000044496	2510039O18Rik	20		ENSMUSG00000013974	Mcemp1	20
ENSMUSG00000116797	2600002D14Rik	20		ENSMUSG00000031139	Mcf2	18
ENSMUSG00000100303	2600014E21Rik	19		ENSMUSG00000031442	Mcf2l	9
ENSMUSG00000103828	2610001A08Rik	6		ENSMUSG00000024150	Mcfd2	19
ENSMUSG00000052419	2610001J05Rik	20		ENSMUSG00000050164	Mchr1	9
ENSMUSG00000031242	2610002M06Rik	1		ENSMUSG00000074651	Mcidas	20
ENSMUSG00000071793	2610005L07Rik	20		ENSMUSG00000038612	Mcl1	1
ENSMUSG00000060301	2610008E11Rik	1		ENSMUSG00000026669	Mcm10	12
ENSMUSG00000102474	2610012C04Rik	20		ENSMUSG00000002870	Mcm2	20
ENSMUSG00000097537	2610020C07Rik	20		ENSMUSG00000041859	Mcm3	20
ENSMUSG00000091474	2610021A01Rik	10		ENSMUSG00000001150	Mcm3ap	9
ENSMUSG00000084362	2610024D14Rik	20		ENSMUSG00000022673	Mcm4	20
ENSMUSG00000110997	2610028D06Rik	20		ENSMUSG00000005410	Mcm5	18
ENSMUSG00000085562	2610028E06Rik	20		ENSMUSG00000026355	Mcm6	20
ENSMUSG00000087259	2610035D17Rik	20		ENSMUSG00000029730	Mcm7	20
ENSMUSG00000085555	2610035F20Rik	20		ENSMUSG00000027353	Mcm8	20
ENSMUSG00000097536	2610037D02Rik	10		ENSMUSG00000058298	Mcm9	2
ENSMUSG00000071302	2610044O15Rik8	20		ENSMUSG00000048170	Mcmbp	1
ENSMUSG00000079671	2610203C22Rik	20		ENSMUSG00000046101	Mcmdc2	20
ENSMUSG00000059482	2610301B20Rik	20		ENSMUSG00000004567	Mcoln1	2
ENSMUSG00000100164	2610306M01Rik	20		ENSMUSG00000039842	Mcph1	1
ENSMUSG00000108614	2610306O10Rik	20		ENSMUSG00000061111	Mcrip1	10
ENSMUSG00000085936	2610307P16Rik	20		ENSMUSG00000025732	Mcrip2	20

ENSMUSG00000097040	2610316D01Rik	20		ENSMUSG00000037570	Mcrs1	20
ENSMUSG00000049916	2610318N02Rik	20		ENSMUSG00000021596	Mctp1	9
ENSMUSG00000010277	2610507B11Rik	18		ENSMUSG00000032776	Mctp2	18
ENSMUSG00000085882	2610507I01Rik	19		ENSMUSG00000000355	Mcts1	20
ENSMUSG00000106691	2700029L08Rik	20		ENSMUSG00000042814	Mcts2	20
ENSMUSG00000086328	2700033N17Rik	20		ENSMUSG00000009647	Mcu	1
ENSMUSG00000097180	2700038G22Rik	20		ENSMUSG00000027994	Mcub	5
ENSMUSG00000041789	2700046A07Rik	20		ENSMUSG00000021371	Mcur1	4
ENSMUSG00000097787	2700046G09Rik	9		ENSMUSG00000061607	Mdc1	20
ENSMUSG00000034601	2700049A03Rik	20		ENSMUSG00000041390	Mdfic	13
ENSMUSG00000117042	2700054A10Rik	20		ENSMUSG00000043557	Mdga1	5
ENSMUSG00000024273	2700062C07Rik	20		ENSMUSG00000034912	Mdga2	20
ENSMUSG00000086172	2700068H02Rik	20		ENSMUSG00000020321	Mdh1	20
ENSMUSG00000103722	2700078F05Rik	9		ENSMUSG00000025963	Mdh1b	20
ENSMUSG00000053080	2700081O15Rik	4		ENSMUSG00000019179	Mdh2	12
ENSMUSG00000062198	2700097O09Rik	20		ENSMUSG00000027239	Mdk	1
ENSMUSG00000098090	2700099C18Rik	20		ENSMUSG00000020212	Mdm1	20
ENSMUSG00000087497	2810001G20Rik	12		ENSMUSG00000020184	Mdm2	20
ENSMUSG00000045464	2810002D19Rik	20		ENSMUSG00000054387	Mdm4	20
ENSMUSG00000031984	2810004N23Rik	3		ENSMUSG00000058006	Mdn1	3
ENSMUSG00000047635	2810006K23Rik	20		ENSMUSG00000002329	Mdp1	6
ENSMUSG00000099881	2810013P06Rik	20		ENSMUSG00000032418	Me1	18
ENSMUSG00000020491	2810021J22Rik	20		ENSMUSG00000024556	Me2	9
ENSMUSG00000049881	2810025M15Rik	10		ENSMUSG00000030621	Me3	6
ENSMUSG00000096995	2810029C07Rik	12		ENSMUSG00000002768	Mea1	12
ENSMUSG00000037735	2810032G03Rik	20		ENSMUSG00000028863	Meaf6	9
ENSMUSG00000099034	2810039B14Rik	20		ENSMUSG00000034105	Meak7	18
ENSMUSG00000100891	2810049E08Rik	20		ENSMUSG00000027684	Mecom	20
ENSMUSG00000099966	2810402E24Rik	20		ENSMUSG00000031393	Mecp2	3
ENSMUSG00000086629	2810403D21Rik	20		ENSMUSG00000028910	Mecr	20
ENSMUSG00000101493	2810405F17Rik	20		ENSMUSG00000018160	Med1	7
ENSMUSG00000055972	2810407A14Rik	20		ENSMUSG00000021598	Med10	9
ENSMUSG00000075389	2810410L24Rik	20		ENSMUSG00000018923	Med11	20
ENSMUSG00000100594	2810414N06Rik	20		ENSMUSG00000079487	Med12	7
ENSMUSG00000105981	2810428J06Rik	20		ENSMUSG00000056476	Med12l	19
ENSMUSG00000097709	2810429I04Rik	20		ENSMUSG00000034297	Med13	2
ENSMUSG00000086283	2810433D01Rik	10		ENSMUSG00000018076	Med13l	9
ENSMUSG00000100876	2810454H06Rik	20		ENSMUSG00000064127	Med14	20
ENSMUSG00000085030	2810455O05Rik	9		ENSMUSG00000012114	Med15	20
ENSMUSG00000026227	2810459M11Rik	20		ENSMUSG00000013833	Med16	19
ENSMUSG00000091475	2810468N07Rik	20		ENSMUSG00000031935	Med17	20
ENSMUSG00000103004	2900002M20Rik	20		ENSMUSG00000066042	Med18	20
ENSMUSG00000043833	2900005J15Rik	20		ENSMUSG00000027080	Med19	20
ENSMUSG00000102014	2900009J06Rik	1		ENSMUSG00000092558	Med20	18
ENSMUSG00000104244	2900018N21Rik	20		ENSMUSG00000030291	Med21	20
ENSMUSG00000102386	2900022M07Rik	20		ENSMUSG00000015776	Med22	10
ENSMUSG00000051339	2900026A02Rik	5		ENSMUSG00000019984	Med23	20
ENSMUSG00000109162	2900027M19Rik	3		ENSMUSG00000017210	Med24	18
ENSMUSG00000103776	2900035J10Rik	20		ENSMUSG00000002968	Med25	9
ENSMUSG00000103663	2900037B21Rik	2		ENSMUSG00000045248	Med26	20
ENSMUSG00000115625	2900040C04Rik	13		ENSMUSG00000026799	Med27	6
ENSMUSG00000111938	2900045O20Rik	20		ENSMUSG00000015804	Med28	12
ENSMUSG00000043993	2900052L18Rik	20		ENSMUSG00000003444	Med29	20
ENSMUSG00000099696	2900052N01Rik	1		ENSMUSG00000038622	Med30	20

ENSMUSG00000113621	2900060N12Rik	20		ENSMUSG00000020801	Med31	12
ENSMUSG00000106746	2900064F13Rik	9		ENSMUSG00000022109	Med4	20
ENSMUSG00000105771	2900064K03Rik	19		ENSMUSG00000002679	Med6	1
ENSMUSG00000087018	2900072N19Rik	20		ENSMUSG00000020397	Med7	20
ENSMUSG00000097277	2900076A07Rik	20		ENSMUSG00000006392	Med8	10
ENSMUSG00000111358	2900078I11Rik	20		ENSMUSG00000061650	Med9	20
ENSMUSG00000087038	2900079G21Rik	20		ENSMUSG00000085260	Med9os	20
ENSMUSG00000087484	2900089D17Rik	20		ENSMUSG00000029659	Medag	18
ENSMUSG00000104034	2900092N22Rik	9		ENSMUSG00000030557	Mef2a	3
ENSMUSG00000099632	2900093K20Rik	20		ENSMUSG00000005583	Mef2c	9
ENSMUSG00000102869	2900097C17Rik	13		ENSMUSG00000001419	Mef2d	7
ENSMUSG00000070282	3000002C10Rik	20		ENSMUSG00000021268	Meg3	20
ENSMUSG00000084885	3010001F23Rik	2		ENSMUSG00000024593	Megf10	15
ENSMUSG00000079737	3110001I22Rik	20		ENSMUSG00000036466	Megf11	16
ENSMUSG00000114649	3110006O06Rik	10		ENSMUSG00000057751	Megf6	20
ENSMUSG00000026388	3110009E18Rik	20		ENSMUSG00000045039	Megf8	12
ENSMUSG00000094958	3110021N24Rik	20		ENSMUSG00000039270	Megf9	19
ENSMUSG00000104861	3110039M20Rik	18		ENSMUSG00000068117	Mei1	6
ENSMUSG00000025102	3110040N11Rik	20		ENSMUSG00000043289	Mei4	9
ENSMUSG00000085642	3110053B16Rik	20		ENSMUSG00000026650	Meig1	20
ENSMUSG00000085622	3110056K07Rik	20		ENSMUSG00000020160	Meis1	20
ENSMUSG00000103634	3110062G12Rik	20		ENSMUSG00000027210	Meis2	9
ENSMUSG00000081187	3110067C02Rik	20		ENSMUSG00000041420	Meis3	20
ENSMUSG00000074635	3110070M22Rik	13		ENSMUSG00000035683	Melk	1
ENSMUSG00000109625	3110080E11Rik	20		ENSMUSG00000058704	Memo1	20
ENSMUSG00000103284	3110080O07Rik	20		ENSMUSG00000001493	Meox1	20
ENSMUSG00000053553	3110082I17Rik	18		ENSMUSG00000029726	Mepce	20
ENSMUSG00000086815	3110082J24Rik	20		ENSMUSG00000014361	Mertk	19
ENSMUSG00000097303	3110083C13Rik	20		ENSMUSG00000038503	Mesd	20
ENSMUSG00000073590	3222401L13Rik	1		ENSMUSG00000051855	Mest	20
ENSMUSG00000033731	3300002A11Rik	14		ENSMUSG00000009376	Met	16
ENSMUSG00000063364	3300002I08Rik	20		ENSMUSG00000005813	Metap1	20
ENSMUSG00000071540	3425401B19Rik	13		ENSMUSG00000041921	Metap1d	20
ENSMUSG00000089759	3632454L22Rik	3		ENSMUSG00000036112	Metap2	19
ENSMUSG00000033111	3830406C13Rik	20		ENSMUSG00000002274	Metrn	20
ENSMUSG00000071181	3830408C21Rik	20		ENSMUSG00000039208	Metrnl	20
ENSMUSG00000106485	3830422I06Rik	9		ENSMUSG00000006732	Mettl1	20
ENSMUSG00000064202	4430402I18Rik	20		ENSMUSG00000040113	Mettl11b	20
ENSMUSG00000042579	4632404H12Rik	20		ENSMUSG00000028114	Mettl14	20
ENSMUSG00000097561	4632411P08Rik	20		ENSMUSG00000057234	Mettl15	20
ENSMUSG00000048106	4632415L05Rik	3		ENSMUSG00000010554	Mettl16	20
ENSMUSG00000111605	4632418H02Rik	20		ENSMUSG00000004561	Mettl17	20
ENSMUSG00000074024	4632427E13Rik	20		ENSMUSG00000041396	Mettl18	20
ENSMUSG00000097184	4632428C04Rik	8		ENSMUSG00000020691	Mettl2	9
ENSMUSG00000105597	4633401B06Rik	20		ENSMUSG00000025956	Mettl21a	20
ENSMUSG00000097004	4731419I09Rik	20		ENSMUSG00000039345	Mettl22	20
ENSMUSG00000086943	4732414G09Rik	20		ENSMUSG00000090266	Mettl23	12
ENSMUSG00000107402	4732416N19Rik	20		ENSMUSG00000045555	Mettl24	20
ENSMUSG00000090031	4732440D04Rik	20		ENSMUSG00000036009	Mettl25	20
ENSMUSG00000097762	4732463B04Rik	1		ENSMUSG00000025731	Mettl26	9
ENSMUSG00000053714	4732471J01Rik	20		ENSMUSG00000040557	Mettl27	20
ENSMUSG00000097350	4732491K20Rik	17		ENSMUSG00000022160	Mettl3	1
ENSMUSG00000108456	4732496C06Rik	10		ENSMUSG00000055660	Mettl4	9
ENSMUSG00000118132	4833408A19Rik	20		ENSMUSG00000051730	Mettl5	20

ENSMUSG00000109089	4833411C07Rik	20		ENSMUSG00000085735	Mettl5os	9
ENSMUSG00000116298	4833412C15Rik	20		ENSMUSG00000021891	Mettl6	20
ENSMUSG00000104018	4833412K13Rik	20		ENSMUSG00000054619	Mettl7a1	1
ENSMUSG00000104950	4833413G10Rik	3		ENSMUSG00000041975	Mettl8	20
ENSMUSG00000115936	4833415N18Rik	9		ENSMUSG00000030876	Mettl9	9
ENSMUSG00000086015	4833417C18Rik	20		ENSMUSG00000074480	Mex3a	20
ENSMUSG00000085287	4833418N02Rik	20		ENSMUSG00000057706	Mex3b	9
ENSMUSG00000062822	4833420G17Rik	20		ENSMUSG00000037253	Mex3c	20
ENSMUSG00000103996	4833421G17Rik	20		ENSMUSG00000048696	Mex3d	12
ENSMUSG00000108519	4833421K07Rik	9		ENSMUSG00000068479	Mfap1a	20
ENSMUSG00000074782	4833422C13Rik	5		ENSMUSG00000048222	Mfap1b	20
ENSMUSG00000097619	4833422M21Rik	20		ENSMUSG00000060572	Mfap2	20
ENSMUSG00000118087	4833438C02Rik	20		ENSMUSG00000020522	Mfap3	20
ENSMUSG00000025871	4833439L19Rik	20		ENSMUSG00000031647	Mfap3l	20
ENSMUSG00000103103	4833445I07Rik	20		ENSMUSG00000042436	Mfap4	20
ENSMUSG00000097626	4921504A21Rik	3		ENSMUSG00000026150	Mff	20
ENSMUSG00000029828	4921507P07Rik	20		ENSMUSG00000030605	Mfge8	3
ENSMUSG00000036934	4921524J17Rik	20		ENSMUSG00000070056	Mfhas1	19
ENSMUSG00000113211	4921525O09Rik	20		ENSMUSG00000027668	Mfn1	9
ENSMUSG00000085704	4921531C22Rik	20		ENSMUSG00000029020	Mfn2	20
ENSMUSG00000020434	4921536K21Rik	1		ENSMUSG00000018169	Mfng	20
ENSMUSG00000027309	4930402H24Rik	15		ENSMUSG00000034739	Mfrp	13
ENSMUSG00000115639	4930404K13Rik	9		ENSMUSG00000027775	Mfsd1	1
ENSMUSG00000086638	4930405A21Rik	20		ENSMUSG00000001082	Mfsd10	20
ENSMUSG00000090565	4930405O22Rik	20		ENSMUSG00000020818	Mfsd11	20
ENSMUSG00000085558	4930412C18Rik	20		ENSMUSG00000034854	Mfsd12	20
ENSMUSG00000110289	4930412F12Rik	20		ENSMUSG00000025227	Mfsd13a	9
ENSMUSG00000115526	4930413F20Rik	20		ENSMUSG00000089911	Mfsd14a	20
ENSMUSG00000108129	4930417O13Rik	20		ENSMUSG00000038212	Mfsd14b	12
ENSMUSG00000116885	4930420G21Rik	20		ENSMUSG00000028655	Mfsd2a	20
ENSMUSG00000043168	4930426D05Rik	20		ENSMUSG00000037336	Mfsd2b	20
ENSMUSG00000097323	4930426I24Rik	20		ENSMUSG00000019080	Mfsd3	20
ENSMUSG00000026736	4930426L09Rik	20		ENSMUSG00000059149	Mfsd4a	19
ENSMUSG00000086918	4930429F24Rik	20		ENSMUSG00000038522	Mfsd4b1	20
ENSMUSG00000046567	4930430F08Rik	20		ENSMUSG00000096687	Mfsd4b4	20
ENSMUSG00000109147	4930431P19Rik	20		ENSMUSG00000045665	Mfsd5	15
ENSMUSG00000097007	4930432B10Rik	20		ENSMUSG00000041439	Mfsd6	3
ENSMUSG00000008129	4930432K21Rik	20		ENSMUSG00000029490	Mfsd7a	20
ENSMUSG00000067795	4930444P10Rik	20		ENSMUSG00000041945	Mfsd9	1
ENSMUSG00000021098	4930447C04Rik	20		ENSMUSG00000033943	Mga	7
ENSMUSG00000113591	4930447K03Rik	20		ENSMUSG00000037161	Mgarp	18
ENSMUSG00000087674	4930447M23Rik	20		ENSMUSG00000020346	Mgat1	18
ENSMUSG00000106583	4930447N08Rik	9		ENSMUSG00000043998	Mgat2	20
ENSMUSG00000022543	4930451G09Rik	20		ENSMUSG00000042428	Mgat3	19
ENSMUSG00000021747	4930452B06Rik	6		ENSMUSG00000026110	Mgat4a	3
ENSMUSG00000059920	4930453N24Rik	20		ENSMUSG00000036620	Mgat4b	9
ENSMUSG00000102985	4930456G14Rik	20		ENSMUSG00000019888	Mgat4c	1
ENSMUSG00000087611	4930458D05Rik	20		ENSMUSG00000036155	Mgat5	18
ENSMUSG00000112332	4930466K18Rik	20		ENSMUSG00000043857	Mgat5b	9
ENSMUSG00000112484	4930473O22Rik	3		ENSMUSG00000033174	Mgll	20
ENSMUSG00000104999	4930477O15Rik	3		ENSMUSG00000027424	Mgme1	20
ENSMUSG00000085238	4930479D17Rik	20		ENSMUSG00000054612	Mgmt	20
ENSMUSG00000107567	4930480K02Rik	20		ENSMUSG00000030218	Mgp	1
ENSMUSG00000086938	4930481A15Rik	20		ENSMUSG00000022517	Mgrn1	20

ENSMUSG00000085956	4930481B07Rik	20		ENSMUSG00000008540	Mgst1	20
ENSMUSG00000097520	4930488L21Rik	1		ENSMUSG00000026688	Mgst3	9
ENSMUSG00000097592	4930500F10Rik	20		ENSMUSG00000097652	Mhrt	1
ENSMUSG00000044906	4930503L19Rik	20		ENSMUSG00000021000	Mia2	20
ENSMUSG00000040919	4930505A04Rik	20		ENSMUSG00000056050	Mia3	3
ENSMUSG00000087478	4930506C21Rik	20		ENSMUSG00000097767	Miat	19
ENSMUSG00000097129	4930507D05Rik	20		ENSMUSG00000024294	Mib1	7
ENSMUSG00000096959	4930509G22Rik	4		ENSMUSG00000029060	Mib2	3
ENSMUSG00000105659	4930509H03Rik	12		ENSMUSG00000019823	Mical1	9
ENSMUSG00000086607	4930511M06Rik	20		ENSMUSG00000038244	Mical2	5
ENSMUSG00000020992	4930512B01Rik	20		ENSMUSG00000051586	Mical3	20
ENSMUSG00000074136	4930513N10Rik	20		ENSMUSG00000033039	Micall1	16
ENSMUSG00000111246	4930517E14Rik	20		ENSMUSG00000036718	Micall2	18
ENSMUSG00000074629	4930518I15Rik	20		ENSMUSG00000050608	Micos10	19
ENSMUSG00000031325	4930519F16Rik	20		ENSMUSG00000049760	Micos13	3
ENSMUSG00000114953	4930519K11Rik	13		ENSMUSG00000020111	Micu1	6
ENSMUSG00000074039	4930520O04Rik	20		ENSMUSG00000021973	Micu2	20
ENSMUSG00000072762	4930522L14Rik	20		ENSMUSG00000039478	Micu3	3
ENSMUSG00000090394	4930523C07Rik	20		ENSMUSG00000035299	Mid1	20
ENSMUSG00000090207	4930524O07Rik	20		ENSMUSG00000095134	Mid1-ps1	20
ENSMUSG00000097276	4930525G20Rik	2		ENSMUSG00000008035	Mid1ip1	18
ENSMUSG00000085521	4930526A20Rik	20		ENSMUSG00000000266	Mid2	3
ENSMUSG00000097502	4930528D03Rik	20		ENSMUSG00000035621	Midn	10
ENSMUSG00000097261	4930532G15Rik	20		ENSMUSG00000022412	Mief1	20
ENSMUSG00000048905	4930539E08Rik	20		ENSMUSG00000018599	Mief2	20
ENSMUSG00000097032	4930539J05Rik	20		ENSMUSG00000002580	Mien1	12
ENSMUSG00000097890	4930547M16Rik	20		ENSMUSG00000028522	Mier1	12
ENSMUSG00000111165	4930553I21Rik	20		ENSMUSG00000042570	Mier2	20
ENSMUSG00000106791	4930553P18Rik	20		ENSMUSG00000032727	Mier3	2
ENSMUSG00000114190	4930556H04Rik	20		ENSMUSG00000033307	Mif	3
ENSMUSG00000034493	4930556J24Rik	20		ENSMUSG00000020743	Mif4gd	9
ENSMUSG00000096971	4930556M19Rik	13		ENSMUSG00000054942	Miga1	10
ENSMUSG00000086277	4930558K02Rik	9		ENSMUSG00000026858	Miga2	18
ENSMUSG00000053574	4930563E22Rik	13		ENSMUSG00000029022	Miip	20
ENSMUSG00000102408	4930568G15Rik	18		ENSMUSG00000040987	Mill2	20
ENSMUSG00000085084	4930570G19Rik	20		ENSMUSG00000039313	Minar1	18
ENSMUSG00000115725	4930572G02Rik	20		ENSMUSG00000050875	Minar2	20
ENSMUSG00000097587	4930578M01Rik	9		ENSMUSG00000038712	Mindy1	9
ENSMUSG00000027811	4930579G24Rik	20		ENSMUSG00000042444	Mindy2	18
ENSMUSG00000051074	4930579K19Rik	20		ENSMUSG00000026767	Mindy3	20
ENSMUSG00000070315	4930581F22Rik	20		ENSMUSG00000038022	Mindy4	20
ENSMUSG00000111540	4930584E12Rik	18		ENSMUSG00000101860	Mindy4b-ps	20
ENSMUSG00000117792	4930588A03Rik	18		ENSMUSG00000020827	Mink1	9
ENSMUSG00000034063	4930590J08Rik	20		ENSMUSG00000024896	Minpp1	20
ENSMUSG00000097725	4930593A02Rik	20		ENSMUSG00000042447	Mios	16
ENSMUSG00000103948	4930594C11Rik	20		ENSMUSG00000021993	Mipep	20
ENSMUSG00000075463	4930594M22Rik	20		ENSMUSG00000047022	Mipol1	20
ENSMUSG00000104900	4930596I21Rik	20		ENSMUSG00000093011	Mir100	20
ENSMUSG00000104964	4930597L12Rik	20		ENSMUSG00000074415	Mir100hg	9
ENSMUSG00000109160	4930598N05Rik	20		ENSMUSG00000065563	Mir103-2	20
ENSMUSG00000073144	4930599N23Rik	20		ENSMUSG00000080575	Mir1190	7
ENSMUSG00000031938	4931406C07Rik	13		ENSMUSG00000105379	Mir1191b	9
ENSMUSG00000066571	4931406P16Rik	18		ENSMUSG00000080626	Mir1192	20
ENSMUSG00000022179	4931414P19Rik	20		ENSMUSG00000080411	Mir1193	20

ENSMUSG00000054910	4931415C17Rik	20		ENSMUSG00000080687	Mir1197	20
ENSMUSG00000026774	4931423N10Rik	20		ENSMUSG00000100252	Mir124-2hg	18
ENSMUSG00000014837	4931428F04Rik	1		ENSMUSG00000097545	Mir124a-1hg	3
ENSMUSG00000078157	4931440F15Rik	20		ENSMUSG00000093122	Mir1264	20
ENSMUSG00000074580	4931440P22Rik	20		ENSMUSG00000065520	Mir128-1	20
ENSMUSG00000075070	4932412D23Rik	9		ENSMUSG00000065441	Mir128-2	20
ENSMUSG00000105881	4932422M17Rik	20		ENSMUSG00000065537	Mir132	20
ENSMUSG00000037270	4932438A13Rik	2		ENSMUSG00000065538	Mir153	20
ENSMUSG00000039851	4932438H23Rik	13		ENSMUSG00000065448	Mir154	20
ENSMUSG00000097216	4932441J04Rik	20		ENSMUSG00000089726	Mir17hg	20
ENSMUSG00000086796	4932702P03Rik	20		ENSMUSG00000065431	Mir186	20
ENSMUSG00000103321	4933403L11Rik	20		ENSMUSG00000084592	Mir1898	20
ENSMUSG00000097908	4933404O12Rik	20		ENSMUSG00000076379	Mir190a	1
ENSMUSG00000084234	4933405O20Rik	19		ENSMUSG00000093039	Mir1912	20
ENSMUSG00000110427	4933406B17Rik	20		ENSMUSG00000089214	Mir1931	20
ENSMUSG00000097494	4933406C10Rik	20		ENSMUSG00000088552	Mir1957a	20
ENSMUSG00000087475	4933406I18Rik	20		ENSMUSG00000088248	Mir1969	20
ENSMUSG00000069713	4933406P04Rik	20		ENSMUSG00000088148	Mir1983	20
ENSMUSG00000087396	4933407K13Rik	20		ENSMUSG00000080662	Mir1b	20
ENSMUSG00000026224	4933407L21Rik	20		ENSMUSG00000065507	Mir204	13
ENSMUSG00000049357	4933408B17Rik	19		ENSMUSG00000065485	Mir219a-2	20
ENSMUSG00000090369	4933411K16Rik	1		ENSMUSG00000085148	Mir22hg	20
ENSMUSG00000112627	4933412E12Rik	18		ENSMUSG00000065513	Mir26a-1	20
ENSMUSG00000113960	4933412O06Rik	20		ENSMUSG00000104794	Mir28c	20
ENSMUSG00000103006	4933417C20Rik	1		ENSMUSG00000065610	Mir29a	20
ENSMUSG00000085037	4933421O10Rik	20		ENSMUSG00000065604	Mir29b-1	20
ENSMUSG00000020807	4933427D14Rik	20		ENSMUSG00000098807	Mir300	20
ENSMUSG00000086697	4933427G23Rik	9		ENSMUSG00000093080	Mir3060	20
ENSMUSG00000097786	4933429H19Rik	20		ENSMUSG00000092751	Mir3080	20
ENSMUSG00000115008	4933429O19Rik	13		ENSMUSG00000092988	Mir3093	20
ENSMUSG00000107172	4933430H06Rik	9		ENSMUSG00000092815	Mir3099	9
ENSMUSG00000058046	4933430I17Rik	13		ENSMUSG00000065617	Mir323	20
ENSMUSG00000086968	4933431E20Rik	1		ENSMUSG00000065425	Mir325	20
ENSMUSG00000103715	4933431K14Rik	20		ENSMUSG00000065571	Mir326	20
ENSMUSG00000097021	4933433G15Rik	20		ENSMUSG00000065577	Mir329	20
ENSMUSG00000114362	4933433G19Rik	20		ENSMUSG00000065465	Mir33	1
ENSMUSG00000027942	4933434E20Rik	20		ENSMUSG00000065600	Mir338	20
ENSMUSG00000072893	4933439C10Rik	20		ENSMUSG00000065417	Mir340	20
ENSMUSG00000042087	4933440N22Rik	20		ENSMUSG00000065436	Mir342	10
ENSMUSG00000073147	5031425E22Rik	20		ENSMUSG00000065568	Mir344	12
ENSMUSG00000097885	5031434O11Rik	20		ENSMUSG00000093187	Mir344-2	9
ENSMUSG00000036046	5031439G07Rik	16		ENSMUSG00000092921	Mir344b	12
ENSMUSG00000102185	5033404E19Rik	20		ENSMUSG00000077107	Mir344c	20
ENSMUSG00000097251	5033417F24Rik	19		ENSMUSG00000093108	Mir344f	20
ENSMUSG00000112276	5033421B08Rik	20		ENSMUSG00000095154	Mir344i	20
ENSMUSG00000038152	5033430I15Rik	6		ENSMUSG00000065481	Mir346	20
ENSMUSG00000104030	5330406M23Rik	20		ENSMUSG00000098896	Mir3473g	20
ENSMUSG00000113927	5330409N07Rik	20		ENSMUSG00000076269	Mir374b	17
ENSMUSG00000086822	5330413P13Rik	8		ENSMUSG00000105178	Mir376a	20
ENSMUSG00000054944	5330416C01Rik	18		ENSMUSG00000076006	Mir376c	20
ENSMUSG00000040412	5330417C22Rik	9		ENSMUSG00000065498	Mir379	20
ENSMUSG00000114282	5330431K02Rik	2		ENSMUSG00000104582	Mir380	20
ENSMUSG00000087620	5330434G04Rik	19		ENSMUSG00000098838	Mir381	20
ENSMUSG00000106855	5330437M03Rik	9		ENSMUSG00000065457	Mir383	18

ENSMUSG00000052291	5330438D12Rik	20		ENSMUSG00000065499	Mir384	20
ENSMUSG00000089708	5330439A09Rik	20		ENSMUSG00000106199	Mir3967	20
ENSMUSG00000097827	5330439K02Rik	20		ENSMUSG00000093100	Mir3971	20
ENSMUSG00000097699	5430400D12Rik	20		ENSMUSG00000105849	Mir409	20
ENSMUSG00000085776	5430402O13Rik	10		ENSMUSG00000065477	Mir411	20
ENSMUSG00000072763	5430403G16Rik	20		ENSMUSG00000065509	Mir421	20
ENSMUSG00000085741	5430405H02Rik	20		ENSMUSG00000065450	Mir448	20
ENSMUSG00000114419	5430414B19Rik	20		ENSMUSG00000077925	Mir453	9
ENSMUSG00000097772	5430416N02Rik	20		ENSMUSG00000092990	Mir466q	20
ENSMUSG00000102808	5430420F09Rik	20		ENSMUSG00000096624	Mir467a-1	20
ENSMUSG00000025058	5430427O19Rik	20		ENSMUSG00000076219	Mir487b	20
ENSMUSG00000108780	5430434F05Rik	20		ENSMUSG00000070103	Mir488	20
ENSMUSG00000087174	5530601H04Rik	20		ENSMUSG00000070077	Mir491	20
ENSMUSG00000087424	5730405O15Rik	20		ENSMUSG00000070141	Mir494	20
ENSMUSG00000104343	5730408A14Rik	3		ENSMUSG00000070105	Mir495	9
ENSMUSG00000073755	5730409E04Rik	20		ENSMUSG00000070136	Mir496a	20
ENSMUSG00000116506	5730414N17Rik	18		ENSMUSG00000092981	Mir5125	20
ENSMUSG00000057181	5730455P16Rik	19		ENSMUSG00000076063	Mir539	20
ENSMUSG00000029089	5730480H06Rik	9		ENSMUSG00000076052	Mir541	20
ENSMUSG00000107468	5730507A11Rik	20		ENSMUSG00000076241	Mir543	20
ENSMUSG00000073197	5730507C01Rik	20		ENSMUSG00000077001	Mir544	20
ENSMUSG00000116145	5730521K06Rik	20		ENSMUSG00000093757	Mir5623	20
ENSMUSG00000032985	5730522E02Rik	18		ENSMUSG00000098973	Mir6236	18
ENSMUSG00000110148	5830408C22Rik	20		ENSMUSG00000098734	Mir6335	20
ENSMUSG00000106219	5830416I19Rik	20		ENSMUSG00000098660	Mir6336	3
ENSMUSG00000078684	5830417I10Rik	20		ENSMUSG00000099141	Mir6352	20
ENSMUSG00000084803	5830444B04Rik	20		ENSMUSG00000099045	Mir6374	20
ENSMUSG00000102744	5830444F18Rik	20		ENSMUSG00000077980	Mir654	20
ENSMUSG00000115007	5830448L01Rik	9		ENSMUSG00000076272	Mir666	20
ENSMUSG00000052658	5830454E08Rik	20		ENSMUSG00000076396	Mir667	1
ENSMUSG00000110972	5830462O15Rik	20		ENSMUSG00000075020	Mir670hg	20
ENSMUSG00000104830	5830487J09Rik	20		ENSMUSG00000076145	Mir679	20
ENSMUSG00000103477	5930409G06Rik	9		ENSMUSG00000076064	Mir690	20
ENSMUSG00000107201	5930420M18Rik	20		ENSMUSG00000099267	Mir6955	20
ENSMUSG00000106951	5930430L01Rik	12		ENSMUSG00000065434	Mir7-1	20
ENSMUSG00000105326	6030400A10Rik	20		ENSMUSG00000104914	Mir7061	20
ENSMUSG00000100301	6030407O03Rik	20		ENSMUSG00000076143	Mir708	20
ENSMUSG00000097760	6030442K20Rik	20		ENSMUSG00000076144	Mir709	20
ENSMUSG00000097207	6030443J06Rik	1		ENSMUSG00000098247	Mir7220	20
ENSMUSG00000022195	6030458C11Rik	20		ENSMUSG00000098299	Mir7224	9
ENSMUSG00000097577	6230400D17Rik	20		ENSMUSG00000076460	Mir744	20
ENSMUSG00000018451	6330403K07Rik	20		ENSMUSG00000076459	Mir758	20
ENSMUSG00000075585	6330403L08Rik	9		ENSMUSG00000076458	Mir764	20
ENSMUSG00000116733	6330408M09Rik	20		ENSMUSG00000098714	Mir7663	20
ENSMUSG00000009551	6330409D20Rik	20		ENSMUSG00000099320	Mir7664	20
ENSMUSG00000110067	6330411D24Rik	9		ENSMUSG00000098397	Mir7669	20
ENSMUSG00000108077	6330415B21Rik	3		ENSMUSG00000098286	Mir8091	20
ENSMUSG00000117322	6330415G19Rik	20		ENSMUSG00000078057	Mir873a	20
ENSMUSG00000085227	6330418K02Rik	20		ENSMUSG00000077851	Mir876	20
ENSMUSG00000107414	6330419E04Rik	20		ENSMUSG00000106493	Mir9-1	20
ENSMUSG00000045327	6330549D23Rik	20		ENSMUSG00000097023	Mir9-3hg	20
ENSMUSG00000093424	6330562C20Rik	20		ENSMUSG00000065530	Mir99a	20
ENSMUSG00000053545	6430503K07Rik	20		ENSMUSG00000090386	Mir99ahg	2
ENSMUSG00000102555	6430511E19Rik	20		ENSMUSG00000097391	Mirg	1

ENSMUSG00000031824	6430548M08Rik	18		ENSMUSG00000092770	Mirlet7a-2	20
ENSMUSG00000074646	6430550D23Rik	20		ENSMUSG00000065557	Mirlet7c-1	20
ENSMUSG00000037977	6430571L13Rik	4		ENSMUSG00000105758	Mirlet7e	20
ENSMUSG00000102545	6430573P05Rik	9		ENSMUSG00000065440	Mirlet7g	19
ENSMUSG00000108228	6430584L05Rik	16		ENSMUSG00000097636	Mirt1	20
ENSMUSG00000110127	6430710M23Rik	20		ENSMUSG00000040599	Mis12	18
ENSMUSG00000091947	6530401F13Rik	20		ENSMUSG00000022978	Mis18a	3
ENSMUSG00000079499	6530402F18Rik	12		ENSMUSG00000047534	Mis18bp1	20
ENSMUSG00000098097	6530403H02Rik	19		ENSMUSG00000035852	Misp	20
ENSMUSG00000043340	6530409C15Rik	20		ENSMUSG00000026088	Mitd1	1
ENSMUSG00000097375	6720427I07Rik	3		ENSMUSG00000035158	Mitf	13
ENSMUSG00000103432	6720464F23Rik	20		ENSMUSG00000031004	Mki67	18
ENSMUSG00000117655	6720468P15Rik	9		ENSMUSG00000027274	Mkks	20
ENSMUSG00000108507	6720469O03Rik	20		ENSMUSG00000025609	Mkln1	12
ENSMUSG00000107009	6720475M21Rik	20		ENSMUSG00000086212	Mkln1os	20
ENSMUSG00000106553	6720482G16Rik	20		ENSMUSG00000028708	Mknk1	20
ENSMUSG00000097934	6720483E21Rik	20		ENSMUSG00000020190	Mknk2	20
ENSMUSG00000072066	6720489N17Rik	1		ENSMUSG00000029922	Mkrn1	9
ENSMUSG00000103706	6820402A03Rik	2		ENSMUSG00000082389	Mkrn1-ps1	20
ENSMUSG00000032680	6820408C15Rik	20		ENSMUSG00000000439	Mkrn2	20
ENSMUSG00000071796	6820431F20Rik	20		ENSMUSG00000070527	Mkrn3	20
ENSMUSG00000116888	7120432I05Rik	3		ENSMUSG00000034121	Mks1	20
ENSMUSG00000098758	7630403G23Rik	1		ENSMUSG00000061013	Mkx	9
ENSMUSG00000103563	8030445P17Rik	20		ENSMUSG00000035805	Mlc1	20
ENSMUSG00000085058	8030453O22Rik	9		ENSMUSG00000048578	Mlec	18
ENSMUSG00000117768	8030456M14Rik	20		ENSMUSG00000048416	Mlf1	13
ENSMUSG00000105136	8030487O14Rik	20		ENSMUSG00000030120	Mlf2	2
ENSMUSG00000113432	8430406P12Rik	20		ENSMUSG00000032498	Mlh1	20
ENSMUSG00000085743	8430419K02Rik	20		ENSMUSG00000021245	Mlh3	6
ENSMUSG00000105095	8430422M14Rik	9		ENSMUSG00000032355	Mlip	1
ENSMUSG00000115970	8430426J06Rik	20		ENSMUSG00000012519	Mlkl	20
ENSMUSG00000034587	8430429K09Rik	20		ENSMUSG00000024212	Mllt1	9
ENSMUSG00000041878	8430432A02Rik	20		ENSMUSG00000026743	Mllt10	9
ENSMUSG00000116953	9030025P20Rik	20		ENSMUSG00000053192	Mllt11	1
ENSMUSG00000097343	9030407P20Rik	20		ENSMUSG00000028496	Mllt3	20
ENSMUSG00000045008	9030612E09Rik	3		ENSMUSG00000038437	Mllt6	9
ENSMUSG00000073158	9030624G23Rik	20		ENSMUSG00000026303	Mlph	20
ENSMUSG00000115837	9130002K18Rik	13		ENSMUSG00000024142	Mlst8	6
ENSMUSG00000030823	9130019O22Rik	20		ENSMUSG00000017801	Mlx	12
ENSMUSG00000073067	9130019P16Rik	20		ENSMUSG00000038342	Mlxip	20
ENSMUSG00000062944	9130023H24Rik	20		ENSMUSG00000005373	Mlxipl	20
ENSMUSG00000087022	9130024F11Rik	20		ENSMUSG00000074064	Mlycd	20
ENSMUSG00000097386	9130213A22Rik	20		ENSMUSG00000097191	Mm2pr	9
ENSMUSG00000090330	9130221H12Rik	20		ENSMUSG00000037022	Mmaa	20
ENSMUSG00000092626	9130230N09Rik	9		ENSMUSG00000029575	Mmab	20
ENSMUSG00000072982	9230009I02Rik	1		ENSMUSG00000028690	Mmachc	20
ENSMUSG00000070461	9230112E08Rik	20		ENSMUSG00000026766	Mmadhc	20
ENSMUSG00000111761	9230112J17Rik	1		ENSMUSG00000003948	Mmd	6
ENSMUSG00000097145	9230114K14Rik	20		ENSMUSG00000039533	Mmd2	20
ENSMUSG00000097403	9230116N13Rik	20		ENSMUSG00000027820	Mme	9
ENSMUSG00000091050	9330020H09Rik	20		ENSMUSG00000058183	Mmel1	18
ENSMUSG00000107879	9330102E08Rik	1		ENSMUSG00000061273	Mmgt1	20
ENSMUSG00000097842	9330104G04Rik	20		ENSMUSG00000048497	Mmgt2	20
ENSMUSG00000097098	9330111N05Rik	1		ENSMUSG00000000901	Mmp11	20

ENSMUSG00000118154	9330117O12Rik	20		ENSMUSG00000000957	Mmp14	20
ENSMUSG00000108242	9330118I20Rik	20		ENSMUSG00000031790	Mmp15	18
ENSMUSG00000103502	9330121J05Rik	20		ENSMUSG00000028226	Mmp16	9
ENSMUSG00000110249	9330121K16Rik	4		ENSMUSG00000029436	Mmp17	9
ENSMUSG00000097781	9330136K24Rik	20		ENSMUSG00000025355	Mmp19	20
ENSMUSG00000097061	9330151L19Rik	3		ENSMUSG00000031740	Mmp2	9
ENSMUSG00000056031	9330154J02Rik	9		ENSMUSG00000029061	Mmp23	20
ENSMUSG00000073154	9330158H04Rik	20		ENSMUSG00000027612	Mmp24	20
ENSMUSG00000004360	9330159F19Rik	20		ENSMUSG00000020682	Mmp28	20
ENSMUSG00000097177	9330159M07Rik	20		ENSMUSG00000017737	Mmp9	20
ENSMUSG00000113695	9330159N22Rik	20		ENSMUSG00000041445	Mmrn2	20
ENSMUSG00000072809	9330160F10Rik	1		ENSMUSG00000025159	Mms19	20
ENSMUSG00000055271	9330161L09Rik	20		ENSMUSG00000045751	Mms22l	20
ENSMUSG00000086448	9330162012Rik	20		ENSMUSG00000023921	Mmut	20
ENSMUSG00000109536	9330162G02Rik	3		ENSMUSG00000070576	Mn1	9
ENSMUSG00000039133	9330171B17Rik	9		ENSMUSG00000021103	Mnat1	20
ENSMUSG00000097166	9330179D12Rik	1		ENSMUSG00000033752	Mnd1	20
ENSMUSG00000056004	9330182L06Rik	20		ENSMUSG00000090272	Mndal	20
ENSMUSG00000097648	9330185C12Rik	20		ENSMUSG00000032221	Mns1	20
ENSMUSG00000115149	9330188P03Rik	18		ENSMUSG00000000282	Mnt	20
ENSMUSG00000104904	9330198I05Rik	20		ENSMUSG00000096458	Moap1	20
ENSMUSG00000114189	9330199G10Rik	20		ENSMUSG00000043131	Mob1a	20
ENSMUSG00000062704	9430002A10Rik	20		ENSMUSG00000006262	Mob1b	10
ENSMUSG00000059939	9430015G10Rik	20		ENSMUSG00000025147	Mob2	20
ENSMUSG00000107709	9430018G01Rik	20		ENSMUSG00000003348	Mob3a	18
ENSMUSG00000054457	9430021M05Rik	20		ENSMUSG00000073910	Mob3b	1
ENSMUSG00000055216	9430025C20Rik	20		ENSMUSG00000028709	Mob3c	20
ENSMUSG00000113341	9430031K09Rik	20		ENSMUSG00000025979	Mob4	20
ENSMUSG00000102964	9430034N14Rik	20		ENSMUSG00000032517	Mobp	16
ENSMUSG00000097073	9430037G07Rik	12		ENSMUSG00000039616	Mocos	20
ENSMUSG00000040139	9430038I01Rik	20		ENSMUSG00000064120	Mocs1	20
ENSMUSG00000086155	9430041J12Rik	20		ENSMUSG00000015536	Mocs2	20
ENSMUSG00000102344	9430053O09Rik	16		ENSMUSG00000074576	Mocs3	20
ENSMUSG00000100632	9430060I03Rik	20		ENSMUSG00000076439	Mog	16
ENSMUSG00000108402	9430064I24Rik	20		ENSMUSG00000030036	Mogs	20
ENSMUSG00000097360	9430065F17Rik	20		ENSMUSG00000056458	Mok	18
ENSMUSG00000111088	9430081H08Rik	20		ENSMUSG00000032583	Mon1a	20
ENSMUSG00000106164	9430085M18Rik	18		ENSMUSG00000078908	Mon1b	20
ENSMUSG00000103522	9430087J23Rik	20		ENSMUSG00000034602	Mon2	12
ENSMUSG00000084808	9430091E24Rik	20		ENSMUSG00000034543	Morc2a	20
ENSMUSG00000102205	9430092D12Rik	20		ENSMUSG00000048602	Morc2b	20
ENSMUSG00000075405	9430097D07Rik	20		ENSMUSG00000039456	Morc3	20
ENSMUSG00000109997	9430099M06Rik	20		ENSMUSG00000031434	Morc4	20
ENSMUSG00000102267	9530003O04Rik	20		ENSMUSG00000062270	Morf4l1	9
ENSMUSG00000112150	9530018F02Rik	20		ENSMUSG00000116875	Morf4l1-ps1	20
ENSMUSG00000102369	9530022L04Rik	20		ENSMUSG00000031422	Morf4l2	16
ENSMUSG00000097462	9530026P05Rik	20		ENSMUSG00000029049	Morn1	20
ENSMUSG00000097726	9530036O11Rik	20		ENSMUSG00000045257	Morn2	19
ENSMUSG00000085982	9530051G07Rik	8		ENSMUSG00000049670	Morn4	20
ENSMUSG00000097858	9530052C20Rik	20		ENSMUSG00000074813	Morrbid	20
ENSMUSG00000096938	9530052E02Rik	20		ENSMUSG00000046096	Mosmo	7
ENSMUSG00000115955	9530056E24Rik	20		ENSMUSG00000023074	Mospd1	20
ENSMUSG00000045345	9530056K15Rik	20		ENSMUSG00000061778	Mospd2	20
ENSMUSG00000097736	9530059O14Rik	6		ENSMUSG00000037221	Mospd3	20

ENSMUSG00000108030	9530062K07Rik	20		ENSMUSG00000046487	Mospd4	20
ENSMUSG00000036275	9530068E07Rik	1		ENSMUSG00000002227	Mov10	3
ENSMUSG00000036411	9530077C05Rik	20		ENSMUSG00000020000	Moxd1	20
ENSMUSG00000044125	9530080O11Rik	20		ENSMUSG00000023861	Mpc1	12
ENSMUSG00000097006	9530082P21Rik	20		ENSMUSG00000091498	Mpc1-ps	20
ENSMUSG00000107742	9530085L11Rik	20		ENSMUSG00000026568	Mpc2	12
ENSMUSG00000108076	9530086O07Rik	20		ENSMUSG00000018761	Mpdu1	2
ENSMUSG00000106371	9530097N15Rik	20		ENSMUSG00000028402	Mpdz	9
ENSMUSG00000097825	9630001P10Rik	18		ENSMUSG00000046805	Mpeg1	20
ENSMUSG00000115012	9630009A06Rik	20		ENSMUSG00000020287	Mpg	20
ENSMUSG00000103867	9630010A21Rik	20		ENSMUSG00000030521	Mphosph10	20
ENSMUSG00000115529	9630013A20Rik	1		ENSMUSG00000031843	Mphosph6	16
ENSMUSG00000118257	9630014M24Rik	20		ENSMUSG00000079184	Mphosph8	20
ENSMUSG00000115275	9630050E16Rik	20		ENSMUSG00000038126	Mphosph9	20
ENSMUSG00000087528	9830144P21Rik	20		ENSMUSG00000032306	Mpi	6
ENSMUSG00000117763	9830166K06Rik	20		ENSMUSG00000012429	Mplkip	9
ENSMUSG00000044551	9930012K11Rik	19		ENSMUSG00000003199	Mpnd	20
ENSMUSG00000097493	9930014A18Rik	20		ENSMUSG00000031402	Mpp1	19
ENSMUSG00000115276	9930017N22Rik	20		ENSMUSG00000017314	Mpp2	18
ENSMUSG00000046138	9930021J03Rik	7		ENSMUSG00000052373	Mpp3	6
ENSMUSG00000111131	9930024M15Rik	12		ENSMUSG00000079550	Mpp4	20
ENSMUSG00000044730	9930104L06Rik	20		ENSMUSG00000021112	Mpp5	13
ENSMUSG00000069893	9930111J21Rik1	19		ENSMUSG00000038388	Mpp6	20
ENSMUSG00000069892	9930111J21Rik2	20		ENSMUSG00000057440	Mpp7	19
ENSMUSG00000108120	9930120I10Rik	20		ENSMUSG00000062526	Mppe1	9
ENSMUSG00000110357	A030001D20Rik	20		ENSMUSG00000041708	Mpped1	20
ENSMUSG00000113880	A030005L19Rik	20		ENSMUSG00000016386	Mpped2	9
ENSMUSG00000069136	A130006I12Rik	20		ENSMUSG00000005417	Mprip	9
ENSMUSG00000079144	A130010J15Rik	20		ENSMUSG00000071711	Mpst	20
ENSMUSG00000112822	A130012E19Rik	20		ENSMUSG00000107283	Mpv17	20
ENSMUSG00000097944	A130014A01Rik	20		ENSMUSG00000022679	Mpv17l	9
ENSMUSG00000102642	A130048G24Rik	20		ENSMUSG00000026566	Mpzl1	20
ENSMUSG00000102277	A130050O07Rik	20		ENSMUSG00000070305	Mpzl3	20
ENSMUSG00000104291	A130071D04Rik	20		ENSMUSG00000026471	Mr1	20
ENSMUSG00000105891	A230001M10Rik	20		ENSMUSG00000042761	Mrap2	9
ENSMUSG00000087306	A230004M16Rik	20		ENSMUSG00000032470	Mras	18
ENSMUSG00000087612	A230005M16Rik	20		ENSMUSG00000026712	Mrc1	20
ENSMUSG00000068151	A230006K03Rik	1		ENSMUSG00000020695	Mrc2	20
ENSMUSG00000089633	A230009B12Rik	1		ENSMUSG00000031928	Mre11a	20
ENSMUSG00000117172	A230051N06Rik	20		ENSMUSG00000039395	Mreg	18
ENSMUSG00000087178	A230056P14Rik	20		ENSMUSG00000055302	Mrfap1	20
ENSMUSG00000109394	A230057D06Rik	20		ENSMUSG00000027569	Mrgbp	20
ENSMUSG00000087627	A230059L01Rik	20		ENSMUSG00000048965	Mrgpre	17
ENSMUSG00000097692	A230060F14Rik	20		ENSMUSG00000031070	Mrgprf	20
ENSMUSG00000086877	A230072C01Rik	20		ENSMUSG00000004996	Mri1	1
ENSMUSG00000084771	A230072E10Rik	20		ENSMUSG00000098404	Mrip-ps	20
ENSMUSG00000074473	A230072I06Rik	20		ENSMUSG00000018405	Mrm1	20
ENSMUSG00000100600	A230077H06Rik	20		ENSMUSG00000029557	Mrm2	12
ENSMUSG00000112367	A230081H15Rik	2		ENSMUSG00000038046	Mrm3	20
ENSMUSG00000055818	A230083G16Rik	20		ENSMUSG00000020381	Mrnip	20
ENSMUSG00000110047	A230085B16Rik	20		ENSMUSG00000064036	Mro	20
ENSMUSG00000097381	A230087F16Rik	8		ENSMUSG00000022558	Mroh1	9
ENSMUSG00000087026	A230103J11Rik	20		ENSMUSG00000087230	Mroh3	20
ENSMUSG00000109122	A230103L15Rik	3		ENSMUSG00000072487	Mroh5	20

ENSMUSG00000097292	A230107N01Rik	20		ENSMUSG00000047502	Mroh7	3
ENSMUSG00000030111	A2m	20		ENSMUSG00000074627	Mroh8	20
ENSMUSG00000047228	A2ml1	20		ENSMUSG00000029486	Mrpl1	20
ENSMUSG00000052479	A330008L17Rik	10		ENSMUSG00000001445	Mrpl10	20
ENSMUSG00000097915	A330009N23Rik	3		ENSMUSG00000024902	Mrpl11	12
ENSMUSG00000104093	A330015K06Rik	1		ENSMUSG00000039640	Mrpl12	20
ENSMUSG00000096929	A330023F24Rik	20		ENSMUSG00000022370	Mrpl13	12
ENSMUSG00000097591	A330032B11Rik	1		ENSMUSG00000023939	Mrpl14	12
ENSMUSG00000097622	A330033J07Rik	20		ENSMUSG00000033845	Mrpl15	10
ENSMUSG00000085615	A330035P11Rik	20		ENSMUSG00000024683	Mrpl16	20
ENSMUSG00000086213	A330040F15Rik	20		ENSMUSG00000030879	Mrpl17	20
ENSMUSG00000087466	A330041J22Rik	20		ENSMUSG00000057388	Mrpl18	10
ENSMUSG00000097326	A330048O09Rik	20		ENSMUSG00000030045	Mrpl19	20
ENSMUSG00000111994	A330049N07Rik	9		ENSMUSG00000002767	Mrpl2	12
ENSMUSG00000117100	A330072L02Rik	1		ENSMUSG00000029066	Mrpl20	19
ENSMUSG00000102995	A330074H02Rik	20		ENSMUSG00000024829	Mrpl21	3
ENSMUSG00000097960	A330074K22Rik	18		ENSMUSG00000020514	Mrpl22	20
ENSMUSG00000113186	A330076C08Rik	18		ENSMUSG00000037772	Mrpl23	12
ENSMUSG00000109321	A330076H08Rik	20		ENSMUSG00000075279	Mrpl23-ps1	12
ENSMUSG00000114210	A330084C13Rik	20		ENSMUSG00000019710	Mrpl24	20
ENSMUSG00000098284	A330093E20Rik	20		ENSMUSG00000024414	Mrpl27	20
ENSMUSG00000086363	A330102I10Rik	20		ENSMUSG00000024181	Mrpl28	12
ENSMUSG00000028794	A3galt2	20		ENSMUSG00000032563	Mrpl3	2
ENSMUSG00000047613	A430005L14Rik	20		ENSMUSG00000026087	Mrpl30	12
ENSMUSG00000097603	A430010J10Rik	6		ENSMUSG00000015672	Mrpl32	20
ENSMUSG00000085315	A430018G15Rik	12		ENSMUSG00000106918	Mrpl33	12
ENSMUSG00000117429	A430019L02Rik	20		ENSMUSG00000034880	Mrpl34	9
ENSMUSG00000104432	A430027C01Rik	20		ENSMUSG00000052962	Mrpl35	20
ENSMUSG00000104507	A430027H14Rik	20		ENSMUSG00000021607	Mrpl36	12
ENSMUSG00000056014	A430033K04Rik	20		ENSMUSG00000028622	Mrpl37	19
ENSMUSG00000087305	A430035B10Rik	20		ENSMUSG00000020775	Mrpl38	2
ENSMUSG00000090564	A430057M04Rik	20		ENSMUSG00000022889	Mrpl39	20
ENSMUSG00000105471	A430073D23Rik	20		ENSMUSG00000003299	Mrpl4	12
ENSMUSG00000112096	A430103D13Rik	20		ENSMUSG00000022706	Mrpl40	20
ENSMUSG00000097784	A430105J06Rik	20		ENSMUSG00000036850	Mrpl41	7
ENSMUSG00000098206	A430106G13Rik	20		ENSMUSG00000062981	Mrpl42	12
ENSMUSG00000086851	A430108G06Rik	20		ENSMUSG00000025208	Mrpl43	20
ENSMUSG00000102415	A430110C17Rik	20		ENSMUSG00000026248	Mrpl44	20
ENSMUSG00000054135	A430110L20Rik	20		ENSMUSG00000018882	Mrpl45	20
ENSMUSG00000047878	A4galt	20		ENSMUSG00000030612	Mrpl46	20
ENSMUSG00000006462	A530013C23Rik	19		ENSMUSG00000037531	Mrpl47	3
ENSMUSG00000072847	A530017D24Rik	19		ENSMUSG00000030706	Mrpl48	20
ENSMUSG00000104973	A530041M06Rik	2		ENSMUSG00000078480	Mrpl48-ps	20
ENSMUSG00000046764	A530053G22Rik	12		ENSMUSG00000007338	Mrpl49	20
ENSMUSG00000087694	A530058N18Rik	20		ENSMUSG00000044018	Mrpl50	20
ENSMUSG00000104273	A530064N14Rik	17		ENSMUSG00000030335	Mrpl51	20
ENSMUSG00000085112	A530072M11Rik	20		ENSMUSG00000010406	Mrpl52	3
ENSMUSG00000097322	A530083I20Rik	20		ENSMUSG00000034932	Mrpl54	12
ENSMUSG00000106024	A530083M17Rik	20		ENSMUSG00000036860	Mrpl55	3
ENSMUSG00000090863	A530084C06Rik	20		ENSMUSG00000021967	Mrpl57	20
ENSMUSG00000052760	A630001G21Rik	20		ENSMUSG00000018858	Mrpl58	7
ENSMUSG00000116809	A630036G19Rik	7		ENSMUSG00000028140	Mrpl9	20
ENSMUSG00000086725	A630052C17Rik	13		ENSMUSG00000030611	Mrps11	20
ENSMUSG00000101013	A630072M18Rik	20		ENSMUSG00000045948	Mrps12	12

ENSMUSG00000102721	A630081D01Rik	20		ENSMUSG00000058267	Mrps14	20
ENSMUSG00000116673	A630089N07Rik	1		ENSMUSG00000028861	Mrps15	20
ENSMUSG00000084846	A730011C13Rik	20		ENSMUSG00000049960	Mrps16	20
ENSMUSG00000085566	A730017L22Rik	9		ENSMUSG00000034211	Mrps17	9
ENSMUSG00000047828	A730018C14Rik	20		ENSMUSG00000023967	Mrps18a	3
ENSMUSG00000097924	A730020E08Rik	20		ENSMUSG00000024436	Mrps18b	20
ENSMUSG00000044522	A730020M07Rik	20		ENSMUSG00000016833	Mrps18c	12
ENSMUSG00000085139	A730046J19Rik	20		ENSMUSG00000035772	Mrps2	20
ENSMUSG00000097756	A730056A06Rik	9		ENSMUSG00000054312	Mrps21	12
ENSMUSG00000092375	A730060N03Rik	20		ENSMUSG00000032459	Mrps22	1
ENSMUSG00000053588	A730061H03Rik	20		ENSMUSG00000020477	Mrps24	19
ENSMUSG00000102971	A730062M13Rik	20		ENSMUSG00000014551	Mrps25	20
ENSMUSG00000112639	A730063M14Rik	20		ENSMUSG00000037740	Mrps26	12
ENSMUSG00000086693	A730081D07Rik	20		ENSMUSG00000041632	Mrps27	20
ENSMUSG00000093598	A730085K08Rik	20		ENSMUSG00000040269	Mrps28	3
ENSMUSG00000103529	A730089K16Rik	9		ENSMUSG00000021731	Mrps30	20
ENSMUSG00000113965	A730091E23Rik	20		ENSMUSG00000031533	Mrps31	19
ENSMUSG00000100812	A730098A19Rik	20		ENSMUSG00000029918	Mrps33	12
ENSMUSG00000053181	A830005F24Rik	9		ENSMUSG00000038880	Mrps34	20
ENSMUSG00000100627	A830008E24Rik	20		ENSMUSG00000040112	Mrps35	20
ENSMUSG00000089957	A830011K09Rik	18		ENSMUSG00000061474	Mrps36	12
ENSMUSG00000085996	A830012C17Rik	20		ENSMUSG00000021631	Mrps36-ps1	12
ENSMUSG00000057715	A830018L16Rik	15		ENSMUSG00000081911	Mrps36-ps2	20
ENSMUSG00000094707	A830019P07Rik	20		ENSMUSG00000027374	Mrps5	11
ENSMUSG00000117645	A830021F12Rik	20		ENSMUSG00000039680	Mrps6	20
ENSMUSG00000055010	A830031A19Rik	20		ENSMUSG00000046756	Mrps7	12
ENSMUSG00000111329	A830035O19Rik	20		ENSMUSG00000060679	Mrps9	12
ENSMUSG00000084890	A830036E02Rik	8		ENSMUSG00000026887	Mrrf	20
ENSMUSG00000097413	A830052D11Rik	1		ENSMUSG00000021339	Mrs2	19
ENSMUSG00000091890	A830073O21Rik	9		ENSMUSG00000042292	Mrtfa	9
ENSMUSG00000087143	A830082K12Rik	18		ENSMUSG00000009569	Mrtfb	6
ENSMUSG00000112280	A830082N09Rik	20		ENSMUSG00000028741	Mrto4	12
ENSMUSG00000074664	A830092H15Rik	9		ENSMUSG00000113749	Mrto4-ps1	20
ENSMUSG00000114961	A930002C04Rik	20		ENSMUSG00000005611	Mrvi1	18
ENSMUSG00000109242	A930002H02Rik	20		ENSMUSG00000024677	Ms4a6b	20
ENSMUSG00000075330	A930003A15Rik	20		ENSMUSG00000079419	Ms4a6c	20
ENSMUSG00000105076	A930003O13Rik	20		ENSMUSG00000024679	Ms4a6d	20
ENSMUSG00000054057	A930004D18Rik	20		ENSMUSG00000051246	Msantd1	9
ENSMUSG00000102602	A930004J17Rik	2		ENSMUSG00000042138	Msantd2	20
ENSMUSG00000106887	A930005G22Rik	20		ENSMUSG00000039693	Msantd3	3
ENSMUSG00000054426	A930005H10Rik	20		ENSMUSG00000041124	Msantd4	20
ENSMUSG00000103001	A930005N03Rik	16		ENSMUSG00000024151	Msh2	20
ENSMUSG00000084866	A930006K02Rik	20		ENSMUSG00000014850	Msh3	4
ENSMUSG00000097855	A930007I19Rik	20		ENSMUSG00000005493	Msh4	20
ENSMUSG00000092210	A930009A15Rik	20		ENSMUSG00000007035	Msh5	20
ENSMUSG00000097610	A930012L18Rik	20		ENSMUSG00000005370	Msh6	12
ENSMUSG00000054181	A930012O16Rik	20		ENSMUSG00000054256	Msi1	9
ENSMUSG00000092368	A930015D03Rik	20		ENSMUSG00000069769	Msi2	12
ENSMUSG00000114526	A930015P04Rik	20		ENSMUSG00000052915	Msl1	9
ENSMUSG00000040705	A930016O22Rik	20		ENSMUSG00000066415	Msl2	12
ENSMUSG00000025727	A930017K11Rik	20		ENSMUSG00000031358	Msl3	20
ENSMUSG00000060530	A930017M01Rik	9		ENSMUSG00000047669	Msl3l2	20
ENSMUSG00000113575	A930023M06Rik	1		ENSMUSG00000031604	Msmo1	1
ENSMUSG00000056735	A930024E05Rik	1		ENSMUSG00000031207	Msn	20

ENSMUSG00000113688	A930027P06Rik	10		ENSMUSG00000025044	Msr1	20
ENSMUSG00000114796	A930028N01Rik	18		ENSMUSG00000054733	Msra	5
ENSMUSG00000105226	A930028O11Rik	20		ENSMUSG00000075705	Msrb1	20
ENSMUSG00000096988	A930029G22Rik	18		ENSMUSG00000023094	Msrb2	1
ENSMUSG00000108985	A930030B08Rik	1		ENSMUSG00000051236	Msrb3	9
ENSMUSG00000090622	A930033H14Rik	20		ENSMUSG00000021815	Mss51	20
ENSMUSG00000052241	A930035D04Rik	20		ENSMUSG00000032584	Mst1r	20
ENSMUSG00000105469	A930036I15Rik	20		ENSMUSG00000068922	Msto1	7
ENSMUSG00000109408	A930037H05Rik	20		ENSMUSG00000048450	Msx1	13
ENSMUSG00000045339	A930104D05Rik	20		ENSMUSG00000064357	mt-Atp6	20
ENSMUSG00000074357	AA386476	20		ENSMUSG00000064356	mt-Atp8	20
ENSMUSG00000117896	AA387883	9		ENSMUSG00000064351	mt-Co1	20
ENSMUSG00000118084	AA388235	11		ENSMUSG00000064354	mt-Co2	20
ENSMUSG00000084381	AA413626	10		ENSMUSG00000064358	mt-Co3	19
ENSMUSG00000083307	AA414768	9		ENSMUSG00000064370	mt-Cytb	16
ENSMUSG00000093483	AA465934	20		ENSMUSG00000064341	mt-Nd1	20
ENSMUSG00000073867	AA474408	20		ENSMUSG00000064345	mt-Nd2	20
ENSMUSG00000103149	AA914427	20		ENSMUSG00000064360	mt-Nd3	12
ENSMUSG00000042510	AA986860	20		ENSMUSG00000064363	mt-Nd4	20
ENSMUSG00000036678	Aaas	20		ENSMUSG00000065947	mt-Nd4l	20
ENSMUSG00000029482	Aacs	20		ENSMUSG00000064367	mt-Nd5	16
ENSMUSG00000037257	Aagab	20		ENSMUSG00000064368	mt-Nd6	20
ENSMUSG00000057230	Aak1	12		ENSMUSG00000064337	mt-Rnr1	20
ENSMUSG00000035642	Aamdc	12		ENSMUSG00000064339	mt-Rnr2	20
ENSMUSG00000006299	Aamp	10		ENSMUSG00000064347	mt-Ta	10
ENSMUSG00000020804	Aanat	20		ENSMUSG00000064349	mt-Tc	20
ENSMUSG00000027628	Aar2	1		ENSMUSG00000064369	mt-Te	20
ENSMUSG00000068522	Aard	9		ENSMUSG00000064336	mt-Tf	10
ENSMUSG00000031960	Aars	9		ENSMUSG00000064359	mt-Tg	10
ENSMUSG00000023938	Aars2	20		ENSMUSG00000064364	mt-Th	10
ENSMUSG00000075528	Aarsd1	12		ENSMUSG00000064342	mt-Ti	10
ENSMUSG00000055923	Aasdh	9		ENSMUSG00000064355	mt-Tk	10
ENSMUSG00000025894	Aasdhppt	13		ENSMUSG00000064340	mt-Tl1	10
ENSMUSG00000029695	Aass	20		ENSMUSG00000064366	mt-Tl2	10
ENSMUSG00000018697	Aatf	1		ENSMUSG00000064344	mt-Tm	10
ENSMUSG00000025375	Aatk	18		ENSMUSG00000064372	mt-Tp	10
ENSMUSG00000046109	AB041806	20		ENSMUSG00000064343	mt-Tq	10
ENSMUSG00000057880	Abat	20		ENSMUSG00000064361	mt-Tr	10
ENSMUSG00000015243	Abca1	15		ENSMUSG00000064352	mt-Ts1	1
ENSMUSG00000035435	Abca17	20		ENSMUSG00000064365	mt-Ts2	10
ENSMUSG00000026944	Abca2	15		ENSMUSG00000064371	mt-Tt	10
ENSMUSG00000024130	Abca3	18		ENSMUSG00000064338	mt-Tv	10
ENSMUSG00000028125	Abca4	13		ENSMUSG00000064346	mt-Tw	20
ENSMUSG00000018800	Abca5	19		ENSMUSG00000064350	mt-Ty	9
ENSMUSG00000044749	Abca6	20		ENSMUSG00000031765	Mt1	9
ENSMUSG00000035722	Abca7	9		ENSMUSG00000031762	Mt2	3
ENSMUSG00000041828	Abca8a	20		ENSMUSG00000031760	Mt3	10
ENSMUSG00000020620	Abca8b	1		ENSMUSG00000021144	Mta1	20
ENSMUSG00000041797	Abca9	20		ENSMUSG00000071646	Mta2	20
ENSMUSG00000031974	Abcb10	20		ENSMUSG00000055817	Mta3	9
ENSMUSG00000040584	Abcb1a	20		ENSMUSG00000091510	Mtag2	5
ENSMUSG00000028970	Abcb1b	20		ENSMUSG00000062937	Mtap	18
ENSMUSG00000042476	Abcb4	1		ENSMUSG00000022369	Mtbp	2
ENSMUSG00000026198	Abcb6	20		ENSMUSG00000024012	Mtch1	20

ENSMUSG00000031333	Abcb7	20		ENSMUSG00000027282	Mtch2	20
ENSMUSG00000028973	Abcb8	20		ENSMUSG00000052105	Mtcl1	9
ENSMUSG00000029408	Abcb9	9		ENSMUSG00000031200	Mtcp1	20
ENSMUSG00000023088	Abcc1	9		ENSMUSG00000022255	Mtdh	20
ENSMUSG00000032842	Abcc10	20		ENSMUSG00000040429	Mterf1a	1
ENSMUSG00000036872	Abcc12	9		ENSMUSG00000053178	Mterf1b	1
ENSMUSG00000020865	Abcc3	20		ENSMUSG00000049038	Mterf2	20
ENSMUSG00000032849	Abcc4	20		ENSMUSG00000021519	Mterf3	20
ENSMUSG00000022822	Abcc5	9		ENSMUSG00000026273	Mterf4	20
ENSMUSG00000030834	Abcc6	20		ENSMUSG00000028890	Mtf1	20
ENSMUSG00000040136	Abcc8	20		ENSMUSG00000029267	Mtf2	18
ENSMUSG00000030249	Abcc9	20		ENSMUSG00000059183	Mtfmt	1
ENSMUSG00000031378	Abcd1	20		ENSMUSG00000004748	Mtfp1	20
ENSMUSG00000055782	Abcd2	20		ENSMUSG00000027601	Mtfr1	20
ENSMUSG00000028127	Abcd3	20		ENSMUSG00000046671	Mtfr1l	9
ENSMUSG00000021240	Abcd4	20		ENSMUSG00000019992	Mtfr2	20
ENSMUSG00000058355	Abce1	20		ENSMUSG00000039018	Mtg1	20
ENSMUSG00000038762	Abcf1	1		ENSMUSG00000039069	Mtg2	20
ENSMUSG00000028953	Abcf2	20		ENSMUSG00000021048	Mthfd1	19
ENSMUSG00000003234	Abcf3	20		ENSMUSG00000040675	Mthfd1l	20
ENSMUSG00000024030	Abcg1	19		ENSMUSG00000005667	Mthfd2	20
ENSMUSG00000029802	Abcg2	20		ENSMUSG00000029376	Mthfd2l	20
ENSMUSG00000029299	Abcg3	20		ENSMUSG00000029009	Mthfr	20
ENSMUSG00000032131	Abcg4	9		ENSMUSG00000066442	Mthfs	20
ENSMUSG00000006638	Abhd1	1		ENSMUSG00000031816	Mthfsd	1
ENSMUSG00000033157	Abhd10	20		ENSMUSG00000079427	Mthfsl	12
ENSMUSG00000040532	Abhd11	20		ENSMUSG00000020459	Mtif2	20
ENSMUSG00000085042	Abhd11os	1		ENSMUSG00000016510	Mtif3	20
ENSMUSG00000032046	Abhd12	20		ENSMUSG00000051319	Mtln	12
ENSMUSG00000090121	Abhd12b	18		ENSMUSG00000031337	Mtm1	20
ENSMUSG00000040396	Abhd13	3		ENSMUSG00000015214	Mtmr1	20
ENSMUSG00000042210	Abhd14a	9		ENSMUSG00000030522	Mtmr10	3
ENSMUSG00000042073	Abhd14b	20		ENSMUSG00000045934	Mtmr11	18
ENSMUSG00000000686	Abhd15	20		ENSMUSG00000039458	Mtmr12	5
ENSMUSG00000007036	Abhd16a	20		ENSMUSG00000030269	Mtmr14	20
ENSMUSG00000003346	Abhd17a	3		ENSMUSG00000031918	Mtmr2	4
ENSMUSG00000047368	Abhd17b	9		ENSMUSG00000034354	Mtmr3	3
ENSMUSG00000038459	Abhd17c	6		ENSMUSG00000018401	Mtmr4	20
ENSMUSG00000037818	Abhd18	20		ENSMUSG00000021987	Mtmr6	20
ENSMUSG00000039202	Abhd2	9		ENSMUSG00000039431	Mtmr7	14
ENSMUSG00000002475	Abhd3	20		ENSMUSG00000035078	Mtmr9	2
ENSMUSG00000040997	Abhd4	2		ENSMUSG00000032342	Mto1	10
ENSMUSG00000032540	Abhd5	20		ENSMUSG00000028991	Mtor	2
ENSMUSG00000025277	Abhd6	20		ENSMUSG00000024234	Mtpap	20
ENSMUSG00000007950	Abhd8	20		ENSMUSG00000029840	Mtpn	9
ENSMUSG00000058835	Abi1	9		ENSMUSG00000021311	Mtr	20
ENSMUSG00000026782	Abi2	9		ENSMUSG00000016018	Mtrex	20
ENSMUSG00000018381	Abi3	20		ENSMUSG00000022022	Mtrf1	20
ENSMUSG00000035258	Abi3bp	9		ENSMUSG00000019774	Mtrf1l	1
ENSMUSG00000038827	Abitram	20		ENSMUSG00000034617	Mtrr	20
ENSMUSG00000026842	Abl1	20		ENSMUSG00000022353	Mtss1	9
ENSMUSG00000026596	Abl2	9		ENSMUSG00000033763	Mtss2	6
ENSMUSG00000025085	Ablim1	19		ENSMUSG00000028158	Mttp	1
ENSMUSG00000029095	Ablim2	19		ENSMUSG00000038065	Mturn	19

ENSMUSG00000032735	Ablim3	19		ENSMUSG00000045636	Mtus1	16
ENSMUSG00000017631	Abr	5		ENSMUSG00000029651	Mtus2	20
ENSMUSG00000078453	Abracl	18		ENSMUSG00000064068	Mtx1	19
ENSMUSG00000035234	Abraxas1	20		ENSMUSG00000027099	Mtx2	20
ENSMUSG00000030965	Abraxas2	20		ENSMUSG00000021704	Mtx3	20
ENSMUSG00000036376	Abt1	20		ENSMUSG00000042784	Muc1	20
ENSMUSG00000030083	Abtb1	20		ENSMUSG00000094840	Muc3a	9
ENSMUSG00000032724	Abtb2	20		ENSMUSG00000041241	Mul1	20
ENSMUSG00000118501	AC020971.1	20		ENSMUSG00000024906	Mus81	1
ENSMUSG00000118577	AC099699.1	20		ENSMUSG00000057280	Musk	9
ENSMUSG00000118534	AC102368.1	3		ENSMUSG00000042485	Mustn1	20
ENSMUSG00000118561	AC102368.2	20		ENSMUSG00000028687	Mutyh	18
ENSMUSG00000117102	AC107792.1	20		ENSMUSG00000031813	Mvb12a	20
ENSMUSG00000117901	AC109619.1	20		ENSMUSG00000038740	Mvb12b	9
ENSMUSG00000111374	AC113305.1	20		ENSMUSG00000006517	Mvd	20
ENSMUSG00000118426	AC115893.1	1		ENSMUSG00000041939	Mvk	20
ENSMUSG00000118492	AC121143.1	20		ENSMUSG00000030681	Mvp	20
ENSMUSG00000118419	AC124413.1	9		ENSMUSG00000000386	Mx1	20
ENSMUSG00000118364	AC124502.1	20		ENSMUSG00000023341	Mx2	20
ENSMUSG00000118029	AC132288.1	20		ENSMUSG00000001156	Mxd1	9
ENSMUSG00000117775	AC132307.1	20		ENSMUSG00000021485	Mxd3	20
ENSMUSG00000096808	AC132444.6	20		ENSMUSG00000037235	Mxd4	20
ENSMUSG00000118550	AC135859.1	20		ENSMUSG00000025025	Mxi1	3
ENSMUSG00000118456	AC137513.1	4		ENSMUSG00000020814	Mxra7	5
ENSMUSG00000117238	AC139131.1	20		ENSMUSG00000029070	Mxra8	14
ENSMUSG00000118410	AC139347.1	20		ENSMUSG00000025141	Myadml2	20
ENSMUSG00000118094	AC142100.1	20		ENSMUSG00000084822	Myadml2os	20
ENSMUSG00000118487	AC145556.1	20		ENSMUSG00000019982	Myb	20
ENSMUSG00000095041	AC149090.1	2		ENSMUSG00000040463	Mybbp1a	9
ENSMUSG00000118555	AC150312.1	20		ENSMUSG00000025912	Mybl1	19
ENSMUSG00000118423	AC154328.1	20		ENSMUSG00000017861	Mybl2	20
ENSMUSG00000117546	AC154542.1	20		ENSMUSG00000020061	Mybpc1	20
ENSMUSG00000118559	AC154607.1	20		ENSMUSG00000038670	Mybpc2	20
ENSMUSG00000118018	AC156546.1	20		ENSMUSG00000042451	Mybph	20
ENSMUSG00000118477	AC159140.2	20		ENSMUSG00000022346	Myc	20
ENSMUSG00000118499	AC160138.1	1		ENSMUSG00000028647	Mycbp	12
ENSMUSG00000117733	AC163347.1	20		ENSMUSG00000033004	Mycbp2	3
ENSMUSG00000118504	AC163616.1	12		ENSMUSG00000039110	Mycbpap	13
ENSMUSG00000118071	AC166172.1	20		ENSMUSG00000028654	Mycl	5
ENSMUSG00000115852	AC169509.1	20		ENSMUSG00000037169	Mycn	20
ENSMUSG00000118449	AC241534.1	20		ENSMUSG00000032508	Myd88	1
ENSMUSG00000036138	Acaa1a	12		ENSMUSG00000019579	Mydgf	20
ENSMUSG00000010651	Acaa1b	20		ENSMUSG00000027201	Myef2	16
ENSMUSG00000036880	Acaa2	12		ENSMUSG00000001285	Myg1	10
ENSMUSG00000020532	Acaca	9		ENSMUSG00000020900	Myh10	9
ENSMUSG00000042010	Acacb	13		ENSMUSG00000018830	Myh11	20
ENSMUSG00000029456	Acad10	20		ENSMUSG00000030739	Myh14	16
ENSMUSG00000090150	Acad11	15		ENSMUSG00000092009	Myh15	20
ENSMUSG00000042647	Acad12	20		ENSMUSG00000020908	Myh3	20
ENSMUSG00000031969	Acad8	20		ENSMUSG00000053093	Myh7	19
ENSMUSG00000027710	Acad9	20		ENSMUSG00000074652	Myh7b	1
ENSMUSG00000026003	Acadl	20		ENSMUSG00000022443	Myh9	20
ENSMUSG00000062908	Acadm	20		ENSMUSG00000024048	Myl12a	20
ENSMUSG00000029545	Acads	5		ENSMUSG00000034868	Myl12b	12

ENSMUSG00000030861	Acadsb	20		ENSMUSG00000013936	Myl2	20
ENSMUSG00000018574	Acadvl	7		ENSMUSG00000061086	Myl4	12
ENSMUSG00000030607	Acan	20		ENSMUSG00000090841	Myl6	12
ENSMUSG00000049076	Acap2	9		ENSMUSG00000039824	Myl6b	9
ENSMUSG00000029033	Acap3	9		ENSMUSG00000067818	Myl9	1
ENSMUSG00000032047	Acat1	20		ENSMUSG00000038175	Mylip	20
ENSMUSG00000023832	Acat2	1		ENSMUSG00000022836	Mylk	12
ENSMUSG00000062480	Acat3	20		ENSMUSG00000030672	Mylpf	20
ENSMUSG00000026499	Acbd3	9		ENSMUSG00000037730	Mynn	20
ENSMUSG00000056938	Acbd4	20		ENSMUSG00000022272	Myo10	20
ENSMUSG00000026781	Acbd5	20		ENSMUSG00000034427	Myo15b	7
ENSMUSG00000033701	Acbd6	20		ENSMUSG00000039057	Myo16	20
ENSMUSG00000040272	Accs	20		ENSMUSG00000000631	Myo18a	1
ENSMUSG00000038000	Acd	20		ENSMUSG00000072720	Myo18b	20
ENSMUSG00000020681	Ace	13		ENSMUSG00000020527	Myo19	20
ENSMUSG00000015405	Ace2	17		ENSMUSG00000025401	Myo1a	1
ENSMUSG00000038007	Acer2	20		ENSMUSG00000018417	Myo1b	9
ENSMUSG00000030760	Acer3	20		ENSMUSG00000017774	Myo1c	9
ENSMUSG00000023328	Ache	9		ENSMUSG00000035441	Myo1d	16
ENSMUSG00000022185	Acin1	9		ENSMUSG00000032220	Myo1e	20
ENSMUSG00000037872	Ackr1	1		ENSMUSG00000024300	Myo1f	20
ENSMUSG00000044534	Ackr2	9		ENSMUSG00000020437	Myo1g	9
ENSMUSG00000044337	Ackr3	20		ENSMUSG00000066952	Myo1h	20
ENSMUSG00000020917	Acly	9		ENSMUSG00000042064	Myo3b	1
ENSMUSG00000028405	Aco1	20		ENSMUSG00000034593	Myo5a	18
ENSMUSG00000022477	Aco2	20		ENSMUSG00000025885	Myo5b	18
ENSMUSG00000072949	Acot1	1		ENSMUSG00000033590	Myo5c	1
ENSMUSG00000034853	Acot11	18		ENSMUSG00000033577	Myo6	20
ENSMUSG00000021620	Acot12	20		ENSMUSG00000030761	Myo7a	20
ENSMUSG00000006717	Acot13	10		ENSMUSG00000024388	Myo7b	9
ENSMUSG00000021226	Acot2	20		ENSMUSG00000039585	Myo9a	7
ENSMUSG00000021228	Acot3	20		ENSMUSG00000004677	Myo9b	20
ENSMUSG00000052392	Acot4	20		ENSMUSG00000026697	Myoc	2
ENSMUSG00000043487	Acot6	20		ENSMUSG00000048612	Myof	20
ENSMUSG00000028937	Acot7	9		ENSMUSG00000024049	Myom1	20
ENSMUSG00000017307	Acot8	8		ENSMUSG00000037139	Myom3	20
ENSMUSG00000025287	Acot9	9		ENSMUSG00000046312	Myorg	20
ENSMUSG00000020777	Acox1	1		ENSMUSG00000024471	Myot	20
ENSMUSG00000029098	Acox3	9		ENSMUSG00000020067	Mypn	20
ENSMUSG00000044573	Acp1	20		ENSMUSG00000048481	Mypop	20
ENSMUSG00000002103	Acp2	12		ENSMUSG00000086533	Mypopos	20
ENSMUSG00000028093	Acp6	1		ENSMUSG00000036098	Myrf	16
ENSMUSG00000037469	Acp7	20		ENSMUSG00000041794	Myrip	19
ENSMUSG00000022622	Acr	20		ENSMUSG00000062627	Mysm1	20
ENSMUSG00000072770	Acrbp	20		ENSMUSG00000010505	Myt1	19
ENSMUSG00000032281	Acsbg1	18		ENSMUSG00000061911	Myt1l	9
ENSMUSG00000076435	Acsf2	20		ENSMUSG00000041361	Myzap	20
ENSMUSG00000015016	Acsf3	20		ENSMUSG00000030380	Mzf1	9
ENSMUSG00000018796	Acsl1	20		ENSMUSG00000033186	Mzt1	20
ENSMUSG00000032883	Acsl3	18		ENSMUSG00000022671	Mzt2	12
ENSMUSG00000031278	Acsl4	19		ENSMUSG00000094265	n-R5s109	10
ENSMUSG00000024981	Acsl5	9		ENSMUSG00000095915	n-R5s115	10
ENSMUSG00000020333	Acsl6	18		ENSMUSG00000096037	n-R5s136	10
ENSMUSG00000030972	Acsm5	1		ENSMUSG00000065893	n-R5s151	10

ENSMUSG00000027452	Acss1	20		ENSMUSG00000084480	n-R5s155	20
ENSMUSG00000027605	Acss2	18		ENSMUSG00000065311	n-R5s156	10
ENSMUSG00000035948	Acss3	20		ENSMUSG00000065662	n-R5s161	20
ENSMUSG00000031972	Acta1	20		ENSMUSG00000064798	n-R5s193	10
ENSMUSG00000035783	Acta2	20		ENSMUSG00000094830	n-R5s194	10
ENSMUSG00000029580	Actb	6		ENSMUSG00000075918	n-R5s2	10
ENSMUSG00000074978	Actg-ps1	12		ENSMUSG00000077382	n-R5s200	18
ENSMUSG00000062825	Actg1	20		ENSMUSG00000064857	n-R5s25	20
ENSMUSG00000027671	Actl6a	20		ENSMUSG00000077789	n-R5s26	20
ENSMUSG00000029712	Actl6b	3		ENSMUSG00000064748	n-R5s28	10
ENSMUSG00000015143	Actn1	9		ENSMUSG00000070225	n-R5s33	10
ENSMUSG00000052374	Actn2	9		ENSMUSG00000064409	n-R5s40	20
ENSMUSG00000054808	Actn4	3		ENSMUSG00000084541	n-R5s45	20
ENSMUSG00000021076	Actr10	9		ENSMUSG00000084431	n-R5s48	17
ENSMUSG00000025228	Actr1a	20		ENSMUSG00000064455	n-R5s56	10
ENSMUSG00000037351	Actr1b	20		ENSMUSG00000064714	n-R5s62	10
ENSMUSG00000020152	Actr2	10		ENSMUSG00000077309	n-R5s8	20
ENSMUSG00000026341	Actr3	18		ENSMUSG00000084545	n-R5s80	20
ENSMUSG00000056367	Actr3b	9		ENSMUSG00000064618	n-R5s95	20
ENSMUSG00000037761	Actr5	20		ENSMUSG00000084498	n-R5s98	20
ENSMUSG00000019948	Actr6	3		ENSMUSG00000031652	N4bp1	20
ENSMUSG00000015971	Actr8	20		ENSMUSG00000037795	N4bp2	18
ENSMUSG00000037737	Actrt3	20		ENSMUSG00000041132	N4bp2l1	20
ENSMUSG00000026836	Acvr1	9		ENSMUSG00000029655	N4bp2l2	7
ENSMUSG00000000532	Acvr1b	9		ENSMUSG00000106978	N4bp2os	1
ENSMUSG00000026834	Acvr1c	9		ENSMUSG00000001053	N4bp3	1
ENSMUSG00000052155	Acvr2a	12		ENSMUSG00000044442	N6amt1	20
ENSMUSG00000061393	Acvr2b	9		ENSMUSG00000031388	Naa10	20
ENSMUSG00000000530	Acvrl1	9		ENSMUSG00000063273	Naa15	2
ENSMUSG00000023262	Acy1	3		ENSMUSG00000022020	Naa16	20
ENSMUSG00000024866	Acy3	9		ENSMUSG00000002728	Naa20	20
ENSMUSG00000008822	Acyp1	20		ENSMUSG00000042719	Naa25	20
ENSMUSG00000060923	Acyp2	20		ENSMUSG00000036282	Naa30	3
ENSMUSG00000017697	Ada	20		ENSMUSG00000021555	Naa35	5
ENSMUSG00000027259	Adal	20		ENSMUSG00000059278	Naa38	12
ENSMUSG00000054693	Adam10	20		ENSMUSG00000024764	Naa40	9
ENSMUSG00000020926	Adam11	19		ENSMUSG00000022698	Naa50	20
ENSMUSG00000054555	Adam12	20		ENSMUSG00000005982	Naa60	20
ENSMUSG00000028041	Adam15	20		ENSMUSG00000079334	Naa80	20
ENSMUSG00000052593	Adam17	20		ENSMUSG00000029413	Naaa	9
ENSMUSG00000031552	Adam18	20		ENSMUSG00000043943	Naalad2	20
ENSMUSG00000011256	Adam19	20		ENSMUSG00000102758	Naaladl2	20
ENSMUSG00000072647	Adam1a	20		ENSMUSG00000002881	Nab1	19
ENSMUSG00000008438	Adam21	20		ENSMUSG00000025402	Nab2	20
ENSMUSG00000040537	Adam22	7		ENSMUSG00000026107	Nabp1	20
ENSMUSG00000025964	Adam23	20		ENSMUSG00000025374	Nabp2	20
ENSMUSG00000037437	Adam32	20		ENSMUSG00000061315	Naca	3
ENSMUSG00000072972	Adam4	1		ENSMUSG00000041073	Nacad	18
ENSMUSG00000025473	Adam8	20		ENSMUSG00000001910	Nacc1	12
ENSMUSG00000031555	Adam9	20		ENSMUSG00000026932	Nacc2	18
ENSMUSG00000022893	Adamts1	20		ENSMUSG00000029063	Nadk	20
ENSMUSG00000024299	Adamts10	9		ENSMUSG00000031090	Nadsyn1	20
ENSMUSG00000047497	Adamts12	20		ENSMUSG00000031878	Nae1	20
ENSMUSG00000033453	Adamts15	18		ENSMUSG00000014907	Naf1	20

ENSMUSG00000049538	Adamts16	18		ENSMUSG00000022453	Naga	20
ENSMUSG00000058145	Adamts17	9		ENSMUSG00000034744	Nagk	1
ENSMUSG00000053399	Adamts18	20		ENSMUSG00000001751	Naglu	20
ENSMUSG00000053441	Adamts19	18		ENSMUSG00000023143	Nagpa	20
ENSMUSG00000036545	Adamts2	18		ENSMUSG00000039164	Naif1	20
ENSMUSG00000022449	Adamts20	20		ENSMUSG00000078945	Naip2	20
ENSMUSG00000043635	Adamts3	9		ENSMUSG00000071203	Naip5	20
ENSMUSG00000006403	Adamts4	16		ENSMUSG00000078942	Naip6	20
ENSMUSG00000022894	Adamts5	1		ENSMUSG00000000197	Nalcn	3
ENSMUSG00000046169	Adamts6	1		ENSMUSG00000020572	Nampt	20
ENSMUSG00000032363	Adamts7	20		ENSMUSG00000072437	Nanos1	9
ENSMUSG00000031994	Adamts8	19		ENSMUSG00000051965	Nanos2	20
ENSMUSG00000030022	Adamts9	20		ENSMUSG00000056155	Nanos3	20
ENSMUSG00000066113	Adamtsl1	9		ENSMUSG00000053916	Nanp	20
ENSMUSG00000036040	Adamtsl2	20		ENSMUSG00000028334	Nans	6
ENSMUSG00000070469	Adamtsl3	1		ENSMUSG00000058799	Nap1l1	18
ENSMUSG00000015850	Adamtsl4	20		ENSMUSG00000082229	Nap1l2	9
ENSMUSG00000043822	Adamtsl5	9		ENSMUSG00000055733	Nap1l3	9
ENSMUSG00000056413	Adap1	18		ENSMUSG00000059119	Nap1l4	7
ENSMUSG00000020709	Adap2	20		ENSMUSG00000055430	Nap1l5	17
ENSMUSG00000027951	Adar	20		ENSMUSG00000006024	Napa	20
ENSMUSG00000020262	Adarb1	18		ENSMUSG00000027438	Napb	19
ENSMUSG00000052551	Adarb2	18		ENSMUSG00000044968	Napepld	19
ENSMUSG00000031949	Adat1	20		ENSMUSG00000024581	Napg	9
ENSMUSG00000019808	Adat2	12		ENSMUSG00000022574	Naprt	20
ENSMUSG00000021044	Adck1	20		ENSMUSG00000000056	Narf	20
ENSMUSG00000046947	Adck2	20		ENSMUSG00000024587	Nars	18
ENSMUSG00000022550	Adck5	20		ENSMUSG00000018995	Nars2	9
ENSMUSG00000020431	Adcy1	19		ENSMUSG00000028693	Nasp	20
ENSMUSG00000026567	Adcy10	20		ENSMUSG00000025588	Nat1	20
ENSMUSG00000021536	Adcy2	5		ENSMUSG00000027185	Nat10	10
ENSMUSG00000020654	Adcy3	9		ENSMUSG00000035285	Nat14	9
ENSMUSG00000022220	Adcy4	20		ENSMUSG00000051147	Nat2	20
ENSMUSG00000022840	Adcy5	9		ENSMUSG00000030004	Nat8	20
ENSMUSG00000022994	Adcy6	6		ENSMUSG00000057103	Nat8f1	20
ENSMUSG00000031659	Adcy7	18		ENSMUSG00000051262	Nat8f3	20
ENSMUSG00000022376	Adcy8	18		ENSMUSG00000068299	Nat8f4	20
ENSMUSG00000005580	Adcy9	9		ENSMUSG00000079494	Nat8f5	20
ENSMUSG00000024256	Adcyap1	9		ENSMUSG00000048142	Nat8l	18
ENSMUSG00000029778	Adcyap1r1	9		ENSMUSG00000015542	Nat9	1
ENSMUSG00000029106	Add1	9		ENSMUSG00000018931	Natd1	20
ENSMUSG00000030000	Add2	9		ENSMUSG00000009418	Nav1	9
ENSMUSG00000025026	Add3	9		ENSMUSG00000052512	Nav2	19
ENSMUSG00000050994	Adgb	20		ENSMUSG00000020181	Nav3	9
ENSMUSG00000025475	Adgra1	18		ENSMUSG00000031505	Naxd	19
ENSMUSG00000031486	Adgra2	20		ENSMUSG00000028070	Naxe	20
ENSMUSG00000029090	Adgra3	20		ENSMUSG00000020576	Nbas	20
ENSMUSG00000034730	Adgrb1	20		ENSMUSG00000086316	Nbdy	8
ENSMUSG00000028782	Adgrb2	9		ENSMUSG00000027799	Nbea	7
ENSMUSG00000033569	Adgrb3	9		ENSMUSG00000073664	Nbeal1	12
ENSMUSG00000044017	Adgrd1	20		ENSMUSG00000056724	Nbeal2	20
ENSMUSG00000004730	Adgre1	9		ENSMUSG00000041120	Nbl1	20
ENSMUSG00000002885	Adgre5	20		ENSMUSG00000028224	Nbn	20
ENSMUSG00000057899	Adgrf2	20		ENSMUSG00000017119	Nbr1	20

ENSMUSG00000023918	Adgrf4	9		ENSMUSG00000051359	Ncald	5
ENSMUSG00000056492	Adgrf5	18		ENSMUSG00000039542	Ncam1	20
ENSMUSG00000031785	Adgrg1	9		ENSMUSG00000022762	Ncam2	20
ENSMUSG00000031298	Adgrg2	9		ENSMUSG00000002341	Ncan	6
ENSMUSG00000060470	Adgrg3	20		ENSMUSG00000038252	Ncapd2	6
ENSMUSG00000039116	Adgrg6	2		ENSMUSG00000035024	Ncapd3	20
ENSMUSG00000013033	Adgrl1	20		ENSMUSG00000015880	Ncapg	20
ENSMUSG00000028184	Adgrl2	20		ENSMUSG00000042029	Ncapg2	20
ENSMUSG00000037605	Adgrl3	7		ENSMUSG00000034906	Ncaph	9
ENSMUSG00000039167	Adgrl4	20		ENSMUSG00000008690	Ncaph2	20
ENSMUSG00000069170	Adgrv1	13		ENSMUSG00000028330	Ncbp1	20
ENSMUSG00000028138	Adh5	20		ENSMUSG00000022774	Ncbp2	20
ENSMUSG00000025911	Adhfe1	20		ENSMUSG00000020783	Ncbp3	1
ENSMUSG00000020629	Adi1	20		ENSMUSG00000028833	Ncdn	9
ENSMUSG00000026457	Adipor1	20		ENSMUSG00000027698	Nceh1	1
ENSMUSG00000030168	Adipor2	20		ENSMUSG00000015950	Ncf1	19
ENSMUSG00000039197	Adk	20		ENSMUSG00000026480	Ncf2	20
ENSMUSG00000030790	Adm	20		ENSMUSG00000071715	Ncf4	20
ENSMUSG00000051149	Adnp	2		ENSMUSG00000032475	Nck1	20
ENSMUSG00000053950	Adnp2	20		ENSMUSG00000066877	Nck2	20
ENSMUSG00000042429	Adora1	1		ENSMUSG00000027002	Nckap1	9
ENSMUSG00000020178	Adora2a	9		ENSMUSG00000022488	Nckap1l	9
ENSMUSG00000018500	Adora2b	20		ENSMUSG00000049690	Nckap5	8
ENSMUSG00000025236	Adpgk	20		ENSMUSG00000023009	Nckap5l	20
ENSMUSG00000002844	Adprh	20		ENSMUSG00000026234	Ncl	3
ENSMUSG00000031448	Adprhl1	20		ENSMUSG00000020238	Ncln	20
ENSMUSG00000042558	Adprhl2	20		ENSMUSG00000043924	Ncmap	20
ENSMUSG00000020910	Adprm	20		ENSMUSG00000020647	Ncoa1	2
ENSMUSG00000045875	Adra1a	20		ENSMUSG00000005886	Ncoa2	20
ENSMUSG00000050541	Adra1b	18		ENSMUSG00000027678	Ncoa3	20
ENSMUSG00000027335	Adra1d	20		ENSMUSG00000056234	Ncoa4	7
ENSMUSG00000033717	Adra2a	18		ENSMUSG00000039804	Ncoa5	18
ENSMUSG00000058620	Adra2b	18		ENSMUSG00000038369	Ncoa6	20
ENSMUSG00000045318	Adra2c	9		ENSMUSG00000039697	Ncoa7	20
ENSMUSG00000035283	Adrb1	20		ENSMUSG00000018501	Ncor1	5
ENSMUSG00000045730	Adrb2	20		ENSMUSG00000029478	Ncor2	19
ENSMUSG00000039041	Adrm1	3		ENSMUSG00000062661	Ncs1	18
ENSMUSG00000022407	Adsl	20		ENSMUSG00000003458	Ncstn	20
ENSMUSG00000015961	Adss	20		ENSMUSG00000028614	Ndc1	20
ENSMUSG00000011148	Adssl1	20		ENSMUSG00000024056	Ndc80	20
ENSMUSG00000020473	Aebp1	14		ENSMUSG00000022678	Nde1	20
ENSMUSG00000030232	Aebp2	20		ENSMUSG00000018736	Ndel1	20
ENSMUSG00000030609	Aen	20		ENSMUSG00000024425	Ndfip1	9
ENSMUSG00000077711	AF357399	20		ENSMUSG00000053253	Ndfip2	9
ENSMUSG00000029094	Afap1	9		ENSMUSG00000033585	Ndn	20
ENSMUSG00000033032	Afap1l1	20		ENSMUSG00000049001	Ndnf	20
ENSMUSG00000025083	Afap1l2	20		ENSMUSG00000006471	Ndor1	20
ENSMUSG00000068036	Afdn	18		ENSMUSG00000040138	Ndp	20
ENSMUSG00000029313	Aff1	20		ENSMUSG00000005125	Ndrg1	16
ENSMUSG00000031189	Aff2	3		ENSMUSG00000004558	Ndrg2	1
ENSMUSG00000037138	Aff3	9		ENSMUSG00000027634	Ndrg3	9
ENSMUSG00000049470	Aff4	2		ENSMUSG00000036564	Ndrg4	1
ENSMUSG00000038302	Afg1l	20		ENSMUSG00000054008	Ndst1	3
ENSMUSG00000031967	Afg3l1	12		ENSMUSG00000039308	Ndst2	12

ENSMUSG00000024527	Afg3l2	20		ENSMUSG00000027977	Ndst3	8
ENSMUSG00000017718	Afmid	1		ENSMUSG00000027971	Ndst4	5
ENSMUSG00000049659	Aftph	20		ENSMUSG00000016427	Ndufa1	10
ENSMUSG00000031521	Aga	20		ENSMUSG00000026260	Ndufa10	2
ENSMUSG00000055013	Agap1	20		ENSMUSG00000002379	Ndufa11	12
ENSMUSG00000025422	Agap2	9		ENSMUSG00000068487	Ndufa11b	1
ENSMUSG00000023353	Agap3	20		ENSMUSG00000020022	Ndufa12	20
ENSMUSG00000025754	Agbl1	20		ENSMUSG00000036199	Ndufa13	12
ENSMUSG00000040812	Agbl2	9		ENSMUSG00000014294	Ndufa2	12
ENSMUSG00000038836	Agbl3	20		ENSMUSG00000035674	Ndufa3	12
ENSMUSG00000061298	Agbl4	20		ENSMUSG00000029632	Ndufa4	10
ENSMUSG00000029165	Agbl5	3		ENSMUSG00000040280	Ndufa4l2	20
ENSMUSG00000015452	Ager	20		ENSMUSG00000023089	Ndufa5	12
ENSMUSG00000026159	Agfg1	3		ENSMUSG00000022450	Ndufa6	12
ENSMUSG00000029722	Agfg2	1		ENSMUSG00000041881	Ndufa7	12
ENSMUSG00000021681	Aggf1	20		ENSMUSG00000026895	Ndufa8	20
ENSMUSG00000029916	Agk	20		ENSMUSG00000000399	Ndufa9	20
ENSMUSG00000033400	Agl	9		ENSMUSG00000030869	Ndufab1	12
ENSMUSG00000040706	Agmat	20		ENSMUSG00000091989	Ndufab1-ps	20
ENSMUSG00000050103	Agmo	9		ENSMUSG00000027305	Ndufaf1	20
ENSMUSG00000041530	Ago1	5		ENSMUSG00000068184	Ndufaf2	2
ENSMUSG00000036698	Ago2	20		ENSMUSG00000070283	Ndufaf3	20
ENSMUSG00000028842	Ago3	3		ENSMUSG00000028261	Ndufaf4	18
ENSMUSG00000042500	Ago4	9		ENSMUSG00000027384	Ndufaf5	20
ENSMUSG00000034254	Agpat1	20		ENSMUSG00000050323	Ndufaf6	20
ENSMUSG00000026922	Agpat2	20		ENSMUSG00000024082	Ndufaf7	1
ENSMUSG00000001211	Agpat3	18		ENSMUSG00000078572	Ndufaf8	19
ENSMUSG00000023827	Agpat4	16		ENSMUSG00000113902	Ndufb1-ps	12
ENSMUSG00000031467	Agpat5	3		ENSMUSG00000040048	Ndufb10	3
ENSMUSG00000042410	Agps	1		ENSMUSG00000031059	Ndufb11	12
ENSMUSG00000041936	Agrn	19		ENSMUSG00000002416	Ndufb2	12
ENSMUSG00000031980	Agt	6		ENSMUSG00000026032	Ndufb3	12
ENSMUSG00000021557	Agtpbp1	12		ENSMUSG00000022820	Ndufb4	2
ENSMUSG00000049115	Agtr1a	1		ENSMUSG00000083380	Ndufb4c	12
ENSMUSG00000068122	Agtr2	6		ENSMUSG00000027673	Ndufb5	20
ENSMUSG00000029007	Agtrap	20		ENSMUSG00000071014	Ndufb6	12
ENSMUSG00000026491	Ahctf1	6		ENSMUSG00000033938	Ndufb7	12
ENSMUSG00000027597	Ahcy	20		ENSMUSG00000025204	Ndufb8	12
ENSMUSG00000027893	Ahcyl1	1		ENSMUSG00000022354	Ndufb9	12
ENSMUSG00000029772	Ahcyl2	20		ENSMUSG00000037152	Ndufc1	12
ENSMUSG00000037692	Ahdc1	20		ENSMUSG00000030647	Ndufc2	12
ENSMUSG00000019986	Ahi1	20		ENSMUSG00000025968	Ndufs1	20
ENSMUSG00000069833	Ahnak	20		ENSMUSG00000013593	Ndufs2	20
ENSMUSG00000019256	Ahr	20		ENSMUSG00000021764	Ndufs4	12
ENSMUSG00000021575	Ahrr	20		ENSMUSG00000028648	Ndufs5	12
ENSMUSG00000021037	Ahsa1	12		ENSMUSG00000021606	Ndufs6	10
ENSMUSG00000020288	Ahsa2	20		ENSMUSG00000020153	Ndufs7	3
ENSMUSG00000035875	AI182371	20		ENSMUSG00000059734	Ndufs8	12
ENSMUSG00000114780	AI197445	9		ENSMUSG00000037916	Ndufv1	20
ENSMUSG00000062753	AI413582	12		ENSMUSG00000024099	Ndufv2	12
ENSMUSG00000074384	AI429214	12		ENSMUSG00000024038	Ndufv3	12
ENSMUSG00000045165	AI467606	20		ENSMUSG00000092274	Neat1	16
ENSMUSG00000090086	AI480526	1		ENSMUSG00000026950	Neb	19
ENSMUSG00000056145	AI504432	2		ENSMUSG00000053702	Nebl	7

ENSMUSG00000105987	AI506816	20		ENSMUSG00000040536	Necab1	20
ENSMUSG00000078307	AI593442	8		ENSMUSG00000031837	Necab2	19
ENSMUSG00000010290	AI597479	1		ENSMUSG00000027489	Necab3	18
ENSMUSG00000093738	AI606473	1		ENSMUSG00000030327	Necap1	20
ENSMUSG00000034382	AI661453	20		ENSMUSG00000028923	Necap2	3
ENSMUSG00000047423	AI837181	20		ENSMUSG00000032012	Nectin1	19
ENSMUSG00000097280	AI849053	1		ENSMUSG00000062300	Nectin2	20
ENSMUSG00000053297	AI854703	18		ENSMUSG00000022656	Nectin3	5
ENSMUSG00000056383	AI987944	19		ENSMUSG00000006411	Nectin4	20
ENSMUSG00000042901	Aida	20		ENSMUSG00000019988	Nedd1	20
ENSMUSG00000024397	Aif1	18		ENSMUSG00000032216	Nedd4	20
ENSMUSG00000001864	Aif1l	20		ENSMUSG00000024589	Nedd4l	9
ENSMUSG00000036932	Aifm1	9		ENSMUSG00000010376	Nedd8	7
ENSMUSG00000020085	Aifm2	9		ENSMUSG00000021365	Nedd9	20
ENSMUSG00000022763	Aifm3	6		ENSMUSG00000020396	Nefh	18
ENSMUSG00000019806	Aig1	15		ENSMUSG00000022055	Nefl	19
ENSMUSG00000037860	Aim2	20		ENSMUSG00000022054	Nefm	18
ENSMUSG00000028029	Aimp1	12		ENSMUSG00000040037	Negr1	7
ENSMUSG00000029610	Aimp2	20		ENSMUSG00000032298	Neil1	20
ENSMUSG00000000731	Aire	20		ENSMUSG00000035121	Neil2	20
ENSMUSG00000078247	Airn	9		ENSMUSG00000039396	Neil3	20
ENSMUSG00000039546	Ajap1	3		ENSMUSG00000031644	Nek1	20
ENSMUSG00000029419	Ajm1	9		ENSMUSG00000042567	Nek10	20
ENSMUSG00000022178	Ajuba	20		ENSMUSG00000035032	Nek11	14
ENSMUSG00000026817	Ak1	18		ENSMUSG00000031478	Nek3	20
ENSMUSG00000078139	AK157302	20		ENSMUSG00000021918	Nek4	6
ENSMUSG00000028792	Ak2	20		ENSMUSG00000037738	Nek5	13
ENSMUSG00000024782	Ak3	1		ENSMUSG00000026749	Nek6	20
ENSMUSG00000081957	Ak3l2-ps	5		ENSMUSG00000026393	Nek7	3
ENSMUSG00000028527	Ak4	1		ENSMUSG00000017405	Nek8	20
ENSMUSG00000039058	Ak5	5		ENSMUSG00000034290	Nek9	12
ENSMUSG00000078941	Ak6	20		ENSMUSG00000029111	Nelfa	20
ENSMUSG00000041323	Ak7	14		ENSMUSG00000013465	Nelfb	9
ENSMUSG00000026807	Ak8	13		ENSMUSG00000016253	Nelfcd	20
ENSMUSG00000091415	Ak9	14		ENSMUSG00000024369	Nelfe	20
ENSMUSG00000091636	Akain1	19		ENSMUSG00000055409	Nell1	18
ENSMUSG00000018428	Akap1	20		ENSMUSG00000022454	Nell2	3
ENSMUSG00000047804	Akap10	3		ENSMUSG00000020982	Nemf	20
ENSMUSG00000022016	Akap11	7		ENSMUSG00000040195	Nemp1	1
ENSMUSG00000038587	Akap12	18		ENSMUSG00000043015	Nemp2	20
ENSMUSG00000066406	Akap13	1		ENSMUSG00000037499	Nenf	12
ENSMUSG00000059708	Akap17b	20		ENSMUSG00000032340	Neo1	18
ENSMUSG00000021057	Akap5	9		ENSMUSG00000036208	Nepro	1
ENSMUSG00000061603	Akap6	12		ENSMUSG00000004891	Nes	20
ENSMUSG00000039166	Akap7	6		ENSMUSG00000021215	Net1	20
ENSMUSG00000024045	Akap8	20		ENSMUSG00000050321	Neto1	9
ENSMUSG00000002625	Akap8l	1		ENSMUSG00000036902	Neto2	9
ENSMUSG00000040407	Akap9	9		ENSMUSG00000007038	Neu1	20
ENSMUSG00000031023	Akip1	20		ENSMUSG00000079434	Neu2	20
ENSMUSG00000023075	Akirin1	20		ENSMUSG00000035239	Neu3	20
ENSMUSG00000028291	Akirin2	20		ENSMUSG00000034000	Neu4	20
ENSMUSG00000039158	Akna	20		ENSMUSG00000006435	Neurl1a	18
ENSMUSG00000028692	Akr1a1	7		ENSMUSG00000034413	Neurl1b	1
ENSMUSG00000061758	Akr1b10	6		ENSMUSG00000039873	Neurl2	20

ENSMUSG00000001642	Akr1b3	20		ENSMUSG00000047180	Neurl3	10
ENSMUSG00000029762	Akr1b8	20		ENSMUSG00000047284	Neurl4	20
ENSMUSG00000033715	Akr1c14	1		ENSMUSG00000034701	Neurod1	5
ENSMUSG00000045410	Akr1e1	1		ENSMUSG00000038255	Neurod2	1
ENSMUSG00000028743	Akr7a5	20		ENSMUSG00000037984	Neurod6	5
ENSMUSG00000001729	Akt1	20		ENSMUSG00000046449	Nexmif	3
ENSMUSG00000011096	Akt1s1	20		ENSMUSG00000039103	Nexn	20
ENSMUSG00000004056	Akt2	9		ENSMUSG00000020716	Nf1	3
ENSMUSG00000084347	Akt2-ps	9		ENSMUSG00000009073	Nf2	20
ENSMUSG00000019699	Akt3	9		ENSMUSG00000058099	Nfam1	9
ENSMUSG00000031667	Aktip	20		ENSMUSG00000026442	Nfasc	18
ENSMUSG00000118394	AL607088.1	9		ENSMUSG00000003847	Nfat5	10
ENSMUSG00000118399	AL844849.1	20		ENSMUSG00000033016	Nfatc1	20
ENSMUSG00000028393	Alad	20		ENSMUSG00000027544	Nfatc2	9
ENSMUSG00000032786	Alas1	3		ENSMUSG00000030722	Nfatc2ip	20
ENSMUSG00000025270	Alas2	20		ENSMUSG00000031902	Nfatc3	20
ENSMUSG00000022636	Alcam	9		ENSMUSG00000023411	Nfatc4	20
ENSMUSG00000007833	Aldh16a1	20		ENSMUSG00000038615	Nfe2l1	20
ENSMUSG00000025007	Aldh18a1	20		ENSMUSG00000015839	Nfe2l2	20
ENSMUSG00000053279	Aldh1a1	20		ENSMUSG00000029832	Nfe2l3	1
ENSMUSG00000013584	Aldh1a2	20		ENSMUSG00000028565	Nfia	19
ENSMUSG00000024747	Aldh1a7	20		ENSMUSG00000008575	Nfib	5
ENSMUSG00000035561	Aldh1b1	18		ENSMUSG00000055053	Nfic	18
ENSMUSG00000030088	Aldh1l1	1		ENSMUSG00000056749	Nfil3	9
ENSMUSG00000020256	Aldh1l2	20		ENSMUSG00000001911	Nfix	5
ENSMUSG00000029455	Aldh2	20		ENSMUSG00000028163	Nfkb1	12
ENSMUSG00000010025	Aldh3a2	1		ENSMUSG00000025225	Nfkb2	20
ENSMUSG00000024885	Aldh3b1	20		ENSMUSG00000021025	Nfkbia	20
ENSMUSG00000028737	Aldh4a1	20		ENSMUSG00000030595	Nfkbib	20
ENSMUSG00000035936	Aldh5a1	20		ENSMUSG00000036931	Nfkbid	20
ENSMUSG00000021238	Aldh6a1	20		ENSMUSG00000023947	Nfkbie	20
ENSMUSG00000053644	Aldh7a1	20		ENSMUSG00000042419	Nfkbil1	20
ENSMUSG00000037542	Aldh8a1	20		ENSMUSG00000035356	Nfkbiz	9
ENSMUSG00000026687	Aldh9a1	9		ENSMUSG00000042185	Nfrkb	20
ENSMUSG00000030695	Aldoa	12		ENSMUSG00000027618	Nfs1	20
ENSMUSG00000114515	Aldoa	20		ENSMUSG00000029993	Nfu1	20
ENSMUSG00000059343	Aldoart1	10		ENSMUSG00000028423	Nfx1	9
ENSMUSG00000028307	Aldob	19		ENSMUSG00000072889	Nfxl1	1
ENSMUSG00000017390	Aldoc	18		ENSMUSG00000020248	Nfyb	18
ENSMUSG00000039427	Alg1	12		ENSMUSG00000032897	Nfyc	1
ENSMUSG00000075470	Alg10b	7		ENSMUSG00000021032	Ngb	17
ENSMUSG00000063362	Alg11	20		ENSMUSG00000022204	Ngdn	20
ENSMUSG00000035845	Alg12	18		ENSMUSG00000026259	Ngef	9
ENSMUSG00000041718	Alg13	20		ENSMUSG00000027859	Ngf	20
ENSMUSG00000039887	Alg14	20		ENSMUSG00000000120	Ngfr	19
ENSMUSG00000039740	Alg2	20		ENSMUSG00000021785	Ngly1	3
ENSMUSG00000033809	Alg3	5		ENSMUSG00000047084	Ngrn	20
ENSMUSG00000036632	Alg5	12		ENSMUSG00000026162	Nhej1	20
ENSMUSG00000035704	Alg8	20		ENSMUSG00000048540	Nhlh2	19
ENSMUSG00000032059	Alg9	1		ENSMUSG00000044231	Nhlrc1	20
ENSMUSG00000055471	Alk	18		ENSMUSG00000025078	Nhlrc2	1
ENSMUSG00000079036	Alkbh1	20		ENSMUSG00000042997	Nhlrc3	20
ENSMUSG00000044339	Alkbh2	3		ENSMUSG00000090113	Nhlrc4	18
ENSMUSG00000040174	Alkbh3	18		ENSMUSG00000001056	Nhp2	3

ENSMUSG00000039754	Alkbh4	20		ENSMUSG00000059493	Nhs	20
ENSMUSG00000042650	Alkbh5	12		ENSMUSG00000039835	Nhsl1	19
ENSMUSG00000042831	Alkbh6	12		ENSMUSG00000079481	Nhsl2	8
ENSMUSG00000002661	Alkbh7	12		ENSMUSG00000032606	Nicn1	20
ENSMUSG00000025899	Alkbh8	20		ENSMUSG00000005397	Nid1	2
ENSMUSG00000063810	Alms1	1		ENSMUSG00000021806	Nid2	13
ENSMUSG00000090256	Alms1-ps1	20		ENSMUSG00000026036	Nif3l1	1
ENSMUSG00000000320	Alox12	20		ENSMUSG00000026377	Nifk	20
ENSMUSG00000032807	Alox12b	20		ENSMUSG00000095930	Nim1k	20
ENSMUSG00000025701	Alox5	20		ENSMUSG00000021068	Nin	7
ENSMUSG00000060063	Alox5ap	20		ENSMUSG00000037966	Ninj1	20
ENSMUSG00000020891	Alox8	20		ENSMUSG00000041377	Ninj2	20
ENSMUSG00000020892	Aloxe3	20		ENSMUSG00000068115	Ninl	20
ENSMUSG00000028028	Alpk1	9		ENSMUSG00000031917	Nip7	9
ENSMUSG00000032845	Alpk2	20		ENSMUSG00000047037	Nipa1	20
ENSMUSG00000038763	Alpk3	20		ENSMUSG00000030452	Nipa2	20
ENSMUSG00000028766	Alpl	20		ENSMUSG00000038879	Nipal2	18
ENSMUSG00000026024	Als2	20		ENSMUSG00000028803	Nipal3	20
ENSMUSG00000044037	Als2cl	9		ENSMUSG00000020411	Nipal4	16
ENSMUSG00000040310	Alx4	9		ENSMUSG00000022141	Nipbl	2
ENSMUSG00000025134	Alyref	20		ENSMUSG00000034285	Nipsnap1	20
ENSMUSG00000060244	Alyref2	20		ENSMUSG00000029432	Nipsnap2	19
ENSMUSG00000022244	Amacr	20		ENSMUSG00000015247	Nipsnap3b	20
ENSMUSG00000040506	Ambra1	20		ENSMUSG00000021910	Nisch	12
ENSMUSG00000060096	Amd-ps3	20		ENSMUSG00000013997	Nit1	20
ENSMUSG00000075232	Amd1	20		ENSMUSG00000022751	Nit2	9
ENSMUSG00000063953	Amd2	20		ENSMUSG00000078532	Nkain1	10
ENSMUSG00000036820	Amdhd2	20		ENSMUSG00000069670	Nkain2	20
ENSMUSG00000050332	Amer1	20		ENSMUSG00000055761	Nkain3	20
ENSMUSG00000021986	Amer2	7		ENSMUSG00000027574	Nkain4	20
ENSMUSG00000045174	Amer3	20		ENSMUSG00000016409	Nkap	20
ENSMUSG00000031751	Amfr	20		ENSMUSG00000059820	Nkapd1	20
ENSMUSG00000035262	Amh	20		ENSMUSG00000059395	Nkapl	20
ENSMUSG00000023047	Amhr2	20		ENSMUSG00000031661	Nkd1	9
ENSMUSG00000050947	Amigo1	20		ENSMUSG00000021567	Nkd2	20
ENSMUSG00000048218	Amigo2	19		ENSMUSG00000021772	Nkiras1	7
ENSMUSG00000032593	Amigo3	20		ENSMUSG00000017837	Nkiras2	20
ENSMUSG00000042225	Ammecr1	1		ENSMUSG00000060621	Nkpd1	9
ENSMUSG00000041915	Ammecr1l	9		ENSMUSG00000044149	Nkrf	3
ENSMUSG00000021278	Amn	20		ENSMUSG00000032525	Nktr	20
ENSMUSG00000068250	Amn1	20		ENSMUSG00000001496	Nkx2-1	20
ENSMUSG00000041688	Amot	19		ENSMUSG00000027434	Nkx2-2	1
ENSMUSG00000013076	Amotl1	18		ENSMUSG00000086509	Nkx2-2os	20
ENSMUSG00000032531	Amotl2	20		ENSMUSG00000058669	Nkx2-9	20
ENSMUSG00000027889	Ampd2	20		ENSMUSG00000022061	Nkx3-1	19
ENSMUSG00000005686	Ampd3	20		ENSMUSG00000035187	Nkx6-1	1
ENSMUSG00000021314	Amph	19		ENSMUSG00000041309	Nkx6-2	16
ENSMUSG00000032607	Amt	1		ENSMUSG00000020692	Nle1	20
ENSMUSG00000074264	Amy1	20		ENSMUSG00000063887	Nlgn1	9
ENSMUSG00000050022	Amz1	9		ENSMUSG00000051790	Nlgn2	20
ENSMUSG00000020610	Amz2	18		ENSMUSG00000031302	Nlgn3	4
ENSMUSG00000014355	Anapc1	1		ENSMUSG00000017376	Nlk	6
ENSMUSG00000036977	Anapc10	20		ENSMUSG00000021710	Nln	20
ENSMUSG00000025135	Anapc11	12		ENSMUSG00000049871	Nlrc3	20

ENSMUSG00000035048	Anapc13	12		ENSMUSG00000074151	Nlrc5	20
ENSMUSG00000030649	Anapc15	2		ENSMUSG00000049709	Nlrp10	20
ENSMUSG00000074780	Anapc15-ps	18		ENSMUSG00000070390	Nlrp1b	20
ENSMUSG00000020107	Anapc16	2		ENSMUSG00000092528	Nlrp1c-ps	20
ENSMUSG00000026965	Anapc2	20		ENSMUSG00000032691	Nlrp3	20
ENSMUSG00000029176	Anapc4	20		ENSMUSG00000041596	Nlrp5-ps	1
ENSMUSG00000029472	Anapc5	12		ENSMUSG00000038745	Nlrp6	20
ENSMUSG00000029466	Anapc7	20		ENSMUSG00000032109	Nlrx1	20
ENSMUSG00000072115	Ang	20		ENSMUSG00000025723	Nmb	20
ENSMUSG00000021257	Angel1	20		ENSMUSG00000019865	Nmbr	9
ENSMUSG00000026634	Angel2	9		ENSMUSG00000027787	Nmd3	1
ENSMUSG00000022309	Angpt1	9		ENSMUSG00000037601	Nme1	20
ENSMUSG00000031465	Angpt2	18		ENSMUSG00000020857	Nme2	12
ENSMUSG00000033544	Angptl1	20		ENSMUSG00000024177	Nme4	20
ENSMUSG00000004105	Angptl2	13		ENSMUSG00000035984	Nme5	20
ENSMUSG00000028553	Angptl3	20		ENSMUSG00000032478	Nme6	20
ENSMUSG00000002289	Angptl4	20		ENSMUSG00000026575	Nme7	20
ENSMUSG00000038742	Angptl6	3		ENSMUSG00000026946	Nmi	20
ENSMUSG00000028989	Angptl7	20		ENSMUSG00000028992	Nmnat1	20
ENSMUSG00000022265	Ank	20		ENSMUSG00000042751	Nmnat2	18
ENSMUSG00000031543	Ank1	18		ENSMUSG00000032456	Nmnat3	3
ENSMUSG00000032826	Ank2	19		ENSMUSG00000063445	Nmral1	20
ENSMUSG00000069601	Ank3	20		ENSMUSG00000037847	Nmrk1	20
ENSMUSG00000066510	Ankdd1a	3		ENSMUSG00000020936	Nmt1	4
ENSMUSG00000047117	Ankdd1b	20		ENSMUSG00000026643	Nmt2	19
ENSMUSG00000074771	Ankef1	20		ENSMUSG00000067786	Nnat	1
ENSMUSG00000047773	Ankfn1	10		ENSMUSG00000025453	Nnt	20
ENSMUSG00000020790	Ankfy1	2		ENSMUSG00000036285	Noa1	20
ENSMUSG00000024483	Ankhd1	20		ENSMUSG00000003848	Nob1	20
ENSMUSG00000040351	Ankib1	14		ENSMUSG00000095567	Noc2l	3
ENSMUSG00000029501	Ankle2	20		ENSMUSG00000024999	Noc3l	20
ENSMUSG00000034212	Ankmy1	20		ENSMUSG00000033294	Noc4l	20
ENSMUSG00000036188	Ankmy2	20		ENSMUSG00000023087	Noct	20
ENSMUSG00000021661	Ankra2	20		ENSMUSG00000038058	Nod1	9
ENSMUSG00000031508	Ankrd10	20		ENSMUSG00000055994	Nod2	20
ENSMUSG00000035569	Ankrd11	1		ENSMUSG00000048616	Nog	20
ENSMUSG00000034647	Ankrd12	20		ENSMUSG00000061458	Nol10	1
ENSMUSG00000041870	Ankrd13a	20		ENSMUSG00000018433	Nol11	1
ENSMUSG00000037907	Ankrd13b	3		ENSMUSG00000033099	Nol12	20
ENSMUSG00000039988	Ankrd13c	12		ENSMUSG00000014776	Nol3	20
ENSMUSG00000005986	Ankrd13d	7		ENSMUSG00000041923	Nol4	18
ENSMUSG00000047909	Ankrd16	20		ENSMUSG00000061411	Nol4l	20
ENSMUSG00000055204	Ankrd17	7		ENSMUSG00000028430	Nol6	20
ENSMUSG00000067653	Ankrd23	20		ENSMUSG00000063200	Nol7	12
ENSMUSG00000054708	Ankrd24	18		ENSMUSG00000021392	Nol8	20
ENSMUSG00000007827	Ankrd26	20		ENSMUSG00000028948	Nol9	1
ENSMUSG00000034867	Ankrd27	9		ENSMUSG00000015176	Nolc1	20
ENSMUSG00000014496	Ankrd28	20		ENSMUSG00000001569	Nom1	20
ENSMUSG00000057766	Ankrd29	3		ENSMUSG00000030835	Nomo1	1
ENSMUSG00000022237	Ankrd33b	17		ENSMUSG00000031311	Nono	20
ENSMUSG00000049097	Ankrd34a	4		ENSMUSG00000027133	Nop10	12
ENSMUSG00000045034	Ankrd34b	20		ENSMUSG00000036693	Nop14	9
ENSMUSG00000047606	Ankrd34c	18		ENSMUSG00000025869	Nop16	20
ENSMUSG00000038354	Ankrd35	1		ENSMUSG00000038279	Nop2	20

ENSMUSG00000050914	Ankrd37	20		ENSMUSG00000041560	Nop53	3
ENSMUSG00000079610	Ankrd39	20		ENSMUSG00000027405	Nop56	20
ENSMUSG00000020864	Ankrd40	20		ENSMUSG00000026020	Nop58	20
ENSMUSG00000041343	Ankrd42	3		ENSMUSG00000019297	Nop9	20
ENSMUSG00000052331	Ankrd44	20		ENSMUSG00000029361	Nos1	18
ENSMUSG00000044835	Ankrd45	1		ENSMUSG00000038473	Nos1ap	1
ENSMUSG00000048307	Ankrd46	20		ENSMUSG00000028978	Nos3	20
ENSMUSG00000031931	Ankrd49	20		ENSMUSG00000003421	Nosip	3
ENSMUSG00000044864	Ankrd50	20		ENSMUSG00000034738	Nostrin	20
ENSMUSG00000014498	Ankrd52	19		ENSMUSG00000026923	Notch1	20
ENSMUSG00000033055	Ankrd54	20		ENSMUSG00000027878	Notch2	13
ENSMUSG00000049985	Ankrd55	9		ENSMUSG00000038146	Notch3	20
ENSMUSG00000040183	Ankrd6	20		ENSMUSG00000015468	Notch4	20
ENSMUSG00000029607	Ankrd61	12		ENSMUSG00000042988	Notum	20
ENSMUSG00000078137	Ankrd63	9		ENSMUSG00000053613	Notumos	20
ENSMUSG00000096140	Ankrd66	14		ENSMUSG00000021047	Nova1	3
ENSMUSG00000037904	Ankrd9	9		ENSMUSG00000030411	Nova2	3
ENSMUSG00000024219	Anks1	20		ENSMUSG00000030562	Nox4	20
ENSMUSG00000058589	Anks1b	9		ENSMUSG00000019320	Noxo1	20
ENSMUSG00000022515	Anks3	12		ENSMUSG00000072919	Noxred1	20
ENSMUSG00000066191	Anks6	20		ENSMUSG00000001988	Npas1	20
ENSMUSG00000074591	Ankub1	20		ENSMUSG00000026077	Npas2	9
ENSMUSG00000026199	Ankzf1	20		ENSMUSG00000021010	Npas3	18
ENSMUSG00000036777	Anln	15		ENSMUSG00000045903	Npas4	20
ENSMUSG00000031075	Ano1	20		ENSMUSG00000033054	Npat	1
ENSMUSG00000037949	Ano10	18		ENSMUSG00000044034	Npb	2
ENSMUSG00000038115	Ano2	9		ENSMUSG00000033774	Npbwr1	20
ENSMUSG00000074968	Ano3	9		ENSMUSG00000024413	Npc1	9
ENSMUSG00000035189	Ano4	18		ENSMUSG00000020447	Npc1l1	12
ENSMUSG00000055489	Ano5	18		ENSMUSG00000021242	Npc2	5
ENSMUSG00000064210	Ano6	20		ENSMUSG00000015094	Npdc1	20
ENSMUSG00000034107	Ano7	20		ENSMUSG00000039263	Npepl1	20
ENSMUSG00000034863	Ano8	1		ENSMUSG00000001441	Npepps	20
ENSMUSG00000069913	Anp32-ps	20		ENSMUSG00000020090	Npffr1	20
ENSMUSG00000032249	Anp32a	20		ENSMUSG00000035528	Npffr2	20
ENSMUSG00000028333	Anp32b	20		ENSMUSG00000027378	Nphp1	20
ENSMUSG00000015749	Anp32e	20		ENSMUSG00000032558	Nphp3	9
ENSMUSG00000039062	Anpep	20		ENSMUSG00000039577	Nphp4	20
ENSMUSG00000033420	Antxr1	13		ENSMUSG00000006649	Nphs1	9
ENSMUSG00000029338	Antxr2	20		ENSMUSG00000087009	Nphs1os	20
ENSMUSG00000031635	Anxa10	20		ENSMUSG00000042684	Npl	20
ENSMUSG00000021866	Anxa11	5		ENSMUSG00000039703	Nploc4	12
ENSMUSG00000032231	Anxa2	20		ENSMUSG00000057113	Npm1	12
ENSMUSG00000029484	Anxa3	20		ENSMUSG00000047911	Npm2	20
ENSMUSG00000029994	Anxa4	20		ENSMUSG00000056209	Npm3	20
ENSMUSG00000027712	Anxa5	9		ENSMUSG00000081669	Npm3-ps1	20
ENSMUSG00000018340	Anxa6	20		ENSMUSG00000040998	Npnt	5
ENSMUSG00000021814	Anxa7	20		ENSMUSG00000026241	Nppc	20
ENSMUSG00000015702	Anxa9	20		ENSMUSG00000027931	Npr1	4
ENSMUSG00000078651	Aoc2	20		ENSMUSG00000028469	Npr2	20
ENSMUSG00000019326	Aoc3	20		ENSMUSG00000022206	Npr3	13
ENSMUSG00000021458	Aopep	20		ENSMUSG00000010057	Nprl2	20
ENSMUSG00000063558	Aox1	20		ENSMUSG00000020289	Nprl3	20
ENSMUSG00000064294	Aox3	20		ENSMUSG00000043659	Npsr1	20

ENSMUSG00000038242	Aox4	20		ENSMUSG00000032336	Nptn	9
ENSMUSG00000074238	Ap1ar	9		ENSMUSG00000025582	Nptx1	5
ENSMUSG00000009090	Ap1b1	9		ENSMUSG00000059991	Nptx2	20
ENSMUSG00000031731	Ap1g1	20		ENSMUSG00000022421	Nptxr	5
ENSMUSG00000040701	Ap1g2	20		ENSMUSG00000029819	Npy	3
ENSMUSG00000003033	Ap1m1	20		ENSMUSG00000036437	Npy1r	10
ENSMUSG00000003309	Ap1m2	20		ENSMUSG00000028004	Npy2r	18
ENSMUSG00000004849	Ap1s1	3		ENSMUSG00000044014	Npy5r	10
ENSMUSG00000031367	Ap1s2	20		ENSMUSG00000003849	Nqo1	9
ENSMUSG00000054702	Ap1s3	20		ENSMUSG00000046949	Nqo2	18
ENSMUSG00000060279	Ap2a1	18		ENSMUSG00000020889	Nr1d1	1
ENSMUSG00000002957	Ap2a2	3		ENSMUSG00000021775	Nr1d2	20
ENSMUSG00000035152	Ap2b1	12		ENSMUSG00000060601	Nr1h2	20
ENSMUSG00000022841	Ap2m1	20		ENSMUSG00000002108	Nr1h3	20
ENSMUSG00000008036	Ap2s1	12		ENSMUSG00000005677	Nr1i3	18
ENSMUSG00000021686	Ap3b1	9		ENSMUSG00000005897	Nr2c1	20
ENSMUSG00000062444	Ap3b2	19		ENSMUSG00000005893	Nr2c2	12
ENSMUSG00000020198	Ap3d1	20		ENSMUSG00000071078	Nr2c2ap	3
ENSMUSG00000021824	Ap3m1	20		ENSMUSG00000019803	Nr2e1	9
ENSMUSG00000094683	Ap3m1-ps	20		ENSMUSG00000069171	Nr2f1	18
ENSMUSG00000031539	Ap3m2	20		ENSMUSG00000030551	Nr2f2	18
ENSMUSG00000024480	Ap3s1	20		ENSMUSG00000002393	Nr2f6	18
ENSMUSG00000085611	Ap3s1-ps1	20		ENSMUSG00000024431	Nr3c1	18
ENSMUSG00000094257	Ap3s1-ps2	20		ENSMUSG00000031618	Nr3c2	4
ENSMUSG00000063801	Ap3s2	9		ENSMUSG00000023034	Nr4a1	20
ENSMUSG00000032952	Ap4b1	20		ENSMUSG00000026826	Nr4a2	20
ENSMUSG00000001998	Ap4e1	1		ENSMUSG00000028341	Nr4a3	20
ENSMUSG00000019518	Ap4m1	20		ENSMUSG00000063972	Nr6a1	20
ENSMUSG00000020955	Ap4s1	20		ENSMUSG00000085201	Nr6a1os	2
ENSMUSG00000049562	Ap5b1	20		ENSMUSG00000032491	Nradd	20
ENSMUSG00000036291	Ap5m1	20		ENSMUSG00000049134	Nrap	20
ENSMUSG00000068264	Ap5s1	20		ENSMUSG00000078202	Nrarp	20
ENSMUSG00000039623	Ap5z1	20		ENSMUSG00000027852	Nras	20
ENSMUSG00000019979	Apaf1	20		ENSMUSG00000029148	Nrbp1	7
ENSMUSG00000024897	Apba1	19		ENSMUSG00000075590	Nrbp2	7
ENSMUSG00000030519	Apba2	19		ENSMUSG00000020598	Nrcam	3
ENSMUSG00000004931	Apba3	9		ENSMUSG00000053510	Nrd1	20
ENSMUSG00000037032	Apbb1	20		ENSMUSG00000021179	Nrde2	20
ENSMUSG00000026786	Apbb1ip	20		ENSMUSG00000042834	Nrep	1
ENSMUSG00000029207	Apbb2	18		ENSMUSG00000058440	Nrf1	20
ENSMUSG00000117679	Apbb3	20		ENSMUSG00000062991	Nrg1	20
ENSMUSG00000005871	Apc	19		ENSMUSG00000060275	Nrg2	9
ENSMUSG00000083152	Apc-ps1	20		ENSMUSG00000041014	Nrg3	9
ENSMUSG00000020135	Apc2	18		ENSMUSG00000032311	Nrg4	20
ENSMUSG00000071847	Apcdd1	5		ENSMUSG00000053310	Nrgn	9
ENSMUSG00000032590	Apeh	20		ENSMUSG00000048490	Nrip1	9
ENSMUSG00000035960	Apex1	9		ENSMUSG00000001520	Nrip2	9
ENSMUSG00000025269	Apex2	20		ENSMUSG00000034825	Nrip3	18
ENSMUSG00000015750	Aph1a	9		ENSMUSG00000059791	Nrm	20
ENSMUSG00000032375	Aph1b	20		ENSMUSG00000039114	Nrn1	9
ENSMUSG00000053040	Aph1c	20		ENSMUSG00000025810	Nrp1	5
ENSMUSG00000027193	Api5	3		ENSMUSG00000025969	Nrp2	5
ENSMUSG00000010911	Apip	20		ENSMUSG00000052384	Nrros	20
ENSMUSG00000030051	Aplf	13		ENSMUSG00000048978	Nrsn1	20

ENSMUSG00000037010	Apln	15		ENSMUSG00000059361	Nrsn2	20
ENSMUSG00000006651	Aplp1	20		ENSMUSG00000039481	Nrtn	1
ENSMUSG00000031996	Aplp2	18		ENSMUSG00000024109	Nrxn1	19
ENSMUSG00000033096	Apmap	20		ENSMUSG00000066392	Nrxn3	9
ENSMUSG00000005681	Apoa2	20		ENSMUSG00000060739	Nsa2	20
ENSMUSG00000040613	Apobec1	20		ENSMUSG00000118353	Nsa2-ps1	20
ENSMUSG00000009585	Apobec3	20		ENSMUSG00000021488	Nsd1	12
ENSMUSG00000042759	Apobr	20		ENSMUSG00000057406	Nsd2	20
ENSMUSG00000040564	Apoc1	7		ENSMUSG00000054823	Nsd3	12
ENSMUSG00000022548	Apod	20		ENSMUSG00000031349	Nsdhl	20
ENSMUSG00000002985	Apoe	3		ENSMUSG00000034187	Nsf	19
ENSMUSG00000056656	Apol8	9		ENSMUSG00000027455	Nsfl1c	20
ENSMUSG00000090698	Apold1	20		ENSMUSG00000029126	Nsg1	2
ENSMUSG00000079508	Apoo	20		ENSMUSG00000020297	Nsg2	18
ENSMUSG00000049233	Apoo-ps	9		ENSMUSG00000062510	Nsl1	1
ENSMUSG00000025525	Apool	20		ENSMUSG00000028245	Nsmaf	9
ENSMUSG00000037787	Apopt1	20		ENSMUSG00000030750	Nsmce1	3
ENSMUSG00000022892	App	20		ENSMUSG00000059586	Nsmce2	19
ENSMUSG00000018481	Appbp2	19		ENSMUSG00000070520	Nsmce3	12
ENSMUSG00000085628	Appbp2os	20		ENSMUSG00000040331	Nsmce4a	9
ENSMUSG00000040760	Appl1	16		ENSMUSG00000006476	Nsmf	18
ENSMUSG00000020263	Appl2	18		ENSMUSG00000037958	Nsrp1	20
ENSMUSG00000006589	Aprt	3		ENSMUSG00000021595	Nsun2	20
ENSMUSG00000110664	Aprt-ps	20		ENSMUSG00000050312	Nsun3	20
ENSMUSG00000028411	Aptx	20		ENSMUSG00000028706	Nsun4	20
ENSMUSG00000004655	Aqp1	13		ENSMUSG00000000916	Nsun5	20
ENSMUSG00000042797	Aqp11	5		ENSMUSG00000026707	Nsun6	20
ENSMUSG00000024411	Aqp4	15		ENSMUSG00000029206	Nsun7	1
ENSMUSG00000032204	Aqp9	20		ENSMUSG00000020736	Nt5c	7
ENSMUSG00000040383	Aqr	7		ENSMUSG00000054958	Nt5c1a	20
ENSMUSG00000046532	Ar	3		ENSMUSG00000025041	Nt5c2	20
ENSMUSG00000001127	Araf	20		ENSMUSG00000029780	Nt5c3	20
ENSMUSG00000032812	Arap1	20		ENSMUSG00000017176	Nt5c3b	20
ENSMUSG00000037999	Arap2	19		ENSMUSG00000039480	Nt5dc1	20
ENSMUSG00000024451	Arap3	9		ENSMUSG00000071547	Nt5dc2	19
ENSMUSG00000022602	Arc	20		ENSMUSG00000054027	Nt5dc3	19
ENSMUSG00000032096	Arcn1	2		ENSMUSG00000032420	Nt5e	20
ENSMUSG00000042350	Arel1	9		ENSMUSG00000032615	Nt5m	9
ENSMUSG00000048076	Arf1	20		ENSMUSG00000022681	Ntan1	20
ENSMUSG00000062421	Arf2	13		ENSMUSG00000049107	Ntf3	5
ENSMUSG00000051853	Arf3	9		ENSMUSG00000041429	Nthl1	20
ENSMUSG00000020440	Arf5	20		ENSMUSG00000059974	Ntm	20
ENSMUSG00000044147	Arf6	9		ENSMUSG00000026857	Ntmt1	9
ENSMUSG00000027575	Arfgap1	20		ENSMUSG00000020902	Ntn1	20
ENSMUSG00000027255	Arfgap2	20		ENSMUSG00000117406	Ntn3	20
ENSMUSG00000054277	Arfgap3	20		ENSMUSG00000020019	Ntn4	20
ENSMUSG00000067851	Arfgef1	3		ENSMUSG00000059857	Ntng1	18
ENSMUSG00000074582	Arfgef2	3		ENSMUSG00000035513	Ntng2	20
ENSMUSG00000019852	Arfgef3	3		ENSMUSG00000031851	Ntpcr	20
ENSMUSG00000074513	Arfip1	20		ENSMUSG00000028072	Ntrk1	19
ENSMUSG00000030881	Arfip2	20		ENSMUSG00000055254	Ntrk2	20
ENSMUSG00000038671	Arfrp1	20		ENSMUSG00000059146	Ntrk3	9
ENSMUSG00000021125	Arg2	1		ENSMUSG00000019890	Nts	8
ENSMUSG00000040459	Arglu1	3		ENSMUSG00000027568	Ntsr1	17

ENSMUSG00000027247	Arhgap1	9		ENSMUSG00000020591	Ntsr2	1
ENSMUSG00000037148	Arhgap10	9		ENSMUSG00000020032	Nuak1	20
ENSMUSG00000041219	Arhgap11a	20		ENSMUSG00000009772	Nuak2	20
ENSMUSG00000041225	Arhgap12	20		ENSMUSG00000028954	Nub1	9
ENSMUSG00000049744	Arhgap15	10		ENSMUSG00000022503	Nubp1	20
ENSMUSG00000030766	Arhgap17	20		ENSMUSG00000039183	Nubp2	20
ENSMUSG00000039031	Arhgap18	20		ENSMUSG00000035142	Nubpl	20
ENSMUSG00000025154	Arhgap19	20		ENSMUSG00000030824	Nucb1	20
ENSMUSG00000053199	Arhgap20	9		ENSMUSG00000030659	Nucb2	18
ENSMUSG00000074354	Arhgap20os	20		ENSMUSG00000026434	Nucks1	18
ENSMUSG00000036591	Arhgap21	5		ENSMUSG00000028851	Nudc	1
ENSMUSG00000063506	Arhgap22	6		ENSMUSG00000110331	Nudc-ps1	20
ENSMUSG00000049807	Arhgap23	15		ENSMUSG00000038736	Nudcd1	18
ENSMUSG00000057315	Arhgap24	18		ENSMUSG00000020328	Nudcd2	20
ENSMUSG00000030047	Arhgap25	20		ENSMUSG00000053838	Nudcd3	20
ENSMUSG00000036452	Arhgap26	18		ENSMUSG00000036639	Nudt1	20
ENSMUSG00000034255	Arhgap27	1		ENSMUSG00000073293	Nudt10	16
ENSMUSG00000085733	Arhgap27os1	20		ENSMUSG00000073295	Nudt11	2
ENSMUSG00000085360	Arhgap27os2	20		ENSMUSG00000024228	Nudt12	3
ENSMUSG00000024043	Arhgap28	20		ENSMUSG00000021809	Nudt13	20
ENSMUSG00000039831	Arhgap29	3		ENSMUSG00000002804	Nudt14	20
ENSMUSG00000048865	Arhgap30	20		ENSMUSG00000033405	Nudt15	20
ENSMUSG00000022799	Arhgap31	20		ENSMUSG00000032565	Nudt16	20
ENSMUSG00000041444	Arhgap32	8		ENSMUSG00000022516	Nudt16l1	3
ENSMUSG00000036882	Arhgap33	19		ENSMUSG00000028100	Nudt17	1
ENSMUSG00000062132	Arhgap33os	20		ENSMUSG00000045211	Nudt18	20
ENSMUSG00000058230	Arhgap35	3		ENSMUSG00000034875	Nudt19	20
ENSMUSG00000036198	Arhgap36	17		ENSMUSG00000028443	Nudt2	20
ENSMUSG00000033697	Arhgap39	5		ENSMUSG00000031754	Nudt21	12
ENSMUSG00000031389	Arhgap4	20		ENSMUSG00000037349	Nudt22	20
ENSMUSG00000074625	Arhgap40	20		ENSMUSG00000024213	Nudt3	20
ENSMUSG00000050730	Arhgap42	3		ENSMUSG00000020029	Nudt4	18
ENSMUSG00000033389	Arhgap44	19		ENSMUSG00000025817	Nudt5	20
ENSMUSG00000035697	Arhgap45	20		ENSMUSG00000050174	Nudt6	20
ENSMUSG00000035133	Arhgap5	20		ENSMUSG00000031767	Nudt7	20
ENSMUSG00000031355	Arhgap6	20		ENSMUSG00000110949	Nudt8	20
ENSMUSG00000078954	Arhgap8	20		ENSMUSG00000029310	Nudt9	20
ENSMUSG00000040345	Arhgap9	20		ENSMUSG00000026683	Nuf2	20
ENSMUSG00000025132	Arhgdia	20		ENSMUSG00000022009	Nufip1	20
ENSMUSG00000030220	Arhgdib	19		ENSMUSG00000037857	Nufip2	3
ENSMUSG00000073433	Arhgdig	20		ENSMUSG00000066306	Numa1	1
ENSMUSG00000040940	Arhgef1	9		ENSMUSG00000021224	Numb	20
ENSMUSG00000071176	Arhgef10	15		ENSMUSG00000063160	Numbl	18
ENSMUSG00000040964	Arhgef10l	9		ENSMUSG00000052798	Nup107	20
ENSMUSG00000041977	Arhgef11	18		ENSMUSG00000039509	Nup133	1
ENSMUSG00000059495	Arhgef12	12		ENSMUSG00000021374	Nup153	20
ENSMUSG00000052921	Arhgef15	20		ENSMUSG00000022142	Nup155	18
ENSMUSG00000029032	Arhgef16	20		ENSMUSG00000051329	Nup160	20
ENSMUSG00000032875	Arhgef17	9		ENSMUSG00000052533	Nup188	20
ENSMUSG00000004568	Arhgef18	20		ENSMUSG00000038759	Nup205	20
ENSMUSG00000028919	Arhgef19	20		ENSMUSG00000030091	Nup210	20
ENSMUSG00000028059	Arhgef2	17		ENSMUSG00000027939	Nup210l	20
ENSMUSG00000019467	Arhgef25	5		ENSMUSG00000001855	Nup214	20
ENSMUSG00000036885	Arhgef26	5		ENSMUSG00000026999	Nup35	9

ENSMUSG00000021662	Arhgef28	20		ENSMUSG00000035351	Nup37	20
ENSMUSG00000021895	Arhgef3	9		ENSMUSG00000016619	Nup50	18
ENSMUSG00000054901	Arhgef33	20		ENSMUSG00000034826	Nup54	20
ENSMUSG00000045094	Arhgef37	20		ENSMUSG00000109511	Nup62	20
ENSMUSG00000051517	Arhgef39	1		ENSMUSG00000020739	Nup85	20
ENSMUSG00000037509	Arhgef4	3		ENSMUSG00000040667	Nup88	18
ENSMUSG00000004562	Arhgef40	18		ENSMUSG00000032939	Nup93	9
ENSMUSG00000033542	Arhgef5	13		ENSMUSG00000063550	Nup98	20
ENSMUSG00000031133	Arhgef6	20		ENSMUSG00000048439	Nupl2	20
ENSMUSG00000031511	Arhgef7	20		ENSMUSG00000030717	Nupr1	12
ENSMUSG00000025656	Arhgef9	9		ENSMUSG00000095789	Nupr1l	20
ENSMUSG00000007880	Arid1a	20		ENSMUSG00000023068	Nus1	9
ENSMUSG00000069729	Arid1b	2		ENSMUSG00000027306	Nusap1	20
ENSMUSG00000033237	Arid2	2		ENSMUSG00000008450	Nutf2	10
ENSMUSG00000019564	Arid3a	20		ENSMUSG00000071497	Nutf2-ps1	20
ENSMUSG00000004661	Arid3b	1		ENSMUSG00000026516	Nvl	20
ENSMUSG00000048118	Arid4a	20		ENSMUSG00000048148	Nwd1	1
ENSMUSG00000039219	Arid4b	20		ENSMUSG00000090061	Nwd2	5
ENSMUSG00000037447	Arid5a	20		ENSMUSG00000010097	Nxf1	20
ENSMUSG00000019947	Arid5b	9		ENSMUSG00000031410	Nxf7	20
ENSMUSG00000025234	Arih1	2		ENSMUSG00000020844	Nxn	3
ENSMUSG00000064145	Arih2	20		ENSMUSG00000075033	Nxpe3	18
ENSMUSG00000060904	Arl1	20		ENSMUSG00000044229	Nxpe4	8
ENSMUSG00000025870	Arl10	20		ENSMUSG00000046178	Nxph1	19
ENSMUSG00000043157	Arl11	20		ENSMUSG00000069132	Nxph2	20
ENSMUSG00000022911	Arl13b	20		ENSMUSG00000046719	Nxph3	20
ENSMUSG00000027122	Arl14ep	1		ENSMUSG00000040258	Nxph4	1
ENSMUSG00000042348	Arl15	1		ENSMUSG00000036992	Nxt1	13
ENSMUSG00000057594	Arl16	20		ENSMUSG00000042271	Nxt2	18
ENSMUSG00000024944	Arl2	7		ENSMUSG00000045348	Nyap1	4
ENSMUSG00000031776	Arl2bp	18		ENSMUSG00000054976	Nyap2	18
ENSMUSG00000025035	Arl3	12		ENSMUSG00000075592	Nynrin	17
ENSMUSG00000047446	Arl4a	20		ENSMUSG00000051228	Nyx	20
ENSMUSG00000049866	Arl4c	18		ENSMUSG00000046610	Oacyl	9
ENSMUSG00000034936	Arl4d	20		ENSMUSG00000032014	Oaf	9
ENSMUSG00000036093	Arl5a	3		ENSMUSG00000029605	Oas1b	18
ENSMUSG00000017418	Arl5b	18		ENSMUSG00000001166	Oas1c	20
ENSMUSG00000038352	Arl5c	20		ENSMUSG00000032690	Oas2	20
ENSMUSG00000022722	Arl6	18		ENSMUSG00000029561	Oasl2	20
ENSMUSG00000030654	Arl6ip1	13		ENSMUSG00000030934	Oat	20
ENSMUSG00000029404	Arl6ip4	20		ENSMUSG00000035242	Oaz1	12
ENSMUSG00000035199	Arl6ip5	13		ENSMUSG00000043801	Oaz1-ps	12
ENSMUSG00000026960	Arl6ip6	20		ENSMUSG00000040652	Oaz2	10
ENSMUSG00000026426	Arl8a	9		ENSMUSG00000083610	Oaz2-ps	20
ENSMUSG00000030105	Arl8b	10		ENSMUSG00000093773	Obox3-ps7	1
ENSMUSG00000027599	Armc1	20		ENSMUSG00000061462	Obscn	20
ENSMUSG00000038525	Armc10	20		ENSMUSG00000026211	Obsl1	20
ENSMUSG00000071324	Armc2	13		ENSMUSG00000030450	Oca2	13
ENSMUSG00000037683	Armc3	20		ENSMUSG00000002396	Ocel1	18
ENSMUSG00000061802	Armc4	20		ENSMUSG00000029152	Ociad1	9
ENSMUSG00000042178	Armc5	20		ENSMUSG00000029153	Ociad2	9
ENSMUSG00000002343	Armc6	16		ENSMUSG00000021638	Ocln	20
ENSMUSG00000057219	Armc7	9		ENSMUSG00000001173	Ocrl	20
ENSMUSG00000032468	Armc8	20		ENSMUSG00000011179	Odc1	3

ENSMUSG00000062590	Armc9	3		ENSMUSG00000026790	Odf2	1
ENSMUSG00000033460	Armcx1	9		ENSMUSG00000028256	Odf2l	20
ENSMUSG00000033436	Armcx2	3		ENSMUSG00000047394	Odf3b	20
ENSMUSG00000049047	Armcx3	4		ENSMUSG00000032921	Odf4	1
ENSMUSG00000049804	Armcx4	9		ENSMUSG00000006010	Odr4	1
ENSMUSG00000072969	Armcx5	1		ENSMUSG00000040586	Ofd1	20
ENSMUSG00000050394	Armcx6	20		ENSMUSG00000025220	Oga	7
ENSMUSG00000060268	Armh1	20		ENSMUSG00000020456	Ogdh	12
ENSMUSG00000039901	Armh3	20		ENSMUSG00000021913	Ogdhl	20
ENSMUSG00000036242	Armh4	19		ENSMUSG00000033009	Ogfod1	20
ENSMUSG00000061759	Armt1	12		ENSMUSG00000023707	Ogfod2	20
ENSMUSG00000015522	Arnt	20		ENSMUSG00000025169	Ogfod3	20
ENSMUSG00000015709	Arnt2	9		ENSMUSG00000049401	Ogfr	20
ENSMUSG00000055116	Arntl	6		ENSMUSG00000026158	Ogfrl1	18
ENSMUSG00000040187	Arntl2	5		ENSMUSG00000030271	Ogg1	20
ENSMUSG00000029621	Arpc1a	12		ENSMUSG00000021390	Ogn	20
ENSMUSG00000029622	Arpc1b	20		ENSMUSG00000034160	Ogt	20
ENSMUSG00000006304	Arpc2	9		ENSMUSG00000072980	Oip5	20
ENSMUSG00000029465	Arpc3	12		ENSMUSG00000085438	Oip5os1	20
ENSMUSG00000079426	Arpc4	20		ENSMUSG00000027108	Ola1	9
ENSMUSG00000008475	Arpc5	4		ENSMUSG00000026833	Olfm1	5
ENSMUSG00000026755	Arpc5l	2		ENSMUSG00000032172	Olfm2	9
ENSMUSG00000039043	Arpin	20		ENSMUSG00000027965	Olfm3	9
ENSMUSG00000007656	Arpp19	9		ENSMUSG00000022026	Olfm4	20
ENSMUSG00000032503	Arpp21	9		ENSMUSG00000051041	Olfml1	20
ENSMUSG00000060890	Arr3	9		ENSMUSG00000046618	Olfml2a	20
ENSMUSG00000018909	Arrb1	1		ENSMUSG00000038463	Olfml2b	5
ENSMUSG00000060216	Arrb2	20		ENSMUSG00000027848	Olfml3	16
ENSMUSG00000026972	Arrdc1	9		ENSMUSG00000108218	Olfr1372-ps1	20
ENSMUSG00000074794	Arrdc3	20		ENSMUSG00000101750	Olfr1392	20
ENSMUSG00000042659	Arrdc4	20		ENSMUSG00000059864	Olfr1393	19
ENSMUSG00000022620	Arsa	18		ENSMUSG00000096169	Olfr1564	20
ENSMUSG00000042082	Arsb	1		ENSMUSG00000043385	Olfr267	20
ENSMUSG00000020604	Arsg	1		ENSMUSG00000090129	Olfr287	20
ENSMUSG00000046561	Arsj	19		ENSMUSG00000093942	Olfr46	5
ENSMUSG00000021592	Arsk	20		ENSMUSG00000070421	Olfr658	20
ENSMUSG00000034842	Art3	20		ENSMUSG00000050266	Olfr690	20
ENSMUSG00000028539	Artn	20		ENSMUSG00000043948	Olfr691	20
ENSMUSG00000031982	Arv1	20		ENSMUSG00000061039	Olfr920	20
ENSMUSG00000000325	Arvcf	20		ENSMUSG00000046160	Olig1	15
ENSMUSG00000035277	Arx	19		ENSMUSG00000039830	Olig2	20
ENSMUSG00000048355	Arxes1	1		ENSMUSG00000035069	Oma1	20
ENSMUSG00000048040	Arxes2	20		ENSMUSG00000049612	Omg	1
ENSMUSG00000003559	As3mt	20		ENSMUSG00000074006	Omp	20
ENSMUSG00000031591	Asah1	20		ENSMUSG00000043013	Onecut1	1
ENSMUSG00000024887	Asah2	20		ENSMUSG00000045991	Onecut2	2
ENSMUSG00000022377	Asap1	3		ENSMUSG00000038084	Opa1	7
ENSMUSG00000052632	Asap2	18		ENSMUSG00000052214	Opa3	20
ENSMUSG00000036995	Asap3	1		ENSMUSG00000050121	Opalin	16
ENSMUSG00000026311	Asb1	20		ENSMUSG00000062257	Opcml	5
ENSMUSG00000031382	Asb11	19		ENSMUSG00000031214	Ophn1	9
ENSMUSG00000033781	Asb13	18		ENSMUSG00000022562	Oplah	4
ENSMUSG00000034768	Asb16	9		ENSMUSG00000026525	Opn3	18
ENSMUSG00000067081	Asb18	20		ENSMUSG00000050511	Oprd1	9

ENSMUSG00000021200	Asb2	20		ENSMUSG00000025905	Oprk1	6
ENSMUSG00000020305	Asb3	9		ENSMUSG00000027584	Oprl1	9
ENSMUSG00000042607	Asb4	20		ENSMUSG00000000766	Oprm1	3
ENSMUSG00000031519	Asb5	20		ENSMUSG00000010311	Optc	20
ENSMUSG00000039483	Asb6	20		ENSMUSG00000026672	Optn	18
ENSMUSG00000030509	Asb7	20		ENSMUSG00000049686	Orai1	20
ENSMUSG00000048175	Asb8	20		ENSMUSG00000039747	Orai2	18
ENSMUSG00000044475	Ascc1	20		ENSMUSG00000043964	Orai3	18
ENSMUSG00000020412	Ascc2	20		ENSMUSG00000026037	Orc2	1
ENSMUSG00000038774	Ascc3	7		ENSMUSG00000040044	Orc3	20
ENSMUSG00000020052	Ascl1	20		ENSMUSG00000026761	Orc4	1
ENSMUSG00000019857	Asf1a	20		ENSMUSG00000029012	Orc5	20
ENSMUSG00000005470	Asf1b	20		ENSMUSG00000031697	Orc6	20
ENSMUSG00000028053	Ash1l	9		ENSMUSG00000026097	Ormdl1	20
ENSMUSG00000031575	Ash2l	20		ENSMUSG00000025353	Ormdl2	19
ENSMUSG00000023017	Asic1	9		ENSMUSG00000038150	Ormdl3	20
ENSMUSG00000020704	Asic2	18		ENSMUSG00000040462	Os9	20
ENSMUSG00000038276	Asic3	20		ENSMUSG00000024687	Osbp	20
ENSMUSG00000033007	Asic4	9		ENSMUSG00000020435	Osbp2	9
ENSMUSG00000025533	Asl	20		ENSMUSG00000040875	Osbpl10	20
ENSMUSG00000052456	Asna1	12		ENSMUSG00000022807	Osbpl11	20
ENSMUSG00000029752	Asns	9		ENSMUSG00000044252	Osbpl1a	18
ENSMUSG00000026095	Asnsd1	20		ENSMUSG00000039050	Osbpl2	18
ENSMUSG00000020774	Aspa	16		ENSMUSG00000029822	Osbpl3	19
ENSMUSG00000038704	Aspdh	20		ENSMUSG00000037606	Osbpl5	9
ENSMUSG00000037686	Aspg	20		ENSMUSG00000042359	Osbpl6	19
ENSMUSG00000028207	Asph	20		ENSMUSG00000038534	Osbpl7	16
ENSMUSG00000046378	Asphd1	20		ENSMUSG00000020189	Osbpl8	9
ENSMUSG00000029348	Asphd2	9		ENSMUSG00000028559	Osbpl9	20
ENSMUSG00000033952	Aspm	20		ENSMUSG00000054594	Oscar	20
ENSMUSG00000021388	Aspn	20		ENSMUSG00000042616	Oscp1	20
ENSMUSG00000033508	Asprv1	18		ENSMUSG00000035399	Oser1	9
ENSMUSG00000025142	Aspscr1	7		ENSMUSG00000006289	Osgep	3
ENSMUSG00000024654	Asrgl1	1		ENSMUSG00000026096	Osgepl1	20
ENSMUSG00000076441	Ass1	1		ENSMUSG00000074063	Osgin1	20
ENSMUSG00000032567	Aste1	20		ENSMUSG00000041153	Osgin2	20
ENSMUSG00000026587	Astn1	12		ENSMUSG00000022146	Osmr	20
ENSMUSG00000028373	Astn2	18		ENSMUSG00000038803	Ost4	20
ENSMUSG00000042548	Asxl1	20		ENSMUSG00000041084	Ostc	19
ENSMUSG00000037486	Asxl2	2		ENSMUSG00000024725	Ostf1	20
ENSMUSG00000045215	Asxl3	9		ENSMUSG00000038280	Ostm1	20
ENSMUSG00000013662	Atad1	20		ENSMUSG00000034990	Otoa	20
ENSMUSG00000052812	Atad2b	3		ENSMUSG00000062372	Otof	20
ENSMUSG00000029036	Atad3a	20		ENSMUSG00000091455	Otogl	18
ENSMUSG00000054514	Atad3aos	12		ENSMUSG00000021685	Otp	20
ENSMUSG00000017550	Atad5	20		ENSMUSG00000024767	Otub1	20
ENSMUSG00000024426	Atat1	20		ENSMUSG00000021203	Otub2	9
ENSMUSG00000034958	Atcay	20		ENSMUSG00000043415	Otud1	20
ENSMUSG00000085779	Atcayos	20		ENSMUSG00000041161	Otud3	20
ENSMUSG00000030850	Ate1	2		ENSMUSG00000036990	Otud4	9
ENSMUSG00000023027	Atf1	20		ENSMUSG00000031154	Otud5	20
ENSMUSG00000080968	Atf1-ps	20		ENSMUSG00000040550	Otud6b	20
ENSMUSG00000027104	Atf2	20		ENSMUSG00000033510	Otud7a	20
ENSMUSG00000026628	Atf3	20		ENSMUSG00000038495	Otud7b	15

ENSMUSG00000042406	Atf4	18		ENSMUSG00000046034	Otulin	20
ENSMUSG00000038539	Atf5	20		ENSMUSG00000056069	Otulinl	9
ENSMUSG00000026663	Atf6	9		ENSMUSG00000005917	Otx1	9
ENSMUSG00000015461	Atf6b	20		ENSMUSG00000021848	Otx2	13
ENSMUSG00000099083	Atf7	10		ENSMUSG00000098682	Otx2os1	13
ENSMUSG00000030213	Atf7ip	9		ENSMUSG00000038268	Ovca2	20
ENSMUSG00000021619	Atg10	12		ENSMUSG00000074340	Ovgp1	6
ENSMUSG00000037204	Atg101	20		ENSMUSG00000000959	Oxa1l	20
ENSMUSG00000032905	Atg12	20		ENSMUSG00000022186	Oxct1	1
ENSMUSG00000027244	Atg13	20		ENSMUSG00000044819	Oxgr1	20
ENSMUSG00000037526	Atg14	9		ENSMUSG00000039670	Oxld1	3
ENSMUSG00000026289	Atg16l1	20		ENSMUSG00000021906	Oxnad1	9
ENSMUSG00000047767	Atg16l2	20		ENSMUSG00000022307	Oxr1	18
ENSMUSG00000041341	Atg2b	3		ENSMUSG00000021786	Oxsm	20
ENSMUSG00000022663	Atg3	20		ENSMUSG00000036737	Oxsr1	20
ENSMUSG00000087119	Atg4a-ps	20		ENSMUSG00000049112	Oxtr	20
ENSMUSG00000026280	Atg4b	18		ENSMUSG00000027071	P2rx3	1
ENSMUSG00000028550	Atg4c	20		ENSMUSG00000029470	P2rx4	1
ENSMUSG00000002820	Atg4d	4		ENSMUSG00000005950	P2rx5	20
ENSMUSG00000038160	Atg5	20		ENSMUSG00000022758	P2rx6	20
ENSMUSG00000030314	Atg7	9		ENSMUSG00000029468	P2rx7	9
ENSMUSG00000033124	Atg9a	9		ENSMUSG00000027765	P2ry1	20
ENSMUSG00000038295	Atg9b	20		ENSMUSG00000054293	P2ry10b	20
ENSMUSG00000026192	Atic	20		ENSMUSG00000036353	P2ry12	11
ENSMUSG00000021066	Atl1	1		ENSMUSG00000036362	P2ry13	20
ENSMUSG00000059811	Atl2	8		ENSMUSG00000036381	P2ry14	20
ENSMUSG00000024759	Atl3	20		ENSMUSG00000032860	P2ry2	20
ENSMUSG00000034218	Atm	12		ENSMUSG00000048779	P2ry6	20
ENSMUSG00000047388	Atmin	20		ENSMUSG00000028641	P3h1	20
ENSMUSG00000004263	Atn1	20		ENSMUSG00000038168	P3h2	13
ENSMUSG00000036816	Atoh7	20		ENSMUSG00000023191	P3h3	2
ENSMUSG00000037621	Atoh8	20		ENSMUSG00000006931	P3h4	20
ENSMUSG00000018585	Atox1	12		ENSMUSG00000019916	P4ha1	9
ENSMUSG00000025324	Atp10a	20		ENSMUSG00000018906	P4ha2	9
ENSMUSG00000055415	Atp10b	15		ENSMUSG00000051048	P4ha3	20
ENSMUSG00000046808	Atp10d	20		ENSMUSG00000025130	P4hb	20
ENSMUSG00000031441	Atp11a	9		ENSMUSG00000006675	P4htm	12
ENSMUSG00000037400	Atp11b	20		ENSMUSG00000025364	Pa2g4	20
ENSMUSG00000062949	Atp11c	20		ENSMUSG00000022283	Pabpc1	9
ENSMUSG00000031862	Atp13a1	1		ENSMUSG00000054582	Pabpc1l	12
ENSMUSG00000036622	Atp13a2	3		ENSMUSG00000084842	Pabpc1l2b-ps	19
ENSMUSG00000022533	Atp13a3	20		ENSMUSG00000011257	Pabpc4	1
ENSMUSG00000038094	Atp13a4	20		ENSMUSG00000090919	Pabpc4l	20
ENSMUSG00000048939	Atp13a5	20		ENSMUSG00000034732	Pabpc5	20
ENSMUSG00000033161	Atp1a1	18		ENSMUSG00000022194	Pabpn1	9
ENSMUSG00000007097	Atp1a2	18		ENSMUSG00000026627	Pacc1	20
ENSMUSG00000040907	Atp1a3	18		ENSMUSG00000037196	Pacrg	10
ENSMUSG00000026576	Atp1b1	9		ENSMUSG00000024855	Pacs1	20
ENSMUSG00000041329	Atp1b2	18		ENSMUSG00000021143	Pacs2	16
ENSMUSG00000032412	Atp1b3	1		ENSMUSG00000040276	Pacsin1	20
ENSMUSG00000025436	Atp23	6		ENSMUSG00000016664	Pacsin2	16
ENSMUSG00000030730	Atp2a1	18		ENSMUSG00000027257	Pacsin3	20
ENSMUSG00000029467	Atp2a2	18		ENSMUSG00000028927	Padi2	16
ENSMUSG00000020788	Atp2a3	20		ENSMUSG00000003437	Paf1	20

ENSMUSG00000019943	Atp2b1	9		ENSMUSG00000020745	Pafah1b1	3
ENSMUSG00000030302	Atp2b2	8		ENSMUSG00000097041	Pafah1b1-ps1	20
ENSMUSG00000031376	Atp2b3	13		ENSMUSG00000003131	Pafah1b2	3
ENSMUSG00000026463	Atp2b4	20		ENSMUSG00000005447	Pafah1b3	20
ENSMUSG00000032570	Atp2c1	2		ENSMUSG00000037366	Pafah2	20
ENSMUSG00000025428	Atp5a1	20		ENSMUSG00000027508	Pag1	9
ENSMUSG00000025393	Atp5b	2		ENSMUSG00000029247	Paics	2
ENSMUSG00000025781	Atp5c1	12		ENSMUSG00000025451	Paip1	19
ENSMUSG00000114809	Atp5c1-ps	20		ENSMUSG00000037058	Paip2	20
ENSMUSG00000003072	Atp5d	20		ENSMUSG00000045896	Paip2b	1
ENSMUSG00000016252	Atp5e	12		ENSMUSG00000030774	Pak1	9
ENSMUSG00000000563	Atp5f1	20		ENSMUSG00000038683	Pak1ip1	20
ENSMUSG00000006057	Atp5g1	5		ENSMUSG00000022781	Pak2	20
ENSMUSG00000062683	Atp5g2	20		ENSMUSG00000031284	Pak3	1
ENSMUSG00000018770	Atp5g3	5		ENSMUSG00000030602	Pak4	20
ENSMUSG00000034566	Atp5h	12		ENSMUSG00000074923	Pak6	18
ENSMUSG00000022890	Atp5j	12		ENSMUSG00000039913	Pak7	20
ENSMUSG00000038690	Atp5j2	12		ENSMUSG00000038729	Pakap	18
ENSMUSG00000050856	Atp5k	10		ENSMUSG00000090053	Pakap	9
ENSMUSG00000081700	Atp5k-ps2	20		ENSMUSG00000089945	Pakap	20
ENSMUSG00000038717	Atp5l	19		ENSMUSG00000044702	Palb2	20
ENSMUSG00000084168	Atp5l2-ps	20		ENSMUSG00000020092	Pald1	20
ENSMUSG00000071528	Atp5md	12		ENSMUSG00000058056	Palld	18
ENSMUSG00000021290	Atp5mpl	12		ENSMUSG00000035863	Palm	9
ENSMUSG00000022956	Atp5o	12		ENSMUSG00000047986	Palm3	20
ENSMUSG00000019087	Atp6ap1	9		ENSMUSG00000033377	Palmd	20
ENSMUSG00000078958	Atp6ap1l	9		ENSMUSG00000026335	Pam	2
ENSMUSG00000031007	Atp6ap2	9		ENSMUSG00000014301	Pam16	12
ENSMUSG00000019302	Atp6v0a1	1		ENSMUSG00000027188	Pamr1	17
ENSMUSG00000038023	Atp6v0a2	20		ENSMUSG00000005682	Pan2	20
ENSMUSG00000033379	Atp6v0b	12		ENSMUSG00000029647	Pan3	20
ENSMUSG00000024121	Atp6v0c	10		ENSMUSG00000097814	Panct2	20
ENSMUSG00000080242	Atp6v0c-ps2	20		ENSMUSG00000033610	Pank1	18
ENSMUSG00000013160	Atp6v0d1	20		ENSMUSG00000037514	Pank2	20
ENSMUSG00000015575	Atp6v0e	20		ENSMUSG00000018846	Pank3	19
ENSMUSG00000039347	Atp6v0e2	20		ENSMUSG00000029056	Pank4	20
ENSMUSG00000052459	Atp6v1a	19		ENSMUSG00000060424	Pantr1	3
ENSMUSG00000006273	Atp6v1b2	3		ENSMUSG00000097063	Pantr2	20
ENSMUSG00000022295	Atp6v1c1	20		ENSMUSG00000031934	Panx1	18
ENSMUSG00000020566	Atp6v1c2	3		ENSMUSG00000058441	Panx2	18
ENSMUSG00000021114	Atp6v1d	19		ENSMUSG00000025464	Paox	12
ENSMUSG00000019210	Atp6v1e1	12		ENSMUSG00000021223	Papln	1
ENSMUSG00000004285	Atp6v1f	12		ENSMUSG00000021111	Papola	9
ENSMUSG00000039105	Atp6v1g1	12		ENSMUSG00000074817	Papolb	20
ENSMUSG00000024403	Atp6v1g2	20		ENSMUSG00000020273	Papolg	20
ENSMUSG00000033793	Atp6v1h	20		ENSMUSG00000028370	Pappa	20
ENSMUSG00000033792	Atp7a	20		ENSMUSG00000028032	Papss1	12
ENSMUSG00000006567	Atp7b	18		ENSMUSG00000024899	Papss2	9
ENSMUSG00000037685	Atp8a1	7		ENSMUSG00000055725	Paqr3	20
ENSMUSG00000021983	Atp8a2	18		ENSMUSG00000023909	Paqr4	20
ENSMUSG00000039529	Atp8b1	20		ENSMUSG00000032278	Paqr5	1
ENSMUSG00000060671	Atp8b2	20		ENSMUSG00000041423	Paqr6	20
ENSMUSG00000028457	Atp8b5	20		ENSMUSG00000037348	Paqr7	9
ENSMUSG00000027546	Atp9a	1		ENSMUSG00000025931	Paqr8	12

ENSMUSG00000024566	Atp9b	7		ENSMUSG00000064225	Paqr9	9
ENSMUSG00000028710	Atpaf1	18		ENSMUSG00000025812	Pard3	9
ENSMUSG00000042709	Atpaf2	20		ENSMUSG00000052062	Pard3b	20
ENSMUSG00000054428	Atpif1	12		ENSMUSG00000005699	Pard6a	20
ENSMUSG00000039065	Atpsckmt	20		ENSMUSG00000044641	Pard6b	9
ENSMUSG00000032409	Atr	2		ENSMUSG00000056214	Pard6g	20
ENSMUSG00000013622	Atraid	1		ENSMUSG00000021911	Parg	12
ENSMUSG00000025646	Atrip	20		ENSMUSG00000028964	Park7	12
ENSMUSG00000027312	Atrn	12		ENSMUSG00000033918	Parl	20
ENSMUSG00000054843	Atrnl1	18		ENSMUSG00000034981	Parm1	20
ENSMUSG00000031229	Atrx	2		ENSMUSG00000022685	Parn	20
ENSMUSG00000046876	Atxn1	7		ENSMUSG00000026496	Parp1	20
ENSMUSG00000016541	Atxn10	20		ENSMUSG00000063268	Parp10	20
ENSMUSG00000069895	Atxn1l	20		ENSMUSG00000037997	Parp11	20
ENSMUSG00000042605	Atxn2	3		ENSMUSG00000038507	Parp12	9
ENSMUSG00000032637	Atxn2l	20		ENSMUSG00000034422	Parp14	20
ENSMUSG00000021189	Atxn3	16		ENSMUSG00000032392	Parp16	19
ENSMUSG00000021738	Atxn7	20		ENSMUSG00000036023	Parp2	20
ENSMUSG00000020564	Atxn7l1	4		ENSMUSG00000023249	Parp3	20
ENSMUSG00000048997	Atxn7l2	20		ENSMUSG00000054509	Parp4	20
ENSMUSG00000059995	Atxn7l3	18		ENSMUSG00000025237	Parp6	9
ENSMUSG00000074748	Atxn7l3b	12		ENSMUSG00000021725	Parp8	9
ENSMUSG00000097415	AU020206	20		ENSMUSG00000022906	Parp9	20
ENSMUSG00000051669	AU021092	20		ENSMUSG00000035365	Parpbp	18
ENSMUSG00000078584	AU022252	12		ENSMUSG00000043572	Pars2	9
ENSMUSG00000073294	AU022751	20		ENSMUSG00000030770	Parva	18
ENSMUSG00000097209	AU022754	5		ENSMUSG00000022438	Parvb	6
ENSMUSG00000109890	AU023762	6		ENSMUSG00000022439	Parvg	20
ENSMUSG00000028830	AU040320	1		ENSMUSG00000026274	Pask	20
ENSMUSG00000091523	AU040972	20		ENSMUSG00000074452	Pate2	20
ENSMUSG00000078435	AU041133	20		ENSMUSG00000061859	Patj	18
ENSMUSG00000021460	Auh	20		ENSMUSG00000046139	Patl1	20
ENSMUSG00000068328	Aup1	20		ENSMUSG00000020453	Patz1	20
ENSMUSG00000027496	Aurka	20		ENSMUSG00000035873	Pawr	20
ENSMUSG00000065990	Aurkaip1	20		ENSMUSG00000004231	Pax2	1
ENSMUSG00000029673	Auts2	20		ENSMUSG00000027168	Pax6	9
ENSMUSG00000087519	AV039307	20		ENSMUSG00000022974	Paxbp1	20
ENSMUSG00000087377	AV099323	18		ENSMUSG00000002221	Paxip1	20
ENSMUSG00000108461	AV356131	20		ENSMUSG00000047617	Paxx	2
ENSMUSG00000003604	Aven	20		ENSMUSG00000031226	Pbdc1	20
ENSMUSG00000025432	Avil	19		ENSMUSG00000020072	Pbld2	20
ENSMUSG00000029787	Avl9	2		ENSMUSG00000042323	Pbrm1	20
ENSMUSG00000037727	Avp	20		ENSMUSG00000052534	Pbx1	16
ENSMUSG00000018821	Avpi1	1		ENSMUSG00000034673	Pbx2	20
ENSMUSG00000078349	AW011738	1		ENSMUSG00000038718	Pbx3	6
ENSMUSG00000110419	AW046200	20		ENSMUSG00000031860	Pbx4	3
ENSMUSG00000097428	AW047730	3		ENSMUSG00000042613	Pbxip1	20
ENSMUSG00000075010	AW112010	20		ENSMUSG00000020098	Pcbd1	9
ENSMUSG00000074166	AW146154	20		ENSMUSG00000021496	Pcbd2	10
ENSMUSG00000039182	AW209491	20		ENSMUSG00000051695	Pcbp1	12
ENSMUSG00000087334	AW495222	20		ENSMUSG00000056851	Pcbp2	9
ENSMUSG00000041935	AW549877	20		ENSMUSG00000001120	Pcbp3	20
ENSMUSG00000038112	AW551984	17		ENSMUSG00000023495	Pcbp4	9
ENSMUSG00000033632	AW554918	20		ENSMUSG00000041650	Pcca	20

ENSMUSG00000085998	AW822252	20		ENSMUSG00000032527	Pccb	20
ENSMUSG00000026601	Axdnd1	20		ENSMUSG00000051375	Pcdh1	5
ENSMUSG00000024182	Axin1	20		ENSMUSG00000049100	Pcdh10	1
ENSMUSG00000000142	Axin2	6		ENSMUSG00000034755	Pcdh11x	1
ENSMUSG00000002602	Axl	20		ENSMUSG00000052613	Pcdh15	9
ENSMUSG00000039285	Azi2	2		ENSMUSG00000035566	Pcdh17	2
ENSMUSG00000037458	Azin1	20		ENSMUSG00000037892	Pcdh18	9
ENSMUSG00000028789	Azin2	20		ENSMUSG00000051323	Pcdh19	9
ENSMUSG00000109995	B020031H02Rik	20		ENSMUSG00000050505	Pcdh20	5
ENSMUSG00000108042	B130021K23Rik	20		ENSMUSG00000029108	Pcdh7	20
ENSMUSG00000100005	B130024G19Rik	16		ENSMUSG00000036422	Pcdh8	5
ENSMUSG00000093606	B130034C11Rik	20		ENSMUSG00000055421	Pcdh9	19
ENSMUSG00000097414	B130046B21Rik	12		ENSMUSG00000103442	Pcdha1	20
ENSMUSG00000086502	B130055M24Rik	20		ENSMUSG00000007440	Pcdha11	18
ENSMUSG00000097547	B230110C06Rik	20		ENSMUSG00000102206	Pcdha11	12
ENSMUSG00000100166	B230110G15Rik	20		ENSMUSG00000103310	Pcdha12	20
ENSMUSG00000107729	B230112I24Rik	20		ENSMUSG00000104148	Pcdha2	12
ENSMUSG00000027165	B230118H07Rik	20		ENSMUSG00000102312	Pcdha3	20
ENSMUSG00000086844	B230206H07Rik	16		ENSMUSG00000104252	Pcdha4	20
ENSMUSG00000109574	B230206I08Rik	9		ENSMUSG00000103092	Pcdha5	20
ENSMUSG00000086003	B230206L02Rik	12		ENSMUSG00000103707	Pcdha6	2
ENSMUSG00000092492	B230208B08Rik	20		ENSMUSG00000104318	Pcdha7	20
ENSMUSG00000097378	B230208H11Rik	20		ENSMUSG00000103800	Pcdha8	20
ENSMUSG00000109006	B230209E15Rik	20		ENSMUSG00000103770	Pcdha9	20
ENSMUSG00000089706	B230216N24Rik	20		ENSMUSG00000103255	Pcdhac1	20
ENSMUSG00000050538	B230217C12Rik	19		ENSMUSG00000102697	Pcdhac2	20
ENSMUSG00000097785	B230217O12Rik	20		ENSMUSG00000051663	Pcdhb1	3
ENSMUSG00000045767	B230219D22Rik	20		ENSMUSG00000045657	Pcdhb10	1
ENSMUSG00000113701	B230303A05Rik	20		ENSMUSG00000051486	Pcdhb11	20
ENSMUSG00000106547	B230303O12Rik	14		ENSMUSG00000043458	Pcdhb12	1
ENSMUSG00000080717	B230307C23Rik	17		ENSMUSG00000047307	Pcdhb13	1
ENSMUSG00000109284	B230311B06Rik	20		ENSMUSG00000044043	Pcdhb14	1
ENSMUSG00000084843	B230312C02Rik	20		ENSMUSG00000047033	Pcdhb15	1
ENSMUSG00000045746	B230317F23Rik	20		ENSMUSG00000047910	Pcdhb16	2
ENSMUSG00000051844	B230319C09Rik	20		ENSMUSG00000046387	Pcdhb17	1
ENSMUSG00000097509	B230322F03Rik	20		ENSMUSG00000048347	Pcdhb18	1
ENSMUSG00000097623	B230323A14Rik	19		ENSMUSG00000043313	Pcdhb19	1
ENSMUSG00000104597	B230334C09Rik	7		ENSMUSG00000051599	Pcdhb2	1
ENSMUSG00000097119	B230354K17Rik	9		ENSMUSG00000046191	Pcdhb20	1
ENSMUSG00000115543	B230362B09Rik	3		ENSMUSG00000044022	Pcdhb21	1
ENSMUSG00000104195	B230377A18Rik	13		ENSMUSG00000073591	Pcdhb22	1
ENSMUSG00000087660	B230398E01Rik	20		ENSMUSG00000045498	Pcdhb3	1
ENSMUSG00000060802	B2m	20		ENSMUSG00000045689	Pcdhb4	1
ENSMUSG00000048406	B330016D10Rik	20		ENSMUSG00000063687	Pcdhb5	2
ENSMUSG00000043300	B3galnt1	20		ENSMUSG00000051678	Pcdhb6	20
ENSMUSG00000039242	B3galnt2	20		ENSMUSG00000045062	Pcdhb7	20
ENSMUSG00000034780	B3galt1	20		ENSMUSG00000045876	Pcdhb8	9
ENSMUSG00000033849	B3galt2	9		ENSMUSG00000051242	Pcdhb9	20
ENSMUSG00000067370	B3galt4	12		ENSMUSG00000103144	Pcdhga1	20
ENSMUSG00000074892	B3galt5	20		ENSMUSG00000102222	Pcdhga10	20
ENSMUSG00000050796	B3galt6	20		ENSMUSG00000102742	Pcdhga11	18
ENSMUSG00000045994	B3gat1	5		ENSMUSG00000102428	Pcdhga12	7
ENSMUSG00000026156	B3gat2	17		ENSMUSG00000103332	Pcdhga2	1
ENSMUSG00000071649	B3gat3	7		ENSMUSG00000104346	Pcdhga3	20

ENSMUSG00000051950	B3glct	19		ENSMUSG00000103677	Pcdhga4	6
ENSMUSG00000051650	B3gnt2	9		ENSMUSG00000103567	Pcdhga5	9
ENSMUSG00000031803	B3gnt3	20		ENSMUSG00000103793	Pcdhga6	9
ENSMUSG00000029431	B3gnt4	10		ENSMUSG00000103472	Pcdhga7	9
ENSMUSG00000022686	B3gnt5	20		ENSMUSG00000103897	Pcdhga8	19
ENSMUSG00000074004	B3gnt6	20		ENSMUSG00000102440	Pcdhga9	6
ENSMUSG00000079445	B3gnt7	20		ENSMUSG00000103037	Pcdhgb1	20
ENSMUSG00000059479	B3gnt8	20		ENSMUSG00000102748	Pcdhgb2	20
ENSMUSG00000069920	B3gnt9	20		ENSMUSG00000103585	Pcdhgb4	20
ENSMUSG00000046605	B3gntl1	1		ENSMUSG00000103749	Pcdhgb5	20
ENSMUSG00000067356	B430203G13Rik	20		ENSMUSG00000103088	Pcdhgb6	20
ENSMUSG00000053706	B430305J03Rik	20		ENSMUSG00000104063	Pcdhgb7	9
ENSMUSG00000110062	B430319F04Rik	20		ENSMUSG00000103081	Pcdhgb8	20
ENSMUSG00000006731	B4galnt1	1		ENSMUSG00000102918	Pcdhgc3	20
ENSMUSG00000055629	B4galnt4	9		ENSMUSG00000023036	Pcdhgc4	20
ENSMUSG00000028413	B4galt1	20		ENSMUSG00000102543	Pcdhgc5	18
ENSMUSG00000028541	B4galt2	9		ENSMUSG00000037773	Pced1a	20
ENSMUSG00000052423	B4galt3	20		ENSMUSG00000044250	Pced1b	20
ENSMUSG00000022793	B4galt4	20		ENSMUSG00000041328	Pcf11	2
ENSMUSG00000017929	B4galt5	19		ENSMUSG00000069678	Pcgf1	20
ENSMUSG00000056124	B4galt6	16		ENSMUSG00000018537	Pcgf2	20
ENSMUSG00000021504	B4galt7	20		ENSMUSG00000033623	Pcgf3	2
ENSMUSG00000047379	B4gat1	20		ENSMUSG00000024805	Pcgf5	20
ENSMUSG00000052364	B630019K06Rik	17		ENSMUSG00000025050	Pcgf6	20
ENSMUSG00000098202	B830012L14Rik	3		ENSMUSG00000038542	Pcid2	20
ENSMUSG00000116652	B830017H08Rik	20		ENSMUSG00000039849	Pcif1	20
ENSMUSG00000108847	B830042I05Rik	20		ENSMUSG00000040618	Pck2	20
ENSMUSG00000113361	B930059L03Rik	10		ENSMUSG00000061601	Pclo	3
ENSMUSG00000110282	B930086L07Rik	20		ENSMUSG00000031592	Pcm1	2
ENSMUSG00000085133	B930095G15Rik	20		ENSMUSG00000051285	Pcmtd1	9
ENSMUSG00000001039	B9d1	20		ENSMUSG00000027589	Pcmtd2	9
ENSMUSG00000060808	B9d1os	20		ENSMUSG00000027342	Pcna	20
ENSMUSG00000063439	B9d2	20		ENSMUSG00000067608	Pcna-ps2	20
ENSMUSG00000031820	Babam1	7		ENSMUSG00000071533	Pcnp	9
ENSMUSG00000052139	Babam2	20		ENSMUSG00000001151	Pcnt	20
ENSMUSG00000032086	Bace1	20		ENSMUSG00000021140	Pcnx	2
ENSMUSG00000040605	Bace2	9		ENSMUSG00000060212	Pcnx2	20
ENSMUSG00000025612	Bach1	20		ENSMUSG00000054874	Pcnx3	20
ENSMUSG00000040270	Bach2	11		ENSMUSG00000034501	Pcnx4	20
ENSMUSG00000087049	Bach2it1	20		ENSMUSG00000029718	Pcolce	13
ENSMUSG00000024959	Bad	13		ENSMUSG00000015354	Pcolce2	20
ENSMUSG00000028416	Bag1	20		ENSMUSG00000090223	Pcp4	1
ENSMUSG00000042215	Bag2	20		ENSMUSG00000038370	Pcp4l1	1
ENSMUSG00000030847	Bag3	20		ENSMUSG00000021587	Pcsk1	1
ENSMUSG00000037316	Bag4	20		ENSMUSG00000039278	Pcsk1n	20
ENSMUSG00000049792	Bag5	3		ENSMUSG00000027419	Pcsk2	20
ENSMUSG00000024392	Bag6	20		ENSMUSG00000086171	Pcsk2os1	20
ENSMUSG00000039741	Bahcc1	20		ENSMUSG00000020131	Pcsk4	20
ENSMUSG00000040007	Bahd1	18		ENSMUSG00000024713	Pcsk5	19
ENSMUSG00000025372	Baiap2	9		ENSMUSG00000030513	Pcsk6	16
ENSMUSG00000038859	Baiap2l1	13		ENSMUSG00000035382	Pcsk7	12
ENSMUSG00000018126	Baiap2l2	20		ENSMUSG00000044254	Pcsk9	20
ENSMUSG00000047507	Baiap3	17		ENSMUSG00000020553	Pctp	20
ENSMUSG00000057789	Bak1	20		ENSMUSG00000024892	Pcx	18

ENSMUSG00000024232	Bambi	20		ENSMUSG00000029998	Pcyox1	12
ENSMUSG00000081219	Bambi-ps1	1		ENSMUSG00000024579	Pcyox1l	20
ENSMUSG00000024844	Banf1	19		ENSMUSG00000005615	Pcyt1a	20
ENSMUSG00000037922	Bank1	9		ENSMUSG00000035246	Pcyt1b	1
ENSMUSG00000025316	Banp	20		ENSMUSG00000025137	Pcyt2	20
ENSMUSG00000021901	Bap1	9		ENSMUSG00000029623	Pdap1	20
ENSMUSG00000026196	Bard1	18		ENSMUSG00000027835	Pdcd10	20
ENSMUSG00000021381	Barx1	1		ENSMUSG00000025047	Pdcd11	20
ENSMUSG00000032033	Barx2	20		ENSMUSG00000014771	Pdcd2	20
ENSMUSG00000045763	Basp1	19		ENSMUSG00000002635	Pdcd2l	20
ENSMUSG00000003873	Bax	20		ENSMUSG00000024975	Pdcd4	20
ENSMUSG00000035021	Baz1a	9		ENSMUSG00000030417	Pdcd5	10
ENSMUSG00000002748	Baz1b	12		ENSMUSG00000102824	Pdcd5-ps	20
ENSMUSG00000040054	Baz2a	1		ENSMUSG00000021576	Pdcd6	6
ENSMUSG00000026987	Baz2b	1		ENSMUSG00000032504	Pdcd6ip	20
ENSMUSG00000085041	BB031773	19		ENSMUSG00000041837	Pdcd7	18
ENSMUSG00000105389	BB187690	9		ENSMUSG00000009030	Pdcl	20
ENSMUSG00000085218	BB218582	9		ENSMUSG00000026078	Pdcl3	20
ENSMUSG00000002083	Bbc3	9		ENSMUSG00000023868	Pde10a	9
ENSMUSG00000084957	Bbip1	12		ENSMUSG00000075270	Pde11a	20
ENSMUSG00000057265	Bbof1	20		ENSMUSG00000059173	Pde1a	20
ENSMUSG00000041660	Bbox1	9		ENSMUSG00000022489	Pde1b	9
ENSMUSG00000006464	Bbs1	2		ENSMUSG00000004347	Pde1c	7
ENSMUSG00000035759	Bbs10	20		ENSMUSG00000110195	Pde2a	9
ENSMUSG00000051444	Bbs12	20		ENSMUSG00000041741	Pde3a	20
ENSMUSG00000031755	Bbs2	1		ENSMUSG00000030671	Pde3b	20
ENSMUSG00000025235	Bbs4	20		ENSMUSG00000032177	Pde4a	20
ENSMUSG00000063145	Bbs5	20		ENSMUSG00000028525	Pde4b	9
ENSMUSG00000037325	Bbs7	18		ENSMUSG00000021699	Pde4d	3
ENSMUSG00000035919	Bbs9	1		ENSMUSG00000038170	Pde4dip	7
ENSMUSG00000022641	Bbx	6		ENSMUSG00000053965	Pde5a	6
ENSMUSG00000060149	BC002059	20		ENSMUSG00000029491	Pde6b	20
ENSMUSG00000081824	BC002163	12		ENSMUSG00000026239	Pde6d	20
ENSMUSG00000067722	BC003965	20		ENSMUSG00000064330	Pde6h	20
ENSMUSG00000052712	BC004004	20		ENSMUSG00000069094	Pde7a	20
ENSMUSG00000019132	BC005537	2		ENSMUSG00000019990	Pde7b	9
ENSMUSG00000079065	BC005561	2		ENSMUSG00000025584	Pde8a	16
ENSMUSG00000026851	BC005624	20		ENSMUSG00000021684	Pde8b	9
ENSMUSG00000041674	BC006965	20		ENSMUSG00000041119	Pde9a	20
ENSMUSG00000033187	BC016579	20		ENSMUSG00000078931	Pdf	20
ENSMUSG00000030780	BC017158	7		ENSMUSG00000025856	Pdgfa	20
ENSMUSG00000056032	BC018473	20		ENSMUSG00000000489	Pdgfb	20
ENSMUSG00000081137	BC022960	7		ENSMUSG00000028019	Pdgfc	1
ENSMUSG00000092365	BC023719	11		ENSMUSG00000032006	Pdgfd	13
ENSMUSG00000112160	BC024063	20		ENSMUSG00000029231	Pdgfra	20
ENSMUSG00000044361	BC024139	7		ENSMUSG00000024620	Pdgfrb	20
ENSMUSG00000078786	BC024978	20		ENSMUSG00000031595	Pdgfrl	20
ENSMUSG00000074862	BC025920	9		ENSMUSG00000031299	Pdha1	9
ENSMUSG00000118004	BC026513	20		ENSMUSG00000021748	Pdhb	2
ENSMUSG00000038543	BC028528	20		ENSMUSG00000010914	Pdhx	9
ENSMUSG00000074649	BC029722	12		ENSMUSG00000027248	Pdia3	20
ENSMUSG00000105345	BC030343	20		ENSMUSG00000025823	Pdia4	20
ENSMUSG00000049946	BC030500	9		ENSMUSG00000022844	Pdia5	20
ENSMUSG00000034773	BC030867	18		ENSMUSG00000020571	Pdia6	20

ENSMUSG00000036299	BC031181	12		ENSMUSG00000050890	Pdik1l	20
ENSMUSG00000033722	BC034090	9		ENSMUSG00000006494	Pdk1	9
ENSMUSG00000090164	BC035044	12		ENSMUSG00000038967	Pdk2	2
ENSMUSG00000090486	BC035947	18		ENSMUSG00000035232	Pdk3	20
ENSMUSG00000087221	BC037032	20		ENSMUSG00000019577	Pdk4	20
ENSMUSG00000103309	BC037039	20		ENSMUSG00000055044	Pdlim1	19
ENSMUSG00000117698	BC037704	20		ENSMUSG00000022090	Pdlim2	3
ENSMUSG00000086272	BC039966	20		ENSMUSG00000031636	Pdlim3	20
ENSMUSG00000056418	BC043934	20		ENSMUSG00000020388	Pdlim4	20
ENSMUSG00000086662	BC046251	9		ENSMUSG00000028273	Pdlim5	9
ENSMUSG00000053684	BC048403	20		ENSMUSG00000021493	Pdlim7	19
ENSMUSG00000091996	BC049352	1		ENSMUSG00000049225	Pdp1	20
ENSMUSG00000047515	BC049715	20		ENSMUSG00000048371	Pdp2	12
ENSMUSG00000057246	BC051142	1		ENSMUSG00000024122	Pdpk1	9
ENSMUSG00000092564	BC051226	20		ENSMUSG00000033624	Pdpr	12
ENSMUSG00000117478	BC051408	20		ENSMUSG00000027472	Pdrg1	12
ENSMUSG00000040282	BC052040	20		ENSMUSG00000029202	Pds5a	11
ENSMUSG00000103973	BC055308	20		ENSMUSG00000034021	Pds5b	4
ENSMUSG00000041406	BC055324	20		ENSMUSG00000026784	Pdss1	20
ENSMUSG00000101429	BC055402	12		ENSMUSG00000038240	Pdss2	20
ENSMUSG00000108737	BC060293	20		ENSMUSG00000022680	Pdxdc1	20
ENSMUSG00000087150	BC064078	20		ENSMUSG00000032788	Pdxk	1
ENSMUSG00000087368	BC065397	20		ENSMUSG00000116165	Pdxp	20
ENSMUSG00000021763	BC067074	20		ENSMUSG00000027400	Pdyn	3
ENSMUSG00000102478	BC085271	20		ENSMUSG00000015668	Pdzd11	20
ENSMUSG00000115783	Bc1	10		ENSMUSG00000022197	Pdzd2	9
ENSMUSG00000045231	BC106179	18		ENSMUSG00000032105	Pdzd3	20
ENSMUSG00000002980	Bcam	13		ENSMUSG00000002006	Pdzd4	9
ENSMUSG00000004892	Bcan	1		ENSMUSG00000074818	Pdzd7	20
ENSMUSG00000020650	Bcap29	20		ENSMUSG00000074746	Pdzd8	12
ENSMUSG00000002015	Bcap31	9		ENSMUSG00000030887	Pdzd9	1
ENSMUSG00000031955	Bcar1	20		ENSMUSG00000024227	Pdzph1	20
ENSMUSG00000028121	Bcar3	20		ENSMUSG00000035357	Pdzrn3	18
ENSMUSG00000013523	Bcas1	16		ENSMUSG00000036218	Pdzrn4	9
ENSMUSG00000086970	Bcas1os1	20		ENSMUSG00000013698	Pea15a	20
ENSMUSG00000086999	Bcas1os2	20		ENSMUSG00000074305	Peak1	19
ENSMUSG00000005687	Bcas2	20		ENSMUSG00000097111	Peak1os	20
ENSMUSG00000059439	Bcas3	20		ENSMUSG00000028073	Pear1	20
ENSMUSG00000030268	Bcat1	18		ENSMUSG00000032959	Pebp1	12
ENSMUSG00000030826	Bcat2	20		ENSMUSG00000020717	Pecam1	20
ENSMUSG00000030983	Bccip	20		ENSMUSG00000026189	Pecr	20
ENSMUSG00000037525	Bcdin3d	20		ENSMUSG00000028779	Pef1	19
ENSMUSG00000027792	Bche	20		ENSMUSG00000092035	Peg10	17
ENSMUSG00000060376	Bckdha	3		ENSMUSG00000106847	Peg13	18
ENSMUSG00000032263	Bckdhb	20		ENSMUSG00000002265	Peg3	18
ENSMUSG00000030802	Bckdk	9		ENSMUSG00000020134	Peli1	19
ENSMUSG00000028191	Bcl10	20		ENSMUSG00000021846	Peli2	1
ENSMUSG00000000861	Bcl11a	18		ENSMUSG00000024901	Peli3	20
ENSMUSG00000048251	Bcl11b	9		ENSMUSG00000042275	Pelo	20
ENSMUSG00000057329	Bcl2	20		ENSMUSG00000018921	Pelp1	20
ENSMUSG00000007659	Bcl2l1	20		ENSMUSG00000000301	Pemt	20
ENSMUSG00000027381	Bcl2l11	9		ENSMUSG00000045573	Penk	9
ENSMUSG00000003190	Bcl2l12	20		ENSMUSG00000063931	Pepd	9
ENSMUSG00000009112	Bcl2l13	12		ENSMUSG00000020893	Per1	16

ENSMUSG00000044165	Bcl2l15	9		ENSMUSG00000055866	Per2	9
ENSMUSG00000089682	Bcl2l2	20		ENSMUSG00000028957	Per3	1
ENSMUSG00000053175	Bcl3	20		ENSMUSG00000078486	Perm1	20
ENSMUSG00000022508	Bcl6	20		ENSMUSG00000019851	Perp	13
ENSMUSG00000000317	Bcl6b	20		ENSMUSG00000020430	Pes1	3
ENSMUSG00000029438	Bcl7a	20		ENSMUSG00000087687	Pet100	12
ENSMUSG00000029681	Bcl7b	20		ENSMUSG00000005907	Pex1	20
ENSMUSG00000030814	Bcl7c	9		ENSMUSG00000029047	Pex10	20
ENSMUSG00000038256	Bcl9	20		ENSMUSG00000030545	Pex11a	20
ENSMUSG00000063382	Bcl9l	9		ENSMUSG00000028102	Pex11b	20
ENSMUSG00000037608	Bclaf1	20		ENSMUSG00000069633	Pex11g	20
ENSMUSG00000044150	Bclaf3	20		ENSMUSG00000018733	Pex12	20
ENSMUSG00000032066	Bco2	18		ENSMUSG00000020283	Pex13	20
ENSMUSG00000040363	Bcor	20		ENSMUSG00000028975	Pex14	20
ENSMUSG00000036959	Bcorl1	20		ENSMUSG00000027222	Pex16	20
ENSMUSG00000009681	Bcr	9		ENSMUSG00000003464	Pex19	20
ENSMUSG00000026172	Bcs1l	20		ENSMUSG00000040374	Pex2	20
ENSMUSG00000046598	Bdh1	18		ENSMUSG00000067825	Pex26	20
ENSMUSG00000028167	Bdh2	20		ENSMUSG00000019809	Pex3	20
ENSMUSG00000021070	Bdkrb2	20		ENSMUSG00000005069	Pex5	20
ENSMUSG00000048482	Bdnf	5		ENSMUSG00000027674	Pex5l	16
ENSMUSG00000049658	Bdp1	20		ENSMUSG00000002763	Pex6	20
ENSMUSG00000031872	Bean1	20		ENSMUSG00000020003	Pex7	20
ENSMUSG00000035086	Becn1	20		ENSMUSG00000020899	Pfas	1
ENSMUSG00000040867	Begain	5		ENSMUSG00000024346	Pfdn1	12
ENSMUSG00000038214	Bend3	20		ENSMUSG00000006412	Pfdn2	10
ENSMUSG00000092060	Bend4	1		ENSMUSG00000052033	Pfdn4	20
ENSMUSG00000028545	Bend5	2		ENSMUSG00000001289	Pfdn5	12
ENSMUSG00000042182	Bend6	18		ENSMUSG00000024309	Pfdn6	12
ENSMUSG00000048186	Bend7	20		ENSMUSG00000025271	Pfkfb1	9
ENSMUSG00000037418	Best1	20		ENSMUSG00000026409	Pfkfb2	20
ENSMUSG00000020169	Best3	19		ENSMUSG00000026773	Pfkfb3	5
ENSMUSG00000032757	Bet1	20		ENSMUSG00000025648	Pfkfb4	20
ENSMUSG00000025484	Bet1l	13		ENSMUSG00000020277	Pfkl	1
ENSMUSG00000050071	Bex1	12		ENSMUSG00000033065	Pfkm	18
ENSMUSG00000042750	Bex2	16		ENSMUSG00000021196	Pfkp	20
ENSMUSG00000046432	Bex3	19		ENSMUSG00000018293	Pfn1	20
ENSMUSG00000047844	Bex4	20		ENSMUSG00000027805	Pfn2	16
ENSMUSG00000022684	Bfar	20		ENSMUSG00000020639	Pfn4	20
ENSMUSG00000027420	Bfsp1	20		ENSMUSG00000011752	Pgam1	9
ENSMUSG00000032556	Bfsp2	20		ENSMUSG00000082016	Pgam1-ps2	20
ENSMUSG00000031375	Bgn	13		ENSMUSG00000020475	Pgam2	3
ENSMUSG00000072964	Bhlhb9	9		ENSMUSG00000029500	Pgam5	20
ENSMUSG00000025128	Bhlhe22	5		ENSMUSG00000073678	Pgap1	20
ENSMUSG00000030103	Bhlhe40	1		ENSMUSG00000030990	Pgap2	20
ENSMUSG00000030256	Bhlhe41	1		ENSMUSG00000038208	Pgap3	20
ENSMUSG00000014329	Bicc1	20		ENSMUSG00000055313	Pgbd1	20
ENSMUSG00000003452	Bicd1	3		ENSMUSG00000050751	Pgbd5	9
ENSMUSG00000037933	Bicd2	20		ENSMUSG00000028961	Pgd	20
ENSMUSG00000041609	Bicdl1	20		ENSMUSG00000004791	Pgf	20
ENSMUSG00000070808	Bicra	20		ENSMUSG00000062031	Pgghg	20
ENSMUSG00000036568	Bicral	20		ENSMUSG00000024477	Pggt1b	2
ENSMUSG00000004446	Bid	9		ENSMUSG00000062070	Pgk1	9
ENSMUSG00000024381	Bin1	20		ENSMUSG00000066632	Pgk1-rs7	20

ENSMUSG00000098112	Bin2	20		ENSMUSG00000031807	Pgls	13
ENSMUSG00000022089	Bin3	20		ENSMUSG00000030413	Pglyrp1	20
ENSMUSG00000057367	Birc2	20		ENSMUSG00000029171	Pgm2	20
ENSMUSG00000032000	Birc3	20		ENSMUSG00000030729	Pgm2l1	5
ENSMUSG00000017716	Birc5	20		ENSMUSG00000056131	Pgm3	20
ENSMUSG00000024073	Birc6	3		ENSMUSG00000041731	Pgm5	20
ENSMUSG00000041684	Bivm	3		ENSMUSG00000043445	Pgp	20
ENSMUSG00000067787	Blcap	20		ENSMUSG00000056204	Pgpep1	20
ENSMUSG00000030528	Blm	20		ENSMUSG00000030553	Pgpep1l	20
ENSMUSG00000020840	Blmh	20		ENSMUSG00000031870	Pgr	12
ENSMUSG00000061132	Blnk	9		ENSMUSG00000006373	Pgrmc1	18
ENSMUSG00000090247	Bloc1s1	12		ENSMUSG00000049940	Pgrmc2	1
ENSMUSG00000057506	Bloc1s2	12		ENSMUSG00000017715	Pgs1	12
ENSMUSG00000025201	Bloc1s2-ps	20		ENSMUSG00000054728	Phactr1	9
ENSMUSG00000057667	Bloc1s3	20		ENSMUSG00000062866	Phactr2	18
ENSMUSG00000060708	Bloc1s4	20		ENSMUSG00000027525	Phactr3	9
ENSMUSG00000038982	Bloc1s5	20		ENSMUSG00000066043	Phactr4	15
ENSMUSG00000005804	Bloc1s6	20		ENSMUSG00000008301	Phax	20
ENSMUSG00000001999	Blvra	20		ENSMUSG00000038845	Phb	20
ENSMUSG00000040466	Blvrb	20		ENSMUSG00000004264	Phb2	20
ENSMUSG00000026577	Blzf1	2		ENSMUSG00000040669	Phc1	20
ENSMUSG00000044117	Bmerb1	9		ENSMUSG00000028796	Phc2	9
ENSMUSG00000040093	Bmf	20		ENSMUSG00000037652	Phc3	2
ENSMUSG00000026739	Bmi1	1		ENSMUSG00000044134	Pheta1	20
ENSMUSG00000022098	Bmp1	20		ENSMUSG00000057457	Phex	1
ENSMUSG00000027358	Bmp2	1		ENSMUSG00000024193	Phf1	20
ENSMUSG00000034663	Bmp2k	15		ENSMUSG00000023883	Phf10	20
ENSMUSG00000029335	Bmp3	20		ENSMUSG00000091144	Phf11c	20
ENSMUSG00000021835	Bmp4	20		ENSMUSG00000068245	Phf11d	20
ENSMUSG00000032179	Bmp5	9		ENSMUSG00000037791	Phf12	9
ENSMUSG00000039004	Bmp6	13		ENSMUSG00000047777	Phf13	20
ENSMUSG00000008999	Bmp7	13		ENSMUSG00000029629	Phf14	2
ENSMUSG00000031963	Bmper	16		ENSMUSG00000026873	Phf19	20
ENSMUSG00000021796	Bmpr1a	20		ENSMUSG00000038025	Phf2	1
ENSMUSG00000052430	Bmpr1b	20		ENSMUSG00000038116	Phf20	5
ENSMUSG00000067336	Bmpr2	2		ENSMUSG00000098506	Phf20-ps	20
ENSMUSG00000030138	Bms1	20		ENSMUSG00000072501	Phf20l1	2
ENSMUSG00000042742	Bmt2	18		ENSMUSG00000058318	Phf21a	18
ENSMUSG00000031377	Bmx	9		ENSMUSG00000016624	Phf21b	20
ENSMUSG00000049086	Bmyc	12		ENSMUSG00000018572	Phf23	20
ENSMUSG00000028487	Bnc2	9		ENSMUSG00000036062	Phf24	9
ENSMUSG00000024191	Bnip1	20		ENSMUSG00000086889	Phf2os1	1
ENSMUSG00000011958	Bnip2	18		ENSMUSG00000048874	Phf3	2
ENSMUSG00000078566	Bnip3	1		ENSMUSG00000061360	Phf5a	3
ENSMUSG00000022051	Bnip3l	20		ENSMUSG00000025626	Phf6	20
ENSMUSG00000073198	Bnip3l-ps	20		ENSMUSG00000021902	Phf7	20
ENSMUSG00000022687	Boc	20		ENSMUSG00000041229	Phf8	2
ENSMUSG00000044502	Bod1	20		ENSMUSG00000023350	Phf8-ps	20
ENSMUSG00000061755	Bod1l	20		ENSMUSG00000053398	Phgdh	3
ENSMUSG00000026278	Bok	19		ENSMUSG00000032253	Phip	2
ENSMUSG00000015943	Bola1	20		ENSMUSG00000034055	Phka1	20
ENSMUSG00000047721	Bola2	12		ENSMUSG00000031295	Phka2	3
ENSMUSG00000045160	Bola3	12		ENSMUSG00000036879	Phkb	1
ENSMUSG00000022557	Bop1	20		ENSMUSG00000025537	Phkg1	12

ENSMUSG00000022070	Bora	1		ENSMUSG00000030815	Phkg2	12
ENSMUSG00000042992	Borcs5	20		ENSMUSG00000020205	Phlda1	18
ENSMUSG00000045176	Borcs6	20		ENSMUSG00000041801	Phlda3	20
ENSMUSG00000062376	Borcs7	10		ENSMUSG00000048537	Phldb1	16
ENSMUSG00000002345	Borcs8	20		ENSMUSG00000033149	Phldb2	13
ENSMUSG00000038871	Bpgm	20		ENSMUSG00000044340	Phlpp1	16
ENSMUSG00000038286	Bphl	20		ENSMUSG00000031732	Phlpp2	2
ENSMUSG00000026617	Bpnt1	12		ENSMUSG00000050860	Phospho1	20
ENSMUSG00000040481	Bptf	2		ENSMUSG00000027088	Phospho2	20
ENSMUSG00000002413	Braf	2		ENSMUSG00000036504	Phpt1	12
ENSMUSG00000029458	Brap	20		ENSMUSG00000038611	Phrf1	6
ENSMUSG00000000148	Brat1	20		ENSMUSG00000058388	Phtf1	1
ENSMUSG00000017146	Brca1	20		ENSMUSG00000089998	Phtf1os	20
ENSMUSG00000041147	Brca2	20		ENSMUSG00000039987	Phtf2	20
ENSMUSG00000031201	Brcc3	9		ENSMUSG00000031802	Phxr4	5
ENSMUSG00000022387	Brd1	10		ENSMUSG00000026664	Phyh	9
ENSMUSG00000024335	Brd2	5		ENSMUSG00000079484	Phyhd1	20
ENSMUSG00000026918	Brd3	20		ENSMUSG00000003469	Phyhip	9
ENSMUSG00000109946	Brd3os	3		ENSMUSG00000037747	Phyhipl	3
ENSMUSG00000024002	Brd4	20		ENSMUSG00000020359	Phykpl	12
ENSMUSG00000031660	Brd7	20		ENSMUSG00000067780	Pi15	20
ENSMUSG00000003778	Brd8	20		ENSMUSG00000024011	Pi16	20
ENSMUSG00000057649	Brd9	5		ENSMUSG00000025178	Pi4k2a	18
ENSMUSG00000029279	Brdt	20		ENSMUSG00000029186	Pi4k2b	20
ENSMUSG00000011158	Brf1	20		ENSMUSG00000041720	Pi4ka	3
ENSMUSG00000031487	Brf2	20		ENSMUSG00000038861	Pi4kb	20
ENSMUSG00000047843	Bri3	20		ENSMUSG00000030329	Pianp	5
ENSMUSG00000037905	Bri3bp	9		ENSMUSG00000032405	Pias1	9
ENSMUSG00000045744	Bricd5	20		ENSMUSG00000025423	Pias2	12
ENSMUSG00000028351	Brinp1	9		ENSMUSG00000028101	Pias3	9
ENSMUSG00000004031	Brinp2	20		ENSMUSG00000004934	Pias4	19
ENSMUSG00000035131	Brinp3	9		ENSMUSG00000022064	Pibf1	20
ENSMUSG00000085208	Brip1os	20		ENSMUSG00000039361	Picalm	20
ENSMUSG00000022247	Brix1	20		ENSMUSG00000068206	Pick1	20
ENSMUSG00000033940	Brk1	3		ENSMUSG00000045658	Pid1	3
ENSMUSG00000080268	Brms1	20		ENSMUSG00000025507	Pidd1	20
ENSMUSG00000012076	Brms1l	20		ENSMUSG00000014444	Piezo1	13
ENSMUSG00000046836	Brox	20		ENSMUSG00000041482	Piezo2	20
ENSMUSG00000001632	Brpf1	20		ENSMUSG00000041064	Pif1	20
ENSMUSG00000063952	Brpf3	18		ENSMUSG00000010136	Pifo	20
ENSMUSG00000035390	Brsk1	20		ENSMUSG00000031381	Piga	16
ENSMUSG00000053046	Brsk2	18		ENSMUSG00000079469	Pigb	20
ENSMUSG00000022914	Brwd1	7		ENSMUSG00000026698	Pigc	1
ENSMUSG00000063663	Brwd3	20		ENSMUSG00000024145	Pigf	20
ENSMUSG00000071657	Bscl2	9		ENSMUSG00000029263	Pigg	20
ENSMUSG00000040859	Bsdc1	12		ENSMUSG00000021120	Pigh	20
ENSMUSG00000023175	Bsg	3		ENSMUSG00000039047	Pigk	20
ENSMUSG00000032589	Bsn	20		ENSMUSG00000014245	Pigl	20
ENSMUSG00000074378	Bsph1	11		ENSMUSG00000050229	Pigm	20
ENSMUSG00000028392	Bspry	20		ENSMUSG00000056536	Pign	20
ENSMUSG00000046718	Bst2	12		ENSMUSG00000028454	Pigo	20
ENSMUSG00000040565	Btaf1	7		ENSMUSG00000022940	Pigp	19
ENSMUSG00000025103	Btbd1	20		ENSMUSG00000025728	Pigq	18
ENSMUSG00000038187	Btbd10	20		ENSMUSG00000041958	Pigs	18

ENSMUSG00000020042	Btbd11	18		ENSMUSG00000017721	Pigt	20
ENSMUSG00000040298	Btbd16	20		ENSMUSG00000038383	Pigu	20
ENSMUSG00000000202	Btbd17	20		ENSMUSG00000043257	Pigv	7
ENSMUSG00000073771	Btbd19	20		ENSMUSG00000045140	Pigw	20
ENSMUSG00000003344	Btbd2	20		ENSMUSG00000023791	Pigx	20
ENSMUSG00000062098	Btbd3	19		ENSMUSG00000010607	Pigyl	19
ENSMUSG00000002803	Btbd6	20		ENSMUSG00000045625	Pigz	20
ENSMUSG00000041702	Btbd7	2		ENSMUSG00000003423	Pih1d1	1
ENSMUSG00000111375	Btbd8	3		ENSMUSG00000000167	Pih1d2	20
ENSMUSG00000062202	Btbd9	3		ENSMUSG00000025017	Pik3ap1	20
ENSMUSG00000082361	Btc	20		ENSMUSG00000030660	Pik3c2a	2
ENSMUSG00000021900	Btd	20		ENSMUSG00000026447	Pik3c2b	9
ENSMUSG00000021660	Btf3	20		ENSMUSG00000030228	Pik3c2g	12
ENSMUSG00000028568	Btf3l4	20		ENSMUSG00000033628	Pik3c3	20
ENSMUSG00000036478	Btg1	20		ENSMUSG00000027665	Pik3ca	3
ENSMUSG00000020423	Btg2	20		ENSMUSG00000032462	Pik3cb	20
ENSMUSG00000022863	Btg3	20		ENSMUSG00000039936	Pik3cd	18
ENSMUSG00000031264	Btk	20		ENSMUSG00000020573	Pik3cg	6
ENSMUSG00000053216	Btn2a2	20		ENSMUSG00000034614	Pik3ip1	9
ENSMUSG00000025217	Btrc	18		ENSMUSG00000041417	Pik3r1	2
ENSMUSG00000040084	Bub1b	6		ENSMUSG00000031834	Pik3r2	20
ENSMUSG00000066979	Bub3	20		ENSMUSG00000028698	Pik3r3	20
ENSMUSG00000032077	Bud13	20		ENSMUSG00000032571	Pik3r4	20
ENSMUSG00000005378	Bud23	20		ENSMUSG00000020901	Pik3r5	20
ENSMUSG00000038722	Bud31	20		ENSMUSG00000046207	Pik3r6	9
ENSMUSG00000071317	Bves	6		ENSMUSG00000025949	Pikfyve	2
ENSMUSG00000098098	Bvht	20		ENSMUSG00000024014	Pim1	19
ENSMUSG00000023988	Bysl	20		ENSMUSG00000031155	Pim2	20
ENSMUSG00000051223	Bzw1	3		ENSMUSG00000035828	Pim3	16
ENSMUSG00000020547	Bzw2	20		ENSMUSG00000032171	Pin1	3
ENSMUSG00000117926	C030004G16Rik	20		ENSMUSG00000079480	Pin4	12
ENSMUSG00000086600	C030005K06Rik	20		ENSMUSG00000028756	Pink1	16
ENSMUSG00000079183	C030005K15Rik	9		ENSMUSG00000021958	Pinx1	20
ENSMUSG00000116138	C030006K11Rik	20		ENSMUSG00000026737	Pip4k2a	20
ENSMUSG00000112409	C030006N10Rik	20		ENSMUSG00000018547	Pip4k2b	5
ENSMUSG00000085078	C030013C21Rik	20		ENSMUSG00000025417	Pip4k2c	20
ENSMUSG00000117314	C030013G03Rik	20		ENSMUSG00000035953	Pip4p1	20
ENSMUSG00000060380	C030014I23Rik	20		ENSMUSG00000028221	Pip4p2	20
ENSMUSG00000108181	C030015A19Rik	1		ENSMUSG00000028126	Pip5k1a	9
ENSMUSG00000106469	C030015E24Rik	3		ENSMUSG00000024867	Pip5k1b	5
ENSMUSG00000118202	C030017B01Rik	9		ENSMUSG00000034902	Pip5k1c	20
ENSMUSG00000107050	C030017G13Rik	20		ENSMUSG00000046854	Pip5kl1	18
ENSMUSG00000105352	C030018K13Rik	18		ENSMUSG00000017453	Pipox	20
ENSMUSG00000110027	C030029H02Rik	20		ENSMUSG00000031379	Pir	14
ENSMUSG00000106063	C030032O16Rik	20		ENSMUSG00000058818	Pirb	20
ENSMUSG00000073374	C030034I22Rik	9		ENSMUSG00000023452	Pisd	20
ENSMUSG00000097365	C030034L19Rik	20		ENSMUSG00000082286	Pisd-ps1	3
ENSMUSG00000087574	C030037D09Rik	20		ENSMUSG00000023795	Pisd-ps2	18
ENSMUSG00000109037	C030038I04Rik	18		ENSMUSG00000028669	Pithd1	20
ENSMUSG00000084873	C030047K22Rik	20		ENSMUSG00000017781	Pitpna	18
ENSMUSG00000103160	C130012C08Rik	18		ENSMUSG00000050017	Pitpnb	20
ENSMUSG00000105509	C130013H08Rik	20		ENSMUSG00000040430	Pitpnc1	18
ENSMUSG00000052951	C130021I20Rik	1		ENSMUSG00000029406	Pitpnm2	9
ENSMUSG00000102854	C130023A14Rik	20		ENSMUSG00000040543	Pitpnm3	1

ENSMUSG00000052848	C130026L21Rik	20		ENSMUSG00000021193	Pitrm1	19
ENSMUSG00000073627	C130036L24Rik	20		ENSMUSG00000028023	Pitx2	18
ENSMUSG00000085154	C130046K22Rik	20		ENSMUSG00000033644	Piwil2	20
ENSMUSG00000044092	C130050O18Rik	20		ENSMUSG00000034403	Pja1	20
ENSMUSG00000114605	C130051F05Rik	20		ENSMUSG00000024083	Pja2	9
ENSMUSG00000097557	C130060C02Rik	20		ENSMUSG00000032855	Pkd1	20
ENSMUSG00000050334	C130071C03Rik	4		ENSMUSG00000048827	Pkd1l3	20
ENSMUSG00000110246	C130073E24Rik	9		ENSMUSG00000034462	Pkd2	20
ENSMUSG00000039349	C130074G19Rik	20		ENSMUSG00000037578	Pkd2l1	1
ENSMUSG00000104735	C130075A20Rik	18		ENSMUSG00000014503	Pkd2l2	20
ENSMUSG00000108812	C130083A15Rik	19		ENSMUSG00000024247	Pkdcc	20
ENSMUSG00000104339	C130089K02Rik	20		ENSMUSG00000052496	Pkdrej	9
ENSMUSG00000000581	C1d	12		ENSMUSG00000038725	Pkhd1l1	20
ENSMUSG00000042460	C1galt1	20		ENSMUSG00000027499	Pkia	18
ENSMUSG00000048970	C1galt1c1	20		ENSMUSG00000019876	Pkib	18
ENSMUSG00000036887	C1qa	5		ENSMUSG00000035268	Pkig	9
ENSMUSG00000036905	C1qb	12		ENSMUSG00000032294	Pkm	2
ENSMUSG00000018446	C1qbp	20		ENSMUSG00000023908	Pkmyt1	20
ENSMUSG00000036896	C1qc	12		ENSMUSG00000057672	Pkn1	20
ENSMUSG00000045532	C1ql1	20		ENSMUSG00000004591	Pkn2	12
ENSMUSG00000036907	C1ql2	5		ENSMUSG00000026785	Pkn3	20
ENSMUSG00000049630	C1ql3	5		ENSMUSG00000006705	Pknox1	20
ENSMUSG00000017446	C1qtnf1	20		ENSMUSG00000035934	Pknox2	6
ENSMUSG00000023571	C1qtnf12	3		ENSMUSG00000041957	Pkp2	5
ENSMUSG00000022440	C1qtnf6	20		ENSMUSG00000026991	Pkp4	16
ENSMUSG00000024371	C2	1		ENSMUSG00000002847	Pla1a	20
ENSMUSG00000046219	C230012O17Rik	20		ENSMUSG00000027999	Pla2g12a	19
ENSMUSG00000117171	C230013L11Rik	20		ENSMUSG00000031903	Pla2g15	20
ENSMUSG00000087461	C230014O12Rik	19		ENSMUSG00000034579	Pla2g3	20
ENSMUSG00000105202	C230031I18Rik	3		ENSMUSG00000056220	Pla2g4a	1
ENSMUSG00000086181	C230034O21Rik	5		ENSMUSG00000098488	Pla2g4b	20
ENSMUSG00000085024	C230035I16Rik	20		ENSMUSG00000033847	Pla2g4c	20
ENSMUSG00000084915	C230037L18Rik	20		ENSMUSG00000050211	Pla2g4e	19
ENSMUSG00000085560	C230038L03Rik	20		ENSMUSG00000041193	Pla2g5	20
ENSMUSG00000110751	C230053D17Rik	20		ENSMUSG00000042632	Pla2g6	20
ENSMUSG00000110523	C230057M02Rik	18		ENSMUSG00000023913	Pla2g7	2
ENSMUSG00000063623	C230062I16Rik	12		ENSMUSG00000054580	Pla2r1	20
ENSMUSG00000085771	C230066G23Rik	20		ENSMUSG00000028577	Plaa	20
ENSMUSG00000112121	C230072F16Rik	20		ENSMUSG00000022525	Plaat1	1
ENSMUSG00000102212	C230085N15Rik	20		ENSMUSG00000060675	Plaat3	3
ENSMUSG00000115471	C230086J09Rik	19		ENSMUSG00000003282	Plag1	9
ENSMUSG00000106205	C230096K16Rik	1		ENSMUSG00000019817	Plagl1	20
ENSMUSG00000045975	C2cd2	20		ENSMUSG00000051413	Plagl2	20
ENSMUSG00000032120	C2cd2l	9		ENSMUSG00000031538	Plat	20
ENSMUSG00000047248	C2cd3	20		ENSMUSG00000074863	Platr25	20
ENSMUSG00000045912	C2cd4c	1		ENSMUSG00000021822	Plau	20
ENSMUSG00000030279	C2cd5	9		ENSMUSG00000046223	Plaur	20
ENSMUSG00000024164	C3	1		ENSMUSG00000029134	Plb1	20
ENSMUSG00000097930	C330002G04Rik	20		ENSMUSG00000029598	Plbd2	20
ENSMUSG00000006423	C330007P06Rik	12		ENSMUSG00000051177	Plcb1	9
ENSMUSG00000056753	C330011M18Rik	14		ENSMUSG00000040061	Plcb2	20
ENSMUSG00000097093	C330013E15Rik	18		ENSMUSG00000024960	Plcb3	20
ENSMUSG00000024592	C330018D20Rik	20		ENSMUSG00000039943	Plcb4	18
ENSMUSG00000040552	C3ar1	20		ENSMUSG00000010660	Plcd1	20

ENSMUSG00000108359	C430039J01Rik	20		ENSMUSG00000020937	Plcd3	20
ENSMUSG00000015451	C4a	3		ENSMUSG00000026173	Plcd4	4
ENSMUSG00000073418	C4b	20		ENSMUSG00000024998	Plce1	5
ENSMUSG00000085408	C530005A16Rik	20		ENSMUSG00000016933	Plcg1	20
ENSMUSG00000036377	C530008M17Rik	9		ENSMUSG00000034330	Plcg2	20
ENSMUSG00000074737	C530025M09Rik	20		ENSMUSG00000036834	Plch1	18
ENSMUSG00000106706	C530043K16Rik	20		ENSMUSG00000029055	Plch2	19
ENSMUSG00000107667	C530044C16Rik	20		ENSMUSG00000038349	Plcl1	9
ENSMUSG00000049130	C5ar1	20		ENSMUSG00000038910	Plcl2	9
ENSMUSG00000074361	C5ar2	20		ENSMUSG00000064247	Plcxd1	1
ENSMUSG00000112663	C630031E19Rik	9		ENSMUSG00000087141	Plcxd2	9
ENSMUSG00000084910	C630043F03Rik	20		ENSMUSG00000049148	Plcxd3	20
ENSMUSG00000114448	C630044B11Rik	20		ENSMUSG00000030230	Plcz1	3
ENSMUSG00000079105	C7	20		ENSMUSG00000027695	Pld1	9
ENSMUSG00000114469	C730002L08Rik	20		ENSMUSG00000020828	Pld2	20
ENSMUSG00000079242	C730034F03Rik	12		ENSMUSG00000003363	Pld3	9
ENSMUSG00000107054	C730045M19Rik	2		ENSMUSG00000055214	Pld5	19
ENSMUSG00000050390	C77080	20		ENSMUSG00000043648	Pld6	20
ENSMUSG00000087242	C78197	20		ENSMUSG00000022565	Plec	20
ENSMUSG00000110710	C78859	9		ENSMUSG00000020120	Plek	20
ENSMUSG00000105741	C79130	20		ENSMUSG00000040268	Plekha1	20
ENSMUSG00000046679	C87436	20		ENSMUSG00000031557	Plekha2	1
ENSMUSG00000097574	C920006O11Rik	20		ENSMUSG00000002733	Plekha3	20
ENSMUSG00000080727	C920021L13Rik	20		ENSMUSG00000040428	Plekha4	20
ENSMUSG00000028300	C9orf72	20		ENSMUSG00000030231	Plekha5	9
ENSMUSG00000063897	CAAA01118383.1	9		ENSMUSG00000041757	Plekha6	18
ENSMUSG00000095742	CAAA01147332.1	20		ENSMUSG00000045659	Plekha7	18
ENSMUSG00000028578	Caap1	20		ENSMUSG00000005225	Plekha8	18
ENSMUSG00000036707	Cab39	12		ENSMUSG00000030701	Plekhb1	16
ENSMUSG00000021981	Cab39l	13		ENSMUSG00000026123	Plekhb2	20
ENSMUSG00000019945	Cabcoco1	13		ENSMUSG00000066438	Plekhd1	18
ENSMUSG00000020196	Cabin1	20		ENSMUSG00000074170	Plekhf1	20
ENSMUSG00000040957	Cables1	20		ENSMUSG00000049969	Plekhf2	20
ENSMUSG00000038990	Cables2	18		ENSMUSG00000040624	Plekhg1	18
ENSMUSG00000029544	Cabp1	9		ENSMUSG00000037552	Plekhg2	20
ENSMUSG00000009075	Cabp7	5		ENSMUSG00000052609	Plekhg3	16
ENSMUSG00000024430	Cabyr	12		ENSMUSG00000014782	Plekhg4	1
ENSMUSG00000015488	Cacfd1	1		ENSMUSG00000039713	Plekhg5	9
ENSMUSG00000028532	Cachd1	19		ENSMUSG00000060716	Plekhh1	16
ENSMUSG00000034656	Cacna1a	3		ENSMUSG00000040852	Plekhh2	1
ENSMUSG00000004113	Cacna1b	16		ENSMUSG00000035172	Plekhh3	2
ENSMUSG00000051331	Cacna1c	18		ENSMUSG00000035278	Plekhj1	20
ENSMUSG00000015968	Cacna1d	20		ENSMUSG00000034247	Plekhm1	20
ENSMUSG00000004110	Cacna1e	9		ENSMUSG00000028917	Plekhm2	16
ENSMUSG00000020866	Cacna1g	18		ENSMUSG00000051344	Plekhm3	2
ENSMUSG00000024112	Cacna1h	6		ENSMUSG00000078485	Plekhn1	2
ENSMUSG00000022416	Cacna1i	9		ENSMUSG00000015745	Plekho1	20
ENSMUSG00000026407	Cacna1s	20		ENSMUSG00000050721	Plekho2	20
ENSMUSG00000040118	Cacna2d1	16		ENSMUSG00000016495	Plgrkt	1
ENSMUSG00000010066	Cacna2d2	19		ENSMUSG00000028494	Plin2	20
ENSMUSG00000021991	Cacna2d3	9		ENSMUSG00000024197	Plin3	20
ENSMUSG00000041460	Cacna2d4	20		ENSMUSG00000002831	Plin4	20
ENSMUSG00000020882	Cacnb1	9		ENSMUSG00000011305	Plin5	20
ENSMUSG00000057914	Cacnb2	8		ENSMUSG00000030867	Plk1	20

ENSMUSG00000003352	Cacnb3	9		ENSMUSG00000021701	Plk2	9
ENSMUSG00000017412	Cacnb4	7		ENSMUSG00000028680	Plk3	20
ENSMUSG00000020722	Cacng1	20		ENSMUSG00000035486	Plk5	1
ENSMUSG00000019146	Cacng2	18		ENSMUSG00000031775	Pllp	16
ENSMUSG00000066189	Cacng3	9		ENSMUSG00000038583	Pln	20
ENSMUSG00000020723	Cacng4	9		ENSMUSG00000019055	Plod1	20
ENSMUSG00000040373	Cacng5	18		ENSMUSG00000032374	Plod2	20
ENSMUSG00000034889	Cactin	20		ENSMUSG00000004846	Plod3	3
ENSMUSG00000033417	Cacul1	19		ENSMUSG00000031425	Plp1	16
ENSMUSG00000014226	Cacybp	20		ENSMUSG00000031146	Plp2	20
ENSMUSG00000013629	Cad	20		ENSMUSG00000031485	Plpbp	20
ENSMUSG00000032076	Cadm1	20		ENSMUSG00000021759	Plpp1	19
ENSMUSG00000064115	Cadm2	7		ENSMUSG00000052151	Plpp2	9
ENSMUSG00000005338	Cadm3	1		ENSMUSG00000028517	Plpp3	1
ENSMUSG00000054793	Cadm4	18		ENSMUSG00000070366	Plpp4	18
ENSMUSG00000054423	Cadps	19		ENSMUSG00000031570	Plpp5	20
ENSMUSG00000017978	Cadps2	5		ENSMUSG00000040105	Plpp6	20
ENSMUSG00000044566	Cage1	20		ENSMUSG00000051373	Plpp7	2
ENSMUSG00000028222	Calb1	9		ENSMUSG00000063446	Plppr1	9
ENSMUSG00000003657	Calb2	17		ENSMUSG00000040563	Plppr2	1
ENSMUSG00000030669	Calca	20		ENSMUSG00000035835	Plppr3	12
ENSMUSG00000023055	Calcoco1	3		ENSMUSG00000044667	Plppr4	18
ENSMUSG00000023964	Calcr	20		ENSMUSG00000033342	Plppr5	6
ENSMUSG00000059588	Calcrl	20		ENSMUSG00000027998	Plrg1	20
ENSMUSG00000029761	Cald1	20		ENSMUSG00000049493	Pls1	20
ENSMUSG00000033033	Calhm2	20		ENSMUSG00000016382	Pls3	19
ENSMUSG00000049872	Calhm5	2		ENSMUSG00000032369	Plscr1	20
ENSMUSG00000001175	Calm1	9		ENSMUSG00000032372	Plscr2	20
ENSMUSG00000036438	Calm2	18		ENSMUSG00000019461	Plscr3	20
ENSMUSG00000019370	Calm3	20		ENSMUSG00000032377	Plscr4	13
ENSMUSG00000032246	Calml4	13		ENSMUSG00000017754	Pltp	13
ENSMUSG00000060371	Caln1	9		ENSMUSG00000034845	Plvap	1
ENSMUSG00000003814	Calr	18		ENSMUSG00000017417	Plxdc1	2
ENSMUSG00000019732	Calr3	20		ENSMUSG00000026748	Plxdc2	18
ENSMUSG00000028558	Calr4	16		ENSMUSG00000030084	Plxna1	19
ENSMUSG00000029767	Calu	20		ENSMUSG00000026640	Plxna2	7
ENSMUSG00000025468	Caly	3		ENSMUSG00000031398	Plxna3	9
ENSMUSG00000030272	Camk1	17		ENSMUSG00000029765	Plxna4	5
ENSMUSG00000039145	Camk1d	9		ENSMUSG00000086763	Plxna4os1	20
ENSMUSG00000016179	Camk1g	20		ENSMUSG00000053646	Plxnb1	20
ENSMUSG00000024617	Camk2a	9		ENSMUSG00000036606	Plxnb2	13
ENSMUSG00000057897	Camk2b	9		ENSMUSG00000031385	Plxnb3	1
ENSMUSG00000053819	Camk2d	9		ENSMUSG00000074785	Plxnc1	17
ENSMUSG00000021820	Camk2g	19		ENSMUSG00000030123	Plxnd1	9
ENSMUSG00000046447	Camk2n1	9		ENSMUSG00000042251	Pm20d1	10
ENSMUSG00000051146	Camk2n2	18		ENSMUSG00000054659	Pm20d2	18
ENSMUSG00000038128	Camk4	9		ENSMUSG00000024521	Pmaip1	20
ENSMUSG00000020785	Camkk1	1		ENSMUSG00000035383	Pmch	18
ENSMUSG00000029471	Camkk2	9		ENSMUSG00000038400	Pmepa1	9
ENSMUSG00000071037	Camkmt	20		ENSMUSG00000087600	Pmepa1os	20
ENSMUSG00000032936	Camkv	9		ENSMUSG00000028066	Pmf1	20
ENSMUSG00000021501	Caml	20		ENSMUSG00000031727	Pmfbp1	18
ENSMUSG00000026933	Camsap1	20		ENSMUSG00000036986	Pml	20
ENSMUSG00000041570	Camsap2	3		ENSMUSG00000022474	Pmm1	20

ENSMUSG00000044433	Camsap3	20		ENSMUSG00000022711	Pmm2	20
ENSMUSG00000014592	Camta1	19		ENSMUSG00000018217	Pmp22	16
ENSMUSG00000040712	Camta2	5		ENSMUSG00000026926	Pmpca	13
ENSMUSG00000020114	Cand1	3		ENSMUSG00000029017	Pmpcb	20
ENSMUSG00000030319	Cand2	20		ENSMUSG00000026098	Pms1	20
ENSMUSG00000025575	Cant1	20		ENSMUSG00000079109	Pms2	20
ENSMUSG00000020368	Canx	19		ENSMUSG00000027952	Pmvk	18
ENSMUSG00000028656	Cap1	9		ENSMUSG00000002012	Pnck	20
ENSMUSG00000021373	Cap2	9		ENSMUSG00000028248	Pnisr	20
ENSMUSG00000056737	Capg	20		ENSMUSG00000026179	Pnkd	9
ENSMUSG00000024942	Capn1	12		ENSMUSG00000002963	Pnkp	20
ENSMUSG00000026270	Capn10	20		ENSMUSG00000073460	Pnldc1	20
ENSMUSG00000058626	Capn11	20		ENSMUSG00000046008	Pnlip	20
ENSMUSG00000054083	Capn12	20		ENSMUSG00000054383	Pnma1	20
ENSMUSG00000037326	Capn15	1		ENSMUSG00000046204	Pnma2	16
ENSMUSG00000026509	Capn2	1		ENSMUSG00000046287	Pnma3	19
ENSMUSG00000079110	Capn3	20		ENSMUSG00000041141	Pnmal1	16
ENSMUSG00000035547	Capn5	20		ENSMUSG00000070802	Pnmal2	20
ENSMUSG00000067276	Capn6	13		ENSMUSG00000038216	Pnmt	20
ENSMUSG00000021893	Capn7	20		ENSMUSG00000020994	Pnn	20
ENSMUSG00000001794	Capns1	7		ENSMUSG00000020116	Pno1	12
ENSMUSG00000078144	Capns2	20		ENSMUSG00000045731	Pnoc	6
ENSMUSG00000027184	Caprin1	7		ENSMUSG00000115338	Pnp	20
ENSMUSG00000030309	Caprin2	9		ENSMUSG00000068417	Pnp2	19
ENSMUSG00000035694	Caps2	20		ENSMUSG00000043286	Pnpla1	20
ENSMUSG00000039676	Capsl	14		ENSMUSG00000025509	Pnpla2	20
ENSMUSG00000070372	Capza1	18		ENSMUSG00000041653	Pnpla3	20
ENSMUSG00000015733	Capza2	4		ENSMUSG00000004565	Pnpla6	9
ENSMUSG00000028745	Capzb	12		ENSMUSG00000036833	Pnpla7	20
ENSMUSG00000056158	Car10	19		ENSMUSG00000036257	Pnpla8	3
ENSMUSG00000003273	Car11	9		ENSMUSG00000018659	Pnpo	9
ENSMUSG00000032373	Car12	9		ENSMUSG00000020464	Pnpt1	20
ENSMUSG00000027555	Car13	20		ENSMUSG00000040128	Pnrc1	12
ENSMUSG00000038526	Car14	13		ENSMUSG00000028675	Pnrc2	20
ENSMUSG00000090236	Car15	20		ENSMUSG00000023345	Poc1a	20
ENSMUSG00000027562	Car2	16		ENSMUSG00000019952	Poc1b	20
ENSMUSG00000000805	Car4	18		ENSMUSG00000021671	Poc5	20
ENSMUSG00000031373	Car5b	9		ENSMUSG00000028600	Podn	13
ENSMUSG00000031883	Car7	20		ENSMUSG00000025608	Podxl	9
ENSMUSG00000041261	Car8	9		ENSMUSG00000033152	Podxl2	6
ENSMUSG00000028463	Car9	20		ENSMUSG00000046020	Pofut1	20
ENSMUSG00000033170	Card10	20		ENSMUSG00000020260	Pofut2	20
ENSMUSG00000036526	Card11	20		ENSMUSG00000040596	Pogk	18
ENSMUSG00000037960	Card19	9		ENSMUSG00000034064	Poglut1	20
ENSMUSG00000041849	Card6	20		ENSMUSG00000026047	Poglut2	20
ENSMUSG00000026928	Card9	12		ENSMUSG00000034487	Poglut3	20
ENSMUSG00000026017	Carf	20		ENSMUSG00000038902	Pogz	6
ENSMUSG00000008393	Carhsp1	20		ENSMUSG00000006678	Pola1	20
ENSMUSG00000097638	Carlr	20		ENSMUSG00000024833	Pola2	20
ENSMUSG00000032185	Carm1	20		ENSMUSG00000031536	Polb	20
ENSMUSG00000021338	Carmil1	20		ENSMUSG00000038644	Pold1	20
ENSMUSG00000050357	Carmil2	19		ENSMUSG00000020471	Pold2	20
ENSMUSG00000022211	Carmil3	9		ENSMUSG00000030726	Pold3	9
ENSMUSG00000097324	Carmn	20		ENSMUSG00000001100	Poldip2	20

ENSMUSG00000024726	Carnmt1	20		ENSMUSG00000041815	Poldip3	20
ENSMUSG00000010755	Cars	18		ENSMUSG00000007080	Pole	20
ENSMUSG00000056228	Cars2	20		ENSMUSG00000020974	Pole2	20
ENSMUSG00000021647	Cartpt	17		ENSMUSG00000028394	Pole3	12
ENSMUSG00000043541	Casc1	20		ENSMUSG00000030042	Pole4	18
ENSMUSG00000078676	Casc3	20		ENSMUSG00000039176	Polg	20
ENSMUSG00000060227	Casc4	19		ENSMUSG00000020718	Polg2	3
ENSMUSG00000015189	Casd1	20		ENSMUSG00000023953	Polh	20
ENSMUSG00000031012	Cask	2		ENSMUSG00000038425	Poli	20
ENSMUSG00000033597	Caskin1	19		ENSMUSG00000021668	Polk	20
ENSMUSG00000034471	Caskin2	20		ENSMUSG00000025218	Poll	20
ENSMUSG00000025888	Casp1	20		ENSMUSG00000020474	Polm	20
ENSMUSG00000025887	Casp12	20		ENSMUSG00000045102	Poln	10
ENSMUSG00000029863	Casp2	20		ENSMUSG00000034206	Polq	9
ENSMUSG00000031628	Casp3	20		ENSMUSG00000049553	Polr1a	20
ENSMUSG00000033538	Casp4	12		ENSMUSG00000027395	Polr1b	5
ENSMUSG00000027997	Casp6	20		ENSMUSG00000067148	Polr1c	20
ENSMUSG00000025076	Casp7	20		ENSMUSG00000029642	Polr1d	12
ENSMUSG00000026029	Casp8	17		ENSMUSG00000028318	Polr1e	13
ENSMUSG00000028282	Casp8ap2	20		ENSMUSG00000005198	Polr2a	1
ENSMUSG00000028914	Casp9	20		ENSMUSG00000029250	Polr2b	20
ENSMUSG00000007122	Casq1	20		ENSMUSG00000031783	Polr2c	20
ENSMUSG00000027861	Casq2	20		ENSMUSG00000024258	Polr2d	20
ENSMUSG00000074570	Cass4	20		ENSMUSG00000004667	Polr2e	3
ENSMUSG00000021585	Cast	9		ENSMUSG00000033020	Polr2f	20
ENSMUSG00000020424	Castor1	4		ENSMUSG00000071662	Polr2g	10
ENSMUSG00000015944	Castor2	3		ENSMUSG00000021018	Polr2h	20
ENSMUSG00000028977	Casz1	20		ENSMUSG00000019738	Polr2i	9
ENSMUSG00000027187	Cat	20		ENSMUSG00000039771	Polr2j	12
ENSMUSG00000073650	Catip	13		ENSMUSG00000045996	Polr2k	20
ENSMUSG00000033486	Catsper2	9		ENSMUSG00000082981	Polr2k-ps	20
ENSMUSG00000021499	Catsper3	9		ENSMUSG00000038489	Polr2l	20
ENSMUSG00000040828	Catsperd	20		ENSMUSG00000032199	Polr2m	9
ENSMUSG00000091476	Catspere2	12		ENSMUSG00000025280	Polr3a	9
ENSMUSG00000049676	Catsperg1	20		ENSMUSG00000034453	Polr3b	20
ENSMUSG00000050623	Catsperz	18		ENSMUSG00000028099	Polr3c	9
ENSMUSG00000007655	Cav1	20		ENSMUSG00000000776	Polr3d	20
ENSMUSG00000000058	Cav2	9		ENSMUSG00000030880	Polr3e	20
ENSMUSG00000004044	Cavin1	20		ENSMUSG00000027427	Polr3f	20
ENSMUSG00000045954	Cavin2	20		ENSMUSG00000035834	Polr3g	20
ENSMUSG00000037060	Cavin3	3		ENSMUSG00000028104	Polr3gl	20
ENSMUSG00000028348	Cavin4	20		ENSMUSG00000022476	Polr3h	12
ENSMUSG00000035640	Cbarp	12		ENSMUSG00000038628	Polr3k	20
ENSMUSG00000038533	Cbfa2t2	20		ENSMUSG00000020329	Polrmt	7
ENSMUSG00000087034	Cbfa2t2-ps1	20		ENSMUSG00000053293	Pom121	20
ENSMUSG00000006362	Cbfa2t3	18		ENSMUSG00000020660	Pomc	20
ENSMUSG00000031885	Cbfb	20		ENSMUSG00000028700	Pomgnt1	12
ENSMUSG00000034342	Cbl	20		ENSMUSG00000066235	Pomgnt2	20
ENSMUSG00000022637	Cblb	1		ENSMUSG00000037251	Pomk	20
ENSMUSG00000020659	Cbll1	20		ENSMUSG00000029649	Pomp	20
ENSMUSG00000031654	Cbln1	9		ENSMUSG00000039254	Pomt1	1
ENSMUSG00000024647	Cbln2	18		ENSMUSG00000034126	Pomt2	20
ENSMUSG00000067578	Cbln4	17		ENSMUSG00000032667	Pon2	20
ENSMUSG00000051483	Cbr1	20		ENSMUSG00000029759	Pon3	13

ENSMUSG00000022947	Cbr3	9		ENSMUSG00000022325	Pop1	20
ENSMUSG00000031641	Cbr4	20		ENSMUSG00000030423	Pop4	20
ENSMUSG00000024039	Cbs	1		ENSMUSG00000060152	Pop5	12
ENSMUSG00000024878	Cbwd1	20		ENSMUSG00000029715	Pop7	10
ENSMUSG00000018666	Cbx1	20		ENSMUSG00000019848	Popdc3	20
ENSMUSG00000025577	Cbx2	20		ENSMUSG00000005514	Por	20
ENSMUSG00000029836	Cbx3	13		ENSMUSG00000031169	Porcn	20
ENSMUSG00000057886	Cbx3-ps6	20		ENSMUSG00000027750	Postn	9
ENSMUSG00000059647	Cbx3-ps7	20		ENSMUSG00000029676	Pot1a	2
ENSMUSG00000039989	Cbx4	1		ENSMUSG00000026565	Pou2f1	19
ENSMUSG00000009575	Cbx5	3		ENSMUSG00000008496	Pou2f2	20
ENSMUSG00000089715	Cbx6	9		ENSMUSG00000090125	Pou3f1	9
ENSMUSG00000053411	Cbx7	1		ENSMUSG00000095139	Pou3f2	19
ENSMUSG00000025578	Cbx8	1		ENSMUSG00000045515	Pou3f3	7
ENSMUSG00000022428	Cby1	20		ENSMUSG00000056854	Pou3f4	18
ENSMUSG00000036686	Cc2d1a	1		ENSMUSG00000048349	Pou4f1	1
ENSMUSG00000028582	Cc2d1b	20		ENSMUSG00000031688	Pou4f2	20
ENSMUSG00000039765	Cc2d2a	19		ENSMUSG00000093668	Pou5f2	20
ENSMUSG00000108929	Cc2d2b	20		ENSMUSG00000009739	Pou6f1	6
ENSMUSG00000020074	Ccar1	1		ENSMUSG00000009734	Pou6f2	9
ENSMUSG00000033712	Ccar2	20		ENSMUSG00000020089	Ppa1	12
ENSMUSG00000046318	Ccbe1	5		ENSMUSG00000028013	Ppa2	11
ENSMUSG00000063605	Ccdc102a	20		ENSMUSG00000004100	Ppan	20
ENSMUSG00000020930	Ccdc103	20		ENSMUSG00000022383	Ppara	20
ENSMUSG00000078442	Ccdc105	20		ENSMUSG00000002250	Ppard	18
ENSMUSG00000035228	Ccdc106	19		ENSMUSG00000000440	Pparg	9
ENSMUSG00000028461	Ccdc107	12		ENSMUSG00000029167	Ppargc1a	12
ENSMUSG00000071104	Ccdc110	20		ENSMUSG00000033871	Ppargc1b	9
ENSMUSG00000071855	Ccdc112	18		ENSMUSG00000029246	Ppat	20
ENSMUSG00000036598	Ccdc113	13		ENSMUSG00000063849	Ppcdc	20
ENSMUSG00000040189	Ccdc114	13		ENSMUSG00000028636	Ppcs	20
ENSMUSG00000042111	Ccdc115	20		ENSMUSG00000016344	Ppdpf	12
ENSMUSG00000022768	Ccdc116	20		ENSMUSG00000062168	Ppef1	20
ENSMUSG00000020482	Ccdc117	20		ENSMUSG00000029410	Ppef2	9
ENSMUSG00000019659	Ccdc12	12		ENSMUSG00000037519	Ppfia1	20
ENSMUSG00000031150	Ccdc120	20		ENSMUSG00000053825	Ppfia2	5
ENSMUSG00000034795	Ccdc122	20		ENSMUSG00000003863	Ppfia3	16
ENSMUSG00000007721	Ccdc124	20		ENSMUSG00000026458	Ppfia4	20
ENSMUSG00000048924	Ccdc125	20		ENSMUSG00000016487	Ppfibp1	9
ENSMUSG00000050786	Ccdc126	20		ENSMUSG00000036528	Ppfibp2	16
ENSMUSG00000021578	Ccdc127	20		ENSMUSG00000036167	Pphln1	9
ENSMUSG00000079235	Ccdc13	18		ENSMUSG00000071866	Ppia	12
ENSMUSG00000004994	Ccdc130	4		ENSMUSG00000032383	Ppib	2
ENSMUSG00000068114	Ccdc134	20		ENSMUSG00000024538	Ppic	20
ENSMUSG00000029769	Ccdc136	18		ENSMUSG00000027804	Ppid	20
ENSMUSG00000049957	Ccdc137	20		ENSMUSG00000028651	Ppie	12
ENSMUSG00000038010	Ccdc138	20		ENSMUSG00000021868	Ppif	20
ENSMUSG00000022833	Ccdc14	20		ENSMUSG00000042133	Ppig	5
ENSMUSG00000044033	Ccdc141	20		ENSMUSG00000060288	Ppih	20
ENSMUSG00000107499	Ccdc142	20		ENSMUSG00000024007	Ppil1	11
ENSMUSG00000087578	Ccdc142os	20		ENSMUSG00000022771	Ppil2	19
ENSMUSG00000064280	Ccdc146	20		ENSMUSG00000026035	Ppil3	20
ENSMUSG00000036641	Ccdc148	20		ENSMUSG00000015757	Ppil4	20
ENSMUSG00000045790	Ccdc149	8		ENSMUSG00000078451	Ppil6	20

ENSMUSG00000034303	Ccdc15	20		ENSMUSG00000033526	Ppip5k1	20
ENSMUSG00000025983	Ccdc150	20		ENSMUSG00000040648	Ppip5k2	20
ENSMUSG00000039632	Ccdc151	3		ENSMUSG00000039457	Ppl	20
ENSMUSG00000070306	Ccdc153	14		ENSMUSG00000021096	Ppm1a	12
ENSMUSG00000038292	Ccdc155	3		ENSMUSG00000061130	Ppm1b	20
ENSMUSG00000051427	Ccdc157	19		ENSMUSG00000020525	Ppm1d	9
ENSMUSG00000050050	Ccdc158	20		ENSMUSG00000046442	Ppm1e	19
ENSMUSG00000006241	Ccdc159	20		ENSMUSG00000026181	Ppm1f	20
ENSMUSG00000073207	Ccdc160	20		ENSMUSG00000029147	Ppm1g	3
ENSMUSG00000075225	Ccdc162	14		ENSMUSG00000034613	Ppm1h	19
ENSMUSG00000028689	Ccdc163	20		ENSMUSG00000002228	Ppm1j	20
ENSMUSG00000098176	Ccdc166	20		ENSMUSG00000037826	Ppm1k	20
ENSMUSG00000024018	Ccdc167	20		ENSMUSG00000027784	Ppm1l	19
ENSMUSG00000048655	Ccdc169	20		ENSMUSG00000020253	Ppm1m	20
ENSMUSG00000034035	Ccdc17	20		ENSMUSG00000030718	Ppme1	20
ENSMUSG00000019767	Ccdc170	14		ENSMUSG00000099746	Ppnr	18
ENSMUSG00000052407	Ccdc171	3		ENSMUSG00000062729	Ppox	20
ENSMUSG00000070883	Ccdc173	18		ENSMUSG00000040385	Ppp1ca	12
ENSMUSG00000034083	Ccdc174	20		ENSMUSG00000014956	Ppp1cb	9
ENSMUSG00000062961	Ccdc177	2		ENSMUSG00000004455	Ppp1cc	3
ENSMUSG00000056531	Ccdc18	20		ENSMUSG00000100153	Ppp1ccb	20
ENSMUSG00000035539	Ccdc180	14		ENSMUSG00000039220	Ppp1r10	20
ENSMUSG00000026578	Ccdc181	20		ENSMUSG00000036398	Ppp1r11	11
ENSMUSG00000029875	Ccdc184	17		ENSMUSG00000019907	Ppp1r12a	2
ENSMUSG00000035173	Ccdc186	20		ENSMUSG00000073557	Ppp1r12b	16
ENSMUSG00000048038	Ccdc187	9		ENSMUSG00000019254	Ppp1r12c	3
ENSMUSG00000057176	Ccdc189	18		ENSMUSG00000021285	Ppp1r13b	20
ENSMUSG00000070532	Ccdc190	20		ENSMUSG00000040734	Ppp1r13l	20
ENSMUSG00000022701	Ccdc191	1		ENSMUSG00000037166	Ppp1r14a	16
ENSMUSG00000058925	Ccdc192	20		ENSMUSG00000056612	Ppp1r14b	20
ENSMUSG00000031143	Ccdc22	20		ENSMUSG00000040653	Ppp1r14c	20
ENSMUSG00000078588	Ccdc24	20		ENSMUSG00000040435	Ppp1r15a	20
ENSMUSG00000022035	Ccdc25	20		ENSMUSG00000046062	Ppp1r15b	20
ENSMUSG00000059554	Ccdc28a	20		ENSMUSG00000033819	Ppp1r16a	20
ENSMUSG00000028795	Ccdc28b	20		ENSMUSG00000037754	Ppp1r16b	9
ENSMUSG00000026676	Ccdc3	20		ENSMUSG00000034595	Ppp1r18	20
ENSMUSG00000028637	Ccdc30	9		ENSMUSG00000022490	Ppp1r1a	3
ENSMUSG00000039983	Ccdc32	20		ENSMUSG00000061718	Ppp1r1b	9
ENSMUSG00000037716	Ccdc33	14		ENSMUSG00000047714	Ppp1r2	9
ENSMUSG00000027160	Ccdc34	20		ENSMUSG00000099662	Ppp1r2-ps2	9
ENSMUSG00000027676	Ccdc39	18		ENSMUSG00000058816	Ppp1r2-ps3	20
ENSMUSG00000039963	Ccdc40	20		ENSMUSG00000059237	Ppp1r2-ps4	20
ENSMUSG00000045915	Ccdc42	19		ENSMUSG00000095869	Ppp1r2-ps6	9
ENSMUSG00000020925	Ccdc43	18		ENSMUSG00000034709	Ppp1r21	20
ENSMUSG00000078622	Ccdc47	20		ENSMUSG00000035829	Ppp1r26	9
ENSMUSG00000038127	Ccdc50	9		ENSMUSG00000035179	Ppp1r32	14
ENSMUSG00000081553	Ccdc50-ps	18		ENSMUSG00000029725	Ppp1r35	20
ENSMUSG00000025645	Ccdc51	20		ENSMUSG00000052221	Ppp1r36	20
ENSMUSG00000048445	Ccdc57	20		ENSMUSG00000051403	Ppp1r37	9
ENSMUSG00000075229	Ccdc58	20		ENSMUSG00000042717	Ppp1r3a	18
ENSMUSG00000019897	Ccdc59	12		ENSMUSG00000046794	Ppp1r3b	13
ENSMUSG00000048701	Ccdc6	20		ENSMUSG00000067279	Ppp1r3c	20
ENSMUSG00000043913	Ccdc60	20		ENSMUSG00000049999	Ppp1r3d	20
ENSMUSG00000074358	Ccdc61	20		ENSMUSG00000072494	Ppp1r3e	1

ENSMUSG00000061882	Ccdc62	20		ENSMUSG00000039556	Ppp1r3f	9
ENSMUSG00000003354	Ccdc65	9		ENSMUSG00000050423	Ppp1r3g	19
ENSMUSG00000046753	Ccdc66	20		ENSMUSG00000025916	Ppp1r42	20
ENSMUSG00000049588	Ccdc69	9		ENSMUSG00000026275	Ppp1r7	9
ENSMUSG00000049305	Ccdc71	12		ENSMUSG00000028882	Ppp1r8	20
ENSMUSG00000090946	Ccdc71l	1		ENSMUSG00000032827	Ppp1r9a	9
ENSMUSG00000045106	Ccdc73	20		ENSMUSG00000038976	Ppp1r9b	1
ENSMUSG00000041617	Ccdc74a	2		ENSMUSG00000020349	Ppp2ca	20
ENSMUSG00000030177	Ccdc77	12		ENSMUSG00000009630	Ppp2cb	9
ENSMUSG00000071202	Ccdc78	14		ENSMUSG00000007564	Ppp2r1a	20
ENSMUSG00000041117	Ccdc8	20		ENSMUSG00000032058	Ppp2r1b	20
ENSMUSG00000022665	Ccdc80	20		ENSMUSG00000022052	Ppp2r2a	9
ENSMUSG00000039391	Ccdc81	14		ENSMUSG00000024500	Ppp2r2b	20
ENSMUSG00000079084	Ccdc82	17		ENSMUSG00000029120	Ppp2r2c	9
ENSMUSG00000043923	Ccdc84	20		ENSMUSG00000041769	Ppp2r2d	20
ENSMUSG00000032878	Ccdc85a	19		ENSMUSG00000043154	Ppp2r3a	1
ENSMUSG00000095098	Ccdc85b	3		ENSMUSG00000021022	Ppp2r3c	20
ENSMUSG00000084883	Ccdc85c	20		ENSMUSG00000026626	Ppp2r5a	9
ENSMUSG00000024732	Ccdc86	20		ENSMUSG00000017843	Ppp2r5c	20
ENSMUSG00000067872	Ccdc87	20		ENSMUSG00000059409	Ppp2r5d	1
ENSMUSG00000032740	Ccdc88a	2		ENSMUSG00000021051	Ppp2r5e	19
ENSMUSG00000047810	Ccdc88b	9		ENSMUSG00000028161	Ppp3ca	9
ENSMUSG00000021182	Ccdc88c	9		ENSMUSG00000021816	Ppp3cb	4
ENSMUSG00000044362	Ccdc89	20		ENSMUSG00000022092	Ppp3cc	20
ENSMUSG00000041375	Ccdc9	12		ENSMUSG00000033953	Ppp3r1	9
ENSMUSG00000030613	Ccdc90b	20		ENSMUSG00000028310	Ppp3r2	20
ENSMUSG00000030301	Ccdc91	6		ENSMUSG00000030697	Ppp4c	20
ENSMUSG00000037979	Ccdc92	18		ENSMUSG00000061950	Ppp4r1	20
ENSMUSG00000069814	Ccdc92b	9		ENSMUSG00000055897	Ppp4r1l-ps	9
ENSMUSG00000026339	Ccdc93	3		ENSMUSG00000052144	Ppp4r2	20
ENSMUSG00000050677	Ccdc96	20		ENSMUSG00000041846	Ppp4r3a	20
ENSMUSG00000002608	Ccdc97	20		ENSMUSG00000020463	Ppp4r3b	4
ENSMUSG00000045838	Ccdc9b	9		ENSMUSG00000021209	Ppp4r4	1
ENSMUSG00000096257	Ccer2	9		ENSMUSG00000003099	Ppp5c	7
ENSMUSG00000040312	Cchcr1	9		ENSMUSG00000026753	Ppp6c	2
ENSMUSG00000032532	Cck	19		ENSMUSG00000052296	Ppp6r1	9
ENSMUSG00000029193	Cckar	16		ENSMUSG00000036561	Ppp6r2	12
ENSMUSG00000030898	Cckbr	20		ENSMUSG00000024908	Ppp6r3	3
ENSMUSG00000035352	Ccl12	20		ENSMUSG00000055491	Pprc1	20
ENSMUSG00000031780	Ccl17	20		ENSMUSG00000028657	Ppt1	20
ENSMUSG00000023235	Ccl25	20		ENSMUSG00000015474	Ppt2	20
ENSMUSG00000095247	Ccl27a	20		ENSMUSG00000038582	Pptc7	9
ENSMUSG00000073888	Ccl27a	12		ENSMUSG00000021713	Ppwd1	20
ENSMUSG00000074715	Ccl28	20		ENSMUSG00000031157	Pqbp1	2
ENSMUSG00000000982	Ccl3	20		ENSMUSG00000034006	Pqlc1	9
ENSMUSG00000018927	Ccl6	20		ENSMUSG00000028744	Pqlc2	20
ENSMUSG00000019122	Ccl9	20		ENSMUSG00000045679	Pqlc3	20
ENSMUSG00000000378	Ccm2	1		ENSMUSG00000030008	Pradc1	9
ENSMUSG00000027474	Ccm2l	20		ENSMUSG00000031149	Praf2	12
ENSMUSG00000028195	Ccn1	1		ENSMUSG00000050271	Prag1	20
ENSMUSG00000019997	Ccn2	20		ENSMUSG00000038943	Prc1	20
ENSMUSG00000037362	Ccn3	20		ENSMUSG00000004895	Prcc	20
ENSMUSG00000005124	Ccn4	1		ENSMUSG00000075410	Prcd	20
ENSMUSG00000027715	Ccna2	20		ENSMUSG00000061119	Prcp	20

ENSMUSG00000032218	Ccnb2	20		ENSMUSG00000038151	Prdm1	20
ENSMUSG00000028252	Ccnc	20		ENSMUSG00000042496	Prdm10	9
ENSMUSG00000070348	Ccnd1	20		ENSMUSG00000075028	Prdm11	20
ENSMUSG00000000184	Ccnd2	9		ENSMUSG00000079466	Prdm12	1
ENSMUSG00000034165	Ccnd3	20		ENSMUSG00000014039	Prdm15	20
ENSMUSG00000023572	Ccndbp1	20		ENSMUSG00000039410	Prdm16	13
ENSMUSG00000002068	Ccne1	20		ENSMUSG00000085069	Prdm16os	9
ENSMUSG00000028212	Ccne2	16		ENSMUSG00000057637	Prdm2	20
ENSMUSG00000072082	Ccnf	18		ENSMUSG00000035529	Prdm4	20
ENSMUSG00000020326	Ccng1	19		ENSMUSG00000029913	Prdm5	20
ENSMUSG00000029385	Ccng2	9		ENSMUSG00000035456	Prdm8	5
ENSMUSG00000021548	Ccnh	20		ENSMUSG00000051977	Prdm9	20
ENSMUSG00000063015	Ccni	12		ENSMUSG00000028691	Prdx1	12
ENSMUSG00000025010	Ccnj	20		ENSMUSG00000118022	Prdx1-ps	20
ENSMUSG00000044707	Ccnjl	20		ENSMUSG00000005161	Prdx2	20
ENSMUSG00000021258	Ccnk	20		ENSMUSG00000082431	Prdx2-ps1	20
ENSMUSG00000027829	Ccnl1	6		ENSMUSG00000024997	Prdx3	20
ENSMUSG00000029068	Ccnl2	20		ENSMUSG00000025289	Prdx4	5
ENSMUSG00000042417	Ccno	20		ENSMUSG00000024953	Prdx5	2
ENSMUSG00000049489	Ccnq	20		ENSMUSG00000026701	Prdx6	19
ENSMUSG00000011960	Ccnt1	20		ENSMUSG00000045302	Preb	20
ENSMUSG00000026349	Ccnt2	20		ENSMUSG00000021486	Prelid1	20
ENSMUSG00000024286	Ccny	20		ENSMUSG00000024530	Prelid3a	9
ENSMUSG00000070871	Ccnyl1	20		ENSMUSG00000016257	Prelid3b	1
ENSMUSG00000033904	Ccp110	16		ENSMUSG00000041577	Prelp	13
ENSMUSG00000034563	Ccpg1	20		ENSMUSG00000019849	Prep	20
ENSMUSG00000086158	Ccpg1os	1		ENSMUSG00000024127	Prepl	9
ENSMUSG00000079227	Ccr5	20		ENSMUSG00000039621	Prex1	20
ENSMUSG00000040899	Ccr6	1		ENSMUSG00000048960	Prex2	9
ENSMUSG00000034108	Ccs	20		ENSMUSG00000006014	Prg4	19
ENSMUSG00000031971	Ccsap	18		ENSMUSG00000036158	Prickle1	10
ENSMUSG00000039578	Ccser1	20		ENSMUSG00000030020	Prickle2	1
ENSMUSG00000058690	Ccser2	3		ENSMUSG00000031145	Prickle3	20
ENSMUSG00000034024	Cct2	20		ENSMUSG00000025395	Prim1	20
ENSMUSG00000001416	Cct3	20		ENSMUSG00000026134	Prim2	20
ENSMUSG00000007739	Cct4	20		ENSMUSG00000041669	Prima1	9
ENSMUSG00000022234	Cct5	20		ENSMUSG00000038225	Primpol	18
ENSMUSG00000029447	Cct6a	8		ENSMUSG00000050697	Prkaa1	20
ENSMUSG00000020698	Cct6b	20		ENSMUSG00000028518	Prkaa2	19
ENSMUSG00000030007	Cct7	12		ENSMUSG00000029513	Prkab1	20
ENSMUSG00000025613	Cct8	20		ENSMUSG00000038205	Prkab2	1
ENSMUSG00000029617	Ccz1	16		ENSMUSG00000005469	Prkaca	9
ENSMUSG00000086564	Cd101	20		ENSMUSG00000005034	Prkacb	6
ENSMUSG00000046186	Cd109	20		ENSMUSG00000067713	Prkag1	20
ENSMUSG00000051439	Cd14	20		ENSMUSG00000028944	Prkag2	20
ENSMUSG00000025510	Cd151	20		ENSMUSG00000020612	Prkar1a	9
ENSMUSG00000008845	Cd163	20		ENSMUSG00000025855	Prkar1b	9
ENSMUSG00000019818	Cd164	20		ENSMUSG00000032601	Prkar2a	3
ENSMUSG00000028865	Cd164l2	2		ENSMUSG00000002997	Prkar2b	9
ENSMUSG00000021624	Cd180	9		ENSMUSG00000050965	Prkca	5
ENSMUSG00000028076	Cd1d1	20		ENSMUSG00000052889	Prkcb	9
ENSMUSG00000027863	Cd2	20		ENSMUSG00000021948	Prkcd	18
ENSMUSG00000022661	Cd200	9		ENSMUSG00000045038	Prkce	9
ENSMUSG00000030577	Cd22	20		ENSMUSG00000078816	Prkcg	9

ENSMUSG00000005763	Cd247	18		ENSMUSG00000021108	Prkch	20
ENSMUSG00000056481	Cd248	20		ENSMUSG00000037643	Prkci	9
ENSMUSG00000047139	Cd24a	20		ENSMUSG00000026778	Prkcq	9
ENSMUSG00000016496	Cd274	20		ENSMUSG00000003402	Prkcsh	20
ENSMUSG00000035914	Cd276	9		ENSMUSG00000029053	Prkcz	9
ENSMUSG00000061665	Cd2ap	20		ENSMUSG00000108314	Prkcz2	3
ENSMUSG00000042502	Cd2bp2	20		ENSMUSG00000002688	Prkd1	9
ENSMUSG00000034652	Cd300a	20		ENSMUSG00000041187	Prkd2	3
ENSMUSG00000044811	Cd300c2	20		ENSMUSG00000024070	Prkd3	20
ENSMUSG00000047798	Cd300lf	20		ENSMUSG00000022672	Prkdc	1
ENSMUSG00000060703	Cd302	9		ENSMUSG00000052920	Prkg1	20
ENSMUSG00000002308	Cd320	20		ENSMUSG00000029334	Prkg2	18
ENSMUSG00000004609	Cd33	20		ENSMUSG00000023826	Prkn	20
ENSMUSG00000016494	Cd34	20		ENSMUSG00000002731	Prkra	20
ENSMUSG00000002944	Cd36	20		ENSMUSG00000039737	Prkrip1	10
ENSMUSG00000030798	Cd37	20		ENSMUSG00000035725	Prkx	20
ENSMUSG00000029084	Cd38	20		ENSMUSG00000045052	Prlhr	20
ENSMUSG00000047649	Cd3eap	20		ENSMUSG00000005268	Prlr	13
ENSMUSG00000023274	Cd4	9		ENSMUSG00000109324	Prmt1	20
ENSMUSG00000005087	Cd44	9		ENSMUSG00000020230	Prmt2	17
ENSMUSG00000016493	Cd46	9		ENSMUSG00000030505	Prmt3	20
ENSMUSG00000055447	Cd47	12		ENSMUSG00000023110	Prmt5	20
ENSMUSG00000015355	Cd48	20		ENSMUSG00000049300	Prmt6	18
ENSMUSG00000000682	Cd52	9		ENSMUSG00000060098	Prmt7	20
ENSMUSG00000040747	Cd53	2		ENSMUSG00000030350	Prmt8	9
ENSMUSG00000026399	Cd55	1		ENSMUSG00000037134	Prmt9	20
ENSMUSG00000032679	Cd59a	9		ENSMUSG00000079037	Prnp	9
ENSMUSG00000068686	Cd59b	20		ENSMUSG00000073600	Prob1	20
ENSMUSG00000025351	Cd63	13		ENSMUSG00000044122	Proca1	20
ENSMUSG00000085939	Cd63-ps	13		ENSMUSG00000050558	Prokr2	20
ENSMUSG00000018774	Cd68	12		ENSMUSG00000029086	Prom1	9
ENSMUSG00000028459	Cd72	18		ENSMUSG00000027376	Prom2	20
ENSMUSG00000024610	Cd74	20		ENSMUSG00000021023	Prorp	12
ENSMUSG00000075122	Cd80	20		ENSMUSG00000032673	Prorsd1	20
ENSMUSG00000037706	Cd81	20		ENSMUSG00000022912	Pros1	20
ENSMUSG00000027215	Cd82	20		ENSMUSG00000091154	Proscos	20
ENSMUSG00000015396	Cd83	6		ENSMUSG00000049504	Proser1	20
ENSMUSG00000038147	Cd84	20		ENSMUSG00000045319	Proser2	20
ENSMUSG00000022901	Cd86	20		ENSMUSG00000036864	Proser3	20
ENSMUSG00000030342	Cd9	19		ENSMUSG00000010175	Prox1	19
ENSMUSG00000027435	Cd93	20		ENSMUSG00000079045	Prox1os	20
ENSMUSG00000035776	Cd99l2	3		ENSMUSG00000042320	Prox2	20
ENSMUSG00000028755	Cda	1		ENSMUSG00000093629	Prox2os	20
ENSMUSG00000021982	Cdadc1	20		ENSMUSG00000039449	Prpf18	20
ENSMUSG00000027284	Cdan1	20		ENSMUSG00000024735	Prpf19	2
ENSMUSG00000039128	Cdc123	12		ENSMUSG00000015748	Prpf3	9
ENSMUSG00000033502	Cdc14a	20		ENSMUSG00000008373	Prpf31	20
ENSMUSG00000033102	Cdc14b	1		ENSMUSG00000063800	Prpf38a	20
ENSMUSG00000038416	Cdc16	1		ENSMUSG00000027881	Prpf38b	20
ENSMUSG00000006398	Cdc20	20		ENSMUSG00000035597	Prpf39	20
ENSMUSG00000024370	Cdc23	1		ENSMUSG00000066148	Prpf4	20
ENSMUSG00000032477	Cdc25a	18		ENSMUSG00000061136	Prpf40a	20
ENSMUSG00000027330	Cdc25b	1		ENSMUSG00000023007	Prpf40b	20
ENSMUSG00000044201	Cdc25c	2		ENSMUSG00000021413	Prpf4b	20

ENSMUSG00000066149	Cdc26	20		ENSMUSG00000002455	Prpf6	20
ENSMUSG00000020687	Cdc27	20		ENSMUSG00000020850	Prpf8	20
ENSMUSG00000020307	Cdc34	3		ENSMUSG00000023484	Prph	19
ENSMUSG00000020870	Cdc34b	1		ENSMUSG00000031432	Prps1	20
ENSMUSG00000019471	Cdc37	20		ENSMUSG00000079104	Prps1l3	20
ENSMUSG00000024780	Cdc37l1	20		ENSMUSG00000025742	Prps2	20
ENSMUSG00000038446	Cdc40	9		ENSMUSG00000015869	Prpsap1	20
ENSMUSG00000006699	Cdc42	20		ENSMUSG00000020528	Prpsap2	18
ENSMUSG00000026490	Cdc42bpa	12		ENSMUSG00000020493	Prr11	20
ENSMUSG00000021279	Cdc42bpb	20		ENSMUSG00000046574	Prr12	20
ENSMUSG00000049521	Cdc42ep1	20		ENSMUSG00000023048	Prr13	18
ENSMUSG00000045664	Cdc42ep2	20		ENSMUSG00000030822	Prr14	20
ENSMUSG00000036533	Cdc42ep3	9		ENSMUSG00000054280	Prr14l	7
ENSMUSG00000041598	Cdc42ep4	18		ENSMUSG00000073565	Prr16	9
ENSMUSG00000046722	Cdc42se1	20		ENSMUSG00000055945	Prr18	16
ENSMUSG00000052298	Cdc42se2	4		ENSMUSG00000090273	Prr22	18
ENSMUSG00000000028	Cdc45	20		ENSMUSG00000038500	Prr3	20
ENSMUSG00000023932	Cdc5l	20		ENSMUSG00000037086	Prr32	13
ENSMUSG00000017499	Cdc6	20		ENSMUSG00000064125	Prr36	20
ENSMUSG00000029283	Cdc7	20		ENSMUSG00000036106	Prr5	20
ENSMUSG00000026361	Cdc73	2		ENSMUSG00000032841	Prr5l	20
ENSMUSG00000048922	Cdca2	20		ENSMUSG00000034686	Prr7	18
ENSMUSG00000023505	Cdca3	20		ENSMUSG00000024594	Prrc1	9
ENSMUSG00000047832	Cdca4	20		ENSMUSG00000024393	Prrc2a	20
ENSMUSG00000055612	Cdca7	20		ENSMUSG00000039262	Prrc2b	20
ENSMUSG00000021175	Cdca7l	9		ENSMUSG00000040225	Prrc2c	2
ENSMUSG00000028873	Cdca8	20		ENSMUSG00000047996	Prrg1	3
ENSMUSG00000035498	Cdcp1	20		ENSMUSG00000007837	Prrg2	20
ENSMUSG00000000303	Cdh1	20		ENSMUSG00000033361	Prrg3	20
ENSMUSG00000022321	Cdh10	2		ENSMUSG00000027171	Prrg4	12
ENSMUSG00000031673	Cdh11	12		ENSMUSG00000015476	Prrt1	9
ENSMUSG00000040452	Cdh12	3		ENSMUSG00000045114	Prrt2	20
ENSMUSG00000031841	Cdh13	1		ENSMUSG00000045009	Prrt3	20
ENSMUSG00000031962	Cdh15	20		ENSMUSG00000079654	Prrt4	1
ENSMUSG00000040420	Cdh18	3		ENSMUSG00000026586	Prrx1	20
ENSMUSG00000047216	Cdh19	9		ENSMUSG00000041730	Prrxl1	20
ENSMUSG00000024304	Cdh2	19		ENSMUSG00000027978	Prss12	20
ENSMUSG00000050840	Cdh20	3		ENSMUSG00000006179	Prss16	1
ENSMUSG00000053166	Cdh22	6		ENSMUSG00000039405	Prss23	18
ENSMUSG00000012819	Cdh23	9		ENSMUSG00000033491	Prss35	20
ENSMUSG00000059674	Cdh24	6		ENSMUSG00000070371	Prss36	9
ENSMUSG00000061048	Cdh3	13		ENSMUSG00000024114	Prss41	20
ENSMUSG00000000305	Cdh4	1		ENSMUSG00000048752	Prss50	20
ENSMUSG00000031871	Cdh5	18		ENSMUSG00000044139	Prss53	20
ENSMUSG00000039385	Cdh6	18		ENSMUSG00000036480	Prss56	20
ENSMUSG00000026312	Cdh7	20		ENSMUSG00000036030	Prtg	20
ENSMUSG00000036510	Cdh8	7		ENSMUSG00000057729	Prtn3	20
ENSMUSG00000025370	Cdh9	9		ENSMUSG00000015711	Prune1	13
ENSMUSG00000021803	Cdhr1	9		ENSMUSG00000039126	Prune2	18
ENSMUSG00000035860	Cdhr3	20		ENSMUSG00000053198	Prx	20
ENSMUSG00000032595	Cdhr4	14		ENSMUSG00000021792	Prxl2a	20
ENSMUSG00000004071	Cdip1	20		ENSMUSG00000029059	Prxl2b	2
ENSMUSG00000030682	Cdipt	20		ENSMUSG00000021482	Prxl2c	20
ENSMUSG00000033862	Cdk10	20		ENSMUSG00000004207	Psap	20

ENSMUSG00000029062	Cdk11b	20		ENSMUSG00000024640	Psat1	15
ENSMUSG00000003119	Cdk12	2		ENSMUSG00000037126	Psd	9
ENSMUSG00000041297	Cdk13	20		ENSMUSG00000024347	Psd2	13
ENSMUSG00000028926	Cdk14	19		ENSMUSG00000030465	Psd3	4
ENSMUSG00000026023	Cdk15	1		ENSMUSG00000026979	Psd4	20
ENSMUSG00000031065	Cdk16	18		ENSMUSG00000019969	Psen1	20
ENSMUSG00000020015	Cdk17	9		ENSMUSG00000010609	Psen2	20
ENSMUSG00000026437	Cdk18	18		ENSMUSG00000036835	Psenen	19
ENSMUSG00000038481	Cdk19	9		ENSMUSG00000066760	Psg16	9
ENSMUSG00000025358	Cdk2	20		ENSMUSG00000028484	Psip1	9
ENSMUSG00000021483	Cdk20	20		ENSMUSG00000048310	Pskh1	20
ENSMUSG00000029394	Cdk2ap1	1		ENSMUSG00000030751	Psma1	20
ENSMUSG00000092300	Cdk3	20		ENSMUSG00000015671	Psma2	20
ENSMUSG00000006728	Cdk4	3		ENSMUSG00000060073	Psma3	20
ENSMUSG00000028969	Cdk5	2		ENSMUSG00000032301	Psma4	12
ENSMUSG00000048895	Cdk5r1	20		ENSMUSG00000068749	Psma5	20
ENSMUSG00000090071	Cdk5r2	9		ENSMUSG00000021024	Psma6	12
ENSMUSG00000027487	Cdk5rap1	13		ENSMUSG00000027566	Psma7	12
ENSMUSG00000039298	Cdk5rap2	15		ENSMUSG00000014769	Psmb1	12
ENSMUSG00000018669	Cdk5rap3	2		ENSMUSG00000031897	Psmb10	20
ENSMUSG00000040274	Cdk6	20		ENSMUSG00000028837	Psmb2	10
ENSMUSG00000069089	Cdk7	1		ENSMUSG00000069744	Psmb3	12
ENSMUSG00000029635	Cdk8	9		ENSMUSG00000005779	Psmb4	12
ENSMUSG00000009555	Cdk9	20		ENSMUSG00000022193	Psmb5	12
ENSMUSG00000006191	Cdkal1	20		ENSMUSG00000018286	Psmb6	12
ENSMUSG00000020990	Cdkl1	19		ENSMUSG00000026750	Psmb7	20
ENSMUSG00000029403	Cdkl2	1		ENSMUSG00000024338	Psmb8	20
ENSMUSG00000020389	Cdkl3	20		ENSMUSG00000096727	Psmb9	10
ENSMUSG00000033966	Cdkl4	18		ENSMUSG00000021178	Psmc1	20
ENSMUSG00000031292	Cdkl5	20		ENSMUSG00000028932	Psmc2	1
ENSMUSG00000023067	Cdkn1a	10		ENSMUSG00000002102	Psmc3	7
ENSMUSG00000003031	Cdkn1b	18		ENSMUSG00000019303	Psmc3ip	20
ENSMUSG00000037664	Cdkn1c	13		ENSMUSG00000030603	Psmc4	12
ENSMUSG00000044303	Cdkn2a	20		ENSMUSG00000020708	Psmc5	20
ENSMUSG00000038069	Cdkn2aip	20		ENSMUSG00000021832	Psmc6	20
ENSMUSG00000020392	Cdkn2aipnl	20		ENSMUSG00000026229	Psmd1	1
ENSMUSG00000028551	Cdkn2c	20		ENSMUSG00000017428	Psmd11	18
ENSMUSG00000096472	Cdkn2d	20		ENSMUSG00000020720	Psmd12	20
ENSMUSG00000039496	Cdnf	20		ENSMUSG00000025487	Psmd13	5
ENSMUSG00000033022	Cdo1	18		ENSMUSG00000026914	Psmd14	16
ENSMUSG00000038119	Cdon	18		ENSMUSG00000006998	Psmd2	20
ENSMUSG00000064284	Cdpf1	19		ENSMUSG00000017221	Psmd3	12
ENSMUSG00000090546	Cdr1	18		ENSMUSG00000005625	Psmd4	12
ENSMUSG00000071753	Cdr1os	18		ENSMUSG00000026869	Psmd5	1
ENSMUSG00000030878	Cdr2	16		ENSMUSG00000021737	Psmd6	18
ENSMUSG00000050910	Cdr2l	9		ENSMUSG00000039067	Psmd7	20
ENSMUSG00000029330	Cds1	18		ENSMUSG00000030591	Psmd8	20
ENSMUSG00000058793	Cds2	18		ENSMUSG00000029440	Psmd9	3
ENSMUSG00000006585	Cdt1	20		ENSMUSG00000022216	Psme1	20
ENSMUSG00000032803	Cdv3	9		ENSMUSG00000079197	Psme2	12
ENSMUSG00000090389	Cdv3-ps	20		ENSMUSG00000078652	Psme3	12
ENSMUSG00000059288	Cdyl	4		ENSMUSG00000040850	Psme4	11
ENSMUSG00000031758	Cdyl2	20		ENSMUSG00000032869	Psmf1	20
ENSMUSG00000074272	Ceacam1	20		ENSMUSG00000022913	Psmg1	20

ENSMUSG00000054385	Ceacam2	20		ENSMUSG00000024537	Psmg2	12
ENSMUSG00000034957	Cebpa	20		ENSMUSG00000029551	Psmg3	12
ENSMUSG00000056501	Cebpb	20		ENSMUSG00000071451	Psmg4	19
ENSMUSG00000071637	Cebpd	20		ENSMUSG00000021938	Pspc1	20
ENSMUSG00000056216	Cebpg	10		ENSMUSG00000029446	Psph	20
ENSMUSG00000024081	Cebpz	20		ENSMUSG00000068744	Psrc1	20
ENSMUSG00000062691	Cebpzos	12		ENSMUSG00000063179	Pstk	20
ENSMUSG00000071226	Cecr2	5		ENSMUSG00000032322	Pstpip1	9
ENSMUSG00000023031	Cela1	20		ENSMUSG00000056529	Ptafr	20
ENSMUSG00000002107	Celf2	9		ENSMUSG00000074925	Ptar1	3
ENSMUSG00000028137	Celf3	9		ENSMUSG00000006498	Ptbp1	20
ENSMUSG00000024268	Celf4	16		ENSMUSG00000028134	Ptbp2	1
ENSMUSG00000034818	Celf5	18		ENSMUSG00000028382	Ptbp3	7
ENSMUSG00000032297	Celf6	20		ENSMUSG00000029624	Ptcd1	1
ENSMUSG00000097881	Celrr	20		ENSMUSG00000021650	Ptcd2	20
ENSMUSG00000016028	Celsr1	20		ENSMUSG00000063884	Ptcd3	20
ENSMUSG00000068740	Celsr2	10		ENSMUSG00000021466	Ptch1	20
ENSMUSG00000052353	Cemip	5		ENSMUSG00000028681	Ptch2	1
ENSMUSG00000024754	Cemip2	20		ENSMUSG00000041552	Ptchd1	9
ENSMUSG00000060240	Cend1	9		ENSMUSG00000042256	Ptchd4	2
ENSMUSG00000029177	Cenpa	20		ENSMUSG00000021518	Ptdss1	20
ENSMUSG00000068267	Cenpb	20		ENSMUSG00000025495	Ptdss2	20
ENSMUSG00000029253	Cenpc1	20		ENSMUSG00000013663	Pten	2
ENSMUSG00000045328	Cenpe	20		ENSMUSG00000026730	Pter	20
ENSMUSG00000026605	Cenpf	20		ENSMUSG00000015090	Ptgds	4
ENSMUSG00000045273	Cenph	20		ENSMUSG00000019464	Ptger1	20
ENSMUSG00000031262	Cenpi	3		ENSMUSG00000040016	Ptger3	9
ENSMUSG00000064128	Cenpj	20		ENSMUSG00000039942	Ptger4	20
ENSMUSG00000021714	Cenpk	20		ENSMUSG00000050737	Ptges	12
ENSMUSG00000026708	Cenpl	20		ENSMUSG00000026820	Ptges2	20
ENSMUSG00000068101	Cenpm	20		ENSMUSG00000071072	Ptges3	20
ENSMUSG00000031756	Cenpn	20		ENSMUSG00000040078	Ptges3-ps	20
ENSMUSG00000020652	Cenpo	20		ENSMUSG00000097487	Ptges3l	9
ENSMUSG00000021391	Cenpp	20		ENSMUSG00000028036	Ptgfr	20
ENSMUSG00000023919	Cenpq	9		ENSMUSG00000027864	Ptgfrn	20
ENSMUSG00000073705	Cenps	20		ENSMUSG00000017969	Ptgis	20
ENSMUSG00000036672	Cenpt	20		ENSMUSG00000028378	Ptgr1	20
ENSMUSG00000031629	Cenpu	20		ENSMUSG00000072946	Ptgr2	1
ENSMUSG00000018509	Cenpv	20		ENSMUSG00000047250	Ptgs1	20
ENSMUSG00000075266	Cenpw	12		ENSMUSG00000032487	Ptgs2	5
ENSMUSG00000025144	Cenpx	20		ENSMUSG00000032492	Pth1r	9
ENSMUSG00000039523	Cep104	20		ENSMUSG00000025946	Pth2r	20
ENSMUSG00000020728	Cep112	1		ENSMUSG00000048776	Pthlh	9
ENSMUSG00000085811	Cep112it	19		ENSMUSG00000022607	Ptk2	20
ENSMUSG00000048799	Cep120	1		ENSMUSG00000059456	Ptk2b	9
ENSMUSG00000040729	Cep126	9		ENSMUSG00000023972	Ptk7	9
ENSMUSG00000061533	Cep128	1		ENSMUSG00000026238	Ptma	1
ENSMUSG00000039781	Cep131	6		ENSMUSG00000030122	Ptms	3
ENSMUSG00000036403	Cep135	20		ENSMUSG00000029838	Ptn	3
ENSMUSG00000068394	Cep152	20		ENSMUSG00000038502	Ptov1	3
ENSMUSG00000056919	Cep162	1		ENSMUSG00000117310	Ptp4a1	20
ENSMUSG00000043987	Cep164	1		ENSMUSG00000026064	Ptp4a1	20
ENSMUSG00000057335	Cep170	3		ENSMUSG00000028788	Ptp4a2	8
ENSMUSG00000072825	Cep170b	20		ENSMUSG00000059895	Ptp4a3	20

ENSMUSG00000035790	Cep19	9		ENSMUSG00000039515	Ptpa	9
ENSMUSG00000024542	Cep192	20		ENSMUSG00000038042	Ptpdc1	1
ENSMUSG00000038241	Cep250	20		ENSMUSG00000027540	Ptpn1	1
ENSMUSG00000019971	Cep290	16		ENSMUSG00000043733	Ptpn11	8
ENSMUSG00000046111	Cep295	9		ENSMUSG00000028771	Ptpn12	20
ENSMUSG00000076433	Cep295nl	3		ENSMUSG00000034573	Ptpn13	20
ENSMUSG00000033671	Cep350	19		ENSMUSG00000026604	Ptpn14	13
ENSMUSG00000029790	Cep41	20		ENSMUSG00000026126	Ptpn18	20
ENSMUSG00000038215	Cep44	20		ENSMUSG00000024539	Ptpn2	20
ENSMUSG00000024989	Cep55	20		ENSMUSG00000021009	Ptpn21	9
ENSMUSG00000031922	Cep57	20		ENSMUSG00000027843	Ptpn22	20
ENSMUSG00000019813	Cep57l1	18		ENSMUSG00000036057	Ptpn23	9
ENSMUSG00000032534	Cep63	9		ENSMUSG00000038764	Ptpn3	18
ENSMUSG00000044066	Cep68	20		ENSMUSG00000026384	Ptpn4	18
ENSMUSG00000056267	Cep70	20		ENSMUSG00000030854	Ptpn5	9
ENSMUSG00000021572	Cep72	20		ENSMUSG00000004266	Ptpn6	20
ENSMUSG00000073542	Cep76	19		ENSMUSG00000031506	Ptpn7	1
ENSMUSG00000041491	Cep78	20		ENSMUSG00000032290	Ptpn9	20
ENSMUSG00000020024	Cep83	9		ENSMUSG00000027303	Ptpra	20
ENSMUSG00000097164	Cep83os	20		ENSMUSG00000020154	Ptprb	20
ENSMUSG00000037443	Cep85	20		ENSMUSG00000026395	Ptprc	20
ENSMUSG00000038594	Cep85l	19		ENSMUSG00000028399	Ptprd	16
ENSMUSG00000023072	Cep89	18		ENSMUSG00000041836	Ptpre	9
ENSMUSG00000018372	Cep95	20		ENSMUSG00000033295	Ptprf	20
ENSMUSG00000022604	Cep97	20		ENSMUSG00000021745	Ptprg	5
ENSMUSG00000040774	Cept1	20		ENSMUSG00000035429	Ptprh	20
ENSMUSG00000039787	Cercam	20		ENSMUSG00000025314	Ptprj	20
ENSMUSG00000035891	Cerk	18		ENSMUSG00000019889	Ptprk	20
ENSMUSG00000075256	Cerkl	20		ENSMUSG00000033278	Ptprm	12
ENSMUSG00000087408	Cers1	9		ENSMUSG00000026204	Ptprn	6
ENSMUSG00000015714	Cers2	15		ENSMUSG00000056553	Ptprn2	9
ENSMUSG00000008206	Cers4	9		ENSMUSG00000030223	Ptpro	4
ENSMUSG00000023021	Cers5	1		ENSMUSG00000020151	Ptprr	20
ENSMUSG00000027035	Cers6	4		ENSMUSG00000013236	Ptprs	1
ENSMUSG00000058019	Ces5a	20		ENSMUSG00000053141	Ptprt	20
ENSMUSG00000031347	Cetn2	20		ENSMUSG00000028909	Ptpru	17
ENSMUSG00000021537	Cetn3	12		ENSMUSG00000097993	Ptprv	9
ENSMUSG00000045031	Cetn4	20		ENSMUSG00000068748	Ptprz1	19
ENSMUSG00000048794	Cfap100	20		ENSMUSG00000053746	Ptrh1	20
ENSMUSG00000026649	Cfap126	20		ENSMUSG00000072582	Ptrh2	20
ENSMUSG00000038987	Cfap157	20		ENSMUSG00000096199	Ptrhd1	3
ENSMUSG00000011154	Cfap161	14		ENSMUSG00000032067	Pts	12
ENSMUSG00000031796	Cfap20	3		ENSMUSG00000020415	Pttg1	20
ENSMUSG00000028294	Cfap206	14		ENSMUSG00000009291	Pttg1ip	3
ENSMUSG00000036962	Cfap221	20		ENSMUSG00000002524	Puf60	3
ENSMUSG00000022972	Cfap298	20		ENSMUSG00000028580	Pum1	7
ENSMUSG00000053070	Cfap300	14		ENSMUSG00000020594	Pum2	7
ENSMUSG00000020462	Cfap36	12		ENSMUSG00000041360	Pum3	12
ENSMUSG00000020284	Cfap410	20		ENSMUSG00000043991	Pura	3
ENSMUSG00000044948	Cfap43	14		ENSMUSG00000094483	Purb	12
ENSMUSG00000071550	Cfap44	14		ENSMUSG00000049184	Purg	19
ENSMUSG00000026546	Cfap45	13		ENSMUSG00000029507	Pus1	9
ENSMUSG00000049571	Cfap46	1		ENSMUSG00000020280	Pus10	20
ENSMUSG00000073077	Cfap47	13		ENSMUSG00000032103	Pus3	20

ENSMUSG00000020904	Cfap52	14		ENSMUSG00000057541	Pus7	20
ENSMUSG00000035394	Cfap53	20		ENSMUSG00000033356	Pus7l	20
ENSMUSG00000020014	Cfap54	14		ENSMUSG00000051557	Pusl1	20
ENSMUSG00000028730	Cfap57	20		ENSMUSG00000005716	Pvalb	16
ENSMUSG00000037143	Cfap61	14		ENSMUSG00000040511	Pvr	1
ENSMUSG00000047021	Cfap65	14		ENSMUSG00000097039	Pvt1	20
ENSMUSG00000040473	Cfap69	20		ENSMUSG00000001785	Pwp1	9
ENSMUSG00000039543	Cfap70	13		ENSMUSG00000032834	Pwp2	20
ENSMUSG00000094282	Cfap73	14		ENSMUSG00000044950	Pwwp2a	7
ENSMUSG00000078490	Cfap74	1		ENSMUSG00000020156	Pwwp3a	20
ENSMUSG00000079502	Cfap77	14		ENSMUSG00000042515	Pwwp3b	20
ENSMUSG00000031631	Cfap97	9		ENSMUSG00000021411	Pxdc1	20
ENSMUSG00000090336	Cfap97d2	13		ENSMUSG00000020674	Pxdn	9
ENSMUSG00000109572	Cfap99	12		ENSMUSG00000033885	Pxk	9
ENSMUSG00000031954	Cfdp1	20		ENSMUSG00000029499	Pxmp2	2
ENSMUSG00000026365	Cfh	20		ENSMUSG00000000876	Pxmp4	20
ENSMUSG00000056201	Cfl1	19		ENSMUSG00000029528	Pxn	20
ENSMUSG00000062929	Cfl2	20		ENSMUSG00000043587	Pxylp1	20
ENSMUSG00000026031	Cflar	1		ENSMUSG00000030793	Pycard	20
ENSMUSG00000001128	Cfp	20		ENSMUSG00000025140	Pycr1	18
ENSMUSG00000041301	Cftr	20		ENSMUSG00000026520	Pycr2	12
ENSMUSG00000032344	Cgas	20		ENSMUSG00000022571	Pycrl	20
ENSMUSG00000054604	Cggbp1	3		ENSMUSG00000033059	Pygb	20
ENSMUSG00000068876	Cgn	17		ENSMUSG00000021069	Pygl	20
ENSMUSG00000032232	Cgnl1	13		ENSMUSG00000034910	Pygo1	19
ENSMUSG00000029161	Cgref1	12		ENSMUSG00000047824	Pygo2	12
ENSMUSG00000055128	Cgrrf1	3		ENSMUSG00000064030	Pym1	20
ENSMUSG00000027313	Chac1	20		ENSMUSG00000041671	Pyroxd1	20
ENSMUSG00000020309	Chac2	20		ENSMUSG00000060224	Pyroxd2	1
ENSMUSG00000063765	Chadl	7		ENSMUSG00000043162	Pyurf	20
ENSMUSG00000002835	Chaf1a	20		ENSMUSG00000032604	Qars	20
ENSMUSG00000047710	Champ1	20		ENSMUSG00000015806	Qdpr	16
ENSMUSG00000021919	Chat	3		ENSMUSG00000062078	Qk	16
ENSMUSG00000063787	Chchd1	12		ENSMUSG00000024084	Qpct	20
ENSMUSG00000049422	Chchd10	19		ENSMUSG00000030407	Qpctl	20
ENSMUSG00000070493	Chchd2	12		ENSMUSG00000030674	Qprt	20
ENSMUSG00000094320	Chchd2-ps	20		ENSMUSG00000058400	Qrfpr	20
ENSMUSG00000053768	Chchd3	10		ENSMUSG00000029917	Qrfprl	9
ENSMUSG00000034203	Chchd4	2		ENSMUSG00000006673	Qrich1	9
ENSMUSG00000037938	Chchd5	20		ENSMUSG00000019863	Qrsl1	3
ENSMUSG00000030086	Chchd6	12		ENSMUSG00000033684	Qsox1	7
ENSMUSG00000042198	Chchd7	12		ENSMUSG00000036327	Qsox2	12
ENSMUSG00000023852	Chd1	20		ENSMUSG00000002825	Qtrt1	20
ENSMUSG00000028089	Chd1l	9		ENSMUSG00000022704	Qtrt2	20
ENSMUSG00000078671	Chd2	20		ENSMUSG00000034194	R3hcc1	20
ENSMUSG00000018474	Chd3	1		ENSMUSG00000025184	R3hcc1l	20
ENSMUSG00000063870	Chd4	9		ENSMUSG00000056211	R3hdm1	9
ENSMUSG00000005045	Chd5	9		ENSMUSG00000025404	R3hdm2	3
ENSMUSG00000057133	Chd6	2		ENSMUSG00000035781	R3hdm4	18
ENSMUSG00000041235	Chd7	18		ENSMUSG00000059277	R74862	20
ENSMUSG00000053754	Chd8	12		ENSMUSG00000020671	Rab10	3
ENSMUSG00000056608	Chd9	2		ENSMUSG00000079179	Rab10os	20
ENSMUSG00000015970	Chdh	1		ENSMUSG00000004771	Rab11a	3
ENSMUSG00000032113	Chek1	20		ENSMUSG00000077450	Rab11b	11

ENSMUSG00000029521	Chek2	20		ENSMUSG00000095690	Rab11b-ps2	20
ENSMUSG00000052488	Cherp	20		ENSMUSG00000031488	Rab11fip1	13
ENSMUSG00000014668	Chfr	20		ENSMUSG00000040022	Rab11fip2	4
ENSMUSG00000021194	Chga	18		ENSMUSG00000037098	Rab11fip3	20
ENSMUSG00000027350	Chgb	5		ENSMUSG00000017639	Rab11fip4	20
ENSMUSG00000062778	Chia1	9		ENSMUSG00000051343	Rab11fip5	18
ENSMUSG00000031327	Chic1	20		ENSMUSG00000023460	Rab12	20
ENSMUSG00000029229	Chic2	20		ENSMUSG00000027935	Rab13	20
ENSMUSG00000025512	Chid1	3		ENSMUSG00000026878	Rab14	2
ENSMUSG00000064246	Chil1	20		ENSMUSG00000021062	Rab15	9
ENSMUSG00000026450	Chit1	20		ENSMUSG00000073639	Rab18	1
ENSMUSG00000024843	Chka	20		ENSMUSG00000020149	Rab1a	9
ENSMUSG00000022617	Chkb	12		ENSMUSG00000024870	Rab1b	12
ENSMUSG00000030077	Chl1	1		ENSMUSG00000031504	Rab20	13
ENSMUSG00000025531	Chm	1		ENSMUSG00000020132	Rab21	1
ENSMUSG00000078185	Chml	18		ENSMUSG00000027519	Rab22a	20
ENSMUSG00000000743	Chmp1a	3		ENSMUSG00000004768	Rab23	3
ENSMUSG00000109901	Chmp1b	9		ENSMUSG00000034789	Rab24	20
ENSMUSG00000033916	Chmp2a	19		ENSMUSG00000079657	Rab26	18
ENSMUSG00000004843	Chmp2b	20		ENSMUSG00000093565	Rab26os	20
ENSMUSG00000053119	Chmp3	18		ENSMUSG00000032202	Rab27a	2
ENSMUSG00000038467	Chmp4b	12		ENSMUSG00000024511	Rab27b	16
ENSMUSG00000028419	Chmp5	12		ENSMUSG00000029128	Rab28	20
ENSMUSG00000025371	Chmp6	20		ENSMUSG00000026433	Rab29	18
ENSMUSG00000034190	Chmp7	11		ENSMUSG00000047187	Rab2a	20
ENSMUSG00000056486	Chn1	9		ENSMUSG00000022159	Rab2b	9
ENSMUSG00000085838	Chn1os1	9		ENSMUSG00000030643	Rab30	20
ENSMUSG00000086544	Chn1os3	9		ENSMUSG00000056515	Rab31	20
ENSMUSG00000004633	Chn2	20		ENSMUSG00000019832	Rab32	20
ENSMUSG00000022860	Chodl	20		ENSMUSG00000031104	Rab33a	20
ENSMUSG00000001774	Chordc1	20		ENSMUSG00000027739	Rab33b	12
ENSMUSG00000014077	Chp1	19		ENSMUSG00000002059	Rab34	18
ENSMUSG00000030865	Chp2	20		ENSMUSG00000029518	Rab35	20
ENSMUSG00000032997	Chpf	20		ENSMUSG00000020175	Rab36	20
ENSMUSG00000038181	Chpf2	1		ENSMUSG00000020732	Rab37	18
ENSMUSG00000060002	Chpt1	3		ENSMUSG00000030559	Rab38	20
ENSMUSG00000068391	Chrac1	12		ENSMUSG00000055069	Rab39	20
ENSMUSG00000006958	Chrd	5		ENSMUSG00000031202	Rab39b	1
ENSMUSG00000031283	Chrdl1	19		ENSMUSG00000003411	Rab3b	3
ENSMUSG00000030732	Chrdl2	20		ENSMUSG00000021700	Rab3c	18
ENSMUSG00000032773	Chrm1	18		ENSMUSG00000019066	Rab3d	15
ENSMUSG00000045613	Chrm2	18		ENSMUSG00000036104	Rab3gap1	12
ENSMUSG00000046159	Chrm3	5		ENSMUSG00000039318	Rab3gap2	20
ENSMUSG00000040495	Chrm4	9		ENSMUSG00000024663	Rab3il1	20
ENSMUSG00000074939	Chrm5	20		ENSMUSG00000064181	Rab3ip	20
ENSMUSG00000086382	Chrna1os	20		ENSMUSG00000025170	Rab40b	3
ENSMUSG00000022041	Chrna2	18		ENSMUSG00000025730	Rab40c	20
ENSMUSG00000032303	Chrna3	9		ENSMUSG00000089687	Rab42	20
ENSMUSG00000027577	Chrna4	18		ENSMUSG00000030055	Rab43	20
ENSMUSG00000035594	Chrna5	2		ENSMUSG00000064147	Rab44	20
ENSMUSG00000030525	Chrna7	20		ENSMUSG00000019478	Rab4a	10
ENSMUSG00000041189	Chrnb1	20		ENSMUSG00000053291	Rab4b	20
ENSMUSG00000027950	Chrnb2	9		ENSMUSG00000017831	Rab5a	20
ENSMUSG00000035200	Chrnb4	20		ENSMUSG00000000711	Rab5b	20

ENSMUSG00000027221	Chst1	9		ENSMUSG00000019173	Rab5c	2
ENSMUSG00000026080	Chst10	20		ENSMUSG00000027637	Rab5if	19
ENSMUSG00000034612	Chst11	9		ENSMUSG00000030704	Rab6a	19
ENSMUSG00000036599	Chst12	20		ENSMUSG00000032549	Rab6b	1
ENSMUSG00000074916	Chst14	20		ENSMUSG00000079477	Rab7	20
ENSMUSG00000030930	Chst15	9		ENSMUSG00000111686	Rab7-ps1	20
ENSMUSG00000033350	Chst2	9		ENSMUSG00000052688	Rab7b	20
ENSMUSG00000057337	Chst3	20		ENSMUSG00000003037	Rab8a	20
ENSMUSG00000031952	Chst5	20		ENSMUSG00000036943	Rab8b	20
ENSMUSG00000037347	Chst7	20		ENSMUSG00000079316	Rab9	20
ENSMUSG00000060402	Chst8	18		ENSMUSG00000043463	Rab9b	16
ENSMUSG00000047161	Chst9	5		ENSMUSG00000003380	Rabac1	12
ENSMUSG00000032640	Chsy1	19		ENSMUSG00000020817	Rabep1	20
ENSMUSG00000058152	Chsy3	9		ENSMUSG00000030727	Rabep2	6
ENSMUSG00000019214	Chtf18	20		ENSMUSG00000070953	Rabepk	20
ENSMUSG00000046691	Chtf8	9		ENSMUSG00000035437	Rabgap1	19
ENSMUSG00000001017	Chtop	9		ENSMUSG00000026721	Rabgap1l	5
ENSMUSG00000025199	Chuk	1		ENSMUSG00000025340	Rabgef1	20
ENSMUSG00000090258	Churc1	20		ENSMUSG00000040472	Rabggta	20
ENSMUSG00000003662	Ciao1	20		ENSMUSG00000038975	Rabggtb	20
ENSMUSG00000032381	Ciao2a	20		ENSMUSG00000042229	Rabif	9
ENSMUSG00000031879	Ciao2b	19		ENSMUSG00000022621	Rabl2	20
ENSMUSG00000002280	Ciao3	3		ENSMUSG00000022827	Rabl3	20
ENSMUSG00000031781	Ciapin1	20		ENSMUSG00000015087	Rabl6	3
ENSMUSG00000038550	Ciart	1		ENSMUSG00000001847	Rac1	20
ENSMUSG00000030538	Cib1	19		ENSMUSG00000033220	Rac2	20
ENSMUSG00000037493	Cib2	6		ENSMUSG00000018012	Rac3	2
ENSMUSG00000005442	Cic	20		ENSMUSG00000023015	Racgap1	20
ENSMUSG00000024526	Cidea	20		ENSMUSG00000020372	Rack1	20
ENSMUSG00000022219	Cideb	20		ENSMUSG00000022248	Rad1	1
ENSMUSG00000021276	Cinp	18		ENSMUSG00000021635	Rad17	20
ENSMUSG00000033031	Cip2a	20		ENSMUSG00000030254	Rad18	20
ENSMUSG00000034157	Cipc	1		ENSMUSG00000022314	Rad21	20
ENSMUSG00000041777	Cir1	9		ENSMUSG00000074704	Rad21l	20
ENSMUSG00000045193	Cirbp	20		ENSMUSG00000003813	Rad23a	20
ENSMUSG00000037710	Cisd1	12		ENSMUSG00000028426	Rad23b	20
ENSMUSG00000028165	Cisd2	20		ENSMUSG00000020380	Rad50	20
ENSMUSG00000078695	Cisd3	20		ENSMUSG00000027323	Rad51	20
ENSMUSG00000032578	Cish	20		ENSMUSG00000030346	Rad51ap1	20
ENSMUSG00000029516	Cit	18		ENSMUSG00000086022	Rad51ap2	19
ENSMUSG00000051159	Cited1	18		ENSMUSG00000059060	Rad51b	20
ENSMUSG00000039910	Cited2	9		ENSMUSG00000007646	Rad51c	20
ENSMUSG00000070803	Cited4	9		ENSMUSG00000018841	Rad51d	20
ENSMUSG00000039205	Ciz1	20		ENSMUSG00000030166	Rad52	2
ENSMUSG00000028813	CK137956	20		ENSMUSG00000078773	Rad54b	20
ENSMUSG00000037725	Ckap2	20		ENSMUSG00000028702	Rad54l	20
ENSMUSG00000048327	Ckap2l	20		ENSMUSG00000040661	Rad54l2	20
ENSMUSG00000046841	Ckap4	19		ENSMUSG00000038569	Rad9b	20
ENSMUSG00000040549	Ckap5	12		ENSMUSG00000029576	Radil	9
ENSMUSG00000001270	Ckb	2		ENSMUSG00000027509	Rae1	20
ENSMUSG00000054400	Cklf	20		ENSMUSG00000000441	Raf1	20
ENSMUSG00000000308	Ckmt1	6		ENSMUSG00000061311	Rag1	1
ENSMUSG00000028044	Cks1b	20		ENSMUSG00000062115	Rai1	9
ENSMUSG00000062248	Cks2	18		ENSMUSG00000022246	Rai14	20

ENSMUSG00000064302	Clasp1	19		ENSMUSG00000043518	Rai2	20
ENSMUSG00000033392	Clasp2	18		ENSMUSG00000008859	Rala	20
ENSMUSG00000061028	Clasrp	7		ENSMUSG00000004451	Ralb	20
ENSMUSG00000037594	Clba1	20		ENSMUSG00000024096	Ralbp1	20
ENSMUSG00000027884	Clcc1	20		ENSMUSG00000021027	Ralgapa1	3
ENSMUSG00000029862	Clcn1	20		ENSMUSG00000037110	Ralgapa2	20
ENSMUSG00000022843	Clcn2	20		ENSMUSG00000027652	Ralgapb	12
ENSMUSG00000004319	Clcn3	3		ENSMUSG00000026821	Ralgds	16
ENSMUSG00000000605	Clcn4	9		ENSMUSG00000038831	Ralgps1	2
ENSMUSG00000004317	Clcn5	9		ENSMUSG00000026594	Ralgps2	9
ENSMUSG00000029016	Clcn6	9		ENSMUSG00000027593	Raly	20
ENSMUSG00000036636	Clcn7	20		ENSMUSG00000039717	Ralyl	9
ENSMUSG00000022512	Cldn1	13		ENSMUSG00000038646	Ramac	12
ENSMUSG00000022132	Cldn10	2		ENSMUSG00000034353	Ramp1	19
ENSMUSG00000037625	Cldn11	16		ENSMUSG00000001240	Ramp2	20
ENSMUSG00000046798	Cldn12	1		ENSMUSG00000041046	Ramp3	18
ENSMUSG00000047109	Cldn14	1		ENSMUSG00000029430	Ran	20
ENSMUSG00000066058	Cldn19	20		ENSMUSG00000037415	Ranbp10	20
ENSMUSG00000047230	Cldn2	13		ENSMUSG00000040594	Ranbp17	20
ENSMUSG00000091530	Cldn20	20		ENSMUSG00000003226	Ranbp2	7
ENSMUSG00000079450	Cldn34c1	20		ENSMUSG00000002372	Ranbp3	20
ENSMUSG00000041378	Cldn5	20		ENSMUSG00000048424	Ranbp3l	5
ENSMUSG00000022744	Cldnd1	20		ENSMUSG00000074909	Ranbp6	20
ENSMUSG00000004473	Clec11a	9		ENSMUSG00000038546	Ranbp9	20
ENSMUSG00000053063	Clec12a	1		ENSMUSG00000022391	Rangap1	9
ENSMUSG00000045930	Clec14a	20		ENSMUSG00000032892	Rangrf	20
ENSMUSG00000068663	Clec16a	5		ENSMUSG00000068798	Rap1a	20
ENSMUSG00000033633	Clec18a	20		ENSMUSG00000052681	Rap1b	1
ENSMUSG00000033082	Clec1a	19		ENSMUSG00000041351	Rap1gap	1
ENSMUSG00000030157	Clec2d	1		ENSMUSG00000038807	Rap1gap2	18
ENSMUSG00000089728	Clec2f	7		ENSMUSG00000028149	Rap1gds1	18
ENSMUSG00000079598	Clec2l	18		ENSMUSG00000051615	Rap2a	9
ENSMUSG00000025784	Clec3b	20		ENSMUSG00000036894	Rap2b	6
ENSMUSG00000029915	Clec5a	20		ENSMUSG00000050029	Rap2c	20
ENSMUSG00000079293	Clec7a	20		ENSMUSG00000039844	Rapgef1	20
ENSMUSG00000002190	Clgn	20		ENSMUSG00000062232	Rapgef2	9
ENSMUSG00000020461	Clhc1	20		ENSMUSG00000022469	Rapgef3	4
ENSMUSG00000007041	Clic1	20		ENSMUSG00000087397	Rapgef3os2	20
ENSMUSG00000037242	Clic4	15		ENSMUSG00000049044	Rapgef4	9
ENSMUSG00000023959	Clic5	20		ENSMUSG00000085001	Rapgef4os2	19
ENSMUSG00000022949	Clic6	13		ENSMUSG00000041992	Rapgef5	9
ENSMUSG00000006169	Clint1	20		ENSMUSG00000037533	Rapgef6	20
ENSMUSG00000049550	Clip1	19		ENSMUSG00000038020	Rapgefl1	5
ENSMUSG00000063146	Clip2	9		ENSMUSG00000026014	Raph1	18
ENSMUSG00000013921	Clip3	9		ENSMUSG00000037992	Rara	20
ENSMUSG00000024059	Clip4	9		ENSMUSG00000017491	Rarb	9
ENSMUSG00000026034	Clk1	20		ENSMUSG00000001288	Rarg	20
ENSMUSG00000068917	Clk2	20		ENSMUSG00000049404	Rarres1	20
ENSMUSG00000032316	Clk3	20		ENSMUSG00000009281	Rarres2	14
ENSMUSG00000020385	Clk4	20		ENSMUSG00000018848	Rars	20
ENSMUSG00000021097	Clmn	18		ENSMUSG00000028292	Rars2	20
ENSMUSG00000032024	Clmp	13		ENSMUSG00000021549	Rasa1	20
ENSMUSG00000030720	Cln3	20		ENSMUSG00000032413	Rasa2	20
ENSMUSG00000022125	Cln5	20		ENSMUSG00000031453	Rasa3	15

ENSMUSG00000032245	Cln6	20		ENSMUSG00000004952	Rasa4	18
ENSMUSG00000026317	Cln8	20		ENSMUSG00000029602	Rasal1	20
ENSMUSG00000025439	Clns1a	20		ENSMUSG00000070565	Rasal2	20
ENSMUSG00000029238	Clock	2		ENSMUSG00000052142	Rasal3	20
ENSMUSG00000027079	Clp1	20		ENSMUSG00000049892	Rasd1	18
ENSMUSG00000001829	Clpb	20		ENSMUSG00000034472	Rasd2	9
ENSMUSG00000002660	Clpp	12		ENSMUSG00000030134	Rasgef1a	9
ENSMUSG00000002981	Clptm1	1		ENSMUSG00000089809	Rasgef1b	9
ENSMUSG00000021610	Clptm1l	9		ENSMUSG00000020374	Rasgef1c	3
ENSMUSG00000015357	Clpx	20		ENSMUSG00000032356	Rasgrf1	5
ENSMUSG00000042489	Clspn	9		ENSMUSG00000021708	Rasgrf2	6
ENSMUSG00000039953	Clstn1	1		ENSMUSG00000027347	Rasgrp1	7
ENSMUSG00000032452	Clstn2	5		ENSMUSG00000071042	Rasgrp3	16
ENSMUSG00000008153	Clstn3	18		ENSMUSG00000030589	Rasgrp4	20
ENSMUSG00000028478	Clta	12		ENSMUSG00000044562	Rasip1	13
ENSMUSG00000047547	Cltb	7		ENSMUSG00000034209	Rasl10a	20
ENSMUSG00000047126	Cltc	12		ENSMUSG00000020684	Rasl10b	9
ENSMUSG00000022037	Clu	3		ENSMUSG00000029641	Rasl11a	18
ENSMUSG00000014232	Cluap1	20		ENSMUSG00000049907	Rasl11b	1
ENSMUSG00000020741	Cluh	20		ENSMUSG00000041696	Rasl12	1
ENSMUSG00000041216	Clvs1	8		ENSMUSG00000010067	Rassf1	20
ENSMUSG00000019785	Clvs2	4		ENSMUSG00000098132	Rassf10	20
ENSMUSG00000025545	Clybl	20		ENSMUSG00000027339	Rassf2	16
ENSMUSG00000030282	Cmas	20		ENSMUSG00000025795	Rassf3	18
ENSMUSG00000022235	Cmbl	9		ENSMUSG00000042129	Rassf4	15
ENSMUSG00000039163	Cmc1	20		ENSMUSG00000026430	Rassf5	18
ENSMUSG00000014633	Cmc2	20		ENSMUSG00000029370	Rassf6	6
ENSMUSG00000090110	Cmc4	20		ENSMUSG00000038618	Rassf7	12
ENSMUSG00000034390	Cmip	9		ENSMUSG00000030259	Rassf8	9
ENSMUSG00000042190	Cmklr1	20		ENSMUSG00000044921	Rassf9	13
ENSMUSG00000028719	Cmpk1	19		ENSMUSG00000035275	Raver2	20
ENSMUSG00000020638	Cmpk2	20		ENSMUSG00000022105	Rb1	20
ENSMUSG00000022748	Cmss1	20		ENSMUSG00000025907	Rb1cc1	3
ENSMUSG00000031875	Cmtm3	20		ENSMUSG00000061898	Rbak	20
ENSMUSG00000096188	Cmtm4	12		ENSMUSG00000057236	Rbbp4	20
ENSMUSG00000040759	Cmtm5	16		ENSMUSG00000026439	Rbbp5	20
ENSMUSG00000032434	Cmtm6	20		ENSMUSG00000030779	Rbbp6	20
ENSMUSG00000032436	Cmtm7	20		ENSMUSG00000031353	Rbbp7	9
ENSMUSG00000041012	Cmtm8	20		ENSMUSG00000041238	Rbbp8	1
ENSMUSG00000024019	Cmtr1	20		ENSMUSG00000027428	Rbbp9	20
ENSMUSG00000046441	Cmtr2	5		ENSMUSG00000027466	Rbck1	9
ENSMUSG00000047419	Cmya5	9		ENSMUSG00000024570	Rbfa	12
ENSMUSG00000038085	Cnbd2	12		ENSMUSG00000118061	Rbfaos	9
ENSMUSG00000030057	Cnbp	9		ENSMUSG00000008658	Rbfox1	9
ENSMUSG00000056162	Cndp1	13		ENSMUSG00000033565	Rbfox2	12
ENSMUSG00000024644	Cndp2	16		ENSMUSG00000025576	Rbfox3	18
ENSMUSG00000036810	Cnep1r1	20		ENSMUSG00000078784	Rbis	12
ENSMUSG00000030897	Cnga4	20		ENSMUSG00000029136	Rbks	20
ENSMUSG00000015759	Cnih1	20		ENSMUSG00000027641	Rbl1	20
ENSMUSG00000024873	Cnih2	20		ENSMUSG00000031666	Rbl2	20
ENSMUSG00000026514	Cnih3	19		ENSMUSG00000031060	Rbm10	20
ENSMUSG00000062169	Cnih4	20		ENSMUSG00000032940	Rbm11	1
ENSMUSG00000025658	Cnksr2	9		ENSMUSG00000089824	Rbm12	20
ENSMUSG00000015202	Cnksr3	18		ENSMUSG00000046667	Rbm12b1	20

ENSMUSG00000022025	Cnmd	20		ENSMUSG00000052137	Rbm12b2	20
ENSMUSG00000001349	Cnn1	20		ENSMUSG00000006456	Rbm14	20
ENSMUSG00000004665	Cnn2	20		ENSMUSG00000048109	Rbm15	2
ENSMUSG00000053931	Cnn3	20		ENSMUSG00000074102	Rbm15b	12
ENSMUSG00000025189	Cnnm1	20		ENSMUSG00000037197	Rbm17	20
ENSMUSG00000064105	Cnnm2	20		ENSMUSG00000026889	Rbm18	20
ENSMUSG00000001138	Cnnm3	20		ENSMUSG00000029594	Rbm19	20
ENSMUSG00000037408	Cnnm4	20		ENSMUSG00000043639	Rbm20	20
ENSMUSG00000036550	Cnot1	12		ENSMUSG00000024604	Rbm22	20
ENSMUSG00000056167	Cnot10	20		ENSMUSG00000038132	Rbm24	18
ENSMUSG00000003135	Cnot11	20		ENSMUSG00000010608	Rbm25	2
ENSMUSG00000020166	Cnot2	4		ENSMUSG00000022119	Rbm26	12
ENSMUSG00000035632	Cnot3	20		ENSMUSG00000024491	Rbm27	2
ENSMUSG00000038784	Cnot4	7		ENSMUSG00000029701	Rbm28	18
ENSMUSG00000020362	Cnot6	6		ENSMUSG00000031167	Rbm3	1
ENSMUSG00000034724	Cnot6l	3		ENSMUSG00000099875	Rbm3-ps	20
ENSMUSG00000031601	Cnot7	9		ENSMUSG00000048271	Rbm33	9
ENSMUSG00000020515	Cnot8	20		ENSMUSG00000033931	Rbm34	20
ENSMUSG00000026174	Cnot9	20		ENSMUSG00000027510	Rbm38	20
ENSMUSG00000006782	Cnp	16		ENSMUSG00000027620	Rbm39	18
ENSMUSG00000033159	Cnppd1	20		ENSMUSG00000055188	Rbm3os	20
ENSMUSG00000025381	Cnpy2	12		ENSMUSG00000094936	Rbm4	20
ENSMUSG00000023973	Cnpy3	3		ENSMUSG00000031433	Rbm41	20
ENSMUSG00000036968	Cnpy4	20		ENSMUSG00000036733	Rbm42	20
ENSMUSG00000044288	Cnr1	9		ENSMUSG00000036249	Rbm43	20
ENSMUSG00000044629	Cnrip1	1		ENSMUSG00000042369	Rbm45	20
ENSMUSG00000038949	Cnst	9		ENSMUSG00000033882	Rbm46	20
ENSMUSG00000078653	Cntd1	20		ENSMUSG00000070780	Rbm47	13
ENSMUSG00000028444	Cntfr	18		ENSMUSG00000040302	Rbm48	20
ENSMUSG00000038070	Cntln	9		ENSMUSG00000033760	Rbm4b	3
ENSMUSG00000055022	Cntn1	3		ENSMUSG00000032580	Rbm5	20
ENSMUSG00000053024	Cntn2	15		ENSMUSG00000032582	Rbm6	16
ENSMUSG00000030075	Cntn3	12		ENSMUSG00000101589	Rbm6-ps1	20
ENSMUSG00000064293	Cntn4	20		ENSMUSG00000042396	Rbm7	20
ENSMUSG00000039488	Cntn5	9		ENSMUSG00000038374	Rbm8a	20
ENSMUSG00000030092	Cntn6	9		ENSMUSG00000078184	Rbm8a2	20
ENSMUSG00000017167	Cntnap1	1		ENSMUSG00000026970	Rbms1	20
ENSMUSG00000039419	Cntnap2	20		ENSMUSG00000040043	Rbms2	20
ENSMUSG00000033063	Cntnap3	1		ENSMUSG00000039607	Rbms3	18
ENSMUSG00000031772	Cntnap4	9		ENSMUSG00000031134	Rbmx	3
ENSMUSG00000070695	Cntnap5a	1		ENSMUSG00000031107	Rbmx2	20
ENSMUSG00000067028	Cntnap5b	20		ENSMUSG00000037070	Rbmxl1	20
ENSMUSG00000038048	Cntnap5c	20		ENSMUSG00000046402	Rbp1	13
ENSMUSG00000057110	Cntrl	9		ENSMUSG00000024990	Rbp4	19
ENSMUSG00000032782	Cntrob	20		ENSMUSG00000039191	Rbpj	20
ENSMUSG00000017188	Coa3	12		ENSMUSG00000079575	Rbpj-ps3	20
ENSMUSG00000044881	Coa4	20		ENSMUSG00000017007	Rbpjl	13
ENSMUSG00000026112	Coa5	20		ENSMUSG00000031586	Rbpms	18
ENSMUSG00000051671	Coa6	20		ENSMUSG00000032387	Rbpms2	20
ENSMUSG00000048351	Coa7	2		ENSMUSG00000014550	Rbsn	13
ENSMUSG00000001755	Coasy	2		ENSMUSG00000022400	Rbx1	20
ENSMUSG00000020173	Cobl	7		ENSMUSG00000049832	Rbx1-ps	20
ENSMUSG00000034903	Cobll1	16		ENSMUSG00000040423	Rc3h1	3
ENSMUSG00000020953	Coch	9		ENSMUSG00000075376	Rc3h2	2

ENSMUSG00000018661	Cog1	20		ENSMUSG00000022951	Rcan1	9
ENSMUSG00000031979	Cog2	20		ENSMUSG00000039601	Rcan2	18
ENSMUSG00000034893	Cog3	20		ENSMUSG00000059713	Rcan3	1
ENSMUSG00000031753	Cog4	3		ENSMUSG00000035469	Rcbtb1	16
ENSMUSG00000035933	Cog5	2		ENSMUSG00000022106	Rcbtb2	20
ENSMUSG00000027742	Cog6	16		ENSMUSG00000028896	Rcc1	20
ENSMUSG00000034951	Cog7	18		ENSMUSG00000061979	Rcc1l	20
ENSMUSG00000031916	Cog8	20		ENSMUSG00000040945	Rcc2	20
ENSMUSG00000033983	Coil	20		ENSMUSG00000038930	Rccd1	20
ENSMUSG00000027966	Col11a1	9		ENSMUSG00000024889	Rce1	20
ENSMUSG00000024330	Col11a2	2		ENSMUSG00000029397	Rchy1	20
ENSMUSG00000032332	Col12a1	19		ENSMUSG00000024785	Rcl1	3
ENSMUSG00000058806	Col13a1	1		ENSMUSG00000005973	Rcn1	1
ENSMUSG00000022371	Col14a1	9		ENSMUSG00000032320	Rcn2	20
ENSMUSG00000028339	Col15a1	9		ENSMUSG00000019539	Rcn3	1
ENSMUSG00000040690	Col16a1	9		ENSMUSG00000037896	Rcor1	20
ENSMUSG00000025064	Col17a1	19		ENSMUSG00000024968	Rcor2	20
ENSMUSG00000001435	Col18a1	13		ENSMUSG00000037395	Rcor3	20
ENSMUSG00000026141	Col19a1	1		ENSMUSG00000040723	Rcsd1	20
ENSMUSG00000001506	Col1a1	20		ENSMUSG00000020907	Rcvrn	20
ENSMUSG00000029661	Col1a2	9		ENSMUSG00000089789	Rdh1	20
ENSMUSG00000016356	Col20a1	1		ENSMUSG00000025921	Rdh10	20
ENSMUSG00000079022	Col22a1	20		ENSMUSG00000066441	Rdh11	9
ENSMUSG00000063564	Col23a1	12		ENSMUSG00000021123	Rdh12	1
ENSMUSG00000028197	Col24a1	16		ENSMUSG00000008435	Rdh13	20
ENSMUSG00000058897	Col25a1	9		ENSMUSG00000020621	Rdh14	20
ENSMUSG00000004415	Col26a1	18		ENSMUSG00000025350	Rdh5	13
ENSMUSG00000045672	Col27a1	20		ENSMUSG00000010362	Rdm1	19
ENSMUSG00000068794	Col28a1	20		ENSMUSG00000032050	Rdx	9
ENSMUSG00000022483	Col2a1	20		ENSMUSG00000074269	Rec114	20
ENSMUSG00000026043	Col3a1	20		ENSMUSG00000002324	Rec8	20
ENSMUSG00000031502	Col4a1	19		ENSMUSG00000028476	Reck	20
ENSMUSG00000031503	Col4a2	19		ENSMUSG00000030243	Recql	20
ENSMUSG00000079465	Col4a3	13		ENSMUSG00000033762	Recql4	20
ENSMUSG00000021669	Col4a3bp	3		ENSMUSG00000020752	Recql5	20
ENSMUSG00000067158	Col4a4	13		ENSMUSG00000052852	Reep1	18
ENSMUSG00000031274	Col4a5	13		ENSMUSG00000038555	Reep2	18
ENSMUSG00000026837	Col5a1	20		ENSMUSG00000019873	Reep3	10
ENSMUSG00000026042	Col5a2	20		ENSMUSG00000033589	Reep4	20
ENSMUSG00000004098	Col5a3	20		ENSMUSG00000005873	Reep5	9
ENSMUSG00000001119	Col6a1	3		ENSMUSG00000035504	Reep6	20
ENSMUSG00000020241	Col6a2	18		ENSMUSG00000020275	Rel	20
ENSMUSG00000048126	Col6a3	20		ENSMUSG00000024927	Rela	9
ENSMUSG00000032572	Col6a4	1		ENSMUSG00000002983	Relb	20
ENSMUSG00000091345	Col6a5	1		ENSMUSG00000026319	Relch	20
ENSMUSG00000043719	Col6a6	20		ENSMUSG00000047881	Rell1	18
ENSMUSG00000025650	Col7a1	20		ENSMUSG00000044024	Rell2	9
ENSMUSG00000068196	Col8a1	13		ENSMUSG00000042453	Reln	6
ENSMUSG00000056174	Col8a2	13		ENSMUSG00000008318	Relt	18
ENSMUSG00000026147	Col9a1	20		ENSMUSG00000000359	Rem1	20
ENSMUSG00000028626	Col9a2	9		ENSMUSG00000022176	Rem2	9
ENSMUSG00000027570	Col9a3	13		ENSMUSG00000031387	Renbp	7
ENSMUSG00000079559	Colca2	20		ENSMUSG00000040121	Rep15	18
ENSMUSG00000036103	Colec12	9		ENSMUSG00000052751	Repin1	1

ENSMUSG00000034807	Colgalt1	3		ENSMUSG00000019854	Reps1	9
ENSMUSG00000032649	Colgalt2	20		ENSMUSG00000040855	Reps2	19
ENSMUSG00000057606	Colq	20		ENSMUSG00000029048	Rer1	1
ENSMUSG00000051355	Commd1	20		ENSMUSG00000039852	Rere	9
ENSMUSG00000042705	Commd10	20		ENSMUSG00000030222	Rerg	19
ENSMUSG00000036513	Commd2	20		ENSMUSG00000032712	Resf1	2
ENSMUSG00000051154	Commd3	20		ENSMUSG00000033061	Resp18	17
ENSMUSG00000032299	Commd4	12		ENSMUSG00000029249	Rest	20
ENSMUSG00000055041	Commd5	20		ENSMUSG00000030110	Ret	18
ENSMUSG00000075486	Commd6	20		ENSMUSG00000012705	Retn	20
ENSMUSG00000056941	Commd7	1		ENSMUSG00000022270	Retreg1	18
ENSMUSG00000029213	Commd8	1		ENSMUSG00000049339	Retreg2	20
ENSMUSG00000027163	Commd9	20		ENSMUSG00000017802	Retreg3	20
ENSMUSG00000021773	Comtd1	20		ENSMUSG00000056666	Retsat	20
ENSMUSG00000040782	Cop1	2		ENSMUSG00000026082	Rev1	9
ENSMUSG00000026553	Copa	1		ENSMUSG00000019841	Rev3l	3
ENSMUSG00000030754	Copb1	20		ENSMUSG00000058833	Rex1bd	12
ENSMUSG00000032458	Copb2	1		ENSMUSG00000047417	Rexo1	9
ENSMUSG00000055681	Cope	7		ENSMUSG00000032026	Rexo2	12
ENSMUSG00000030058	Copg1	20		ENSMUSG00000052406	Rexo4	20
ENSMUSG00000025607	Copg2	20		ENSMUSG00000030924	Rexo5	9
ENSMUSG00000031458	Coprs	9		ENSMUSG00000029191	Rfc1	20
ENSMUSG00000027206	Cops2	20		ENSMUSG00000023104	Rfc2	19
ENSMUSG00000019373	Cops3	20		ENSMUSG00000033970	Rfc3	20
ENSMUSG00000035297	Cops4	20		ENSMUSG00000022881	Rfc4	20
ENSMUSG00000025917	Cops5	20		ENSMUSG00000029363	Rfc5	9
ENSMUSG00000019494	Cops6	12		ENSMUSG00000043190	Rfesd	20
ENSMUSG00000030127	Cops7a	12		ENSMUSG00000020696	Rffl	15
ENSMUSG00000026240	Cops7b	20		ENSMUSG00000024712	Rfk	12
ENSMUSG00000034432	Cops8	20		ENSMUSG00000020846	Rflnb	18
ENSMUSG00000073616	Cops9	12		ENSMUSG00000025158	Rfng	20
ENSMUSG00000060992	Copz1	2		ENSMUSG00000052395	Rft1	20
ENSMUSG00000018672	Copz2	13		ENSMUSG00000039316	Rftn1	20
ENSMUSG00000039914	Coq10a	20		ENSMUSG00000025978	Rftn2	20
ENSMUSG00000025981	Coq10b	20		ENSMUSG00000033596	Rfwd3	20
ENSMUSG00000029319	Coq2	20		ENSMUSG00000031706	Rfx1	9
ENSMUSG00000028247	Coq3	20		ENSMUSG00000024206	Rfx2	1
ENSMUSG00000026798	Coq4	20		ENSMUSG00000040929	Rfx3	18
ENSMUSG00000041733	Coq5	20		ENSMUSG00000020037	Rfx4	1
ENSMUSG00000021235	Coq6	20		ENSMUSG00000005774	Rfx5	20
ENSMUSG00000030652	Coq7	20		ENSMUSG00000037674	Rfx7	2
ENSMUSG00000026489	Coq8a	20		ENSMUSG00000036120	Rfxank	20
ENSMUSG00000003762	Coq8b	20		ENSMUSG00000036615	Rfxap	20
ENSMUSG00000031782	Coq9	2		ENSMUSG00000022018	Rgcc	20
ENSMUSG00000030707	Coro1a	20		ENSMUSG00000026482	Rgl1	18
ENSMUSG00000024835	Coro1b	20		ENSMUSG00000041354	Rgl2	20
ENSMUSG00000004530	Coro1c	9		ENSMUSG00000040146	Rgl3	18
ENSMUSG00000028337	Coro2a	17		ENSMUSG00000070509	Rgma	18
ENSMUSG00000041729	Coro2b	20		ENSMUSG00000048027	Rgmb	1
ENSMUSG00000020836	Coro6	18		ENSMUSG00000028468	Rgp1	9
ENSMUSG00000039637	Coro7	20		ENSMUSG00000026358	Rgs1	20
ENSMUSG00000031827	Cotl1	20		ENSMUSG00000030844	Rgs10	3
ENSMUSG00000042148	Cox10	20		ENSMUSG00000024186	Rgs11	1
ENSMUSG00000020544	Cox11	20		ENSMUSG00000029101	Rgs12	9

ENSMUSG00000023020	Cox14	12		ENSMUSG00000052087	Rgs14	3
ENSMUSG00000040018	Cox15	20		ENSMUSG00000026475	Rgs16	18
ENSMUSG00000091803	Cox16	20		ENSMUSG00000019775	Rgs17	17
ENSMUSG00000046516	Cox17	12		ENSMUSG00000002458	Rgs19	20
ENSMUSG00000035505	Cox18	12		ENSMUSG00000026360	Rgs2	19
ENSMUSG00000045438	Cox19	20		ENSMUSG00000002459	Rgs20	9
ENSMUSG00000031818	Cox4i1	12		ENSMUSG00000037627	Rgs22	20
ENSMUSG00000009876	Cox4i2	12		ENSMUSG00000059810	Rgs3	18
ENSMUSG00000000088	Cox5a	12		ENSMUSG00000038530	Rgs4	9
ENSMUSG00000061518	Cox5b	12		ENSMUSG00000026678	Rgs5	20
ENSMUSG00000041697	Cox6a1	12		ENSMUSG00000021219	Rgs6	18
ENSMUSG00000030785	Cox6a2	5		ENSMUSG00000026527	Rgs7	19
ENSMUSG00000036751	Cox6b1	5		ENSMUSG00000021719	Rgs7bp	8
ENSMUSG00000051811	Cox6b2	20		ENSMUSG00000042671	Rgs8	3
ENSMUSG00000014313	Cox6c	12		ENSMUSG00000020599	Rgs9	9
ENSMUSG00000066491	Cox6c2	12		ENSMUSG00000042641	Rgsl1	18
ENSMUSG00000074218	Cox7a1	20		ENSMUSG00000026142	Rhbdd1	20
ENSMUSG00000032330	Cox7a2	12		ENSMUSG00000039917	Rhbdd2	20
ENSMUSG00000024248	Cox7a2l	20		ENSMUSG00000034175	Rhbdd3	12
ENSMUSG00000031231	Cox7b	12		ENSMUSG00000020282	Rhbdf1	20
ENSMUSG00000017778	Cox7c	12		ENSMUSG00000020806	Rhbdf2	20
ENSMUSG00000035885	Cox8a	12		ENSMUSG00000025735	Rhbdl1	20
ENSMUSG00000025488	Cox8b	13		ENSMUSG00000043333	Rhbdl2	20
ENSMUSG00000003617	Cp	20		ENSMUSG00000017692	Rhbdl3	19
ENSMUSG00000071553	Cpa2	20		ENSMUSG00000030549	Rhcg	12
ENSMUSG00000042501	Cpa6	1		ENSMUSG00000028945	Rheb	20
ENSMUSG00000020841	Cpd	20		ENSMUSG00000023755	Rhebl1	17
ENSMUSG00000037852	Cpe	1		ENSMUSG00000048668	Rhno1	20
ENSMUSG00000025586	Cpeb1	9		ENSMUSG00000007815	Rhoa	5
ENSMUSG00000109401	Cpeb1os1	20		ENSMUSG00000054364	Rhob	20
ENSMUSG00000039782	Cpeb2	20		ENSMUSG00000019944	Rhobtb1	20
ENSMUSG00000039652	Cpeb3	3		ENSMUSG00000022075	Rhobtb2	9
ENSMUSG00000020300	Cpeb4	20		ENSMUSG00000021589	Rhobtb3	3
ENSMUSG00000062980	Cped1	20		ENSMUSG00000002233	Rhoc	20
ENSMUSG00000039801	Cplane1	20		ENSMUSG00000041845	Rhod	13
ENSMUSG00000073733	Cplane2	20		ENSMUSG00000029449	Rhof	20
ENSMUSG00000033615	Cplx1	18		ENSMUSG00000073982	Rhog	16
ENSMUSG00000025867	Cplx2	9		ENSMUSG00000029204	Rhoh	20
ENSMUSG00000039714	Cplx3	16		ENSMUSG00000046768	Rhoj	20
ENSMUSG00000020183	Cpm	20		ENSMUSG00000024143	Rhoq	16
ENSMUSG00000025196	Cpn1	20		ENSMUSG00000017686	Rhot1	20
ENSMUSG00000074643	Cpne1	20		ENSMUSG00000025733	Rhot2	1
ENSMUSG00000034361	Cpne2	13		ENSMUSG00000039960	Rhou	20
ENSMUSG00000028228	Cpne3	20		ENSMUSG00000034226	Rhov	20
ENSMUSG00000032564	Cpne4	19		ENSMUSG00000022580	Rhpn1	20
ENSMUSG00000024008	Cpne5	19		ENSMUSG00000030494	Rhpn2	9
ENSMUSG00000022212	Cpne6	5		ENSMUSG00000097451	Rian	20
ENSMUSG00000034796	Cpne7	6		ENSMUSG00000025257	Ribc1	20
ENSMUSG00000052560	Cpne8	20		ENSMUSG00000022431	Ribc2	20
ENSMUSG00000030270	Cpne9	18		ENSMUSG00000038658	Ric1	2
ENSMUSG00000022742	Cpox	1		ENSMUSG00000048330	Ric3	9
ENSMUSG00000065979	Cpped1	20		ENSMUSG00000025485	Ric8a	20
ENSMUSG00000039007	Cpq	13		ENSMUSG00000035620	Ric8b	9
ENSMUSG00000034022	Cpsf1	8		ENSMUSG00000050310	Rictor	2

ENSMUSG00000041781	Cpsf2	20		ENSMUSG00000022323	Rida	20
ENSMUSG00000054309	Cpsf3	1		ENSMUSG00000036202	Rif1	9
ENSMUSG00000029625	Cpsf4	3		ENSMUSG00000028139	Riiad1	20
ENSMUSG00000055531	Cpsf6	2		ENSMUSG00000038195	Rilp	13
ENSMUSG00000034820	Cpsf7	20		ENSMUSG00000029392	Rilpl1	20
ENSMUSG00000024900	Cpt1a	20		ENSMUSG00000029401	Rilpl2	20
ENSMUSG00000007783	Cpt1c	20		ENSMUSG00000029420	Rimbp2	1
ENSMUSG00000028607	Cpt2	18		ENSMUSG00000071636	Rimbp3	9
ENSMUSG00000029073	Cptp	20		ENSMUSG00000048899	Rimkla	18
ENSMUSG00000027408	Cpxm1	20		ENSMUSG00000040649	Rimklb	20
ENSMUSG00000030862	Cpxm2	20		ENSMUSG00000041670	Rims1	20
ENSMUSG00000016481	Cr1l	20		ENSMUSG00000037386	Rims2	18
ENSMUSG00000026616	Cr2	20		ENSMUSG00000032890	Rims3	18
ENSMUSG00000032291	Crabp1	9		ENSMUSG00000035226	Rims4	20
ENSMUSG00000004885	Crabp2	9		ENSMUSG00000024883	Rin1	9
ENSMUSG00000061414	Cracr2a	9		ENSMUSG00000001768	Rin2	20
ENSMUSG00000048200	Cracr2b	20		ENSMUSG00000044456	Rin3	20
ENSMUSG00000045867	Cradd	1		ENSMUSG00000024325	Ring1	11
ENSMUSG00000038002	Cramp1l	20		ENSMUSG00000051735	Rinl	20
ENSMUSG00000026853	Crat	20		ENSMUSG00000028999	Rint1	20
ENSMUSG00000063681	Crb1	20		ENSMUSG00000021428	Riok1	20
ENSMUSG00000035403	Crb2	19		ENSMUSG00000116564	Riok2	20
ENSMUSG00000044279	Crb3	20		ENSMUSG00000024404	Riok3	10
ENSMUSG00000005362	Crbn	20		ENSMUSG00000046791	Riox1	20
ENSMUSG00000025532	Crcp	12		ENSMUSG00000022724	Riox2	9
ENSMUSG00000025958	Creb1	1		ENSMUSG00000021408	Ripk1	20
ENSMUSG00000028466	Creb3	20		ENSMUSG00000041135	Ripk2	18
ENSMUSG00000027230	Creb3l1	20		ENSMUSG00000005251	Ripk4	20
ENSMUSG00000038648	Creb3l2	1		ENSMUSG00000038604	Ripor1	20
ENSMUSG00000035041	Creb3l3	20		ENSMUSG00000036006	Ripor2	18
ENSMUSG00000053007	Creb5	20		ENSMUSG00000047897	Ripply2	20
ENSMUSG00000022521	Crebbp	2		ENSMUSG00000028057	Rit1	20
ENSMUSG00000032652	Crebl2	20		ENSMUSG00000057455	Rit2	20
ENSMUSG00000048249	Crebrf	8		ENSMUSG00000029600	Rita1	20
ENSMUSG00000051451	Crebzf	1		ENSMUSG00000039194	Rlbp1	20
ENSMUSG00000040713	Creg1	18		ENSMUSG00000049878	Rlf	20
ENSMUSG00000050967	Creg2	5		ENSMUSG00000056537	Rlim	2
ENSMUSG00000030284	Creld1	19		ENSMUSG00000024410	Rmc1	20
ENSMUSG00000023272	Creld2	20		ENSMUSG00000028229	Rmdn1	20
ENSMUSG00000063889	Crem	20		ENSMUSG00000036368	Rmdn2	20
ENSMUSG00000049796	Crh	1		ENSMUSG00000070730	Rmdn3	1
ENSMUSG00000021680	Crhbp	5		ENSMUSG00000035367	Rmi1	9
ENSMUSG00000018634	Crhr1	20		ENSMUSG00000019763	Rmnd1	20
ENSMUSG00000003476	Crhr2	13		ENSMUSG00000002222	Rmnd5a	20
ENSMUSG00000024074	Crim1	18		ENSMUSG00000001054	Rmnd5b	9
ENSMUSG00000006360	Crip1	20		ENSMUSG00000112117	Rmst	17
ENSMUSG00000006356	Crip2	19		ENSMUSG00000099021	Rn7s1	10
ENSMUSG00000023968	Crip3	20		ENSMUSG00000099250	Rn7s2	12
ENSMUSG00000024146	Cript	1		ENSMUSG00000092746	Rn7s6	20
ENSMUSG00000025776	Crispld1	9		ENSMUSG00000065037	Rn7sk	12
ENSMUSG00000031825	Crispld2	18		ENSMUSG00000035896	Rnase1	20
ENSMUSG00000017776	Crk	20		ENSMUSG00000021876	Rnase4	20
ENSMUSG00000006134	Crkl	3		ENSMUSG00000020630	Rnaseh1	20
ENSMUSG00000007888	Crlf1	5		ENSMUSG00000052926	Rnaseh2a	20

ENSMUSG00000033467	Crlf2	20		ENSMUSG00000021932	Rnaseh2b	20
ENSMUSG00000017561	Crlf3	20		ENSMUSG00000024925	Rnaseh2c	20
ENSMUSG00000027357	Crls1	20		ENSMUSG00000093989	Rnasek	20
ENSMUSG00000029121	Crmp1	19		ENSMUSG00000066800	Rnasel	20
ENSMUSG00000031736	Crnde	1		ENSMUSG00000094724	Rnaset2b	9
ENSMUSG00000001767	Crnkl1	20		ENSMUSG00000054855	Rnd1	7
ENSMUSG00000040860	Crocc	9		ENSMUSG00000001313	Rnd2	2
ENSMUSG00000084989	Crocc2	14		ENSMUSG00000017144	Rnd3	20
ENSMUSG00000003623	Crot	20		ENSMUSG00000041740	Rnf10	20
ENSMUSG00000043153	Crppa	20		ENSMUSG00000052656	Rnf103	9
ENSMUSG00000047657	Crry-ps	1		ENSMUSG00000028557	Rnf11	9
ENSMUSG00000042401	Crtac1	5		ENSMUSG00000032217	Rnf111	12
ENSMUSG00000032431	Crtap	20		ENSMUSG00000010086	Rnf112	20
ENSMUSG00000003575	Crtc1	9		ENSMUSG00000036537	Rnf113a1	1
ENSMUSG00000027936	Crtc2	20		ENSMUSG00000098134	Rnf113a2	20
ENSMUSG00000030527	Crtc3	1		ENSMUSG00000006418	Rnf114	20
ENSMUSG00000020038	Cry1	20		ENSMUSG00000028098	Rnf115	19
ENSMUSG00000068742	Cry2	1		ENSMUSG00000070426	Rnf121	20
ENSMUSG00000032060	Cryab	16		ENSMUSG00000039328	Rnf122	20
ENSMUSG00000029343	Crybb1	20		ENSMUSG00000041528	Rnf123	20
ENSMUSG00000029352	Crybb3	20		ENSMUSG00000033107	Rnf125	20
ENSMUSG00000019866	Crybg1	18		ENSMUSG00000035890	Rnf126	19
ENSMUSG00000012123	Crybg2	20		ENSMUSG00000031438	Rnf128	20
ENSMUSG00000022723	Crybg3	20		ENSMUSG00000036503	Rnf13	16
ENSMUSG00000038135	Crygn	20		ENSMUSG00000020376	Rnf130	19
ENSMUSG00000021947	Cryl1	20		ENSMUSG00000020707	Rnf135	20
ENSMUSG00000030905	Crym	3		ENSMUSG00000024317	Rnf138	20
ENSMUSG00000028199	Cryz	20		ENSMUSG00000083695	Rnf138rt1	20
ENSMUSG00000058240	Cryzl1	12		ENSMUSG00000037075	Rnf139	3
ENSMUSG00000033488	Cryzl2	20		ENSMUSG00000060450	Rnf14	20
ENSMUSG00000005683	Cs	20		ENSMUSG00000030788	Rnf141	18
ENSMUSG00000023044	Csad	20		ENSMUSG00000020642	Rnf144a	1
ENSMUSG00000042109	Csdc2	18		ENSMUSG00000038068	Rnf144b	16
ENSMUSG00000068823	Csde1	3		ENSMUSG00000019189	Rnf145	1
ENSMUSG00000002718	Cse1l	17		ENSMUSG00000038876	Rnf146	2
ENSMUSG00000014599	Csf1	16		ENSMUSG00000048234	Rnf149	20
ENSMUSG00000024621	Csf1r	20		ENSMUSG00000047747	Rnf150	9
ENSMUSG00000059326	Csf2ra	20		ENSMUSG00000047496	Rnf152	18
ENSMUSG00000071713	Csf2rb	20		ENSMUSG00000052949	Rnf157	20
ENSMUSG00000071714	Csf2rb2	12		ENSMUSG00000025427	Rnf165	20
ENSMUSG00000028859	Csf3r	20		ENSMUSG00000014470	Rnf166	9
ENSMUSG00000036356	Csgalnact1	9		ENSMUSG00000040746	Rnf167	18
ENSMUSG00000042042	Csgalnact2	20		ENSMUSG00000014074	Rnf168	3
ENSMUSG00000032312	Csk	20		ENSMUSG00000058761	Rnf169	20
ENSMUSG00000060924	Csmd1	9		ENSMUSG00000000365	Rnf17	20
ENSMUSG00000028804	Csmd2	1		ENSMUSG00000013878	Rnf170	20
ENSMUSG00000022311	Csmd3	6		ENSMUSG00000101800	Rnf170-ps	20
ENSMUSG00000024576	Csnk1a1	2		ENSMUSG00000021720	Rnf180	1
ENSMUSG00000025162	Csnk1d	1		ENSMUSG00000044164	Rnf182	5
ENSMUSG00000022433	Csnk1e	20		ENSMUSG00000020448	Rnf185	20
ENSMUSG00000032384	Csnk1g1	9		ENSMUSG00000020496	Rnf187	20
ENSMUSG00000003345	Csnk1g2	1		ENSMUSG00000022280	Rnf19a	2
ENSMUSG00000073563	Csnk1g3	20		ENSMUSG00000028793	Rnf19b	20
ENSMUSG00000074698	Csnk2a1	20		ENSMUSG00000026484	Rnf2	4

ENSMUSG00000046707	Csnk2a2	20		ENSMUSG00000028309	Rnf20	20
ENSMUSG00000101523	Csnk2a3	20		ENSMUSG00000058498	Rnf207	1
ENSMUSG00000024387	Csnk2b	20		ENSMUSG00000044628	Rnf208	12
ENSMUSG00000068167	Csnka2ip	12		ENSMUSG00000070327	Rnf213	20
ENSMUSG00000032911	Cspg4	20		ENSMUSG00000042790	Rnf214	5
ENSMUSG00000032482	Cspg5	20		ENSMUSG00000003581	Rnf215	20
ENSMUSG00000056763	Cspp1	20		ENSMUSG00000045078	Rnf216	12
ENSMUSG00000032515	Csrnp1	20		ENSMUSG00000063760	Rnf217	1
ENSMUSG00000044636	Csrnp2	12		ENSMUSG00000022120	Rnf219	1
ENSMUSG00000044647	Csrnp3	1		ENSMUSG00000028677	Rnf220	18
ENSMUSG00000026421	Csrp1	16		ENSMUSG00000110404	Rnf223	20
ENSMUSG00000020186	Csrp2	13		ENSMUSG00000033967	Rnf225	20
ENSMUSG00000027447	Cst3	19		ENSMUSG00000043419	Rnf227	18
ENSMUSG00000024846	Cst6	20		ENSMUSG00000048911	Rnf24	3
ENSMUSG00000068129	Cst7	20		ENSMUSG00000026171	Rnf25	20
ENSMUSG00000047363	Cstad	20		ENSMUSG00000053128	Rnf26	20
ENSMUSG00000005054	Cstb	20		ENSMUSG00000047098	Rnf31	20
ENSMUSG00000027498	Cstf1	20		ENSMUSG00000029130	Rnf32	20
ENSMUSG00000031256	Cstf2	20		ENSMUSG00000029474	Rnf34	20
ENSMUSG00000053536	Cstf2t	12		ENSMUSG00000035696	Rnf38	20
ENSMUSG00000027176	Cstf3	12		ENSMUSG00000036492	Rnf39	20
ENSMUSG00000106106	CT010467.1	20		ENSMUSG00000029110	Rnf4	1
ENSMUSG00000037373	Ctbp1	20		ENSMUSG00000030816	Rnf40	20
ENSMUSG00000030970	Ctbp2	20		ENSMUSG00000025373	Rnf41	20
ENSMUSG00000028189	Ctbs	20		ENSMUSG00000034177	Rnf43	20
ENSMUSG00000020898	Ctc1	20		ENSMUSG00000034928	Rnf44	9
ENSMUSG00000005698	Ctcf	9		ENSMUSG00000015478	Rnf5	2
ENSMUSG00000018559	Ctdnep1	20		ENSMUSG00000029634	Rnf6	13
ENSMUSG00000033323	Ctdp1	19		ENSMUSG00000051234	Rnf7	3
ENSMUSG00000026176	Ctdsp1	20		ENSMUSG00000090083	Rnf8	20
ENSMUSG00000078429	Ctdsp2	9		ENSMUSG00000020521	Rnft1	20
ENSMUSG00000047409	Ctdspl	20		ENSMUSG00000032850	Rnft2	20
ENSMUSG00000033411	Ctdspl2	2		ENSMUSG00000028274	Rngtt	7
ENSMUSG00000042340	Ctf1	20		ENSMUSG00000038650	Rnh1	20
ENSMUSG00000028179	Cth	20		ENSMUSG00000071573	Rnls	20
ENSMUSG00000052928	Ctif	18		ENSMUSG00000009535	Rnmt	20
ENSMUSG00000044258	Ctla2a	20		ENSMUSG00000027981	Rnpc3	8
ENSMUSG00000037815	Ctnna1	1		ENSMUSG00000041926	Rnpep	20
ENSMUSG00000063063	Ctnna2	3		ENSMUSG00000026269	Rnpepl1	20
ENSMUSG00000060843	Ctnna3	20		ENSMUSG00000034681	Rnps1	20
ENSMUSG00000038816	Ctnnal1	20		ENSMUSG00000077323	Rnu11	10
ENSMUSG00000006932	Ctnnb1	9		ENSMUSG00000065176	Rnu12	10
ENSMUSG00000028988	Ctnnbip1	18		ENSMUSG00000095969	Rnu1a1	20
ENSMUSG00000027649	Ctnnbl1	18		ENSMUSG00000095580	Rnu1b1	20
ENSMUSG00000034101	Ctnnd1	20		ENSMUSG00000065773	Rnu1b6	10
ENSMUSG00000022240	Ctnnd2	20		ENSMUSG00000065944	Rnu2-10	20
ENSMUSG00000005949	Ctns	20		ENSMUSG00000095892	Rnu5g	10
ENSMUSG00000028633	Ctps	20		ENSMUSG00000065701	Rny1	10
ENSMUSG00000031360	Ctps2	9		ENSMUSG00000064945	Rny3	10
ENSMUSG00000005609	Ctr9	18		ENSMUSG00000018199	Ro60	19
ENSMUSG00000031896	Ctrl	19		ENSMUSG00000022883	Robo1	20
ENSMUSG00000017760	Ctsa	12		ENSMUSG00000052516	Robo2	6
ENSMUSG00000021939	Ctsb	3		ENSMUSG00000032128	Robo3	9
ENSMUSG00000030560	Ctsc	20		ENSMUSG00000032125	Robo4	20

ENSMUSG00000007891	Ctsd	19		ENSMUSG00000024290	Rock1	20
ENSMUSG00000083282	Ctsf	20		ENSMUSG00000020580	Rock2	7
ENSMUSG00000032359	Ctsh	20		ENSMUSG00000022540	Rogdi	20
ENSMUSG00000028111	Ctsk	20		ENSMUSG00000071648	Rom1	12
ENSMUSG00000021477	Ctsl	20		ENSMUSG00000067847	Romo1	12
ENSMUSG00000028015	Ctso	20		ENSMUSG00000035305	Ror1	9
ENSMUSG00000038642	Ctss	12		ENSMUSG00000021464	Ror2	20
ENSMUSG00000016256	Ctsz	19		ENSMUSG00000032238	Rora	18
ENSMUSG00000031078	Cttn	20		ENSMUSG00000036192	Rorb	18
ENSMUSG00000000416	Cttnbp2	9		ENSMUSG00000028150	Rorc	20
ENSMUSG00000062127	Cttnbp2nl	3		ENSMUSG00000060090	Rp2	20
ENSMUSG00000038888	Ctu1	20		ENSMUSG00000032239	Rp9	2
ENSMUSG00000049482	Ctu2	20		ENSMUSG00000000751	Rpa1	20
ENSMUSG00000048644	Ctxn1	9		ENSMUSG00000028884	Rpa2	20
ENSMUSG00000074872	Ctxn2	1		ENSMUSG00000012483	Rpa3	19
ENSMUSG00000069372	Ctxn3	9		ENSMUSG00000018449	Rpain	12
ENSMUSG00000026726	Cubn	10		ENSMUSG00000034032	Rpap1	20
ENSMUSG00000018378	Cuedc1	20		ENSMUSG00000033773	Rpap2	20
ENSMUSG00000036748	Cuedc2	12		ENSMUSG00000022466	Rpap3	20
ENSMUSG00000029686	Cul1	20		ENSMUSG00000026005	Rpe	9
ENSMUSG00000024231	Cul2	20		ENSMUSG00000028174	Rpe65	20
ENSMUSG00000004364	Cul3	12		ENSMUSG00000028187	Rpf1	20
ENSMUSG00000031446	Cul4a	1		ENSMUSG00000038510	Rpf2	20
ENSMUSG00000031095	Cul4b	13		ENSMUSG00000031174	Rpgr	18
ENSMUSG00000032030	Cul5	2		ENSMUSG00000057132	Rpgrip1	10
ENSMUSG00000038545	Cul7	9		ENSMUSG00000033282	Rpgrip1l	20
ENSMUSG00000040327	Cul9	20		ENSMUSG00000029608	Rph3a	1
ENSMUSG00000024194	Cuta	20		ENSMUSG00000020847	Rph3al	20
ENSMUSG00000026870	Cutal	20		ENSMUSG00000053604	Rpia	1
ENSMUSG00000025193	Cutc	14		ENSMUSG00000008682	Rpl10	19
ENSMUSG00000029705	Cux1	20		ENSMUSG00000058443	Rpl10-ps3	20
ENSMUSG00000042589	Cux2	18		ENSMUSG00000110679	Rpl10-ps5	20
ENSMUSG00000004096	Cwc15	20		ENSMUSG00000037805	Rpl10a	12
ENSMUSG00000027014	Cwc22	9		ENSMUSG00000084416	Rpl10a-ps1	12
ENSMUSG00000018541	Cwc25	20		ENSMUSG00000059291	Rpl11	3
ENSMUSG00000021715	Cwc27	20		ENSMUSG00000038900	Rpl12	3
ENSMUSG00000025200	Cwf19l1	20		ENSMUSG00000000740	Rpl13	12
ENSMUSG00000025898	Cwf19l2	20		ENSMUSG00000082806	Rpl13-ps1	20
ENSMUSG00000029154	Cwh43	20		ENSMUSG00000059835	Rpl13-ps3	10
ENSMUSG00000031778	Cx3cl1	9		ENSMUSG00000059776	Rpl13-ps6	12
ENSMUSG00000052336	Cx3cr1	20		ENSMUSG00000074129	Rpl13a	12
ENSMUSG00000022865	Cxadr	20		ENSMUSG00000062083	Rpl13a-ps1	20
ENSMUSG00000061353	Cxcl12	9		ENSMUSG00000025794	Rpl14	12
ENSMUSG00000021508	Cxcl14	3		ENSMUSG00000046721	Rpl14-ps1	12
ENSMUSG00000018920	Cxcl16	20		ENSMUSG00000012405	Rpl15	20
ENSMUSG00000024560	Cxxc1	20		ENSMUSG00000098915	Rpl15-ps2	7
ENSMUSG00000044365	Cxxc4	3		ENSMUSG00000061167	Rpl15-ps3	20
ENSMUSG00000046668	Cxxc5	18		ENSMUSG00000108442	Rpl15-ps5	18
ENSMUSG00000019590	Cyb561	20		ENSMUSG00000062328	Rpl17	20
ENSMUSG00000034445	Cyb561a3	12		ENSMUSG00000081895	Rpl17-ps10	12
ENSMUSG00000048796	Cyb561d1	20		ENSMUSG00000113948	Rpl17-ps3	20
ENSMUSG00000037190	Cyb561d2	20		ENSMUSG00000084345	Rpl17-ps4	20
ENSMUSG00000024646	Cyb5a	19		ENSMUSG00000081855	Rpl17-ps5	20
ENSMUSG00000031924	Cyb5b	20		ENSMUSG00000082035	Rpl17-ps8	2

ENSMUSG00000044795	Cyb5d1	7		ENSMUSG00000066543	Rpl17-ps9	3
ENSMUSG00000057778	Cyb5d2	20		ENSMUSG00000059070	Rpl18	12
ENSMUSG00000026456	Cyb5r1	1		ENSMUSG00000053173	Rpl18-ps2	20
ENSMUSG00000048065	Cyb5r2	20		ENSMUSG00000045128	Rpl18a	3
ENSMUSG00000018042	Cyb5r3	1		ENSMUSG00000059033	Rpl18a-ps1	10
ENSMUSG00000032872	Cyb5r4	20		ENSMUSG00000017404	Rpl19	12
ENSMUSG00000028621	Cyb5rl	20		ENSMUSG00000064281	Rpl19-ps1	12
ENSMUSG00000006519	Cyba	19		ENSMUSG00000081094	Rpl19-ps11	3
ENSMUSG00000015340	Cybb	20		ENSMUSG00000082109	Rpl19-ps12	20
ENSMUSG00000039294	Cybc1	20		ENSMUSG00000116090	Rpl19-ps6	12
ENSMUSG00000027015	Cybrd1	20		ENSMUSG00000117405	Rpl19-ps7	1
ENSMUSG00000022551	Cyc1	12		ENSMUSG00000041453	Rpl21	7
ENSMUSG00000063694	Cycs	20		ENSMUSG00000105359	Rpl21-ps10	2
ENSMUSG00000030447	Cyfip1	20		ENSMUSG00000105144	Rpl21-ps11	20
ENSMUSG00000020340	Cyfip2	9		ENSMUSG00000058700	Rpl21-ps12	20
ENSMUSG00000020810	Cygb	12		ENSMUSG00000083596	Rpl21-ps15	12
ENSMUSG00000053929	Cyhr1	20		ENSMUSG00000060566	Rpl21-ps3	12
ENSMUSG00000036712	Cyld	9		ENSMUSG00000072714	Rpl21-ps4	9
ENSMUSG00000032323	Cyp11a1	20		ENSMUSG00000059912	Rpl21-ps6	20
ENSMUSG00000024087	Cyp1b1	20		ENSMUSG00000094256	Rpl21-ps7	20
ENSMUSG00000049439	Cyp20a1	20		ENSMUSG00000061684	Rpl21-ps8	20
ENSMUSG00000063415	Cyp26b1	18		ENSMUSG00000028936	Rpl22	12
ENSMUSG00000026170	Cyp27a1	20		ENSMUSG00000080877	Rpl22-ps1	12
ENSMUSG00000006724	Cyp27b1	20		ENSMUSG00000039221	Rpl22l1	12
ENSMUSG00000005547	Cyp2a5	9		ENSMUSG00000071415	Rpl23	12
ENSMUSG00000061740	Cyp2d22	20		ENSMUSG00000058546	Rpl23a	20
ENSMUSG00000022445	Cyp2d26	20		ENSMUSG00000083723	Rpl23a-ps14	20
ENSMUSG00000081225	Cyp2j12	20		ENSMUSG00000081968	Rpl23a-ps2	20
ENSMUSG00000052914	Cyp2j6	19		ENSMUSG00000078126	Rpl23a-ps3	12
ENSMUSG00000015224	Cyp2j9	20		ENSMUSG00000098274	Rpl24	12
ENSMUSG00000030670	Cyp2r1	9		ENSMUSG00000060938	Rpl26	3
ENSMUSG00000040703	Cyp2s1	19		ENSMUSG00000063316	Rpl27	20
ENSMUSG00000027983	Cyp2u1	18		ENSMUSG00000073640	Rpl27-ps3	20
ENSMUSG00000023963	Cyp39a1	20		ENSMUSG00000046364	Rpl27a	20
ENSMUSG00000029727	Cyp3a13	20		ENSMUSG00000061488	Rpl27a-ps1	20
ENSMUSG00000021259	Cyp46a1	9		ENSMUSG00000030432	Rpl28	12
ENSMUSG00000024055	Cyp4f13	2		ENSMUSG00000058603	Rpl28-ps1	12
ENSMUSG00000024292	Cyp4f14	20		ENSMUSG00000079942	Rpl28-ps3	20
ENSMUSG00000073424	Cyp4f15	20		ENSMUSG00000048758	Rpl29	3
ENSMUSG00000048440	Cyp4f16	20		ENSMUSG00000094772	Rpl29-ps2	20
ENSMUSG00000091586	Cyp4f17	20		ENSMUSG00000091785	Rpl29-ps5	20
ENSMUSG00000079057	Cyp4v3	20		ENSMUSG00000060036	Rpl3	20
ENSMUSG00000047155	Cyp4x1	1		ENSMUSG00000084349	Rpl3-ps1	12
ENSMUSG00000086896	Cyp4x1os	20		ENSMUSG00000084131	Rpl3-ps2	20
ENSMUSG00000001467	Cyp51	9		ENSMUSG00000058600	Rpl30	20
ENSMUSG00000039519	Cyp7b1	20		ENSMUSG00000057696	Rpl30-ps1	20
ENSMUSG00000046806	Cyren	6		ENSMUSG00000083411	Rpl30-ps10	20
ENSMUSG00000062563	Cys1	1		ENSMUSG00000085791	Rpl30-ps9	9
ENSMUSG00000052821	Cysltr1	20		ENSMUSG00000073702	Rpl31	3
ENSMUSG00000033470	Cysltr2	20		ENSMUSG00000070667	Rpl31-ps10	20
ENSMUSG00000046727	Cystm1	19		ENSMUSG00000116648	Rpl31-ps12	18
ENSMUSG00000017132	Cyth1	9		ENSMUSG00000051723	Rpl31-ps13	20
ENSMUSG00000003269	Cyth2	3		ENSMUSG00000097803	Rpl31-ps16	20
ENSMUSG00000018001	Cyth3	20		ENSMUSG00000067870	Rpl31-ps8	12

ENSMUSG00000018008	Cyth4	20		ENSMUSG00000094122	Rpl31-ps9	20
ENSMUSG00000041134	Cyyr1	12		ENSMUSG00000057841	Rpl32	12
ENSMUSG00000028608	Czib	8		ENSMUSG00000111356	Rpl32l	3
ENSMUSG00000078700	D030028A08Rik	5		ENSMUSG00000062006	Rpl34	12
ENSMUSG00000085886	D030047H15Rik	20		ENSMUSG00000068396	Rpl34-ps1	12
ENSMUSG00000086296	D030055H07Rik	13		ENSMUSG00000062997	Rpl35	19
ENSMUSG00000047044	D030056L22Rik	20		ENSMUSG00000060636	Rpl35a	12
ENSMUSG00000097393	D030068K23Rik	18		ENSMUSG00000094664	Rpl35a-ps6	20
ENSMUSG00000020255	D10Wsu102e	8		ENSMUSG00000057863	Rpl36	12
ENSMUSG00000041623	D11Wsu47e	20		ENSMUSG00000091845	Rpl36-ps12	3
ENSMUSG00000107208	D130004A15Rik	20		ENSMUSG00000024205	Rpl36-ps2	12
ENSMUSG00000054304	D130007C19Rik	2		ENSMUSG00000066629	Rpl36-ps3	12
ENSMUSG00000097287	D130017N08Rik	1		ENSMUSG00000079435	Rpl36a	12
ENSMUSG00000104145	D130019J16Rik	20		ENSMUSG00000060377	Rpl36a-ps1	20
ENSMUSG00000097121	D130020L05Rik	20		ENSMUSG00000105388	Rpl36a-ps2	12
ENSMUSG00000079038	D130040H23Rik	20		ENSMUSG00000071141	Rpl36a-ps3	20
ENSMUSG00000006711	D130043K22Rik	16		ENSMUSG00000049751	Rpl36al	12
ENSMUSG00000092627	D130058E05Rik	18		ENSMUSG00000041841	Rpl37	12
ENSMUSG00000114598	D130062J10Rik	2		ENSMUSG00000046330	Rpl37a	10
ENSMUSG00000022864	D16Ertd472e	20		ENSMUSG00000072692	Rpl37rt	12
ENSMUSG00000043311	D17H6S53E	18		ENSMUSG00000057322	Rpl38	12
ENSMUSG00000044768	D1Ertd622e	20		ENSMUSG00000083326	Rpl38-ps1	12
ENSMUSG00000087176	D230022J07Rik	1		ENSMUSG00000080921	Rpl38-ps2	6
ENSMUSG00000031889	D230025D16Rik	20		ENSMUSG00000079641	Rpl39	10
ENSMUSG00000113196	D230049E03Rik	20		ENSMUSG00000036305	Rpl39-ps	10
ENSMUSG00000073609	D2hgdh	13		ENSMUSG00000002500	Rpl3l	20
ENSMUSG00000087269	D330023K18Rik	20		ENSMUSG00000032399	Rpl4	12
ENSMUSG00000073437	D330041H03Rik	20		ENSMUSG00000093674	Rpl41	3
ENSMUSG00000085316	D330050G23Rik	20		ENSMUSG00000058558	Rpl5	12
ENSMUSG00000025766	D3Ertd751e	20		ENSMUSG00000082193	Rpl5-ps1	20
ENSMUSG00000103821	D430013B06Rik	20		ENSMUSG00000082064	Rpl5-ps2	20
ENSMUSG00000097559	D430018E03Rik	20		ENSMUSG00000029614	Rpl6	12
ENSMUSG00000094910	D430019H16Rik	18		ENSMUSG00000091086	Rpl6l	20
ENSMUSG00000112980	D430020J02Rik	1		ENSMUSG00000043716	Rpl7	3
ENSMUSG00000097466	D430036J16Rik	9		ENSMUSG00000106926	Rpl7-ps7	20
ENSMUSG00000087589	D430040D24Rik	20		ENSMUSG00000062647	Rpl7a	3
ENSMUSG00000068373	D430041D05Rik	9		ENSMUSG00000101502	Rpl7a-ps10	20
ENSMUSG00000032743	D430042O09Rik	20		ENSMUSG00000067147	Rpl7a-ps11	12
ENSMUSG00000107610	D530018E20Rik	20		ENSMUSG00000071052	Rpl7a-ps5	20
ENSMUSG00000029190	D5Ertd579e	9		ENSMUSG00000106258	Rpl7a-ps7	10
ENSMUSG00000097293	D630002J18Rik	20		ENSMUSG00000063888	Rpl7l1	13
ENSMUSG00000037813	D630003M21Rik	12		ENSMUSG00000003970	Rpl8	10
ENSMUSG00000090168	D630014O11Rik	20		ENSMUSG00000047215	Rpl9	3
ENSMUSG00000044816	D630023F18Rik	1		ENSMUSG00000094989	Rpl9-ps4	12
ENSMUSG00000085772	D630024D03Rik	13		ENSMUSG00000062456	Rpl9-ps6	12
ENSMUSG00000106837	D630030B08Rik	20		ENSMUSG00000047965	Rpl9-ps7	12
ENSMUSG00000112237	D630033A02Rik	20		ENSMUSG00000067274	Rplp0	3
ENSMUSG00000091007	D630036H23Rik	20		ENSMUSG00000007892	Rplp1	3
ENSMUSG00000063455	D630045J12Rik	19		ENSMUSG00000025508	Rplp2	3
ENSMUSG00000030347	D6Wsu163e	20		ENSMUSG00000030062	Rpn1	20
ENSMUSG00000073478	D730003I15Rik	20		ENSMUSG00000027642	Rpn2	20
ENSMUSG00000115685	D730044K07Rik	20		ENSMUSG00000023156	Rpp14	20
ENSMUSG00000054622	D730045B01Rik	1		ENSMUSG00000024446	Rpp21	20
ENSMUSG00000109198	D7Bwg0826e	20		ENSMUSG00000062309	Rpp25	3

ENSMUSG00000030994	D7Ertd443e	9		ENSMUSG00000036114	Rpp25l	20
ENSMUSG00000111828	D830035M03Rik	20		ENSMUSG00000024800	Rpp30	20
ENSMUSG00000107994	D830050J10Rik	20		ENSMUSG00000049950	Rpp38	20
ENSMUSG00000019362	D8Ertd738e	10		ENSMUSG00000021418	Rpp40	1
ENSMUSG00000042874	D930007J09Rik	11		ENSMUSG00000092837	Rpph1	10
ENSMUSG00000097392	D930016D06Rik	1		ENSMUSG00000040446	Rprd1a	9
ENSMUSG00000097083	D930019O06Rik	20		ENSMUSG00000027651	Rprd1b	20
ENSMUSG00000047642	D930020B18Rik	9		ENSMUSG00000028106	Rprd2	7
ENSMUSG00000102553	D930036K23Rik	20		ENSMUSG00000075334	Rprm	1
ENSMUSG00000052563	D930048N14Rik	20		ENSMUSG00000046215	Rprml	18
ENSMUSG00000034574	Daam1	9		ENSMUSG00000052146	Rps10	20
ENSMUSG00000040260	Daam2	20		ENSMUSG00000060438	Rps10-ps1	20
ENSMUSG00000028519	Dab1	9		ENSMUSG00000099764	Rps10-ps2	12
ENSMUSG00000022150	Dab2	20		ENSMUSG00000003429	Rps11	12
ENSMUSG00000026883	Dab2ip	9		ENSMUSG00000090516	Rps11-ps1	20
ENSMUSG00000055639	Dach1	9		ENSMUSG00000061983	Rps12	3
ENSMUSG00000025592	Dach2	1		ENSMUSG00000082746	Rps12-ps1	20
ENSMUSG00000044548	Dact1	6		ENSMUSG00000070692	Rps12-ps10	2
ENSMUSG00000048826	Dact2	18		ENSMUSG00000098181	Rps12-ps24	20
ENSMUSG00000078794	Dact3	20		ENSMUSG00000081103	Rps12-ps26	20
ENSMUSG00000022174	Dad1	20		ENSMUSG00000067038	Rps12-ps3	12
ENSMUSG00000039952	Dag1	7		ENSMUSG00000109509	Rps12-ps4	20
ENSMUSG00000035735	Dagla	20		ENSMUSG00000069862	Rps12-ps9	20
ENSMUSG00000039206	Daglb	20		ENSMUSG00000078087	Rps12l1	12
ENSMUSG00000099784	Dalir	20		ENSMUSG00000090862	Rps13	12
ENSMUSG00000019039	Dalrd3	20		ENSMUSG00000066362	Rps13-ps1	20
ENSMUSG00000106943	Dancr	20		ENSMUSG00000069972	Rps13-ps2	12
ENSMUSG00000053226	Dand5	20		ENSMUSG00000081378	Rps13-ps4	20
ENSMUSG00000039168	Dap	20		ENSMUSG00000024608	Rps14	12
ENSMUSG00000068921	Dap3	20		ENSMUSG00000063457	Rps15	12
ENSMUSG00000021559	Dapk1	15		ENSMUSG00000071419	Rps15-ps2	12
ENSMUSG00000032380	Dapk2	20		ENSMUSG00000118264	Rps15-ps3	20
ENSMUSG00000034974	Dapk3	1		ENSMUSG00000008683	Rps15a	2
ENSMUSG00000028159	Dapp1	20		ENSMUSG00000084314	Rps15a-ps3	20
ENSMUSG00000026356	Dars	18		ENSMUSG00000083757	Rps15a-ps4	12
ENSMUSG00000026709	Dars2	20		ENSMUSG00000067058	Rps15a-ps5	12
ENSMUSG00000053161	Daw1	14		ENSMUSG00000083022	Rps15a-ps6	20
ENSMUSG00000002307	Daxx	20		ENSMUSG00000081087	Rps15a-ps7	20
ENSMUSG00000069565	Dazap1	20		ENSMUSG00000037563	Rps16	12
ENSMUSG00000000346	Dazap2	1		ENSMUSG00000060419	Rps16-ps2	3
ENSMUSG00000010592	Dazl	9		ENSMUSG00000061787	Rps17	12
ENSMUSG00000002297	Dbf4	20		ENSMUSG00000008668	Rps18	11
ENSMUSG00000026385	Dbi	12		ENSMUSG00000083914	Rps18-ps1	12
ENSMUSG00000038057	Dbil5	20		ENSMUSG00000057657	Rps18-ps3	11
ENSMUSG00000034675	Dbn1	18		ENSMUSG00000040952	Rps19	12
ENSMUSG00000031970	Dbndd1	18		ENSMUSG00000080059	Rps19-ps3	20
ENSMUSG00000017734	Dbndd2	20		ENSMUSG00000096942	Rps19-ps6	3
ENSMUSG00000020476	Dbnl	20		ENSMUSG00000051518	Rps19bp1	9
ENSMUSG00000059824	Dbp	1		ENSMUSG00000044533	Rps2	20
ENSMUSG00000029878	Dbpht2	9		ENSMUSG00000081684	Rps2-ps13	10
ENSMUSG00000032469	Dbr1	20		ENSMUSG00000028234	Rps20	12
ENSMUSG00000000340	Dbt	20		ENSMUSG00000039001	Rps21	12
ENSMUSG00000045608	Dbx2	20		ENSMUSG00000049517	Rps23	12
ENSMUSG00000040325	Dcaf1	9		ENSMUSG00000100755	Rps23-ps1	12

ENSMUSG00000035572	Dcaf10	9		ENSMUSG00000066902	Rps23-ps2	12
ENSMUSG00000022214	Dcaf11	20		ENSMUSG00000025290	Rps24	3
ENSMUSG00000028436	Dcaf12	20		ENSMUSG00000069125	Rps24-ps2	20
ENSMUSG00000045284	Dcaf12l1	9		ENSMUSG00000081049	Rps24-ps3	19
ENSMUSG00000050926	Dcaf12l2	20		ENSMUSG00000009927	Rps25	12
ENSMUSG00000022300	Dcaf13	20		ENSMUSG00000067344	Rps25-ps1	12
ENSMUSG00000037103	Dcaf15	3		ENSMUSG00000025362	Rps26	12
ENSMUSG00000041966	Dcaf17	20		ENSMUSG00000059775	Rps26-ps1	10
ENSMUSG00000021222	Dcaf4	12		ENSMUSG00000090733	Rps27	3
ENSMUSG00000049106	Dcaf5	1		ENSMUSG00000020460	Rps27a	12
ENSMUSG00000026571	Dcaf6	9		ENSMUSG00000101501	Rps27a-ps1	20
ENSMUSG00000049354	Dcaf7	20		ENSMUSG00000058838	Rps27a-ps2	20
ENSMUSG00000026554	Dcaf8	20		ENSMUSG00000036781	Rps27l	1
ENSMUSG00000020935	Dcakd	20		ENSMUSG00000050621	Rps27rt	12
ENSMUSG00000019891	Dcbld1	20		ENSMUSG00000067288	Rps28	11
ENSMUSG00000035107	Dcbld2	9		ENSMUSG00000034892	Rps29	10
ENSMUSG00000060534	Dcc	9		ENSMUSG00000030744	Rps3	12
ENSMUSG00000035910	Dcdc2a	20		ENSMUSG00000028081	Rps3a1	20
ENSMUSG00000078552	Dcdc2b	1		ENSMUSG00000062611	Rps3a2	20
ENSMUSG00000074981	Dcdc5	9		ENSMUSG00000059751	Rps3a3	18
ENSMUSG00000036862	Dchs1	1		ENSMUSG00000063171	Rps4l	20
ENSMUSG00000102692	Dchs2	18		ENSMUSG00000031320	Rps4x	3
ENSMUSG00000029366	Dck	1		ENSMUSG00000104699	Rps4x-ps	20
ENSMUSG00000027797	Dclk1	9		ENSMUSG00000012848	Rps5	3
ENSMUSG00000028078	Dclk2	20		ENSMUSG00000028495	Rps6	3
ENSMUSG00000032500	Dclk3	9		ENSMUSG00000063875	Rps6-ps1	20
ENSMUSG00000025077	Dclre1a	9		ENSMUSG00000082465	Rps6-ps3	20
ENSMUSG00000027845	Dclre1b	20		ENSMUSG00000081406	Rps6-ps4	2
ENSMUSG00000026648	Dclre1c	4		ENSMUSG00000003644	Rps6ka1	16
ENSMUSG00000019929	Dcn	1		ENSMUSG00000023809	Rps6ka2	9
ENSMUSG00000021962	Dcp1a	20		ENSMUSG00000031309	Rps6ka3	6
ENSMUSG00000041477	Dcp1b	9		ENSMUSG00000024952	Rps6ka4	9
ENSMUSG00000024472	Dcp2	9		ENSMUSG00000021180	Rps6ka5	19
ENSMUSG00000032040	Dcps	20		ENSMUSG00000020516	Rps6kb1	10
ENSMUSG00000042672	Dcst1	20		ENSMUSG00000089872	Rps6kc1	20
ENSMUSG00000109293	Dcst2	20		ENSMUSG00000019235	Rps6kl1	2
ENSMUSG00000022129	Dct	20		ENSMUSG00000061477	Rps7	12
ENSMUSG00000031562	Dctd	12		ENSMUSG00000047675	Rps8	20
ENSMUSG00000031865	Dctn1	12		ENSMUSG00000071303	Rps8-ps1	20
ENSMUSG00000025410	Dctn2	9		ENSMUSG00000083481	Rps8-ps2	20
ENSMUSG00000028447	Dctn3	12		ENSMUSG00000074547	Rps8-ps4	12
ENSMUSG00000024603	Dctn4	20		ENSMUSG00000006333	Rps9	12
ENSMUSG00000030868	Dctn5	12		ENSMUSG00000032518	Rpsa	3
ENSMUSG00000031516	Dctn6	20		ENSMUSG00000047676	Rpsa-ps10	10
ENSMUSG00000042462	Dctpp1	3		ENSMUSG00000082978	Rpsa-ps11	1
ENSMUSG00000027708	Dcun1d1	12		ENSMUSG00000045055	Rpsa-ps2	1
ENSMUSG00000038506	Dcun1d2	9		ENSMUSG00000117438	Rpsa-ps7	2
ENSMUSG00000048787	Dcun1d3	3		ENSMUSG00000082895	Rpsa-ps9	12
ENSMUSG00000051674	Dcun1d4	20		ENSMUSG00000025583	Rptor	2
ENSMUSG00000032002	Dcun1d5	20		ENSMUSG00000041199	Rpusd1	3
ENSMUSG00000031285	Dcx	16		ENSMUSG00000027324	Rpusd2	13
ENSMUSG00000039450	Dcxr	20		ENSMUSG00000051169	Rpusd3	20
ENSMUSG00000074247	Dda1	20		ENSMUSG00000032044	Rpusd4	20
ENSMUSG00000028194	Ddah1	18		ENSMUSG00000031880	Rrad	20

ENSMUSG00000007039	Ddah2	20		ENSMUSG00000070934	Rraga	20
ENSMUSG00000024740	Ddb1	20		ENSMUSG00000041658	Rragb	18
ENSMUSG00000002109	Ddb2	20		ENSMUSG00000028646	Rragc	20
ENSMUSG00000020182	Ddc	9		ENSMUSG00000028278	Rragd	9
ENSMUSG00000037697	Ddhd1	19		ENSMUSG00000038387	Rras	9
ENSMUSG00000061313	Ddhd2	20		ENSMUSG00000055723	Rras2	5
ENSMUSG00000078515	Ddi2	20		ENSMUSG00000027422	Rrbp1	9
ENSMUSG00000030641	Ddias	20		ENSMUSG00000039087	Rreb1	20
ENSMUSG00000025408	Ddit3	3		ENSMUSG00000028012	Rrh	13
ENSMUSG00000020108	Ddit4	20		ENSMUSG00000030978	Rrm1	20
ENSMUSG00000046818	Ddit4l	1		ENSMUSG00000020649	Rrm2	20
ENSMUSG00000059213	Ddn	9		ENSMUSG00000022292	Rrm2b	16
ENSMUSG00000063428	Ddo	6		ENSMUSG00000022682	Rrn3	20
ENSMUSG00000028757	Ddost	19		ENSMUSG00000004896	Rrnad1	20
ENSMUSG00000003534	Ddr1	15		ENSMUSG00000061032	Rrp1	12
ENSMUSG00000026674	Ddr2	20		ENSMUSG00000035049	Rrp12	1
ENSMUSG00000068290	Ddrgk1	3		ENSMUSG00000001305	Rrp15	20
ENSMUSG00000001666	Ddt	12		ENSMUSG00000058392	Rrp1b	20
ENSMUSG00000037149	Ddx1	20		ENSMUSG00000023971	Rrp36	20
ENSMUSG00000053289	Ddx10	20		ENSMUSG00000018040	Rrp7a	20
ENSMUSG00000035842	Ddx11	1		ENSMUSG00000030888	Rrp8	20
ENSMUSG00000055065	Ddx17	20		ENSMUSG00000041506	Rrp9	20
ENSMUSG00000001674	Ddx18	20		ENSMUSG00000061024	Rrs1	20
ENSMUSG00000015023	Ddx19a	20		ENSMUSG00000031293	Rs1	20
ENSMUSG00000033658	Ddx19b	2		ENSMUSG00000039096	Rsad1	20
ENSMUSG00000027905	Ddx20	12		ENSMUSG00000020641	Rsad2	20
ENSMUSG00000020075	Ddx21	1		ENSMUSG00000044098	Rsbn1	2
ENSMUSG00000003360	Ddx23	20		ENSMUSG00000039968	Rsbn1l	12
ENSMUSG00000041645	Ddx24	20		ENSMUSG00000035623	Rsf1	20
ENSMUSG00000032101	Ddx25	18		ENSMUSG00000037593	Rskr	19
ENSMUSG00000017999	Ddx27	20		ENSMUSG00000058900	Rsl1	20
ENSMUSG00000045538	Ddx28	20		ENSMUSG00000005846	Rsl1d1	20
ENSMUSG00000026806	Ddx31	20		ENSMUSG00000032215	Rsl24d1	20
ENSMUSG00000005481	Ddx39	20		ENSMUSG00000074824	Rslcan18	9
ENSMUSG00000019432	Ddx39b	20		ENSMUSG00000024033	Rsph1	14
ENSMUSG00000000787	Ddx3x	20		ENSMUSG00000075569	Rsph10b	14
ENSMUSG00000069045	Ddx3y	19		ENSMUSG00000009070	Rsph14	20
ENSMUSG00000021758	Ddx4	20		ENSMUSG00000073471	Rsph3a	18
ENSMUSG00000021494	Ddx41	20		ENSMUSG00000023806	Rsph3b	20
ENSMUSG00000020705	Ddx42	20		ENSMUSG00000039552	Rsph4a	14
ENSMUSG00000070291	Ddx43	20		ENSMUSG00000023966	Rsph9	13
ENSMUSG00000021500	Ddx46	2		ENSMUSG00000028871	Rspo1	20
ENSMUSG00000030204	Ddx47	20		ENSMUSG00000051920	Rspo2	20
ENSMUSG00000057788	Ddx49	12		ENSMUSG00000019880	Rspo3	20
ENSMUSG00000020719	Ddx5	18		ENSMUSG00000032852	Rspo4	20
ENSMUSG00000020076	Ddx50	20		ENSMUSG00000050079	Rspry1	20
ENSMUSG00000029504	Ddx51	20		ENSMUSG00000034544	Rsrc1	18
ENSMUSG00000020677	Ddx52	20		ENSMUSG00000029422	Rsrc2	20
ENSMUSG00000029599	Ddx54	20		ENSMUSG00000037266	Rsrp1	20
ENSMUSG00000029389	Ddx55	9		ENSMUSG00000026727	Rsu1	20
ENSMUSG00000004393	Ddx56	20		ENSMUSG00000048617	Rtbdn	6
ENSMUSG00000040296	Ddx58	2		ENSMUSG00000000339	Rtca	20
ENSMUSG00000026404	Ddx59	1		ENSMUSG00000001783	Rtcb	20
ENSMUSG00000032097	Ddx6	7		ENSMUSG00000038685	Rtel1	20

ENSMUSG00000037921	Ddx60	20		ENSMUSG00000027304	Rtf1	20
ENSMUSG00000058886	Deaf1	19		ENSMUSG00000027502	Rtf2	20
ENSMUSG00000028223	Decr1	20		ENSMUSG00000034930	Rtkn	3
ENSMUSG00000036775	Decr2	17		ENSMUSG00000037846	Rtkn2	20
ENSMUSG00000013973	Dedd	20		ENSMUSG00000086965	Rtl10	20
ENSMUSG00000054499	Dedd2	20		ENSMUSG00000071679	Rtl4	20
ENSMUSG00000002257	Def6	9		ENSMUSG00000049191	Rtl5	5
ENSMUSG00000001482	Def8	20		ENSMUSG00000055745	Rtl6	20
ENSMUSG00000038633	Degs1	20		ENSMUSG00000085584	Rtl9	9
ENSMUSG00000021263	Degs2	20		ENSMUSG00000021087	Rtn1	18
ENSMUSG00000021377	Dek	9		ENSMUSG00000030401	Rtn2	20
ENSMUSG00000024442	Dele1	20		ENSMUSG00000024758	Rtn3	1
ENSMUSG00000035392	Dennd1a	20		ENSMUSG00000020458	Rtn4	10
ENSMUSG00000056268	Dennd1b	12		ENSMUSG00000019864	Rtn4ip1	20
ENSMUSG00000002668	Dennd1c	20		ENSMUSG00000045287	Rtn4rl1	19
ENSMUSG00000038456	Dennd2a	20		ENSMUSG00000050896	Rtn4rl2	5
ENSMUSG00000007379	Dennd2c	20		ENSMUSG00000033355	Rtp4	20
ENSMUSG00000027901	Dennd2d	20		ENSMUSG00000021807	Rtraf	20
ENSMUSG00000036661	Dennd3	20		ENSMUSG00000074479	Rtraf-ps	20
ENSMUSG00000053641	Dennd4a	12		ENSMUSG00000023066	Rttn	18
ENSMUSG00000042404	Dennd4b	20		ENSMUSG00000035629	Rubcn	1
ENSMUSG00000038024	Dennd4c	20		ENSMUSG00000034959	Rubcnl	13
ENSMUSG00000035901	Dennd5a	18		ENSMUSG00000020375	Rufy1	20
ENSMUSG00000030313	Dennd5b	12		ENSMUSG00000020070	Rufy2	1
ENSMUSG00000040818	Dennd6a	20		ENSMUSG00000029291	Rufy3	1
ENSMUSG00000015377	Dennd6b	20		ENSMUSG00000035007	Rundc1	9
ENSMUSG00000023106	Denr	20		ENSMUSG00000006575	Rundc3a	2
ENSMUSG00000021697	Depdc1b	20		ENSMUSG00000040570	Rundc3b	20
ENSMUSG00000037426	Depdc5	18		ENSMUSG00000022952	Runx1	20
ENSMUSG00000027173	Depdc7	20		ENSMUSG00000006586	Runx1t1	9
ENSMUSG00000048489	Depp1	1		ENSMUSG00000039153	Runx2	2
ENSMUSG00000022419	Deptor	18		ENSMUSG00000073394	Runx2os1	20
ENSMUSG00000030225	Dera	20		ENSMUSG00000041263	Rusc1	18
ENSMUSG00000022365	Derl1	20		ENSMUSG00000035969	Rusc2	18
ENSMUSG00000018442	Derl2	20		ENSMUSG00000030079	Ruvbl1	20
ENSMUSG00000009092	Derl3	3		ENSMUSG00000003868	Ruvbl2	20
ENSMUSG00000026208	Des	20		ENSMUSG00000019782	Rwdd1	12
ENSMUSG00000022472	Desi1	16		ENSMUSG00000032417	Rwdd2a	6
ENSMUSG00000030610	Det1	20		ENSMUSG00000041079	Rwdd2b	20
ENSMUSG00000039977	Deup1	20		ENSMUSG00000028133	Rwdd3	20
ENSMUSG00000038055	Dexi	15		ENSMUSG00000031568	Rwdd4a	20
ENSMUSG00000028974	Dffa	12		ENSMUSG00000034009	Rxfp1	9
ENSMUSG00000029027	Dffb	20		ENSMUSG00000053368	Rxfp2	20
ENSMUSG00000022555	Dgat1	20		ENSMUSG00000060735	Rxfp3	20
ENSMUSG00000030747	Dgat2	9		ENSMUSG00000015846	Rxra	20
ENSMUSG00000067597	Dgat2l6	2		ENSMUSG00000039656	Rxrb	20
ENSMUSG00000003166	Dgcr2	20		ENSMUSG00000015843	Rxrg	9
ENSMUSG00000025357	Dgka	7		ENSMUSG00000034620	Rxylt1	20
ENSMUSG00000036095	Dgkb	9		ENSMUSG00000072872	Rybp	7
ENSMUSG00000070738	Dgkd	4		ENSMUSG00000055763	Rybp-ps	20
ENSMUSG00000000276	Dgke	1		ENSMUSG00000032547	Ryk	20
ENSMUSG00000022861	Dgkg	5		ENSMUSG00000030592	Ryr1	9
ENSMUSG00000034731	Dgkh	7		ENSMUSG00000021313	Ryr2	9
ENSMUSG00000038665	Dgki	7		ENSMUSG00000057378	Ryr3	9

ENSMUSG00000004815	Dgkq	19		ENSMUSG00000044080	S100a1	12
ENSMUSG00000040479	Dgkz	5		ENSMUSG00000041959	S100a10	2
ENSMUSG00000021185	Dglucy	1		ENSMUSG00000027907	S100a11	2
ENSMUSG00000014554	Dguok	20		ENSMUSG00000042312	S100a13	12
ENSMUSG00000034926	Dhcr24	20		ENSMUSG00000074457	S100a16	12
ENSMUSG00000058454	Dhcr7	9		ENSMUSG00000001021	S100a3	20
ENSMUSG00000012117	Dhdds	9		ENSMUSG00000001025	S100a6	19
ENSMUSG00000011382	Dhdh	20		ENSMUSG00000033208	S100b	5
ENSMUSG00000021707	Dhfr	9		ENSMUSG00000040928	S100pbp	9
ENSMUSG00000031730	Dhodh	20		ENSMUSG00000045092	S1pr1	9
ENSMUSG00000060038	Dhps	20		ENSMUSG00000043895	S1pr2	20
ENSMUSG00000002332	Dhrs1	20		ENSMUSG00000067586	S1pr3	20
ENSMUSG00000034449	Dhrs11	20		ENSMUSG00000045087	S1pr5	16
ENSMUSG00000020834	Dhrs13	20		ENSMUSG00000006763	Saal1	3
ENSMUSG00000087050	Dhrs13os	20		ENSMUSG00000024790	Sac3d1	20
ENSMUSG00000066026	Dhrs3	1		ENSMUSG00000025240	Sacm1l	3
ENSMUSG00000022210	Dhrs4	20		ENSMUSG00000048279	Sacs	12
ENSMUSG00000021094	Dhrs7	20		ENSMUSG00000052833	Sae1	20
ENSMUSG00000042569	Dhrs7b	20		ENSMUSG00000071054	Safb	9
ENSMUSG00000025815	Dhtkd1	5		ENSMUSG00000042625	Safb2	20
ENSMUSG00000029169	Dhx15	20		ENSMUSG00000056055	Sag	20
ENSMUSG00000024422	Dhx16	20		ENSMUSG00000031665	Sall1	9
ENSMUSG00000042426	Dhx29	5		ENSMUSG00000049532	Sall2	9
ENSMUSG00000032480	Dhx30	20		ENSMUSG00000024565	Sall3	9
ENSMUSG00000030986	Dhx32	1		ENSMUSG00000027547	Sall4	20
ENSMUSG00000040620	Dhx33	5		ENSMUSG00000079003	Samd1	20
ENSMUSG00000006019	Dhx34	20		ENSMUSG00000038605	Samd10	18
ENSMUSG00000027655	Dhx35	9		ENSMUSG00000096351	Samd11	20
ENSMUSG00000027770	Dhx36	20		ENSMUSG00000058656	Samd12	9
ENSMUSG00000029480	Dhx37	20		ENSMUSG00000047181	Samd14	9
ENSMUSG00000037993	Dhx38	20		ENSMUSG00000090812	Samd15	9
ENSMUSG00000018425	Dhx40	7		ENSMUSG00000021838	Samd4	18
ENSMUSG00000035051	Dhx57	4		ENSMUSG00000109336	Samd4b	20
ENSMUSG00000017830	Dhx58	11		ENSMUSG00000060487	Samd5	18
ENSMUSG00000034931	Dhx8	20		ENSMUSG00000021770	Samd8	20
ENSMUSG00000042699	Dhx9	20		ENSMUSG00000047735	Samd9l	20
ENSMUSG00000029433	Diablo	20		ENSMUSG00000027639	Samhd1	19
ENSMUSG00000024456	Diaph1	9		ENSMUSG00000022437	Samm50	20
ENSMUSG00000034480	Diaph2	9		ENSMUSG00000022876	Samsn1	20
ENSMUSG00000022021	Diaph3	1		ENSMUSG00000024260	Sap130	12
ENSMUSG00000041415	Dicer1	3		ENSMUSG00000021963	Sap18	20
ENSMUSG00000038914	Dido1	20		ENSMUSG00000061104	Sap18b	12
ENSMUSG00000021692	Dimt1	20		ENSMUSG00000031609	Sap30	12
ENSMUSG00000007682	Dio2	20		ENSMUSG00000020755	Sap30bp	20
ENSMUSG00000020231	Dip2a	18		ENSMUSG00000087064	Sap30bpos	20
ENSMUSG00000023026	Dip2b	20		ENSMUSG00000020519	Sap30l	20
ENSMUSG00000048264	Dip2c	20		ENSMUSG00000026955	Sapcd2	20
ENSMUSG00000029270	Dipk1a	20		ENSMUSG00000020088	Sar1a	20
ENSMUSG00000036186	Dipk1b	7		ENSMUSG00000020386	Sar1b	19
ENSMUSG00000047992	Dipk1c	20		ENSMUSG00000031532	Saraf	20
ENSMUSG00000045414	Dipk2a	20		ENSMUSG00000009614	Sardh	20
ENSMUSG00000037358	Dipk2b	20		ENSMUSG00000050132	Sarm1	3
ENSMUSG00000043670	Diras1	18		ENSMUSG00000078427	Sarnp	20
ENSMUSG00000047842	Diras2	9		ENSMUSG00000068739	Sars	7

ENSMUSG00000033166	Dis3	1		ENSMUSG00000070699	Sars2	20
ENSMUSG00000032396	Dis3l	20		ENSMUSG00000039148	Sart1	20
ENSMUSG00000053333	Dis3l2	7		ENSMUSG00000018974	Sart3	20
ENSMUSG00000043051	Disc1	1		ENSMUSG00000015305	Sash1	18
ENSMUSG00000030768	Disp1	20		ENSMUSG00000031101	Sash3	20
ENSMUSG00000040035	Disp2	18		ENSMUSG00000027959	Sass6	20
ENSMUSG00000041544	Disp3	19		ENSMUSG00000025283	Sat1	20
ENSMUSG00000032064	Dixdc1	20		ENSMUSG00000069835	Sat2	20
ENSMUSG00000031403	Dkc1	20		ENSMUSG00000023927	Satb1	3
ENSMUSG00000028031	Dkk2	20		ENSMUSG00000038331	Satb2	20
ENSMUSG00000030772	Dkk3	5		ENSMUSG00000021067	Sav1	1
ENSMUSG00000030792	Dkkl1	20		ENSMUSG00000038570	Saxo2	18
ENSMUSG00000000168	Dlat	20		ENSMUSG00000045107	Saysd1	20
ENSMUSG00000031523	Dlc1	18		ENSMUSG00000025337	Sbds	2
ENSMUSG00000020664	Dld	20		ENSMUSG00000036529	Sbf1	1
ENSMUSG00000038060	Dlec1	14		ENSMUSG00000038371	Sbf2	20
ENSMUSG00000097589	Dleu2	1		ENSMUSG00000042978	Sbk1	9
ENSMUSG00000048281	Dleu7	9		ENSMUSG00000085272	Sbk3	20
ENSMUSG00000022770	Dlg1	6		ENSMUSG00000038095	Sbno1	20
ENSMUSG00000052572	Dlg2	9		ENSMUSG00000035673	Sbno2	20
ENSMUSG00000000881	Dlg3	4		ENSMUSG00000046056	Sbsn	3
ENSMUSG00000020886	Dlg4	9		ENSMUSG00000032719	Sbspon	18
ENSMUSG00000021782	Dlg5	19		ENSMUSG00000032018	Sc5d	3
ENSMUSG00000003279	Dlgap1	5		ENSMUSG00000038406	Scaf1	20
ENSMUSG00000047495	Dlgap2	9		ENSMUSG00000033228	Scaf11	3
ENSMUSG00000042388	Dlgap3	9		ENSMUSG00000022983	Scaf4	20
ENSMUSG00000061689	Dlgap4	19		ENSMUSG00000046201	Scaf8	3
ENSMUSG00000047428	Dlk2	20		ENSMUSG00000035236	Scai	19
ENSMUSG00000014773	Dll1	20		ENSMUSG00000021687	Scamp1	9
ENSMUSG00000003436	Dll3	20		ENSMUSG00000040188	Scamp2	20
ENSMUSG00000027314	Dll4	20		ENSMUSG00000028049	Scamp3	20
ENSMUSG00000004789	Dlst	20		ENSMUSG00000113949	Scamp4	20
ENSMUSG00000041911	Dlx1	20		ENSMUSG00000040722	Scamp5	9
ENSMUSG00000084946	Dlx1as	20		ENSMUSG00000046229	Scand1	20
ENSMUSG00000023391	Dlx2	20		ENSMUSG00000032485	Scap	20
ENSMUSG00000029755	Dlx5	18		ENSMUSG00000034007	Scaper	16
ENSMUSG00000029754	Dlx6	9		ENSMUSG00000034463	Scara3	9
ENSMUSG00000090063	Dlx6os1	9		ENSMUSG00000022032	Scara5	13
ENSMUSG00000094868	Dlx6os2	20		ENSMUSG00000037936	Scarb1	18
ENSMUSG00000028398	Dmac1	20		ENSMUSG00000029426	Scarb2	18
ENSMUSG00000057229	Dmac2	20		ENSMUSG00000038188	Scarf1	20
ENSMUSG00000054894	Dmac2l	20		ENSMUSG00000012017	Scarf2	20
ENSMUSG00000009640	Dmap1	20		ENSMUSG00000088789	Scarna13	10
ENSMUSG00000022429	Dmc1	20		ENSMUSG00000088689	Scarna17	10
ENSMUSG00000045103	Dmd	1		ENSMUSG00000088185	Scarna2	20
ENSMUSG00000060962	Dmkn	19		ENSMUSG00000089536	Scarna3a	10
ENSMUSG00000029307	Dmp1	20		ENSMUSG00000089281	Scarna6	10
ENSMUSG00000030409	Dmpk	3		ENSMUSG00000088958	Scarna8	10
ENSMUSG00000042372	Dmrt3	13		ENSMUSG00000077506	Scarna9	20
ENSMUSG00000043753	Dmrta1	9		ENSMUSG00000038936	Sccpdh	18
ENSMUSG00000047143	Dmrta2	1		ENSMUSG00000037071	Scd1	16
ENSMUSG00000028610	Dmrtb1	20		ENSMUSG00000025203	Scd2	16
ENSMUSG00000031323	Dmrtc1a	20		ENSMUSG00000025202	Scd3	20
ENSMUSG00000042508	Dmtf1	9		ENSMUSG00000050195	Scd4	20

ENSMUSG00000022099	Dmtn	9		ENSMUSG00000022123	Scel	20
ENSMUSG00000030410	Dmwd	20		ENSMUSG00000020952	Scfd1	1
ENSMUSG00000037416	Dmxl1	2		ENSMUSG00000062110	Scfd2	20
ENSMUSG00000041268	Dmxl2	2		ENSMUSG00000050711	Scg2	20
ENSMUSG00000036875	Dna2	20		ENSMUSG00000032181	Scg3	20
ENSMUSG00000031831	Dnaaf1	20		ENSMUSG00000023236	Scg5	20
ENSMUSG00000020973	Dnaaf2	20		ENSMUSG00000027777	Schip1	5
ENSMUSG00000055809	Dnaaf3	13		ENSMUSG00000059834	Sclt1	20
ENSMUSG00000092192	Dnaaf4	20		ENSMUSG00000026307	Scly	20
ENSMUSG00000025857	Dnaaf5	20		ENSMUSG00000000085	Scmh1	9
ENSMUSG00000019027	Dnah1	1		ENSMUSG00000000037	Scml2	13
ENSMUSG00000038011	Dnah10	14		ENSMUSG00000044770	Scml4	9
ENSMUSG00000018581	Dnah11	14		ENSMUSG00000064329	Scn1a	18
ENSMUSG00000047369	Dnah14	20		ENSMUSG00000019194	Scn1b	18
ENSMUSG00000033987	Dnah17	20		ENSMUSG00000075318	Scn2a	7
ENSMUSG00000005237	Dnah2	14		ENSMUSG00000070304	Scn2b	8
ENSMUSG00000052273	Dnah3	14		ENSMUSG00000057182	Scn3a	7
ENSMUSG00000022262	Dnah5	14		ENSMUSG00000049281	Scn3b	18
ENSMUSG00000052861	Dnah6	20		ENSMUSG00000046480	Scn4b	9
ENSMUSG00000096141	Dnah7a	20		ENSMUSG00000032511	Scn5a	20
ENSMUSG00000041144	Dnah7b	9		ENSMUSG00000034810	Scn7a	9
ENSMUSG00000101337	Dnah7c	20		ENSMUSG00000023033	Scn8a	3
ENSMUSG00000033826	Dnah8	20		ENSMUSG00000075316	Scn9a	20
ENSMUSG00000056752	Dnah9	14		ENSMUSG00000092607	Scnm1	2
ENSMUSG00000061322	Dnaic1	20		ENSMUSG00000030340	Scnn1a	20
ENSMUSG00000034706	Dnaic2	1		ENSMUSG00000069844	Sco1	9
ENSMUSG00000028410	Dnaja1	20		ENSMUSG00000091780	Sco2	20
ENSMUSG00000090197	Dnaja1-ps	20		ENSMUSG00000063253	Scoc	20
ENSMUSG00000031701	Dnaja2	20		ENSMUSG00000028603	Scp2	12
ENSMUSG00000004069	Dnaja3	20		ENSMUSG00000058492	Scp2-ps2	11
ENSMUSG00000032285	Dnaja4	9		ENSMUSG00000000278	Scpep1	9
ENSMUSG00000005483	Dnajb1	20		ENSMUSG00000031610	Scrg1	12
ENSMUSG00000004460	Dnajb11	20		ENSMUSG00000022568	Scrib	16
ENSMUSG00000020109	Dnajb12	20		ENSMUSG00000019124	Scrn1	9
ENSMUSG00000030708	Dnajb13	20		ENSMUSG00000020877	Scrn2	12
ENSMUSG00000074212	Dnajb14	3		ENSMUSG00000008226	Scrn3	1
ENSMUSG00000026203	Dnajb2	2		ENSMUSG00000048385	Scrt1	18
ENSMUSG00000081984	Dnajb3	20		ENSMUSG00000060257	Scrt2	20
ENSMUSG00000028035	Dnajb4	20		ENSMUSG00000026387	Sctr	20
ENSMUSG00000036052	Dnajb5	18		ENSMUSG00000016763	Scube1	20
ENSMUSG00000029131	Dnajb6	20		ENSMUSG00000007279	Scube2	18
ENSMUSG00000014905	Dnajb9	12		ENSMUSG00000038677	Scube3	9
ENSMUSG00000026740	Dnajc1	6		ENSMUSG00000034161	Scx	13
ENSMUSG00000027006	Dnajc10	10		ENSMUSG00000024941	Scyl1	3
ENSMUSG00000039768	Dnajc11	20		ENSMUSG00000069539	Scyl2	3
ENSMUSG00000036764	Dnajc12	20		ENSMUSG00000026584	Scyl3	3
ENSMUSG00000032560	Dnajc13	20		ENSMUSG00000029415	Sdad1	20
ENSMUSG00000025354	Dnajc14	1		ENSMUSG00000020592	Sdc1	18
ENSMUSG00000022013	Dnajc15	9		ENSMUSG00000022261	Sdc2	20
ENSMUSG00000040697	Dnajc16	20		ENSMUSG00000025743	Sdc3	18
ENSMUSG00000034278	Dnajc17	20		ENSMUSG00000017009	Sdc4	20
ENSMUSG00000024350	Dnajc18	9		ENSMUSG00000028249	Sdcbp	9
ENSMUSG00000027679	Dnajc19	12		ENSMUSG00000027456	Sdcbp2	12
ENSMUSG00000083854	Dnajc19-ps	20		ENSMUSG00000026504	Sdccag8	20

ENSMUSG00000029014	Dnajc2	3		ENSMUSG00000038806	Sde2	12
ENSMUSG00000044224	Dnajc21	19		ENSMUSG00000002064	Sdf2	20
ENSMUSG00000027166	Dnajc24	20		ENSMUSG00000022769	Sdf2l1	10
ENSMUSG00000070972	Dnajc25	18		ENSMUSG00000029076	Sdf4	20
ENSMUSG00000020657	Dnajc27	3		ENSMUSG00000021577	Sdha	9
ENSMUSG00000039763	Dnajc28	20		ENSMUSG00000074211	Sdhaf1	12
ENSMUSG00000022136	Dnajc3	20		ENSMUSG00000024668	Sdhaf2	20
ENSMUSG00000061118	Dnajc30	20		ENSMUSG00000042505	Sdhaf3	20
ENSMUSG00000024963	Dnajc4	20		ENSMUSG00000026154	Sdhaf4	12
ENSMUSG00000000826	Dnajc5	20		ENSMUSG00000009863	Sdhb	12
ENSMUSG00000028528	Dnajc6	19		ENSMUSG00000058076	Sdhc	20
ENSMUSG00000014195	Dnajc7	3		ENSMUSG00000000171	Sdhd	20
ENSMUSG00000054405	Dnajc8	1		ENSMUSG00000039683	Sdk1	20
ENSMUSG00000021811	Dnajc9	20		ENSMUSG00000041592	Sdk2	18
ENSMUSG00000042523	Dnal1	18		ENSMUSG00000022223	Sdr39u1	20
ENSMUSG00000022420	Dnal4	3		ENSMUSG00000034308	Sdr42e1	20
ENSMUSG00000042707	Dnali1	14		ENSMUSG00000029596	Sdsl	20
ENSMUSG00000019088	Dnase1l1	20		ENSMUSG00000001103	Sebox	20
ENSMUSG00000024136	Dnase1l2	1		ENSMUSG00000040364	Sec1	12
ENSMUSG00000003812	Dnase2a	20		ENSMUSG00000025724	Sec11a	1
ENSMUSG00000044595	Dnd1	20		ENSMUSG00000024516	Sec11c	7
ENSMUSG00000036766	Dner	18		ENSMUSG00000030298	Sec13	19
ENSMUSG00000030882	Dnhd1	20		ENSMUSG00000020823	Sec14l1	9
ENSMUSG00000075467	Dnlz	12		ENSMUSG00000003585	Sec14l2	1
ENSMUSG00000026825	Dnm1	19		ENSMUSG00000054986	Sec14l3	9
ENSMUSG00000022789	Dnm1l	3		ENSMUSG00000019368	Sec14l4	19
ENSMUSG00000033335	Dnm2	19		ENSMUSG00000091712	Sec14l5	16
ENSMUSG00000040265	Dnm3	18		ENSMUSG00000026924	Sec16a	20
ENSMUSG00000025195	Dnmbp	1		ENSMUSG00000026589	Sec16b	20
ENSMUSG00000004099	Dnmt1	20		ENSMUSG00000034473	Sec22a	20
ENSMUSG00000020661	Dnmt3a	20		ENSMUSG00000027879	Sec22b	20
ENSMUSG00000027478	Dnmt3b	20		ENSMUSG00000061536	Sec22c	20
ENSMUSG00000026209	Dnpep	2		ENSMUSG00000020986	Sec23a	1
ENSMUSG00000040658	Dnph1	20		ENSMUSG00000027429	Sec23b	12
ENSMUSG00000017299	Dnttip1	10		ENSMUSG00000055319	Sec23ip	12
ENSMUSG00000039756	Dnttip2	20		ENSMUSG00000036391	Sec24a	19
ENSMUSG00000052301	Doc2a	20		ENSMUSG00000001052	Sec24b	20
ENSMUSG00000020848	Doc2b	6		ENSMUSG00000039367	Sec24c	20
ENSMUSG00000024871	Doc2g	2		ENSMUSG00000039234	Sec24d	5
ENSMUSG00000058325	Dock1	20		ENSMUSG00000035325	Sec31a	1
ENSMUSG00000038608	Dock10	9		ENSMUSG00000051984	Sec31b	18
ENSMUSG00000031093	Dock11	1		ENSMUSG00000030082	Sec61a1	20
ENSMUSG00000020143	Dock2	20		ENSMUSG00000025816	Sec61a2	1
ENSMUSG00000039716	Dock3	9		ENSMUSG00000053317	Sec61b	20
ENSMUSG00000035954	Dock4	9		ENSMUSG00000078974	Sec61g	12
ENSMUSG00000044447	Dock5	20		ENSMUSG00000027706	Sec62	6
ENSMUSG00000032198	Dock6	20		ENSMUSG00000019802	Sec63	20
ENSMUSG00000028556	Dock7	19		ENSMUSG00000035139	Secisbp2	20
ENSMUSG00000052085	Dock8	20		ENSMUSG00000035093	Secisbp2l	18
ENSMUSG00000025558	Dock9	9		ENSMUSG00000079614	Seh1l	20
ENSMUSG00000078440	Dohh	20		ENSMUSG00000020964	Sel1l	20
ENSMUSG00000068335	Dok1	20		ENSMUSG00000029189	Sel1l3	18
ENSMUSG00000022102	Dok2	18		ENSMUSG00000068874	Selenbp1	20
ENSMUSG00000035711	Dok3	1		ENSMUSG00000037072	Selenof	12

ENSMUSG00000040631	Dok4	20		ENSMUSG00000076437	Selenoh	20
ENSMUSG00000027560	Dok5	20		ENSMUSG00000075703	Selenoi	9
ENSMUSG00000073514	Dok6	1		ENSMUSG00000042682	Selenok	12
ENSMUSG00000044716	Dok7	9		ENSMUSG00000085415	Selenok-ps1	20
ENSMUSG00000075419	Dolk	19		ENSMUSG00000113504	Selenok-ps5	20
ENSMUSG00000026856	Dolpp1	9		ENSMUSG00000113865	Selenok-ps6	20
ENSMUSG00000022960	Donson	1		ENSMUSG00000113014	Selenok-ps8	20
ENSMUSG00000034973	Dop1a	2		ENSMUSG00000075702	Selenom	2
ENSMUSG00000022946	Dop1b	20		ENSMUSG00000050989	Selenon	9
ENSMUSG00000061589	Dot1l	20		ENSMUSG00000035757	Selenoo	18
ENSMUSG00000032123	Dpagt1	20		ENSMUSG00000064373	Selenop	4
ENSMUSG00000030584	Dpf1	9		ENSMUSG00000075701	Selenos	20
ENSMUSG00000024826	Dpf2	20		ENSMUSG00000075700	Selenot	20
ENSMUSG00000021221	Dpf3	5		ENSMUSG00000046750	Selenov	1
ENSMUSG00000078789	Dph1	9		ENSMUSG00000041571	Selenow	19
ENSMUSG00000028540	Dph2	20		ENSMUSG00000048163	Selplg	20
ENSMUSG00000021905	Dph3	20		ENSMUSG00000042541	Sem1	12
ENSMUSG00000033554	Dph5	20		ENSMUSG00000028883	Sema3a	19
ENSMUSG00000057147	Dph6	9		ENSMUSG00000057969	Sema3b	13
ENSMUSG00000026975	Dph7	20		ENSMUSG00000028780	Sema3c	7
ENSMUSG00000078919	Dpm1	20		ENSMUSG00000040254	Sema3d	9
ENSMUSG00000026810	Dpm2	12		ENSMUSG00000063531	Sema3e	6
ENSMUSG00000042737	Dpm3	12		ENSMUSG00000034684	Sema3f	20
ENSMUSG00000036815	Dpp10	9		ENSMUSG00000021904	Sema3g	20
ENSMUSG00000063904	Dpp3	9		ENSMUSG00000028064	Sema4a	1
ENSMUSG00000035000	Dpp4	18		ENSMUSG00000030539	Sema4b	20
ENSMUSG00000061576	Dpp6	13		ENSMUSG00000026121	Sema4c	20
ENSMUSG00000026958	Dpp7	20		ENSMUSG00000021451	Sema4d	1
ENSMUSG00000032393	Dpp8	20		ENSMUSG00000000627	Sema4f	18
ENSMUSG00000001229	Dpp9	20		ENSMUSG00000025207	Sema4g	18
ENSMUSG00000043067	Dpy19l1	15		ENSMUSG00000022231	Sema5a	19
ENSMUSG00000043671	Dpy19l3	9		ENSMUSG00000052133	Sema5b	20
ENSMUSG00000045205	Dpy19l4	20		ENSMUSG00000019647	Sema6a	18
ENSMUSG00000024067	Dpy30	11		ENSMUSG00000001227	Sema6b	20
ENSMUSG00000033308	Dpyd	1		ENSMUSG00000038777	Sema6c	9
ENSMUSG00000022304	Dpys	20		ENSMUSG00000027200	Sema6d	18
ENSMUSG00000022048	Dpysl2	12		ENSMUSG00000038264	Sema7a	16
ENSMUSG00000024501	Dpysl3	9		ENSMUSG00000033075	Senp1	12
ENSMUSG00000025478	Dpysl4	18		ENSMUSG00000022855	Senp2	19
ENSMUSG00000029168	Dpysl5	9		ENSMUSG00000005204	Senp3	20
ENSMUSG00000009145	Dqx1	20		ENSMUSG00000022772	Senp5	19
ENSMUSG00000029265	Dr1	20		ENSMUSG00000034252	Senp6	2
ENSMUSG00000020057	Dram1	20		ENSMUSG00000052917	Senp7	2
ENSMUSG00000027900	Dram2	20		ENSMUSG00000051705	Senp8	20
ENSMUSG00000024914	Drap1	12		ENSMUSG00000026662	Sephs1	20
ENSMUSG00000029005	Draxin	20		ENSMUSG00000049091	Sephs2	20
ENSMUSG00000073102	Drc1	18		ENSMUSG00000029173	Sepsecs	20
ENSMUSG00000056598	Drc3	20		ENSMUSG00000015659	Serac1	20
ENSMUSG00000031786	Drc7	13		ENSMUSG00000036371	Serbp1	16
ENSMUSG00000021478	Drd1	9		ENSMUSG00000021643	Serf1	20
ENSMUSG00000032259	Drd2	9		ENSMUSG00000074884	Serf2	12
ENSMUSG00000022705	Drd3	20		ENSMUSG00000030839	Sergef	10
ENSMUSG00000025496	Drd4	20		ENSMUSG00000058586	Serhl	20
ENSMUSG00000039358	Drd5	5		ENSMUSG00000019877	Serinc1	3

ENSMUSG00000020457	Drg1	19		ENSMUSG00000023232	Serinc2	13
ENSMUSG00000020537	Drg2	20		ENSMUSG00000017707	Serinc3	20
ENSMUSG00000022191	Drosha	20		ENSMUSG00000046110	Serinc4	20
ENSMUSG00000000223	Drp2	1		ENSMUSG00000021703	Serinc5	20
ENSMUSG00000059898	Dsc3	20		ENSMUSG00000027808	Serp1	20
ENSMUSG00000050272	Dscam	19		ENSMUSG00000052584	Serp2	12
ENSMUSG00000032087	Dscaml1	1		ENSMUSG00000021091	Serpina3n	20
ENSMUSG00000022422	Dscc1	20		ENSMUSG00000058260	Serpina9	19
ENSMUSG00000039497	Dse	20		ENSMUSG00000044734	Serpinb1a	16
ENSMUSG00000038702	Dsel	18		ENSMUSG00000051029	Serpinb1b	19
ENSMUSG00000034774	Dsg1c	20		ENSMUSG00000060147	Serpinb6a	20
ENSMUSG00000044393	Dsg2	8		ENSMUSG00000042842	Serpinb6b	20
ENSMUSG00000027635	Dsn1	20		ENSMUSG00000026315	Serpinb8	5
ENSMUSG00000054889	Dsp	5		ENSMUSG00000045827	Serpinb9	20
ENSMUSG00000053268	Dspp	20		ENSMUSG00000022766	Serpind1	2
ENSMUSG00000026131	Dst	18		ENSMUSG00000037411	Serpine1	20
ENSMUSG00000015932	Dstn	20		ENSMUSG00000026249	Serpine2	20
ENSMUSG00000042046	Dstyk	20		ENSMUSG00000000753	Serpinf1	16
ENSMUSG00000027430	Dtd1	12		ENSMUSG00000023224	Serping1	20
ENSMUSG00000020956	Dtd2	20		ENSMUSG00000070436	Serpinh1	20
ENSMUSG00000090326	Dthd1	14		ENSMUSG00000027834	Serpini1	19
ENSMUSG00000037474	Dtl	16		ENSMUSG00000008384	Sertad1	20
ENSMUSG00000024302	Dtna	20		ENSMUSG00000049800	Sertad2	9
ENSMUSG00000071454	Dtnb	9		ENSMUSG00000055200	Sertad3	20
ENSMUSG00000057531	Dtnbp1	3		ENSMUSG00000016262	Sertad4	9
ENSMUSG00000023330	Dtwd1	20		ENSMUSG00000056306	Sertm1	5
ENSMUSG00000024505	Dtwd2	20		ENSMUSG00000038332	Sesn1	20
ENSMUSG00000029603	Dtx1	9		ENSMUSG00000028893	Sesn2	12
ENSMUSG00000004947	Dtx2	20		ENSMUSG00000032009	Sesn3	9
ENSMUSG00000040415	Dtx3	19		ENSMUSG00000042272	Sestd1	19
ENSMUSG00000049502	Dtx3l	9		ENSMUSG00000054766	Set	20
ENSMUSG00000039982	Dtx4	9		ENSMUSG00000024548	Setbp1	1
ENSMUSG00000026281	Dtymk	20		ENSMUSG00000042308	Setd1a	20
ENSMUSG00000022639	Dubr	20		ENSMUSG00000038384	Setd1b	20
ENSMUSG00000025155	Dus1l	20		ENSMUSG00000044791	Setd2	7
ENSMUSG00000031901	Dus2	8		ENSMUSG00000056770	Setd3	20
ENSMUSG00000007603	Dus3l	20		ENSMUSG00000022948	Setd4	20
ENSMUSG00000020648	Dus4l	20		ENSMUSG00000034269	Setd5	12
ENSMUSG00000024190	Dusp1	16		ENSMUSG00000031671	Setd6	20
ENSMUSG00000039384	Dusp10	20		ENSMUSG00000037111	Setd7	3
ENSMUSG00000030002	Dusp11	20		ENSMUSG00000015697	Setdb1	20
ENSMUSG00000026659	Dusp12	20		ENSMUSG00000071350	Setdb2	20
ENSMUSG00000018648	Dusp14	9		ENSMUSG00000034639	Setmar	20
ENSMUSG00000042662	Dusp15	15		ENSMUSG00000043535	Setx	2
ENSMUSG00000030203	Dusp16	3		ENSMUSG00000000632	Sez6	9
ENSMUSG00000047205	Dusp18	9		ENSMUSG00000058153	Sez6l	19
ENSMUSG00000027001	Dusp19	9		ENSMUSG00000030683	Sez6l2	9
ENSMUSG00000069255	Dusp22	20		ENSMUSG00000002129	Sf3a1	20
ENSMUSG00000026544	Dusp23	20		ENSMUSG00000020211	Sf3a2	9
ENSMUSG00000039661	Dusp26	12		ENSMUSG00000028902	Sf3a3	20
ENSMUSG00000026564	Dusp27	18		ENSMUSG00000025982	Sf3b1	9
ENSMUSG00000047067	Dusp28	20		ENSMUSG00000024853	Sf3b2	20
ENSMUSG00000003518	Dusp3	20		ENSMUSG00000033732	Sf3b3	20
ENSMUSG00000031530	Dusp4	5		ENSMUSG00000068856	Sf3b4	20

ENSMUSG00000034765	Dusp5	20		ENSMUSG00000078348	Sf3b5	12
ENSMUSG00000019960	Dusp6	20		ENSMUSG00000037361	Sf3b6	12
ENSMUSG00000053716	Dusp7	20		ENSMUSG00000023764	Sfi1	20
ENSMUSG00000037887	Dusp8	20		ENSMUSG00000006527	Sfmbt1	20
ENSMUSG00000027203	Dut	20		ENSMUSG00000061186	Sfmbt2	1
ENSMUSG00000029071	Dvl1	20		ENSMUSG00000047281	Sfn	1
ENSMUSG00000020888	Dvl2	20		ENSMUSG00000028820	Sfpq	20
ENSMUSG00000003233	Dvl3	18		ENSMUSG00000025066	Sfr1	20
ENSMUSG00000040482	Dxo	20		ENSMUSG00000031548	Sfrp1	3
ENSMUSG00000021791	Dydc2	20		ENSMUSG00000027996	Sfrp2	20
ENSMUSG00000035765	Dym	7		ENSMUSG00000021319	Sfrp4	10
ENSMUSG00000018707	Dync1h1	3		ENSMUSG00000018822	Sfrp5	20
ENSMUSG00000029757	Dync1i1	19		ENSMUSG00000029439	Sfswap	20
ENSMUSG00000027012	Dync1i2	4		ENSMUSG00000073468	Sft2d1	20
ENSMUSG00000032435	Dync1li1	20		ENSMUSG00000040848	Sft2d2	19
ENSMUSG00000035770	Dync1li2	20		ENSMUSG00000044982	Sft2d3	20
ENSMUSG00000047193	Dync2h1	2		ENSMUSG00000112343	Sfta3-ps	20
ENSMUSG00000024253	Dync2li1	20		ENSMUSG00000021474	Sfxn1	20
ENSMUSG00000009013	Dynll1	12		ENSMUSG00000025036	Sfxn2	20
ENSMUSG00000020483	Dynll2	18		ENSMUSG00000025212	Sfxn3	19
ENSMUSG00000047459	Dynlrb1	12		ENSMUSG00000063698	Sfxn4	20
ENSMUSG00000034467	Dynlrb2	14		ENSMUSG00000033720	Sfxn5	8
ENSMUSG00000082691	Dynlt1-ps1	20		ENSMUSG00000029156	Sgcb	20
ENSMUSG00000092074	Dynlt1a	20		ENSMUSG00000020354	Sgcd	20
ENSMUSG00000000579	Dynlt1c	20		ENSMUSG00000004631	Sgce	20
ENSMUSG00000031176	Dynlt3	20		ENSMUSG00000039539	Sgcz	17
ENSMUSG00000022897	Dyrk1a	3		ENSMUSG00000030714	Sgf29	20
ENSMUSG00000002409	Dyrk1b	1		ENSMUSG00000028524	Sgip1	3
ENSMUSG00000028630	Dyrk2	5		ENSMUSG00000019970	Sgk1	2
ENSMUSG00000016526	Dyrk3	20		ENSMUSG00000017868	Sgk2	20
ENSMUSG00000033788	Dysf	3		ENSMUSG00000025915	Sgk3	9
ENSMUSG00000069085	Dytn	20		ENSMUSG00000040451	Sgms1	20
ENSMUSG00000037259	Dzank1	18		ENSMUSG00000026039	Sgo2a	9
ENSMUSG00000042156	Dzip1	9		ENSMUSG00000020097	Sgpl1	20
ENSMUSG00000037784	Dzip1l	9		ENSMUSG00000021054	Sgpp1	20
ENSMUSG00000064061	Dzip3	16		ENSMUSG00000032908	Sgpp2	20
ENSMUSG00000111264	E030022I16Rik	20		ENSMUSG00000005043	Sgsh	20
ENSMUSG00000104851	E030026E10Rik	20		ENSMUSG00000042216	Sgsm1	20
ENSMUSG00000097327	E030030I06Rik	8		ENSMUSG00000038351	Sgsm2	9
ENSMUSG00000087222	E030042O20Rik	20		ENSMUSG00000042303	Sgsm3	20
ENSMUSG00000117011	E130008D07Rik	11		ENSMUSG00000004937	Sgta	2
ENSMUSG00000086782	E130102H24Rik	20		ENSMUSG00000042743	Sgtb	20
ENSMUSG00000048747	E130114P18Rik	20		ENSMUSG00000030733	Sh2b1	20
ENSMUSG00000114069	E130119H09Rik	9		ENSMUSG00000005057	Sh2b2	20
ENSMUSG00000044141	E130201H02Rik	20		ENSMUSG00000042594	Sh2b3	20
ENSMUSG00000074210	E130208F15Rik	20		ENSMUSG00000059013	Sh2d3c	9
ENSMUSG00000087177	E130307A14Rik	20		ENSMUSG00000037833	Sh2d4b	18
ENSMUSG00000045071	E130308A19Rik	3		ENSMUSG00000045349	Sh2d5	9
ENSMUSG00000039244	E130309D02Rik	20		ENSMUSG00000052631	Sh2d6	12
ENSMUSG00000048581	E130311K13Rik	20		ENSMUSG00000040666	Sh3bgr	1
ENSMUSG00000091994	E130317F20Rik	20		ENSMUSG00000031246	Sh3bgrl	20
ENSMUSG00000066170	E230001N04Rik	20		ENSMUSG00000032261	Sh3bgrl2	18
ENSMUSG00000096957	E230013L22Rik	13		ENSMUSG00000028843	Sh3bgrl3	3
ENSMUSG00000087231	E230016M11Rik	20		ENSMUSG00000022436	Sh3bp1	20

ENSMUSG00000104420	E230020A03Rik	20		ENSMUSG00000054520	Sh3bp2	20
ENSMUSG00000108912	E230020D15Rik	9		ENSMUSG00000036206	Sh3bp4	9
ENSMUSG00000044719	E230025N22Rik	9		ENSMUSG00000021892	Sh3bp5	1
ENSMUSG00000097585	E230029C05Rik	20		ENSMUSG00000013646	Sh3bp5l	6
ENSMUSG00000027490	E2f1	20		ENSMUSG00000028082	Sh3d19	18
ENSMUSG00000018983	E2f2	2		ENSMUSG00000073758	Sh3d21	3
ENSMUSG00000016477	E2f3	4		ENSMUSG00000003200	Sh3gl1	20
ENSMUSG00000014859	E2f4	6		ENSMUSG00000028488	Sh3gl2	19
ENSMUSG00000027552	E2f5	20		ENSMUSG00000030638	Sh3gl3	3
ENSMUSG00000057469	E2f6	20		ENSMUSG00000037062	Sh3glb1	1
ENSMUSG00000020185	E2f7	20		ENSMUSG00000026860	Sh3glb2	3
ENSMUSG00000046179	E2f8	20		ENSMUSG00000040990	Sh3kbp1	20
ENSMUSG00000037172	E330009J07Rik	6		ENSMUSG00000053617	Sh3pxd2a	9
ENSMUSG00000073538	E330020D12Rik	20		ENSMUSG00000040711	Sh3pxd2b	19
ENSMUSG00000106383	E330034L11Rik	20		ENSMUSG00000031642	Sh3rf1	2
ENSMUSG00000107531	E330037G11Rik	10		ENSMUSG00000057719	Sh3rf2	9
ENSMUSG00000102973	E430014B02Rik	20		ENSMUSG00000037990	Sh3rf3	18
ENSMUSG00000078580	E430018J23Rik	20		ENSMUSG00000036553	Sh3tc1	20
ENSMUSG00000105519	E430021H15Rik	20		ENSMUSG00000045629	Sh3tc2	20
ENSMUSG00000112964	E430024I08Rik	10		ENSMUSG00000020669	Sh3yl1	3
ENSMUSG00000090069	E430024P14Rik	20		ENSMUSG00000038738	Shank1	19
ENSMUSG00000024137	E4f1	20		ENSMUSG00000037541	Shank2	1
ENSMUSG00000083889	E530001F21Rik	20		ENSMUSG00000022623	Shank3	8
ENSMUSG00000097820	E530011L22Rik	20		ENSMUSG00000022552	Sharpin	20
ENSMUSG00000021890	Eaf1	20		ENSMUSG00000044813	Shb	9
ENSMUSG00000022838	Eaf2	20		ENSMUSG00000042626	Shc1	20
ENSMUSG00000054302	Eapp	20		ENSMUSG00000020312	Shc2	9
ENSMUSG00000030871	Ears2	20		ENSMUSG00000021448	Shc3	19
ENSMUSG00000022339	Ebag9	12		ENSMUSG00000035109	Shc4	20
ENSMUSG00000057098	Ebf1	20		ENSMUSG00000022322	Shcbp1	20
ENSMUSG00000010476	Ebf3	9		ENSMUSG00000042708	Shcbp1l	1
ENSMUSG00000053552	Ebf4	20		ENSMUSG00000039154	Shd	6
ENSMUSG00000003206	Ebi3	20		ENSMUSG00000046280	She	20
ENSMUSG00000028729	Ebna1bp2	12		ENSMUSG00000033256	Shf	9
ENSMUSG00000031168	Ebp	20		ENSMUSG00000038884	Shfl	3
ENSMUSG00000021928	Ebpl	20		ENSMUSG00000002633	Shh	20
ENSMUSG00000021810	Ecd	20		ENSMUSG00000044461	Shisa2	1
ENSMUSG00000057530	Ece1	20		ENSMUSG00000050010	Shisa3	9
ENSMUSG00000022842	Ece2	9		ENSMUSG00000041889	Shisa4	20
ENSMUSG00000026247	Ecel1	19		ENSMUSG00000025647	Shisa5	20
ENSMUSG00000053898	Ech1	10		ENSMUSG00000053930	Shisa6	19
ENSMUSG00000019883	Echdc1	20		ENSMUSG00000053550	Shisa7	9
ENSMUSG00000028601	Echdc2	12		ENSMUSG00000096883	Shisa8	20
ENSMUSG00000039063	Echdc3	20		ENSMUSG00000022494	Shisa9	20
ENSMUSG00000025465	Echs1	20		ENSMUSG00000062760	Shisal1	18
ENSMUSG00000024132	Eci1	20		ENSMUSG00000089832	Shkbp1	9
ENSMUSG00000021417	Eci2	20		ENSMUSG00000044991	Shld1	1
ENSMUSG00000028108	Ecm1	20		ENSMUSG00000041471	Shld2	20
ENSMUSG00000043631	Ecm2	20		ENSMUSG00000118537	Shld3	20
ENSMUSG00000050812	Ecpas	7		ENSMUSG00000020534	Shmt1	20
ENSMUSG00000026051	Ecrg4	13		ENSMUSG00000025403	Shmt2	20
ENSMUSG00000073599	Ecscr	20		ENSMUSG00000024976	Shoc2	20
ENSMUSG00000066839	Ecsit	1		ENSMUSG00000027833	Shox2	9
ENSMUSG00000027699	Ect2	20		ENSMUSG00000005951	Shpk	20

ENSMUSG00000071392	Ect2l	20		ENSMUSG00000090112	Shprh	20
ENSMUSG00000059327	Eda	20		ENSMUSG00000035378	Shq1	20
ENSMUSG00000034457	Eda2r	20		ENSMUSG00000018387	Shroom1	20
ENSMUSG00000003227	Edar	20		ENSMUSG00000045180	Shroom2	20
ENSMUSG00000095105	Edaradd	18		ENSMUSG00000029381	Shroom3	9
ENSMUSG00000038957	Edc3	20		ENSMUSG00000068270	Shroom4	12
ENSMUSG00000036270	Edc4	18		ENSMUSG00000041362	Shtn1	20
ENSMUSG00000030104	Edem1	20		ENSMUSG00000001942	Siae	20
ENSMUSG00000038312	Edem2	20		ENSMUSG00000036840	Siah1a	20
ENSMUSG00000043019	Edem3	20		ENSMUSG00000040749	Siah1b	20
ENSMUSG00000015092	Edf1	12		ENSMUSG00000036432	Siah2	20
ENSMUSG00000034488	Edil3	18		ENSMUSG00000091722	Siah3	18
ENSMUSG00000021367	Edn1	20		ENSMUSG00000022696	Sidt1	5
ENSMUSG00000027524	Edn3	13		ENSMUSG00000034908	Sidt2	15
ENSMUSG00000031616	Ednra	20		ENSMUSG00000025494	Sigirr	20
ENSMUSG00000022122	Ednrb	16		ENSMUSG00000027322	Siglec1	20
ENSMUSG00000039990	Edrf1	20		ENSMUSG00000030474	Siglece	20
ENSMUSG00000036499	Eea1	20		ENSMUSG00000039013	Siglecf	20
ENSMUSG00000030619	Eed	20		ENSMUSG00000051504	Siglech	20
ENSMUSG00000037742	Eef1a1	20		ENSMUSG00000036078	Sigmar1	20
ENSMUSG00000117822	Eef1a1-ps1	10		ENSMUSG00000024042	Sik1	1
ENSMUSG00000016349	Eef1a2	19		ENSMUSG00000037112	Sik2	7
ENSMUSG00000021951	Eef1akmt1	19		ENSMUSG00000034135	Sik3	1
ENSMUSG00000030960	Eef1akmt2	20		ENSMUSG00000027854	Sike1	20
ENSMUSG00000115219	Eef1akmt4	20		ENSMUSG00000024357	Sil1	19
ENSMUSG00000026694	Eef1aknmt	20		ENSMUSG00000043183	Simc1	1
ENSMUSG00000025967	Eef1b2	12		ENSMUSG00000042557	Sin3a	7
ENSMUSG00000055762	Eef1d	2		ENSMUSG00000031622	Sin3b	20
ENSMUSG00000001707	Eef1e1	20		ENSMUSG00000039985	Sinhcaf	20
ENSMUSG00000071644	Eef1g	20		ENSMUSG00000056917	Sipa1	20
ENSMUSG00000034994	Eef2	18		ENSMUSG00000042700	Sipa1l1	9
ENSMUSG00000035064	Eef2k	20		ENSMUSG00000001995	Sipa1l2	9
ENSMUSG00000022544	Eef2kmt	1		ENSMUSG00000030583	Sipa1l3	5
ENSMUSG00000033216	Eefsec	18		ENSMUSG00000037902	Sirpa	18
ENSMUSG00000036611	Eepd1	9		ENSMUSG00000020063	Sirt1	20
ENSMUSG00000068617	Efcab1	20		ENSMUSG00000015149	Sirt2	16
ENSMUSG00000020562	Efcab10	20		ENSMUSG00000025486	Sirt3	20
ENSMUSG00000021176	Efcab11	20		ENSMUSG00000029524	Sirt4	20
ENSMUSG00000030321	Efcab12	20		ENSMUSG00000054021	Sirt5	20
ENSMUSG00000034210	Efcab14	12		ENSMUSG00000034748	Sirt6	20
ENSMUSG00000026495	Efcab2	9		ENSMUSG00000025138	Sirt7	1
ENSMUSG00000050944	Efcab5	20		ENSMUSG00000064326	Siva1	12
ENSMUSG00000022441	Efcab6	20		ENSMUSG00000038805	Six3	9
ENSMUSG00000044083	Efcab8	1		ENSMUSG00000093460	Six3os1	19
ENSMUSG00000044056	Efcab9	20		ENSMUSG00000034460	Six4	18
ENSMUSG00000068263	Efcc1	20		ENSMUSG00000040841	Six5	20
ENSMUSG00000020467	Efemp1	20		ENSMUSG00000020492	Ska2	20
ENSMUSG00000024909	Efemp2	20		ENSMUSG00000021965	Ska3	20
ENSMUSG00000023931	Efhb	13		ENSMUSG00000057058	Skap1	20
ENSMUSG00000041809	Efhc1	13		ENSMUSG00000059182	Skap2	20
ENSMUSG00000025038	Efhc2	1		ENSMUSG00000029050	Ski	9
ENSMUSG00000026255	Efhd1	18		ENSMUSG00000054074	Skida1	20
ENSMUSG00000040659	Efhd2	19		ENSMUSG00000027660	Skil	7
ENSMUSG00000038563	Efl1	20		ENSMUSG00000040356	Skiv2l	7

ENSMUSG00000027954	Efna1	20		ENSMUSG00000022245	Skor1	20
ENSMUSG00000003070	Efna2	20		ENSMUSG00000036309	Skp1a	12
ENSMUSG00000028039	Efna3	20		ENSMUSG00000022372	Sla	20
ENSMUSG00000048915	Efna5	19		ENSMUSG00000055717	Slain1	16
ENSMUSG00000031217	Efnb1	20		ENSMUSG00000036087	Slain2	12
ENSMUSG00000001300	Efnb2	9		ENSMUSG00000053318	Slamf8	20
ENSMUSG00000003934	Efnb3	16		ENSMUSG00000026548	Slamf9	20
ENSMUSG00000015002	Efr3a	19		ENSMUSG00000004642	Slbp	9
ENSMUSG00000020658	Efr3b	18		ENSMUSG00000032806	Slc10a3	20
ENSMUSG00000022203	Efs	13		ENSMUSG00000029219	Slc10a4	9
ENSMUSG00000020929	Eftud2	3		ENSMUSG00000031684	Slc10a7	20
ENSMUSG00000028017	Egf	20		ENSMUSG00000026177	Slc11a1	20
ENSMUSG00000063600	Egfem1	11		ENSMUSG00000023030	Slc11a2	20
ENSMUSG00000000402	Egfl6	20		ENSMUSG00000027202	Slc12a1	20
ENSMUSG00000026921	Egfl7	20		ENSMUSG00000024597	Slc12a2	15
ENSMUSG00000015467	Egfl8	20		ENSMUSG00000031766	Slc12a3	20
ENSMUSG00000042961	Egflam	19		ENSMUSG00000017765	Slc12a4	13
ENSMUSG00000020122	Egfr	20		ENSMUSG00000017740	Slc12a5	20
ENSMUSG00000031987	Egln1	20		ENSMUSG00000027130	Slc12a6	4
ENSMUSG00000058709	Egln2	20		ENSMUSG00000017756	Slc12a7	13
ENSMUSG00000035105	Egln3	18		ENSMUSG00000035506	Slc12a8	18
ENSMUSG00000038418	Egr1	9		ENSMUSG00000037344	Slc12a9	3
ENSMUSG00000033730	Egr3	9		ENSMUSG00000018459	Slc13a3	20
ENSMUSG00000071341	Egr4	20		ENSMUSG00000029843	Slc13a4	13
ENSMUSG00000042302	Ehbp1	19		ENSMUSG00000020805	Slc13a5	20
ENSMUSG00000024937	Ehbp1l1	20		ENSMUSG00000059336	Slc14a1	20
ENSMUSG00000074364	Ehd2	9		ENSMUSG00000022899	Slc15a2	20
ENSMUSG00000024065	Ehd3	9		ENSMUSG00000024737	Slc15a3	15
ENSMUSG00000027293	Ehd4	20		ENSMUSG00000029416	Slc15a4	20
ENSMUSG00000022853	Ehhadh	20		ENSMUSG00000032902	Slc16a1	18
ENSMUSG00000036893	Ehmt1	3		ENSMUSG00000019838	Slc16a10	20
ENSMUSG00000013787	Ehmt2	20		ENSMUSG00000040938	Slc16a11	20
ENSMUSG00000062762	Ei24	18		ENSMUSG00000009378	Slc16a12	13
ENSMUSG00000091337	Eid1	1		ENSMUSG00000044367	Slc16a13	20
ENSMUSG00000046058	Eid2	20		ENSMUSG00000026220	Slc16a14	1
ENSMUSG00000070705	Eid2b	20		ENSMUSG00000033965	Slc16a2	13
ENSMUSG00000109864	Eid3	20		ENSMUSG00000025161	Slc16a3	20
ENSMUSG00000035530	Eif1	19		ENSMUSG00000027896	Slc16a4	20
ENSMUSG00000074553	Eif1-ps1	6		ENSMUSG00000041920	Slc16a6	9
ENSMUSG00000114179	Eif1-ps2	20		ENSMUSG00000020102	Slc16a7	2
ENSMUSG00000057561	Eif1a	20		ENSMUSG00000032988	Slc16a8	13
ENSMUSG00000024841	Eif1ad	9		ENSMUSG00000037762	Slc16a9	13
ENSMUSG00000067194	Eif1ax	19		ENSMUSG00000049624	Slc17a5	20
ENSMUSG00000006941	Eif1b	20		ENSMUSG00000030500	Slc17a6	18
ENSMUSG00000027810	Eif2a	20		ENSMUSG00000070570	Slc17a7	5
ENSMUSG00000029613	Eif2ak1	20		ENSMUSG00000019935	Slc17a8	9
ENSMUSG00000024079	Eif2ak2	20		ENSMUSG00000023393	Slc17a9	9
ENSMUSG00000031668	Eif2ak3	9		ENSMUSG00000025094	Slc18a2	17
ENSMUSG00000005102	Eif2ak4	1		ENSMUSG00000100241	Slc18a3	20
ENSMUSG00000029388	Eif2b1	20		ENSMUSG00000037455	Slc18b1	18
ENSMUSG00000004788	Eif2b2	20		ENSMUSG00000001436	Slc19a1	20
ENSMUSG00000028683	Eif2b3	20		ENSMUSG00000040918	Slc19a2	20
ENSMUSG00000029145	Eif2b4	20		ENSMUSG00000038496	Slc19a3	20
ENSMUSG00000003235	Eif2b5	20		ENSMUSG00000024935	Slc1a1	19

ENSMUSG00000026427	Eif2d	20		ENSMUSG00000005089	Slc1a2	9
ENSMUSG00000021116	Eif2s1	20		ENSMUSG00000005360	Slc1a3	9
ENSMUSG00000074656	Eif2s2	20		ENSMUSG00000020142	Slc1a4	20
ENSMUSG00000035150	Eif2s3x	19		ENSMUSG00000001918	Slc1a5	20
ENSMUSG00000069049	Eif2s3y	19		ENSMUSG00000005357	Slc1a6	7
ENSMUSG00000024991	Eif3a	20		ENSMUSG00000027397	Slc20a1	18
ENSMUSG00000056076	Eif3b	9		ENSMUSG00000037656	Slc20a2	20
ENSMUSG00000030738	Eif3c	20		ENSMUSG00000033147	Slc22a15	18
ENSMUSG00000016554	Eif3d	20		ENSMUSG00000022199	Slc22a17	18
ENSMUSG00000022336	Eif3e	20		ENSMUSG00000063652	Slc22a21	20
ENSMUSG00000031029	Eif3f	1		ENSMUSG00000038267	Slc22a23	9
ENSMUSG00000070319	Eif3g	20		ENSMUSG00000023828	Slc22a3	1
ENSMUSG00000022312	Eif3h	12		ENSMUSG00000020334	Slc22a4	18
ENSMUSG00000028798	Eif3i	3		ENSMUSG00000018900	Slc22a5	12
ENSMUSG00000027236	Eif3j1	20		ENSMUSG00000024650	Slc22a6	10
ENSMUSG00000043424	Eif3j2	20		ENSMUSG00000063796	Slc22a8	19
ENSMUSG00000053565	Eif3k	12		ENSMUSG00000027340	Slc23a2	18
ENSMUSG00000033047	Eif3l	20		ENSMUSG00000029847	Slc23a4	20
ENSMUSG00000027170	Eif3m	1		ENSMUSG00000037996	Slc24a2	18
ENSMUSG00000081629	Eif3s6-ps1	20		ENSMUSG00000063873	Slc24a3	19
ENSMUSG00000091697	Eif3s6-ps2	20		ENSMUSG00000041771	Slc24a4	9
ENSMUSG00000101188	Eif4a-ps4	20		ENSMUSG00000035183	Slc24a5	13
ENSMUSG00000059796	Eif4a1	20		ENSMUSG00000003528	Slc25a1	2
ENSMUSG00000022884	Eif4a2	20		ENSMUSG00000025792	Slc25a10	20
ENSMUSG00000025580	Eif4a3	10		ENSMUSG00000014606	Slc25a11	20
ENSMUSG00000058655	Eif4b	20		ENSMUSG00000027010	Slc25a12	9
ENSMUSG00000028156	Eif4e	20		ENSMUSG00000015112	Slc25a13	20
ENSMUSG00000026254	Eif4e2	9		ENSMUSG00000031105	Slc25a14	20
ENSMUSG00000093661	Eif4e3	20		ENSMUSG00000031482	Slc25a15	20
ENSMUSG00000031490	Eif4ebp1	12		ENSMUSG00000071253	Slc25a16	20
ENSMUSG00000020091	Eif4ebp2	12		ENSMUSG00000022404	Slc25a17	20
ENSMUSG00000020454	Eif4enif1	20		ENSMUSG00000004902	Slc25a18	6
ENSMUSG00000045983	Eif4g1	19		ENSMUSG00000020744	Slc25a19	20
ENSMUSG00000005610	Eif4g2	3		ENSMUSG00000032602	Slc25a20	20
ENSMUSG00000028760	Eif4g3	19		ENSMUSG00000035472	Slc25a21	20
ENSMUSG00000040731	Eif4h	20		ENSMUSG00000019082	Slc25a22	19
ENSMUSG00000021282	Eif5	1		ENSMUSG00000046329	Slc25a23	9
ENSMUSG00000078812	Eif5a	2		ENSMUSG00000040322	Slc25a24	3
ENSMUSG00000050192	Eif5a2	18		ENSMUSG00000026819	Slc25a25	1
ENSMUSG00000043618	Eif5al3-ps	18		ENSMUSG00000045100	Slc25a26	20
ENSMUSG00000026083	Eif5b	20		ENSMUSG00000023912	Slc25a27	20
ENSMUSG00000027613	Eif6	12		ENSMUSG00000040414	Slc25a28	20
ENSMUSG00000036613	Eipr1	20		ENSMUSG00000021265	Slc25a29	9
ENSMUSG00000036941	Elac1	20		ENSMUSG00000061904	Slc25a3	20
ENSMUSG00000020549	Elac2	20		ENSMUSG00000022003	Slc25a30	20
ENSMUSG00000040028	Elavl1	3		ENSMUSG00000028982	Slc25a33	8
ENSMUSG00000008489	Elavl2	19		ENSMUSG00000040740	Slc25a34	20
ENSMUSG00000003410	Elavl3	9		ENSMUSG00000018740	Slc25a35	13
ENSMUSG00000028546	Elavl4	18		ENSMUSG00000032449	Slc25a36	3
ENSMUSG00000036461	Elf1	20		ENSMUSG00000034248	Slc25a37	18
ENSMUSG00000037174	Elf2	20		ENSMUSG00000032519	Slc25a38	20
ENSMUSG00000031103	Elf4	20		ENSMUSG00000018677	Slc25a39	20
ENSMUSG00000027186	Elf5	20		ENSMUSG00000031633	Slc25a4	20
ENSMUSG00000048988	Elfn1	20		ENSMUSG00000054099	Slc25a40	20

ENSMUSG00000043460	Elfn2	5		ENSMUSG00000002346	Slc25a42	20
ENSMUSG00000009406	Elk1	9		ENSMUSG00000037636	Slc25a43	20
ENSMUSG00000008398	Elk3	20		ENSMUSG00000050144	Slc25a44	13
ENSMUSG00000026436	Elk4	10		ENSMUSG00000024818	Slc25a45	20
ENSMUSG00000070002	Ell	20		ENSMUSG00000024259	Slc25a46	3
ENSMUSG00000001542	Ell2	3		ENSMUSG00000048856	Slc25a47	20
ENSMUSG00000041112	Elmo1	1		ENSMUSG00000016319	Slc25a5	20
ENSMUSG00000017670	Elmo2	20		ENSMUSG00000045973	Slc25a51	20
ENSMUSG00000014791	Elmo3	1		ENSMUSG00000044348	Slc25a53	20
ENSMUSG00000041986	Elmod1	9		ENSMUSG00000046959	Slc26a1	20
ENSMUSG00000035151	Elmod2	20		ENSMUSG00000040441	Slc26a10	9
ENSMUSG00000056698	Elmod3	20		ENSMUSG00000039908	Slc26a11	9
ENSMUSG00000042507	Elmsan1	20		ENSMUSG00000034320	Slc26a2	20
ENSMUSG00000029675	Eln	20		ENSMUSG00000029015	Slc26a5	9
ENSMUSG00000028668	Eloa	20		ENSMUSG00000040569	Slc26a7	20
ENSMUSG00000055839	Elob	12		ENSMUSG00000036196	Slc26a8	20
ENSMUSG00000079658	Eloc	20		ENSMUSG00000031808	Slc27a1	4
ENSMUSG00000013822	Elof1	20		ENSMUSG00000027359	Slc27a2	20
ENSMUSG00000006390	Elovl1	3		ENSMUSG00000027932	Slc27a3	6
ENSMUSG00000021364	Elovl2	14		ENSMUSG00000059316	Slc27a4	9
ENSMUSG00000032262	Elovl4	12		ENSMUSG00000021553	Slc28a3	13
ENSMUSG00000032349	Elovl5	16		ENSMUSG00000023942	Slc29a1	20
ENSMUSG00000041220	Elovl6	3		ENSMUSG00000024891	Slc29a2	9
ENSMUSG00000021696	Elovl7	20		ENSMUSG00000020100	Slc29a3	18
ENSMUSG00000028431	Elp1	20		ENSMUSG00000050822	Slc29a4	13
ENSMUSG00000024271	Elp2	9		ENSMUSG00000028645	Slc2a1	20
ENSMUSG00000022031	Elp3	20		ENSMUSG00000027661	Slc2a10	20
ENSMUSG00000027167	Elp4	1		ENSMUSG00000037490	Slc2a12	13
ENSMUSG00000018565	Elp5	20		ENSMUSG00000036298	Slc2a13	7
ENSMUSG00000054836	Elp6	20		ENSMUSG00000003153	Slc2a3	7
ENSMUSG00000021728	Emb	13		ENSMUSG00000018566	Slc2a4	18
ENSMUSG00000078517	Emc1	1		ENSMUSG00000085028	Slc2a4rg-ps	20
ENSMUSG00000008140	Emc10	20		ENSMUSG00000028976	Slc2a5	20
ENSMUSG00000022337	Emc2	20		ENSMUSG00000036067	Slc2a6	20
ENSMUSG00000030286	Emc3	1		ENSMUSG00000026791	Slc2a8	20
ENSMUSG00000027131	Emc4	20		ENSMUSG00000005107	Slc2a9	20
ENSMUSG00000047260	Emc6	20		ENSMUSG00000026614	Slc30a10	3
ENSMUSG00000055943	Emc7	11		ENSMUSG00000029151	Slc30a3	5
ENSMUSG00000031819	Emc8	20		ENSMUSG00000021629	Slc30a5	3
ENSMUSG00000022217	Emc9	18		ENSMUSG00000024069	Slc30a6	20
ENSMUSG00000054690	Emcn	20		ENSMUSG00000054414	Slc30a7	20
ENSMUSG00000001964	Emd	20		ENSMUSG00000029221	Slc30a9	20
ENSMUSG00000073436	Eme2	20		ENSMUSG00000066150	Slc31a1	13
ENSMUSG00000004268	Emg1	18		ENSMUSG00000066152	Slc31a2	17
ENSMUSG00000034164	Emid1	20		ENSMUSG00000037771	Slc32a1	19
ENSMUSG00000029163	Emilin1	20		ENSMUSG00000027822	Slc33a1	10
ENSMUSG00000024053	Emilin2	20		ENSMUSG00000006469	Slc34a3	20
ENSMUSG00000058070	Eml1	18		ENSMUSG00000028293	Slc35a1	20
ENSMUSG00000040811	Eml2	18		ENSMUSG00000031156	Slc35a2	20
ENSMUSG00000071647	Eml3	20		ENSMUSG00000027957	Slc35a3	20
ENSMUSG00000032624	Eml4	16		ENSMUSG00000033272	Slc35a4	20
ENSMUSG00000051166	Eml5	9		ENSMUSG00000022664	Slc35a5	20
ENSMUSG00000044072	Eml6	20		ENSMUSG00000020873	Slc35b1	20
ENSMUSG00000030208	Emp1	20		ENSMUSG00000037089	Slc35b2	20

ENSMUSG00000022505	Emp2	20		ENSMUSG00000021432	Slc35b3	20
ENSMUSG00000040212	Emp3	20		ENSMUSG00000018999	Slc35b4	13
ENSMUSG00000035401	Emsy	9		ENSMUSG00000049922	Slc35c1	20
ENSMUSG00000033726	Emx1	20		ENSMUSG00000017664	Slc35c2	20
ENSMUSG00000043969	Emx2	1		ENSMUSG00000028521	Slc35d1	9
ENSMUSG00000087095	Emx2os	1		ENSMUSG00000033114	Slc35d2	3
ENSMUSG00000039095	En2	1		ENSMUSG00000050473	Slc35d3	6
ENSMUSG00000022995	Enah	9		ENSMUSG00000019731	Slc35e1	12
ENSMUSG00000041773	Enc1	4		ENSMUSG00000042202	Slc35e2	20
ENSMUSG00000037419	Endod1	4		ENSMUSG00000060181	Slc35e3	18
ENSMUSG00000015337	Endog	20		ENSMUSG00000048807	Slc35e4	20
ENSMUSG00000022468	Endou	6		ENSMUSG00000038602	Slc35f1	9
ENSMUSG00000039850	Endov	20		ENSMUSG00000042195	Slc35f2	20
ENSMUSG00000026814	Eng	20		ENSMUSG00000057060	Slc35f3	18
ENSMUSG00000033857	Engase	20		ENSMUSG00000021852	Slc35f4	7
ENSMUSG00000028445	Enho	4		ENSMUSG00000026342	Slc35f5	9
ENSMUSG00000013155	Enkd1	20		ENSMUSG00000029175	Slc35f6	20
ENSMUSG00000026679	Enkur	20		ENSMUSG00000044026	Slc35g1	20
ENSMUSG00000063524	Eno1	2		ENSMUSG00000070287	Slc35g2	20
ENSMUSG00000059040	Eno1b	12		ENSMUSG00000020261	Slc36a1	1
ENSMUSG00000004267	Eno2	20		ENSMUSG00000043885	Slc36a4	19
ENSMUSG00000060600	Eno3	6		ENSMUSG00000024036	Slc37a1	20
ENSMUSG00000048029	Eno4	20		ENSMUSG00000032122	Slc37a2	13
ENSMUSG00000029326	Enoph1	20		ENSMUSG00000029924	Slc37a3	20
ENSMUSG00000022012	Enox1	9		ENSMUSG00000032114	Slc37a4	20
ENSMUSG00000031109	Enox2	20		ENSMUSG00000023169	Slc38a1	18
ENSMUSG00000028024	Enpep	12		ENSMUSG00000061306	Slc38a10	1
ENSMUSG00000037370	Enpp1	1		ENSMUSG00000061171	Slc38a11	20
ENSMUSG00000022425	Enpp2	13		ENSMUSG00000022462	Slc38a2	18
ENSMUSG00000019989	Enpp3	2		ENSMUSG00000010064	Slc38a3	20
ENSMUSG00000023961	Enpp4	20		ENSMUSG00000022464	Slc38a4	18
ENSMUSG00000023960	Enpp5	9		ENSMUSG00000031170	Slc38a5	20
ENSMUSG00000038173	Enpp6	1		ENSMUSG00000044712	Slc38a6	20
ENSMUSG00000038619	Ensa	12		ENSMUSG00000036534	Slc38a7	20
ENSMUSG00000048120	Entpd1	9		ENSMUSG00000034224	Slc38a8	1
ENSMUSG00000015085	Entpd2	20		ENSMUSG00000047789	Slc38a9	19
ENSMUSG00000041608	Entpd3	20		ENSMUSG00000052310	Slc39a1	20
ENSMUSG00000022066	Entpd4b	20		ENSMUSG00000025986	Slc39a10	9
ENSMUSG00000021236	Entpd5	9		ENSMUSG00000041654	Slc39a11	20
ENSMUSG00000033068	Entpd6	20		ENSMUSG00000036949	Slc39a12	18
ENSMUSG00000025192	Entpd7	20		ENSMUSG00000002105	Slc39a13	20
ENSMUSG00000026927	Entr1	20		ENSMUSG00000022094	Slc39a14	16
ENSMUSG00000022338	Eny2	20		ENSMUSG00000072572	Slc39a2	20
ENSMUSG00000035245	Eogt	18		ENSMUSG00000046822	Slc39a3	6
ENSMUSG00000032446	Eomes	1		ENSMUSG00000063354	Slc39a4	20
ENSMUSG00000055024	Ep300	20		ENSMUSG00000024270	Slc39a6	19
ENSMUSG00000029505	Ep400	12		ENSMUSG00000024327	Slc39a7	20
ENSMUSG00000024140	Epas1	18		ENSMUSG00000053897	Slc39a8	20
ENSMUSG00000028906	Epb41	7		ENSMUSG00000048833	Slc39a9	18
ENSMUSG00000027624	Epb41l1	9		ENSMUSG00000024131	Slc3a1	20
ENSMUSG00000019978	Epb41l2	20		ENSMUSG00000010095	Slc3a2	20
ENSMUSG00000024044	Epb41l3	18		ENSMUSG00000025993	Slc40a1	20
ENSMUSG00000024376	Epb41l4a	9		ENSMUSG00000013275	Slc41a1	9
ENSMUSG00000087590	Epb41l4aos	20		ENSMUSG00000034591	Slc41a2	9

ENSMUSG00000028434	Epb41l4b	9		ENSMUSG00000030089	Slc41a3	17
ENSMUSG00000026383	Epb41l5	12		ENSMUSG00000038178	Slc43a2	20
ENSMUSG00000024240	Epc1	20		ENSMUSG00000027074	Slc43a3	1
ENSMUSG00000069495	Epc2	20		ENSMUSG00000028412	Slc44a1	16
ENSMUSG00000002808	Epdr1	20		ENSMUSG00000057193	Slc44a2	1
ENSMUSG00000029859	Epha1	20		ENSMUSG00000028360	Slc44a5	20
ENSMUSG00000028876	Epha10	9		ENSMUSG00000039838	Slc45a1	20
ENSMUSG00000006445	Epha2	20		ENSMUSG00000026435	Slc45a3	1
ENSMUSG00000052504	Epha3	5		ENSMUSG00000079020	Slc45a4	9
ENSMUSG00000026235	Epha4	5		ENSMUSG00000020829	Slc46a1	9
ENSMUSG00000029245	Epha5	5		ENSMUSG00000029650	Slc46a3	20
ENSMUSG00000055540	Epha6	2		ENSMUSG00000081534	Slc48a1	16
ENSMUSG00000028289	Epha7	5		ENSMUSG00000022848	Slc49a4	20
ENSMUSG00000028661	Epha8	20		ENSMUSG00000026904	Slc4a10	1
ENSMUSG00000032537	Ephb1	7		ENSMUSG00000074796	Slc4a11	1
ENSMUSG00000028664	Ephb2	19		ENSMUSG00000029141	Slc4a1ap	9
ENSMUSG00000005958	Ephb3	20		ENSMUSG00000028962	Slc4a2	13
ENSMUSG00000029710	Ephb4	20		ENSMUSG00000006576	Slc4a3	20
ENSMUSG00000029869	Ephb6	18		ENSMUSG00000060961	Slc4a4	8
ENSMUSG00000038776	Ephx1	1		ENSMUSG00000068323	Slc4a5	13
ENSMUSG00000022040	Ephx2	9		ENSMUSG00000021733	Slc4a7	5
ENSMUSG00000033805	Ephx4	20		ENSMUSG00000023032	Slc4a8	18
ENSMUSG00000055493	Epm2a	19		ENSMUSG00000024485	Slc4a9	20
ENSMUSG00000046785	Epm2aip1	3		ENSMUSG00000027953	Slc50a1	12
ENSMUSG00000035203	Epn1	20		ENSMUSG00000022560	Slc52a2	20
ENSMUSG00000001036	Epn2	9		ENSMUSG00000027463	Slc52a3	20
ENSMUSG00000010080	Epn3	18		ENSMUSG00000030769	Slc5a11	19
ENSMUSG00000043439	Epop	1		ENSMUSG00000030781	Slc5a2	6
ENSMUSG00000006235	Epor	20		ENSMUSG00000089774	Slc5a3	20
ENSMUSG00000115388	Eppk1	14		ENSMUSG00000000792	Slc5a5	9
ENSMUSG00000026615	Eprs	1		ENSMUSG00000006641	Slc5a6	13
ENSMUSG00000028552	Eps15	19		ENSMUSG00000023945	Slc5a7	9
ENSMUSG00000006276	Eps15l1	1		ENSMUSG00000030310	Slc6a1	20
ENSMUSG00000015766	Eps8	19		ENSMUSG00000030307	Slc6a11	18
ENSMUSG00000006154	Eps8l1	20		ENSMUSG00000030108	Slc6a13	20
ENSMUSG00000025504	Eps8l2	18		ENSMUSG00000019894	Slc6a15	16
ENSMUSG00000040600	Eps8l3	20		ENSMUSG00000094152	Slc6a16	20
ENSMUSG00000022014	Epsti1	20		ENSMUSG00000027894	Slc6a17	9
ENSMUSG00000028575	Eqtn	20		ENSMUSG00000036814	Slc6a20a	13
ENSMUSG00000020832	Eral1	20		ENSMUSG00000021609	Slc6a3	9
ENSMUSG00000021583	Erap1	18		ENSMUSG00000039728	Slc6a5	1
ENSMUSG00000062312	Erbb2	20		ENSMUSG00000030096	Slc6a6	9
ENSMUSG00000018166	Erbb3	16		ENSMUSG00000052026	Slc6a7	6
ENSMUSG00000062209	Erbb4	20		ENSMUSG00000019558	Slc6a8	16
ENSMUSG00000021709	Erbin	1		ENSMUSG00000028542	Slc6a9	18
ENSMUSG00000030172	Erc1	5		ENSMUSG00000041313	Slc7a1	1
ENSMUSG00000040640	Erc2	9		ENSMUSG00000030495	Slc7a10	13
ENSMUSG00000003549	Ercc1	20		ENSMUSG00000027737	Slc7a11	1
ENSMUSG00000030400	Ercc2	18		ENSMUSG00000069072	Slc7a14	4
ENSMUSG00000024382	Ercc3	20		ENSMUSG00000031596	Slc7a2	20
ENSMUSG00000022545	Ercc4	20		ENSMUSG00000031297	Slc7a3	18
ENSMUSG00000026048	Ercc5	12		ENSMUSG00000022756	Slc7a4	19
ENSMUSG00000054051	Ercc6	9		ENSMUSG00000040010	Slc7a5	20
ENSMUSG00000021470	Ercc6l2	20		ENSMUSG00000031904	Slc7a6	20

ENSMUSG00000021694	Ercc8	20		ENSMUSG00000033106	Slc7a6os	20
ENSMUSG00000040857	Erf	3		ENSMUSG00000000958	Slc7a7	20
ENSMUSG00000040732	Erg	20		ENSMUSG00000022180	Slc7a8	20
ENSMUSG00000021252	Erg28	20		ENSMUSG00000054640	Slc8a1	19
ENSMUSG00000001576	Ergic1	20		ENSMUSG00000030376	Slc8a2	9
ENSMUSG00000030304	Ergic2	20		ENSMUSG00000079055	Slc8a3	18
ENSMUSG00000005881	Ergic3	12		ENSMUSG00000032754	Slc8b1	14
ENSMUSG00000021131	Erh	12		ENSMUSG00000028854	Slc9a1	1
ENSMUSG00000031527	Eri1	20		ENSMUSG00000026062	Slc9a2	9
ENSMUSG00000030929	Eri2	20		ENSMUSG00000036123	Slc9a3	20
ENSMUSG00000033423	Eri3	10		ENSMUSG00000020733	Slc9a3r1	18
ENSMUSG00000051978	Erich1	20		ENSMUSG00000002504	Slc9a3r2	20
ENSMUSG00000075302	Erich2	20		ENSMUSG00000014786	Slc9a5	9
ENSMUSG00000078161	Erich3	20		ENSMUSG00000060681	Slc9a6	20
ENSMUSG00000044726	Erich5	9		ENSMUSG00000037341	Slc9a7	20
ENSMUSG00000070471	Erich6	20		ENSMUSG00000039463	Slc9a8	20
ENSMUSG00000020311	Erlec1	3		ENSMUSG00000031129	Slc9a9	1
ENSMUSG00000025198	Erlin1	3		ENSMUSG00000037994	Slc9b2	20
ENSMUSG00000031483	Erlin2	13		ENSMUSG00000030237	Slco1a4	1
ENSMUSG00000028644	Ermap	20		ENSMUSG00000030236	Slco1b2	20
ENSMUSG00000036552	Ermard	20		ENSMUSG00000030235	Slco1c1	13
ENSMUSG00000026830	Ermn	16		ENSMUSG00000032548	Slco2a1	18
ENSMUSG00000046324	Ermp1	20		ENSMUSG00000030737	Slco2b1	20
ENSMUSG00000020715	Ern1	20		ENSMUSG00000025790	Slco3a1	20
ENSMUSG00000030866	Ern2	20		ENSMUSG00000038963	Slco4a1	9
ENSMUSG00000021831	Ero1l	20		ENSMUSG00000040693	Slco4c1	20
ENSMUSG00000057069	Ero1lb	20		ENSMUSG00000025938	Slco5a1	18
ENSMUSG00000029616	Erp29	20		ENSMUSG00000021597	Slf1	7
ENSMUSG00000028343	Erp44	20		ENSMUSG00000036097	Slf2	20
ENSMUSG00000028967	Errfi1	20		ENSMUSG00000054404	Slfn5	20
ENSMUSG00000001946	Esam	20		ENSMUSG00000047518	Slfnl1	20
ENSMUSG00000024293	Esco1	20		ENSMUSG00000021040	Slirp	12
ENSMUSG00000021996	Esd	12		ENSMUSG00000025020	Slit1	5
ENSMUSG00000045624	Esf1	20		ENSMUSG00000031558	Slit2	20
ENSMUSG00000058290	Espl1	20		ENSMUSG00000056427	Slit3	9
ENSMUSG00000028943	Espn	20		ENSMUSG00000075478	Slitrk1	19
ENSMUSG00000019768	Esr1	1		ENSMUSG00000036790	Slitrk2	20
ENSMUSG00000024955	Esrra	20		ENSMUSG00000048304	Slitrk3	3
ENSMUSG00000021255	Esrrb	20		ENSMUSG00000046699	Slitrk4	4
ENSMUSG00000026610	Esrrg	16		ENSMUSG00000033214	Slitrk5	4
ENSMUSG00000003527	Ess2	20		ENSMUSG00000045871	Slitrk6	18
ENSMUSG00000025366	Esyt1	18		ENSMUSG00000025060	Slk	9
ENSMUSG00000021171	Esyt2	20		ENSMUSG00000021870	Slmap	9
ENSMUSG00000037681	Esyt3	20		ENSMUSG00000017002	Slpi	20
ENSMUSG00000016984	Etaa1	20		ENSMUSG00000032212	Sltm	20
ENSMUSG00000086468	Etaa1os	20		ENSMUSG00000020409	Slu7	20
ENSMUSG00000024360	Etf1	12		ENSMUSG00000059772	Slx1b	9
ENSMUSG00000032314	Etfa	20		ENSMUSG00000039738	Slx4	20
ENSMUSG00000004610	Etfb	12		ENSMUSG00000027281	Slx4ip	1
ENSMUSG00000039958	Etfbkmt	20		ENSMUSG00000031681	Smad1	9
ENSMUSG00000027809	Etfdh	20		ENSMUSG00000024563	Smad2	9
ENSMUSG00000040370	Etfrf1	11		ENSMUSG00000032402	Smad3	9
ENSMUSG00000064254	Ethe1	20		ENSMUSG00000024515	Smad4	7
ENSMUSG00000036617	Etl4	20		ENSMUSG00000021540	Smad5	20

ENSMUSG00000030275	Etnk1	7		ENSMUSG00000036867	Smad6	20
ENSMUSG00000070644	Etnk2	9		ENSMUSG00000025880	Smad7	16
ENSMUSG00000019232	Etnppl	1		ENSMUSG00000027796	Smad9	18
ENSMUSG00000089875	Etohd2	20		ENSMUSG00000053559	Smagp	20
ENSMUSG00000032035	Ets1	20		ENSMUSG00000026155	Smap1	20
ENSMUSG00000022895	Ets2	18		ENSMUSG00000032870	Smap2	6
ENSMUSG00000004151	Etv1	3		ENSMUSG00000031099	Smarca1	15
ENSMUSG00000003382	Etv3	20		ENSMUSG00000024921	Smarca2	9
ENSMUSG00000017724	Etv4	20		ENSMUSG00000032187	Smarca4	20
ENSMUSG00000013089	Etv5	7		ENSMUSG00000031715	Smarca5	20
ENSMUSG00000030199	Etv6	18		ENSMUSG00000052912	Smarca5-ps	20
ENSMUSG00000035104	Eva1a	20		ENSMUSG00000029920	Smarcad1	20
ENSMUSG00000050212	Eva1b	20		ENSMUSG00000039354	Smarcal1	20
ENSMUSG00000039903	Eva1c	20		ENSMUSG00000000902	Smarcb1	18
ENSMUSG00000029122	Evc	20		ENSMUSG00000032481	Smarcc1	9
ENSMUSG00000050248	Evc2	20		ENSMUSG00000025369	Smarcc2	18
ENSMUSG00000078771	Evi2a	16		ENSMUSG00000023018	Smarcd1	9
ENSMUSG00000011831	Evi5	20		ENSMUSG00000078619	Smarcd2	20
ENSMUSG00000011832	Evi5l	18		ENSMUSG00000028949	Smarcd3	1
ENSMUSG00000021262	Evl	20		ENSMUSG00000037935	Smarce1	20
ENSMUSG00000034282	Evpl	20		ENSMUSG00000094568	Smarce1-ps1	20
ENSMUSG00000009079	Ewsr1	18		ENSMUSG00000041133	Smc1a	9
ENSMUSG00000048647	Exd1	18		ENSMUSG00000022432	Smc1b	10
ENSMUSG00000032705	Exd2	18		ENSMUSG00000028312	Smc2	13
ENSMUSG00000039748	Exo1	9		ENSMUSG00000024974	Smc3	20
ENSMUSG00000028629	Exo5	20		ENSMUSG00000034349	Smc4	20
ENSMUSG00000036435	Exoc1	20		ENSMUSG00000024943	Smc5	20
ENSMUSG00000021357	Exoc2	20		ENSMUSG00000020608	Smc6	5
ENSMUSG00000034152	Exoc3	20		ENSMUSG00000024054	Smchd1	20
ENSMUSG00000043251	Exoc3l	20		ENSMUSG00000043298	Smco3	12
ENSMUSG00000021280	Exoc3l4	20		ENSMUSG00000058173	Smco4	20
ENSMUSG00000029763	Exoc4	20		ENSMUSG00000049323	Smcr8	2
ENSMUSG00000061244	Exoc5	2		ENSMUSG00000022452	Smdt1	12
ENSMUSG00000053799	Exoc6	6		ENSMUSG00000030655	Smg1	2
ENSMUSG00000033769	Exoc6b	2		ENSMUSG00000001415	Smg5	9
ENSMUSG00000020792	Exoc7	1		ENSMUSG00000038290	Smg6	20
ENSMUSG00000074030	Exoc8	20		ENSMUSG00000042772	Smg7	20
ENSMUSG00000042787	Exog	20		ENSMUSG00000020495	Smg8	20
ENSMUSG00000034321	Exosc1	20		ENSMUSG00000002210	Smg9	9
ENSMUSG00000017264	Exosc10	3		ENSMUSG00000078350	Smim1	2
ENSMUSG00000039356	Exosc2	20		ENSMUSG00000072704	Smim10l1	20
ENSMUSG00000028322	Exosc3	20		ENSMUSG00000054850	Smim10l2a	16
ENSMUSG00000034259	Exosc4	9		ENSMUSG00000051989	Smim11	12
ENSMUSG00000061286	Exosc5	20		ENSMUSG00000042380	Smim12	12
ENSMUSG00000025785	Exosc7	19		ENSMUSG00000091264	Smim13	3
ENSMUSG00000027752	Exosc8	20		ENSMUSG00000037822	Smim14	20
ENSMUSG00000027714	Exosc9	20		ENSMUSG00000071180	Smim15	12
ENSMUSG00000034584	Exph5	1		ENSMUSG00000093536	Smim17	20
ENSMUSG00000061731	Ext1	12		ENSMUSG00000094500	Smim18	20
ENSMUSG00000027198	Ext2	20		ENSMUSG00000031534	Smim19	20
ENSMUSG00000028838	Extl1	20		ENSMUSG00000061461	Smim20	20
ENSMUSG00000027963	Extl2	20		ENSMUSG00000078439	Smim24	20
ENSMUSG00000021978	Extl3	18		ENSMUSG00000074754	Smim26	12
ENSMUSG00000025932	Eya1	20		ENSMUSG00000028407	Smim27	20

ENSMUSG00000017897	Eya2	20		ENSMUSG00000038059	Smim3	19
ENSMUSG00000028886	Eya3	20		ENSMUSG00000058351	Smim4	12
ENSMUSG00000010461	Eya4	1		ENSMUSG00000048442	Smim5	12
ENSMUSG00000006920	Ezh1	10		ENSMUSG00000044600	Smim7	20
ENSMUSG00000029687	Ezh2	9		ENSMUSG00000028295	Smim8	1
ENSMUSG00000052397	Ezr	9		ENSMUSG00000021645	Smn1	20
ENSMUSG00000038235	F11r	20		ENSMUSG00000025024	Smndc1	20
ENSMUSG00000021492	F12	20		ENSMUSG00000001761	Smo	20
ENSMUSG00000039109	F13a1	20		ENSMUSG00000021136	Smoc1	9
ENSMUSG00000048376	F2r	18		ENSMUSG00000023886	Smoc2	5
ENSMUSG00000028128	F3	1		ENSMUSG00000027333	Smox	4
ENSMUSG00000097331	F420014N23Rik	20		ENSMUSG00000037049	Smpd1	9
ENSMUSG00000026579	F5	13		ENSMUSG00000019822	Smpd2	16
ENSMUSG00000097727	F630040K05Rik	9		ENSMUSG00000031906	Smpd3	9
ENSMUSG00000114442	F630042J09Rik	20		ENSMUSG00000005899	Smpd4	20
ENSMUSG00000052125	F730043M19Rik	5		ENSMUSG00000019872	Smpdl3a	5
ENSMUSG00000031196	F8	20		ENSMUSG00000028885	Smpdl3b	18
ENSMUSG00000090942	F830016B08Rik	20		ENSMUSG00000071708	Sms	9
ENSMUSG00000103159	F830112A20Rik	20		ENSMUSG00000081752	Sms-ps	9
ENSMUSG00000104576	F830115B05Rik	20		ENSMUSG00000082617	Smt3h2-ps	20
ENSMUSG00000103130	F830212C03Rik	12		ENSMUSG00000020439	Smtn	6
ENSMUSG00000078317	F8a	20		ENSMUSG00000045667	Smtnl2	20
ENSMUSG00000033579	Fa2h	16		ENSMUSG00000028409	Smu1	12
ENSMUSG00000034171	Faah	9		ENSMUSG00000036061	Smug1	1
ENSMUSG00000025384	Faap100	9		ENSMUSG00000038780	Smurf1	9
ENSMUSG00000073684	Faap20	20		ENSMUSG00000018363	Smurf2	20
ENSMUSG00000030493	Faap24	20		ENSMUSG00000055027	Smyd1	20
ENSMUSG00000028773	Fabp3	7		ENSMUSG00000026603	Smyd2	19
ENSMUSG00000056366	Fabp3-ps1	20		ENSMUSG00000055067	Smyd3	20
ENSMUSG00000027533	Fabp5	12		ENSMUSG00000018809	Smyd4	20
ENSMUSG00000019874	Fabp7	20		ENSMUSG00000033706	Smyd5	9
ENSMUSG00000031077	Fadd	20		ENSMUSG00000022676	Snai2	20
ENSMUSG00000010663	Fads1	1		ENSMUSG00000006587	Snai3	1
ENSMUSG00000024665	Fads2	1		ENSMUSG00000027287	Snap23	20
ENSMUSG00000024664	Fads3	13		ENSMUSG00000027273	Snap25	19
ENSMUSG00000044788	Fads6	9		ENSMUSG00000022765	Snap29	20
ENSMUSG00000010517	Faf1	1		ENSMUSG00000009894	Snap47	9
ENSMUSG00000025873	Faf2	20		ENSMUSG00000033419	Snap91	19
ENSMUSG00000030630	Fah	9		ENSMUSG00000021113	Snapc1	19
ENSMUSG00000045316	Fahd1	12		ENSMUSG00000011837	Snapc2	12
ENSMUSG00000027371	Fahd2a	1		ENSMUSG00000028483	Snapc3	20
ENSMUSG00000032463	Faim	20		ENSMUSG00000036281	Snapc4	20
ENSMUSG00000023011	Faim2	9		ENSMUSG00000032398	Snapc5	12
ENSMUSG00000039157	Fam102a	16		ENSMUSG00000001018	Snapin	20
ENSMUSG00000040339	Fam102b	9		ENSMUSG00000025889	Snca	18
ENSMUSG00000041629	Fam104a	17		ENSMUSG00000024534	Sncaip	20
ENSMUSG00000021750	Fam107a	1		ENSMUSG00000034891	Sncb	18
ENSMUSG00000026655	Fam107b	1		ENSMUSG00000023064	Sncg	18
ENSMUSG00000027459	Fam110a	20		ENSMUSG00000001424	Snd1	4
ENSMUSG00000049119	Fam110b	20		ENSMUSG00000047793	Sned1	9
ENSMUSG00000024691	Fam111a	20		ENSMUSG00000006058	Snf8	10
ENSMUSG00000029185	Fam114a1	1		ENSMUSG00000108414	Snhg1	1
ENSMUSG00000020523	Fam114a2	9		ENSMUSG00000113722	Snhg10	20
ENSMUSG00000038893	Fam117a	3		ENSMUSG00000044349	Snhg11	11

ENSMUSG00000041040	Fam117b	20		ENSMUSG00000086290	Snhg12	20
ENSMUSG00000022434	Fam118a	20		ENSMUSG00000100826	Snhg14	3
ENSMUSG00000050471	Fam118b	20		ENSMUSG00000085156	Snhg15	20
ENSMUSG00000038014	Fam120a	7		ENSMUSG00000020812	Snhg16	12
ENSMUSG00000014763	Fam120b	20		ENSMUSG00000085385	Snhg17	20
ENSMUSG00000025262	Fam120c	2		ENSMUSG00000096956	Snhg18	20
ENSMUSG00000074922	Fam122a	20		ENSMUSG00000086859	Snhg20	12
ENSMUSG00000036022	Fam122b	20		ENSMUSG00000085241	Snhg3	20
ENSMUSG00000035184	Fam124a	1		ENSMUSG00000117869	Snhg4	15
ENSMUSG00000028995	Fam126a	1		ENSMUSG00000117694	Snhg4	20
ENSMUSG00000038174	Fam126b	3		ENSMUSG00000097195	Snhg5	20
ENSMUSG00000026483	Fam129a	20		ENSMUSG00000098234	Snhg6	12
ENSMUSG00000026796	Fam129b	20		ENSMUSG00000104960	Snhg8	12
ENSMUSG00000043243	Fam129c	20		ENSMUSG00000090101	Snhg9	20
ENSMUSG00000050821	Fam131a	20		ENSMUSG00000050213	Snip1	20
ENSMUSG00000029861	Fam131b	20		ENSMUSG00000037972	Snn	9
ENSMUSG00000006218	Fam131c	18		ENSMUSG00000077192	Snora17	10
ENSMUSG00000058503	Fam133b	20		ENSMUSG00000095530	Snora19	20
ENSMUSG00000026153	Fam135a	2		ENSMUSG00000064637	Snora20	10
ENSMUSG00000036800	Fam135b	2		ENSMUSG00000064901	Snora21	10
ENSMUSG00000057497	Fam136a	20		ENSMUSG00000064451	Snora23	10
ENSMUSG00000037709	Fam13a	9		ENSMUSG00000065259	Snora30	10
ENSMUSG00000036501	Fam13b	9		ENSMUSG00000065147	Snora31	10
ENSMUSG00000043259	Fam13c	5		ENSMUSG00000077767	Snora35	10
ENSMUSG00000070044	Fam149a	18		ENSMUSG00000077571	Snora36b	20
ENSMUSG00000039599	Fam149b	20		ENSMUSG00000064602	Snora41	10
ENSMUSG00000034334	Fam151b	20		ENSMUSG00000088108	Snora47	10
ENSMUSG00000079157	Fam155a	19		ENSMUSG00000070167	Snora57	10
ENSMUSG00000051000	Fam160a1	13		ENSMUSG00000064925	Snora62	10
ENSMUSG00000044465	Fam160a2	2		ENSMUSG00000065642	Snora69	10
ENSMUSG00000033478	Fam160b1	20		ENSMUSG00000064387	Snora73a	10
ENSMUSG00000022095	Fam160b2	9		ENSMUSG00000065353	Snora73b	10
ENSMUSG00000049811	Fam161a	13		ENSMUSG00000089255	Snora78	10
ENSMUSG00000021234	Fam161b	20		ENSMUSG00000065126	Snord104	10
ENSMUSG00000003955	Fam162a	12		ENSMUSG00000089093	Snord11	10
ENSMUSG00000015484	Fam163a	18		ENSMUSG00000080610	Snord110	10
ENSMUSG00000009216	Fam163b	9		ENSMUSG00000094274	Snord116l12	20
ENSMUSG00000042788	Fam166b	20		ENSMUSG00000064899	Snord118	10
ENSMUSG00000035095	Fam167a	19		ENSMUSG00000088252	Snord13	10
ENSMUSG00000029461	Fam168a	12		ENSMUSG00000065822	Snord15a	10
ENSMUSG00000037503	Fam168b	20		ENSMUSG00000077714	Snord17	10
ENSMUSG00000041817	Fam169a	3		ENSMUSG00000080478	Snord23	10
ENSMUSG00000074071	Fam169b	20		ENSMUSG00000065219	Snord32a	10
ENSMUSG00000050530	Fam171a1	9		ENSMUSG00000065878	Snord34	10
ENSMUSG00000034685	Fam171a2	20		ENSMUSG00000065818	Snord35a	10
ENSMUSG00000048388	Fam171b	20		ENSMUSG00000065734	Snord49a	10
ENSMUSG00000064138	Fam172a	7		ENSMUSG00000064437	Snord49b	10
ENSMUSG00000057411	Fam173a	20		ENSMUSG00000092680	Snord55	10
ENSMUSG00000051185	Fam174a	20		ENSMUSG00000064871	Snord58b	2
ENSMUSG00000078670	Fam174b	20		ENSMUSG00000064778	Snord59a	10
ENSMUSG00000095595	Fam177a	20		ENSMUSG00000065110	Snord61	10
ENSMUSG00000047420	Fam180a	20		ENSMUSG00000077457	Snord65	10
ENSMUSG00000096753	Fam181a	20		ENSMUSG00000077239	Snord66	10
ENSMUSG00000051515	Fam181b	20		ENSMUSG00000077345	Snord70	20

ENSMUSG00000049154	Fam183b	20		ENSMUSG00000077549	Snord71	20
ENSMUSG00000019856	Fam184a	9		ENSMUSG00000093044	Snord8	10
ENSMUSG00000015879	Fam184b	9		ENSMUSG00000064823	Snord82	10
ENSMUSG00000047221	Fam185a	9		ENSMUSG00000077734	Snord83b	10
ENSMUSG00000045350	Fam186a	20		ENSMUSG00000093178	Snord87	10
ENSMUSG00000078907	Fam186b	9		ENSMUSG00000077704	Snord89	10
ENSMUSG00000030518	Fam189a1	9		ENSMUSG00000077756	Snord90	10
ENSMUSG00000071604	Fam189a2	18		ENSMUSG00000077493	Snord91a	20
ENSMUSG00000032657	Fam189b	20		ENSMUSG00000027457	Snph	8
ENSMUSG00000031774	Fam192a	20		ENSMUSG00000038145	Snrk	18
ENSMUSG00000037210	Fam193a	2		ENSMUSG00000003660	Snrnp200	7
ENSMUSG00000021495	Fam193b	20		ENSMUSG00000040767	Snrnp25	20
ENSMUSG00000042595	Fam199x	19		ENSMUSG00000001158	Snrnp27	12
ENSMUSG00000057858	Fam204a	20		ENSMUSG00000029402	Snrnp35	20
ENSMUSG00000032977	Fam207a	20		ENSMUSG00000021431	Snrnp48	1
ENSMUSG00000020614	Fam20a	20		ENSMUSG00000063511	Snrnp70	20
ENSMUSG00000033557	Fam20b	18		ENSMUSG00000061479	Snrpa	20
ENSMUSG00000025854	Fam20c	18		ENSMUSG00000030512	Snrpa1	20
ENSMUSG00000038121	Fam210a	3		ENSMUSG00000027404	Snrpb	20
ENSMUSG00000027495	Fam210b	20		ENSMUSG00000008333	Snrpb2	20
ENSMUSG00000034858	Fam214a	9		ENSMUSG00000024217	Snrpc	20
ENSMUSG00000036002	Fam214b	9		ENSMUSG00000002477	Snrpd1	3
ENSMUSG00000029463	Fam216a	9		ENSMUSG00000040824	Snrpd2	10
ENSMUSG00000045655	Fam216b	14		ENSMUSG00000020180	Snrpd3	20
ENSMUSG00000021414	Fam217a	20		ENSMUSG00000090553	Snrpe	12
ENSMUSG00000070476	Fam217b	20		ENSMUSG00000082044	Snrpert	12
ENSMUSG00000028439	Fam219a	20		ENSMUSG00000020018	Snrpf	12
ENSMUSG00000032305	Fam219b	20		ENSMUSG00000057278	Snrpg	20
ENSMUSG00000083012	Fam220a	9		ENSMUSG00000102252	Snrpn	9
ENSMUSG00000047115	Fam221a	20		ENSMUSG00000027488	Snta1	20
ENSMUSG00000043633	Fam221b	20		ENSMUSG00000060429	Sntb1	9
ENSMUSG00000041930	Fam222a	1		ENSMUSG00000041308	Sntb2	9
ENSMUSG00000037750	Fam222b	20		ENSMUSG00000025909	Sntg1	20
ENSMUSG00000042564	Fam227a	16		ENSMUSG00000020672	Sntg2	1
ENSMUSG00000079177	Fam228a	20		ENSMUSG00000044772	Sntn	20
ENSMUSG00000050545	Fam228b	19		ENSMUSG00000063480	Snu13	20
ENSMUSG00000051736	Fam229b	20		ENSMUSG00000055334	Snupn	20
ENSMUSG00000024187	Fam234a	16		ENSMUSG00000021039	Snw1	9
ENSMUSG00000030207	Fam234b	20		ENSMUSG00000032382	Snx1	20
ENSMUSG00000050549	Fam241a	20		ENSMUSG00000038301	Snx10	1
ENSMUSG00000020083	Fam241b	20		ENSMUSG00000020876	Snx11	20
ENSMUSG00000003039	Fam32a	9		ENSMUSG00000046032	Snx12	3
ENSMUSG00000031399	Fam3a	20		ENSMUSG00000020590	Snx13	20
ENSMUSG00000029672	Fam3c	3		ENSMUSG00000032422	Snx14	20
ENSMUSG00000046546	Fam43a	4		ENSMUSG00000024787	Snx15	20
ENSMUSG00000078235	Fam43b	20		ENSMUSG00000027534	Snx16	20
ENSMUSG00000024993	Fam45a	20		ENSMUSG00000029146	Snx17	20
ENSMUSG00000020589	Fam49a	9		ENSMUSG00000042364	Snx18	20
ENSMUSG00000022378	Fam49b	20		ENSMUSG00000031993	Snx19	20
ENSMUSG00000001962	Fam50a	20		ENSMUSG00000034484	Snx2	20
ENSMUSG00000037339	Fam53a	20		ENSMUSG00000031662	Snx20	20
ENSMUSG00000030956	Fam53b	16		ENSMUSG00000050373	Snx21	9
ENSMUSG00000034300	Fam53c	9		ENSMUSG00000039452	Snx22	20
ENSMUSG00000069808	Fam57a	20		ENSMUSG00000024535	Snx24	20

ENSMUSG00000058966	Fam57b	9		ENSMUSG00000038291	Snx25	20
ENSMUSG00000056987	Fam71d	20		ENSMUSG00000028136	Snx27	20
ENSMUSG00000051113	Fam71e1	20		ENSMUSG00000071669	Snx29	9
ENSMUSG00000079652	Fam71f2	20		ENSMUSG00000019804	Snx3	19
ENSMUSG00000055184	Fam72a	3		ENSMUSG00000028385	Snx30	9
ENSMUSG00000028878	Fam76a	20		ENSMUSG00000013611	Snx31	9
ENSMUSG00000037808	Fam76b	20		ENSMUSG00000056185	Snx32	11
ENSMUSG00000050592	Fam78a	9		ENSMUSG00000032733	Snx33	1
ENSMUSG00000060568	Fam78b	6		ENSMUSG00000022808	Snx4	5
ENSMUSG00000032224	Fam81a	5		ENSMUSG00000027423	Snx5	20
ENSMUSG00000032358	Fam83b	9		ENSMUSG00000005656	Snx6	20
ENSMUSG00000027654	Fam83d	1		ENSMUSG00000028007	Snx7	1
ENSMUSG00000022408	Fam83f	9		ENSMUSG00000029560	Snx8	18
ENSMUSG00000046761	Fam83h	18		ENSMUSG00000002365	Snx9	9
ENSMUSG00000043068	Fam89a	20		ENSMUSG00000026600	Soat1	20
ENSMUSG00000069237	Fam8a1	3		ENSMUSG00000038248	Sobp	19
ENSMUSG00000028218	Fam92a	18		ENSMUSG00000020027	Socs2	20
ENSMUSG00000002017	Fam98a	20		ENSMUSG00000053113	Socs3	20
ENSMUSG00000027349	Fam98b	20		ENSMUSG00000048379	Socs4	20
ENSMUSG00000030590	Fam98c	3		ENSMUSG00000037104	Socs5	9
ENSMUSG00000033458	Fan1	20		ENSMUSG00000056153	Socs6	18
ENSMUSG00000032815	Fanca	12		ENSMUSG00000038485	Socs7	9
ENSMUSG00000047757	Fancb	9		ENSMUSG00000022982	Sod1	3
ENSMUSG00000021461	Fancc	12		ENSMUSG00000006818	Sod2	3
ENSMUSG00000034023	Fancd2	20		ENSMUSG00000072941	Sod3	13
ENSMUSG00000007570	Fance	20		ENSMUSG00000055485	Soga1	9
ENSMUSG00000092118	Fancf	9		ENSMUSG00000038916	Soga3	2
ENSMUSG00000028453	Fancg	20		ENSMUSG00000022961	Son	4
ENSMUSG00000039187	Fanci	20		ENSMUSG00000025006	Sorbs1	9
ENSMUSG00000004018	Fancl	20		ENSMUSG00000031626	Sorbs2	9
ENSMUSG00000055884	Fancm	20		ENSMUSG00000022091	Sorbs3	16
ENSMUSG00000053111	Fank1	20		ENSMUSG00000043531	Sorcs1	6
ENSMUSG00000000392	Fap	13		ENSMUSG00000029093	Sorcs2	8
ENSMUSG00000030759	Far1	7		ENSMUSG00000063434	Sorcs3	20
ENSMUSG00000030303	Far2	18		ENSMUSG00000027227	Sord	9
ENSMUSG00000089948	Far2os1	20		ENSMUSG00000049313	Sorl1	2
ENSMUSG00000086777	Far2os2	20		ENSMUSG00000068747	Sort1	9
ENSMUSG00000025555	Farp1	18		ENSMUSG00000024241	Sos1	1
ENSMUSG00000034066	Farp2	20		ENSMUSG00000034801	Sos2	20
ENSMUSG00000021420	Fars2	20		ENSMUSG00000036169	Sostdc1	13
ENSMUSG00000003808	Farsa	20		ENSMUSG00000044352	Sowaha	9
ENSMUSG00000026245	Farsb	19		ENSMUSG00000045314	Sowahb	18
ENSMUSG00000024778	Fas	20		ENSMUSG00000098188	Sowahc	20
ENSMUSG00000025153	Fasn	12		ENSMUSG00000096014	Sox1	20
ENSMUSG00000028959	Fastk	20		ENSMUSG00000033006	Sox10	16
ENSMUSG00000027086	Fastkd1	3		ENSMUSG00000063632	Sox11	1
ENSMUSG00000025962	Fastkd2	20		ENSMUSG00000051817	Sox12	20
ENSMUSG00000021532	Fastkd3	20		ENSMUSG00000070643	Sox13	18
ENSMUSG00000079043	Fastkd5	20		ENSMUSG00000025902	Sox17	20
ENSMUSG00000070047	Fat1	9		ENSMUSG00000046470	Sox18	20
ENSMUSG00000074505	Fat3	7		ENSMUSG00000047935	Sox1ot	1
ENSMUSG00000046743	Fat4	5		ENSMUSG00000074637	Sox2	12
ENSMUSG00000038274	Fau	3		ENSMUSG00000061517	Sox21	20
ENSMUSG00000028246	Faxc	3		ENSMUSG00000105265	Sox2ot	20

ENSMUSG00000020776	Fbf1	1		ENSMUSG00000045179	Sox3	20
ENSMUSG00000058594	Fbh1	20		ENSMUSG00000076431	Sox4	14
ENSMUSG00000046865	Fbl	20		ENSMUSG00000041540	Sox5	9
ENSMUSG00000006219	Fblim1	20		ENSMUSG00000072677	Sox5it	20
ENSMUSG00000051062	Fbll1	12		ENSMUSG00000051910	Sox6	20
ENSMUSG00000006369	Fbln1	13		ENSMUSG00000063060	Sox7	20
ENSMUSG00000064080	Fbln2	20		ENSMUSG00000024176	Sox8	20
ENSMUSG00000021186	Fbln5	20		ENSMUSG00000000567	Sox9	20
ENSMUSG00000027386	Fbln7	14		ENSMUSG00000001280	Sp1	20
ENSMUSG00000027204	Fbn1	20		ENSMUSG00000026222	Sp100	13
ENSMUSG00000024598	Fbn2	20		ENSMUSG00000070034	Sp110	20
ENSMUSG00000042423	Fbrs	20		ENSMUSG00000070031	Sp140	20
ENSMUSG00000043323	Fbrsl1	20		ENSMUSG00000018678	Sp2	20
ENSMUSG00000066892	Fbxl12	20		ENSMUSG00000027109	Sp3	20
ENSMUSG00000062470	Fbxl12os	20		ENSMUSG00000063714	Sp3os	9
ENSMUSG00000048520	Fbxl13	13		ENSMUSG00000025323	Sp4	2
ENSMUSG00000030019	Fbxl14	12		ENSMUSG00000038560	Sp6	20
ENSMUSG00000025226	Fbxl15	19		ENSMUSG00000060284	Sp7	20
ENSMUSG00000025738	Fbxl16	9		ENSMUSG00000048562	Sp8	12
ENSMUSG00000023965	Fbxl17	3		ENSMUSG00000068859	Sp9	19
ENSMUSG00000066640	Fbxl18	20		ENSMUSG00000001948	Spa17	20
ENSMUSG00000030811	Fbxl19	20		ENSMUSG00000028475	Spaar	20
ENSMUSG00000032507	Fbxl2	20		ENSMUSG00000080316	Spaca6	1
ENSMUSG00000020883	Fbxl20	20		ENSMUSG00000037617	Spag1	20
ENSMUSG00000035509	Fbxl21	1		ENSMUSG00000053153	Spag16	13
ENSMUSG00000050503	Fbxl22	20		ENSMUSG00000027867	Spag17	14
ENSMUSG00000022124	Fbxl3	20		ENSMUSG00000038180	Spag4	18
ENSMUSG00000040410	Fbxl4	6		ENSMUSG00000002055	Spag5	19
ENSMUSG00000039753	Fbxl5	20		ENSMUSG00000037708	Spag6	1
ENSMUSG00000022559	Fbxl6	20		ENSMUSG00000022783	Spag6l	20
ENSMUSG00000043556	Fbxl7	20		ENSMUSG00000018287	Spag7	20
ENSMUSG00000033313	Fbxl8	20		ENSMUSG00000066196	Spag8	14
ENSMUSG00000048232	Fbxo10	5		ENSMUSG00000020859	Spag9	3
ENSMUSG00000005371	Fbxo11	20		ENSMUSG00000018593	Sparc	18
ENSMUSG00000034532	Fbxo16	9		ENSMUSG00000029309	Sparcl1	18
ENSMUSG00000030598	Fbxo17	20		ENSMUSG00000024068	Spast	3
ENSMUSG00000041556	Fbxo2	20		ENSMUSG00000028188	Spata1	1
ENSMUSG00000032898	Fbxo21	20		ENSMUSG00000021990	Spata13	9
ENSMUSG00000032309	Fbxo22	20		ENSMUSG00000026611	Spata17	20
ENSMUSG00000038365	Fbxo25	18		ENSMUSG00000029155	Spata18	14
ENSMUSG00000037463	Fbxo27	20		ENSMUSG00000031991	Spata19	20
ENSMUSG00000047539	Fbxo28	2		ENSMUSG00000047030	Spata2	3
ENSMUSG00000027180	Fbxo3	18		ENSMUSG00000045004	Spata21	20
ENSMUSG00000047648	Fbxo30	20		ENSMUSG00000024352	Spata24	16
ENSMUSG00000052934	Fbxo31	20		ENSMUSG00000033594	Spata2l	9
ENSMUSG00000035329	Fbxo33	19		ENSMUSG00000026226	Spata3	20
ENSMUSG00000037536	Fbxo34	5		ENSMUSG00000044787	Spata32	20
ENSMUSG00000073633	Fbxo36	20		ENSMUSG00000048478	Spata33	1
ENSMUSG00000042211	Fbxo38	20		ENSMUSG00000020191	Spata48	20
ENSMUSG00000022184	Fbxo4	20		ENSMUSG00000027722	Spata5	20
ENSMUSG00000047746	Fbxo40	18		ENSMUSG00000034401	Spata6	17
ENSMUSG00000047013	Fbxo41	4		ENSMUSG00000021007	Spata7	20
ENSMUSG00000028920	Fbxo42	20		ENSMUSG00000023935	Spats1	20
ENSMUSG00000029001	Fbxo44	20		ENSMUSG00000051934	Spats2	20

ENSMUSG00000035764	Fbxo45	20		ENSMUSG00000038305	Spats2l	9
ENSMUSG00000050428	Fbxo46	20		ENSMUSG00000074476	Spc24	20
ENSMUSG00000070336	Fbxo47	20		ENSMUSG00000005233	Spc25	20
ENSMUSG00000044966	Fbxo48	20		ENSMUSG00000021917	Spcs1	20
ENSMUSG00000019773	Fbxo5	20		ENSMUSG00000035227	Spcs2	20
ENSMUSG00000055401	Fbxo6	19		ENSMUSG00000081888	Spcs2-ps	20
ENSMUSG00000001786	Fbxo7	20		ENSMUSG00000054408	Spcs3	9
ENSMUSG00000038206	Fbxo8	20		ENSMUSG00000024215	Spdef	20
ENSMUSG00000001366	Fbxo9	19		ENSMUSG00000069910	Spdl1	20
ENSMUSG00000090173	Fbxw10	20		ENSMUSG00000052525	Spdya	20
ENSMUSG00000020271	Fbxw11	20		ENSMUSG00000042331	Specc1	20
ENSMUSG00000037816	Fbxw17	20		ENSMUSG00000033444	Specc1l	20
ENSMUSG00000035949	Fbxw2	20		ENSMUSG00000027329	Spef1	20
ENSMUSG00000104572	Fbxw23	1		ENSMUSG00000072663	Spef2	13
ENSMUSG00000040913	Fbxw4	20		ENSMUSG00000026207	Speg	9
ENSMUSG00000015095	Fbxw5	20		ENSMUSG00000040761	Spen	20
ENSMUSG00000028086	Fbxw7	9		ENSMUSG00000033396	Spg11	9
ENSMUSG00000032867	Fbxw8	20		ENSMUSG00000036580	Spg20	18
ENSMUSG00000008167	Fbxw9	20		ENSMUSG00000032388	Spg21	3
ENSMUSG00000058715	Fcer1g	5		ENSMUSG00000000738	Spg7	7
ENSMUSG00000021243	Fcf1	10		ENSMUSG00000061878	Sphk1	20
ENSMUSG00000015947	Fcgr1	20		ENSMUSG00000057342	Sphk2	20
ENSMUSG00000026656	Fcgr2b	15		ENSMUSG00000026163	Sphkap	5
ENSMUSG00000059498	Fcgr3	20		ENSMUSG00000002111	Spi1	20
ENSMUSG00000003420	Fcgrt	9		ENSMUSG00000043065	Spice1	20
ENSMUSG00000070000	Fcho1	3		ENSMUSG00000041974	Spidr	20
ENSMUSG00000041685	Fcho2	20		ENSMUSG00000021395	Spin1	2
ENSMUSG00000038524	Fchsd1	9		ENSMUSG00000046550	Spin2c	20
ENSMUSG00000030691	Fchsd2	20		ENSMUSG00000071722	Spin4	12
ENSMUSG00000052403	Fcnaos	20		ENSMUSG00000024970	Spindoc	20
ENSMUSG00000089665	Fcor	20		ENSMUSG00000044176	Spink10	18
ENSMUSG00000070524	Fcrlb	20		ENSMUSG00000050074	Spink8	4
ENSMUSG00000015852	Fcrls	18		ENSMUSG00000027315	Spint1	9
ENSMUSG00000033703	Fcsk	20		ENSMUSG00000074227	Spint2	13
ENSMUSG00000021273	Fdft1	12		ENSMUSG00000024533	Spire1	19
ENSMUSG00000059743	Fdps	20		ENSMUSG00000010154	Spire2	9
ENSMUSG00000032051	Fdx1	20		ENSMUSG00000030741	Spns1	3
ENSMUSG00000079677	Fdx2	20		ENSMUSG00000040447	Spns2	20
ENSMUSG00000037845	Fdxacb1	2		ENSMUSG00000020798	Spns3	20
ENSMUSG00000018861	Fdxr	7		ENSMUSG00000028784	Spocd1	20
ENSMUSG00000024588	Fech	5		ENSMUSG00000056222	Spock1	18
ENSMUSG00000043683	Fem1a	10		ENSMUSG00000058297	Spock2	18
ENSMUSG00000032244	Fem1b	2		ENSMUSG00000054162	Spock3	9
ENSMUSG00000033319	Fem1c	12		ENSMUSG00000038156	Spon1	19
ENSMUSG00000024742	Fen1	20		ENSMUSG00000057522	Spop	20
ENSMUSG00000097336	Fendrr	20		ENSMUSG00000026771	Spopl	2
ENSMUSG00000000127	Fer	20		ENSMUSG00000039660	Spout1	3
ENSMUSG00000037712	Fermt2	20		ENSMUSG00000029304	Spp1	16
ENSMUSG00000024965	Fermt3	20		ENSMUSG00000027366	Sppl2a	7
ENSMUSG00000053158	Fes	20		ENSMUSG00000035206	Sppl2b	9
ENSMUSG00000032118	Fez1	3		ENSMUSG00000029550	Sppl3	20
ENSMUSG00000056121	Fez2	9		ENSMUSG00000033735	Spr	12
ENSMUSG00000021743	Fezf2	20		ENSMUSG00000027351	Spred1	7
ENSMUSG00000025265	Fgd1	1		ENSMUSG00000045671	Spred2	9

ENSMUSG00000024013	Fgd2	20		ENSMUSG00000037239	Spred3	20
ENSMUSG00000037946	Fgd3	18		ENSMUSG00000045733	Sprn	19
ENSMUSG00000022788	Fgd4	1		ENSMUSG00000050359	Sprr1a	1
ENSMUSG00000034037	Fgd5	20		ENSMUSG00000031986	Sprtn	20
ENSMUSG00000020021	Fgd6	19		ENSMUSG00000037211	Spry1	20
ENSMUSG00000036585	Fgf1	15		ENSMUSG00000022114	Spry2	9
ENSMUSG00000021732	Fgf10	19		ENSMUSG00000061654	Spry3	20
ENSMUSG00000042826	Fgf11	20		ENSMUSG00000024427	Spry4	9
ENSMUSG00000022523	Fgf12	18		ENSMUSG00000036966	Spryd3	12
ENSMUSG00000031137	Fgf13	5		ENSMUSG00000051346	Spryd4	20
ENSMUSG00000025551	Fgf14	7		ENSMUSG00000021930	Spryd7	20
ENSMUSG00000031230	Fgf16	9		ENSMUSG00000039911	Spsb1	20
ENSMUSG00000057967	Fgf18	18		ENSMUSG00000038451	Spsb2	20
ENSMUSG00000037225	Fgf2	9		ENSMUSG00000024160	Spsb3	20
ENSMUSG00000031603	Fgf20	20		ENSMUSG00000046997	Spsb4	18
ENSMUSG00000020327	Fgf22	9		ENSMUSG00000057738	Sptan1	12
ENSMUSG00000000182	Fgf23	9		ENSMUSG00000021061	Sptb	9
ENSMUSG00000031074	Fgf3	19		ENSMUSG00000020315	Sptbn1	19
ENSMUSG00000029337	Fgf5	5		ENSMUSG00000067889	Sptbn2	9
ENSMUSG00000027208	Fgf7	9		ENSMUSG00000011751	Sptbn4	9
ENSMUSG00000021974	Fgf9	18		ENSMUSG00000074899	Sptbn5	1
ENSMUSG00000047632	Fgfbp3	20		ENSMUSG00000021468	Sptlc1	20
ENSMUSG00000031565	Fgfr1	5		ENSMUSG00000021036	Sptlc2	18
ENSMUSG00000069135	Fgfr1op	20		ENSMUSG00000044408	Sptssa	20
ENSMUSG00000040242	Fgfr1op2	9		ENSMUSG00000043461	Sptssb	18
ENSMUSG00000030849	Fgfr2	16		ENSMUSG00000049516	Spty2d1	20
ENSMUSG00000054252	Fgfr3	20		ENSMUSG00000071112	Spx	20
ENSMUSG00000005320	Fgfr4	1		ENSMUSG00000022351	Sqle	9
ENSMUSG00000008090	Fgfrl1	20		ENSMUSG00000005803	Sqor	19
ENSMUSG00000028573	Fggy	20		ENSMUSG00000015837	Sqstm1	20
ENSMUSG00000031594	Fgl1	20		ENSMUSG00000006050	Sra1	12
ENSMUSG00000039899	Fgl2	13		ENSMUSG00000070637	Srarp	20
ENSMUSG00000028874	Fgr	20		ENSMUSG00000024135	Srbd1	20
ENSMUSG00000026526	Fh1	20		ENSMUSG00000027646	Src	19
ENSMUSG00000051435	Fhad1	14		ENSMUSG00000053877	Srcap	20
ENSMUSG00000041842	Fhdc1	18		ENSMUSG00000038453	Srcin1	19
ENSMUSG00000060579	Fhit	20		ENSMUSG00000021594	Srd5a1	20
ENSMUSG00000023092	Fhl1	2		ENSMUSG00000029233	Srd5a3	18
ENSMUSG00000008136	Fhl2	4		ENSMUSG00000020538	Srebf1	20
ENSMUSG00000032643	Fhl3	9		ENSMUSG00000022463	Srebf2	20
ENSMUSG00000050035	Fhl4	20		ENSMUSG00000032621	Srek1	20
ENSMUSG00000014778	Fhod1	20		ENSMUSG00000021716	Srek1ip1	20
ENSMUSG00000034295	Fhod3	18		ENSMUSG00000015605	Srf	9
ENSMUSG00000026841	Fibcd1	9		ENSMUSG00000024528	Srfbp1	20
ENSMUSG00000074971	Fibin	20		ENSMUSG00000020121	Srgap1	20
ENSMUSG00000024911	Fibp	20		ENSMUSG00000026425	Srgap2	3
ENSMUSG00000053334	Ficd	20		ENSMUSG00000030257	Srgap3	4
ENSMUSG00000038417	Fig4	9		ENSMUSG00000020077	Srgn	9
ENSMUSG00000075324	Fign	18		ENSMUSG00000003161	Sri	20
ENSMUSG00000035455	Fignl1	20		ENSMUSG00000022519	Srl	1
ENSMUSG00000095440	Fignl2	20		ENSMUSG00000006442	Srm	19
ENSMUSG00000034898	Filip1	9		ENSMUSG00000009549	Srp14	12
ENSMUSG00000043336	Filip1l	20		ENSMUSG00000014504	Srp19	18
ENSMUSG00000029227	Fip1l1	20		ENSMUSG00000073079	Srp54a	20

ENSMUSG00000085396	Firre	8		ENSMUSG00000112449	Srp54b	20
ENSMUSG00000019054	Fis1	12		ENSMUSG00000079108	Srp54c	20
ENSMUSG00000048486	Fitm2	18		ENSMUSG00000020780	Srp68	20
ENSMUSG00000061374	Fiz1	3		ENSMUSG00000036323	Srp72	20
ENSMUSG00000075012	Fjx1	20		ENSMUSG00000026511	Srp9	12
ENSMUSG00000001555	Fkbp10	20		ENSMUSG00000004865	Srpk1	9
ENSMUSG00000003355	Fkbp11	12		ENSMUSG00000062604	Srpk2	18
ENSMUSG00000038074	Fkbp14	20		ENSMUSG00000002007	Srpk3	20
ENSMUSG00000066151	Fkbp15	20		ENSMUSG00000032042	Srpr	20
ENSMUSG00000032966	Fkbp1a	18		ENSMUSG00000032553	Srprb	20
ENSMUSG00000020635	Fkbp1b	9		ENSMUSG00000090084	Srpx	11
ENSMUSG00000056629	Fkbp2	10		ENSMUSG00000031253	Srpx2	18
ENSMUSG00000020949	Fkbp3	20		ENSMUSG00000001323	Srr	9
ENSMUSG00000030357	Fkbp4	9		ENSMUSG00000029346	Srrd	20
ENSMUSG00000024222	Fkbp5	18		ENSMUSG00000028809	Srrm1	20
ENSMUSG00000002732	Fkbp7	9		ENSMUSG00000039218	Srrm2	19
ENSMUSG00000019428	Fkbp8	3		ENSMUSG00000039860	Srrm3	20
ENSMUSG00000029781	Fkbp9	20		ENSMUSG00000063919	Srrm4	19
ENSMUSG00000033739	Fkbpl	20		ENSMUSG00000037364	Srrt	1
ENSMUSG00000048920	Fkrp	20		ENSMUSG00000018379	Srsf1	5
ENSMUSG00000028414	Fktn	20		ENSMUSG00000028676	Srsf10	20
ENSMUSG00000042642	Flad1	18		ENSMUSG00000055436	Srsf11	20
ENSMUSG00000032633	Flcn	16		ENSMUSG00000054679	Srsf12	16
ENSMUSG00000016087	Fli1	20		ENSMUSG00000034120	Srsf2	9
ENSMUSG00000002812	Flii	18		ENSMUSG00000071172	Srsf3	20
ENSMUSG00000031328	Flna	20		ENSMUSG00000028911	Srsf4	20
ENSMUSG00000025278	Flnb	6		ENSMUSG00000021134	Srsf5	20
ENSMUSG00000068699	Flnc	1		ENSMUSG00000016921	Srsf6	1
ENSMUSG00000059714	Flot1	7		ENSMUSG00000024097	Srsf7	3
ENSMUSG00000061981	Flot2	20		ENSMUSG00000029538	Srsf9	12
ENSMUSG00000047787	Flrt1	9		ENSMUSG00000032802	Srxn1	2
ENSMUSG00000047414	Flrt2	2		ENSMUSG00000037013	Ss18	20
ENSMUSG00000051379	Flrt3	9		ENSMUSG00000039086	Ss18l1	2
ENSMUSG00000029648	Flt1	20		ENSMUSG00000032526	Ss18l2	12
ENSMUSG00000042817	Flt3	18		ENSMUSG00000068882	Ssb	20
ENSMUSG00000110206	Flt3l	1		ENSMUSG00000029911	Ssbp1	18
ENSMUSG00000020357	Flt4	20		ENSMUSG00000003992	Ssbp2	19
ENSMUSG00000066595	Flvcr1	20		ENSMUSG00000061887	Ssbp3	20
ENSMUSG00000034258	Flvcr2	20		ENSMUSG00000070003	Ssbp4	3
ENSMUSG00000040097	Flywch1	18		ENSMUSG00000029699	Ssc4d	20
ENSMUSG00000023911	Flywch2	20		ENSMUSG00000035279	Ssc5d	20
ENSMUSG00000019689	Fmc1	12		ENSMUSG00000042121	Ssh1	9
ENSMUSG00000044042	Fmn1	19		ENSMUSG00000037926	Ssh2	3
ENSMUSG00000028354	Fmn2	3		ENSMUSG00000034616	Ssh3	20
ENSMUSG00000055805	Fmnl1	9		ENSMUSG00000026966	Ssna1	12
ENSMUSG00000036053	Fmnl2	18		ENSMUSG00000030255	Sspn	20
ENSMUSG00000023008	Fmnl3	20		ENSMUSG00000029797	Sspo	9
ENSMUSG00000040181	Fmo1	18		ENSMUSG00000021427	Ssr1	20
ENSMUSG00000040170	Fmo2	20		ENSMUSG00000041355	Ssr2	20
ENSMUSG00000028088	Fmo5	1		ENSMUSG00000027828	Ssr3	3
ENSMUSG00000041559	Fmod	19		ENSMUSG00000002014	Ssr4	12
ENSMUSG00000000838	Fmr1	20		ENSMUSG00000027067	Ssrp1	20
ENSMUSG00000026193	Fn1	20		ENSMUSG00000004366	Sst	19
ENSMUSG00000025175	Fn3k	3		ENSMUSG00000035431	Sstr1	12

ENSMUSG00000039253	Fn3krp	20		ENSMUSG00000047904	Sstr2	20
ENSMUSG00000075415	Fnbp1	16		ENSMUSG00000044933	Sstr3	1
ENSMUSG00000039735	Fnbp1l	9		ENSMUSG00000037014	Sstr4	4
ENSMUSG00000008200	Fnbp4	20		ENSMUSG00000050824	Sstr5	20
ENSMUSG00000071984	Fndc1	20		ENSMUSG00000029038	Ssu72	3
ENSMUSG00000074738	Fndc10	20		ENSMUSG00000036825	Ssx2ip	5
ENSMUSG00000033487	Fndc3a	20		ENSMUSG00000022403	St13	20
ENSMUSG00000039286	Fndc3b	6		ENSMUSG00000031995	St14	20
ENSMUSG00000038552	Fndc4	9		ENSMUSG00000033740	St18	9
ENSMUSG00000001334	Fndc5	18		ENSMUSG00000013846	St3gal1	20
ENSMUSG00000048721	Fndc9	20		ENSMUSG00000031749	St3gal2	9
ENSMUSG00000035992	Fnip1	7		ENSMUSG00000028538	St3gal3	20
ENSMUSG00000061175	Fnip2	6		ENSMUSG00000032038	St3gal4	18
ENSMUSG00000015994	Fnta	20		ENSMUSG00000056091	St3gal5	1
ENSMUSG00000033373	Fntb	20		ENSMUSG00000022747	St3gal6	20
ENSMUSG00000038368	Focad	7		ENSMUSG00000031024	St5	13
ENSMUSG00000001773	Folh1	1		ENSMUSG00000022885	St6gal1	20
ENSMUSG00000001827	Folr1	13		ENSMUSG00000110170	St6galnac2	20
ENSMUSG00000032725	Folr2	20		ENSMUSG00000057286	St6galnac2	13
ENSMUSG00000022677	Fopnl	20		ENSMUSG00000052544	St6galnac3	9
ENSMUSG00000021250	Fos	20		ENSMUSG00000079442	St6galnac4	20
ENSMUSG00000003545	Fosb	6		ENSMUSG00000039037	St6galnac5	18
ENSMUSG00000029135	Fosl2	20		ENSMUSG00000026811	St6galnac6	20
ENSMUSG00000050295	Foxc1	20		ENSMUSG00000029534	St7	2
ENSMUSG00000046714	Foxc2	20		ENSMUSG00000045576	St7l	2
ENSMUSG00000078302	Foxd1	20		ENSMUSG00000030283	St8sia1	18
ENSMUSG00000042812	Foxf1	20		ENSMUSG00000025789	St8sia2	1
ENSMUSG00000038402	Foxf2	20		ENSMUSG00000056812	St8sia3	9
ENSMUSG00000020950	Foxg1	9		ENSMUSG00000086128	St8sia3os	9
ENSMUSG00000034227	Foxj1	13		ENSMUSG00000040710	St8sia4	12
ENSMUSG00000003154	Foxj2	12		ENSMUSG00000025425	St8sia5	18
ENSMUSG00000032998	Foxj3	20		ENSMUSG00000003418	St8sia6	18
ENSMUSG00000056493	Foxk1	5		ENSMUSG00000042286	Stab1	12
ENSMUSG00000039275	Foxk2	20		ENSMUSG00000035459	Stab2	10
ENSMUSG00000050397	Foxl2	20		ENSMUSG00000032502	Stac	20
ENSMUSG00000097072	Foxl2os	20		ENSMUSG00000017400	Stac2	20
ENSMUSG00000001517	Foxm1	9		ENSMUSG00000040287	Stac3	20
ENSMUSG00000034998	Foxn2	9		ENSMUSG00000037286	Stag1	9
ENSMUSG00000033713	Foxn3	15		ENSMUSG00000025862	Stag2	2
ENSMUSG00000044167	Foxo1	9		ENSMUSG00000036928	Stag3	20
ENSMUSG00000048756	Foxo3	9		ENSMUSG00000026718	Stam	3
ENSMUSG00000042903	Foxo4	20		ENSMUSG00000055371	Stam2	20
ENSMUSG00000052135	Foxo6	20		ENSMUSG00000006906	Stambp	12
ENSMUSG00000030067	Foxp1	9		ENSMUSG00000024776	Stambpl1	9
ENSMUSG00000029563	Foxp2	20		ENSMUSG00000038781	Stap2	20
ENSMUSG00000023991	Foxp4	9		ENSMUSG00000031574	Star	20
ENSMUSG00000038415	Foxq1	20		ENSMUSG00000030688	Stard10	19
ENSMUSG00000071665	Foxr2	20		ENSMUSG00000016128	Stard13	20
ENSMUSG00000039048	Foxred1	20		ENSMUSG00000018167	Stard3	12
ENSMUSG00000016552	Foxred2	6		ENSMUSG00000003062	Stard3nl	18
ENSMUSG00000009566	Fpgs	20		ENSMUSG00000024378	Stard4	18
ENSMUSG00000053870	Fpgt	9		ENSMUSG00000046027	Stard5	20
ENSMUSG00000054237	Fra10ac1	15		ENSMUSG00000079608	Stard6	20
ENSMUSG00000034687	Fras1	2		ENSMUSG00000027367	Stard7	1

ENSMUSG00000067199	Frat1	9		ENSMUSG00000031216	Stard8	9
ENSMUSG00000047604	Frat2	20		ENSMUSG00000033705	Stard9	18
ENSMUSG00000059049	Frem1	13		ENSMUSG00000026104	Stat1	20
ENSMUSG00000037016	Frem2	9		ENSMUSG00000040033	Stat2	20
ENSMUSG00000042353	Frem3	18		ENSMUSG00000004040	Stat3	20
ENSMUSG00000031590	Frg1	20		ENSMUSG00000062939	Stat4	20
ENSMUSG00000087385	Frg2f1	20		ENSMUSG00000004043	Stat5a	9
ENSMUSG00000049122	Frmd3	20		ENSMUSG00000020919	Stat5b	20
ENSMUSG00000026657	Frmd4a	3		ENSMUSG00000002147	Stat6	20
ENSMUSG00000030064	Frmd4b	9		ENSMUSG00000039536	Stau1	1
ENSMUSG00000027238	Frmd5	9		ENSMUSG00000025920	Stau2	19
ENSMUSG00000048285	Frmd6	9		ENSMUSG00000047963	Stbd1	20
ENSMUSG00000036131	Frmd7	20		ENSMUSG00000014813	Stc1	9
ENSMUSG00000024816	Frmd8	20		ENSMUSG00000020303	Stc2	20
ENSMUSG00000035615	Frmpd1	18		ENSMUSG00000015652	Steap1	13
ENSMUSG00000108841	Frmpd2	14		ENSMUSG00000015653	Steap2	20
ENSMUSG00000042425	Frmpd3	17		ENSMUSG00000026389	Steap3	20
ENSMUSG00000049176	Frmpd4	20		ENSMUSG00000012428	Steap4	13
ENSMUSG00000033386	Frrs1	20		ENSMUSG00000028718	Stil	20
ENSMUSG00000045589	Frrs1l	10		ENSMUSG00000030987	Stim1	20
ENSMUSG00000020170	Frs2	20		ENSMUSG00000039156	Stim2	18
ENSMUSG00000023266	Frs3	20		ENSMUSG00000006526	Stimate	20
ENSMUSG00000056602	Fry	2		ENSMUSG00000024966	Stip1	20
ENSMUSG00000070733	Fryl	18		ENSMUSG00000020272	Stk10	20
ENSMUSG00000027004	Frzb	5		ENSMUSG00000003068	Stk11	20
ENSMUSG00000094595	Fsbp	9		ENSMUSG00000026213	Stk11ip	20
ENSMUSG00000029581	Fscn1	20		ENSMUSG00000026201	Stk16	3
ENSMUSG00000011589	Fsd1	20		ENSMUSG00000026094	Stk17b	20
ENSMUSG00000054752	Fsd1l	3		ENSMUSG00000061207	Stk19	20
ENSMUSG00000027344	Fsip1	9		ENSMUSG00000063410	Stk24	18
ENSMUSG00000021765	Fst	1		ENSMUSG00000026277	Stk25	20
ENSMUSG00000022816	Fstl1	20		ENSMUSG00000031112	Stk26	9
ENSMUSG00000020325	Fstl3	20		ENSMUSG00000022329	Stk3	20
ENSMUSG00000036264	Fstl4	6		ENSMUSG00000039954	Stk32a	9
ENSMUSG00000034098	Fstl5	1		ENSMUSG00000029123	Stk32b	17
ENSMUSG00000001155	Ftcd	20		ENSMUSG00000015981	Stk32c	20
ENSMUSG00000091313	Fth-ps2	20		ENSMUSG00000031027	Stk33	20
ENSMUSG00000024661	Fth1	16		ENSMUSG00000037885	Stk35	20
ENSMUSG00000050708	Ftl1	19		ENSMUSG00000033276	Stk36	3
ENSMUSG00000062382	Ftl1-ps1	20		ENSMUSG00000024006	Stk38	3
ENSMUSG00000082062	Ftl2-ps	12		ENSMUSG00000001630	Stk38l	18
ENSMUSG00000055932	Fto	20		ENSMUSG00000027030	Stk39	13
ENSMUSG00000031171	Ftsj1	20		ENSMUSG00000018209	Stk4	20
ENSMUSG00000020706	Ftsj3	20		ENSMUSG00000042608	Stk40	20
ENSMUSG00000086370	Ftx	18		ENSMUSG00000028832	Stmn1	19
ENSMUSG00000028034	Fubp1	20		ENSMUSG00000027500	Stmn2	9
ENSMUSG00000026843	Fubp3	2		ENSMUSG00000027581	Stmn3	7
ENSMUSG00000028673	Fuca1	2		ENSMUSG00000022044	Stmn4	2
ENSMUSG00000019810	Fuca2	9		ENSMUSG00000042694	Stn1	9
ENSMUSG00000025040	Fundc1	12		ENSMUSG00000026880	Stom	20
ENSMUSG00000031198	Fundc2	20		ENSMUSG00000032333	Stoml1	7
ENSMUSG00000025466	Fuom	12		ENSMUSG00000028455	Stoml2	12
ENSMUSG00000030530	Furin	20		ENSMUSG00000027744	Stoml3	20
ENSMUSG00000030795	Fus	12		ENSMUSG00000033855	Ston1	20

ENSMUSG00000046152	Fut10	1		ENSMUSG00000020961	Ston2	20
ENSMUSG00000039357	Fut11	9		ENSMUSG00000036923	Stox1	20
ENSMUSG00000049307	Fut4	20		ENSMUSG00000038143	Stox2	20
ENSMUSG00000021065	Fut8	12		ENSMUSG00000028801	Stpg1	20
ENSMUSG00000055373	Fut9	3		ENSMUSG00000047940	Stpg2	20
ENSMUSG00000011658	Fuz	20		ENSMUSG00000032327	Stra6	20
ENSMUSG00000070583	Fv1	20		ENSMUSG00000028327	Stra6l	20
ENSMUSG00000059363	Fxn	20		ENSMUSG00000069631	Strada	20
ENSMUSG00000027680	Fxr1	20		ENSMUSG00000026027	Stradb	20
ENSMUSG00000018765	Fxr2	20		ENSMUSG00000030224	Strap	20
ENSMUSG00000036570	Fxyd1	3		ENSMUSG00000026915	Strbp	18
ENSMUSG00000059412	Fxyd2	19		ENSMUSG00000033498	Strc	18
ENSMUSG00000057092	Fxyd3	1		ENSMUSG00000014601	Strip1	9
ENSMUSG00000009687	Fxyd5	20		ENSMUSG00000039629	Strip2	18
ENSMUSG00000066705	Fxyd6	17		ENSMUSG00000103476	Strit1	1
ENSMUSG00000036578	Fxyd7	16		ENSMUSG00000024077	Strn	9
ENSMUSG00000022148	Fyb	20		ENSMUSG00000020954	Strn3	7
ENSMUSG00000078612	Fyb2	4		ENSMUSG00000030374	Strn4	9
ENSMUSG00000025241	Fyco1	20		ENSMUSG00000032116	Stt3a	20
ENSMUSG00000019843	Fyn	20		ENSMUSG00000032437	Stt3b	12
ENSMUSG00000022800	Fyttd1	20		ENSMUSG00000039615	Stub1	2
ENSMUSG00000044674	Fzd1	9		ENSMUSG00000053963	Stum	16
ENSMUSG00000081683	Fzd10	18		ENSMUSG00000028879	Stx12	1
ENSMUSG00000072591	Fzd10os	18		ENSMUSG00000027522	Stx16	10
ENSMUSG00000050288	Fzd2	20		ENSMUSG00000061455	Stx17	20
ENSMUSG00000007989	Fzd3	7		ENSMUSG00000029125	Stx18	20
ENSMUSG00000049791	Fzd4	13		ENSMUSG00000007207	Stx1a	16
ENSMUSG00000045005	Fzd5	20		ENSMUSG00000030806	Stx1b	20
ENSMUSG00000041075	Fzd7	13		ENSMUSG00000029428	Stx2	20
ENSMUSG00000036904	Fzd8	18		ENSMUSG00000041488	Stx3	20
ENSMUSG00000049551	Fzd9	9		ENSMUSG00000030805	Stx4a	20
ENSMUSG00000020235	Fzr1	20		ENSMUSG00000010110	Stx5a	5
ENSMUSG00000009633	G0s2	20		ENSMUSG00000026470	Stx6	18
ENSMUSG00000035293	G2e3	20		ENSMUSG00000019998	Stx7	1
ENSMUSG00000018583	G3bp1	20		ENSMUSG00000020903	Stx8	10
ENSMUSG00000029405	G3bp2	3		ENSMUSG00000026797	Stxbp1	9
ENSMUSG00000086308	G630016G05Rik	18		ENSMUSG00000004626	Stxbp2	20
ENSMUSG00000085620	G630018N14Rik	1		ENSMUSG00000027882	Stxbp3	20
ENSMUSG00000106896	G630022F23Rik	20		ENSMUSG00000071640	Stxbp3-ps	20
ENSMUSG00000097149	G630030J09Rik	20		ENSMUSG00000020546	Stxbp4	20
ENSMUSG00000114959	G630093K05Rik	1		ENSMUSG00000019790	Stxbp5	1
ENSMUSG00000034793	G6pc3	9		ENSMUSG00000022829	Stxbp5l	2
ENSMUSG00000031400	G6pdx	4		ENSMUSG00000046314	Stxbp6	19
ENSMUSG00000097573	G730003C15Rik	20		ENSMUSG00000032899	Styk1	20
ENSMUSG00000025579	Gaa	10		ENSMUSG00000053205	Styx	20
ENSMUSG00000031714	Gab1	16		ENSMUSG00000019178	Styxl1	20
ENSMUSG00000004508	Gab2	1		ENSMUSG00000022205	Sub1	18
ENSMUSG00000032750	Gab3	9		ENSMUSG00000022110	Sucla2	9
ENSMUSG00000018567	Gabarap	12		ENSMUSG00000052738	Suclg1	10
ENSMUSG00000030161	Gabarapl1	10		ENSMUSG00000061838	Suclg2	20
ENSMUSG00000031950	Gabarapl2	12		ENSMUSG00000040297	Suco	20
ENSMUSG00000024462	Gabbr1	20		ENSMUSG00000066900	Suds3	20
ENSMUSG00000039809	Gabbr2	19		ENSMUSG00000025231	Sufu	20
ENSMUSG00000008976	Gabpa	20		ENSMUSG00000055137	Sugct	1

ENSMUSG00000027361	Gabpb1	20		ENSMUSG00000011306	Sugp1	20
ENSMUSG00000038766	Gabpb2	20		ENSMUSG00000036054	Sugp2	1
ENSMUSG00000010803	Gabra1	3		ENSMUSG00000022024	Sugt1	20
ENSMUSG00000000560	Gabra2	18		ENSMUSG00000016918	Sulf1	13
ENSMUSG00000031343	Gabra3	17		ENSMUSG00000006800	Sulf2	9
ENSMUSG00000029211	Gabra4	8		ENSMUSG00000030711	Sult1a1	20
ENSMUSG00000055078	Gabra5	5		ENSMUSG00000003271	Sult2b1	20
ENSMUSG00000029212	Gabrb1	19		ENSMUSG00000018865	Sult4a1	9
ENSMUSG00000007653	Gabrb2	3		ENSMUSG00000030101	Sumf1	20
ENSMUSG00000033676	Gabrb3	6		ENSMUSG00000025538	Sumf2	1
ENSMUSG00000029054	Gabrd	1		ENSMUSG00000026021	Sumo1	20
ENSMUSG00000031340	Gabre	17		ENSMUSG00000020738	Sumo2	20
ENSMUSG00000001260	Gabrg1	20		ENSMUSG00000020265	Sumo3	19
ENSMUSG00000020436	Gabrg2	20		ENSMUSG00000036817	Sun1	20
ENSMUSG00000055026	Gabrg3	9		ENSMUSG00000042524	Sun2	9
ENSMUSG00000031344	Gabrq	17		ENSMUSG00000049858	Suox	20
ENSMUSG00000023267	Gabrr2	16		ENSMUSG00000035726	Supt16	20
ENSMUSG00000070880	Gad1	19		ENSMUSG00000027751	Supt20	6
ENSMUSG00000090665	Gad1-ps	20		ENSMUSG00000038954	Supt3	9
ENSMUSG00000087264	Gad1os	20		ENSMUSG00000020485	Supt4a	2
ENSMUSG00000026787	Gad2	19		ENSMUSG00000110960	Supt4b	20
ENSMUSG00000036390	Gadd45a	20		ENSMUSG00000003435	Supt5	20
ENSMUSG00000015312	Gadd45b	20		ENSMUSG00000002052	Supt6	9
ENSMUSG00000021453	Gadd45g	20		ENSMUSG00000053134	Supt7l	10
ENSMUSG00000033751	Gadd45gip1	12		ENSMUSG00000020079	Supv3l1	20
ENSMUSG00000056880	Gadl1	12		ENSMUSG00000015790	Surf1	20
ENSMUSG00000062234	Gak	20		ENSMUSG00000014873	Surf2	20
ENSMUSG00000024907	Gal	20		ENSMUSG00000014867	Surf4	20
ENSMUSG00000049721	Gal3st1	18		ENSMUSG00000036160	Surf6	20
ENSMUSG00000047658	Gal3st3	9		ENSMUSG00000038578	Susd1	6
ENSMUSG00000021003	Galc	19		ENSMUSG00000006342	Susd2	20
ENSMUSG00000028671	Gale	9		ENSMUSG00000021384	Susd3	12
ENSMUSG00000020766	Galk1	20		ENSMUSG00000038576	Susd4	20
ENSMUSG00000027207	Galk2	20		ENSMUSG00000086596	Susd5	9
ENSMUSG00000035473	Galm	13		ENSMUSG00000021133	Susd6	5
ENSMUSG00000015027	Galns	20		ENSMUSG00000039231	Suv39h1	20
ENSMUSG00000000420	Galnt1	18		ENSMUSG00000026646	Suv39h2	20
ENSMUSG00000020520	Galnt10	9		ENSMUSG00000017548	Suz12	20
ENSMUSG00000038072	Galnt11	20		ENSMUSG00000038486	Sv2a	9
ENSMUSG00000039774	Galnt12	20		ENSMUSG00000053025	Sv2b	1
ENSMUSG00000060988	Galnt13	7		ENSMUSG00000051111	Sv2c	20
ENSMUSG00000024064	Galnt14	20		ENSMUSG00000028643	Svbp	20
ENSMUSG00000021903	Galnt15	20		ENSMUSG00000028369	Svep1	17
ENSMUSG00000021130	Galnt16	19		ENSMUSG00000109648	Svet1	20
ENSMUSG00000034040	Galnt17	19		ENSMUSG00000024236	Svil	9
ENSMUSG00000038296	Galnt18	9		ENSMUSG00000074093	Svip	18
ENSMUSG00000089704	Galnt2	20		ENSMUSG00000042078	Svop	20
ENSMUSG00000026994	Galnt3	20		ENSMUSG00000031015	Swap70	19
ENSMUSG00000090035	Galnt4	20		ENSMUSG00000044627	Swi5	12
ENSMUSG00000037280	Galnt6	15		ENSMUSG00000051238	Swsap1	20
ENSMUSG00000031608	Galnt7	20		ENSMUSG00000052748	Swt1	20
ENSMUSG00000033316	Galnt9	19		ENSMUSG00000031357	Syap1	20
ENSMUSG00000096914	Galntl6	7		ENSMUSG00000022340	Sybu	18
ENSMUSG00000024553	Galr1	20		ENSMUSG00000025480	Syce1	20

ENSMUSG00000036073	Galt	5		ENSMUSG00000003824	Syce2	20
ENSMUSG00000020150	Gamt	9		ENSMUSG00000060445	Sycp2	1
ENSMUSG00000052557	Gan	3		ENSMUSG00000038651	Sycp2l	20
ENSMUSG00000071650	Ganab	20		ENSMUSG00000020059	Sycp3	20
ENSMUSG00000062646	Ganc	20		ENSMUSG00000032714	Syde1	20
ENSMUSG00000047261	Gap43	9		ENSMUSG00000036863	Syde2	1
ENSMUSG00000057666	Gapdh	12		ENSMUSG00000028821	Syf2	3
ENSMUSG00000098149	Gapdh-ps14	20		ENSMUSG00000021457	Syk	12
ENSMUSG00000096438	Gapdh-ps15	12		ENSMUSG00000023118	Sympk	20
ENSMUSG00000061099	Gapdhs	20		ENSMUSG00000037217	Syn1	1
ENSMUSG00000026867	Gapvd1	12		ENSMUSG00000009394	Syn2	5
ENSMUSG00000028010	Gar1	20		ENSMUSG00000059602	Syn3	6
ENSMUSG00000042680	Garem1	9		ENSMUSG00000001333	Sync	20
ENSMUSG00000044576	Garem2	3		ENSMUSG00000032423	Syncrip	2
ENSMUSG00000038860	Garnl3	20		ENSMUSG00000074736	Syndig1	20
ENSMUSG00000029777	Gars	9		ENSMUSG00000071234	Syndig1l	9
ENSMUSG00000022962	Gart	20		ENSMUSG00000096054	Syne1	7
ENSMUSG00000052957	Gas1	20		ENSMUSG00000063450	Syne2	13
ENSMUSG00000030498	Gas2	20		ENSMUSG00000054150	Syne3	20
ENSMUSG00000034201	Gas2l1	20		ENSMUSG00000019737	Syne4	20
ENSMUSG00000020686	Gas2l2	13		ENSMUSG00000067629	Syngap1	9
ENSMUSG00000074802	Gas2l3	20		ENSMUSG00000022415	Syngr1	18
ENSMUSG00000053332	Gas5	12		ENSMUSG00000048277	Syngr2	20
ENSMUSG00000031451	Gas6	13		ENSMUSG00000007021	Syngr3	20
ENSMUSG00000033066	Gas7	9		ENSMUSG00000022973	Synj1	3
ENSMUSG00000040220	Gas8	20		ENSMUSG00000023805	Synj2	18
ENSMUSG00000038233	Gask1a	20		ENSMUSG00000090935	Synj2bp	12
ENSMUSG00000027955	Gask1b	1		ENSMUSG00000030554	Synm	18
ENSMUSG00000015053	Gata2	1		ENSMUSG00000043079	Synpo	9
ENSMUSG00000007415	Gatad1	20		ENSMUSG00000050315	Synpo2	18
ENSMUSG00000036180	Gatad2a	18		ENSMUSG00000056296	Synpr	9
ENSMUSG00000042390	Gatad2b	7		ENSMUSG00000034940	Synrg	3
ENSMUSG00000028085	Gatb	9		ENSMUSG00000031144	Syp	9
ENSMUSG00000029536	Gatc	20		ENSMUSG00000020570	Sypl	20
ENSMUSG00000051007	Gatd1	20		ENSMUSG00000027887	Sypl2	5
ENSMUSG00000053329	Gatd3a	20		ENSMUSG00000045503	Sys1	20
ENSMUSG00000027199	Gatm	20		ENSMUSG00000035864	Syt1	19
ENSMUSG00000028048	Gba	20		ENSMUSG00000063260	Syt10	9
ENSMUSG00000028467	Gba2	9		ENSMUSG00000068923	Syt11	20
ENSMUSG00000022707	Gbe1	18		ENSMUSG00000049303	Syt12	9
ENSMUSG00000025224	Gbf1	3		ENSMUSG00000027220	Syt13	19
ENSMUSG00000026829	Gbgt1	20		ENSMUSG00000016200	Syt14	9
ENSMUSG00000092021	Gbp11	20		ENSMUSG00000041479	Syt15	20
ENSMUSG00000028270	Gbp2	20		ENSMUSG00000044912	Syt16	18
ENSMUSG00000028268	Gbp3	20		ENSMUSG00000058420	Syt17	5
ENSMUSG00000079363	Gbp4	20		ENSMUSG00000026452	Syt2	18
ENSMUSG00000105504	Gbp5	1		ENSMUSG00000030731	Syt3	20
ENSMUSG00000104713	Gbp6	20		ENSMUSG00000024261	Syt4	2
ENSMUSG00000040253	Gbp7	20		ENSMUSG00000004961	Syt5	18
ENSMUSG00000029298	Gbp9	9		ENSMUSG00000027849	Syt6	6
ENSMUSG00000067724	Gbx1	1		ENSMUSG00000024743	Syt7	19
ENSMUSG00000034486	Gbx2	18		ENSMUSG00000062542	Syt9	18
ENSMUSG00000026893	Gca	1		ENSMUSG00000030616	Sytl2	20
ENSMUSG00000006378	Gcat	20		ENSMUSG00000031255	Sytl4	20

ENSMUSG00000029708	Gcc1	20		ENSMUSG00000054453	Sytl5	9
ENSMUSG00000038039	Gcc2	20		ENSMUSG00000024807	Syvn1	18
ENSMUSG00000003809	Gcdh	20		ENSMUSG00000040842	Szrd1	20
ENSMUSG00000035125	Gcfc2	20		ENSMUSG00000033253	Szt2	20
ENSMUSG00000037580	Gch1	20		ENSMUSG00000058159	T2	20
ENSMUSG00000041798	Gck	10		ENSMUSG00000022414	Tab1	20
ENSMUSG00000059434	Gckr	6		ENSMUSG00000015755	Tab2	20
ENSMUSG00000032350	Gclc	20		ENSMUSG00000035476	Tab3	20
ENSMUSG00000028124	Gclm	20		ENSMUSG00000061762	Tac1	9
ENSMUSG00000041638	Gcn1	20		ENSMUSG00000025400	Tac2	17
ENSMUSG00000038843	Gcnt1	20		ENSMUSG00000065954	Tacc1	9
ENSMUSG00000021360	Gcnt2	9		ENSMUSG00000030852	Tacc2	20
ENSMUSG00000091387	Gcnt4	20		ENSMUSG00000037313	Tacc3	20
ENSMUSG00000034424	Gcsh	20		ENSMUSG00000001983	Taco1	20
ENSMUSG00000058624	Gda	18		ENSMUSG00000085255	Taco1os	20
ENSMUSG00000025777	Gdap1	3		ENSMUSG00000030043	Tacr1	20
ENSMUSG00000017943	Gdap1l1	9		ENSMUSG00000028172	Tacr3	20
ENSMUSG00000027865	Gdap2	12		ENSMUSG00000026563	Tada1	20
ENSMUSG00000033917	Gde1	20		ENSMUSG00000018651	Tada2a	20
ENSMUSG00000021943	Gdf10	20		ENSMUSG00000029196	Tada2b	10
ENSMUSG00000025352	Gdf11	19		ENSMUSG00000048930	Tada3	20
ENSMUSG00000037660	Gdf7	9		ENSMUSG00000031314	Taf1	3
ENSMUSG00000018238	Gdf9	20		ENSMUSG00000043866	Taf10	3
ENSMUSG00000015291	Gdi1	1		ENSMUSG00000024218	Taf11	20
ENSMUSG00000021218	Gdi2	20		ENSMUSG00000028899	Taf12	20
ENSMUSG00000022144	Gdnf	9		ENSMUSG00000048100	Taf13	9
ENSMUSG00000061666	Gdpd1	20		ENSMUSG00000020680	Taf15	1
ENSMUSG00000019359	Gdpd2	20		ENSMUSG00000072258	Taf1a	20
ENSMUSG00000030703	Gdpd3	20		ENSMUSG00000059669	Taf1b	18
ENSMUSG00000035314	Gdpd5	5		ENSMUSG00000031832	Taf1c	19
ENSMUSG00000050973	Gdpgp1	9		ENSMUSG00000031939	Taf1d	20
ENSMUSG00000028214	Gem	20		ENSMUSG00000037343	Taf2	20
ENSMUSG00000060121	Gemin2	20		ENSMUSG00000025782	Taf3	20
ENSMUSG00000049396	Gemin4	20		ENSMUSG00000039117	Taf4	20
ENSMUSG00000037275	Gemin5	20		ENSMUSG00000054321	Taf4b	1
ENSMUSG00000055760	Gemin6	1		ENSMUSG00000025049	Taf5	20
ENSMUSG00000044709	Gemin7	7		ENSMUSG00000038697	Taf5l	9
ENSMUSG00000040621	Gemin8	20		ENSMUSG00000036980	Taf6	20
ENSMUSG00000051235	Gen1	20		ENSMUSG00000003680	Taf6l	20
ENSMUSG00000025858	Get4	20		ENSMUSG00000051316	Taf7	19
ENSMUSG00000020932	Gfap	16		ENSMUSG00000023980	Taf8	20
ENSMUSG00000040888	Gfer	20		ENSMUSG00000052293	Taf9	20
ENSMUSG00000027774	Gfm1	3		ENSMUSG00000047242	Taf9b	7
ENSMUSG00000021666	Gfm2	20		ENSMUSG00000059187	Tafa1	5
ENSMUSG00000051335	Gfod1	7		ENSMUSG00000044071	Tafa2	19
ENSMUSG00000013150	Gfod2	9		ENSMUSG00000046500	Tafa4	18
ENSMUSG00000029992	Gfpt1	3		ENSMUSG00000054863	Tafa5	20
ENSMUSG00000020363	Gfpt2	9		ENSMUSG00000033450	Tagap	20
ENSMUSG00000025089	Gfra1	20		ENSMUSG00000052031	Tagap1	9
ENSMUSG00000022103	Gfra2	18		ENSMUSG00000032085	Tagln	20
ENSMUSG00000027316	Gfra4	20		ENSMUSG00000026547	Tagln2	20
ENSMUSG00000033128	Gga1	20		ENSMUSG00000022658	Tagln3	7
ENSMUSG00000030872	Gga2	9		ENSMUSG00000028717	Tal1	20
ENSMUSG00000020740	Gga3	2		ENSMUSG00000028417	Tal2	20

ENSMUSG00000041625	Ggact	9		ENSMUSG00000025503	Taldo1	3
ENSMUSG00000002797	Ggct	20		ENSMUSG00000030316	Tamm41	20
ENSMUSG00000073987	Ggh	20		ENSMUSG00000035168	Tanc1	19
ENSMUSG00000031493	Ggn	20		ENSMUSG00000053580	Tanc2	7
ENSMUSG00000048731	Ggnbp1	14		ENSMUSG00000041949	Tango6	20
ENSMUSG00000020530	Ggnbp2	20		ENSMUSG00000064289	Tank	9
ENSMUSG00000021302	Ggps1	20		ENSMUSG00000017291	Taok1	12
ENSMUSG00000006345	Ggt1	20		ENSMUSG00000059981	Taok2	20
ENSMUSG00000006344	Ggt5	20		ENSMUSG00000061288	Taok3	8
ENSMUSG00000027603	Ggt7	9		ENSMUSG00000037321	Tap1	20
ENSMUSG00000035778	Ggta1	20		ENSMUSG00000024339	Tap2	20
ENSMUSG00000017747	Ghdc	20		ENSMUSG00000024308	Tapbp	20
ENSMUSG00000041028	Ghitm	9		ENSMUSG00000038213	Tapbpl	20
ENSMUSG00000055737	Ghr	20		ENSMUSG00000046985	Tapt1	20
ENSMUSG00000018415	Gid4	20		ENSMUSG00000090290	Tarbp1	12
ENSMUSG00000027573	Gid8	20		ENSMUSG00000023051	Tarbp2	12
ENSMUSG00000029714	Gigyf1	1		ENSMUSG00000041459	Tardbp	20
ENSMUSG00000048000	Gigyf2	20		ENSMUSG00000022241	Tars	20
ENSMUSG00000090019	Gimap1	20		ENSMUSG00000028107	Tars2	12
ENSMUSG00000043505	Gimap5	20		ENSMUSG00000030515	Tarsl2	9
ENSMUSG00000047867	Gimap6	20		ENSMUSG00000029072	Tas1r3	20
ENSMUSG00000064262	Gimap8	1		ENSMUSG00000040651	Tasor	2
ENSMUSG00000051124	Gimap9	20		ENSMUSG00000033799	Tasor2	2
ENSMUSG00000026333	Gin1	20		ENSMUSG00000039033	Tasp1	20
ENSMUSG00000040006	Ginm1	20		ENSMUSG00000050891	Tatdn1	10
ENSMUSG00000027454	Gins1	20		ENSMUSG00000056952	Tatdn2	13
ENSMUSG00000031821	Gins2	20		ENSMUSG00000026632	Tatdn3	18
ENSMUSG00000031669	Gins3	20		ENSMUSG00000004535	Tax1bp1	20
ENSMUSG00000031546	Gins4	20		ENSMUSG00000040158	Tax1bp3	20
ENSMUSG00000019433	Gipc1	20		ENSMUSG00000009995	Taz	20
ENSMUSG00000039131	Gipc2	1		ENSMUSG00000029174	Tbc1d1	19
ENSMUSG00000034872	Gipc3	20		ENSMUSG00000034412	Tbc1d10a	12
ENSMUSG00000030406	Gipr	16		ENSMUSG00000042492	Tbc1d10b	9
ENSMUSG00000011877	Git1	1		ENSMUSG00000048720	Tbc1d12	20
ENSMUSG00000041890	Git2	18		ENSMUSG00000039678	Tbc1d13	20
ENSMUSG00000050953	Gja1	1		ENSMUSG00000029192	Tbc1d14	9
ENSMUSG00000050234	Gja4	20		ENSMUSG00000020130	Tbc1d15	20
ENSMUSG00000057123	Gja5	20		ENSMUSG00000039976	Tbc1d16	9
ENSMUSG00000055691	Gja6	20		ENSMUSG00000038520	Tbc1d17	20
ENSMUSG00000047797	Gjb1	20		ENSMUSG00000039178	Tbc1d19	13
ENSMUSG00000046352	Gjb2	20		ENSMUSG00000039813	Tbc1d2	13
ENSMUSG00000040055	Gjb6	18		ENSMUSG00000027465	Tbc1d20	9
ENSMUSG00000034520	Gjc1	18		ENSMUSG00000051864	Tbc1d22a	20
ENSMUSG00000043448	Gjc2	16		ENSMUSG00000042203	Tbc1d22b	9
ENSMUSG00000056966	Gjc3	16		ENSMUSG00000089900	Tbc1d22bos	20
ENSMUSG00000068615	Gjd2	20		ENSMUSG00000022749	Tbc1d23	20
ENSMUSG00000047197	Gjd3	20		ENSMUSG00000036473	Tbc1d24	19
ENSMUSG00000025059	Gk	20		ENSMUSG00000039201	Tbc1d25	20
ENSMUSG00000041440	Gk5	10		ENSMUSG00000037410	Tbc1d2b	20
ENSMUSG00000021552	Gkap1	7		ENSMUSG00000052302	Tbc1d30	8
ENSMUSG00000030048	Gkn3	20		ENSMUSG00000022364	Tbc1d31	20
ENSMUSG00000031266	Gla	20		ENSMUSG00000038122	Tbc1d32	20
ENSMUSG00000045594	Glb1	9		ENSMUSG00000033083	Tbc1d4	1
ENSMUSG00000026200	Glb1l	20		ENSMUSG00000023923	Tbc1d5	20

ENSMUSG00000036395	Glb1l2	13		ENSMUSG00000021368	Tbc1d7	18
ENSMUSG00000029638	Glcci1	6		ENSMUSG00000003134	Tbc1d8	9
ENSMUSG00000032252	Glce	9		ENSMUSG00000042473	Tbc1d8b	2
ENSMUSG00000024827	Gldc	9		ENSMUSG00000031709	Tbc1d9	13
ENSMUSG00000046167	Gldn	20		ENSMUSG00000036644	Tbc1d9b	20
ENSMUSG00000019715	Gle1	20		ENSMUSG00000042043	Tbca	12
ENSMUSG00000003316	Glg1	3		ENSMUSG00000006095	Tbcb	20
ENSMUSG00000025407	Gli1	6		ENSMUSG00000036430	Tbcc	20
ENSMUSG00000048402	Gli2	20		ENSMUSG00000004462	Tbccd1	1
ENSMUSG00000021318	Gli3	9		ENSMUSG00000039230	Tbcd	20
ENSMUSG00000056888	Glipr1	9		ENSMUSG00000039233	Tbce	12
ENSMUSG00000028480	Glipr2	20		ENSMUSG00000037287	Tbcel	11
ENSMUSG00000034762	Glis1	20		ENSMUSG00000028030	Tbck	7
ENSMUSG00000014303	Glis2	20		ENSMUSG00000020115	Tbk1	20
ENSMUSG00000052942	Glis3	20		ENSMUSG00000038517	Tbkbp1	9
ENSMUSG00000029276	Glmn	20		ENSMUSG00000025246	Tbl1x	5
ENSMUSG00000001418	Glmp	20		ENSMUSG00000027630	Tbl1xr1	20
ENSMUSG00000082100	Glns-ps1	1		ENSMUSG00000005374	Tbl2	20
ENSMUSG00000024026	Glo1	18		ENSMUSG00000040688	Tbl3	20
ENSMUSG00000017286	Glod4	20		ENSMUSG00000014767	Tbp	20
ENSMUSG00000024027	Glp1r	20		ENSMUSG00000071359	Tbpl1	20
ENSMUSG00000049928	Glp2r	20		ENSMUSG00000035033	Tbr1	5
ENSMUSG00000000263	Glra1	18		ENSMUSG00000011114	Tbrg1	20
ENSMUSG00000018589	Glra2	20		ENSMUSG00000000384	Tbrg4	20
ENSMUSG00000038257	Glra3	17		ENSMUSG00000009097	Tbx1	20
ENSMUSG00000028020	Glrb	18		ENSMUSG00000032419	Tbx18	20
ENSMUSG00000062310	Glrp1	20		ENSMUSG00000000093	Tbx2	20
ENSMUSG00000021591	Glrx	9		ENSMUSG00000018604	Tbx3	20
ENSMUSG00000018196	Glrx2	20		ENSMUSG00000087516	Tbx3os1	20
ENSMUSG00000031068	Glrx3	20		ENSMUSG00000030699	Tbx6	20
ENSMUSG00000021102	Glrx5	20		ENSMUSG00000029925	Tbxas1	20
ENSMUSG00000026103	Gls	3		ENSMUSG00000021187	Tc2n	13
ENSMUSG00000044005	Gls2	18		ENSMUSG00000036667	Tcaf1	20
ENSMUSG00000049971	Glt1d1	20		ENSMUSG00000029851	Tcaf2	20
ENSMUSG00000021916	Glt8d1	20		ENSMUSG00000046603	Tcaim	20
ENSMUSG00000020251	Glt8d2	5		ENSMUSG00000033813	Tcea1	20
ENSMUSG00000011884	Gltp	16		ENSMUSG00000067869	Tcea1-ps1	20
ENSMUSG00000108799	Glud-ps	20		ENSMUSG00000059540	Tcea2	20
ENSMUSG00000021794	Glud1	18		ENSMUSG00000001604	Tcea3	13
ENSMUSG00000026473	Glul	1		ENSMUSG00000049536	Tceal1	17
ENSMUSG00000020258	Glyctk	9		ENSMUSG00000044550	Tceal3	12
ENSMUSG00000022536	Glyr1	20		ENSMUSG00000054034	Tceal5	20
ENSMUSG00000056771	Gm10010	20		ENSMUSG00000031409	Tceal6	19
ENSMUSG00000057580	Gm10012	20		ENSMUSG00000079428	Tceal7	20
ENSMUSG00000056943	Gm10015	20		ENSMUSG00000051579	Tceal8	20
ENSMUSG00000094012	Gm10024	20		ENSMUSG00000042712	Tceal9	8
ENSMUSG00000057802	Gm10030	20		ENSMUSG00000051224	Tceanc	20
ENSMUSG00000110444	Gm10033	20		ENSMUSG00000028619	Tceanc2	20
ENSMUSG00000058064	Gm10036	12		ENSMUSG00000024498	Tcerg1	3
ENSMUSG00000058246	Gm10037	20		ENSMUSG00000091002	Tcerg1l	20
ENSMUSG00000091478	Gm10039	20		ENSMUSG00000032228	Tcf12	20
ENSMUSG00000109793	Gm10043	20		ENSMUSG00000068079	Tcf15	20
ENSMUSG00000072723	Gm10044	9		ENSMUSG00000050410	Tcf19	20
ENSMUSG00000103544	Gm10048	19		ENSMUSG00000041852	Tcf20	8

ENSMUSG00000058905	Gm10051	12		ENSMUSG00000001472	Tcf25	18
ENSMUSG00000058922	Gm10052	20		ENSMUSG00000020167	Tcf3	19
ENSMUSG00000058927	Gm10053	10		ENSMUSG00000053477	Tcf4	5
ENSMUSG00000113368	Gm10054	20		ENSMUSG00000000782	Tcf7	9
ENSMUSG00000059064	Gm10059	20		ENSMUSG00000055799	Tcf7l1	6
ENSMUSG00000110669	Gm10060	20		ENSMUSG00000024985	Tcf7l2	18
ENSMUSG00000059244	Gm10062	20		ENSMUSG00000038932	Tcfl5	20
ENSMUSG00000059659	Gm10069	20		ENSMUSG00000052415	Tchh	1
ENSMUSG00000060019	Gm10073	3		ENSMUSG00000002486	Tchp	20
ENSMUSG00000060143	Gm10076	12		ENSMUSG00000056313	Tcim	20
ENSMUSG00000060467	Gm10080	10		ENSMUSG00000001750	Tcirg1	20
ENSMUSG00000060730	Gm10086	20		ENSMUSG00000020432	Tcn2	19
ENSMUSG00000061062	Gm10093	9		ENSMUSG00000024613	Tcof1	1
ENSMUSG00000113555	Gm10095	12		ENSMUSG00000068039	Tcp1	20
ENSMUSG00000062038	Gm10108	20		ENSMUSG00000062859	Tcp11	14
ENSMUSG00000062093	Gm10110	20		ENSMUSG00000027175	Tcp11l1	18
ENSMUSG00000062319	Gm10115	9		ENSMUSG00000020034	Tcp11l2	9
ENSMUSG00000062561	Gm10118	9		ENSMUSG00000039461	Tcta	20
ENSMUSG00000112909	Gm10120	3		ENSMUSG00000023949	Tcte1	20
ENSMUSG00000062933	Gm10123	12		ENSMUSG00000038347	Tcte2	9
ENSMUSG00000063087	Gm10125	20		ENSMUSG00000014075	Tctex1d2	20
ENSMUSG00000095315	Gm10130	20		ENSMUSG00000038593	Tctn1	9
ENSMUSG00000063412	Gm10131	2		ENSMUSG00000029386	Tctn2	13
ENSMUSG00000063556	Gm10132	20		ENSMUSG00000025008	Tctn3	20
ENSMUSG00000063611	Gm10134	20		ENSMUSG00000034674	Tdg	9
ENSMUSG00000063754	Gm10136	12		ENSMUSG00000032494	Tdgf1	20
ENSMUSG00000100954	Gm10138	20		ENSMUSG00000021177	Tdp1	20
ENSMUSG00000064208	Gm10145	20		ENSMUSG00000035958	Tdp2	20
ENSMUSG00000064317	Gm10146	12		ENSMUSG00000025081	Tdrd1	12
ENSMUSG00000066116	Gm10154	20		ENSMUSG00000030491	Tdrd12	20
ENSMUSG00000066180	Gm10155	20		ENSMUSG00000022019	Tdrd3	20
ENSMUSG00000066245	Gm10156	20		ENSMUSG00000060985	Tdrd5	9
ENSMUSG00000066270	Gm10157	20		ENSMUSG00000040140	Tdrd6	20
ENSMUSG00000066554	Gm10167	20		ENSMUSG00000035517	Tdrd7	20
ENSMUSG00000083669	Gm10169	20		ENSMUSG00000054003	Tdrd9	20
ENSMUSG00000089803	Gm10171	3		ENSMUSG00000041912	Tdrkh	20
ENSMUSG00000066724	Gm10175	12		ENSMUSG00000050052	Tdrp	9
ENSMUSG00000066752	Gm10176	20		ENSMUSG00000055320	Tead1	20
ENSMUSG00000090136	Gm10177	10		ENSMUSG00000030796	Tead2	20
ENSMUSG00000066807	Gm10179	11		ENSMUSG00000002249	Tead3	20
ENSMUSG00000066809	Gm10180	18		ENSMUSG00000030353	Tead4	20
ENSMUSG00000066878	Gm10184	20		ENSMUSG00000029217	Tec	20
ENSMUSG00000067719	Gm10221	7		ENSMUSG00000066621	Tecpr1	20
ENSMUSG00000067736	Gm10222	20		ENSMUSG00000021275	Tecpr2	20
ENSMUSG00000080932	Gm10224	20		ENSMUSG00000031708	Tecr	2
ENSMUSG00000067929	Gm10226	20		ENSMUSG00000037705	Tecta	20
ENSMUSG00000068120	Gm10231	19		ENSMUSG00000037466	Tedc1	18
ENSMUSG00000068141	Gm10232	19		ENSMUSG00000024118	Tedc2	20
ENSMUSG00000068397	Gm10240	20		ENSMUSG00000045968	Teddm2	1
ENSMUSG00000084838	Gm10241	20		ENSMUSG00000022389	Tef	1
ENSMUSG00000095526	Gm10243	12		ENSMUSG00000046909	Tefm	20
ENSMUSG00000068674	Gm10247	20		ENSMUSG00000006386	Tek	20
ENSMUSG00000068706	Gm10250	12		ENSMUSG00000020799	Tekt1	13
ENSMUSG00000104333	Gm10253	20		ENSMUSG00000028845	Tekt2	1

ENSMUSG00000069011	Gm10254	20		ENSMUSG00000024175	Tekt4	20
ENSMUSG00000114759	Gm10257	20		ENSMUSG00000039179	Tekt5	20
ENSMUSG00000069117	Gm10260	11		ENSMUSG00000024170	Telo2	9
ENSMUSG00000066407	Gm10263	11		ENSMUSG00000020778	Ten1	12
ENSMUSG00000066475	Gm10268	18		ENSMUSG00000016150	Tenm1	9
ENSMUSG00000091449	Gm10269	12		ENSMUSG00000049336	Tenm2	20
ENSMUSG00000069622	Gm10273	20		ENSMUSG00000031561	Tenm3	12
ENSMUSG00000069682	Gm10275	12		ENSMUSG00000048078	Tenm4	20
ENSMUSG00000070713	Gm10282	20		ENSMUSG00000042167	Tent2	9
ENSMUSG00000070025	Gm10284	9		ENSMUSG00000034575	Tent4a	20
ENSMUSG00000070342	Gm10287	6		ENSMUSG00000036779	Tent4b	1
ENSMUSG00000070343	Gm10288	12		ENSMUSG00000032265	Tent5a	20
ENSMUSG00000105775	Gm10290	20		ENSMUSG00000044468	Tent5c	12
ENSMUSG00000070443	Gm10291	20		ENSMUSG00000006281	Tep1	20
ENSMUSG00000102846	Gm10292	20		ENSMUSG00000025377	Tepsin	20
ENSMUSG00000070490	Gm10293	20		ENSMUSG00000052616	Terb1	20
ENSMUSG00000109222	Gm10297	9		ENSMUSG00000025925	Terf1	19
ENSMUSG00000104479	Gm10305	20		ENSMUSG00000031921	Terf2	20
ENSMUSG00000071102	Gm10313	20		ENSMUSG00000033430	Terf2ip	20
ENSMUSG00000071195	Gm10318	20		ENSMUSG00000021611	Tert	20
ENSMUSG00000071343	Gm10327	20		ENSMUSG00000029552	Tes	20
ENSMUSG00000071532	Gm10335	12		ENSMUSG00000113255	Tes3-ps	9
ENSMUSG00000071586	Gm10337	20		ENSMUSG00000029359	Tesc	9
ENSMUSG00000071671	Gm10343	20		ENSMUSG00000028458	Tesk1	20
ENSMUSG00000114220	Gm10353	10		ENSMUSG00000033985	Tesk2	20
ENSMUSG00000072387	Gm10356	20		ENSMUSG00000047146	Tet1	20
ENSMUSG00000094708	Gm10359	20		ENSMUSG00000040943	Tet2	2
ENSMUSG00000116174	Gm10362	20		ENSMUSG00000034832	Tet3	7
ENSMUSG00000115767	Gm10366	20		ENSMUSG00000028345	Tex10	20
ENSMUSG00000107456	Gm10400	20		ENSMUSG00000009670	Tex11	16
ENSMUSG00000114266	Gm10403	20		ENSMUSG00000010342	Tex14	19
ENSMUSG00000105044	Gm10416	20		ENSMUSG00000009628	Tex15	19
ENSMUSG00000072769	Gm10419	9		ENSMUSG00000040548	Tex2	20
ENSMUSG00000072789	Gm10420	20		ENSMUSG00000029660	Tex26	20
ENSMUSG00000099907	Gm10421	9		ENSMUSG00000014748	Tex261	20
ENSMUSG00000117286	Gm1043	1		ENSMUSG00000040813	Tex264	3
ENSMUSG00000113679	Gm10432	20		ENSMUSG00000026049	Tex30	1
ENSMUSG00000072902	Gm10435	20		ENSMUSG00000026592	Tex35	20
ENSMUSG00000072940	Gm10443	10		ENSMUSG00000079304	Tex52	20
ENSMUSG00000073000	Gm10451	1		ENSMUSG00000090626	Tex9	13
ENSMUSG00000113707	Gm10457	20		ENSMUSG00000003923	Tfam	20
ENSMUSG00000107277	Gm10461	20		ENSMUSG00000005718	Tfap4	20
ENSMUSG00000073179	Gm10478	20		ENSMUSG00000036983	Tfb1m	20
ENSMUSG00000105864	Gm10484	20		ENSMUSG00000026492	Tfb2m	20
ENSMUSG00000073367	Gm10493	9		ENSMUSG00000009733	Tfcp2	19
ENSMUSG00000073415	Gm10501	1		ENSMUSG00000026380	Tfcp2l1	9
ENSMUSG00000073448	Gm10509	20		ENSMUSG00000038482	Tfdp1	9
ENSMUSG00000097316	Gm10516	20		ENSMUSG00000032411	Tfdp2	9
ENSMUSG00000073492	Gm10521	20		ENSMUSG00000000134	Tfe3	20
ENSMUSG00000097141	Gm10524	20		ENSMUSG00000023990	Tfeb	20
ENSMUSG00000073528	Gm10530	20		ENSMUSG00000022757	Tfg	9
ENSMUSG00000073594	Gm10545	20		ENSMUSG00000029345	Tfip11	20
ENSMUSG00000073607	Gm10548	20		ENSMUSG00000027082	Tfpi	20
ENSMUSG00000073647	Gm10557	20		ENSMUSG00000006335	Tfpt	20

ENSMUSG00000073656	Gm10558	20		ENSMUSG00000029716	Tfr2	20
ENSMUSG00000097649	Gm10561	20		ENSMUSG00000022797	Tfrc	20
ENSMUSG00000073737	Gm10566	9		ENSMUSG00000022130	Tgds	20
ENSMUSG00000102348	Gm10568	18		ENSMUSG00000029999	Tgfa	9
ENSMUSG00000073752	Gm10570	20		ENSMUSG00000002603	Tgfb1	20
ENSMUSG00000073753	Gm10571	20		ENSMUSG00000030782	Tgfb1i1	16
ENSMUSG00000073787	Gm10575	20		ENSMUSG00000039239	Tgfb2	20
ENSMUSG00000073985	Gm10602	20		ENSMUSG00000021253	Tgfb3	20
ENSMUSG00000097974	Gm10605	9		ENSMUSG00000035493	Tgfbi	20
ENSMUSG00000111197	Gm10608	20		ENSMUSG00000007613	Tgfbr1	20
ENSMUSG00000108599	Gm10616	20		ENSMUSG00000032440	Tgfbr2	20
ENSMUSG00000074113	Gm10629	20		ENSMUSG00000029287	Tgfbr3	1
ENSMUSG00000074178	Gm10638	20		ENSMUSG00000089736	Tgfbr3l	20
ENSMUSG00000074213	Gm10642	20		ENSMUSG00000070939	Tgfbrap1	19
ENSMUSG00000074215	Gm10643	20		ENSMUSG00000047407	Tgif1	20
ENSMUSG00000074219	Gm10644	20		ENSMUSG00000062175	Tgif2	20
ENSMUSG00000074228	Gm10645	20		ENSMUSG00000037820	Tgm2	20
ENSMUSG00000074232	Gm10647	20		ENSMUSG00000025787	Tgm4	20
ENSMUSG00000074235	Gm10649	20		ENSMUSG00000079103	Tgm7	20
ENSMUSG00000074243	Gm10651	20		ENSMUSG00000056429	Tgoln1	9
ENSMUSG00000074252	Gm10654	20		ENSMUSG00000028233	Tgs1	20
ENSMUSG00000103486	Gm10657	6		ENSMUSG00000000214	Th	17
ENSMUSG00000074355	Gm10676	20		ENSMUSG00000017713	Tha1	20
ENSMUSG00000074385	Gm10684	18		ENSMUSG00000024251	Thada	12
ENSMUSG00000097617	Gm10687	20		ENSMUSG00000037214	Thap1	20
ENSMUSG00000074412	Gm10689	20		ENSMUSG00000036442	Thap11	20
ENSMUSG00000079884	Gm10698	20		ENSMUSG00000030753	Thap12	20
ENSMUSG00000074461	Gm10699	20		ENSMUSG00000020137	Thap2	20
ENSMUSG00000074467	Gm10702	20		ENSMUSG00000039759	Thap3	20
ENSMUSG00000074506	Gm10705	20		ENSMUSG00000026279	Thap4	20
ENSMUSG00000074508	Gm10706	9		ENSMUSG00000102644	Thap6	20
ENSMUSG00000074516	Gm10709	10		ENSMUSG00000022760	Thap7	20
ENSMUSG00000074517	Gm10710	20		ENSMUSG00000074743	Thbd	12
ENSMUSG00000103345	Gm10723	19		ENSMUSG00000040152	Thbs1	20
ENSMUSG00000103701	Gm10728	1		ENSMUSG00000023885	Thbs2	9
ENSMUSG00000096842	Gm10736	12		ENSMUSG00000028047	Thbs3	3
ENSMUSG00000074776	Gm10754	9		ENSMUSG00000021702	Thbs4	1
ENSMUSG00000074807	Gm10762	20		ENSMUSG00000020317	Theg	1
ENSMUSG00000074826	Gm10767	20		ENSMUSG00000028145	Them4	20
ENSMUSG00000096856	Gm10778	20		ENSMUSG00000056665	Them6	2
ENSMUSG00000085169	Gm10785	20		ENSMUSG00000037731	Themis2	20
ENSMUSG00000113856	Gm10790	20		ENSMUSG00000011254	Thg1l	20
ENSMUSG00000115518	Gm10791	20		ENSMUSG00000048550	Thnsl1	20
ENSMUSG00000075014	Gm10800	20		ENSMUSG00000054474	Thnsl2	20
ENSMUSG00000075015	Gm10801	20		ENSMUSG00000024287	Thoc1	20
ENSMUSG00000097404	Gm10814	1		ENSMUSG00000037475	Thoc2	2
ENSMUSG00000117710	Gm10817	20		ENSMUSG00000025872	Thoc3	12
ENSMUSG00000075268	Gm10819	20		ENSMUSG00000034274	Thoc5	20
ENSMUSG00000096977	Gm10827	12		ENSMUSG00000041319	Thoc6	20
ENSMUSG00000075525	Gm10849	1		ENSMUSG00000053453	Thoc7	9
ENSMUSG00000075538	Gm10855	7		ENSMUSG00000004929	Thop1	3
ENSMUSG00000104284	Gm10862	3		ENSMUSG00000022847	Thpo	20
ENSMUSG00000075555	Gm10863	1		ENSMUSG00000058756	Thra	9
ENSMUSG00000078097	Gm10913	20		ENSMUSG00000043962	Thrap3	1

ENSMUSG00000100862	Gm10925	18		ENSMUSG00000021779	Thrb	20
ENSMUSG00000113792	Gm10933	20		ENSMUSG00000035686	Thrsp	9
ENSMUSG00000108926	Gm10988	20		ENSMUSG00000031480	Thsd1	1
ENSMUSG00000109879	Gm10997	9		ENSMUSG00000032289	Thsd4	20
ENSMUSG00000094475	Gm11007	20		ENSMUSG00000032625	Thsd7a	7
ENSMUSG00000079414	Gm11110	20		ENSMUSG00000042581	Thsd7b	20
ENSMUSG00000079564	Gm11149	20		ENSMUSG00000045691	Thtpa	20
ENSMUSG00000083245	Gm11185	9		ENSMUSG00000030942	Thumpd1	9
ENSMUSG00000058073	Gm11189	20		ENSMUSG00000024246	Thumpd2	20
ENSMUSG00000055045	Gm11190	1		ENSMUSG00000030264	Thumpd3	20
ENSMUSG00000082110	Gm11196	20		ENSMUSG00000032011	Thy1	9
ENSMUSG00000084969	Gm11205	10		ENSMUSG00000035443	Thyn1	18
ENSMUSG00000082998	Gm11221	20		ENSMUSG00000071337	Tia1	2
ENSMUSG00000085627	Gm11222	20		ENSMUSG00000115568	Tiaf2	20
ENSMUSG00000046341	Gm11223	20		ENSMUSG00000030846	Tial1	20
ENSMUSG00000082432	Gm11231	20		ENSMUSG00000002489	Tiam1	20
ENSMUSG00000083372	Gm11235	18		ENSMUSG00000023800	Tiam2	9
ENSMUSG00000081936	Gm11244	20		ENSMUSG00000047123	Ticam1	1
ENSMUSG00000083087	Gm11249	20		ENSMUSG00000056130	Ticam2	20
ENSMUSG00000083496	Gm11263	12		ENSMUSG00000033191	Tie1	20
ENSMUSG00000087413	Gm11266	20		ENSMUSG00000046688	Tifa	19
ENSMUSG00000079941	Gm11273	12		ENSMUSG00000049625	Tifab	20
ENSMUSG00000085603	Gm11346	20		ENSMUSG00000038028	Tigar	1
ENSMUSG00000060198	Gm11353	20		ENSMUSG00000049232	Tigd2	20
ENSMUSG00000113061	Gm11361	20		ENSMUSG00000044390	Tigd3	20
ENSMUSG00000082861	Gm11362	20		ENSMUSG00000103906	Tigd5	9
ENSMUSG00000081208	Gm11400	20		ENSMUSG00000039994	Timeless	13
ENSMUSG00000082553	Gm11404	20		ENSMUSG00000027076	Timm10	20
ENSMUSG00000083767	Gm11405	9		ENSMUSG00000089847	Timm10b	20
ENSMUSG00000082179	Gm11407	18		ENSMUSG00000020219	Timm13	3
ENSMUSG00000073130	Gm1141	20		ENSMUSG00000062580	Timm17a	20
ENSMUSG00000084996	Gm11419	20		ENSMUSG00000031158	Timm17b	20
ENSMUSG00000085175	Gm11423	20		ENSMUSG00000024645	Timm21	20
ENSMUSG00000083876	Gm11439	20		ENSMUSG00000020843	Timm22	18
ENSMUSG00000082917	Gm11440	20		ENSMUSG00000013701	Timm23	20
ENSMUSG00000083567	Gm11451	20		ENSMUSG00000048429	Timm29	1
ENSMUSG00000085538	Gm11455	20		ENSMUSG00000002949	Timm44	3
ENSMUSG00000081946	Gm11472	20		ENSMUSG00000003438	Timm50	20
ENSMUSG00000083992	Gm11478	3		ENSMUSG00000048007	Timm8a1	20
ENSMUSG00000081871	Gm11488	20		ENSMUSG00000039016	Timm8b	12
ENSMUSG00000084411	Gm11510	20		ENSMUSG00000021079	Timm9	20
ENSMUSG00000083268	Gm11511	20		ENSMUSG00000002846	Timmdc1	20
ENSMUSG00000081604	Gm11518	20		ENSMUSG00000017466	Timp2	9
ENSMUSG00000085143	Gm11520	20		ENSMUSG00000020044	Timp3	20
ENSMUSG00000083337	Gm11539	12		ENSMUSG00000030317	Timp4	4
ENSMUSG00000085051	Gm11542	18		ENSMUSG00000028776	Tinagl1	20
ENSMUSG00000085007	Gm11549	20		ENSMUSG00000007589	Tinf2	20
ENSMUSG00000082900	Gm11557	20		ENSMUSG00000034640	Tiparp	20
ENSMUSG00000086688	Gm11560	19		ENSMUSG00000032397	Tipin	20
ENSMUSG00000085262	Gm11574	20		ENSMUSG00000040843	Tiprl	20
ENSMUSG00000082160	Gm11578	20		ENSMUSG00000032041	Tirap	20
ENSMUSG00000086680	Gm11592	20		ENSMUSG00000012296	Tjap1	20
ENSMUSG00000082456	Gm11598	20		ENSMUSG00000030516	Tjp1	20
ENSMUSG00000081643	Gm11605	20		ENSMUSG00000024812	Tjp2	20

ENSMUSG00000087042	Gm11611	20		ENSMUSG00000034917	Tjp3	13
ENSMUSG00000085586	Gm11613	20		ENSMUSG00000035824	Tk2	20
ENSMUSG00000081965	Gm11620	20		ENSMUSG00000034371	Tkfc	20
ENSMUSG00000078640	Gm11627	1		ENSMUSG00000021957	Tkt	6
ENSMUSG00000082487	Gm11628	19		ENSMUSG00000019437	Tlcd1	20
ENSMUSG00000082715	Gm11633	20		ENSMUSG00000038217	Tlcd2	20
ENSMUSG00000040838	Gm11639	19		ENSMUSG00000008305	Tle1	9
ENSMUSG00000086794	Gm11642	20		ENSMUSG00000034771	Tle2	6
ENSMUSG00000080895	Gm11645	20		ENSMUSG00000032280	Tle3	20
ENSMUSG00000087184	Gm11650	20		ENSMUSG00000024642	Tle4	9
ENSMUSG00000083465	Gm11652	20		ENSMUSG00000054452	Tle5	6
ENSMUSG00000081270	Gm11653	20		ENSMUSG00000034758	Tle6	20
ENSMUSG00000082706	Gm11663	20		ENSMUSG00000041997	Tlk1	9
ENSMUSG00000075437	Gm11681	20		ENSMUSG00000020694	Tlk2	19
ENSMUSG00000056687	Gm11696	20		ENSMUSG00000053626	Tll1	20
ENSMUSG00000083394	Gm11703	20		ENSMUSG00000025013	Tll2	20
ENSMUSG00000086442	Gm11722	20		ENSMUSG00000028465	Tln1	20
ENSMUSG00000080810	Gm11737	20		ENSMUSG00000052698	Tln2	12
ENSMUSG00000084306	Gm11743	20		ENSMUSG00000070462	Tlnrd1	9
ENSMUSG00000084052	Gm11745	20		ENSMUSG00000044827	Tlr1	20
ENSMUSG00000084887	Gm11749	20		ENSMUSG00000062545	Tlr12	20
ENSMUSG00000085548	Gm11753	20		ENSMUSG00000033777	Tlr13	20
ENSMUSG00000086547	Gm11755	20		ENSMUSG00000027995	Tlr2	20
ENSMUSG00000081048	Gm11760	20		ENSMUSG00000031639	Tlr3	20
ENSMUSG00000085207	Gm11767	19		ENSMUSG00000039005	Tlr4	3
ENSMUSG00000085636	Gm11769	20		ENSMUSG00000079164	Tlr5	20
ENSMUSG00000085501	Gm11772	20		ENSMUSG00000044583	Tlr7	20
ENSMUSG00000068240	Gm11808	12		ENSMUSG00000045322	Tlr9	20
ENSMUSG00000084215	Gm11814	12		ENSMUSG00000028563	Tm2d1	20
ENSMUSG00000083328	Gm11826	20		ENSMUSG00000031556	Tm2d2	12
ENSMUSG00000081239	Gm11836	12		ENSMUSG00000078681	Tm2d3	2
ENSMUSG00000086587	Gm11837	20		ENSMUSG00000027800	Tm4sf1	14
ENSMUSG00000083246	Gm11839	20		ENSMUSG00000038623	Tm6sf1	18
ENSMUSG00000081593	Gm11841	20		ENSMUSG00000036151	Tm6sf2	20
ENSMUSG00000060989	Gm11847	20		ENSMUSG00000024799	Tm7sf2	20
ENSMUSG00000081026	Gm11855	20		ENSMUSG00000040234	Tm7sf3	20
ENSMUSG00000050550	Gm11868	1		ENSMUSG00000002320	Tm9sf1	20
ENSMUSG00000084408	Gm11870	20		ENSMUSG00000025544	Tm9sf2	1
ENSMUSG00000086017	Gm11872	20		ENSMUSG00000025016	Tm9sf3	3
ENSMUSG00000082876	Gm11889	20		ENSMUSG00000068040	Tm9sf4	20
ENSMUSG00000084179	Gm11890	20		ENSMUSG00000025591	Tma16	20
ENSMUSG00000085885	Gm11906	20		ENSMUSG00000091537	Tma7	10
ENSMUSG00000082674	Gm11914	20		ENSMUSG00000059058	Tma7-ps	20
ENSMUSG00000087692	Gm11940	20		ENSMUSG00000006301	Tmbim1	16
ENSMUSG00000094344	Gm11942	2		ENSMUSG00000020225	Tmbim4	20
ENSMUSG00000084941	Gm11944	10		ENSMUSG00000023010	Tmbim6	20
ENSMUSG00000081059	Gm11945	20		ENSMUSG00000038540	Tmc3	1
ENSMUSG00000087055	Gm11948	20		ENSMUSG00000019734	Tmc4	20
ENSMUSG00000082816	Gm11953	20		ENSMUSG00000025572	Tmc6	9
ENSMUSG00000084170	Gm11954	9		ENSMUSG00000042246	Tmc7	4
ENSMUSG00000081021	Gm11964	20		ENSMUSG00000050106	Tmc8	20
ENSMUSG00000080904	Gm11966	20		ENSMUSG00000030126	Tmcc1	20
ENSMUSG00000083209	Gm11969	1		ENSMUSG00000042066	Tmcc2	18
ENSMUSG00000086839	Gm11973	20		ENSMUSG00000020023	Tmcc3	15

ENSMUSG00000083819	Gm11977	20		ENSMUSG00000086269	Tmcc3os	18
ENSMUSG00000040978	Gm11992	1		ENSMUSG00000052428	Tmco1	12
ENSMUSG00000101841	Gm11993	20		ENSMUSG00000038497	Tmco3	20
ENSMUSG00000081631	Gm12005	20		ENSMUSG00000041143	Tmco4	20
ENSMUSG00000084183	Gm12009	20		ENSMUSG00000027355	Tmco5	20
ENSMUSG00000078965	Gm12033	12		ENSMUSG00000041255	Tmco5b	20
ENSMUSG00000084048	Gm12042	20		ENSMUSG00000006850	Tmco6	20
ENSMUSG00000084081	Gm12057	20		ENSMUSG00000032180	Tmed1	12
ENSMUSG00000082576	Gm12058	20		ENSMUSG00000021248	Tmed10	13
ENSMUSG00000085665	Gm12059	20		ENSMUSG00000114251	Tmed10-ps	20
ENSMUSG00000085167	Gm12063	18		ENSMUSG00000029390	Tmed2	20
ENSMUSG00000069939	Gm12070	20		ENSMUSG00000032353	Tmed3	20
ENSMUSG00000084064	Gm12079	20		ENSMUSG00000004394	Tmed4	20
ENSMUSG00000085165	Gm12089	20		ENSMUSG00000063406	Tmed5	19
ENSMUSG00000081712	Gm12096	9		ENSMUSG00000033184	Tmed7	7
ENSMUSG00000081887	Gm12097	20		ENSMUSG00000034111	Tmed8	1
ENSMUSG00000082078	Gm12098	1		ENSMUSG00000058569	Tmed9	3
ENSMUSG00000083990	Gm12101	20		ENSMUSG00000028347	Tmeff1	9
ENSMUSG00000084146	Gm12104	20		ENSMUSG00000026109	Tmeff2	10
ENSMUSG00000081254	Gm12112	20		ENSMUSG00000069763	Tmem100	20
ENSMUSG00000099902	Gm12115	20		ENSMUSG00000020921	Tmem101	20
ENSMUSG00000098456	Gm12117	20		ENSMUSG00000045980	Tmem104	20
ENSMUSG00000086311	Gm12122	20		ENSMUSG00000034947	Tmem106a	20
ENSMUSG00000082087	Gm12138	2		ENSMUSG00000029571	Tmem106b	20
ENSMUSG00000081251	Gm12164	12		ENSMUSG00000052369	Tmem106c	2
ENSMUSG00000069899	Gm12166	20		ENSMUSG00000020895	Tmem107	3
ENSMUSG00000082530	Gm12168	20		ENSMUSG00000042757	Tmem108	18
ENSMUSG00000080776	Gm12174	12		ENSMUSG00000034659	Tmem109	3
ENSMUSG00000081952	Gm12178	20		ENSMUSG00000043284	Tmem11	20
ENSMUSG00000083460	Gm12182	20		ENSMUSG00000010045	Tmem115	20
ENSMUSG00000082454	Gm12183	18		ENSMUSG00000029452	Tmem116	1
ENSMUSG00000078154	Gm12184	20		ENSMUSG00000063296	Tmem117	9
ENSMUSG00000083061	Gm12191	7		ENSMUSG00000054675	Tmem119	20
ENSMUSG00000084302	Gm12194	20		ENSMUSG00000039886	Tmem120a	20
ENSMUSG00000085564	Gm12198	18		ENSMUSG00000054434	Tmem120b	20
ENSMUSG00000086920	Gm12207	20		ENSMUSG00000049036	Tmem121	13
ENSMUSG00000085707	Gm12212	20		ENSMUSG00000094626	Tmem121b	9
ENSMUSG00000081769	Gm12216	20		ENSMUSG00000050912	Tmem123	20
ENSMUSG00000078151	Gm12226	20		ENSMUSG00000050854	Tmem125	20
ENSMUSG00000082503	Gm12229	20		ENSMUSG00000030615	Tmem126a	20
ENSMUSG00000044751	Gm12231	20		ENSMUSG00000030614	Tmem126b	13
ENSMUSG00000086020	Gm12239	20		ENSMUSG00000034850	Tmem127	12
ENSMUSG00000084384	Gm12251	20		ENSMUSG00000067365	Tmem128	12
ENSMUSG00000085342	Gm12254	20		ENSMUSG00000019295	Tmem129	20
ENSMUSG00000072915	Gm12258	20		ENSMUSG00000043388	Tmem130	17
ENSMUSG00000080152	Gm12260	20		ENSMUSG00000026116	Tmem131	18
ENSMUSG00000084145	Gm12263	5		ENSMUSG00000033767	Tmem131l	1
ENSMUSG00000081944	Gm12276	20		ENSMUSG00000024736	Tmem132a	3
ENSMUSG00000087120	Gm12279	20		ENSMUSG00000070498	Tmem132b	7
ENSMUSG00000081600	Gm12286	20		ENSMUSG00000034324	Tmem132c	18
ENSMUSG00000083935	Gm12294	20		ENSMUSG00000034310	Tmem132d	1
ENSMUSG00000084967	Gm12296	12		ENSMUSG00000020701	Tmem132e	16
ENSMUSG00000082145	Gm12312	20		ENSMUSG00000039428	Tmem135	3
ENSMUSG00000087116	Gm12324	20		ENSMUSG00000048503	Tmem136	6

ENSMUSG00000085251	Gm12326	20		ENSMUSG00000024666	Tmem138	20
ENSMUSG00000081552	Gm12328	3		ENSMUSG00000057137	Tmem140	20
ENSMUSG00000081485	Gm12338	20		ENSMUSG00000026939	Tmem141	9
ENSMUSG00000087059	Gm12339	20		ENSMUSG00000002781	Tmem143	20
ENSMUSG00000102038	Gm12345	20		ENSMUSG00000027956	Tmem144	1
ENSMUSG00000083899	Gm12346	9		ENSMUSG00000043843	Tmem145	18
ENSMUSG00000095588	Gm12350	19		ENSMUSG00000006315	Tmem147	12
ENSMUSG00000078772	Gm12353	1		ENSMUSG00000097320	Tmem147os	20
ENSMUSG00000078134	Gm12355	20		ENSMUSG00000025933	Tmem14a	1
ENSMUSG00000075576	Gm12359	20		ENSMUSG00000021361	Tmem14c	20
ENSMUSG00000082762	Gm12366	20		ENSMUSG00000046456	Tmem150b	19
ENSMUSG00000084828	Gm12367	20		ENSMUSG00000050640	Tmem150c	9
ENSMUSG00000082169	Gm12369	20		ENSMUSG00000061451	Tmem151a	16
ENSMUSG00000084898	Gm12371	20		ENSMUSG00000096847	Tmem151b	19
ENSMUSG00000056412	Gm12396	20		ENSMUSG00000056498	Tmem154	12
ENSMUSG00000081309	Gm12397	20		ENSMUSG00000037913	Tmem156	20
ENSMUSG00000082186	Gm12403	20		ENSMUSG00000054871	Tmem158	19
ENSMUSG00000087623	Gm12404	20		ENSMUSG00000030917	Tmem159	20
ENSMUSG00000085980	Gm12408	20		ENSMUSG00000019158	Tmem160	20
ENSMUSG00000081559	Gm12411	20		ENSMUSG00000002342	Tmem161a	1
ENSMUSG00000083044	Gm12416	20		ENSMUSG00000035762	Tmem161b	20
ENSMUSG00000083139	Gm12418	20		ENSMUSG00000026347	Tmem163	9
ENSMUSG00000082185	Gm12428	20		ENSMUSG00000047045	Tmem164	20
ENSMUSG00000081152	Gm12430	20		ENSMUSG00000029234	Tmem165	20
ENSMUSG00000081953	Gm12435	20		ENSMUSG00000012422	Tmem167	20
ENSMUSG00000080850	Gm12439	20		ENSMUSG00000068732	Tmem167b	10
ENSMUSG00000082194	Gm12444	20		ENSMUSG00000029569	Tmem168	20
ENSMUSG00000080816	Gm12447	20		ENSMUSG00000026188	Tmem169	17
ENSMUSG00000082725	Gm12451	18		ENSMUSG00000049904	Tmem17	20
ENSMUSG00000087142	Gm12454	1		ENSMUSG00000031953	Tmem170	6
ENSMUSG00000083153	Gm12459	20		ENSMUSG00000087370	Tmem170b	3
ENSMUSG00000086211	Gm12462	20		ENSMUSG00000024349	Tmem173	20
ENSMUSG00000081516	Gm12470	1		ENSMUSG00000013495	Tmem175	20
ENSMUSG00000043770	Gm12481	12		ENSMUSG00000023367	Tmem176a	20
ENSMUSG00000081975	Gm12482	20		ENSMUSG00000029810	Tmem176b	20
ENSMUSG00000056411	Gm12500	16		ENSMUSG00000036975	Tmem177	20
ENSMUSG00000083627	Gm12502	20		ENSMUSG00000024245	Tmem178	5
ENSMUSG00000074281	Gm12522	20		ENSMUSG00000057716	Tmem178b	3
ENSMUSG00000078601	Gm12525	20		ENSMUSG00000054013	Tmem179	20
ENSMUSG00000083979	Gm12527	20		ENSMUSG00000118346	Tmem179b	20
ENSMUSG00000085654	Gm12536	9		ENSMUSG00000043061	Tmem18	20
ENSMUSG00000083353	Gm12540	20		ENSMUSG00000038141	Tmem181a	9
ENSMUSG00000080772	Gm12543	20		ENSMUSG00000096780	Tmem181b-p	9
ENSMUSG00000081078	Gm12568	20		ENSMUSG00000093880	Tmem181c-p	20
ENSMUSG00000085235	Gm12576	20		ENSMUSG00000042305	Tmem183a	20
ENSMUSG00000083834	Gm12577	12		ENSMUSG00000009035	Tmem184b	20
ENSMUSG00000084401	Gm12586	20		ENSMUSG00000031617	Tmem184c	20
ENSMUSG00000053263	Gm12592	20		ENSMUSG00000073139	Tmem185a	20
ENSMUSG00000081325	Gm12599	20		ENSMUSG00000098923	Tmem185b	20
ENSMUSG00000085147	Gm12609	20		ENSMUSG00000043140	Tmem186	20
ENSMUSG00000086035	Gm12610	1		ENSMUSG00000090213	Tmem189	20
ENSMUSG00000087128	Gm12655	20		ENSMUSG00000069520	Tmem19	20
ENSMUSG00000063314	Gm12657	20		ENSMUSG00000055692	Tmem191c	3
ENSMUSG00000101316	Gm12663	20		ENSMUSG00000025521	Tmem192	12

ENSMUSG00000100863	Gm12669	20		ENSMUSG00000048004	Tmem196	3
ENSMUSG00000070891	Gm12689	16		ENSMUSG00000051703	Tmem198	20
ENSMUSG00000083179	Gm12693	20		ENSMUSG00000047090	Tmem198b	20
ENSMUSG00000084159	Gm12696	20		ENSMUSG00000051232	Tmem199	20
ENSMUSG00000082683	Gm12697	20		ENSMUSG00000049420	Tmem200a	5
ENSMUSG00000086385	Gm12701	20		ENSMUSG00000070720	Tmem200b	9
ENSMUSG00000081811	Gm12713	20		ENSMUSG00000095407	Tmem200c	19
ENSMUSG00000059195	Gm12715	9		ENSMUSG00000044700	Tmem201	20
ENSMUSG00000081696	Gm12717	20		ENSMUSG00000049526	Tmem202	3
ENSMUSG00000081819	Gm12722	20		ENSMUSG00000078201	Tmem203	20
ENSMUSG00000081451	Gm12734	12		ENSMUSG00000024168	Tmem204	12
ENSMUSG00000085093	Gm12737	20		ENSMUSG00000040883	Tmem205	13
ENSMUSG00000084968	Gm12743	10		ENSMUSG00000014856	Tmem208	20
ENSMUSG00000083512	Gm12749	20		ENSMUSG00000029782	Tmem209	20
ENSMUSG00000062554	Gm12751	20		ENSMUSG00000043164	Tmem212	14
ENSMUSG00000085105	Gm12758	1		ENSMUSG00000038828	Tmem214	20
ENSMUSG00000082928	Gm12785	20		ENSMUSG00000046593	Tmem215	20
ENSMUSG00000081121	Gm12791	20		ENSMUSG00000024667	Tmem216	20
ENSMUSG00000082264	Gm12799	20		ENSMUSG00000032121	Tmem218	20
ENSMUSG00000081068	Gm12804	20		ENSMUSG00000060538	Tmem219	20
ENSMUSG00000081813	Gm12806	20		ENSMUSG00000050270	Tmem220	20
ENSMUSG00000083011	Gm12816	10		ENSMUSG00000028857	Tmem222	19
ENSMUSG00000085278	Gm12841	20		ENSMUSG00000117924	Tmem223	19
ENSMUSG00000084260	Gm12844	20		ENSMUSG00000048022	Tmem229a	18
ENSMUSG00000081046	Gm12846	19		ENSMUSG00000046157	Tmem229b	18
ENSMUSG00000082941	Gm12850	20		ENSMUSG00000027341	Tmem230	20
ENSMUSG00000081733	Gm12852	20		ENSMUSG00000031951	Tmem231	9
ENSMUSG00000082515	Gm12902	20		ENSMUSG00000045036	Tmem232	9
ENSMUSG00000085875	Gm12905	1		ENSMUSG00000028797	Tmem234	20
ENSMUSG00000083104	Gm12917	20		ENSMUSG00000038079	Tmem237	13
ENSMUSG00000066315	Gm12918	18		ENSMUSG00000030431	Tmem238	20
ENSMUSG00000082381	Gm12928	20		ENSMUSG00000084845	Tmem240	9
ENSMUSG00000084099	Gm12929	20		ENSMUSG00000049411	Tmem241	20
ENSMUSG00000087011	Gm12930	1		ENSMUSG00000004945	Tmem242	12
ENSMUSG00000082888	Gm12934	20		ENSMUSG00000079659	Tmem243	20
ENSMUSG00000085334	Gm12940	20		ENSMUSG00000055296	Tmem245	3
ENSMUSG00000084088	Gm12941	20		ENSMUSG00000039611	Tmem246	18
ENSMUSG00000087299	Gm12953	20		ENSMUSG00000053094	Tmem248	20
ENSMUSG00000100514	Gm12960	20		ENSMUSG00000002032	Tmem25	18
ENSMUSG00000082686	Gm12961	20		ENSMUSG00000087679	Tmem250-ps	20
ENSMUSG00000085517	Gm12963	20		ENSMUSG00000046675	Tmem251	20
ENSMUSG00000070729	Gm12966	20		ENSMUSG00000048572	Tmem252	20
ENSMUSG00000087449	Gm12971	20		ENSMUSG00000036502	Tmem255a	17
ENSMUSG00000082711	Gm12987	20		ENSMUSG00000038457	Tmem255b	13
ENSMUSG00000082743	Gm12988	20		ENSMUSG00000070394	Tmem256	12
ENSMUSG00000083678	Gm12989	20		ENSMUSG00000036372	Tmem258	12
ENSMUSG00000081136	Gm12990	20		ENSMUSG00000013858	Tmem259	20
ENSMUSG00000085667	Gm12992	9		ENSMUSG00000060044	Tmem26	20
ENSMUSG00000082063	Gm12993	20		ENSMUSG00000036339	Tmem260	20
ENSMUSG00000086417	Gm12996	20		ENSMUSG00000060935	Tmem263	7
ENSMUSG00000087352	Gm12999	20		ENSMUSG00000032313	Tmem266	20
ENSMUSG00000087084	Gm13016	9		ENSMUSG00000074634	Tmem267	20
ENSMUSG00000086159	Gm13025	20		ENSMUSG00000045917	Tmem268	20
ENSMUSG00000087698	Gm13031	3		ENSMUSG00000028642	Tmem269	20

ENSMUSG00000083816	Gm13033	20		ENSMUSG00000041707	Tmem273	20
ENSMUSG00000086806	Gm13054	20		ENSMUSG00000071719	Tmem28	2
ENSMUSG00000086665	Gm13067	20		ENSMUSG00000041353	Tmem29	20
ENSMUSG00000083811	Gm13071	13		ENSMUSG00000032328	Tmem30a	20
ENSMUSG00000044377	Gm13080	20		ENSMUSG00000022753	Tmem30c	20
ENSMUSG00000081227	Gm13082	20		ENSMUSG00000037720	Tmem33	20
ENSMUSG00000085213	Gm13091	9		ENSMUSG00000033578	Tmem35a	12
ENSMUSG00000085787	Gm13092	20		ENSMUSG00000070737	Tmem35b	20
ENSMUSG00000087094	Gm13093	20		ENSMUSG00000050777	Tmem37	20
ENSMUSG00000087437	Gm13112	6		ENSMUSG00000031791	Tmem38a	9
ENSMUSG00000083708	Gm13123	20		ENSMUSG00000028420	Tmem38b	20
ENSMUSG00000070610	Gm13127	20		ENSMUSG00000002845	Tmem39a	20
ENSMUSG00000082925	Gm13135	20		ENSMUSG00000053730	Tmem39b	18
ENSMUSG00000081179	Gm13136	1		ENSMUSG00000022856	Tmem41a	11
ENSMUSG00000083027	Gm13140	2		ENSMUSG00000047554	Tmem41b	20
ENSMUSG00000081405	Gm13142	20		ENSMUSG00000066233	Tmem42	12
ENSMUSG00000082248	Gm13161	20		ENSMUSG00000030095	Tmem43	20
ENSMUSG00000082779	Gm13162	20		ENSMUSG00000022537	Tmem44	9
ENSMUSG00000086921	Gm13189	18		ENSMUSG00000025666	Tmem47	1
ENSMUSG00000086670	Gm13194	20		ENSMUSG00000028822	Tmem50a	19
ENSMUSG00000086606	Gm13205	18		ENSMUSG00000022964	Tmem50b	20
ENSMUSG00000083885	Gm13213	20		ENSMUSG00000048772	Tmem53	20
ENSMUSG00000082953	Gm13217	20		ENSMUSG00000028132	Tmem56	18
ENSMUSG00000096233	Gm13238	6		ENSMUSG00000028618	Tmem59	3
ENSMUSG00000073716	Gm13241	20		ENSMUSG00000035964	Tmem59l	9
ENSMUSG00000084105	Gm13252	20		ENSMUSG00000045435	Tmem60	20
ENSMUSG00000086850	Gm13257	9		ENSMUSG00000054484	Tmem62	20
ENSMUSG00000070271	Gm13268	3		ENSMUSG00000026519	Tmem63a	16
ENSMUSG00000087086	Gm13269	20		ENSMUSG00000036026	Tmem63b	3
ENSMUSG00000086358	Gm13270	20		ENSMUSG00000034145	Tmem63c	20
ENSMUSG00000086006	Gm13293	20		ENSMUSG00000043252	Tmem64	20
ENSMUSG00000083633	Gm13312	9		ENSMUSG00000062373	Tmem65	18
ENSMUSG00000083305	Gm13315	1		ENSMUSG00000049488	Tmem67	20
ENSMUSG00000085193	Gm13322	20		ENSMUSG00000028232	Tmem68	2
ENSMUSG00000082235	Gm13326	20		ENSMUSG00000055900	Tmem69	20
ENSMUSG00000085680	Gm13327	20		ENSMUSG00000025940	Tmem70	20
ENSMUSG00000081128	Gm13328	20		ENSMUSG00000036944	Tmem71	20
ENSMUSG00000081519	Gm13331	20		ENSMUSG00000048108	Tmem72	13
ENSMUSG00000082884	Gm13339	9		ENSMUSG00000054409	Tmem74	20
ENSMUSG00000083563	Gm13340	20		ENSMUSG00000044364	Tmem74b	20
ENSMUSG00000083863	Gm13341	20		ENSMUSG00000001420	Tmem79	20
ENSMUSG00000082516	Gm13342	20		ENSMUSG00000024180	Tmem8	19
ENSMUSG00000082520	Gm13349	20		ENSMUSG00000025505	Tmem80	20
ENSMUSG00000083070	Gm13350	2		ENSMUSG00000048174	Tmem81	20
ENSMUSG00000083650	Gm13357	19		ENSMUSG00000043085	Tmem82	20
ENSMUSG00000060795	Gm13363	12		ENSMUSG00000010307	Tmem86a	20
ENSMUSG00000084383	Gm13370	12		ENSMUSG00000045282	Tmem86b	20
ENSMUSG00000075514	Gm13375	20		ENSMUSG00000033808	Tmem87a	20
ENSMUSG00000085206	Gm13380	20		ENSMUSG00000014353	Tmem87b	3
ENSMUSG00000087079	Gm13389	18		ENSMUSG00000045377	Tmem88	9
ENSMUSG00000083773	Gm13394	20		ENSMUSG00000073680	Tmem88b	15
ENSMUSG00000085987	Gm13403	20		ENSMUSG00000078716	Tmem8b	19
ENSMUSG00000086904	Gm13404	20		ENSMUSG00000026411	Tmem9	7
ENSMUSG00000081992	Gm13408	20		ENSMUSG00000061702	Tmem91	12

ENSMUSG00000083116	Gm13410	20		ENSMUSG00000020747	Tmem94	9
ENSMUSG00000087635	Gm13414	20		ENSMUSG00000037278	Tmem97	1
ENSMUSG00000084241	Gm13416	18		ENSMUSG00000035413	Tmem98	20
ENSMUSG00000085929	Gm13421	12		ENSMUSG00000031021	Tmem9b	20
ENSMUSG00000084098	Gm13422	20		ENSMUSG00000030059	Tmf1	3
ENSMUSG00000081648	Gm13423	20		ENSMUSG00000049555	Tmie	6
ENSMUSG00000085224	Gm13425	1		ENSMUSG00000079834	Tmlhe	1
ENSMUSG00000083101	Gm13430	20		ENSMUSG00000028328	Tmod1	9
ENSMUSG00000083716	Gm13436	12		ENSMUSG00000032186	Tmod2	8
ENSMUSG00000075391	Gm13443	20		ENSMUSG00000058587	Tmod3	20
ENSMUSG00000086555	Gm13446	18		ENSMUSG00000019961	Tmpo	20
ENSMUSG00000083240	Gm13453	20		ENSMUSG00000079260	Tmppe	20
ENSMUSG00000082536	Gm13456	18		ENSMUSG00000072845	Tmprss11a	13
ENSMUSG00000081999	Gm13461	18		ENSMUSG00000032091	Tmprss4	20
ENSMUSG00000080994	Gm13464	12		ENSMUSG00000032268	Tmprss5	20
ENSMUSG00000085591	Gm13479	20		ENSMUSG00000016942	Tmprss6	1
ENSMUSG00000085862	Gm13483	9		ENSMUSG00000033177	Tmprss7	9
ENSMUSG00000087701	Gm13493	12		ENSMUSG00000059406	Tmprss9	9
ENSMUSG00000086349	Gm13497	20		ENSMUSG00000079523	Tmsb10	10
ENSMUSG00000083287	Gm13502	11		ENSMUSG00000089768	Tmsb15b1	20
ENSMUSG00000081272	Gm13509	20		ENSMUSG00000089996	Tmsb15b2	20
ENSMUSG00000080937	Gm13510	20		ENSMUSG00000072955	Tmsb15l	12
ENSMUSG00000085397	Gm13524	20		ENSMUSG00000049775	Tmsb4x	12
ENSMUSG00000082879	Gm13525	13		ENSMUSG00000030306	Tmtc1	6
ENSMUSG00000086939	Gm13530	20		ENSMUSG00000036019	Tmtc2	3
ENSMUSG00000086995	Gm13544	20		ENSMUSG00000036676	Tmtc3	20
ENSMUSG00000085815	Gm13548	20		ENSMUSG00000041594	Tmtc4	20
ENSMUSG00000084250	Gm13550	20		ENSMUSG00000028958	Tmub1	20
ENSMUSG00000081157	Gm13552	20		ENSMUSG00000034757	Tmub2	19
ENSMUSG00000086129	Gm13558	20		ENSMUSG00000021072	Tmx1	20
ENSMUSG00000086779	Gm13562	20		ENSMUSG00000050043	Tmx2	2
ENSMUSG00000085767	Gm13563	9		ENSMUSG00000024614	Tmx3	1
ENSMUSG00000087181	Gm13570	20		ENSMUSG00000034723	Tmx4	1
ENSMUSG00000080198	Gm13573	20		ENSMUSG00000028364	Tnc	18
ENSMUSG00000081236	Gm13574	20		ENSMUSG00000017615	Tnfaip1	20
ENSMUSG00000084104	Gm13578	20		ENSMUSG00000021281	Tnfaip2	20
ENSMUSG00000082931	Gm13586	20		ENSMUSG00000019850	Tnfaip3	20
ENSMUSG00000081299	Gm13603	20		ENSMUSG00000053475	Tnfaip6	16
ENSMUSG00000083910	Gm13604	12		ENSMUSG00000062210	Tnfaip8	13
ENSMUSG00000081257	Gm13607	20		ENSMUSG00000044469	Tnfaip8l1	20
ENSMUSG00000081480	Gm13608	20		ENSMUSG00000013707	Tnfaip8l2	20
ENSMUSG00000066068	Gm13611	20		ENSMUSG00000074345	Tnfaip8l3	18
ENSMUSG00000082345	Gm13622	20		ENSMUSG00000022074	Tnfrsf10b	20
ENSMUSG00000089902	Gm13625	20		ENSMUSG00000026321	Tnfrsf11a	18
ENSMUSG00000081307	Gm13627	20		ENSMUSG00000063727	Tnfrsf11b	20
ENSMUSG00000087301	Gm13629	18		ENSMUSG00000023905	Tnfrsf12a	9
ENSMUSG00000085386	Gm13630	20		ENSMUSG00000010142	Tnfrsf13b	20
ENSMUSG00000082190	Gm13641	20		ENSMUSG00000068105	Tnfrsf13c	1
ENSMUSG00000084087	Gm13650	9		ENSMUSG00000042333	Tnfrsf14	20
ENSMUSG00000081611	Gm13651	20		ENSMUSG00000041954	Tnfrsf18	20
ENSMUSG00000080979	Gm13675	20		ENSMUSG00000060548	Tnfrsf19	20
ENSMUSG00000081400	Gm13680	20		ENSMUSG00000030341	Tnfrsf1a	20
ENSMUSG00000083284	Gm13688	20		ENSMUSG00000028599	Tnfrsf1b	12
ENSMUSG00000081487	Gm13689	20		ENSMUSG00000023915	Tnfrsf21	1

ENSMUSG00000096337	Gm13694	2		ENSMUSG00000010751	Tnfrsf22	10
ENSMUSG00000083864	Gm13719	20		ENSMUSG00000037613	Tnfrsf23	20
ENSMUSG00000082908	Gm13736	20		ENSMUSG00000024793	Tnfrsf25	20
ENSMUSG00000085547	Gm13767	9		ENSMUSG00000029075	Tnfrsf4	9
ENSMUSG00000080977	Gm13772	20		ENSMUSG00000028965	Tnfrsf9	20
ENSMUSG00000081730	Gm13785	20		ENSMUSG00000039304	Tnfsf10	20
ENSMUSG00000083951	Gm13786	20		ENSMUSG00000097328	Tnfsf12	20
ENSMUSG00000081248	Gm13787	20		ENSMUSG00000031497	Tnfsf13b	20
ENSMUSG00000082820	Gm13803	20		ENSMUSG00000085890	Tnfsf13os	20
ENSMUSG00000086368	Gm13830	20		ENSMUSG00000035678	Tnfsf9	20
ENSMUSG00000087292	Gm13832	20		ENSMUSG00000027692	Tnik	9
ENSMUSG00000086922	Gm13835	20		ENSMUSG00000020400	Tnip1	20
ENSMUSG00000086849	Gm13840	20		ENSMUSG00000059866	Tnip2	20
ENSMUSG00000083833	Gm13841	12		ENSMUSG00000044162	Tnip3	20
ENSMUSG00000086855	Gm13844	2		ENSMUSG00000022791	Tnk2	20
ENSMUSG00000085382	Gm13861	20		ENSMUSG00000031529	Tnks	3
ENSMUSG00000082920	Gm13864	20		ENSMUSG00000033955	Tnks1bp1	5
ENSMUSG00000083306	Gm13868	20		ENSMUSG00000024811	Tnks2	2
ENSMUSG00000087185	Gm13872	20		ENSMUSG00000091898	Tnnc1	9
ENSMUSG00000081010	Gm13880	20		ENSMUSG00000026418	Tnni1	20
ENSMUSG00000081721	Gm13882	20		ENSMUSG00000035458	Tnni3	20
ENSMUSG00000087473	Gm13883	20		ENSMUSG00000064179	Tnnt1	18
ENSMUSG00000083856	Gm13886	20		ENSMUSG00000026414	Tnnt2	8
ENSMUSG00000087006	Gm13889	9		ENSMUSG00000009470	Tnpo1	16
ENSMUSG00000063684	Gm13910	20		ENSMUSG00000031691	Tnpo2	19
ENSMUSG00000084094	Gm13961	20		ENSMUSG00000012535	Tnpo3	2
ENSMUSG00000085222	Gm13974	9		ENSMUSG00000015829	Tnr	1
ENSMUSG00000081787	Gm13991	20		ENSMUSG00000039477	Tnrc18	20
ENSMUSG00000082651	Gm14001	20		ENSMUSG00000052707	Tnrc6a	18
ENSMUSG00000087056	Gm14004	20		ENSMUSG00000047888	Tnrc6b	2
ENSMUSG00000081967	Gm14017	20		ENSMUSG00000025571	Tnrc6c	20
ENSMUSG00000081264	Gm14021	20		ENSMUSG00000055322	Tns1	20
ENSMUSG00000082274	Gm14026	20		ENSMUSG00000037003	Tns2	1
ENSMUSG00000090248	Gm14027	20		ENSMUSG00000020422	Tns3	9
ENSMUSG00000087040	Gm14033	20		ENSMUSG00000017607	Tns4	20
ENSMUSG00000082399	Gm14036	20		ENSMUSG00000033327	Tnxb	20
ENSMUSG00000083879	Gm14038	20		ENSMUSG00000037573	Tob1	18
ENSMUSG00000083483	Gm14044	12		ENSMUSG00000048546	Tob2	20
ENSMUSG00000080989	Gm14048	20		ENSMUSG00000028688	Toe1	20
ENSMUSG00000083953	Gm14049	16		ENSMUSG00000035614	Togaram1	2
ENSMUSG00000089662	Gm14057	20		ENSMUSG00000045761	Togaram2	20
ENSMUSG00000085071	Gm14066	20		ENSMUSG00000025139	Tollip	19
ENSMUSG00000087158	Gm14093	20		ENSMUSG00000042870	Tom1	20
ENSMUSG00000083689	Gm14094	20		ENSMUSG00000020541	Tom1l1	20
ENSMUSG00000080870	Gm14111	20		ENSMUSG00000000538	Tom1l2	18
ENSMUSG00000084371	Gm14113	20		ENSMUSG00000093904	Tomm20	9
ENSMUSG00000042938	Gm14117	20		ENSMUSG00000022427	Tomm22	12
ENSMUSG00000083325	Gm14121	20		ENSMUSG00000018322	Tomm34	20
ENSMUSG00000083964	Gm14122	20		ENSMUSG00000002984	Tomm40	20
ENSMUSG00000081494	Gm14130	20		ENSMUSG00000005674	Tomm40l	20
ENSMUSG00000081470	Gm14131	20		ENSMUSG00000078713	Tomm5	7
ENSMUSG00000083391	Gm14148	20		ENSMUSG00000033475	Tomm6	1
ENSMUSG00000082809	Gm14150	20		ENSMUSG00000084880	Tomm6os	20
ENSMUSG00000081777	Gm14159	20		ENSMUSG00000028998	Tomm7	12

ENSMUSG00000081434	Gm14165	3		ENSMUSG00000022752	Tomm70a	9
ENSMUSG00000087433	Gm14167	20		ENSMUSG00000059323	Tonsl	9
ENSMUSG00000055494	Gm14168	1		ENSMUSG00000070544	Top1	20
ENSMUSG00000086118	Gm14169	7		ENSMUSG00000000934	Top1mt	20
ENSMUSG00000061272	Gm14173	20		ENSMUSG00000020914	Top2a	19
ENSMUSG00000062846	Gm14176	20		ENSMUSG00000017485	Top2b	2
ENSMUSG00000086496	Gm14204	3		ENSMUSG00000002814	Top3a	20
ENSMUSG00000087563	Gm14205	5		ENSMUSG00000022779	Top3b	13
ENSMUSG00000080237	Gm14239	20		ENSMUSG00000032555	Topbp1	20
ENSMUSG00000082321	Gm14253	20		ENSMUSG00000036822	Topors	1
ENSMUSG00000084067	Gm14269	20		ENSMUSG00000026849	Tor1a	20
ENSMUSG00000084013	Gm14270	20		ENSMUSG00000026466	Tor1aip1	20
ENSMUSG00000083793	Gm14274	20		ENSMUSG00000050565	Tor1aip2	20
ENSMUSG00000082163	Gm14276	20		ENSMUSG00000026848	Tor1b	20
ENSMUSG00000084113	Gm14277	20		ENSMUSG00000009563	Tor2a	3
ENSMUSG00000081740	Gm14279	12		ENSMUSG00000060519	Tor3a	20
ENSMUSG00000087445	Gm14286	19		ENSMUSG00000059555	Tor4a	20
ENSMUSG00000082329	Gm14287	20		ENSMUSG00000041272	Tox	9
ENSMUSG00000078889	Gm14288	20		ENSMUSG00000074607	Tox2	6
ENSMUSG00000086098	Gm14291	20		ENSMUSG00000043668	Tox3	3
ENSMUSG00000061897	Gm14292	20		ENSMUSG00000016831	Tox4	12
ENSMUSG00000078877	Gm14295	3		ENSMUSG00000035274	Tpbg	6
ENSMUSG00000074527	Gm14296	20		ENSMUSG00000096606	Tpbgl	20
ENSMUSG00000081344	Gm14303	3		ENSMUSG00000032741	Tpcn1	1
ENSMUSG00000078878	Gm14305	20		ENSMUSG00000048677	Tpcn2	20
ENSMUSG00000078864	Gm14322	20		ENSMUSG00000027506	Tpd52	20
ENSMUSG00000095362	Gm14325	20		ENSMUSG00000094594	Tpd52-ps	20
ENSMUSG00000078862	Gm14326	20		ENSMUSG00000000296	Tpd52l1	2
ENSMUSG00000095419	Gm14328	10		ENSMUSG00000000827	Tpd52l2	9
ENSMUSG00000086239	Gm14329	20		ENSMUSG00000020308	Tpgs1	20
ENSMUSG00000081805	Gm14335	20		ENSMUSG00000024269	Tpgs2	3
ENSMUSG00000082543	Gm14336	20		ENSMUSG00000006764	Tph2	3
ENSMUSG00000081232	Gm14373	20		ENSMUSG00000106755	Tpi-rs11	12
ENSMUSG00000087252	Gm14379	20		ENSMUSG00000023456	Tpi1	2
ENSMUSG00000084230	Gm14388	20		ENSMUSG00000029735	Tpk1	20
ENSMUSG00000078903	Gm14391	20		ENSMUSG00000032366	Tpm1	9
ENSMUSG00000078905	Gm14393	1		ENSMUSG00000028464	Tpm2	1
ENSMUSG00000090093	Gm14399	20		ENSMUSG00000027940	Tpm3	20
ENSMUSG00000078872	Gm14401	20		ENSMUSG00000058126	Tpm3-rs7	20
ENSMUSG00000094786	Gm14403	20		ENSMUSG00000031799	Tpm4	9
ENSMUSG00000083170	Gm14405	20		ENSMUSG00000021376	Tpmt	20
ENSMUSG00000078865	Gm14406	20		ENSMUSG00000020673	Tpo	20
ENSMUSG00000082023	Gm14407	20		ENSMUSG00000030894	Tpp1	9
ENSMUSG00000078870	Gm14410	20		ENSMUSG00000041763	Tpp2	20
ENSMUSG00000082765	Gm14411	20		ENSMUSG00000021573	Tppp	20
ENSMUSG00000078868	Gm14412	20		ENSMUSG00000014846	Tppp3	3
ENSMUSG00000081590	Gm14413	13		ENSMUSG00000006005	Tpr	3
ENSMUSG00000082377	Gm14415	11		ENSMUSG00000002871	Tpra1	13
ENSMUSG00000082724	Gm14416	20		ENSMUSG00000029030	Tprgl	20
ENSMUSG00000078867	Gm14418	20		ENSMUSG00000054226	Tprkb	1
ENSMUSG00000078875	Gm14419	20		ENSMUSG00000048707	Tprn	16
ENSMUSG00000083111	Gm14421	20		ENSMUSG00000034118	Tpst1	9
ENSMUSG00000081156	Gm14425	20		ENSMUSG00000029344	Tpst2	1
ENSMUSG00000081574	Gm14429	1		ENSMUSG00000060126	Tpt1	3

ENSMUSG00000078902	Gm14443	20		ENSMUSG00000084319	Tpt1-ps3	3
ENSMUSG00000078906	Gm14444	20		ENSMUSG00000059252	Tpt1-ps5	20
ENSMUSG00000080186	Gm14448	20		ENSMUSG00000084284	Tpt1-ps6	20
ENSMUSG00000087633	Gm14455	18		ENSMUSG00000027469	Tpx2	1
ENSMUSG00000081308	Gm14480	20		ENSMUSG00000029817	Tra2a	20
ENSMUSG00000085587	Gm14493	20		ENSMUSG00000022858	Tra2b	18
ENSMUSG00000083097	Gm14494	20		ENSMUSG00000015363	Trabd	18
ENSMUSG00000086931	Gm14532	20		ENSMUSG00000070867	Trabd2b	20
ENSMUSG00000082855	Gm14537	20		ENSMUSG00000076928	Trac	20
ENSMUSG00000084830	Gm14539	12		ENSMUSG00000031887	Tradd	20
ENSMUSG00000080896	Gm14567	20		ENSMUSG00000026875	Traf1	20
ENSMUSG00000091556	Gm14569	20		ENSMUSG00000026942	Traf2	20
ENSMUSG00000085624	Gm14573	20		ENSMUSG00000021277	Traf3	12
ENSMUSG00000083098	Gm14578	20		ENSMUSG00000034292	Traf3ip1	20
ENSMUSG00000082968	Gm14581	12		ENSMUSG00000019842	Traf3ip2	20
ENSMUSG00000083798	Gm14584	20		ENSMUSG00000037318	Traf3ip3	9
ENSMUSG00000083621	Gm14586	18		ENSMUSG00000017386	Traf4	9
ENSMUSG00000081620	Gm14633	20		ENSMUSG00000027164	Traf6	1
ENSMUSG00000085891	Gm14634	20		ENSMUSG00000052752	Traf7	20
ENSMUSG00000073274	Gm14636	18		ENSMUSG00000042726	Trafd1	20
ENSMUSG00000082253	Gm14639	20		ENSMUSG00000032586	Traip	9
ENSMUSG00000068014	Gm14648	20		ENSMUSG00000032536	Trak1	12
ENSMUSG00000083074	Gm14670	20		ENSMUSG00000026028	Trak2	20
ENSMUSG00000081603	Gm14681	20		ENSMUSG00000025935	Tram1	9
ENSMUSG00000071748	Gm14698	20		ENSMUSG00000044528	Tram1l1	20
ENSMUSG00000087644	Gm14703	20		ENSMUSG00000041779	Tram2	9
ENSMUSG00000082192	Gm14719	20		ENSMUSG00000062296	Trank1	9
ENSMUSG00000083877	Gm14740	20		ENSMUSG00000005981	Trap1	20
ENSMUSG00000081583	Gm14769	20		ENSMUSG00000049299	Trappc1	20
ENSMUSG00000081424	Gm14774	20		ENSMUSG00000000374	Trappc10	18
ENSMUSG00000083421	Gm14776	13		ENSMUSG00000038102	Trappc11	20
ENSMUSG00000083744	Gm14824	19		ENSMUSG00000020628	Trappc12	3
ENSMUSG00000084904	Gm14827	10		ENSMUSG00000021711	Trappc13	20
ENSMUSG00000083936	Gm14829	20		ENSMUSG00000079317	Trappc2	20
ENSMUSG00000081418	Gm14830	3		ENSMUSG00000015013	Trappc2l	19
ENSMUSG00000079965	Gm14853	20		ENSMUSG00000028847	Trappc3	10
ENSMUSG00000082812	Gm14860	20		ENSMUSG00000032112	Trappc4	12
ENSMUSG00000081978	Gm14895	1		ENSMUSG00000040236	Trappc5	20
ENSMUSG00000098559	Gm15013	20		ENSMUSG00000002043	Trappc6a	3
ENSMUSG00000086286	Gm15138	20		ENSMUSG00000020993	Trappc6b	1
ENSMUSG00000055109	Gm15155	20		ENSMUSG00000033382	Trappc8	3
ENSMUSG00000085711	Gm15163	20		ENSMUSG00000047921	Trappc9	20
ENSMUSG00000086188	Gm15169	20		ENSMUSG00000030775	Trat1	1
ENSMUSG00000083906	Gm15186	20		ENSMUSG00000026723	Trdmt1	20
ENSMUSG00000082778	Gm15191	20		ENSMUSG00000032554	Trf	16
ENSMUSG00000081873	Gm15197	18		ENSMUSG00000005892	Trh	16
ENSMUSG00000086034	Gm15201	20		ENSMUSG00000050663	Trhde	5
ENSMUSG00000082007	Gm15206	20		ENSMUSG00000038760	Trhr	9
ENSMUSG00000087159	Gm15246	20		ENSMUSG00000039079	Trhr2	20
ENSMUSG00000081355	Gm15264	6		ENSMUSG00000029535	Triap1	20
ENSMUSG00000086288	Gm15265	20		ENSMUSG00000032501	Trib1	12
ENSMUSG00000083474	Gm15267	20		ENSMUSG00000020601	Trib2	2
ENSMUSG00000087400	Gm15270	9		ENSMUSG00000032715	Trib3	20
ENSMUSG00000081277	Gm15285	20		ENSMUSG00000043496	Tril	18

ENSMUSG00000074449	Gm15319	20		ENSMUSG00000020455	Trim11	9
ENSMUSG00000086095	Gm15328	1		ENSMUSG00000066258	Trim12a	20
ENSMUSG00000085289	Gm15337	12		ENSMUSG00000057143	Trim12c	20
ENSMUSG00000085899	Gm15338	20		ENSMUSG00000035235	Trim13	20
ENSMUSG00000084899	Gm15344	20		ENSMUSG00000039853	Trim14	20
ENSMUSG00000086770	Gm15351	20		ENSMUSG00000047821	Trim16	20
ENSMUSG00000083283	Gm15361	20		ENSMUSG00000036964	Trim17	20
ENSMUSG00000082208	Gm15379	9		ENSMUSG00000027993	Trim2	10
ENSMUSG00000081771	Gm15381	20		ENSMUSG00000030966	Trim21	3
ENSMUSG00000084240	Gm15383	20		ENSMUSG00000021712	Trim23	20
ENSMUSG00000083093	Gm15385	1		ENSMUSG00000029833	Trim24	20
ENSMUSG00000082585	Gm15387	20		ENSMUSG00000000275	Trim25	20
ENSMUSG00000109491	Gm15396	3		ENSMUSG00000024457	Trim26	18
ENSMUSG00000085176	Gm15397	18		ENSMUSG00000021326	Trim27	20
ENSMUSG00000085971	Gm15411	20		ENSMUSG00000005566	Trim28	20
ENSMUSG00000074466	Gm15417	18		ENSMUSG00000036989	Trim3	19
ENSMUSG00000085573	Gm15418	18		ENSMUSG00000030921	Trim30a	20
ENSMUSG00000084235	Gm15421	9		ENSMUSG00000057596	Trim30d	18
ENSMUSG00000082738	Gm15422	9		ENSMUSG00000051675	Trim32	12
ENSMUSG00000081051	Gm15427	12		ENSMUSG00000033014	Trim33	4
ENSMUSG00000040323	Gm15429	13		ENSMUSG00000056144	Trim34a	20
ENSMUSG00000084370	Gm15442	20		ENSMUSG00000022043	Trim35	20
ENSMUSG00000082588	Gm15443	20		ENSMUSG00000033949	Trim36	16
ENSMUSG00000085311	Gm15445	20		ENSMUSG00000018548	Trim37	12
ENSMUSG00000090015	Gm15446	9		ENSMUSG00000045409	Trim39	20
ENSMUSG00000081402	Gm15455	20		ENSMUSG00000073399	Trim40	9
ENSMUSG00000100801	Gm15459	1		ENSMUSG00000040365	Trim41	9
ENSMUSG00000086189	Gm15462	2		ENSMUSG00000027189	Trim44	5
ENSMUSG00000082609	Gm15464	20		ENSMUSG00000033233	Trim45	20
ENSMUSG00000081657	Gm15466	12		ENSMUSG00000042766	Trim46	18
ENSMUSG00000087261	Gm15477	12		ENSMUSG00000020773	Trim47	20
ENSMUSG00000086145	Gm15479	20		ENSMUSG00000060441	Trim5	20
ENSMUSG00000081431	Gm15483	20		ENSMUSG00000043279	Trim56	20
ENSMUSG00000081865	Gm15484	20		ENSMUSG00000034317	Trim59	16
ENSMUSG00000085012	Gm15492	20		ENSMUSG00000072244	Trim6	20
ENSMUSG00000091177	Gm15494	18		ENSMUSG00000041000	Trim62	20
ENSMUSG00000085953	Gm15496	20		ENSMUSG00000054517	Trim65	20
ENSMUSG00000086583	Gm15500	12		ENSMUSG00000031026	Trim66	9
ENSMUSG00000087412	Gm15501	12		ENSMUSG00000036913	Trim67	18
ENSMUSG00000084964	Gm15503	20		ENSMUSG00000073968	Trim68	18
ENSMUSG00000086477	Gm15506	20		ENSMUSG00000040350	Trim7	6
ENSMUSG00000086604	Gm15510	20		ENSMUSG00000079259	Trim71	20
ENSMUSG00000086291	Gm15513	1		ENSMUSG00000025034	Trim8	19
ENSMUSG00000083443	Gm15519	10		ENSMUSG00000021071	Trim9	20
ENSMUSG00000085638	Gm15521	6		ENSMUSG00000022263	Trio	2
ENSMUSG00000080205	Gm15526	20		ENSMUSG00000033088	Triobp	20
ENSMUSG00000081816	Gm15528	1		ENSMUSG00000019487	Trip10	13
ENSMUSG00000081651	Gm15530	20		ENSMUSG00000021188	Trip11	3
ENSMUSG00000085527	Gm15535	20		ENSMUSG00000026219	Trip12	2
ENSMUSG00000085048	Gm15537	20		ENSMUSG00000032386	Trip4	20
ENSMUSG00000083579	Gm15538	20		ENSMUSG00000023348	Trip6	20
ENSMUSG00000081809	Gm15539	20		ENSMUSG00000055963	Triqk	13
ENSMUSG00000083396	Gm15542	20		ENSMUSG00000041203	Trir	20
ENSMUSG00000087138	Gm15545	20		ENSMUSG00000028653	Trit1	20

ENSMUSG00000082265	Gm15547	20		ENSMUSG00000028331	Trmo	20
ENSMUSG00000081745	Gm15549	20		ENSMUSG00000001909	Trmt1	3
ENSMUSG00000086401	Gm15559	9		ENSMUSG00000004127	Trmt10a	9
ENSMUSG00000089870	Gm15562	20		ENSMUSG00000035601	Trmt10b	20
ENSMUSG00000086324	Gm15564	9		ENSMUSG00000044763	Trmt10c	13
ENSMUSG00000083772	Gm15566	10		ENSMUSG00000019792	Trmt11	20
ENSMUSG00000081872	Gm15572	20		ENSMUSG00000038812	Trmt112	20
ENSMUSG00000083122	Gm15578	20		ENSMUSG00000070572	Trmt112-ps2	20
ENSMUSG00000080078	Gm15583	20		ENSMUSG00000037085	Trmt12	20
ENSMUSG00000086769	Gm15587	20		ENSMUSG00000033439	Trmt13	18
ENSMUSG00000058542	Gm15590	20		ENSMUSG00000053286	Trmt1l	20
ENSMUSG00000084203	Gm15593	20		ENSMUSG00000067369	Trmt2b	1
ENSMUSG00000087531	Gm15606	20		ENSMUSG00000029097	Trmt44	20
ENSMUSG00000086602	Gm15609	18		ENSMUSG00000034442	Trmt5	20
ENSMUSG00000078183	Gm15610	20		ENSMUSG00000037376	Trmt6	20
ENSMUSG00000085828	Gm15612	18		ENSMUSG00000060950	Trmt61a	20
ENSMUSG00000082705	Gm15616	20		ENSMUSG00000085492	Trmt61b	20
ENSMUSG00000085782	Gm15624	9		ENSMUSG00000039620	Trmt9b	1
ENSMUSG00000085067	Gm15631	20		ENSMUSG00000022386	Trmu	20
ENSMUSG00000086414	Gm15645	20		ENSMUSG00000028898	Trnau1ap	20
ENSMUSG00000085282	Gm15663	1		ENSMUSG00000056596	Trnp1	20
ENSMUSG00000083420	Gm15665	16		ENSMUSG00000013736	Trnt1	20
ENSMUSG00000084399	Gm15666	9		ENSMUSG00000025272	Tro	20
ENSMUSG00000115370	Gm15667	20		ENSMUSG00000059552	Trp53	20
ENSMUSG00000083795	Gm15670	20		ENSMUSG00000090704	Trp53-ps	18
ENSMUSG00000086873	Gm15672	20		ENSMUSG00000043909	Trp53bp1	19
ENSMUSG00000086825	Gm15675	20		ENSMUSG00000026510	Trp53bp2	20
ENSMUSG00000090116	Gm15680	20		ENSMUSG00000085912	Trp53cor1	20
ENSMUSG00000086842	Gm15681	20		ENSMUSG00000068735	Trp53i11	1
ENSMUSG00000095524	Gm15682	12		ENSMUSG00000044328	Trp53i13	20
ENSMUSG00000089737	Gm15688	20		ENSMUSG00000028211	Trp53inp1	20
ENSMUSG00000083853	Gm15696	20		ENSMUSG00000038375	Trp53inp2	16
ENSMUSG00000081670	Gm15697	20		ENSMUSG00000039725	Trp53rka	20
ENSMUSG00000085658	Gm15704	20		ENSMUSG00000042854	Trp53rkb	20
ENSMUSG00000086013	Gm15706	20		ENSMUSG00000022510	Trp63	20
ENSMUSG00000086894	Gm15708	20		ENSMUSG00000029026	Trp73	14
ENSMUSG00000084111	Gm15710	12		ENSMUSG00000032839	Trpc1	7
ENSMUSG00000082570	Gm15711	11		ENSMUSG00000027716	Trpc3	18
ENSMUSG00000083827	Gm15712	20		ENSMUSG00000027748	Trpc4	5
ENSMUSG00000082120	Gm15720	20		ENSMUSG00000038324	Trpc4ap	20
ENSMUSG00000085565	Gm15721	9		ENSMUSG00000041710	Trpc5	20
ENSMUSG00000084865	Gm15723	20		ENSMUSG00000031997	Trpc6	9
ENSMUSG00000086075	Gm15728	20		ENSMUSG00000021541	Trpc7	18
ENSMUSG00000086115	Gm15733	20		ENSMUSG00000009292	Trpm2	9
ENSMUSG00000087526	Gm15738	20		ENSMUSG00000052387	Trpm3	13
ENSMUSG00000080957	Gm15739	20		ENSMUSG00000038260	Trpm4	20
ENSMUSG00000090027	Gm15740	20		ENSMUSG00000024727	Trpm6	18
ENSMUSG00000087605	Gm15742	20		ENSMUSG00000027365	Trpm7	2
ENSMUSG00000086645	Gm15743	20		ENSMUSG00000038679	Trps1	12
ENSMUSG00000082088	Gm15753	9		ENSMUSG00000047656	Trpt1	20
ENSMUSG00000082308	Gm15770	9		ENSMUSG00000018507	Trpv2	1
ENSMUSG00000062353	Gm15772	12		ENSMUSG00000043029	Trpv3	20
ENSMUSG00000082872	Gm15773	20		ENSMUSG00000014158	Trpv4	13
ENSMUSG00000081126	Gm15784	20		ENSMUSG00000029868	Trpv6	9

ENSMUSG00000086247	Gm15787	20		ENSMUSG00000045482	Trrap	9
ENSMUSG00000082057	Gm15789	20		ENSMUSG00000025086	Trub1	20
ENSMUSG00000089648	Gm15790	12		ENSMUSG00000039826	Trub2	1
ENSMUSG00000082769	Gm15793	20		ENSMUSG00000010538	Tsacc	20
ENSMUSG00000082130	Gm15799	20		ENSMUSG00000026812	Tsc1	9
ENSMUSG00000086175	Gm15802	20		ENSMUSG00000002496	Tsc2	20
ENSMUSG00000083536	Gm15808	20		ENSMUSG00000022010	Tsc22d1	18
ENSMUSG00000090135	Gm15809	7		ENSMUSG00000027806	Tsc22d2	3
ENSMUSG00000089710	Gm15810	7		ENSMUSG00000031431	Tsc22d3	20
ENSMUSG00000083285	Gm15815	20		ENSMUSG00000029723	Tsc22d4	3
ENSMUSG00000082823	Gm15828	20		ENSMUSG00000014980	Tsen15	20
ENSMUSG00000085894	Gm15832	20		ENSMUSG00000042389	Tsen2	13
ENSMUSG00000085054	Gm15834	20		ENSMUSG00000035585	Tsen34	20
ENSMUSG00000083909	Gm15842	20		ENSMUSG00000020781	Tsen54	20
ENSMUSG00000090079	Gm15849	20		ENSMUSG00000040521	Tsfm	20
ENSMUSG00000086264	Gm15850	7		ENSMUSG00000014402	Tsg101	9
ENSMUSG00000103400	Gm15853	20		ENSMUSG00000106988	Tsg101-ps	20
ENSMUSG00000086944	Gm15859	20		ENSMUSG00000060771	Tsga10	20
ENSMUSG00000087336	Gm15860	20		ENSMUSG00000020963	Tshr	20
ENSMUSG00000089812	Gm15867	20		ENSMUSG00000046982	Tshz1	20
ENSMUSG00000084790	Gm15879	20		ENSMUSG00000047907	Tshz2	18
ENSMUSG00000085254	Gm15881	20		ENSMUSG00000021217	Tshz3	19
ENSMUSG00000085754	Gm15886	20		ENSMUSG00000085715	Tsix	19
ENSMUSG00000085068	Gm15895	6		ENSMUSG00000059891	Tsks	20
ENSMUSG00000081273	Gm15896	20		ENSMUSG00000049580	Tsku	20
ENSMUSG00000085917	Gm15899	20		ENSMUSG00000026374	Tsn	20
ENSMUSG00000086012	Gm15902	3		ENSMUSG00000056820	Tsnax	18
ENSMUSG00000086916	Gm15903	20		ENSMUSG00000031893	Tsnaxip1	13
ENSMUSG00000085881	Gm15912	20		ENSMUSG00000030351	Tspan11	18
ENSMUSG00000087652	Gm15918	20		ENSMUSG00000029669	Tspan12	18
ENSMUSG00000080893	Gm15920	20		ENSMUSG00000020577	Tspan13	5
ENSMUSG00000085039	Gm15927	20		ENSMUSG00000037824	Tspan14	18
ENSMUSG00000084024	Gm15937	20		ENSMUSG00000037031	Tspan15	9
ENSMUSG00000086313	Gm15940	12		ENSMUSG00000025875	Tspan17	18
ENSMUSG00000086992	Gm15941	20		ENSMUSG00000027217	Tspan18	5
ENSMUSG00000085655	Gm15952	20		ENSMUSG00000027858	Tspan2	16
ENSMUSG00000086935	Gm15956	20		ENSMUSG00000086745	Tspan2os	9
ENSMUSG00000081692	Gm15971	20		ENSMUSG00000032324	Tspan3	12
ENSMUSG00000083902	Gm15975	20		ENSMUSG00000006736	Tspan31	20
ENSMUSG00000087016	Gm15979	20		ENSMUSG00000052281	Tspan32os	20
ENSMUSG00000080700	Gm15981	20		ENSMUSG00000001763	Tspan33	18
ENSMUSG00000084132	Gm15982	20		ENSMUSG00000025511	Tspan4	20
ENSMUSG00000081104	Gm15989	20		ENSMUSG00000028152	Tspan5	20
ENSMUSG00000087266	Gm15991	1		ENSMUSG00000067377	Tspan6	9
ENSMUSG00000107132	Gm15997	20		ENSMUSG00000058254	Tspan7	9
ENSMUSG00000086389	Gm15998	20		ENSMUSG00000030352	Tspan9	18
ENSMUSG00000085433	Gm16001	20		ENSMUSG00000069581	Tspear	20
ENSMUSG00000087381	Gm16008	6		ENSMUSG00000041736	Tspo	20
ENSMUSG00000081303	Gm16011	20		ENSMUSG00000034156	Tspoap1	20
ENSMUSG00000072576	Gm16020	20		ENSMUSG00000047514	Tspyl1	20
ENSMUSG00000086682	Gm16023	20		ENSMUSG00000041096	Tspyl2	20
ENSMUSG00000107724	Gm16042	20		ENSMUSG00000074671	Tspyl3	1
ENSMUSG00000081339	Gm16044	20		ENSMUSG00000039485	Tspyl4	18
ENSMUSG00000085705	Gm16046	20		ENSMUSG00000038984	Tspyl5	20

ENSMUSG00000116760	Gm1604a	9		ENSMUSG00000038335	Tsr1	20
ENSMUSG00000094083	Gm1604b	18		ENSMUSG00000025264	Tsr2	16
ENSMUSG00000083974	Gm16053	20		ENSMUSG00000015126	Tsr3	20
ENSMUSG00000087431	Gm16054	20		ENSMUSG00000045752	Tssc4	20
ENSMUSG00000087249	Gm16062	20		ENSMUSG00000007591	Tssk4	20
ENSMUSG00000085125	Gm16070	20		ENSMUSG00000044986	Tst	20
ENSMUSG00000082051	Gm16072	20		ENSMUSG00000022570	Tsta3	20
ENSMUSG00000082247	Gm16074	20		ENSMUSG00000035495	Tstd2	20
ENSMUSG00000087322	Gm16075	1		ENSMUSG00000028251	Tstd3	10
ENSMUSG00000087514	Gm16076	20		ENSMUSG00000015599	Ttbk1	20
ENSMUSG00000082967	Gm16082	18		ENSMUSG00000090100	Ttbk2	3
ENSMUSG00000085526	Gm16083	20		ENSMUSG00000041278	Ttc1	20
ENSMUSG00000081228	Gm16089	10		ENSMUSG00000040219	Ttc12	20
ENSMUSG00000089910	Gm16113	20		ENSMUSG00000037300	Ttc13	19
ENSMUSG00000086914	Gm16124	20		ENSMUSG00000027677	Ttc14	20
ENSMUSG00000080971	Gm16128	1		ENSMUSG00000039021	Ttc16	20
ENSMUSG00000084985	Gm16135	20		ENSMUSG00000027194	Ttc17	19
ENSMUSG00000085687	Gm16153	20		ENSMUSG00000042298	Ttc19	20
ENSMUSG00000090118	Gm16163	20		ENSMUSG00000032514	Ttc21a	14
ENSMUSG00000089941	Gm16168	20		ENSMUSG00000034848	Ttc21b	20
ENSMUSG00000082484	Gm16177	20		ENSMUSG00000034919	Ttc22	20
ENSMUSG00000086067	Gm16183	20		ENSMUSG00000030555	Ttc23	20
ENSMUSG00000081520	Gm16200	20		ENSMUSG00000006784	Ttc25	13
ENSMUSG00000086742	Gm16201	20		ENSMUSG00000056832	Ttc26	20
ENSMUSG00000081316	Gm16205	20		ENSMUSG00000024078	Ttc27	1
ENSMUSG00000090006	Gm16227	20		ENSMUSG00000033209	Ttc28	9
ENSMUSG00000089627	Gm16228	20		ENSMUSG00000040785	Ttc3	20
ENSMUSG00000090040	Gm16232	20		ENSMUSG00000075271	Ttc30a1	9
ENSMUSG00000085773	Gm16233	16		ENSMUSG00000075273	Ttc30b	20
ENSMUSG00000089988	Gm16238	20		ENSMUSG00000066637	Ttc32	20
ENSMUSG00000089711	Gm16240	20		ENSMUSG00000022151	Ttc33	20
ENSMUSG00000090235	Gm16244	20		ENSMUSG00000046637	Ttc34	20
ENSMUSG00000087610	Gm16253	20		ENSMUSG00000033991	Ttc37	1
ENSMUSG00000089959	Gm16268	10		ENSMUSG00000035944	Ttc38	20
ENSMUSG00000084862	Gm16278	20		ENSMUSG00000028555	Ttc39a	6
ENSMUSG00000084998	Gm16279	20		ENSMUSG00000085873	Ttc39aos1	6
ENSMUSG00000086235	Gm16284	20		ENSMUSG00000038172	Ttc39b	12
ENSMUSG00000024571	Gm16286	12		ENSMUSG00000024424	Ttc39c	5
ENSMUSG00000073739	Gm16287	20		ENSMUSG00000025413	Ttc4	2
ENSMUSG00000085099	Gm16288	20		ENSMUSG00000044937	Ttc41	20
ENSMUSG00000089679	Gm16299	20		ENSMUSG00000006288	Ttc5	20
ENSMUSG00000089828	Gm16300	1		ENSMUSG00000046782	Ttc6	9
ENSMUSG00000086331	Gm16310	20		ENSMUSG00000036918	Ttc7	9
ENSMUSG00000087435	Gm16323	17		ENSMUSG00000033530	Ttc7b	18
ENSMUSG00000090050	Gm16339	3		ENSMUSG00000021013	Ttc8	18
ENSMUSG00000115205	Gm16374	20		ENSMUSG00000042734	Ttc9	20
ENSMUSG00000095762	Gm16378	6		ENSMUSG00000007944	Ttc9b	20
ENSMUSG00000059658	Gm16379	20		ENSMUSG00000071660	Ttc9c	12
ENSMUSG00000116811	Gm16385	20		ENSMUSG00000026803	Ttf1	20
ENSMUSG00000117333	Gm16386	20		ENSMUSG00000033222	Ttf2	9
ENSMUSG00000075581	Gm16409	18		ENSMUSG00000027650	Tti1	20
ENSMUSG00000084093	Gm16418	12		ENSMUSG00000031577	Tti2	20
ENSMUSG00000043024	Gm16433	20		ENSMUSG00000027394	Ttl	9
ENSMUSG00000024761	Gm16437	12		ENSMUSG00000022442	Ttll1	7

ENSMUSG00000072594	Gm16439	1		ENSMUSG00000026885	Ttll11	9
ENSMUSG00000072738	Gm16440	20		ENSMUSG00000016757	Ttll12	20
ENSMUSG00000082154	Gm16464	20		ENSMUSG00000045467	Ttll13	9
ENSMUSG00000098091	Gm16470	20		ENSMUSG00000030276	Ttll3	1
ENSMUSG00000062758	Gm16477	20		ENSMUSG00000033257	Ttll4	3
ENSMUSG00000078117	Gm16485	20		ENSMUSG00000012609	Ttll5	18
ENSMUSG00000108175	Gm16499	20		ENSMUSG00000038756	Ttll6	14
ENSMUSG00000108255	Gm16499	20		ENSMUSG00000036745	Ttll7	18
ENSMUSG00000083015	Gm16518	9		ENSMUSG00000022388	Ttll8	1
ENSMUSG00000082315	Gm16523	20		ENSMUSG00000074673	Ttll9	12
ENSMUSG00000089810	Gm16536	1		ENSMUSG00000051747	Ttn	19
ENSMUSG00000089671	Gm16537	20		ENSMUSG00000073988	Ttpa	20
ENSMUSG00000089673	Gm16546	20		ENSMUSG00000017679	Ttpal	20
ENSMUSG00000066477	Gm16551	1		ENSMUSG00000061808	Ttr	13
ENSMUSG00000089908	Gm16558	12		ENSMUSG00000030428	Ttyh1	1
ENSMUSG00000089806	Gm16570	20		ENSMUSG00000034714	Ttyh2	16
ENSMUSG00000087543	Gm16576	20		ENSMUSG00000036565	Ttyh3	1
ENSMUSG00000089764	Gm16580	10		ENSMUSG00000031028	Tub	5
ENSMUSG00000089670	Gm16581	20		ENSMUSG00000117974	Tuba-rs1	20
ENSMUSG00000074482	Gm16589	12		ENSMUSG00000072235	Tuba1a	20
ENSMUSG00000097788	Gm16596	20		ENSMUSG00000023004	Tuba1b	9
ENSMUSG00000097434	Gm16630	20		ENSMUSG00000043091	Tuba1c	1
ENSMUSG00000087088	Gm16638	20		ENSMUSG00000026202	Tuba4a	20
ENSMUSG00000097534	Gm16675	20		ENSMUSG00000030137	Tuba8	20
ENSMUSG00000102548	Gm16701	20		ENSMUSG00000058672	Tubb2a	9
ENSMUSG00000097823	Gm16701	20		ENSMUSG00000113275	Tubb2a-ps2	20
ENSMUSG00000097077	Gm16712	20		ENSMUSG00000045136	Tubb2b	20
ENSMUSG00000070858	Gm1673	12		ENSMUSG00000062380	Tubb3	18
ENSMUSG00000097167	Gm16740	20		ENSMUSG00000062591	Tubb4a	16
ENSMUSG00000085303	Gm16751	20		ENSMUSG00000036752	Tubb4b	9
ENSMUSG00000097439	Gm16754	20		ENSMUSG00000095159	Tubb4b-ps1	20
ENSMUSG00000086534	Gm16758	20		ENSMUSG00000001525	Tubb5	9
ENSMUSG00000086539	Gm16759	20		ENSMUSG00000001473	Tubb6	19
ENSMUSG00000097357	Gm16793	20		ENSMUSG00000020513	Tubd1	20
ENSMUSG00000097777	Gm16794	7		ENSMUSG00000019845	Tube1	20
ENSMUSG00000085495	Gm16796	20		ENSMUSG00000035198	Tubg1	20
ENSMUSG00000087479	Gm16835	16		ENSMUSG00000045007	Tubg2	20
ENSMUSG00000096981	Gm16845	20		ENSMUSG00000025474	Tubgcp2	9
ENSMUSG00000097397	Gm16861	20		ENSMUSG00000000759	Tubgcp3	20
ENSMUSG00000093954	Gm16867	20		ENSMUSG00000027263	Tubgcp4	3
ENSMUSG00000097876	Gm16892	9		ENSMUSG00000033790	Tubgcp5	20
ENSMUSG00000097899	Gm16894	18		ENSMUSG00000051786	Tubgcp6	18
ENSMUSG00000097325	Gm16897	20		ENSMUSG00000073838	Tufm	20
ENSMUSG00000097456	Gm16958	12		ENSMUSG00000005968	Tuft1	9
ENSMUSG00000097743	Gm16973	20		ENSMUSG00000056579	Tug1	20
ENSMUSG00000074404	Gm1698	20		ENSMUSG00000001521	Tulp3	20
ENSMUSG00000097069	Gm16998	9		ENSMUSG00000034377	Tulp4	20
ENSMUSG00000041035	Gm17018	2		ENSMUSG00000097929	Tunar	18
ENSMUSG00000090582	Gm17024	20		ENSMUSG00000054000	Tusc1	20
ENSMUSG00000091165	Gm17036	20		ENSMUSG00000010054	Tusc2	9
ENSMUSG00000091509	Gm17066	20		ENSMUSG00000039530	Tusc3	9
ENSMUSG00000078528	Gm17068	20		ENSMUSG00000071645	Tut1	1
ENSMUSG00000091288	Gm17075	20		ENSMUSG00000034610	Tut4	20
ENSMUSG00000090449	Gm17076	20		ENSMUSG00000035248	Tut7	20

ENSMUSG00000090674	Gm17082	20		ENSMUSG00000050908	Tvp23a	20
ENSMUSG00000099104	Gm17087	20		ENSMUSG00000014177	Tvp23b	20
ENSMUSG00000091817	Gm17091	1		ENSMUSG00000086677	Tvp23bos	20
ENSMUSG00000091623	Gm17092	20		ENSMUSG00000022451	Twf1	3
ENSMUSG00000090397	Gm17096	20		ENSMUSG00000023277	Twf2	20
ENSMUSG00000091305	Gm17100	9		ENSMUSG00000035799	Twist1	1
ENSMUSG00000091665	Gm17101	9		ENSMUSG00000020561	Twistnb	20
ENSMUSG00000091864	Gm17102	20		ENSMUSG00000025209	Twnk	20
ENSMUSG00000091079	Gm17105	20		ENSMUSG00000024098	Twsg1	20
ENSMUSG00000090873	Gm17112	9		ENSMUSG00000053841	Txlna	20
ENSMUSG00000091968	Gm17115	20		ENSMUSG00000039891	Txlnb	1
ENSMUSG00000090785	Gm17116	20		ENSMUSG00000038344	Txlng	16
ENSMUSG00000091881	Gm17146	18		ENSMUSG00000028367	Txn1	5
ENSMUSG00000090939	Gm17147	20		ENSMUSG00000005354	Txn2	3
ENSMUSG00000090758	Gm17178	20		ENSMUSG00000022498	Txndc11	20
ENSMUSG00000090589	Gm17180	20		ENSMUSG00000028567	Txndc12	2
ENSMUSG00000099115	Gm17190	20		ENSMUSG00000021497	Txndc15	12
ENSMUSG00000091587	Gm17191	20		ENSMUSG00000021830	Txndc16	9
ENSMUSG00000079645	Gm17193	20		ENSMUSG00000020803	Txndc17	20
ENSMUSG00000091534	Gm17195	20		ENSMUSG00000050612	Txndc2	20
ENSMUSG00000090965	Gm17203	11		ENSMUSG00000038991	Txndc5	20
ENSMUSG00000090561	Gm17207	10		ENSMUSG00000058407	Txndc9	20
ENSMUSG00000092085	Gm17224	20		ENSMUSG00000038393	Txnip	14
ENSMUSG00000091426	Gm17228	7		ENSMUSG00000024583	Txnl1	20
ENSMUSG00000092051	Gm17229	20		ENSMUSG00000057130	Txnl4a	20
ENSMUSG00000090706	Gm17233	20		ENSMUSG00000031723	Txnl4b	20
ENSMUSG00000115938	Gm17241	12		ENSMUSG00000020250	Txnrd1	20
ENSMUSG00000102997	Gm17244	14		ENSMUSG00000000811	Txnrd3	19
ENSMUSG00000097309	Gm17249	20		ENSMUSG00000032175	Tyk2	20
ENSMUSG00000090952	Gm17251	3		ENSMUSG00000022615	Tymp	20
ENSMUSG00000094108	Gm17258	20		ENSMUSG00000025747	Tyms	20
ENSMUSG00000097793	Gm17259	20		ENSMUSG00000027298	Tyro3	3
ENSMUSG00000097305	Gm17276	1		ENSMUSG00000030579	Tyrobp	19
ENSMUSG00000097604	Gm17322	20		ENSMUSG00000020087	Tysnd1	20
ENSMUSG00000090317	Gm17324	20		ENSMUSG00000056310	Tyw1	20
ENSMUSG00000078143	Gm17344	20		ENSMUSG00000047583	Tyw3	20
ENSMUSG00000091604	Gm17349	20		ENSMUSG00000048495	Tyw5	9
ENSMUSG00000091985	Gm17354	20		ENSMUSG00000061613	U2af1	20
ENSMUSG00000078838	Gm17382	9		ENSMUSG00000078765	U2af1l4	20
ENSMUSG00000058625	Gm17383	20		ENSMUSG00000030435	U2af2	20
ENSMUSG00000079669	Gm17396	20		ENSMUSG00000032407	U2surp	20
ENSMUSG00000097000	Gm17435	20		ENSMUSG00000034485	Uaca	20
ENSMUSG00000092004	Gm17482	20		ENSMUSG00000026670	Uap1	19
ENSMUSG00000091514	Gm17484	18		ENSMUSG00000026956	Uap1l1	20
ENSMUSG00000097042	Gm17491	20		ENSMUSG00000001924	Uba1	1
ENSMUSG00000057359	Gm17494	9		ENSMUSG00000052997	Uba2	20
ENSMUSG00000097183	Gm17501	20		ENSMUSG00000030061	Uba3	9
ENSMUSG00000091423	Gm17509	9		ENSMUSG00000032557	Uba5	20
ENSMUSG00000090460	Gm17511	18		ENSMUSG00000090137	Uba52	12
ENSMUSG00000103731	Gm17530	12		ENSMUSG00000061390	Uba52-ps	1
ENSMUSG00000091058	Gm17538	18		ENSMUSG00000035898	Uba6	20
ENSMUSG00000097613	Gm17597	9		ENSMUSG00000032596	Uba7	20
ENSMUSG00000097482	Gm17634	18		ENSMUSG00000036352	Ubac1	20
ENSMUSG00000097057	Gm17638	20		ENSMUSG00000041765	Ubac2	20

ENSMUSG00000090963	Gm17655	20		ENSMUSG00000039568	Ubald1	9
ENSMUSG00000091034	Gm17660	1		ENSMUSG00000050628	Ubald2	12
ENSMUSG00000091695	Gm17669	20		ENSMUSG00000028437	Ubap1	20
ENSMUSG00000097204	Gm17690	20		ENSMUSG00000086228	Ubap1l	6
ENSMUSG00000090936	Gm17705	20		ENSMUSG00000028433	Ubap2	20
ENSMUSG00000091952	Gm17709	20		ENSMUSG00000042520	Ubap2l	20
ENSMUSG00000097486	Gm17733	20		ENSMUSG00000032020	Ubash3b	15
ENSMUSG00000098087	Gm17750	20		ENSMUSG00000019505	Ubb	12
ENSMUSG00000115867	Gm17753	9		ENSMUSG00000008348	Ubc	20
ENSMUSG00000097987	Gm17786	18		ENSMUSG00000016308	Ube2a	20
ENSMUSG00000112727	Gm17794	20		ENSMUSG00000020390	Ube2b	9
ENSMUSG00000118093	Gm17802	9		ENSMUSG00000032415	Ube2cbp	1
ENSMUSG00000101814	Gm17807	20		ENSMUSG00000083844	Ube2d-ps	20
ENSMUSG00000116919	Gm17809	20		ENSMUSG00000019927	Ube2d1	20
ENSMUSG00000112239	Gm17823	20		ENSMUSG00000091896	Ube2d2a	3
ENSMUSG00000106484	Gm17833	20		ENSMUSG00000078578	Ube2d3	20
ENSMUSG00000112642	Gm17849	20		ENSMUSG00000021774	Ube2e1	20
ENSMUSG00000111092	Gm17875	19		ENSMUSG00000058317	Ube2e2	20
ENSMUSG00000107491	Gm17893	20		ENSMUSG00000027011	Ube2e3	20
ENSMUSG00000108833	Gm17909	12		ENSMUSG00000034343	Ube2f	20
ENSMUSG00000100515	Gm17916	20		ENSMUSG00000020794	Ube2g1	20
ENSMUSG00000115632	Gm17922	20		ENSMUSG00000009293	Ube2g2	1
ENSMUSG00000109384	Gm17931	3		ENSMUSG00000039159	Ube2h	20
ENSMUSG00000106664	Gm17936	20		ENSMUSG00000015120	Ube2i	12
ENSMUSG00000101122	Gm17971	20		ENSMUSG00000028277	Ube2j1	9
ENSMUSG00000104564	Gm17977	20		ENSMUSG00000023286	Ube2j2	20
ENSMUSG00000101135	Gm17981	20		ENSMUSG00000029203	Ube2k	3
ENSMUSG00000108647	Gm17984	20		ENSMUSG00000038965	Ube2l3	20
ENSMUSG00000110422	Gm17999	20		ENSMUSG00000027078	Ube2l6	20
ENSMUSG00000112779	Gm18029	20		ENSMUSG00000005575	Ube2m	12
ENSMUSG00000113192	Gm18032	10		ENSMUSG00000074781	Ube2n	20
ENSMUSG00000113028	Gm18065	20		ENSMUSG00000106831	Ube2n-ps1	20
ENSMUSG00000117182	Gm18068	9		ENSMUSG00000059585	Ube2nl	20
ENSMUSG00000115856	Gm18095	20		ENSMUSG00000020802	Ube2o	9
ENSMUSG00000110726	Gm18101	3		ENSMUSG00000042572	Ube2q1	1
ENSMUSG00000112918	Gm18120	5		ENSMUSG00000032307	Ube2q2	20
ENSMUSG00000114340	Gm18132	20		ENSMUSG00000052981	Ube2ql1	19
ENSMUSG00000103601	Gm18150	20		ENSMUSG00000036241	Ube2r2	20
ENSMUSG00000091277	Gm1818	20		ENSMUSG00000060860	Ube2s	20
ENSMUSG00000099342	Gm18180	20		ENSMUSG00000026429	Ube2t	20
ENSMUSG00000112906	Gm18189	20		ENSMUSG00000078923	Ube2v1	13
ENSMUSG00000098900	Gm18190	20		ENSMUSG00000022674	Ube2v2	20
ENSMUSG00000109724	Gm18194	20		ENSMUSG00000025939	Ube2w	1
ENSMUSG00000044285	Gm1821	12		ENSMUSG00000014349	Ube2z	9
ENSMUSG00000109600	Gm18212	20		ENSMUSG00000025326	Ube3a	2
ENSMUSG00000111808	Gm18225	20		ENSMUSG00000029577	Ube3b	20
ENSMUSG00000082028	Gm1826	20		ENSMUSG00000039000	Ube3c	2
ENSMUSG00000092325	Gm18284	20		ENSMUSG00000059890	Ube4a	10
ENSMUSG00000114653	Gm18398	3		ENSMUSG00000028960	Ube4b	9
ENSMUSG00000043192	Gm1840	20		ENSMUSG00000030870	Ubfd1	20
ENSMUSG00000101054	Gm18406	5		ENSMUSG00000047719	Ubiad1	20
ENSMUSG00000083892	Gm1848	1		ENSMUSG00000001687	Ubl3	9
ENSMUSG00000104264	Gm18529	9		ENSMUSG00000015290	Ubl4a	20
ENSMUSG00000108484	Gm18537	20		ENSMUSG00000084786	Ubl5	12

ENSMUSG00000110768	Gm18541	20		ENSMUSG00000055720	Ubl7	20
ENSMUSG00000110123	Gm18562	20		ENSMUSG00000041231	Ublcp1	20
ENSMUSG00000093402	Gm18588	20		ENSMUSG00000039473	Ubn1	1
ENSMUSG00000108964	Gm18600	20		ENSMUSG00000038538	Ubn2	8
ENSMUSG00000110446	Gm18646	20		ENSMUSG00000027300	Ubox5	9
ENSMUSG00000082070	Gm1866	20		ENSMUSG00000009741	Ubp1	1
ENSMUSG00000099809	Gm18665	19		ENSMUSG00000005312	Ubqln1	18
ENSMUSG00000112511	Gm18704	20		ENSMUSG00000050148	Ubqln2	7
ENSMUSG00000110847	Gm18705	18		ENSMUSG00000008604	Ubqln4	9
ENSMUSG00000109882	Gm18706	2		ENSMUSG00000027272	Ubr1	2
ENSMUSG00000116348	Gm18722	20		ENSMUSG00000023977	Ubr2	20
ENSMUSG00000112218	Gm18726	20		ENSMUSG00000044308	Ubr3	3
ENSMUSG00000117300	Gm18736	11		ENSMUSG00000066036	Ubr4	3
ENSMUSG00000112712	Gm18755	20		ENSMUSG00000037487	Ubr5	11
ENSMUSG00000114705	Gm18760	20		ENSMUSG00000041712	Ubr7	20
ENSMUSG00000099473	Gm18775	20		ENSMUSG00000025171	Ubtd1	20
ENSMUSG00000110569	Gm18860	20		ENSMUSG00000044949	Ubtd2	20
ENSMUSG00000101994	Gm18875	20		ENSMUSG00000020923	Ubtf	9
ENSMUSG00000114418	Gm18883	20		ENSMUSG00000071655	Ubxn1	3
ENSMUSG00000116478	Gm18890	20		ENSMUSG00000043621	Ubxn10	20
ENSMUSG00000107596	Gm18913	20		ENSMUSG00000012126	Ubxn11	3
ENSMUSG00000114147	Gm18939	10		ENSMUSG00000020634	Ubxn2a	18
ENSMUSG00000109472	Gm18943	20		ENSMUSG00000028243	Ubxn2b	9
ENSMUSG00000115808	Gm18949	20		ENSMUSG00000026353	Ubxn4	1
ENSMUSG00000113840	Gm18955	20		ENSMUSG00000019578	Ubxn6	20
ENSMUSG00000100867	Gm18981	20		ENSMUSG00000053774	Ubxn7	20
ENSMUSG00000114622	Gm18990	20		ENSMUSG00000052906	Ubxn8	20
ENSMUSG00000110394	Gm18991	3		ENSMUSG00000029223	Uchl1	9
ENSMUSG00000115040	Gm18994	20		ENSMUSG00000022111	Uchl3	20
ENSMUSG00000103845	Gm19026	2		ENSMUSG00000035337	Uchl4	20
ENSMUSG00000100662	Gm19029	20		ENSMUSG00000018189	Uchl5	20
ENSMUSG00000102232	Gm19035	20		ENSMUSG00000002550	Uck1	20
ENSMUSG00000107411	Gm19040	20		ENSMUSG00000026558	Uck2	20
ENSMUSG00000113511	Gm19046	20		ENSMUSG00000089917	Uckl1	20
ENSMUSG00000111945	Gm19169	20		ENSMUSG00000010492	Uckl1os	20
ENSMUSG00000113357	Gm19195	20		ENSMUSG00000026668	Ucma	20
ENSMUSG00000109975	Gm19196	20		ENSMUSG00000033685	Ucp2	13
ENSMUSG00000113491	Gm19221	20		ENSMUSG00000032942	Ucp3	20
ENSMUSG00000115238	Gm19236	20		ENSMUSG00000043262	Uevld	5
ENSMUSG00000107230	Gm19265	20		ENSMUSG00000062963	Ufc1	20
ENSMUSG00000110238	Gm19269	20		ENSMUSG00000005262	Ufd1	19
ENSMUSG00000111353	Gm19324	20		ENSMUSG00000040359	Ufl1	20
ENSMUSG00000101365	Gm19325	20		ENSMUSG00000027746	Ufm1	20
ENSMUSG00000112789	Gm19326	1		ENSMUSG00000051502	Ufsp1	20
ENSMUSG00000113776	Gm19327	20		ENSMUSG00000031634	Ufsp2	1
ENSMUSG00000103307	Gm19335	20		ENSMUSG00000028381	Ugcg	20
ENSMUSG00000112411	Gm19337	20		ENSMUSG00000029201	Ugdh	20
ENSMUSG00000092216	Gm19345	20		ENSMUSG00000037470	Uggt1	20
ENSMUSG00000098492	Gm19353	20		ENSMUSG00000042104	Uggt2	20
ENSMUSG00000105139	Gm19391	20		ENSMUSG00000001891	Ugp2	9
ENSMUSG00000106229	Gm19409	1		ENSMUSG00000090124	Ugt1a7c	20
ENSMUSG00000109372	Gm19410	9		ENSMUSG00000032854	Ugt8a	16
ENSMUSG00000101438	Gm19412	20		ENSMUSG00000026667	Uhmk1	18
ENSMUSG00000099310	Gm19427	20		ENSMUSG00000001228	Uhrf1	20

ENSMUSG00000110528	Gm1943	20		ENSMUSG00000039512	Uhrf1bp1	18
ENSMUSG00000105843	Gm19439	2		ENSMUSG00000019951	Uhrf1bp1l	3
ENSMUSG00000102498	Gm19445	16		ENSMUSG00000024817	Uhrf2	20
ENSMUSG00000101693	Gm19461	12		ENSMUSG00000025878	Uimc1	20
ENSMUSG00000117864	Gm19500	20		ENSMUSG00000029512	Ulk1	3
ENSMUSG00000108478	Gm19514	20		ENSMUSG00000004798	Ulk2	1
ENSMUSG00000116903	Gm19522	20		ENSMUSG00000032308	Ulk3	20
ENSMUSG00000111348	Gm19531	3		ENSMUSG00000040936	Ulk4	18
ENSMUSG00000104493	Gm19552	20		ENSMUSG00000089862	Umad1	20
ENSMUSG00000099094	Gm19569	20		ENSMUSG00000022814	Umps	20
ENSMUSG00000100701	Gm19587	20		ENSMUSG00000002058	Unc119	9
ENSMUSG00000113585	Gm19605	20		ENSMUSG00000046562	Unc119b	20
ENSMUSG00000107198	Gm19619	19		ENSMUSG00000034799	Unc13a	18
ENSMUSG00000073902	Gm1966	20		ENSMUSG00000028456	Unc13b	1
ENSMUSG00000105766	Gm19666	20		ENSMUSG00000062151	Unc13c	16
ENSMUSG00000070960	Gm19680	1		ENSMUSG00000057948	Unc13d	20
ENSMUSG00000097113	Gm19705	20		ENSMUSG00000030533	Unc45a	20
ENSMUSG00000114593	Gm19716	20		ENSMUSG00000026111	Unc50	20
ENSMUSG00000102324	Gm19721	9		ENSMUSG00000025876	Unc5a	20
ENSMUSG00000100706	Gm19744	20		ENSMUSG00000020099	Unc5b	16
ENSMUSG00000066057	Gm1976	6		ENSMUSG00000059921	Unc5c	19
ENSMUSG00000099488	Gm19774	20		ENSMUSG00000063626	Unc5d	17
ENSMUSG00000113495	Gm19792	10		ENSMUSG00000021198	Unc79	7
ENSMUSG00000092223	Gm19807	20		ENSMUSG00000055567	Unc80	18
ENSMUSG00000111897	Gm19810	9		ENSMUSG00000036908	Unc93b1	20
ENSMUSG00000093716	Gm19815	12		ENSMUSG00000029546	Uncx	9
ENSMUSG00000105302	Gm19817	20		ENSMUSG00000029591	Ung	20
ENSMUSG00000091194	Gm19840	9		ENSMUSG00000020770	Unk	20
ENSMUSG00000117582	Gm19853	20		ENSMUSG00000015127	Unkl	20
ENSMUSG00000102650	Gm19863	9		ENSMUSG00000033427	Upb1	1
ENSMUSG00000102961	Gm19918	20		ENSMUSG00000058301	Upf1	1
ENSMUSG00000110332	Gm19935	20		ENSMUSG00000043241	Upf2	1
ENSMUSG00000102331	Gm19938	20		ENSMUSG00000038398	Upf3a	4
ENSMUSG00000093858	Gm19967	20		ENSMUSG00000036572	Upf3b	20
ENSMUSG00000112592	Gm19972	20		ENSMUSG00000006313	Upk1a	20
ENSMUSG00000078193	Gm2000	19		ENSMUSG00000049436	Upk1b	20
ENSMUSG00000117183	Gm20008	9		ENSMUSG00000020407	Upp1	20
ENSMUSG00000097640	Gm20033	20		ENSMUSG00000026839	Upp2	9
ENSMUSG00000093579	Gm20036	20		ENSMUSG00000073016	Uprt	20
ENSMUSG00000095648	Gm2004	20		ENSMUSG00000005882	Uqcc1	20
ENSMUSG00000103983	Gm20045	2		ENSMUSG00000024208	Uqcc2	12
ENSMUSG00000103847	Gm20056	20		ENSMUSG00000071654	Uqcc3	12
ENSMUSG00000113429	Gm20063	1		ENSMUSG00000059534	Uqcr10	12
ENSMUSG00000114133	Gm20075	20		ENSMUSG00000020163	Uqcr11	12
ENSMUSG00000108778	Gm20083	20		ENSMUSG00000021520	Uqcrb	12
ENSMUSG00000117385	Gm20147	20		ENSMUSG00000025651	Uqcrc1	20
ENSMUSG00000103483	Gm20162	1		ENSMUSG00000030884	Uqcrc2	20
ENSMUSG00000110611	Gm20163	20		ENSMUSG00000038462	Uqcrfs1	20
ENSMUSG00000104257	Gm20172	20		ENSMUSG00000063882	Uqcrh	12
ENSMUSG00000106874	Gm20186	20		ENSMUSG00000044894	Uqcrq	12
ENSMUSG00000112390	Gm20208	20		ENSMUSG00000039929	Urb1	1
ENSMUSG00000110218	Gm20219	13		ENSMUSG00000031976	Urb2	20
ENSMUSG00000114503	Gm20242	9		ENSMUSG00000049680	Urgcp	20
ENSMUSG00000102059	Gm20257	20		ENSMUSG00000030421	Uri1	20

ENSMUSG00000112795	Gm2027	9		ENSMUSG00000069020	Urm1	20
ENSMUSG00000108551	Gm20274	20		ENSMUSG00000028684	Urod	20
ENSMUSG00000110986	Gm20276	20		ENSMUSG00000030979	Uros	20
ENSMUSG00000111080	Gm20300	3		ENSMUSG00000031792	Usb1	20
ENSMUSG00000101599	Gm20342	1		ENSMUSG00000002395	Use1	3
ENSMUSG00000108264	Gm20371	4		ENSMUSG00000026641	Usf1	20
ENSMUSG00000107412	Gm20383	20		ENSMUSG00000058239	Usf2	9
ENSMUSG00000092603	Gm20386	20		ENSMUSG00000068284	Usf3	10
ENSMUSG00000092448	Gm20387	20		ENSMUSG00000030838	Ush1c	20
ENSMUSG00000092509	Gm20394	20		ENSMUSG00000034911	Ushbp1	20
ENSMUSG00000092405	Gm20402	20		ENSMUSG00000029407	Uso1	3
ENSMUSG00000092495	Gm20405	20		ENSMUSG00000028560	Usp1	20
ENSMUSG00000092181	Gm20432	20		ENSMUSG00000031826	Usp10	20
ENSMUSG00000092258	Gm20444	20		ENSMUSG00000031066	Usp11	20
ENSMUSG00000092394	Gm20445	20		ENSMUSG00000029640	Usp12	4
ENSMUSG00000092592	Gm20449	20		ENSMUSG00000056900	Usp13	3
ENSMUSG00000092187	Gm20457	20		ENSMUSG00000047879	Usp14	9
ENSMUSG00000092395	Gm20463	9		ENSMUSG00000020124	Usp15	3
ENSMUSG00000092565	Gm20477	20		ENSMUSG00000025616	Usp16	20
ENSMUSG00000092549	Gm20491	20		ENSMUSG00000030107	Usp18	20
ENSMUSG00000092593	Gm20492	20		ENSMUSG00000006676	Usp19	9
ENSMUSG00000021139	Gm20498	20		ENSMUSG00000032010	Usp2	9
ENSMUSG00000092536	Gm20501	20		ENSMUSG00000026854	Usp20	3
ENSMUSG00000092371	Gm20511	20		ENSMUSG00000053483	Usp21	9
ENSMUSG00000092242	Gm20515	20		ENSMUSG00000042506	Usp22	1
ENSMUSG00000023984	Gm20517	20		ENSMUSG00000028514	Usp24	2
ENSMUSG00000092470	Gm20518	20		ENSMUSG00000022867	Usp25	1
ENSMUSG00000092241	Gm20522	20		ENSMUSG00000046269	Usp27x	20
ENSMUSG00000092373	Gm20535	20		ENSMUSG00000032267	Usp28	9
ENSMUSG00000092386	Gm20536	20		ENSMUSG00000051527	Usp29	9
ENSMUSG00000092569	Gm20544	18		ENSMUSG00000032376	Usp3	19
ENSMUSG00000092354	Gm20548	20		ENSMUSG00000029592	Usp30	20
ENSMUSG00000115161	Gm20555	20		ENSMUSG00000063317	Usp31	12
ENSMUSG00000106734	Gm20559	20		ENSMUSG00000000804	Usp32	3
ENSMUSG00000105233	Gm20568	20		ENSMUSG00000025437	Usp33	20
ENSMUSG00000074903	Gm2058	20		ENSMUSG00000056342	Usp34	16
ENSMUSG00000096887	Gm20594	20		ENSMUSG00000035713	Usp35	20
ENSMUSG00000041716	Gm20604	3		ENSMUSG00000033909	Usp36	20
ENSMUSG00000029720	Gm20605	1		ENSMUSG00000033364	Usp37	20
ENSMUSG00000097615	Gm2061	12		ENSMUSG00000038250	Usp38	20
ENSMUSG00000093507	Gm20627	20		ENSMUSG00000032612	Usp4	20
ENSMUSG00000093656	Gm20628	13		ENSMUSG00000005501	Usp40	20
ENSMUSG00000093577	Gm20632	20		ENSMUSG00000051306	Usp42	12
ENSMUSG00000093553	Gm20633	20		ENSMUSG00000020905	Usp43	20
ENSMUSG00000070392	Gm20634	10		ENSMUSG00000040455	Usp45	4
ENSMUSG00000093720	Gm20635	1		ENSMUSG00000054814	Usp46	20
ENSMUSG00000093436	Gm20646	9		ENSMUSG00000059263	Usp47	1
ENSMUSG00000093672	Gm20655	12		ENSMUSG00000043411	Usp48	9
ENSMUSG00000079323	Gm20661	20		ENSMUSG00000090115	Usp49	20
ENSMUSG00000093465	Gm20682	9		ENSMUSG00000038429	Usp5	20
ENSMUSG00000093405	Gm20684	20		ENSMUSG00000027364	Usp50	20
ENSMUSG00000093384	Gm20689	20		ENSMUSG00000067215	Usp51	20
ENSMUSG00000093505	Gm20691	20		ENSMUSG00000039701	Usp53	9
ENSMUSG00000030068	Gm20696	9		ENSMUSG00000034235	Usp54	15

ENSMUSG00000093622	Gm20703	20		ENSMUSG00000039046	Usp6nl	9
ENSMUSG00000093559	Gm20705	7		ENSMUSG00000022710	Usp7	2
ENSMUSG00000093594	Gm20707	20		ENSMUSG00000027363	Usp8	19
ENSMUSG00000093677	Gm20712	20		ENSMUSG00000031010	Usp9x	2
ENSMUSG00000045929	Gm20715	9		ENSMUSG00000041264	Uspl1	20
ENSMUSG00000090625	Gm20721	18		ENSMUSG00000047712	Ust	1
ENSMUSG00000102912	Gm20731	20		ENSMUSG00000028907	Utp11	3
ENSMUSG00000093387	Gm20732	20		ENSMUSG00000063785	Utp14a	19
ENSMUSG00000104117	Gm20743	20		ENSMUSG00000079470	Utp14b	16
ENSMUSG00000110016	Gm20751	20		ENSMUSG00000041747	Utp15	20
ENSMUSG00000103428	Gm20754	5		ENSMUSG00000054079	Utp18	9
ENSMUSG00000098092	Gm2076	9		ENSMUSG00000004356	Utp20	2
ENSMUSG00000118197	Gm20762	7		ENSMUSG00000022313	Utp23	9
ENSMUSG00000106190	Gm20768	20		ENSMUSG00000016181	Utp25	20
ENSMUSG00000094421	Gm20778	20		ENSMUSG00000070697	Utp3	20
ENSMUSG00000093800	Gm20796	2		ENSMUSG00000041438	Utp4	20
ENSMUSG00000078180	Gm20900	20		ENSMUSG00000035575	Utp6	20
ENSMUSG00000095193	Gm20939	19		ENSMUSG00000019820	Utrn	9
ENSMUSG00000095403	Gm21092	20		ENSMUSG00000068457	Uty	20
ENSMUSG00000096256	Gm21093	12		ENSMUSG00000035354	Uvrag	20
ENSMUSG00000095294	Gm21119	10		ENSMUSG00000037355	Uvssa	1
ENSMUSG00000097789	Gm2115	20		ENSMUSG00000057363	Uxs1	20
ENSMUSG00000104651	Gm21168	20		ENSMUSG00000001134	Uxt	20
ENSMUSG00000112975	Gm21297	1		ENSMUSG00000010936	Vac14	20
ENSMUSG00000103713	Gm2136	20		ENSMUSG00000030337	Vamp1	18
ENSMUSG00000114940	Gm21378	20		ENSMUSG00000020894	Vamp2	20
ENSMUSG00000094530	Gm21399	20		ENSMUSG00000028955	Vamp3	20
ENSMUSG00000097288	Gm2155	20		ENSMUSG00000026696	Vamp4	6
ENSMUSG00000113280	Gm21559	20		ENSMUSG00000051412	Vamp7	18
ENSMUSG00000096006	Gm21596	12		ENSMUSG00000105078	Vamp9	20
ENSMUSG00000098760	Gm2164	19		ENSMUSG00000027860	Vangl1	18
ENSMUSG00000116840	Gm21691	20		ENSMUSG00000026556	Vangl2	9
ENSMUSG00000095280	Gm21738	20		ENSMUSG00000024091	Vapa	20
ENSMUSG00000110458	Gm21769	20		ENSMUSG00000054455	Vapb	18
ENSMUSG00000095123	Gm21781	20		ENSMUSG00000007029	Vars	20
ENSMUSG00000096401	Gm21811	20		ENSMUSG00000038838	Vars2	20
ENSMUSG00000096299	Gm21814	20		ENSMUSG00000021256	Vash1	10
ENSMUSG00000096870	Gm21816	20		ENSMUSG00000037568	Vash2	18
ENSMUSG00000095651	Gm21817	1		ENSMUSG00000039646	Vasn	20
ENSMUSG00000094030	Gm21833	1		ENSMUSG00000030403	Vasp	20
ENSMUSG00000095913	Gm21844	20		ENSMUSG00000034993	Vat1	20
ENSMUSG00000094832	Gm21846	3		ENSMUSG00000046844	Vat1l	3
ENSMUSG00000095366	Gm21860	20		ENSMUSG00000065145	Vaultrc5	10
ENSMUSG00000095562	Gm21887	20		ENSMUSG00000034116	Vav1	20
ENSMUSG00000116995	Gm21926	20		ENSMUSG00000009621	Vav2	9
ENSMUSG00000095334	Gm21984	20		ENSMUSG00000033721	Vav3	18
ENSMUSG00000096056	Gm21986	20		ENSMUSG00000006270	Vax1	9
ENSMUSG00000083829	Gm2199	20		ENSMUSG00000034777	Vax2	20
ENSMUSG00000096370	Gm21992	12		ENSMUSG00000031197	Vbp1	9
ENSMUSG00000075833	Gm22003	10		ENSMUSG00000027962	Vcam1	13
ENSMUSG00000089417	Gm22009	10		ENSMUSG00000021614	Vcan	20
ENSMUSG00000084686	Gm22027	10		ENSMUSG00000021823	Vcl	7
ENSMUSG00000064923	Gm22042	20		ENSMUSG00000028452	Vcp	3
ENSMUSG00000065327	Gm22058	20		ENSMUSG00000083327	Vcp-rs	20

ENSMUSG00000084739	Gm22067	20		ENSMUSG00000045210	Vcpip1	3
ENSMUSG00000096205	Gm22068	20		ENSMUSG00000049882	Vcpkmt	20
ENSMUSG00000093334	Gm22118	2		ENSMUSG00000020402	Vdac1	11
ENSMUSG00000065778	Gm22154	10		ENSMUSG00000021771	Vdac2	20
ENSMUSG00000079297	Gm2223	20		ENSMUSG00000008892	Vdac3	20
ENSMUSG00000064410	Gm22247	10		ENSMUSG00000075053	Vdac3-ps1	20
ENSMUSG00000110057	Gm2225	20		ENSMUSG00000022479	Vdr	20
ENSMUSG00000096866	Gm22260	20		ENSMUSG00000023951	Vegfa	18
ENSMUSG00000094131	Gm22265	10		ENSMUSG00000024962	Vegfb	20
ENSMUSG00000077222	Gm22270	10		ENSMUSG00000031520	Vegfc	20
ENSMUSG00000064620	Gm22303	10		ENSMUSG00000031380	Vegfd	20
ENSMUSG00000064626	Gm22306	10		ENSMUSG00000036099	Vezt	1
ENSMUSG00000088633	Gm22313	12		ENSMUSG00000037428	Vgf	20
ENSMUSG00000096206	Gm22317	20		ENSMUSG00000049641	Vgll2	1
ENSMUSG00000088699	Gm22328	20		ENSMUSG00000091243	Vgll3	20
ENSMUSG00000065036	Gm22362	10		ENSMUSG00000030315	Vgll4	20
ENSMUSG00000077772	Gm22373	20		ENSMUSG00000033933	Vhl	20
ENSMUSG00000103138	Gm2238	13		ENSMUSG00000026175	Vil1	20
ENSMUSG00000092666	Gm22408	9		ENSMUSG00000038775	Vill	1
ENSMUSG00000064994	Gm22422	10		ENSMUSG00000026728	Vim	1
ENSMUSG00000087881	Gm22442	10		ENSMUSG00000019772	Vip	20
ENSMUSG00000080465	Gm22486	10		ENSMUSG00000021038	Vipas39	20
ENSMUSG00000077681	Gm22496	20		ENSMUSG00000032528	Vipr1	9
ENSMUSG00000089607	Gm22500	20		ENSMUSG00000011171	Vipr2	18
ENSMUSG00000089606	Gm22501	20		ENSMUSG00000040720	Virma	7
ENSMUSG00000064890	Gm22505	20		ENSMUSG00000098320	Vis1	20
ENSMUSG00000096349	Gm22513	10		ENSMUSG00000024076	Vit	20
ENSMUSG00000088891	Gm22516	20		ENSMUSG00000096145	Vkorc1	12
ENSMUSG00000110244	Gm2253	20		ENSMUSG00000066735	Vkorc1l1	20
ENSMUSG00000089447	Gm22532	20		ENSMUSG00000024924	Vldlr	10
ENSMUSG00000089446	Gm22533	20		ENSMUSG00000073131	Vma21	9
ENSMUSG00000088793	Gm22540	20		ENSMUSG00000054723	Vmac	20
ENSMUSG00000077535	Gm22543	20		ENSMUSG00000064259	Vmn1r13	20
ENSMUSG00000065661	Gm22580	20		ENSMUSG00000100586	Vmn1r90	1
ENSMUSG00000065669	Gm22581	20		ENSMUSG00000074164	Vmn2r-ps54	20
ENSMUSG00000088226	Gm22596	20		ENSMUSG00000027824	Vmn2r1	1
ENSMUSG00000084628	Gm22613	20		ENSMUSG00000095093	Vmn2r111	20
ENSMUSG00000094452	Gm22623	20		ENSMUSG00000072778	Vmn2r27	9
ENSMUSG00000088769	Gm22637	20		ENSMUSG00000095730	Vmn2r29	20
ENSMUSG00000092879	Gm22650	18		ENSMUSG00000096002	Vmn2r53	20
ENSMUSG00000077380	Gm22661	20		ENSMUSG00000090762	Vmn2r56	9
ENSMUSG00000089343	Gm22667	20		ENSMUSG00000066537	Vmn2r57	20
ENSMUSG00000065788	Gm22682	20		ENSMUSG00000070601	Vmn2r84	3
ENSMUSG00000064782	Gm22685	20		ENSMUSG00000092048	Vmn2r85	3
ENSMUSG00000080543	Gm22709	20		ENSMUSG00000092162	Vmn2r86	2
ENSMUSG00000080544	Gm22714	20		ENSMUSG00000091511	Vmn2r87	3
ENSMUSG00000095178	Gm22716	20		ENSMUSG00000018171	Vmp1	1
ENSMUSG00000088712	Gm22728	20		ENSMUSG00000037788	Vopp1	2
ENSMUSG00000088711	Gm22731	20		ENSMUSG00000032127	Vps11	20
ENSMUSG00000065374	Gm22738	20		ENSMUSG00000046230	Vps13a	3
ENSMUSG00000065371	Gm22739	9		ENSMUSG00000037646	Vps13b	7
ENSMUSG00000064427	Gm22748	10		ENSMUSG00000035284	Vps13c	3
ENSMUSG00000065591	Gm22753	10		ENSMUSG00000020220	Vps13d	19
ENSMUSG00000087768	Gm22755	1		ENSMUSG00000027411	Vps16	20

ENSMUSG00000088990	Gm22767	10		ENSMUSG00000034216	Vps18	20
ENSMUSG00000077276	Gm22784	18		ENSMUSG00000078656	Vps25	20
ENSMUSG00000077274	Gm22786	10		ENSMUSG00000020078	Vps26a	19
ENSMUSG00000065926	Gm22798	20		ENSMUSG00000031988	Vps26b	9
ENSMUSG00000089061	Gm22839	20		ENSMUSG00000022898	Vps26c	9
ENSMUSG00000092713	Gm22858	20		ENSMUSG00000115987	Vps28	20
ENSMUSG00000065883	Gm22865	20		ENSMUSG00000029462	Vps29	20
ENSMUSG00000088001	Gm22883	10		ENSMUSG00000029434	Vps33a	20
ENSMUSG00000096346	Gm22887	20		ENSMUSG00000030534	Vps33b	19
ENSMUSG00000089265	Gm22888	20		ENSMUSG00000031696	Vps35	4
ENSMUSG00000089185	Gm22940	20		ENSMUSG00000030982	Vps35l	20
ENSMUSG00000080370	Gm22952	20		ENSMUSG00000031479	Vps36	20
ENSMUSG00000080374	Gm22953	20		ENSMUSG00000031600	Vps37a	12
ENSMUSG00000084460	Gm22957	20		ENSMUSG00000066278	Vps37b	9
ENSMUSG00000094229	Gm22969	20		ENSMUSG00000048832	Vps37c	20
ENSMUSG00000065232	Gm22973	20		ENSMUSG00000043614	Vps37d	20
ENSMUSG00000064542	Gm22980	20		ENSMUSG00000027291	Vps39	20
ENSMUSG00000064779	Gm22997	10		ENSMUSG00000041236	Vps41	18
ENSMUSG00000064738	Gm23054	20		ENSMUSG00000015747	Vps45	20
ENSMUSG00000076305	Gm23063	10		ENSMUSG00000031913	Vps4a	20
ENSMUSG00000092257	Gm2308	20		ENSMUSG00000009907	Vps4b	20
ENSMUSG00000088176	Gm23094	20		ENSMUSG00000001376	Vps50	4
ENSMUSG00000089103	Gm23114	20		ENSMUSG00000024797	Vps51	20
ENSMUSG00000089235	Gm23119	20		ENSMUSG00000024319	Vps52	20
ENSMUSG00000088278	Gm23121	20		ENSMUSG00000017288	Vps53	20
ENSMUSG00000077990	Gm23134	20		ENSMUSG00000020128	Vps54	20
ENSMUSG00000095868	Gm23136	20		ENSMUSG00000008958	Vps72	20
ENSMUSG00000065299	Gm23138	19		ENSMUSG00000033653	Vps8	20
ENSMUSG00000094405	Gm23143	10		ENSMUSG00000001062	Vps9d1	20
ENSMUSG00000093830	Gm23145	10		ENSMUSG00000021115	Vrk1	9
ENSMUSG00000093064	Gm23153	20		ENSMUSG00000064090	Vrk2	20
ENSMUSG00000092829	Gm23159	20		ENSMUSG00000002205	Vrk3	20
ENSMUSG00000087782	Gm23169	20		ENSMUSG00000071235	Vrtn	20
ENSMUSG00000087785	Gm23172	20		ENSMUSG00000066894	Vsig10	18
ENSMUSG00000084544	Gm23183	20		ENSMUSG00000070604	Vsig10l	20
ENSMUSG00000111554	Gm232	20		ENSMUSG00000001943	Vsig2	20
ENSMUSG00000077567	Gm23200	20		ENSMUSG00000020101	Vsir	20
ENSMUSG00000077565	Gm23201	10		ENSMUSG00000054459	Vsnl1	19
ENSMUSG00000077564	Gm23202	10		ENSMUSG00000048834	Vstm2a	1
ENSMUSG00000089296	Gm23205	20		ENSMUSG00000039257	Vstm2b	20
ENSMUSG00000089292	Gm23209	20		ENSMUSG00000037843	Vstm2l	20
ENSMUSG00000084664	Gm23213	20		ENSMUSG00000050666	Vstm4	20
ENSMUSG00000064941	Gm23238	20		ENSMUSG00000031937	Vstm5	20
ENSMUSG00000064943	Gm23240	20		ENSMUSG00000019868	Vta1	9
ENSMUSG00000065304	Gm23245	10		ENSMUSG00000051076	Vtcn1	3
ENSMUSG00000088942	Gm23260	20		ENSMUSG00000024983	Vti1a	20
ENSMUSG00000064486	Gm23293	12		ENSMUSG00000021124	Vti1b	1
ENSMUSG00000065118	Gm23297	10		ENSMUSG00000017344	Vtn	20
ENSMUSG00000064647	Gm23301	10		ENSMUSG00000042116	Vwa1	20
ENSMUSG00000065752	Gm23344	1		ENSMUSG00000030889	Vwa3a	14
ENSMUSG00000065750	Gm23346	20		ENSMUSG00000050122	Vwa3b	14
ENSMUSG00000094726	Gm23355	10		ENSMUSG00000023186	Vwa5a	1
ENSMUSG00000093262	Gm23413	20		ENSMUSG00000028753	Vwa5b1	13
ENSMUSG00000077194	Gm23431	20		ENSMUSG00000046613	Vwa5b2	9

ENSMUSG00000064853	Gm23442	10		ENSMUSG00000007030	Vwa7	1
ENSMUSG00000065198	Gm23462	20		ENSMUSG00000058997	Vwa8	12
ENSMUSG00000084417	Gm23463	20		ENSMUSG00000050830	Vwc2	18
ENSMUSG00000094050	Gm23472	14		ENSMUSG00000045648	Vwc2l	18
ENSMUSG00000089508	Gm23473	20		ENSMUSG00000043789	Vwce	20
ENSMUSG00000080485	Gm23493	20		ENSMUSG00000001930	Vwf	20
ENSMUSG00000065836	Gm23502	9		ENSMUSG00000067879	Vxn	5
ENSMUSG00000065014	Gm23509	20		ENSMUSG00000024283	Wac	2
ENSMUSG00000065018	Gm23511	10		ENSMUSG00000041408	Wapl	2
ENSMUSG00000088836	Gm23546	20		ENSMUSG00000021266	Wars	20
ENSMUSG00000088835	Gm23547	12		ENSMUSG00000004233	Wars2	20
ENSMUSG00000088442	Gm23553	20		ENSMUSG00000031165	Was	20
ENSMUSG00000096882	Gm23570	20		ENSMUSG00000019831	Wasf1	18
ENSMUSG00000089158	Gm23583	20		ENSMUSG00000028868	Wasf2	20
ENSMUSG00000077733	Gm23608	20		ENSMUSG00000029636	Wasf3	20
ENSMUSG00000092819	Gm23639	10		ENSMUSG00000024101	Washc1	2
ENSMUSG00000084572	Gm23657	9		ENSMUSG00000024104	Washc2	20
ENSMUSG00000096391	Gm23686	20		ENSMUSG00000020056	Washc3	20
ENSMUSG00000075829	Gm23692	20		ENSMUSG00000034560	Washc4	20
ENSMUSG00000089465	Gm23702	20		ENSMUSG00000022350	Washc5	3
ENSMUSG00000064936	Gm23722	12		ENSMUSG00000029684	Wasl	20
ENSMUSG00000065687	Gm23734	10		ENSMUSG00000030035	Wbp1	20
ENSMUSG00000088208	Gm23751	10		ENSMUSG00000030216	Wbp11	20
ENSMUSG00000088972	Gm23761	20		ENSMUSG00000047731	Wbp1l	1
ENSMUSG00000094826	Gm23804	20		ENSMUSG00000034341	Wbp2	2
ENSMUSG00000092898	Gm23806	10		ENSMUSG00000022023	Wbp4	20
ENSMUSG00000092896	Gm23811	20		ENSMUSG00000054909	Wbscr25	20
ENSMUSG00000065764	Gm23850	20		ENSMUSG00000051721	Wdcp	20
ENSMUSG00000093921	Gm23864	20		ENSMUSG00000073643	Wdfy1	1
ENSMUSG00000115457	Gm2387	20		ENSMUSG00000014547	Wdfy2	3
ENSMUSG00000093170	Gm23873	20		ENSMUSG00000043940	Wdfy3	3
ENSMUSG00000084632	Gm23887	12		ENSMUSG00000051506	Wdfy4	20
ENSMUSG00000092997	Gm23917	9		ENSMUSG00000037572	Wdhd1	20
ENSMUSG00000096297	Gm23934	3		ENSMUSG00000020319	Wdpcp	20
ENSMUSG00000076258	Gm23935	20		ENSMUSG00000005103	Wdr1	19
ENSMUSG00000077611	Gm23946	10		ENSMUSG00000042055	Wdr11	20
ENSMUSG00000095624	Gm23947	10		ENSMUSG00000026019	Wdr12	20
ENSMUSG00000087836	Gm23956	20		ENSMUSG00000031166	Wdr13	9
ENSMUSG00000065251	Gm23971	10		ENSMUSG00000039375	Wdr17	9
ENSMUSG00000080680	Gm23989	20		ENSMUSG00000035754	Wdr18	1
ENSMUSG00000088682	Gm23999	20		ENSMUSG00000037890	Wdr19	20
ENSMUSG00000088359	Gm24009	20		ENSMUSG00000037957	Wdr20	20
ENSMUSG00000095023	Gm24019	20		ENSMUSG00000025737	Wdr24	20
ENSMUSG00000089242	Gm24056	20		ENSMUSG00000040877	Wdr25	20
ENSMUSG00000088118	Gm24059	20		ENSMUSG00000038733	Wdr26	20
ENSMUSG00000088119	Gm24060	12		ENSMUSG00000046991	Wdr27	20
ENSMUSG00000095491	Gm24088	3		ENSMUSG00000033285	Wdr3	20
ENSMUSG00000096563	Gm24095	20		ENSMUSG00000028391	Wdr31	10
ENSMUSG00000077167	Gm24119	10		ENSMUSG00000024400	Wdr33	20
ENSMUSG00000089161	Gm24130	10		ENSMUSG00000039715	Wdr34	3
ENSMUSG00000064566	Gm24144	20		ENSMUSG00000066643	Wdr35	1
ENSMUSG00000064565	Gm24145	20		ENSMUSG00000038299	Wdr36	20
ENSMUSG00000064694	Gm24146	10		ENSMUSG00000021147	Wdr37	2
ENSMUSG00000086119	Gm2415	19		ENSMUSG00000035295	Wdr38	20

ENSMUSG00000093006	Gm24157	20		ENSMUSG00000024037	Wdr4	20
ENSMUSG00000088609	Gm24187	20		ENSMUSG00000042015	Wdr41	9
ENSMUSG00000088600	Gm24189	20		ENSMUSG00000041057	Wdr43	1
ENSMUSG00000080455	Gm24194	20		ENSMUSG00000036769	Wdr44	20
ENSMUSG00000082305	Gm2420	1		ENSMUSG00000039382	Wdr45	20
ENSMUSG00000061724	Gm2423	9		ENSMUSG00000025173	Wdr45b	18
ENSMUSG00000077505	Gm24233	10		ENSMUSG00000024312	Wdr46	20
ENSMUSG00000087943	Gm24245	10		ENSMUSG00000105893	Wdr46-ps	20
ENSMUSG00000065675	Gm24260	10		ENSMUSG00000040389	Wdr47	4
ENSMUSG00000096243	Gm24265	10		ENSMUSG00000032512	Wdr48	20
ENSMUSG00000076281	Gm24270	20		ENSMUSG00000104301	Wdr49	14
ENSMUSG00000088254	Gm24289	10		ENSMUSG00000026917	Wdr5	20
ENSMUSG00000064822	Gm24292	10		ENSMUSG00000022787	Wdr53	20
ENSMUSG00000095590	Gm24305	10		ENSMUSG00000030032	Wdr54	9
ENSMUSG00000093854	Gm24310	20		ENSMUSG00000042660	Wdr55	20
ENSMUSG00000077391	Gm24336	10		ENSMUSG00000031959	Wdr59	20
ENSMUSG00000077394	Gm24339	20		ENSMUSG00000034379	Wdr5b	20
ENSMUSG00000089337	Gm24346	9		ENSMUSG00000066357	Wdr6	17
ENSMUSG00000077581	Gm24373	20		ENSMUSG00000042050	Wdr60	20
ENSMUSG00000084644	Gm24379	20		ENSMUSG00000061559	Wdr61	20
ENSMUSG00000080555	Gm24396	20		ENSMUSG00000037020	Wdr62	9
ENSMUSG00000088493	Gm24401	20		ENSMUSG00000043020	Wdr63	14
ENSMUSG00000094377	Gm24407	10		ENSMUSG00000029442	Wdr66	14
ENSMUSG00000065360	Gm24412	20		ENSMUSG00000040560	Wdr7	3
ENSMUSG00000065911	Gm24447	10		ENSMUSG00000039828	Wdr70	20
ENSMUSG00000098113	Gm2445	20		ENSMUSG00000044976	Wdr72	13
ENSMUSG00000094704	Gm24487	1		ENSMUSG00000025722	Wdr73	20
ENSMUSG00000065738	Gm24494	10		ENSMUSG00000042729	Wdr74	12
ENSMUSG00000093958	Gm24495	20		ENSMUSG00000025995	Wdr75	1
ENSMUSG00000093956	Gm24497	10		ENSMUSG00000027242	Wdr76	20
ENSMUSG00000089070	Gm24498	20		ENSMUSG00000000561	Wdr77	20
ENSMUSG00000088308	Gm24507	10		ENSMUSG00000035126	Wdr78	1
ENSMUSG00000092702	Gm24514	10		ENSMUSG00000045374	Wdr81	12
ENSMUSG00000064851	Gm24525	20		ENSMUSG00000020257	Wdr82	20
ENSMUSG00000088573	Gm24530	20		ENSMUSG00000005150	Wdr83	10
ENSMUSG00000093874	Gm24539	9		ENSMUSG00000059355	Wdr83os	20
ENSMUSG00000089271	Gm24552	20		ENSMUSG00000055235	Wdr86	13
ENSMUSG00000092928	Gm24563	20		ENSMUSG00000045690	Wdr89	20
ENSMUSG00000077155	Gm24582	20		ENSMUSG00000073434	Wdr90	1
ENSMUSG00000084453	Gm24596	20		ENSMUSG00000058486	Wdr91	20
ENSMUSG00000065200	Gm24613	20		ENSMUSG00000078970	Wdr92	1
ENSMUSG00000065203	Gm24614	10		ENSMUSG00000039099	Wdr93	20
ENSMUSG00000065208	Gm24616	10		ENSMUSG00000029658	Wdr95	20
ENSMUSG00000064558	Gm24620	10		ENSMUSG00000026988	Wdsub1	1
ENSMUSG00000102215	Gm2464	20		ENSMUSG00000037622	Wdtc1	20
ENSMUSG00000087739	Gm24644	20		ENSMUSG00000031016	Wee1	20
ENSMUSG00000098102	Gm2467	12		ENSMUSG00000037159	Wee2	20
ENSMUSG00000077347	Gm24671	20		ENSMUSG00000023336	Wfdc1	1
ENSMUSG00000088856	Gm24727	10		ENSMUSG00000000983	Wfdc18	20
ENSMUSG00000088850	Gm24729	20		ENSMUSG00000017723	Wfdc2	13
ENSMUSG00000089133	Gm24771	20		ENSMUSG00000076434	Wfdc3	20
ENSMUSG00000077472	Gm24791	18		ENSMUSG00000071192	Wfikkn1	20
ENSMUSG00000095701	Gm24830	20		ENSMUSG00000044177	Wfikkn2	13
ENSMUSG00000089405	Gm24876	20		ENSMUSG00000039474	Wfs1	3

ENSMUSG00000089407	Gm24878	20		ENSMUSG00000045795	Whamm	9
ENSMUSG00000095133	Gm24897	10		ENSMUSG00000039137	Whrn	20
ENSMUSG00000113563	Gm2492	20		ENSMUSG00000020218	Wif1	20
ENSMUSG00000064952	Gm24920	10		ENSMUSG00000075284	Wipf1	15
ENSMUSG00000094304	Gm24923	20		ENSMUSG00000038013	Wipf2	18
ENSMUSG00000094306	Gm24924	10		ENSMUSG00000086040	Wipf3	9
ENSMUSG00000065331	Gm24927	20		ENSMUSG00000041895	Wipi1	20
ENSMUSG00000098425	Gm24949	3		ENSMUSG00000029578	Wipi2	20
ENSMUSG00000064377	Gm24966	20		ENSMUSG00000024050	Wiz	1
ENSMUSG00000095361	Gm24967	10		ENSMUSG00000028173	Wls	9
ENSMUSG00000096046	Gm24969	20		ENSMUSG00000045962	Wnk1	20
ENSMUSG00000077645	Gm24983	20		ENSMUSG00000037989	Wnk2	18
ENSMUSG00000089026	Gm25014	19		ENSMUSG00000041245	Wnk3	20
ENSMUSG00000064867	Gm25039	20		ENSMUSG00000035112	Wnk4	1
ENSMUSG00000084616	Gm25047	20		ENSMUSG00000026167	Wnt10a	20
ENSMUSG00000093734	Gm25082	3		ENSMUSG00000022996	Wnt10b	20
ENSMUSG00000095676	Gm25099	10		ENSMUSG00000010797	Wnt2	19
ENSMUSG00000095678	Gm25101	20		ENSMUSG00000027840	Wnt2b	20
ENSMUSG00000084421	Gm25107	10		ENSMUSG00000000125	Wnt3	18
ENSMUSG00000087819	Gm25117	10		ENSMUSG00000036856	Wnt4	6
ENSMUSG00000065273	Gm25128	10		ENSMUSG00000021994	Wnt5a	20
ENSMUSG00000064585	Gm25129	10		ENSMUSG00000030170	Wnt5b	12
ENSMUSG00000064586	Gm25132	20		ENSMUSG00000030093	Wnt7a	20
ENSMUSG00000064581	Gm25133	20		ENSMUSG00000022382	Wnt7b	12
ENSMUSG00000064964	Gm25147	1		ENSMUSG00000036961	Wnt8b	1
ENSMUSG00000094068	Gm25156	10		ENSMUSG00000000126	Wnt9a	9
ENSMUSG00000095009	Gm25175	20		ENSMUSG00000018486	Wnt9b	18
ENSMUSG00000088371	Gm25180	20		ENSMUSG00000041346	Wrap53	20
ENSMUSG00000088378	Gm25184	20		ENSMUSG00000029029	Wrap73	20
ENSMUSG00000065847	Gm25188	20		ENSMUSG00000023147	Wrb	1
ENSMUSG00000065845	Gm25189	20		ENSMUSG00000031583	Wrn	20
ENSMUSG00000093404	Gm25190	20		ENSMUSG00000021400	Wrnip1	20
ENSMUSG00000088820	Gm25224	16		ENSMUSG00000017677	Wsb1	9
ENSMUSG00000096547	Gm25235	19		ENSMUSG00000029364	Wsb2	3
ENSMUSG00000094977	Gm25256	10		ENSMUSG00000084020	Wsb2-ps	20
ENSMUSG00000092675	Gm25262	1		ENSMUSG00000020811	Wscd1	16
ENSMUSG00000084744	Gm25291	10		ENSMUSG00000063430	Wscd2	6
ENSMUSG00000058567	Gm2531	20		ENSMUSG00000016458	Wt1	1
ENSMUSG00000095738	Gm25313	10		ENSMUSG00000060475	Wtap	2
ENSMUSG00000089586	Gm25348	20		ENSMUSG00000036459	Wtip	20
ENSMUSG00000094655	Gm25360	10		ENSMUSG00000018849	Wwc1	20
ENSMUSG00000089475	Gm25363	20		ENSMUSG00000031563	Wwc2	20
ENSMUSG00000087963	Gm25394	10		ENSMUSG00000004637	Wwox	18
ENSMUSG00000087968	Gm25395	12		ENSMUSG00000041058	Wwp1	12
ENSMUSG00000088224	Gm25406	20		ENSMUSG00000031930	Wwp2	20
ENSMUSG00000089698	Gm2541	17		ENSMUSG00000027803	Wwtr1	20
ENSMUSG00000065694	Gm25411	20		ENSMUSG00000019470	Xab2	20
ENSMUSG00000096222	Gm25412	10		ENSMUSG00000040483	Xaf1	20
ENSMUSG00000117771	Gm25432	20		ENSMUSG00000020484	Xbp1	20
ENSMUSG00000088902	Gm25435	20		ENSMUSG00000024066	Xdh	20
ENSMUSG00000094199	Gm25450	20		ENSMUSG00000025860	Xiap	3
ENSMUSG00000094440	Gm25453	20		ENSMUSG00000086503	Xist	19
ENSMUSG00000098019	Gm2546	20		ENSMUSG00000015342	Xk	18
ENSMUSG00000088008	Gm25492	10		ENSMUSG00000051951	Xkr4	19

ENSMUSG00000077615	Gm25499	20		ENSMUSG00000035067	Xkr6	9
ENSMUSG00000080530	Gm25540	20		ENSMUSG00000042631	Xkr7	20
ENSMUSG00000080538	Gm25541	10		ENSMUSG00000037752	Xkr8	20
ENSMUSG00000084625	Gm25544	20		ENSMUSG00000031258	Xkrx	20
ENSMUSG00000096841	Gm25596	20		ENSMUSG00000054626	Xlr	20
ENSMUSG00000095641	Gm25615	20		ENSMUSG00000057836	Xlr3a	20
ENSMUSG00000065934	Gm25630	20		ENSMUSG00000073125	Xlr3b	20
ENSMUSG00000077578	Gm25631	1		ENSMUSG00000099481	Xndc1	20
ENSMUSG00000094251	Gm25632	20		ENSMUSG00000028329	Xpa	20
ENSMUSG00000064609	Gm25635	20		ENSMUSG00000030094	Xpc	9
ENSMUSG00000064600	Gm25636	20		ENSMUSG00000025027	Xpnpep1	20
ENSMUSG00000096496	Gm25646	10		ENSMUSG00000022401	Xpnpep3	18
ENSMUSG00000094095	Gm25648	20		ENSMUSG00000020290	Xpo1	20
ENSMUSG00000089097	Gm25658	20		ENSMUSG00000021952	Xpo4	20
ENSMUSG00000106879	Gm2566	20		ENSMUSG00000067150	Xpo5	20
ENSMUSG00000096659	Gm25679	20		ENSMUSG00000000131	Xpo6	1
ENSMUSG00000065876	Gm25682	20		ENSMUSG00000022100	Xpo7	7
ENSMUSG00000093189	Gm25694	2		ENSMUSG00000034667	Xpot	1
ENSMUSG00000089258	Gm25711	20		ENSMUSG00000026469	Xpr1	20
ENSMUSG00000088128	Gm25720	9		ENSMUSG00000051768	Xrcc1	20
ENSMUSG00000115017	Gm2573	20		ENSMUSG00000028933	Xrcc2	1
ENSMUSG00000078162	Gm2574	20		ENSMUSG00000021287	Xrcc3	20
ENSMUSG00000077176	Gm25759	20		ENSMUSG00000021615	Xrcc4	20
ENSMUSG00000080365	Gm25776	10		ENSMUSG00000026187	Xrcc5	1
ENSMUSG00000084477	Gm25780	20		ENSMUSG00000022471	Xrcc6	20
ENSMUSG00000064655	Gm25788	10		ENSMUSG00000032410	Xrn1	12
ENSMUSG00000065220	Gm25794	20		ENSMUSG00000027433	Xrn2	18
ENSMUSG00000077607	Gm25803	20		ENSMUSG00000035211	Xrra1	1
ENSMUSG00000064682	Gm25813	10		ENSMUSG00000047434	Xxylt1	20
ENSMUSG00000070166	Gm25821	10		ENSMUSG00000035769	Xylb	20
ENSMUSG00000089549	Gm25831	20		ENSMUSG00000030657	Xylt1	20
ENSMUSG00000089542	Gm25835	10		ENSMUSG00000020868	Xylt2	18
ENSMUSG00000084537	Gm25845	20		ENSMUSG00000075054	Yae1d1	3
ENSMUSG00000080440	Gm25848	10		ENSMUSG00000022634	Yaf2	6
ENSMUSG00000064724	Gm25852	10		ENSMUSG00000053110	Yap1	9
ENSMUSG00000094609	Gm25870	20		ENSMUSG00000028811	Yars	6
ENSMUSG00000094605	Gm25873	20		ENSMUSG00000022792	Yars2	20
ENSMUSG00000094604	Gm25874	10		ENSMUSG00000033126	Ybey	20
ENSMUSG00000095260	Gm25890	20		ENSMUSG00000028639	Ybx1	9
ENSMUSG00000088246	Gm25911	20		ENSMUSG00000107035	Ybx1-ps2	18
ENSMUSG00000064833	Gm25926	20		ENSMUSG00000018554	Ybx2	20
ENSMUSG00000064719	Gm25930	20		ENSMUSG00000030189	Ybx3	20
ENSMUSG00000094476	Gm25934	10		ENSMUSG00000041774	Ydjc	20
ENSMUSG00000093843	Gm25939	10		ENSMUSG00000041215	Yeats2	1
ENSMUSG00000084433	Gm25945	20		ENSMUSG00000020171	Yeats4	20
ENSMUSG00000087790	Gm25970	10		ENSMUSG00000014932	Yes1	20
ENSMUSG00000096684	Gm25989	10		ENSMUSG00000024875	Yif1a	20
ENSMUSG00000075864	Gm26002	10		ENSMUSG00000030588	Yif1b	3
ENSMUSG00000089282	Gm26024	20		ENSMUSG00000057375	Yipf1	3
ENSMUSG00000064999	Gm26035	20		ENSMUSG00000032182	Yipf2	20
ENSMUSG00000088775	Gm26037	20		ENSMUSG00000071074	Yipf3	20
ENSMUSG00000087938	Gm26049	20		ENSMUSG00000024072	Yipf4	20
ENSMUSG00000077830	Gm26050	20		ENSMUSG00000024487	Yipf5	20
ENSMUSG00000078300	Gm2606	12		ENSMUSG00000047694	Yipf6	20

ENSMUSG00000065315	Gm26064	20		ENSMUSG00000048967	Yjefn3	20
ENSMUSG00000065195	Gm26071	10		ENSMUSG00000003208	Yju2	20
ENSMUSG00000094165	Gm26073	20		ENSMUSG00000002741	Ykt6	20
ENSMUSG00000088527	Gm26086	20		ENSMUSG00000021244	Ylpm1	9
ENSMUSG00000065906	Gm26107	10		ENSMUSG00000026775	Yme1l1	2
ENSMUSG00000065904	Gm26109	20		ENSMUSG00000046404	Yod1	20
ENSMUSG00000065298	Gm26121	20		ENSMUSG00000022773	Ypel1	18
ENSMUSG00000065127	Gm26127	20		ENSMUSG00000018427	Ypel2	9
ENSMUSG00000065104	Gm26128	10		ENSMUSG00000042675	Ypel3	7
ENSMUSG00000113113	Gm2614	20		ENSMUSG00000034059	Ypel4	5
ENSMUSG00000093040	Gm26141	20		ENSMUSG00000039770	Ypel5	11
ENSMUSG00000065725	Gm26165	10		ENSMUSG00000028889	Yrdc	20
ENSMUSG00000065728	Gm26175	10		ENSMUSG00000035851	Ythdc1	9
ENSMUSG00000065729	Gm26176	10		ENSMUSG00000034653	Ythdc2	20
ENSMUSG00000095068	Gm26189	2		ENSMUSG00000038848	Ythdf1	20
ENSMUSG00000092774	Gm26197	20		ENSMUSG00000040025	Ythdf2	20
ENSMUSG00000064844	Gm26202	10		ENSMUSG00000047213	Ythdf3	20
ENSMUSG00000089209	Gm26215	20		ENSMUSG00000018326	Ywhab	9
ENSMUSG00000064442	Gm26225	10		ENSMUSG00000020849	Ywhae	13
ENSMUSG00000096838	Gm26232	20		ENSMUSG00000051391	Ywhag	19
ENSMUSG00000095616	Gm26244	10		ENSMUSG00000018965	Ywhah	19
ENSMUSG00000117613	Gm2629	20		ENSMUSG00000076432	Ywhaq	1
ENSMUSG00000078006	Gm26301	20		ENSMUSG00000080902	Ywhaq-ps3	4
ENSMUSG00000065824	Gm26315	20		ENSMUSG00000022285	Ywhaz	9
ENSMUSG00000065007	Gm26324	10		ENSMUSG00000021264	Yy1	20
ENSMUSG00000064755	Gm26328	18		ENSMUSG00000049090	Zadh2	20
ENSMUSG00000096328	Gm26337	10		ENSMUSG00000079173	Zan	6
ENSMUSG00000088845	Gm26355	20		ENSMUSG00000026117	Zap70	20
ENSMUSG00000088456	Gm26372	20		ENSMUSG00000056586	Zar1l	20
ENSMUSG00000077426	Gm26387	20		ENSMUSG00000034151	Zbbx	20
ENSMUSG00000065637	Gm26397	10		ENSMUSG00000041995	Zbed3	17
ENSMUSG00000089125	Gm26403	20		ENSMUSG00000034333	Zbed4	19
ENSMUSG00000089129	Gm26407	20		ENSMUSG00000034173	Zbed5	20
ENSMUSG00000088299	Gm26414	20		ENSMUSG00000027514	Zbp1	20
ENSMUSG00000064525	Gm26440	10		ENSMUSG00000033454	Zbtb1	20
ENSMUSG00000093815	Gm26444	10		ENSMUSG00000069114	Zbtb10	1
ENSMUSG00000064389	Gm26446	20		ENSMUSG00000022601	Zbtb11	20
ENSMUSG00000064380	Gm26448	12		ENSMUSG00000102101	Zbtb11os1	20
ENSMUSG00000092805	Gm26461	10		ENSMUSG00000049823	Zbtb12	20
ENSMUSG00000095713	Gm26466	20		ENSMUSG00000049672	Zbtb14	20
ENSMUSG00000065089	Gm26493	10		ENSMUSG00000066687	Zbtb16	20
ENSMUSG00000093994	Gm26498	20		ENSMUSG00000006215	Zbtb17	9
ENSMUSG00000096019	Gm26504	10		ENSMUSG00000063659	Zbtb18	9
ENSMUSG00000097612	Gm26509	20		ENSMUSG00000075327	Zbtb2	20
ENSMUSG00000097157	Gm26512	20		ENSMUSG00000022708	Zbtb20	2
ENSMUSG00000097150	Gm26513	20		ENSMUSG00000046962	Zbtb21	20
ENSMUSG00000097429	Gm26520	20		ENSMUSG00000051390	Zbtb22	20
ENSMUSG00000097296	Gm26532	20		ENSMUSG00000019826	Zbtb24	20
ENSMUSG00000097748	Gm26533	20		ENSMUSG00000056459	Zbtb25	9
ENSMUSG00000097887	Gm26542	1		ENSMUSG00000050714	Zbtb26	16
ENSMUSG00000097884	Gm26543	20		ENSMUSG00000071661	Zbtb3	20
ENSMUSG00000097217	Gm26549	12		ENSMUSG00000048047	Zbtb33	2
ENSMUSG00000097399	Gm26555	20		ENSMUSG00000068966	Zbtb34	20
ENSMUSG00000097396	Gm26556	9		ENSMUSG00000043467	Zbtb37	20

ENSMUSG00000097027	Gm26559	20		ENSMUSG00000040433	Zbtb38	3
ENSMUSG00000097078	Gm26566	9		ENSMUSG00000044617	Zbtb39	20
ENSMUSG00000096953	Gm26571	20		ENSMUSG00000018750	Zbtb4	18
ENSMUSG00000097926	Gm26575	20		ENSMUSG00000060862	Zbtb40	20
ENSMUSG00000097923	Gm26577	19		ENSMUSG00000033964	Zbtb41	3
ENSMUSG00000097476	Gm26583	20		ENSMUSG00000037638	Zbtb42	20
ENSMUSG00000097730	Gm26588	20		ENSMUSG00000026788	Zbtb43	20
ENSMUSG00000097535	Gm26592	20		ENSMUSG00000047412	Zbtb44	3
ENSMUSG00000010529	Gm266	20		ENSMUSG00000049600	Zbtb45	20
ENSMUSG00000097854	Gm26602	20		ENSMUSG00000027583	Zbtb46	20
ENSMUSG00000097079	Gm26604	7		ENSMUSG00000028952	Zbtb48	20
ENSMUSG00000097240	Gm26614	20		ENSMUSG00000029127	Zbtb49	20
ENSMUSG00000097342	Gm26618	20		ENSMUSG00000049657	Zbtb5	20
ENSMUSG00000096968	Gm26620	9		ENSMUSG00000066798	Zbtb6	4
ENSMUSG00000096967	Gm26621	20		ENSMUSG00000035011	Zbtb7a	20
ENSMUSG00000097445	Gm26631	20		ENSMUSG00000028042	Zbtb7b	18
ENSMUSG00000097441	Gm26633	20		ENSMUSG00000044646	Zbtb7c	18
ENSMUSG00000097763	Gm26636	20		ENSMUSG00000028807	Zbtb8a	9
ENSMUSG00000097563	Gm26638	2		ENSMUSG00000048485	Zbtb8b	6
ENSMUSG00000097684	Gm26645	1		ENSMUSG00000057572	Zbtb8os	2
ENSMUSG00000097275	Gm26648	13		ENSMUSG00000079605	Zbtb9	20
ENSMUSG00000097497	Gm26652	9		ENSMUSG00000043542	Zc2hc1a	19
ENSMUSG00000097496	Gm26653	10		ENSMUSG00000045064	Zc2hc1c	20
ENSMUSG00000096934	Gm26658	20		ENSMUSG00000039810	Zc3h10	20
ENSMUSG00000097601	Gm26660	20		ENSMUSG00000102976	Zc3h11a	20
ENSMUSG00000097419	Gm26666	20		ENSMUSG00000042677	Zc3h12a	20
ENSMUSG00000097339	Gm26671	20		ENSMUSG00000035045	Zc3h12b	4
ENSMUSG00000097330	Gm26672	13		ENSMUSG00000035164	Zc3h12c	20
ENSMUSG00000097596	Gm26673	1		ENSMUSG00000022000	Zc3h13	20
ENSMUSG00000097888	Gm26682	20		ENSMUSG00000021012	Zc3h14	20
ENSMUSG00000097286	Gm26684	13		ENSMUSG00000027091	Zc3h15	20
ENSMUSG00000097911	Gm26691	20		ENSMUSG00000017478	Zc3h18	9
ENSMUSG00000097035	Gm26692	1		ENSMUSG00000075600	Zc3h3	20
ENSMUSG00000097030	Gm26693	20		ENSMUSG00000059273	Zc3h4	20
ENSMUSG00000097513	Gm26696	20		ENSMUSG00000042851	Zc3h6	20
ENSMUSG00000097877	Gm26703	6		ENSMUSG00000037965	Zc3h7a	20
ENSMUSG00000101162	Gm26728	20		ENSMUSG00000022390	Zc3h7b	20
ENSMUSG00000097464	Gm26736	12		ENSMUSG00000027387	Zc3h8	20
ENSMUSG00000097467	Gm26737	20		ENSMUSG00000029826	Zc3hav1	12
ENSMUSG00000097705	Gm26740	20		ENSMUSG00000047749	Zc3hav1l	20
ENSMUSG00000097783	Gm26747	9		ENSMUSG00000039130	Zc3hc1	20
ENSMUSG00000097840	Gm26756	4		ENSMUSG00000035062	Zc4h2	20
ENSMUSG00000109055	Gm2676	20		ENSMUSG00000018239	Zcchc10	20
ENSMUSG00000097068	Gm26760	9		ENSMUSG00000036699	Zcchc12	17
ENSMUSG00000097259	Gm26766	20		ENSMUSG00000061410	Zcchc14	9
ENSMUSG00000097250	Gm26771	20		ENSMUSG00000028772	Zcchc17	20
ENSMUSG00000097801	Gm26777	9		ENSMUSG00000031428	Zcchc18	20
ENSMUSG00000097140	Gm26779	20		ENSMUSG00000038866	Zcchc2	20
ENSMUSG00000097433	Gm26781	20		ENSMUSG00000055538	Zcchc24	20
ENSMUSG00000097431	Gm26782	18		ENSMUSG00000074682	Zcchc3	20
ENSMUSG00000096992	Gm26788	20		ENSMUSG00000029179	Zcchc4	20
ENSMUSG00000096999	Gm26793	20		ENSMUSG00000035649	Zcchc7	20
ENSMUSG00000097572	Gm26797	20		ENSMUSG00000029427	Zcchc8	20
ENSMUSG00000097892	Gm26801	20		ENSMUSG00000021621	Zcchc9	20

ENSMUSG00000097265	Gm26803	20		ENSMUSG00000083274	Zcchc9-ps	20
ENSMUSG00000097268	Gm26805	20		ENSMUSG00000022635	Zcrb1	20
ENSMUSG00000097385	Gm26814	9		ENSMUSG00000037108	Zcwpw1	20
ENSMUSG00000097635	Gm26826	20		ENSMUSG00000032443	Zcwpw2	20
ENSMUSG00000110559	Gm26843	12		ENSMUSG00000027520	Zdbf2	1
ENSMUSG00000097311	Gm26871	4		ENSMUSG00000039199	Zdhhc1	13
ENSMUSG00000097372	Gm26876	20		ENSMUSG00000015335	Zdhhc12	20
ENSMUSG00000096974	Gm26881	19		ENSMUSG00000030471	Zdhhc13	9
ENSMUSG00000097125	Gm26885	12		ENSMUSG00000034265	Zdhhc14	20
ENSMUSG00000097797	Gm26901	7		ENSMUSG00000033906	Zdhhc15	20
ENSMUSG00000097558	Gm26902	20		ENSMUSG00000025157	Zdhhc16	20
ENSMUSG00000097835	Gm26910	20		ENSMUSG00000035798	Zdhhc17	7
ENSMUSG00000097834	Gm26911	20		ENSMUSG00000037553	Zdhhc18	20
ENSMUSG00000097971	Gm26917	2		ENSMUSG00000039470	Zdhhc2	20
ENSMUSG00000100819	Gm2693	20		ENSMUSG00000021969	Zdhhc20	20
ENSMUSG00000102142	Gm26930	20		ENSMUSG00000028403	Zdhhc21	20
ENSMUSG00000098146	Gm26935	20		ENSMUSG00000048483	Zdhhc22	18
ENSMUSG00000097248	Gm2694	3		ENSMUSG00000036304	Zdhhc23	1
ENSMUSG00000098066	Gm26944	20		ENSMUSG00000006463	Zdhhc24	20
ENSMUSG00000098055	Gm26947	20		ENSMUSG00000025786	Zdhhc3	12
ENSMUSG00000097983	Gm26971	20		ENSMUSG00000001844	Zdhhc4	20
ENSMUSG00000097994	Gm26982	20		ENSMUSG00000034075	Zdhhc5	3
ENSMUSG00000097961	Gm27000	20		ENSMUSG00000024982	Zdhhc6	20
ENSMUSG00000098024	Gm27003	20		ENSMUSG00000031823	Zdhhc7	10
ENSMUSG00000098108	Gm27008	20		ENSMUSG00000036985	Zdhhc9	20
ENSMUSG00000098183	Gm27010	1		ENSMUSG00000024238	Zeb1	9
ENSMUSG00000097998	Gm27019	20		ENSMUSG00000026872	Zeb2	5
ENSMUSG00000097970	Gm27028	20		ENSMUSG00000052248	Zeb2os	18
ENSMUSG00000097239	Gm27029	20		ENSMUSG00000039686	Zer1	20
ENSMUSG00000098221	Gm27030	20		ENSMUSG00000049576	Zfa-ps	20
ENSMUSG00000098051	Gm27032	12		ENSMUSG00000039795	Zfand1	20
ENSMUSG00000098121	Gm27038	20		ENSMUSG00000053581	Zfand2a	1
ENSMUSG00000098129	Gm27040	20		ENSMUSG00000026197	Zfand2b	20
ENSMUSG00000099190	Gm27188	20		ENSMUSG00000044477	Zfand3	9
ENSMUSG00000103042	Gm2719	20		ENSMUSG00000042213	Zfand4	20
ENSMUSG00000098889	Gm27206	20		ENSMUSG00000024750	Zfand5	20
ENSMUSG00000098985	Gm27219	20		ENSMUSG00000030629	Zfand6	20
ENSMUSG00000099150	Gm27243	19		ENSMUSG00000074578	Zfas1	20
ENSMUSG00000098708	Gm27252	20		ENSMUSG00000022335	Zfat	18
ENSMUSG00000098340	Gm27253	20		ENSMUSG00000034163	Zfc3h1	20
ENSMUSG00000099252	Gm27265	20		ENSMUSG00000040721	Zfhx2	20
ENSMUSG00000098828	Gm27283	20		ENSMUSG00000093452	Zfhx2os	20
ENSMUSG00000099127	Gm27320	1		ENSMUSG00000038872	Zfhx3	18
ENSMUSG00000098589	Gm27353	20		ENSMUSG00000025255	Zfhx4	18
ENSMUSG00000099224	Gm27379	20		ENSMUSG00000055835	Zfp1	20
ENSMUSG00000098827	Gm27399	1		ENSMUSG00000055240	Zfp101	20
ENSMUSG00000098791	Gm27402	20		ENSMUSG00000057895	Zfp105	20
ENSMUSG00000076355	Gm27509	10		ENSMUSG00000027288	Zfp106	20
ENSMUSG00000098259	Gm27616	12		ENSMUSG00000030486	Zfp108	20
ENSMUSG00000107008	Gm2762	20		ENSMUSG00000074283	Zfp109	20
ENSMUSG00000098750	Gm27653	20		ENSMUSG00000051034	Zfp11	13
ENSMUSG00000110318	Gm2767	20		ENSMUSG00000058638	Zfp110	20
ENSMUSG00000098349	Gm27711	2		ENSMUSG00000087598	Zfp111	20
ENSMUSG00000099155	Gm27805	20		ENSMUSG00000052675	Zfp112	20

ENSMUSG00000098674	Gm27813	20		ENSMUSG00000037007	Zfp113	20
ENSMUSG00000098271	Gm27828	20		ENSMUSG00000068962	Zfp114	20
ENSMUSG00000098733	Gm27833	12		ENSMUSG00000057835	Zfp119a	13
ENSMUSG00000098628	Gm27976	20		ENSMUSG00000062101	Zfp119b	20
ENSMUSG00000099041	Gm28035	20		ENSMUSG00000029587	Zfp12	18
ENSMUSG00000098374	Gm28043	20		ENSMUSG00000068134	Zfp120	20
ENSMUSG00000116536	Gm2805	20		ENSMUSG00000069755	Zfp125	20
ENSMUSG00000099061	Gm28050	20		ENSMUSG00000060397	Zfp128	12
ENSMUSG00000100929	Gm28064	20		ENSMUSG00000062012	Zfp13	20
ENSMUSG00000116560	Gm2808	20		ENSMUSG00000094870	Zfp131	20
ENSMUSG00000082938	Gm2810	20		ENSMUSG00000083674	Zfp133-ps	20
ENSMUSG00000099954	Gm28112	20		ENSMUSG00000053985	Zfp14	20
ENSMUSG00000100158	Gm28119	20		ENSMUSG00000092416	Zfp141	20
ENSMUSG00000100213	Gm28151	20		ENSMUSG00000026135	Zfp142	20
ENSMUSG00000100053	Gm28154	20		ENSMUSG00000061079	Zfp143	20
ENSMUSG00000101299	Gm28175	20		ENSMUSG00000037029	Zfp146	20
ENSMUSG00000101596	Gm28182	1		ENSMUSG00000022811	Zfp148	20
ENSMUSG00000099375	Gm28187	20		ENSMUSG00000036898	Zfp157	2
ENSMUSG00000100255	Gm28196	20		ENSMUSG00000067942	Zfp160	1
ENSMUSG00000101335	Gm28229	20		ENSMUSG00000050954	Zfp169	20
ENSMUSG00000097452	Gm2824	19		ENSMUSG00000054939	Zfp174	20
ENSMUSG00000100620	Gm28277	3		ENSMUSG00000057101	Zfp180	19
ENSMUSG00000101014	Gm28289	20		ENSMUSG00000054737	Zfp182	20
ENSMUSG00000101006	Gm28299	20		ENSMUSG00000006720	Zfp184	20
ENSMUSG00000086567	Gm2830	20		ENSMUSG00000031351	Zfp185	13
ENSMUSG00000099924	Gm28320	20		ENSMUSG00000039634	Zfp189	20
ENSMUSG00000100671	Gm28322	9		ENSMUSG00000049321	Zfp2	20
ENSMUSG00000100313	Gm28323	20		ENSMUSG00000025602	Zfp202	20
ENSMUSG00000096751	Gm28373	20		ENSMUSG00000017421	Zfp207	18
ENSMUSG00000100672	Gm28404	16		ENSMUSG00000052763	Zfp212	20
ENSMUSG00000101037	Gm28424	19		ENSMUSG00000071256	Zfp213	20
ENSMUSG00000101111	Gm28437	20		ENSMUSG00000052056	Zfp217	20
ENSMUSG00000101939	Gm28438	12		ENSMUSG00000049295	Zfp219	18
ENSMUSG00000100131	Gm28439	20		ENSMUSG00000061544	Zfp229	9
ENSMUSG00000100395	Gm28448	20		ENSMUSG00000047603	Zfp235	20
ENSMUSG00000100490	Gm28455	20		ENSMUSG00000041258	Zfp236	7
ENSMUSG00000101166	Gm28496	20		ENSMUSG00000042097	Zfp239	20
ENSMUSG00000101662	Gm28499	20		ENSMUSG00000051469	Zfp24	20
ENSMUSG00000100183	Gm28512	20		ENSMUSG00000030145	Zfp248	1
ENSMUSG00000099568	Gm28513	9		ENSMUSG00000022526	Zfp251	1
ENSMUSG00000100707	Gm28523	20		ENSMUSG00000063108	Zfp26	2
ENSMUSG00000099635	Gm28528	18		ENSMUSG00000049421	Zfp260	19
ENSMUSG00000101320	Gm28529	20		ENSMUSG00000022529	Zfp263	8
ENSMUSG00000101452	Gm28530	20		ENSMUSG00000109176	Zfp264	20
ENSMUSG00000100235	Gm28557	20		ENSMUSG00000060510	Zfp266	20
ENSMUSG00000100417	Gm28578	18		ENSMUSG00000033883	Zfp267	5
ENSMUSG00000100176	Gm28586	20		ENSMUSG00000062040	Zfp27	1
ENSMUSG00000100113	Gm28592	20		ENSMUSG00000030446	Zfp273	1
ENSMUSG00000102070	Gm28661	12		ENSMUSG00000031365	Zfp275	18
ENSMUSG00000101964	Gm28693	20		ENSMUSG00000001065	Zfp276	20
ENSMUSG00000099512	Gm28703	18		ENSMUSG00000055917	Zfp277	19
ENSMUSG00000102001	Gm28755	20		ENSMUSG00000062861	Zfp28	20
ENSMUSG00000109887	Gm28756	20		ENSMUSG00000049764	Zfp280b	20
ENSMUSG00000100394	Gm28791	20		ENSMUSG00000036916	Zfp280c	20

ENSMUSG00000099868	Gm28792	20		ENSMUSG00000038535	Zfp280d	7
ENSMUSG00000099760	Gm28800	20		ENSMUSG00000041483	Zfp281	20
ENSMUSG00000099343	Gm28836	20		ENSMUSG00000025821	Zfp282	2
ENSMUSG00000100975	Gm28875	20		ENSMUSG00000047342	Zfp286	20
ENSMUSG00000101168	Gm28892	15		ENSMUSG00000005267	Zfp287	12
ENSMUSG00000101621	Gm28905	1		ENSMUSG00000039967	Zfp292	20
ENSMUSG00000101450	Gm28941	20		ENSMUSG00000011267	Zfp296	20
ENSMUSG00000100039	Gm28959	20		ENSMUSG00000043602	Zfp3	20
ENSMUSG00000079410	Gm2897	20		ENSMUSG00000047473	Zfp30	20
ENSMUSG00000100552	Gm29019	20		ENSMUSG00000031079	Zfp300	20
ENSMUSG00000101587	Gm29036	14		ENSMUSG00000046658	Zfp316	20
ENSMUSG00000101144	Gm29054	20		ENSMUSG00000057551	Zfp317	20
ENSMUSG00000100768	Gm29055	20		ENSMUSG00000015597	Zfp318	1
ENSMUSG00000100811	Gm29083	19		ENSMUSG00000046556	Zfp319	20
ENSMUSG00000100201	Gm29093	20		ENSMUSG00000046351	Zfp322a	20
ENSMUSG00000100815	Gm29112	20		ENSMUSG00000004500	Zfp324	20
ENSMUSG00000100764	Gm29155	20		ENSMUSG00000029290	Zfp326	20
ENSMUSG00000100480	Gm29156	9		ENSMUSG00000057894	Zfp329	20
ENSMUSG00000100455	Gm29170	20		ENSMUSG00000031711	Zfp330	6
ENSMUSG00000101344	Gm29183	20		ENSMUSG00000017667	Zfp334	20
ENSMUSG00000101301	Gm29214	20		ENSMUSG00000039834	Zfp335	20
ENSMUSG00000101249	Gm29216	20		ENSMUSG00000085436	Zfp335os	20
ENSMUSG00000073174	Gm29254	20		ENSMUSG00000059842	Zfp341	20
ENSMUSG00000100228	Gm29257	20		ENSMUSG00000021481	Zfp346	20
ENSMUSG00000099543	Gm29322	20		ENSMUSG00000063281	Zfp35	20
ENSMUSG00000099625	Gm29325	10		ENSMUSG00000020364	Zfp354a	20
ENSMUSG00000099470	Gm29340	20		ENSMUSG00000020335	Zfp354b	20
ENSMUSG00000099931	Gm29358	9		ENSMUSG00000044807	Zfp354c	20
ENSMUSG00000100396	Gm29367	3		ENSMUSG00000047264	Zfp358	20
ENSMUSG00000099839	Gm29374	20		ENSMUSG00000044786	Zfp36	20
ENSMUSG00000101648	Gm29388	20		ENSMUSG00000028799	Zfp362	20
ENSMUSG00000101309	Gm29397	9		ENSMUSG00000037855	Zfp365	9
ENSMUSG00000101823	Gm29438	20		ENSMUSG00000050919	Zfp366	20
ENSMUSG00000101724	Gm29453	20		ENSMUSG00000044934	Zfp367	19
ENSMUSG00000101179	Gm29455	1		ENSMUSG00000021514	Zfp369	20
ENSMUSG00000101995	Gm29480	20		ENSMUSG00000021127	Zfp36l1	20
ENSMUSG00000099959	Gm29487	18		ENSMUSG00000045817	Zfp36l2	20
ENSMUSG00000101778	Gm29488	20		ENSMUSG00000028389	Zfp37	20
ENSMUSG00000100789	Gm29508	20		ENSMUSG00000074220	Zfp382	20
ENSMUSG00000099964	Gm29514	9		ENSMUSG00000099689	Zfp383	20
ENSMUSG00000100475	Gm29543	20		ENSMUSG00000038346	Zfp384	20
ENSMUSG00000101678	Gm29609	20		ENSMUSG00000000552	Zfp385a	18
ENSMUSG00000089838	Gm2962	12		ENSMUSG00000027016	Zfp385b	9
ENSMUSG00000100002	Gm29642	20		ENSMUSG00000014198	Zfp385c	20
ENSMUSG00000099876	Gm29650	20		ENSMUSG00000042063	Zfp386	20
ENSMUSG00000100967	Gm29666	20		ENSMUSG00000082396	Zfp389	20
ENSMUSG00000100794	Gm29667	3		ENSMUSG00000037001	Zfp39	20
ENSMUSG00000099835	Gm29668	9		ENSMUSG00000034522	Zfp395	18
ENSMUSG00000100302	Gm29670	20		ENSMUSG00000024276	Zfp397	20
ENSMUSG00000112466	Gm29674	12		ENSMUSG00000062519	Zfp398	3
ENSMUSG00000113515	Gm29675	18		ENSMUSG00000002617	Zfp40	4
ENSMUSG00000113689	Gm29676	20		ENSMUSG00000048410	Zfp407	7
ENSMUSG00000109097	Gm29683	18		ENSMUSG00000075040	Zfp408	20
ENSMUSG00000118219	Gm29695	20		ENSMUSG00000047003	Zfp41	20

ENSMUSG00000111673	Gm29724	20		ENSMUSG00000042472	Zfp410	9
ENSMUSG00000103334	Gm29771	9		ENSMUSG00000073423	Zfp414	20
ENSMUSG00000103419	Gm29808	20		ENSMUSG00000034538	Zfp418	1
ENSMUSG00000111027	Gm2981	20		ENSMUSG00000058402	Zfp420	5
ENSMUSG00000114256	Gm29844	20		ENSMUSG00000059878	Zfp422	5
ENSMUSG00000105331	Gm29865	19		ENSMUSG00000091515	Zfp422-ps	20
ENSMUSG00000097794	Gm2990	1		ENSMUSG00000045333	Zfp423	18
ENSMUSG00000092454	Gm2991	20		ENSMUSG00000059475	Zfp426	1
ENSMUSG00000115868	Gm2999	20		ENSMUSG00000064264	Zfp428	10
ENSMUSG00000000594	Gm2a	20		ENSMUSG00000078994	Zfp429	20
ENSMUSG00000107318	Gm30003	9		ENSMUSG00000096795	Zfp433	20
ENSMUSG00000112278	Gm30025	1		ENSMUSG00000050945	Zfp438	1
ENSMUSG00000114937	Gm30054	20		ENSMUSG00000068130	Zfp442	20
ENSMUSG00000108513	Gm30075	20		ENSMUSG00000044876	Zfp444	1
ENSMUSG00000112433	Gm30122	20		ENSMUSG00000047036	Zfp445	20
ENSMUSG00000103965	Gm30173	3		ENSMUSG00000033961	Zfp446	20
ENSMUSG00000103898	Gm30238	20		ENSMUSG00000073176	Zfp449	20
ENSMUSG00000111066	Gm30313	20		ENSMUSG00000042197	Zfp451	3
ENSMUSG00000110388	Gm30329	1		ENSMUSG00000048728	Zfp454	9
ENSMUSG00000104411	Gm30340	20		ENSMUSG00000051037	Zfp455	20
ENSMUSG00000116525	Gm30371	20		ENSMUSG00000078995	Zfp456	20
ENSMUSG00000111758	Gm30373	20		ENSMUSG00000055341	Zfp457	20
ENSMUSG00000106515	Gm30382	20		ENSMUSG00000055480	Zfp458	3
ENSMUSG00000109776	Gm30400	20		ENSMUSG00000055560	Zfp459	20
ENSMUSG00000113257	Gm30409	20		ENSMUSG00000051351	Zfp46	9
ENSMUSG00000110260	Gm30504	18		ENSMUSG00000060206	Zfp462	9
ENSMUSG00000101462	Gm3052	1		ENSMUSG00000068551	Zfp467	20
ENSMUSG00000117013	Gm30531	20		ENSMUSG00000043903	Zfp469	20
ENSMUSG00000118369	Gm30541	1		ENSMUSG00000053600	Zfp472	20
ENSMUSG00000094622	Gm3055	20		ENSMUSG00000048012	Zfp473	20
ENSMUSG00000115965	Gm30564	18		ENSMUSG00000044519	Zfp488	1
ENSMUSG00000117980	Gm30571	20		ENSMUSG00000090659	Zfp493	10
ENSMUSG00000113459	Gm30655	20		ENSMUSG00000039081	Zfp503	20
ENSMUSG00000111090	Gm30698	20		ENSMUSG00000044452	Zfp507	12
ENSMUSG00000107859	Gm30731	20		ENSMUSG00000023892	Zfp51	20
ENSMUSG00000113456	Gm30738	16		ENSMUSG00000025470	Zfp511	20
ENSMUSG00000090114	Gm3076	12		ENSMUSG00000062761	Zfp512	20
ENSMUSG00000114697	Gm30806	1		ENSMUSG00000000823	Zfp512b	20
ENSMUSG00000103624	Gm3081	20		ENSMUSG00000043059	Zfp513	1
ENSMUSG00000079076	Gm3086	20		ENSMUSG00000058881	Zfp516	20
ENSMUSG00000109341	Gm30873	12		ENSMUSG00000049164	Zfp518a	20
ENSMUSG00000115637	Gm30970	20		ENSMUSG00000046572	Zfp518b	20
ENSMUSG00000109015	Gm31024	20		ENSMUSG00000051341	Zfp52	3
ENSMUSG00000117674	Gm31087	1		ENSMUSG00000024420	Zfp521	20
ENSMUSG00000114621	Gm31107	9		ENSMUSG00000024220	Zfp523	9
ENSMUSG00000110320	Gm31152	20		ENSMUSG00000051184	Zfp524	20
ENSMUSG00000109695	Gm31166	20		ENSMUSG00000046541	Zfp526	20
ENSMUSG00000113244	Gm31218	20		ENSMUSG00000057409	Zfp53	18
ENSMUSG00000114396	Gm31227	20		ENSMUSG00000042439	Zfp532	20
ENSMUSG00000115610	Gm31251	16		ENSMUSG00000043456	Zfp536	20
ENSMUSG00000115591	Gm31282	9		ENSMUSG00000023882	Zfp54	20
ENSMUSG00000116589	Gm31323	9		ENSMUSG00000078796	Zfp541	9
ENSMUSG00000113986	Gm31333	20		ENSMUSG00000034071	Zfp551	20
ENSMUSG00000118099	Gm31356	19		ENSMUSG00000045598	Zfp553	20

ENSMUSG00000099797	Gm3145	20		ENSMUSG00000074500	Zfp558	20
ENSMUSG00000109632	Gm31479	2		ENSMUSG00000045519	Zfp560	16
ENSMUSG00000113387	Gm31508	20		ENSMUSG00000067424	Zfp563	20
ENSMUSG00000114789	Gm31577	20		ENSMUSG00000078768	Zfp566	20
ENSMUSG00000109602	Gm31597	20		ENSMUSG00000074221	Zfp568	20
ENSMUSG00000085371	Gm3160	20		ENSMUSG00000036036	Zfp57	17
ENSMUSG00000111236	Gm31614	1		ENSMUSG00000045252	Zfp574	20
ENSMUSG00000117358	Gm31645	12		ENSMUSG00000066721	Zfp575	20
ENSMUSG00000114662	Gm31683	9		ENSMUSG00000051550	Zfp579	13
ENSMUSG00000108617	Gm31749	20		ENSMUSG00000071291	Zfp58	20
ENSMUSG00000112925	Gm31763	20		ENSMUSG00000055633	Zfp580	20
ENSMUSG00000112742	Gm31793	20		ENSMUSG00000030443	Zfp583	20
ENSMUSG00000111620	Gm31848	20		ENSMUSG00000078779	Zfp59	20
ENSMUSG00000118074	Gm31907	20		ENSMUSG00000005621	Zfp592	3
ENSMUSG00000112422	Gm31938	20		ENSMUSG00000028840	Zfp593	20
ENSMUSG00000114639	Gm31946	18		ENSMUSG00000057842	Zfp595	20
ENSMUSG00000097388	Gm3200	20		ENSMUSG00000039789	Zfp597	20
ENSMUSG00000110623	Gm32005	20		ENSMUSG00000041130	Zfp598	20
ENSMUSG00000111602	Gm32014	20		ENSMUSG00000062794	Zfp599	20
ENSMUSG00000109118	Gm32031	20		ENSMUSG00000037640	Zfp60	20
ENSMUSG00000113313	Gm32036	1		ENSMUSG00000023284	Zfp605	20
ENSMUSG00000110633	Gm32122	20		ENSMUSG00000030386	Zfp606	20
ENSMUSG00000112945	Gm32151	20		ENSMUSG00000020420	Zfp607a	20
ENSMUSG00000115431	Gm3219	20		ENSMUSG00000057093	Zfp607b	20
ENSMUSG00000103114	Gm32200	20		ENSMUSG00000052713	Zfp608	19
ENSMUSG00000079311	Gm3222	20		ENSMUSG00000040524	Zfp609	20
ENSMUSG00000114271	Gm32224	20		ENSMUSG00000050605	Zfp61	20
ENSMUSG00000114547	Gm3226	9		ENSMUSG00000044676	Zfp612	20
ENSMUSG00000117648	Gm32282	20		ENSMUSG00000066880	Zfp617	20
ENSMUSG00000104011	Gm32391	10		ENSMUSG00000028358	Zfp618	20
ENSMUSG00000112404	Gm32442	20		ENSMUSG00000068959	Zfp619	20
ENSMUSG00000102729	Gm32444	20		ENSMUSG00000046311	Zfp62	9
ENSMUSG00000091275	Gm3248	20		ENSMUSG00000052253	Zfp622	20
ENSMUSG00000107902	Gm32592	20		ENSMUSG00000050846	Zfp623	1
ENSMUSG00000115317	Gm32618	20		ENSMUSG00000030604	Zfp626	2
ENSMUSG00000108393	Gm32633	2		ENSMUSG00000074406	Zfp628	20
ENSMUSG00000108532	Gm32647	9		ENSMUSG00000045639	Zfp629	18
ENSMUSG00000116891	Gm32679	9		ENSMUSG00000059689	Zfp637	10
ENSMUSG00000113528	Gm32699	20		ENSMUSG00000030016	Zfp638	20
ENSMUSG00000109590	Gm32710	20		ENSMUSG00000027667	Zfp639	20
ENSMUSG00000078967	Gm3272	20		ENSMUSG00000027551	Zfp64	20
ENSMUSG00000109958	Gm32786	20		ENSMUSG00000022987	Zfp641	20
ENSMUSG00000112252	Gm32802	20		ENSMUSG00000049606	Zfp644	8
ENSMUSG00000115777	Gm32815	20		ENSMUSG00000049739	Zfp646	20
ENSMUSG00000109857	Gm32817	20		ENSMUSG00000054967	Zfp647	9
ENSMUSG00000112323	Gm32828	20		ENSMUSG00000071281	Zfp65	20
ENSMUSG00000112959	Gm32834	10		ENSMUSG00000013419	Zfp651	20
ENSMUSG00000110480	Gm32842	20		ENSMUSG00000075595	Zfp652	20
ENSMUSG00000109203	Gm32849	20		ENSMUSG00000086191	Zfp652os	20
ENSMUSG00000111915	Gm3285	20		ENSMUSG00000038895	Zfp653	12
ENSMUSG00000110605	Gm32856	20		ENSMUSG00000047141	Zfp654	20
ENSMUSG00000111474	Gm32926	20		ENSMUSG00000007812	Zfp655	1
ENSMUSG00000092090	Gm3294	20		ENSMUSG00000056592	Zfp658	20
ENSMUSG00000108713	Gm33027	20		ENSMUSG00000034800	Zfp661	20

ENSMUSG00000109853	Gm33045	20		ENSMUSG00000079215	Zfp664	20
ENSMUSG00000093751	Gm3307	20		ENSMUSG00000054893	Zfp667	20
ENSMUSG00000110125	Gm33148	20		ENSMUSG00000049728	Zfp668	6
ENSMUSG00000113780	Gm33195	20		ENSMUSG00000049755	Zfp672	20
ENSMUSG00000117604	Gm33228	20		ENSMUSG00000062743	Zfp677	20
ENSMUSG00000113836	Gm3325	20		ENSMUSG00000058291	Zfp68	20
ENSMUSG00000102771	Gm33320	20		ENSMUSG00000019338	Zfp687	20
ENSMUSG00000102969	Gm33366	20		ENSMUSG00000045251	Zfp688	20
ENSMUSG00000117375	Gm33373	20		ENSMUSG00000048921	Zfp689	20
ENSMUSG00000112790	Gm33378	9		ENSMUSG00000064141	Zfp69	20
ENSMUSG00000114446	Gm33524	20		ENSMUSG00000045268	Zfp691	20
ENSMUSG00000104237	Gm33533	9		ENSMUSG00000037243	Zfp692	20
ENSMUSG00000089883	Gm3355	12		ENSMUSG00000050064	Zfp697	5
ENSMUSG00000110205	Gm33594	16		ENSMUSG00000033669	Zfp7	9
ENSMUSG00000101439	Gm336	20		ENSMUSG00000085795	Zfp703	20
ENSMUSG00000085442	Gm3362	12		ENSMUSG00000040209	Zfp704	1
ENSMUSG00000105224	Gm3364	20		ENSMUSG00000062397	Zfp706	1
ENSMUSG00000111594	Gm3365	12		ENSMUSG00000034429	Zfp707	16
ENSMUSG00000104939	Gm33651	19		ENSMUSG00000056019	Zfp709	9
ENSMUSG00000112054	Gm33680	17		ENSMUSG00000048897	Zfp710	18
ENSMUSG00000111147	Gm33699	20		ENSMUSG00000025529	Zfp711	20
ENSMUSG00000102298	Gm33707	20		ENSMUSG00000090641	Zfp712	20
ENSMUSG00000117437	Gm33727	9		ENSMUSG00000012640	Zfp715	1
ENSMUSG00000113625	Gm3379	20		ENSMUSG00000030469	Zfp719	1
ENSMUSG00000111792	Gm33858	20		ENSMUSG00000069184	Zfp72	20
ENSMUSG00000112358	Gm33869	20		ENSMUSG00000021510	Zfp729a	1
ENSMUSG00000109733	Gm33940	20		ENSMUSG00000058093	Zfp729b	9
ENSMUSG00000117473	Gm33948	20		ENSMUSG00000048280	Zfp738	20
ENSMUSG00000111293	Gm34006	20		ENSMUSG00000059975	Zfp74	20
ENSMUSG00000111097	Gm34069	9		ENSMUSG00000046897	Zfp740	20
ENSMUSG00000106883	Gm34091	20		ENSMUSG00000057691	Zfp746	20
ENSMUSG00000079396	Gm3411	20		ENSMUSG00000054381	Zfp747	5
ENSMUSG00000108659	Gm34121	20		ENSMUSG00000095432	Zfp748	20
ENSMUSG00000102922	Gm34176	9		ENSMUSG00000039238	Zfp750	20
ENSMUSG00000114045	Gm34220	20		ENSMUSG00000044501	Zfp758	20
ENSMUSG00000113330	Gm34237	20		ENSMUSG00000057396	Zfp759	6
ENSMUSG00000105542	Gm34248	20		ENSMUSG00000067928	Zfp760	20
ENSMUSG00000116793	Gm34256	20		ENSMUSG00000067430	Zfp763	20
ENSMUSG00000109559	Gm34280	20		ENSMUSG00000045757	Zfp764	20
ENSMUSG00000103662	Gm34294	9		ENSMUSG00000047371	Zfp768	20
ENSMUSG00000112763	Gm34304	20		ENSMUSG00000040321	Zfp770	3
ENSMUSG00000116895	Gm3435	20		ENSMUSG00000054716	Zfp771	20
ENSMUSG00000114854	Gm34354	20		ENSMUSG00000066838	Zfp772	4
ENSMUSG00000096160	Gm3436	20		ENSMUSG00000063535	Zfp773	3
ENSMUSG00000115319	Gm34417	20		ENSMUSG00000007216	Zfp775	20
ENSMUSG00000110653	Gm34418	20		ENSMUSG00000071477	Zfp777	20
ENSMUSG00000114715	Gm34471	20		ENSMUSG00000055150	Zfp78	20
ENSMUSG00000117185	Gm34510	20		ENSMUSG00000063047	Zfp780b	20
ENSMUSG00000113031	Gm34552	20		ENSMUSG00000072653	Zfp783	9
ENSMUSG00000113982	Gm34557	1		ENSMUSG00000043290	Zfp784	20
ENSMUSG00000117254	Gm34567	5		ENSMUSG00000051499	Zfp786	20
ENSMUSG00000112805	Gm34574	20		ENSMUSG00000046792	Zfp787	20
ENSMUSG00000107272	Gm34583	20		ENSMUSG00000074165	Zfp788	20
ENSMUSG00000115816	Gm34589	20		ENSMUSG00000011427	Zfp790	20

ENSMUSG00000115333	Gm34590	20		ENSMUSG00000074194	Zfp791	20
ENSMUSG00000105577	Gm34599	20		ENSMUSG00000095253	Zfp799	20
ENSMUSG00000110863	Gm34655	20		ENSMUSG00000039841	Zfp800	20
ENSMUSG00000114200	Gm34721	1		ENSMUSG00000070866	Zfp804a	18
ENSMUSG00000112168	Gm34776	20		ENSMUSG00000092094	Zfp804b	18
ENSMUSG00000114623	Gm34788	13		ENSMUSG00000074867	Zfp808	20
ENSMUSG00000115762	Gm34907	20		ENSMUSG00000057982	Zfp809	20
ENSMUSG00000115100	Gm34934	9		ENSMUSG00000003929	Zfp81	20
ENSMUSG00000116250	Gm34939	9		ENSMUSG00000066829	Zfp810	10
ENSMUSG00000115326	Gm35019	20		ENSMUSG00000055202	Zfp811	20
ENSMUSG00000109714	Gm35021	20		ENSMUSG00000098022	Zfp82	20
ENSMUSG00000108616	Gm35040	1		ENSMUSG00000069743	Zfp820	18
ENSMUSG00000096936	Gm3510	7		ENSMUSG00000031728	Zfp821	20
ENSMUSG00000105031	Gm3511	10		ENSMUSG00000069208	Zfp825	20
ENSMUSG00000089746	Gm3513	20		ENSMUSG00000071064	Zfp827	2
ENSMUSG00000113121	Gm35161	19		ENSMUSG00000046010	Zfp830	20
ENSMUSG00000110554	Gm35256	20		ENSMUSG00000050600	Zfp831	9
ENSMUSG00000114774	Gm35281	20		ENSMUSG00000021271	Zfp839	1
ENSMUSG00000089782	Gm3531	20		ENSMUSG00000046185	Zfp84	20
ENSMUSG00000109771	Gm35315	20		ENSMUSG00000058192	Zfp846	20
ENSMUSG00000109179	Gm35339	20		ENSMUSG00000058331	Zfp85	1
ENSMUSG00000098192	Gm3534	20		ENSMUSG00000096916	Zfp850	20
ENSMUSG00000105686	Gm35394	9		ENSMUSG00000093910	Zfp853	20
ENSMUSG00000112195	Gm35405	20		ENSMUSG00000044081	Zfp85os	20
ENSMUSG00000118020	Gm35438	20		ENSMUSG00000107476	Zfp862-ps	20
ENSMUSG00000106240	Gm3544	15		ENSMUSG00000074405	Zfp865	20
ENSMUSG00000078240	Gm3550	20		ENSMUSG00000043090	Zfp866	20
ENSMUSG00000111068	Gm35501	20		ENSMUSG00000054519	Zfp867	20
ENSMUSG00000089791	Gm3555	20		ENSMUSG00000060427	Zfp868	1
ENSMUSG00000113702	Gm35558	1		ENSMUSG00000054648	Zfp869	1
ENSMUSG00000113662	Gm35558	20		ENSMUSG00000097333	Zfp87	20
ENSMUSG00000115912	Gm35569	20		ENSMUSG00000095325	Zfp870	6
ENSMUSG00000112721	Gm35608	9		ENSMUSG00000024298	Zfp871	20
ENSMUSG00000113188	Gm35638	20		ENSMUSG00000061371	Zfp873	20
ENSMUSG00000090610	Gm3571	20		ENSMUSG00000069206	Zfp874a	20
ENSMUSG00000113666	Gm35725	20		ENSMUSG00000059839	Zfp874b	1
ENSMUSG00000107851	Gm35736	18		ENSMUSG00000044296	Zfp879	20
ENSMUSG00000113941	Gm35755	9		ENSMUSG00000089857	Zfp882	20
ENSMUSG00000115311	Gm35823	20		ENSMUSG00000072623	Zfp9	20
ENSMUSG00000110862	Gm35835	20		ENSMUSG00000031907	Zfp90	9
ENSMUSG00000109028	Gm35842	18		ENSMUSG00000024695	Zfp91	3
ENSMUSG00000109870	Gm35850	20		ENSMUSG00000031374	Zfp92	1
ENSMUSG00000110532	Gm35857	10		ENSMUSG00000055305	Zfp93	13
ENSMUSG00000117848	Gm35867	20		ENSMUSG00000059897	Zfp930	20
ENSMUSG00000096904	Gm3591	20		ENSMUSG00000078861	Zfp931	20
ENSMUSG00000118380	Gm36037	9		ENSMUSG00000066613	Zfp932	18
ENSMUSG00000094942	Gm3604	9		ENSMUSG00000059423	Zfp933	2
ENSMUSG00000067017	Gm3608	20		ENSMUSG00000074865	Zfp934	20
ENSMUSG00000114810	Gm36101	1		ENSMUSG00000060336	Zfp937	20
ENSMUSG00000092563	Gm3617	20		ENSMUSG00000062931	Zfp938	20
ENSMUSG00000112653	Gm36176	20		ENSMUSG00000030424	Zfp939	1
ENSMUSG00000113774	Gm36236	20		ENSMUSG00000074282	Zfp94	20
ENSMUSG00000106617	Gm36266	20		ENSMUSG00000050855	Zfp940	1
ENSMUSG00000112532	Gm36283	20		ENSMUSG00000060314	Zfp941	6

ENSMUSG00000114172	Gm36298	20		ENSMUSG00000071267	Zfp942	20
ENSMUSG00000114457	Gm36346	20		ENSMUSG00000053347	Zfp943	9
ENSMUSG00000109598	Gm36356	20		ENSMUSG00000033972	Zfp944	20
ENSMUSG00000091754	Gm3636	19		ENSMUSG00000059142	Zfp945	18
ENSMUSG00000109108	Gm36371	20		ENSMUSG00000071266	Zfp946	20
ENSMUSG00000107082	Gm36378	20		ENSMUSG00000063383	Zfp947	2
ENSMUSG00000103961	Gm36388	20		ENSMUSG00000067931	Zfp948	20
ENSMUSG00000114138	Gm36423	13		ENSMUSG00000032425	Zfp949	20
ENSMUSG00000110393	Gm36445	20		ENSMUSG00000074733	Zfp950	20
ENSMUSG00000106692	Gm36447	20		ENSMUSG00000072774	Zfp951	20
ENSMUSG00000117575	Gm36486	20		ENSMUSG00000053390	Zfp952	20
ENSMUSG00000114018	Gm36495	20		ENSMUSG00000098905	Zfp953	20
ENSMUSG00000097891	Gm3650	20		ENSMUSG00000062116	Zfp954	1
ENSMUSG00000113690	Gm36501	9		ENSMUSG00000094441	Zfp955a	11
ENSMUSG00000103238	Gm36527	2		ENSMUSG00000096910	Zfp955b	20
ENSMUSG00000118249	Gm36608	20		ENSMUSG00000045466	Zfp956	20
ENSMUSG00000104214	Gm36638	20		ENSMUSG00000058748	Zfp958	20
ENSMUSG00000102635	Gm36638	20		ENSMUSG00000003198	Zfp959	20
ENSMUSG00000090691	Gm3667	20		ENSMUSG00000096696	Zfp960	20
ENSMUSG00000098232	Gm3671	20		ENSMUSG00000052446	Zfp961	20
ENSMUSG00000110396	Gm36737	1		ENSMUSG00000092260	Zfp963	20
ENSMUSG00000116965	Gm36742	10		ENSMUSG00000091764	Zfp964	16
ENSMUSG00000113088	Gm36757	20		ENSMUSG00000095990	Zfp97	19
ENSMUSG00000105516	Gm36823	20		ENSMUSG00000078866	Zfp970	20
ENSMUSG00000113170	Gm36839	20		ENSMUSG00000074519	Zfp971	20
ENSMUSG00000109981	Gm36849	20		ENSMUSG00000074529	Zfp972	20
ENSMUSG00000111110	Gm36855	20		ENSMUSG00000070709	Zfp974	20
ENSMUSG00000104240	Gm36858	20		ENSMUSG00000069727	Zfp975	20
ENSMUSG00000115138	Gm36899	20		ENSMUSG00000074158	Zfp976	9
ENSMUSG00000112630	Gm36908	20		ENSMUSG00000092335	Zfp977	20
ENSMUSG00000098128	Gm3693	20		ENSMUSG00000066000	Zfp979	20
ENSMUSG00000103778	Gm36930	20		ENSMUSG00000078496	Zfp982	1
ENSMUSG00000103779	Gm36931	20		ENSMUSG00000035868	Zfp983	3
ENSMUSG00000103774	Gm36932	1		ENSMUSG00000078495	Zfp984	17
ENSMUSG00000103772	Gm36933	20		ENSMUSG00000067916	Zfp991	20
ENSMUSG00000102410	Gm36935	1		ENSMUSG00000070605	Zfp992	10
ENSMUSG00000102411	Gm36936	20		ENSMUSG00000096433	Zfp994	20
ENSMUSG00000102417	Gm36939	20		ENSMUSG00000078546	Zfp995	20
ENSMUSG00000102419	Gm36940	20		ENSMUSG00000024792	Zfpl1	20
ENSMUSG00000102160	Gm36944	20		ENSMUSG00000049577	Zfpm1	9
ENSMUSG00000103445	Gm36948	20		ENSMUSG00000022306	Zfpm2	20
ENSMUSG00000104138	Gm36949	20		ENSMUSG00000022201	Zfr	4
ENSMUSG00000067224	Gm3695	9		ENSMUSG00000034949	Zfr2	20
ENSMUSG00000104130	Gm36951	20		ENSMUSG00000079509	Zfx	20
ENSMUSG00000104132	Gm36952	2		ENSMUSG00000042628	Zfyve1	20
ENSMUSG00000104136	Gm36955	20		ENSMUSG00000021706	Zfyve16	20
ENSMUSG00000103931	Gm36962	20		ENSMUSG00000068580	Zfyve19	12
ENSMUSG00000103932	Gm36963	20		ENSMUSG00000021286	Zfyve21	3
ENSMUSG00000103060	Gm36969	20		ENSMUSG00000066440	Zfyve26	12
ENSMUSG00000102494	Gm36988	20		ENSMUSG00000018820	Zfyve27	20
ENSMUSG00000102496	Gm36989	20		ENSMUSG00000037224	Zfyve28	9
ENSMUSG00000094955	Gm3699	12		ENSMUSG00000034557	Zfyve9	19
ENSMUSG00000103436	Gm36995	10		ENSMUSG00000027582	Zgpat	9
ENSMUSG00000102287	Gm37003	20		ENSMUSG00000051278	Zgrf1	1

ENSMUSG00000103884	Gm37005	20		ENSMUSG00000071757	Zhx2	9
ENSMUSG00000103885	Gm37006	9		ENSMUSG00000035877	Zhx3	12
ENSMUSG00000103887	Gm37008	20		ENSMUSG00000032368	Zic1	18
ENSMUSG00000103889	Gm37011	2		ENSMUSG00000061524	Zic2	18
ENSMUSG00000102956	Gm37017	9		ENSMUSG00000067860	Zic3	18
ENSMUSG00000102950	Gm37018	1		ENSMUSG00000036972	Zic4	6
ENSMUSG00000102953	Gm37019	20		ENSMUSG00000041703	Zic5	20
ENSMUSG00000103609	Gm37022	20		ENSMUSG00000030393	Zik1	20
ENSMUSG00000104078	Gm37023	19		ENSMUSG00000002266	Zim1	17
ENSMUSG00000104388	Gm37033	20		ENSMUSG00000029729	Zkscan1	1
ENSMUSG00000102565	Gm37036	20		ENSMUSG00000029627	Zkscan14	20
ENSMUSG00000104221	Gm37051	2		ENSMUSG00000038630	Zkscan16	20
ENSMUSG00000103343	Gm37052	12		ENSMUSG00000020472	Zkscan17	20
ENSMUSG00000103348	Gm37053	20		ENSMUSG00000030757	Zkscan2	20
ENSMUSG00000102753	Gm37056	20		ENSMUSG00000021327	Zkscan3	20
ENSMUSG00000102750	Gm37058	20		ENSMUSG00000054931	Zkscan4	20
ENSMUSG00000103808	Gm37060	20		ENSMUSG00000055991	Zkscan5	20
ENSMUSG00000103809	Gm37061	20		ENSMUSG00000018347	Zkscan6	1
ENSMUSG00000103804	Gm37062	20		ENSMUSG00000063488	Zkscan7	20
ENSMUSG00000103805	Gm37063	19		ENSMUSG00000111063	Zkscan7	20
ENSMUSG00000103803	Gm37064	20		ENSMUSG00000063894	Zkscan8	20
ENSMUSG00000104410	Gm37066	20		ENSMUSG00000052676	Zmat1	20
ENSMUSG00000104416	Gm37067	20		ENSMUSG00000001383	Zmat2	1
ENSMUSG00000104415	Gm37069	1		ENSMUSG00000027663	Zmat3	20
ENSMUSG00000104418	Gm37070	20		ENSMUSG00000037492	Zmat4	18
ENSMUSG00000103685	Gm37074	18		ENSMUSG00000009076	Zmat5	20
ENSMUSG00000102659	Gm37077	20		ENSMUSG00000007817	Zmiz1	20
ENSMUSG00000102653	Gm37079	20		ENSMUSG00000087535	Zmiz1os1	20
ENSMUSG00000102651	Gm37080	20		ENSMUSG00000041164	Zmiz2	20
ENSMUSG00000102850	Gm37082	20		ENSMUSG00000043207	Zmpste24	20
ENSMUSG00000102856	Gm37084	20		ENSMUSG00000043872	Zmym1	20
ENSMUSG00000103183	Gm37090	18		ENSMUSG00000021945	Zmym2	3
ENSMUSG00000102449	Gm37100	6		ENSMUSG00000031310	Zmym3	3
ENSMUSG00000102448	Gm37101	20		ENSMUSG00000042446	Zmym4	20
ENSMUSG00000102672	Gm37105	20		ENSMUSG00000040123	Zmym5	18
ENSMUSG00000102133	Gm37106	20		ENSMUSG00000042408	Zmym6	20
ENSMUSG00000102136	Gm37107	3		ENSMUSG00000010044	Zmynd10	20
ENSMUSG00000102135	Gm37108	12		ENSMUSG00000021156	Zmynd11	4
ENSMUSG00000104494	Gm37111	20		ENSMUSG00000070806	Zmynd12	20
ENSMUSG00000103572	Gm37115	20		ENSMUSG00000040829	Zmynd15	20
ENSMUSG00000103032	Gm37121	4		ENSMUSG00000026974	Zmynd19	1
ENSMUSG00000103039	Gm37123	20		ENSMUSG00000039671	Zmynd8	1
ENSMUSG00000103038	Gm37124	20		ENSMUSG00000084350	Znf41-ps	20
ENSMUSG00000103443	Gm37132	16		ENSMUSG00000039501	Znfx1	19
ENSMUSG00000102874	Gm37137	20		ENSMUSG00000059518	Znhit1	20
ENSMUSG00000104149	Gm37138	20		ENSMUSG00000075227	Znhit2	16
ENSMUSG00000102875	Gm37139	20		ENSMUSG00000020526	Znhit3	12
ENSMUSG00000104140	Gm37140	20		ENSMUSG00000074182	Znhit6	20
ENSMUSG00000102929	Gm37154	20		ENSMUSG00000036315	Znrd1	20
ENSMUSG00000103233	Gm37159	20		ENSMUSG00000036214	Znrd1as	1
ENSMUSG00000103232	Gm37160	20		ENSMUSG00000079478	Znrd2	20
ENSMUSG00000104519	Gm37161	20		ENSMUSG00000033545	Znrf1	9
ENSMUSG00000104512	Gm37165	20		ENSMUSG00000058446	Znrf2	9
ENSMUSG00000102461	Gm37166	20		ENSMUSG00000041961	Znrf3	1

ENSMUSG00000103174	Gm37168	20		ENSMUSG00000042554	Zp3r	20
ENSMUSG00000104253	Gm37174	20		ENSMUSG00000020193	Zpbp	20
ENSMUSG00000103378	Gm37176	9		ENSMUSG00000032078	Zpr1	9
ENSMUSG00000103377	Gm37180	19		ENSMUSG00000030967	Zranb1	20
ENSMUSG00000102764	Gm37183	2		ENSMUSG00000028180	Zranb2	20
ENSMUSG00000103937	Gm37186	20		ENSMUSG00000036086	Zranb3	20
ENSMUSG00000103611	Gm37195	20		ENSMUSG00000044068	Zrsr1	18
ENSMUSG00000103657	Gm37204	2		ENSMUSG00000031370	Zrsr2	20
ENSMUSG00000103651	Gm37206	20		ENSMUSG00000036721	Zscan12	20
ENSMUSG00000104027	Gm37211	7		ENSMUSG00000070822	Zscan18	20
ENSMUSG00000104026	Gm37212	9		ENSMUSG00000038797	Zscan2	20
ENSMUSG00000104020	Gm37215	20		ENSMUSG00000061894	Zscan20	12
ENSMUSG00000102951	Gm37216	20		ENSMUSG00000037017	Zscan21	20
ENSMUSG00000102681	Gm37221	20		ENSMUSG00000054715	Zscan22	9
ENSMUSG00000102533	Gm37226	20		ENSMUSG00000070420	Zscan25	20
ENSMUSG00000102336	Gm37233	9		ENSMUSG00000022228	Zscan26	1
ENSMUSG00000102330	Gm37234	20		ENSMUSG00000050619	Zscan29	20
ENSMUSG00000103373	Gm37238	3		ENSMUSG00000024274	Zscan30	20
ENSMUSG00000106133	Gm3724	20		ENSMUSG00000017764	Zswim1	20
ENSMUSG00000102575	Gm37241	20		ENSMUSG00000045822	Zswim3	20
ENSMUSG00000103630	Gm37242	20		ENSMUSG00000035671	Zswim4	20
ENSMUSG00000102475	Gm37246	20		ENSMUSG00000033948	Zswim5	9
ENSMUSG00000103466	Gm37247	20		ENSMUSG00000032846	Zswim6	9
ENSMUSG00000103216	Gm37248	20		ENSMUSG00000014243	Zswim7	20
ENSMUSG00000103857	Gm37249	20		ENSMUSG00000021819	Zswim8	20
ENSMUSG00000103854	Gm37250	20		ENSMUSG00000070814	Zswim9	2
ENSMUSG00000104394	Gm37254	20		ENSMUSG00000039531	Zup1	20
ENSMUSG00000104449	Gm37255	20		ENSMUSG00000032264	Zw10	20
ENSMUSG00000102760	Gm37258	2		ENSMUSG00000032400	Zwilch	20
ENSMUSG00000103525	Gm37262	20		ENSMUSG00000019923	Zwint	17
ENSMUSG00000102605	Gm37264	20		ENSMUSG00000073060	Zxda	20
ENSMUSG00000102607	Gm37265	9		ENSMUSG00000073062	Zxdb	20
ENSMUSG00000102600	Gm37266	20		ENSMUSG00000034430	Zxdc	2
ENSMUSG00000103952	Gm37268	20		ENSMUSG00000034636	Zyg11b	3
ENSMUSG00000103285	Gm37274	20		ENSMUSG00000029860	Zyx	9
ENSMUSG00000103280	Gm37277	18		ENSMUSG00000055670	Zzef1	20
ENSMUSG00000103751	Gm37283	20		ENSMUSG00000039068	Zzz3	20
ENSMUSG00000103756	Gm37285	20		ENSMUSG00000024165	Jpt2	20
ENSMUSG00000102630	Gm37289	3		ENSMUSG00000097571	Jpx	6
ENSMUSG00000104113	Gm37292	20		ENSMUSG00000046380	Jrk	20
ENSMUSG00000104116	Gm37296	20		ENSMUSG00000079083	Jrkl	3
ENSMUSG00000104114	Gm37297	1		ENSMUSG00000020216	Jsrp1	19
ENSMUSG00000104118	Gm37298	20		ENSMUSG00000027937	Jtb	20
ENSMUSG00000103133	Gm37303	9		ENSMUSG00000052684	Jun	19
ENSMUSG00000103041	Gm37305	20		ENSMUSG00000052837	Junb	20
ENSMUSG00000103046	Gm37309	20		ENSMUSG00000071076	Jund	20
ENSMUSG00000103047	Gm37310	20		ENSMUSG00000087366	Junos	20
ENSMUSG00000103049	Gm37311	20		ENSMUSG00000001552	Jup	20
ENSMUSG00000104094	Gm37314	20		ENSMUSG00000110630	K230015D01I	6
ENSMUSG00000104324	Gm37320	20		ENSMUSG00000061751	Kalrn	9
ENSMUSG00000104325	Gm37321	20		ENSMUSG00000032702	Kank1	9
ENSMUSG00000103202	Gm37326	20		ENSMUSG00000032194	Kank2	20
ENSMUSG00000103200	Gm37328	20		ENSMUSG00000042099	Kank3	20
ENSMUSG00000103201	Gm37329	19		ENSMUSG00000035407	Kank4	18

ENSMUSG00000103209	Gm37331	20		ENSMUSG00000018412	Kansl1	1
ENSMUSG00000104524	Gm37333	20		ENSMUSG00000026004	Kansl1l	20
ENSMUSG00000104525	Gm37334	20		ENSMUSG00000022992	Kansl2	19
ENSMUSG00000102732	Gm37342	20		ENSMUSG00000097530	Kansl2-ps	20
ENSMUSG00000103947	Gm37345	1		ENSMUSG00000010453	Kansl3	20
ENSMUSG00000103593	Gm37352	20		ENSMUSG00000087403	Kantr	20
ENSMUSG00000103596	Gm37354	20		ENSMUSG00000031948	Kars	3
ENSMUSG00000103625	Gm37357	20		ENSMUSG00000027425	Kat14	20
ENSMUSG00000104017	Gm37363	19		ENSMUSG00000020918	Kat2a	20
ENSMUSG00000104010	Gm37366	20		ENSMUSG00000000708	Kat2b	20
ENSMUSG00000102508	Gm37367	20		ENSMUSG00000024926	Kat5	20
ENSMUSG00000102509	Gm37368	20		ENSMUSG00000031540	Kat6a	20
ENSMUSG00000102503	Gm37370	20		ENSMUSG00000021767	Kat6b	9
ENSMUSG00000102349	Gm37376	20		ENSMUSG00000038909	Kat7	1
ENSMUSG00000102831	Gm37382	20		ENSMUSG00000030801	Kat8	20
ENSMUSG00000102837	Gm37383	20		ENSMUSG00000041298	Katnal1	18
ENSMUSG00000091472	Gm3739	1		ENSMUSG00000025420	Katnal2	18
ENSMUSG00000103123	Gm37390	12		ENSMUSG00000031787	Katnb1	3
ENSMUSG00000103122	Gm37391	20		ENSMUSG00000027132	Katnbl1	20
ENSMUSG00000103129	Gm37393	20		ENSMUSG00000025213	Kazald1	1
ENSMUSG00000104205	Gm37396	18		ENSMUSG00000040606	Kazn	9
ENSMUSG00000104204	Gm37397	20		ENSMUSG00000055675	Kbtbd11	9
ENSMUSG00000104200	Gm37399	20		ENSMUSG00000033182	Kbtbd12	20
ENSMUSG00000103324	Gm37402	20		ENSMUSG00000059486	Kbtbd2	12
ENSMUSG00000103497	Gm37407	20		ENSMUSG00000025893	Kbtbd3	20
ENSMUSG00000103495	Gm37409	20		ENSMUSG00000075502	Kbtbd6	5
ENSMUSG00000103493	Gm37411	20		ENSMUSG00000043881	Kbtbd7	2
ENSMUSG00000103865	Gm37416	20		ENSMUSG00000030031	Kbtbd8	20
ENSMUSG00000103869	Gm37420	3		ENSMUSG00000055239	Kcmf1	12
ENSMUSG00000104435	Gm37422	18		ENSMUSG00000047976	Kcna1	20
ENSMUSG00000104436	Gm37423	20		ENSMUSG00000040724	Kcna2	18
ENSMUSG00000102670	Gm37436	20		ENSMUSG00000047959	Kcna3	20
ENSMUSG00000102673	Gm37437	8		ENSMUSG00000042604	Kcna4	9
ENSMUSG00000102581	Gm37443	20		ENSMUSG00000045534	Kcna5	18
ENSMUSG00000103984	Gm37447	20		ENSMUSG00000038077	Kcna6	18
ENSMUSG00000103882	Gm37452	20		ENSMUSG00000027827	Kcnab1	9
ENSMUSG00000104283	Gm37459	20		ENSMUSG00000028931	Kcnab2	20
ENSMUSG00000104282	Gm37460	20		ENSMUSG00000018470	Kcnab3	18
ENSMUSG00000102427	Gm37463	20		ENSMUSG00000086900	Kcnab3os	20
ENSMUSG00000102420	Gm37464	20		ENSMUSG00000050556	Kcnb1	7
ENSMUSG00000102423	Gm37465	20		ENSMUSG00000092083	Kcnb2	12
ENSMUSG00000103972	Gm37466	1		ENSMUSG00000058975	Kcnc1	18
ENSMUSG00000102157	Gm37470	20		ENSMUSG00000035681	Kcnc2	18
ENSMUSG00000102151	Gm37472	20		ENSMUSG00000062785	Kcnc3	18
ENSMUSG00000102153	Gm37474	20		ENSMUSG00000027895	Kcnc4	19
ENSMUSG00000102801	Gm37478	9		ENSMUSG00000009731	Kcnd1	10
ENSMUSG00000102800	Gm37479	20		ENSMUSG00000060882	Kcnd2	9
ENSMUSG00000104121	Gm37485	10		ENSMUSG00000040896	Kcnd3	19
ENSMUSG00000104125	Gm37488	20		ENSMUSG00000074346	Kcnd3os	20
ENSMUSG00000102211	Gm37490	20		ENSMUSG00000090122	Kcne1l	20
ENSMUSG00000108621	Gm37494	20		ENSMUSG00000039672	Kcne2	13
ENSMUSG00000103907	Gm37498	20		ENSMUSG00000047330	Kcne4	18
ENSMUSG00000103901	Gm37499	20		ENSMUSG00000051726	Kcnf1	18
ENSMUSG00000103016	Gm37506	18		ENSMUSG00000074575	Kcng1	18

ENSMUSG00000102456	Gm37508	20		ENSMUSG00000059852	Kcng2	5
ENSMUSG00000104379	Gm37509	20		ENSMUSG00000045053	Kcng3	9
ENSMUSG00000104378	Gm37510	12		ENSMUSG00000045246	Kcng4	18
ENSMUSG00000104374	Gm37517	5		ENSMUSG00000058248	Kcnh1	18
ENSMUSG00000103251	Gm37519	20		ENSMUSG00000038319	Kcnh2	18
ENSMUSG00000103252	Gm37521	1		ENSMUSG00000037579	Kcnh3	9
ENSMUSG00000103783	Gm37522	20		ENSMUSG00000035355	Kcnh4	9
ENSMUSG00000103780	Gm37524	16		ENSMUSG00000034402	Kcnh5	20
ENSMUSG00000103784	Gm37526	20		ENSMUSG00000001901	Kcnh6	20
ENSMUSG00000103693	Gm37529	20		ENSMUSG00000059742	Kcnh7	3
ENSMUSG00000103696	Gm37531	20		ENSMUSG00000035580	Kcnh8	20
ENSMUSG00000103427	Gm37534	20		ENSMUSG00000053519	Kcnip1	18
ENSMUSG00000103422	Gm37536	20		ENSMUSG00000025221	Kcnip2	3
ENSMUSG00000103420	Gm37537	20		ENSMUSG00000079056	Kcnip3	1
ENSMUSG00000102293	Gm37541	20		ENSMUSG00000029088	Kcnip4	18
ENSMUSG00000102296	Gm37543	20		ENSMUSG00000044708	Kcnj10	18
ENSMUSG00000102940	Gm37551	20		ENSMUSG00000096146	Kcnj11	20
ENSMUSG00000104383	Gm37553	20		ENSMUSG00000042529	Kcnj12	18
ENSMUSG00000103094	Gm37558	20		ENSMUSG00000079436	Kcnj13	13
ENSMUSG00000103098	Gm37559	20		ENSMUSG00000058743	Kcnj14	20
ENSMUSG00000091639	Gm3756	20		ENSMUSG00000051497	Kcnj16	9
ENSMUSG00000104041	Gm37562	20		ENSMUSG00000041695	Kcnj2	9
ENSMUSG00000104044	Gm37566	20		ENSMUSG00000026824	Kcnj3	19
ENSMUSG00000102554	Gm37568	20		ENSMUSG00000044216	Kcnj4	9
ENSMUSG00000102556	Gm37569	20		ENSMUSG00000032034	Kcnj5	17
ENSMUSG00000102559	Gm37570	20		ENSMUSG00000043301	Kcnj6	5
ENSMUSG00000102397	Gm37573	20		ENSMUSG00000030247	Kcnj8	20
ENSMUSG00000102391	Gm37578	20		ENSMUSG00000038026	Kcnj9	18
ENSMUSG00000103388	Gm37581	20		ENSMUSG00000033998	Kcnk1	4
ENSMUSG00000103119	Gm37583	20		ENSMUSG00000033854	Kcnk10	19
ENSMUSG00000103115	Gm37584	20		ENSMUSG00000050138	Kcnk12	19
ENSMUSG00000104239	Gm37588	20		ENSMUSG00000045404	Kcnk13	1
ENSMUSG00000104235	Gm37589	7		ENSMUSG00000037624	Kcnk2	9
ENSMUSG00000103358	Gm37593	9		ENSMUSG00000049265	Kcnk3	18
ENSMUSG00000102749	Gm37598	20		ENSMUSG00000024957	Kcnk4	18
ENSMUSG00000102747	Gm37602	2		ENSMUSG00000023243	Kcnk5	20
ENSMUSG00000103851	Gm37606	20		ENSMUSG00000046410	Kcnk6	20
ENSMUSG00000103839	Gm37607	20		ENSMUSG00000036760	Kcnk9	19
ENSMUSG00000103831	Gm37608	20		ENSMUSG00000063142	Kcnma1	19
ENSMUSG00000103832	Gm37611	20		ENSMUSG00000020155	Kcnmb1	20
ENSMUSG00000103835	Gm37612	20		ENSMUSG00000037610	Kcnmb2	16
ENSMUSG00000103834	Gm37613	20		ENSMUSG00000054934	Kcnmb4	20
ENSMUSG00000102714	Gm37618	20		ENSMUSG00000085837	Kcnmb4os2	20
ENSMUSG00000103672	Gm37621	3		ENSMUSG00000002908	Kcnn1	20
ENSMUSG00000102782	Gm37625	20		ENSMUSG00000054477	Kcnn2	9
ENSMUSG00000102319	Gm37626	20		ENSMUSG00000000794	Kcnn3	12
ENSMUSG00000102317	Gm37628	7		ENSMUSG00000101609	Kcnq1ot1	1
ENSMUSG00000102316	Gm37629	10		ENSMUSG00000016346	Kcnq2	9
ENSMUSG00000104197	Gm37632	20		ENSMUSG00000056258	Kcnq3	8
ENSMUSG00000102868	Gm37633	1		ENSMUSG00000028631	Kcnq4	20
ENSMUSG00000102861	Gm37637	20		ENSMUSG00000028033	Kcnq5	9
ENSMUSG00000102863	Gm37639	20		ENSMUSG00000040164	Kcns1	19
ENSMUSG00000097156	Gm3764	20		ENSMUSG00000050963	Kcns2	4
ENSMUSG00000103197	Gm37642	20		ENSMUSG00000043673	Kcns3	18

ENSMUSG00000103194	Gm37643	20		ENSMUSG00000058740	Kcnt1	1
ENSMUSG00000103192	Gm37645	20		ENSMUSG00000052726	Kcnt2	16
ENSMUSG00000102257	Gm37649	20		ENSMUSG00000031576	Kcnu1	20
ENSMUSG00000103734	Gm37651	20		ENSMUSG00000022342	Kcnv1	18
ENSMUSG00000103738	Gm37652	20		ENSMUSG00000059022	Kcp	20
ENSMUSG00000103739	Gm37653	20		ENSMUSG00000036225	Kctd1	9
ENSMUSG00000104467	Gm37660	20		ENSMUSG00000001098	Kctd10	20
ENSMUSG00000104462	Gm37661	10		ENSMUSG00000046731	Kctd11	20
ENSMUSG00000104469	Gm37663	2		ENSMUSG00000098557	Kctd12	1
ENSMUSG00000103546	Gm37666	20		ENSMUSG00000041633	Kctd12b	20
ENSMUSG00000103540	Gm37668	10		ENSMUSG00000030685	Kctd13	3
ENSMUSG00000103543	Gm37669	20		ENSMUSG00000051727	Kctd14	20
ENSMUSG00000103548	Gm37670	20		ENSMUSG00000030499	Kctd15	20
ENSMUSG00000102625	Gm37672	20		ENSMUSG00000051401	Kctd16	7
ENSMUSG00000102623	Gm37673	1		ENSMUSG00000033287	Kctd17	1
ENSMUSG00000102621	Gm37674	20		ENSMUSG00000054770	Kctd18	20
ENSMUSG00000102620	Gm37675	20		ENSMUSG00000016940	Kctd2	12
ENSMUSG00000103976	Gm37677	20		ENSMUSG00000005936	Kctd20	1
ENSMUSG00000103970	Gm37678	20		ENSMUSG00000044952	Kctd21	20
ENSMUSG00000103025	Gm37686	12		ENSMUSG00000026608	Kctd3	1
ENSMUSG00000104347	Gm37689	10		ENSMUSG00000046523	Kctd4	5
ENSMUSG00000104348	Gm37691	20		ENSMUSG00000016946	Kctd5	20
ENSMUSG00000102562	Gm37694	1		ENSMUSG00000021752	Kctd6	20
ENSMUSG00000103473	Gm37696	1		ENSMUSG00000034110	Kctd7	20
ENSMUSG00000104170	Gm37702	20		ENSMUSG00000037653	Kctd8	20
ENSMUSG00000103646	Gm37706	20		ENSMUSG00000034327	Kctd9	18
ENSMUSG00000102796	Gm37711	9		ENSMUSG00000002778	Kdelr1	20
ENSMUSG00000102241	Gm37716	20		ENSMUSG00000079111	Kdelr2	20
ENSMUSG00000102240	Gm37717	20		ENSMUSG00000036940	Kdm1a	20
ENSMUSG00000102243	Gm37718	20		ENSMUSG00000038080	Kdm1b	20
ENSMUSG00000103959	Gm37720	20		ENSMUSG00000054611	Kdm2a	20
ENSMUSG00000102916	Gm37725	4		ENSMUSG00000029475	Kdm2b	1
ENSMUSG00000102919	Gm37726	20		ENSMUSG00000053470	Kdm3a	9
ENSMUSG00000103220	Gm37728	20		ENSMUSG00000038773	Kdm3b	20
ENSMUSG00000103225	Gm37731	1		ENSMUSG00000033326	Kdm4a	20
ENSMUSG00000103227	Gm37733	20		ENSMUSG00000024201	Kdm4b	9
ENSMUSG00000104508	Gm37735	20		ENSMUSG00000028397	Kdm4c	20
ENSMUSG00000104501	Gm37736	20		ENSMUSG00000053914	Kdm4d	1
ENSMUSG00000104503	Gm37738	20		ENSMUSG00000030180	Kdm5a	7
ENSMUSG00000103146	Gm37745	20		ENSMUSG00000042207	Kdm5b	18
ENSMUSG00000104262	Gm37747	20		ENSMUSG00000025332	Kdm5c	20
ENSMUSG00000104266	Gm37748	20		ENSMUSG00000056673	Kdm5d	19
ENSMUSG00000103384	Gm37753	20		ENSMUSG00000037369	Kdm6a	19
ENSMUSG00000103382	Gm37755	20		ENSMUSG00000018476	Kdm6b	8
ENSMUSG00000102719	Gm37760	20		ENSMUSG00000042599	Kdm7a	9
ENSMUSG00000102994	Gm37767	20		ENSMUSG00000030752	Kdm8	20
ENSMUSG00000103642	Gm37769	20		ENSMUSG00000062960	Kdr	20
ENSMUSG00000104039	Gm37772	18		ENSMUSG00000009905	Kdsr	20
ENSMUSG00000104033	Gm37773	4		ENSMUSG00000003308	Keap1	20
ENSMUSG00000104037	Gm37776	20		ENSMUSG00000028060	Khdc4	20
ENSMUSG00000102698	Gm37777	2		ENSMUSG00000028790	Khdrbs1	20
ENSMUSG00000102691	Gm37780	20		ENSMUSG00000026058	Khdrbs2	1
ENSMUSG00000102521	Gm37781	9		ENSMUSG00000022332	Khdrbs3	5
ENSMUSG00000102523	Gm37783	20		ENSMUSG00000029162	Khk	20

ENSMUSG00000102526	Gm37785	20		ENSMUSG00000047153	Khnyn	1
ENSMUSG00000102632	Gm37786	20		ENSMUSG00000007670	Khsrp	9
ENSMUSG00000102326	Gm37788	20		ENSMUSG00000036333	Kidins220	7
ENSMUSG00000102323	Gm37790	1		ENSMUSG00000021375	Kif13a	16
ENSMUSG00000102321	Gm37792	20		ENSMUSG00000060012	Kif13b	16
ENSMUSG00000102813	Gm37795	18		ENSMUSG00000036768	Kif15	20
ENSMUSG00000103303	Gm37802	20		ENSMUSG00000038844	Kif16b	20
ENSMUSG00000103755	Gm37805	20		ENSMUSG00000028758	Kif17	9
ENSMUSG00000103846	Gm37809	20		ENSMUSG00000027115	Kif18a	20
ENSMUSG00000102900	Gm37811	20		ENSMUSG00000010021	Kif19a	20
ENSMUSG00000102816	Gm37814	3		ENSMUSG00000014602	Kif1a	18
ENSMUSG00000104184	Gm37818	3		ENSMUSG00000063077	Kif1b	3
ENSMUSG00000104459	Gm37824	20		ENSMUSG00000036955	Kif1bp	1
ENSMUSG00000104454	Gm37827	20		ENSMUSG00000020821	Kif1c	16
ENSMUSG00000103539	Gm37834	12		ENSMUSG00000003779	Kif20a	20
ENSMUSG00000103536	Gm37837	9		ENSMUSG00000024795	Kif20b	20
ENSMUSG00000102615	Gm37844	20		ENSMUSG00000022629	Kif21a	19
ENSMUSG00000102618	Gm37848	20		ENSMUSG00000041642	Kif21b	6
ENSMUSG00000102619	Gm37849	12		ENSMUSG00000030677	Kif22	20
ENSMUSG00000103761	Gm37859	9		ENSMUSG00000032254	Kif23	20
ENSMUSG00000103763	Gm37860	20		ENSMUSG00000028438	Kif24	20
ENSMUSG00000103762	Gm37861	3		ENSMUSG00000021294	Kif26a	9
ENSMUSG00000102407	Gm37862	7		ENSMUSG00000026494	Kif26b	1
ENSMUSG00000102401	Gm37864	20		ENSMUSG00000060176	Kif27	3
ENSMUSG00000102179	Gm37868	20		ENSMUSG00000021693	Kif2a	9
ENSMUSG00000102172	Gm37873	20		ENSMUSG00000018395	Kif3a	5
ENSMUSG00000104104	Gm37877	20		ENSMUSG00000027475	Kif3b	20
ENSMUSG00000104107	Gm37879	20		ENSMUSG00000020668	Kif3c	18
ENSMUSG00000094392	Gm3788	20		ENSMUSG00000034311	Kif4	20
ENSMUSG00000104100	Gm37880	16		ENSMUSG00000074657	Kif5a	16
ENSMUSG00000102945	Gm37884	20		ENSMUSG00000006740	Kif5b	16
ENSMUSG00000102234	Gm37885	1		ENSMUSG00000026764	Kif5c	1
ENSMUSG00000104271	Gm37891	8		ENSMUSG00000023999	Kif6	20
ENSMUSG00000103928	Gm37893	20		ENSMUSG00000050382	Kif7	20
ENSMUSG00000103926	Gm37894	20		ENSMUSG00000032489	Kif9	13
ENSMUSG00000103924	Gm37895	20		ENSMUSG00000026585	Kifap3	20
ENSMUSG00000103923	Gm37896	20		ENSMUSG00000079553	Kifc1	20
ENSMUSG00000103920	Gm37897	20		ENSMUSG00000004187	Kifc2	20
ENSMUSG00000103000	Gm37900	20		ENSMUSG00000031788	Kifc3	18
ENSMUSG00000103076	Gm37902	20		ENSMUSG00000024301	Kifc5b	20
ENSMUSG00000104315	Gm37906	20		ENSMUSG00000037262	Kin	20
ENSMUSG00000104316	Gm37909	13		ENSMUSG00000041734	Kirrel	9
ENSMUSG00000113423	Gm3791	20		ENSMUSG00000036915	Kirrel2	20
ENSMUSG00000104313	Gm37911	20		ENSMUSG00000032036	Kirrel3	9
ENSMUSG00000103272	Gm37914	20		ENSMUSG00000053889	Kirrel3os	20
ENSMUSG00000102481	Gm37925	9		ENSMUSG00000035773	Kiss1r	20
ENSMUSG00000104362	Gm37928	4		ENSMUSG00000005672	Kit	18
ENSMUSG00000108260	Gm3793	20		ENSMUSG00000019966	Kitl	18
ENSMUSG00000103403	Gm37931	20		ENSMUSG00000074749	Kiz	20
ENSMUSG00000103408	Gm37933	20		ENSMUSG00000058488	Kl	13
ENSMUSG00000102723	Gm37936	20		ENSMUSG00000021288	Klc1	12
ENSMUSG00000103899	Gm37940	2		ENSMUSG00000024862	Klc2	18
ENSMUSG00000103891	Gm37941	20		ENSMUSG00000040714	Klc3	9
ENSMUSG00000102960	Gm37943	19		ENSMUSG00000003546	Klc4	20

ENSMUSG00000102963	Gm37945	20		ENSMUSG00000037465	Klf10	2
ENSMUSG00000103614	Gm37949	20		ENSMUSG00000020653	Klf11	20
ENSMUSG00000104061	Gm37953	20		ENSMUSG00000072294	Klf12	19
ENSMUSG00000104060	Gm37954	20		ENSMUSG00000052040	Klf13	2
ENSMUSG00000104066	Gm37955	20		ENSMUSG00000073209	Klf14	20
ENSMUSG00000104064	Gm37956	20		ENSMUSG00000030087	Klf15	20
ENSMUSG00000104396	Gm37959	9		ENSMUSG00000035397	Klf16	9
ENSMUSG00000104391	Gm37960	20		ENSMUSG00000055148	Klf2	20
ENSMUSG00000104392	Gm37962	3		ENSMUSG00000029178	Klf3	3
ENSMUSG00000104399	Gm37963	1		ENSMUSG00000003032	Klf4	20
ENSMUSG00000104398	Gm37964	7		ENSMUSG00000005148	Klf5	20
ENSMUSG00000102579	Gm37965	20		ENSMUSG00000000078	Klf6	3
ENSMUSG00000102571	Gm37967	20		ENSMUSG00000025959	Klf7	3
ENSMUSG00000102570	Gm37968	1		ENSMUSG00000041649	Klf8	12
ENSMUSG00000102577	Gm37969	20		ENSMUSG00000033863	Klf9	9
ENSMUSG00000102370	Gm37973	20		ENSMUSG00000051890	Klhdc1	20
ENSMUSG00000102376	Gm37975	20		ENSMUSG00000029775	Klhdc10	9
ENSMUSG00000103132	Gm37978	20		ENSMUSG00000020978	Klhdc2	9
ENSMUSG00000103135	Gm37979	20		ENSMUSG00000063576	Klhdc3	19
ENSMUSG00000103136	Gm37980	20		ENSMUSG00000040263	Klhdc4	20
ENSMUSG00000102225	Gm37983	20		ENSMUSG00000078234	Klhdc7a	20
ENSMUSG00000104210	Gm37984	1		ENSMUSG00000091680	Klhdc7b	18
ENSMUSG00000104211	Gm37985	20		ENSMUSG00000042115	Klhdc8a	18
ENSMUSG00000103331	Gm37995	9		ENSMUSG00000032609	Klhdc8b	20
ENSMUSG00000103482	Gm37999	1		ENSMUSG00000045259	Klhdc9	20
ENSMUSG00000114194	Gm3800	20		ENSMUSG00000022076	Klhl1	20
ENSMUSG00000103819	Gm38008	20		ENSMUSG00000048732	Klhl11	3
ENSMUSG00000103818	Gm38009	20		ENSMUSG00000026455	Klhl12	1
ENSMUSG00000104400	Gm38010	20		ENSMUSG00000036782	Klhl13	6
ENSMUSG00000104406	Gm38014	20		ENSMUSG00000042514	Klhl14	1
ENSMUSG00000103697	Gm38020	19		ENSMUSG00000043929	Klhl15	20
ENSMUSG00000102649	Gm38021	20		ENSMUSG00000054792	Klhl18	19
ENSMUSG00000102647	Gm38024	20		ENSMUSG00000031605	Klhl2	9
ENSMUSG00000103992	Gm38029	20		ENSMUSG00000026705	Klhl20	3
ENSMUSG00000103991	Gm38031	20		ENSMUSG00000073700	Klhl21	20
ENSMUSG00000102441	Gm38032	20		ENSMUSG00000022750	Klhl22	20
ENSMUSG00000102844	Gm38034	9		ENSMUSG00000042155	Klhl23	20
ENSMUSG00000102841	Gm38036	20		ENSMUSG00000062901	Klhl24	20
ENSMUSG00000104292	Gm38042	3		ENSMUSG00000055652	Klhl25	12
ENSMUSG00000104293	Gm38043	20		ENSMUSG00000055707	Klhl26	20
ENSMUSG00000102436	Gm38047	20		ENSMUSG00000020948	Klhl28	3
ENSMUSG00000102147	Gm38055	20		ENSMUSG00000020627	Klhl29	18
ENSMUSG00000102145	Gm38056	16		ENSMUSG00000014164	Klhl3	9
ENSMUSG00000102143	Gm38057	20		ENSMUSG00000040387	Klhl32	9
ENSMUSG00000102140	Gm38058	6		ENSMUSG00000090799	Klhl33	20
ENSMUSG00000102148	Gm38059	20		ENSMUSG00000047485	Klhl34	20
ENSMUSG00000104488	Gm38062	20		ENSMUSG00000035298	Klhl35	20
ENSMUSG00000103569	Gm38067	20		ENSMUSG00000031828	Klhl36	20
ENSMUSG00000103561	Gm38069	20		ENSMUSG00000025597	Klhl4	16
ENSMUSG00000103560	Gm38070	20		ENSMUSG00000074001	Klhl40	20
ENSMUSG00000103916	Gm38071	10		ENSMUSG00000075307	Klhl41	20
ENSMUSG00000103914	Gm38073	20		ENSMUSG00000040102	Klhl42	12
ENSMUSG00000103002	Gm38075	20		ENSMUSG00000054920	Klhl5	20
ENSMUSG00000104344	Gm38077	1		ENSMUSG00000043008	Klhl6	10

ENSMUSG00000104363	Gm38081	20		ENSMUSG00000028986	Klhl7	18
ENSMUSG00000104369	Gm38082	20		ENSMUSG00000029312	Klhl8	20
ENSMUSG00000103354	Gm38083	20		ENSMUSG00000070923	Klhl9	6
ENSMUSG00000102517	Gm38085	3		ENSMUSG00000050063	Klk6	20
ENSMUSG00000097935	Gm3809	20		ENSMUSG00000064023	Klk8	1
ENSMUSG00000104157	Gm38101	1		ENSMUSG00000030187	Klra2	9
ENSMUSG00000104155	Gm38103	20		ENSMUSG00000071537	Klrg2	20
ENSMUSG00000104154	Gm38104	20		ENSMUSG00000002028	Kmt2a	20
ENSMUSG00000104153	Gm38105	9		ENSMUSG00000006307	Kmt2b	20
ENSMUSG00000103075	Gm38109	20		ENSMUSG00000038056	Kmt2c	2
ENSMUSG00000102939	Gm38111	20		ENSMUSG00000048154	Kmt2d	20
ENSMUSG00000102930	Gm38115	20		ENSMUSG00000029004	Kmt2e	20
ENSMUSG00000102937	Gm38116	20		ENSMUSG00000049327	Kmt5a	20
ENSMUSG00000103085	Gm38120	1		ENSMUSG00000045098	Kmt5b	2
ENSMUSG00000103082	Gm38124	20		ENSMUSG00000059851	Kmt5c	20
ENSMUSG00000104052	Gm38125	20		ENSMUSG00000066129	Kndc1	18
ENSMUSG00000102383	Gm38142	20		ENSMUSG00000027326	Knl1	20
ENSMUSG00000103163	Gm38146	20		ENSMUSG00000030980	Knop1	20
ENSMUSG00000103162	Gm38147	20		ENSMUSG00000027331	Knstrn	20
ENSMUSG00000103161	Gm38148	20		ENSMUSG00000029414	Kntc1	20
ENSMUSG00000118088	Gm3815	20		ENSMUSG00000022905	Kpna1	2
ENSMUSG00000103360	Gm38156	20		ENSMUSG00000018362	Kpna2	20
ENSMUSG00000103364	Gm38157	20		ENSMUSG00000021929	Kpna3	4
ENSMUSG00000103367	Gm38158	12		ENSMUSG00000027782	Kpna4	13
ENSMUSG00000102776	Gm38162	3		ENSMUSG00000003731	Kpna6	2
ENSMUSG00000102770	Gm38163	20		ENSMUSG00000038770	Kpna7	20
ENSMUSG00000102192	Gm38167	20		ENSMUSG00000001440	Kpnb1	12
ENSMUSG00000102194	Gm38169	20		ENSMUSG00000006021	Kptn	9
ENSMUSG00000103824	Gm38177	20		ENSMUSG00000030265	Kras	3
ENSMUSG00000103825	Gm38178	20		ENSMUSG00000042810	Krba1	20
ENSMUSG00000102403	Gm38187	20		ENSMUSG00000053012	Krcc1	20
ENSMUSG00000102302	Gm38190	12		ENSMUSG00000020393	Kremen1	9
ENSMUSG00000102305	Gm38192	20		ENSMUSG00000040680	Kremen2	20
ENSMUSG00000102460	Gm38197	20		ENSMUSG00000035047	Kri1	20
ENSMUSG00000102465	Gm38198	7		ENSMUSG00000000600	Krit1	1
ENSMUSG00000102466	Gm38200	20		ENSMUSG00000063334	Krr1	20
ENSMUSG00000103967	Gm38214	20		ENSMUSG00000046834	Krt1	20
ENSMUSG00000103552	Gm38217	20		ENSMUSG00000019761	Krt10	9
ENSMUSG00000103558	Gm38220	5		ENSMUSG00000020912	Krt12	20
ENSMUSG00000102639	Gm38223	9		ENSMUSG00000023043	Krt18	13
ENSMUSG00000102633	Gm38224	20		ENSMUSG00000035849	Krt222	6
ENSMUSG00000102634	Gm38225	20		ENSMUSG00000075570	Krt26	20
ENSMUSG00000103945	Gm38228	20		ENSMUSG00000067594	Krt77	3
ENSMUSG00000102259	Gm38230	20		ENSMUSG00000049382	Krt8	13
ENSMUSG00000103291	Gm38235	12		ENSMUSG00000051617	Krt9	9
ENSMUSG00000103747	Gm38236	20		ENSMUSG00000112223	Krtap10-10	20
ENSMUSG00000103742	Gm38240	9		ENSMUSG00000069583	Krtap12-1	20
ENSMUSG00000103741	Gm38241	12		ENSMUSG00000042747	Krtcap2	20
ENSMUSG00000103748	Gm38243	9		ENSMUSG00000029149	Krtcap3	20
ENSMUSG00000103467	Gm38245	20		ENSMUSG00000018334	Ksr1	5
ENSMUSG00000104164	Gm38248	20		ENSMUSG00000061578	Ksr2	20
ENSMUSG00000104168	Gm38250	20		ENSMUSG00000073775	Kti12	20
ENSMUSG00000102786	Gm38252	9		ENSMUSG00000021843	Ktn1	20
ENSMUSG00000102780	Gm38253	20		ENSMUSG00000055553	Kxd1	20

ENSMUSG00000102783	Gm38254	1		ENSMUSG00000035606	Ky	20
ENSMUSG00000102788	Gm38255	1		ENSMUSG00000039648	Kyat1	20
ENSMUSG00000102258	Gm38257	5		ENSMUSG00000040213	Kyat3	1
ENSMUSG00000102253	Gm38259	20		ENSMUSG00000031391	L1cam	18
ENSMUSG00000102250	Gm38260	7		ENSMUSG00000020988	L2hgdh	18
ENSMUSG00000102733	Gm38262	20		ENSMUSG00000019718	L3hypdh	20
ENSMUSG00000102902	Gm38263	9		ENSMUSG00000035576	L3mbtl1	11
ENSMUSG00000103050	Gm38273	20		ENSMUSG00000022394	L3mbtl2	20
ENSMUSG00000083808	Gm3828	20		ENSMUSG00000039089	L3mbtl3	9
ENSMUSG00000103214	Gm38286	9		ENSMUSG00000041565	L3mbtl4	20
ENSMUSG00000103151	Gm38292	20		ENSMUSG00000044350	Lacc1	20
ENSMUSG00000104272	Gm38297	20		ENSMUSG00000032370	Lactb	20
ENSMUSG00000104277	Gm38299	20		ENSMUSG00000025937	Lactb2	1
ENSMUSG00000103390	Gm38301	20		ENSMUSG00000070683	Lactbl1	20
ENSMUSG00000103391	Gm38302	20		ENSMUSG00000041782	Lad1	20
ENSMUSG00000102709	Gm38308	19		ENSMUSG00000030124	Lag3	20
ENSMUSG00000103732	Gm38315	20		ENSMUSG00000015289	Lage3	20
ENSMUSG00000103735	Gm38317	20		ENSMUSG00000055541	Lair1	9
ENSMUSG00000102989	Gm38318	20		ENSMUSG00000032796	Lama1	20
ENSMUSG00000102982	Gm38319	20		ENSMUSG00000019899	Lama2	2
ENSMUSG00000103632	Gm38329	20		ENSMUSG00000024421	Lama3	20
ENSMUSG00000103636	Gm38331	9		ENSMUSG00000019846	Lama4	20
ENSMUSG00000104002	Gm38336	20		ENSMUSG00000015647	Lama5	13
ENSMUSG00000102516	Gm38340	20		ENSMUSG00000002900	Lamb1	20
ENSMUSG00000102452	Gm38344	20		ENSMUSG00000052911	Lamb2	13
ENSMUSG00000102352	Gm38346	20		ENSMUSG00000026639	Lamb3	20
ENSMUSG00000102357	Gm38348	3		ENSMUSG00000026478	Lamc1	7
ENSMUSG00000102459	Gm38352	20		ENSMUSG00000026479	Lamc2	1
ENSMUSG00000102820	Gm38353	1		ENSMUSG00000026840	Lamc3	20
ENSMUSG00000102935	Gm38355	20		ENSMUSG00000031447	Lamp1	16
ENSMUSG00000103313	Gm38357	20		ENSMUSG00000016534	Lamp2	20
ENSMUSG00000103317	Gm38359	20		ENSMUSG00000027270	Lamp5	18
ENSMUSG00000103591	Gm38365	20		ENSMUSG00000030842	Lamtor1	20
ENSMUSG00000103621	Gm38366	20		ENSMUSG00000028062	Lamtor2	20
ENSMUSG00000103509	Gm38372	20		ENSMUSG00000091512	Lamtor3	20
ENSMUSG00000103505	Gm38374	2		ENSMUSG00000050552	Lamtor4	20
ENSMUSG00000103507	Gm38375	20		ENSMUSG00000087260	Lamtor5	12
ENSMUSG00000103506	Gm38376	20		ENSMUSG00000026000	Lancl1	19
ENSMUSG00000102662	Gm38377	20		ENSMUSG00000062190	Lancl2	3
ENSMUSG00000102664	Gm38380	20		ENSMUSG00000047344	Lancl3	18
ENSMUSG00000102594	Gm38381	20		ENSMUSG00000039682	Lap3	16
ENSMUSG00000102374	Gm38387	9		ENSMUSG00000020585	Laptm4a	20
ENSMUSG00000103413	Gm38389	9		ENSMUSG00000022257	Laptm4b	9
ENSMUSG00000103622	Gm38391	20		ENSMUSG00000028581	Laptm5	20
ENSMUSG00000000948	Gm38393	3		ENSMUSG00000004383	Large1	5
ENSMUSG00000094410	Gm38394	20		ENSMUSG00000040434	Large2	13
ENSMUSG00000105218	Gm38413	9		ENSMUSG00000037331	Larp1	9
ENSMUSG00000108917	Gm38451	3		ENSMUSG00000025762	Larp1b	3
ENSMUSG00000109398	Gm3854	20		ENSMUSG00000023025	Larp4	12
ENSMUSG00000105489	Gm38562	20		ENSMUSG00000033499	Larp4b	12
ENSMUSG00000111752	Gm38575	20		ENSMUSG00000034839	Larp6	20
ENSMUSG00000118075	Gm38576	20		ENSMUSG00000027968	Larp7	4
ENSMUSG00000114401	Gm38604	20		ENSMUSG00000024493	Lars	1
ENSMUSG00000111219	Gm38642	20		ENSMUSG00000035202	Lars2	20

ENSMUSG00000111360	Gm38642	20		ENSMUSG00000057421	Las1l	20
ENSMUSG00000108010	Gm38708	20		ENSMUSG00000038366	Lasp1	19
ENSMUSG00000116093	Gm3888	12		ENSMUSG00000030742	Lat	20
ENSMUSG00000107728	Gm38910	20		ENSMUSG00000040751	Lat2	10
ENSMUSG00000109549	Gm38941	9		ENSMUSG00000021959	Lats2	20
ENSMUSG00000109080	Gm38944	20		ENSMUSG00000060594	Layn	20
ENSMUSG00000108390	Gm39038	20		ENSMUSG00000024063	Lbh	18
ENSMUSG00000108460	Gm39041	2		ENSMUSG00000096740	Lbhd1	3
ENSMUSG00000109450	Gm39043	9		ENSMUSG00000016024	Lbp	13
ENSMUSG00000108801	Gm39090	20		ENSMUSG00000004880	Lbr	19
ENSMUSG00000109277	Gm39121	20		ENSMUSG00000025216	Lbx1	1
ENSMUSG00000109868	Gm39244	1		ENSMUSG00000032258	Lca5	1
ENSMUSG00000111229	Gm39323	20		ENSMUSG00000045275	Lca5l	13
ENSMUSG00000110773	Gm39326	1		ENSMUSG00000035237	Lcat	18
ENSMUSG00000110760	Gm39456	3		ENSMUSG00000000409	Lck	12
ENSMUSG00000111873	Gm39463	20		ENSMUSG00000054469	Lclat1	20
ENSMUSG00000113845	Gm39473	20		ENSMUSG00000030763	Lcmt1	9
ENSMUSG00000109459	Gm39526	20		ENSMUSG00000074890	Lcmt2	20
ENSMUSG00000105003	Gm40055	9		ENSMUSG00000026822	Lcn2	20
ENSMUSG00000105355	Gm40117	20		ENSMUSG00000025019	Lcor	2
ENSMUSG00000117662	Gm4013	20		ENSMUSG00000015882	Lcorl	20
ENSMUSG00000101059	Gm4017	20		ENSMUSG00000021998	Lcp1	20
ENSMUSG00000107362	Gm40309	20		ENSMUSG00000002699	Lcp2	20
ENSMUSG00000104873	Gm40332	20		ENSMUSG00000032401	Lctl	20
ENSMUSG00000110649	Gm40466	20		ENSMUSG00000037669	Ldah	20
ENSMUSG00000110236	Gm40493	20		ENSMUSG00000025223	Ldb1	3
ENSMUSG00000110924	Gm40518	20		ENSMUSG00000039706	Ldb2	20
ENSMUSG00000113362	Gm40557	20		ENSMUSG00000021798	Ldb3	16
ENSMUSG00000113029	Gm40578	20		ENSMUSG00000063229	Ldha	18
ENSMUSG00000112352	Gm40617	20		ENSMUSG00000083836	Ldha-ps2	1
ENSMUSG00000114017	Gm40662	20		ENSMUSG00000101959	Ldhal6b	20
ENSMUSG00000113241	Gm40663	1		ENSMUSG00000030246	Ldhb	2
ENSMUSG00000096449	Gm4076	16		ENSMUSG00000031958	Ldhd	18
ENSMUSG00000113216	Gm40841	9		ENSMUSG00000032193	Ldlr	9
ENSMUSG00000118002	Gm4107	20		ENSMUSG00000048058	Ldlrad3	18
ENSMUSG00000114369	Gm41077	20		ENSMUSG00000024544	Ldlrad4	9
ENSMUSG00000114815	Gm41118	20		ENSMUSG00000037295	Ldlrap1	15
ENSMUSG00000089940	Gm4117	13		ENSMUSG00000057615	Ldoc1	20
ENSMUSG00000114902	Gm4118	20		ENSMUSG00000027985	Lef1	18
ENSMUSG00000114833	Gm41192	20		ENSMUSG00000106086	Lef1os1	18
ENSMUSG00000115580	Gm41230	20		ENSMUSG00000074579	Lekr1	20
ENSMUSG00000114579	Gm4130	20		ENSMUSG00000079330	Lemd1	20
ENSMUSG00000116288	Gm41349	20		ENSMUSG00000044857	Lemd2	20
ENSMUSG00000116125	Gm41361	20		ENSMUSG00000048661	Lemd3	20
ENSMUSG00000116223	Gm41396	20		ENSMUSG00000078813	Leng1	20
ENSMUSG00000116029	Gm41414	18		ENSMUSG00000035545	Leng8	20
ENSMUSG00000074800	Gm4149	12		ENSMUSG00000043432	Leng9	20
ENSMUSG00000116763	Gm41492	20		ENSMUSG00000042487	Leo1	20
ENSMUSG00000117007	Gm41556	20		ENSMUSG00000057722	Lepr	13
ENSMUSG00000117548	Gm41662	12		ENSMUSG00000035212	Leprot	20
ENSMUSG00000117519	Gm41664	20		ENSMUSG00000031513	Leprotl1	3
ENSMUSG00000117992	Gm41760	1		ENSMUSG00000005299	Letm1	20
ENSMUSG00000117526	Gm41780	20		ENSMUSG00000037363	Letm2	20
ENSMUSG00000118280	Gm41804	20		ENSMUSG00000037353	Letmd1	13

ENSMUSG00000092384	Gm4189	20		ENSMUSG00000029570	Lfng	20
ENSMUSG00000091421	Gm4202	20		ENSMUSG00000068220	Lgals1	13
ENSMUSG00000079139	Gm4204	18		ENSMUSG00000050335	Lgals3	20
ENSMUSG00000110631	Gm42047	20		ENSMUSG00000033880	Lgals3bp	20
ENSMUSG00000110156	Gm42067	20		ENSMUSG00000053964	Lgals4	20
ENSMUSG00000097146	Gm4211	18		ENSMUSG00000057554	Lgals8	20
ENSMUSG00000097954	Gm4217	20		ENSMUSG00000001123	Lgals9	20
ENSMUSG00000091378	Gm4219	20		ENSMUSG00000042363	Lgalsl	20
ENSMUSG00000096948	Gm4221	1		ENSMUSG00000067242	Lgi1	4
ENSMUSG00000110373	Gm42303	20		ENSMUSG00000039252	Lgi2	16
ENSMUSG00000108348	Gm42372	9		ENSMUSG00000033595	Lgi3	18
ENSMUSG00000105255	Gm42413	20		ENSMUSG00000036560	Lgi4	20
ENSMUSG00000098178	Gm42418	9		ENSMUSG00000021190	Lgmn	9
ENSMUSG00000104633	Gm42421	20		ENSMUSG00000050199	Lgr4	19
ENSMUSG00000105335	Gm42423	20		ENSMUSG00000020140	Lgr5	20
ENSMUSG00000105962	Gm42432	20		ENSMUSG00000042793	Lgr6	9
ENSMUSG00000105230	Gm42433	20		ENSMUSG00000024107	Lhcgr	20
ENSMUSG00000105517	Gm42436	20		ENSMUSG00000048332	Lhfp	18
ENSMUSG00000106048	Gm42444	20		ENSMUSG00000045312	Lhfpl2	9
ENSMUSG00000106411	Gm42445	20		ENSMUSG00000106379	Lhfpl3	18
ENSMUSG00000106491	Gm42446	1		ENSMUSG00000042873	Lhfpl4	12
ENSMUSG00000105485	Gm42447	20		ENSMUSG00000062252	Lhfpl5	20
ENSMUSG00000104625	Gm42448	20		ENSMUSG00000030946	Lhpp	20
ENSMUSG00000104612	Gm42449	19		ENSMUSG00000018698	Lhx1	18
ENSMUSG00000106637	Gm42455	20		ENSMUSG00000087211	Lhx1os	9
ENSMUSG00000105736	Gm42456	9		ENSMUSG00000000247	Lhx2	9
ENSMUSG00000104728	Gm42462	20		ENSMUSG00000029595	Lhx5	9
ENSMUSG00000106032	Gm42463	20		ENSMUSG00000026890	Lhx6	3
ENSMUSG00000107136	Gm42466	20		ENSMUSG00000096225	Lhx8	19
ENSMUSG00000106751	Gm42467	20		ENSMUSG00000019230	Lhx9	19
ENSMUSG00000110124	Gm4247	1		ENSMUSG00000029199	Lias	20
ENSMUSG00000106337	Gm42478	20		ENSMUSG00000034394	Lif	20
ENSMUSG00000105877	Gm42479	20		ENSMUSG00000054263	Lifr	1
ENSMUSG00000105051	Gm4248	20		ENSMUSG00000056394	Lig1	7
ENSMUSG00000105637	Gm42480	20		ENSMUSG00000020697	Lig3	20
ENSMUSG00000104821	Gm42481	20		ENSMUSG00000049717	Lig4	20
ENSMUSG00000105050	Gm42482	20		ENSMUSG00000070873	Lilra5	9
ENSMUSG00000106646	Gm42483	20		ENSMUSG00000112148	Lilrb4a	20
ENSMUSG00000104709	Gm42484	20		ENSMUSG00000023022	Lima1	9
ENSMUSG00000105049	Gm42486	20		ENSMUSG00000037736	Limch1	17
ENSMUSG00000104833	Gm42489	20		ENSMUSG00000025239	Limd1	20
ENSMUSG00000106265	Gm42490	20		ENSMUSG00000040699	Limd2	9
ENSMUSG00000106275	Gm42495	20		ENSMUSG00000090077	Lime1	20
ENSMUSG00000105772	Gm42496	3		ENSMUSG00000029674	Limk1	18
ENSMUSG00000104581	Gm42497	20		ENSMUSG00000020451	Limk2	9
ENSMUSG00000104727	Gm42500	20		ENSMUSG00000019920	Lims1	20
ENSMUSG00000104708	Gm42503	20		ENSMUSG00000024395	Lims2	20
ENSMUSG00000106934	Gm42506	20		ENSMUSG00000063804	Lin28b	20
ENSMUSG00000104860	Gm42510	20		ENSMUSG00000036845	Lin37	20
ENSMUSG00000105553	Gm42514	20		ENSMUSG00000085793	Lin52	20
ENSMUSG00000105378	Gm42515	20		ENSMUSG00000035310	Lin54	7
ENSMUSG00000105867	Gm42517	20		ENSMUSG00000019906	Lin7a	20
ENSMUSG00000106304	Gm42518	20		ENSMUSG00000003872	Lin7b	3
ENSMUSG00000105745	Gm42521	20		ENSMUSG00000027162	Lin7c	9

ENSMUSG00000105253	Gm42522	20		ENSMUSG00000058729	Lin9	12
ENSMUSG00000105969	Gm42525	9		ENSMUSG00000049556	Lingo1	19
ENSMUSG00000105592	Gm42527	6		ENSMUSG00000045083	Lingo2	9
ENSMUSG00000107134	Gm42528	9		ENSMUSG00000051067	Lingo3	9
ENSMUSG00000107178	Gm42531	19		ENSMUSG00000044505	Lingo4	9
ENSMUSG00000105502	Gm42536	20		ENSMUSG00000053091	Lins1	20
ENSMUSG00000105184	Gm42537	20		ENSMUSG00000024781	Lipa	20
ENSMUSG00000104622	Gm42541	20		ENSMUSG00000003123	Lipe	9
ENSMUSG00000104869	Gm42544	18		ENSMUSG00000053846	Lipg	20
ENSMUSG00000106990	Gm42547	20		ENSMUSG00000044626	Liph	20
ENSMUSG00000106959	Gm42548	20		ENSMUSG00000087303	Lipo2	1
ENSMUSG00000107320	Gm42549	20		ENSMUSG00000024766	Lipo3	1
ENSMUSG00000107199	Gm42550	7		ENSMUSG00000037216	Lipt1	1
ENSMUSG00000105374	Gm42551	20		ENSMUSG00000030725	Lipt2	20
ENSMUSG00000105087	Gm42552	20		ENSMUSG00000022500	Litaf	16
ENSMUSG00000107227	Gm42559	20		ENSMUSG00000047786	Lix1	17
ENSMUSG00000107246	Gm42560	1		ENSMUSG00000049288	Lix1l	1
ENSMUSG00000103703	Gm42568	20		ENSMUSG00000020536	Llgl1	1
ENSMUSG00000107109	Gm42571	20		ENSMUSG00000020782	Llgl2	20
ENSMUSG00000107171	Gm42572	16		ENSMUSG00000020224	Llph	12
ENSMUSG00000107205	Gm42576	1		ENSMUSG00000083364	Llph-ps2	20
ENSMUSG00000107306	Gm42577	20		ENSMUSG00000041891	Lman1	20
ENSMUSG00000098243	Gm4258	20		ENSMUSG00000021484	Lman2	20
ENSMUSG00000106826	Gm42583	9		ENSMUSG00000001143	Lman2l	20
ENSMUSG00000107097	Gm42584	1		ENSMUSG00000010721	Lmbr1	20
ENSMUSG00000105161	Gm42595	2		ENSMUSG00000022999	Lmbr1l	20
ENSMUSG00000105980	Gm42597	6		ENSMUSG00000073725	Lmbrd1	20
ENSMUSG00000105711	Gm42598	20		ENSMUSG00000057604	Lmcd1	20
ENSMUSG00000107278	Gm42600	20		ENSMUSG00000002279	Lmf1	9
ENSMUSG00000104698	Gm42602	20		ENSMUSG00000022614	Lmf2	20
ENSMUSG00000105356	Gm42603	20		ENSMUSG00000022802	Lmln	20
ENSMUSG00000106354	Gm42607	9		ENSMUSG00000028063	Lmna	9
ENSMUSG00000106651	Gm42608	8		ENSMUSG00000024590	Lmnb1	20
ENSMUSG00000105643	Gm42611	10		ENSMUSG00000062075	Lmnb2	20
ENSMUSG00000105442	Gm42614	20		ENSMUSG00000054966	Lmntd1	18
ENSMUSG00000105622	Gm42615	20		ENSMUSG00000036111	Lmo1	20
ENSMUSG00000106010	Gm42616	20		ENSMUSG00000032698	Lmo2	9
ENSMUSG00000104919	Gm42617	20		ENSMUSG00000030226	Lmo3	18
ENSMUSG00000106832	Gm42632	20		ENSMUSG00000028266	Lmo4	18
ENSMUSG00000106458	Gm42633	20		ENSMUSG00000033060	Lmo7	9
ENSMUSG00000105940	Gm42635	20		ENSMUSG00000048096	Lmod1	20
ENSMUSG00000104682	Gm42636	20		ENSMUSG00000038970	Lmtk2	9
ENSMUSG00000104871	Gm42639	20		ENSMUSG00000062044	Lmtk3	20
ENSMUSG00000105431	Gm42640	20		ENSMUSG00000026686	Lmx1a	13
ENSMUSG00000106682	Gm42648	20		ENSMUSG00000038765	Lmx1b	1
ENSMUSG00000104877	Gm42651	9		ENSMUSG00000044471	Lncpint	20
ENSMUSG00000106841	Gm42656	20		ENSMUSG00000116305	Lncppara	2
ENSMUSG00000106251	Gm42658	20		ENSMUSG00000109588	Lnp1	20
ENSMUSG00000105655	Gm42659	1		ENSMUSG00000023845	Lnpep	3
ENSMUSG00000106099	Gm42664	20		ENSMUSG00000009207	Lnpk	12
ENSMUSG00000106631	Gm42669	20		ENSMUSG00000029228	Lnx1	18
ENSMUSG00000105395	Gm42672	20		ENSMUSG00000016520	Lnx2	20
ENSMUSG00000104626	Gm42675	20		ENSMUSG00000041168	Lonp1	20
ENSMUSG00000105041	Gm42676	20		ENSMUSG00000047866	Lonp2	20

ENSMUSG00000105848	Gm42683	20		ENSMUSG00000039633	Lonrf1	20
ENSMUSG00000105392	Gm42684	20		ENSMUSG00000048814	Lonrf2	3
ENSMUSG00000105740	Gm42685	20		ENSMUSG00000016239	Lonrf3	20
ENSMUSG00000104535	Gm42686	20		ENSMUSG00000043165	Lor	20
ENSMUSG00000079511	Gm42688	20		ENSMUSG00000024529	Lox	20
ENSMUSG00000104901	Gm42692	20		ENSMUSG00000032818	Loxhd1	18
ENSMUSG00000104721	Gm42696	20		ENSMUSG00000032334	Loxl1	3
ENSMUSG00000105083	Gm42699	20		ENSMUSG00000034205	Loxl2	20
ENSMUSG00000105148	Gm42700	20		ENSMUSG00000000693	Loxl3	20
ENSMUSG00000104583	Gm42701	20		ENSMUSG00000038668	Lpar1	20
ENSMUSG00000106443	Gm42702	20		ENSMUSG00000031861	Lpar2	20
ENSMUSG00000105811	Gm42707	20		ENSMUSG00000049929	Lpar4	9
ENSMUSG00000107023	Gm42715	20		ENSMUSG00000033446	Lpar6	20
ENSMUSG00000105264	Gm42716	1		ENSMUSG00000021608	Lpcat1	20
ENSMUSG00000106197	Gm42717	20		ENSMUSG00000033192	Lpcat2	1
ENSMUSG00000106093	Gm42722	1		ENSMUSG00000004270	Lpcat3	20
ENSMUSG00000105601	Gm42725	20		ENSMUSG00000027134	Lpcat4	1
ENSMUSG00000106767	Gm42727	20		ENSMUSG00000026623	Lpgat1	3
ENSMUSG00000107158	Gm42728	20		ENSMUSG00000020593	Lpin1	9
ENSMUSG00000106696	Gm42729	20		ENSMUSG00000024052	Lpin2	19
ENSMUSG00000106758	Gm42731	20		ENSMUSG00000027412	Lpin3	20
ENSMUSG00000107331	Gm42732	1		ENSMUSG00000015568	Lpl	18
ENSMUSG00000107041	Gm42735	20		ENSMUSG00000009356	Lpo	20
ENSMUSG00000106859	Gm42737	9		ENSMUSG00000033306	Lpp	1
ENSMUSG00000107219	Gm42738	6		ENSMUSG00000024696	Lpxn	20
ENSMUSG00000107068	Gm42742	20		ENSMUSG00000028003	Lrat	20
ENSMUSG00000050936	Gm42743	16		ENSMUSG00000020607	Lratd1	18
ENSMUSG00000105742	Gm42748	1		ENSMUSG00000072568	Lratd2	13
ENSMUSG00000104674	Gm42756	1		ENSMUSG00000028080	Lrba	7
ENSMUSG00000104983	Gm42763	20		ENSMUSG00000068015	Lrch1	18
ENSMUSG00000104929	Gm42766	19		ENSMUSG00000031290	Lrch2	4
ENSMUSG00000104562	Gm42767	19		ENSMUSG00000022801	Lrch3	3
ENSMUSG00000104661	Gm42768	19		ENSMUSG00000093445	Lrch4	20
ENSMUSG00000105922	Gm42769	12		ENSMUSG00000030600	Lrfn1	9
ENSMUSG00000105983	Gm42770	19		ENSMUSG00000040490	Lrfn2	9
ENSMUSG00000106579	Gm42771	19		ENSMUSG00000036957	Lrfn3	1
ENSMUSG00000105700	Gm42772	19		ENSMUSG00000045045	Lrfn4	19
ENSMUSG00000105009	Gm42774	20		ENSMUSG00000035653	Lrfn5	12
ENSMUSG00000104676	Gm42777	20		ENSMUSG00000056215	Lrguk	9
ENSMUSG00000105112	Gm42778	20		ENSMUSG00000056260	Lrif1	20
ENSMUSG00000105890	Gm42780	18		ENSMUSG00000030029	Lrig1	18
ENSMUSG00000105976	Gm42782	20		ENSMUSG00000032913	Lrig2	10
ENSMUSG00000104795	Gm42783	20		ENSMUSG00000020105	Lrig3	20
ENSMUSG00000106892	Gm42791	20		ENSMUSG00000063458	Lrmda	20
ENSMUSG00000106717	Gm42798	9		ENSMUSG00000030263	Lrmp	20
ENSMUSG00000105728	Gm42819	13		ENSMUSG00000040249	Lrp1	9
ENSMUSG00000106427	Gm42820	13		ENSMUSG00000022175	Lrp10	13
ENSMUSG00000106596	Gm42822	9		ENSMUSG00000022305	Lrp12	20
ENSMUSG00000106001	Gm42826	10		ENSMUSG00000049252	Lrp1b	2
ENSMUSG00000105895	Gm42829	20		ENSMUSG00000027070	Lrp2	20
ENSMUSG00000105520	Gm42837	20		ENSMUSG00000031637	Lrp2bp	20
ENSMUSG00000104938	Gm42840	20		ENSMUSG00000001802	Lrp3	1
ENSMUSG00000106528	Gm42841	20		ENSMUSG00000027253	Lrp4	20
ENSMUSG00000106211	Gm42842	9		ENSMUSG00000024913	Lrp5	13

ENSMUSG00000105293	Gm42843	12		ENSMUSG00000030201	Lrp6	20
ENSMUSG00000106743	Gm42847	5		ENSMUSG00000028613	Lrp8	19
ENSMUSG00000107043	Gm42849	18		ENSMUSG00000073779	Lrp8os2	3
ENSMUSG00000085829	Gm4285	18		ENSMUSG00000087200	Lrp8os3	20
ENSMUSG00000106688	Gm42851	20		ENSMUSG00000029103	Lrpap1	19
ENSMUSG00000107021	Gm42853	9		ENSMUSG00000024120	Lrpprc	3
ENSMUSG00000106914	Gm42854	20		ENSMUSG00000032352	Lrrc1	9
ENSMUSG00000106946	Gm42856	20		ENSMUSG00000090291	Lrrc10b	9
ENSMUSG00000107276	Gm42858	10		ENSMUSG00000033728	Lrrc14	20
ENSMUSG00000105179	Gm42866	20		ENSMUSG00000021579	Lrrc14b	20
ENSMUSG00000107128	Gm42867	20		ENSMUSG00000039883	Lrrc17	10
ENSMUSG00000105137	Gm42869	20		ENSMUSG00000041673	Lrrc18	12
ENSMUSG00000105719	Gm42872	20		ENSMUSG00000032495	Lrrc2	20
ENSMUSG00000105217	Gm42873	20		ENSMUSG00000037151	Lrrc20	20
ENSMUSG00000105259	Gm42874	20		ENSMUSG00000030125	Lrrc23	13
ENSMUSG00000105691	Gm42876	20		ENSMUSG00000033707	Lrrc24	20
ENSMUSG00000107085	Gm42883	20		ENSMUSG00000030556	Lrrc28	20
ENSMUSG00000107090	Gm42884	20		ENSMUSG00000041679	Lrrc29	20
ENSMUSG00000107148	Gm42888	9		ENSMUSG00000051652	Lrrc3	18
ENSMUSG00000105852	Gm42890	20		ENSMUSG00000090958	Lrrc32	20
ENSMUSG00000106073	Gm42892	20		ENSMUSG00000054320	Lrrc36	14
ENSMUSG00000106415	Gm42893	20		ENSMUSG00000045201	Lrrc3b	20
ENSMUSG00000106694	Gm42894	3		ENSMUSG00000049939	Lrrc4	4
ENSMUSG00000106676	Gm42895	20		ENSMUSG00000063052	Lrrc40	20
ENSMUSG00000107295	Gm42896	3		ENSMUSG00000028703	Lrrc41	1
ENSMUSG00000107207	Gm42897	20		ENSMUSG00000028617	Lrrc42	20
ENSMUSG00000104687	Gm42899	18		ENSMUSG00000063409	Lrrc43	14
ENSMUSG00000106671	Gm42900	20		ENSMUSG00000025145	Lrrc45	20
ENSMUSG00000104850	Gm42901	20		ENSMUSG00000020878	Lrrc46	20
ENSMUSG00000106396	Gm42902	18		ENSMUSG00000029028	Lrrc47	20
ENSMUSG00000106254	Gm42907	20		ENSMUSG00000047766	Lrrc49	20
ENSMUSG00000104940	Gm42908	20		ENSMUSG00000047085	Lrrc4b	12
ENSMUSG00000105514	Gm42913	20		ENSMUSG00000050587	Lrrc4c	9
ENSMUSG00000106209	Gm42918	20		ENSMUSG00000064307	Lrrc51	20
ENSMUSG00000106404	Gm42919	19		ENSMUSG00000075224	Lrrc55	20
ENSMUSG00000106022	Gm42929	12		ENSMUSG00000038637	Lrrc56	20
ENSMUSG00000106940	Gm42930	20		ENSMUSG00000027286	Lrrc57	19
ENSMUSG00000106295	Gm42932	20		ENSMUSG00000034158	Lrrc58	3
ENSMUSG00000106189	Gm42933	20		ENSMUSG00000020869	Lrrc59	18
ENSMUSG00000104737	Gm42937	20		ENSMUSG00000022375	Lrrc6	1
ENSMUSG00000078377	Gm4294	20		ENSMUSG00000073096	Lrrc61	1
ENSMUSG00000106025	Gm42940	20		ENSMUSG00000023151	Lrrc69	20
ENSMUSG00000104693	Gm42941	20		ENSMUSG00000028176	Lrrc7	7
ENSMUSG00000106047	Gm42942	20		ENSMUSG00000023084	Lrrc71	20
ENSMUSG00000106385	Gm42943	9		ENSMUSG00000071073	Lrrc73	20
ENSMUSG00000104696	Gm42946	20		ENSMUSG00000022759	Lrrc74b	14
ENSMUSG00000104822	Gm42967	20		ENSMUSG00000046417	Lrrc75a	20
ENSMUSG00000105822	Gm42969	3		ENSMUSG00000046807	Lrrc75b	18
ENSMUSG00000106446	Gm42970	20		ENSMUSG00000007476	Lrrc8a	20
ENSMUSG00000105568	Gm42971	3		ENSMUSG00000070639	Lrrc8b	9
ENSMUSG00000105445	Gm42972	16		ENSMUSG00000054720	Lrrc8c	20
ENSMUSG00000105733	Gm42973	20		ENSMUSG00000046079	Lrrc8d	20
ENSMUSG00000104963	Gm42974	3		ENSMUSG00000021090	Lrrc9	20
ENSMUSG00000104814	Gm42979	20		ENSMUSG00000027550	Lrrcc1	5

ENSMUSG00000105282	Gm42981	17		ENSMUSG00000040367	Lrrd1	1
ENSMUSG00000104784	Gm42984	20		ENSMUSG00000026305	Lrrfip1	9
ENSMUSG00000107118	Gm42986	20		ENSMUSG00000032497	Lrrfip2	20
ENSMUSG00000106178	Gm42987	20		ENSMUSG00000019892	Lrriq1	13
ENSMUSG00000106364	Gm42994	7		ENSMUSG00000028182	Lrriq3	20
ENSMUSG00000105466	Gm42998	20		ENSMUSG00000015133	Lrrk1	20
ENSMUSG00000107333	Gm43008	10		ENSMUSG00000036273	Lrrk2	9
ENSMUSG00000105586	Gm43009	20		ENSMUSG00000034648	Lrrn1	3
ENSMUSG00000104571	Gm43010	18		ENSMUSG00000026443	Lrrn2	19
ENSMUSG00000106475	Gm43011	20		ENSMUSG00000036295	Lrrn3	1
ENSMUSG00000105472	Gm43024	1		ENSMUSG00000060780	Lrrtm1	18
ENSMUSG00000107257	Gm43028	20		ENSMUSG00000071862	Lrrtm2	3
ENSMUSG00000106904	Gm43029	20		ENSMUSG00000042846	Lrrtm3	7
ENSMUSG00000106535	Gm43031	20		ENSMUSG00000052581	Lrrtm4	2
ENSMUSG00000106498	Gm43032	3		ENSMUSG00000026792	Lrsam1	9
ENSMUSG00000106662	Gm43034	20		ENSMUSG00000045776	Lrtm1	20
ENSMUSG00000107200	Gm43039	20		ENSMUSG00000055003	Lrtm2	18
ENSMUSG00000106244	Gm43046	19		ENSMUSG00000029703	Lrwd1	20
ENSMUSG00000105828	Gm43048	5		ENSMUSG00000061080	Lsamp	19
ENSMUSG00000107364	Gm43051	20		ENSMUSG00000022538	Lsg1	20
ENSMUSG00000107115	Gm43052	20		ENSMUSG00000037296	Lsm1	20
ENSMUSG00000104569	Gm43054	20		ENSMUSG00000050188	Lsm10	12
ENSMUSG00000107120	Gm43059	20		ENSMUSG00000044847	Lsm11	19
ENSMUSG00000104925	Gm43061	20		ENSMUSG00000020922	Lsm12	3
ENSMUSG00000104671	Gm43062	20		ENSMUSG00000066568	Lsm14a	20
ENSMUSG00000104764	Gm43066	9		ENSMUSG00000039108	Lsm14b	9
ENSMUSG00000105272	Gm43071	20		ENSMUSG00000007050	Lsm2	20
ENSMUSG00000106092	Gm43072	9		ENSMUSG00000034192	Lsm3	20
ENSMUSG00000104745	Gm43073	20		ENSMUSG00000031848	Lsm4	12
ENSMUSG00000105004	Gm43075	20		ENSMUSG00000091625	Lsm5	20
ENSMUSG00000105556	Gm43080	20		ENSMUSG00000031683	Lsm6	20
ENSMUSG00000106103	Gm43081	20		ENSMUSG00000035215	Lsm7	12
ENSMUSG00000107375	Gm43084	20		ENSMUSG00000044155	Lsm8	20
ENSMUSG00000105748	Gm43088	20		ENSMUSG00000071342	Lsmem1	20
ENSMUSG00000105948	Gm43089	1		ENSMUSG00000018819	Lsp1	20
ENSMUSG00000104882	Gm43096	20		ENSMUSG00000001247	Lsr	20
ENSMUSG00000105358	Gm43099	6		ENSMUSG00000033105	Lss	20
ENSMUSG00000104653	Gm43109	20		ENSMUSG00000073412	Lst1	20
ENSMUSG00000105081	Gm43110	20		ENSMUSG00000024402	Lta	20
ENSMUSG00000106472	Gm43111	20		ENSMUSG00000015889	Lta4h	20
ENSMUSG00000106212	Gm43112	20		ENSMUSG00000001870	Ltbp1	20
ENSMUSG00000105062	Gm43113	3		ENSMUSG00000002020	Ltbp2	20
ENSMUSG00000104559	Gm43118	20		ENSMUSG00000024940	Ltbp3	9
ENSMUSG00000106560	Gm43119	10		ENSMUSG00000040488	Ltbp4	9
ENSMUSG00000106961	Gm43128	20		ENSMUSG00000030339	Ltbr	20
ENSMUSG00000105750	Gm43130	6		ENSMUSG00000020377	Ltc4s	13
ENSMUSG00000104704	Gm43133	20		ENSMUSG00000027297	Ltk	20
ENSMUSG00000104970	Gm43137	20		ENSMUSG00000052299	Ltn1	20
ENSMUSG00000104720	Gm43138	20		ENSMUSG00000031072	LTO1	20
ENSMUSG00000105286	Gm43140	11		ENSMUSG00000019814	Ltv1	1
ENSMUSG00000106241	Gm43143	20		ENSMUSG00000024188	Luc7l	4
ENSMUSG00000104591	Gm43145	20		ENSMUSG00000029823	Luc7l2	2
ENSMUSG00000105229	Gm43149	20		ENSMUSG00000020863	Luc7l3	20
ENSMUSG00000086804	Gm43154	20		ENSMUSG00000036446	Lum	20

ENSMUSG00000104587	Gm43161	20		ENSMUSG00000028701	Lurap1	20
ENSMUSG00000104724	Gm43162	1		ENSMUSG00000048706	Lurap1l	20
ENSMUSG00000106729	Gm43165	20		ENSMUSG00000001089	Luzp1	19
ENSMUSG00000106710	Gm43166	20		ENSMUSG00000063297	Luzp2	19
ENSMUSG00000106992	Gm43167	20		ENSMUSG00000115546	Gm49077	20
ENSMUSG00000107358	Gm43168	20		ENSMUSG00000115249	Gm49085	19
ENSMUSG00000107374	Gm43172	20		ENSMUSG00000115454	Gm49086	20
ENSMUSG00000107194	Gm43173	20		ENSMUSG00000115318	Gm49089	20
ENSMUSG00000106292	Gm43174	20		ENSMUSG00000115842	Gm49092	20
ENSMUSG00000105942	Gm43175	2		ENSMUSG00000115316	Gm49096	20
ENSMUSG00000106648	Gm43176	12		ENSMUSG00000114880	Gm49098	20
ENSMUSG00000104847	Gm43177	20		ENSMUSG00000115148	Gm49125	20
ENSMUSG00000106607	Gm43178	20		ENSMUSG00000115234	Gm49130	1
ENSMUSG00000104621	Gm43185	9		ENSMUSG00000115429	Gm49153	13
ENSMUSG00000106497	Gm43195	20		ENSMUSG00000115306	Gm49159	20
ENSMUSG00000107215	Gm43197	20		ENSMUSG00000115718	Gm49179	20
ENSMUSG00000105457	Gm43200	20		ENSMUSG00000115155	Gm49182	3
ENSMUSG00000104852	Gm43201	20		ENSMUSG00000115373	Gm49194	9
ENSMUSG00000104897	Gm43203	20		ENSMUSG00000115833	Gm49196	12
ENSMUSG00000105419	Gm43205	20		ENSMUSG00000115184	Gm49197	3
ENSMUSG00000105011	Gm43210	20		ENSMUSG00000115564	Gm49199	20
ENSMUSG00000105760	Gm43211	20		ENSMUSG00000115124	Gm49201	20
ENSMUSG00000087233	Gm43213	19		ENSMUSG00000115624	Gm49204	20
ENSMUSG00000107312	Gm43229	20		ENSMUSG00000115224	Gm49215	20
ENSMUSG00000106753	Gm43230	20		ENSMUSG00000115383	Gm49216	20
ENSMUSG00000107240	Gm43231	20		ENSMUSG00000114602	Gm49223	20
ENSMUSG00000105694	Gm43237	20		ENSMUSG00000115469	Gm49227	20
ENSMUSG00000105966	Gm43242	1		ENSMUSG00000073427	Gm4924	1
ENSMUSG00000105957	Gm43254	20		ENSMUSG00000115141	Gm49256	20
ENSMUSG00000104568	Gm43255	1		ENSMUSG00000115264	Gm49260	20
ENSMUSG00000105818	Gm43256	20		ENSMUSG00000115329	Gm49272	20
ENSMUSG00000105846	Gm43258	20		ENSMUSG00000112515	Gm4928	12
ENSMUSG00000106432	Gm43259	10		ENSMUSG00000115073	Gm49283	20
ENSMUSG00000105933	Gm43260	20		ENSMUSG00000114995	Gm49284	20
ENSMUSG00000107173	Gm43266	20		ENSMUSG00000115645	Gm49288	20
ENSMUSG00000104662	Gm43267	20		ENSMUSG00000114860	Gm49291	20
ENSMUSG00000107182	Gm43268	20		ENSMUSG00000115674	Gm49297	20
ENSMUSG00000105632	Gm43272	20		ENSMUSG00000115406	Gm49300	20
ENSMUSG00000104966	Gm43273	20		ENSMUSG00000115555	Gm49307	20
ENSMUSG00000107116	Gm43274	20		ENSMUSG00000115210	Gm49308	20
ENSMUSG00000106798	Gm43275	20		ENSMUSG00000115686	Gm49310	20
ENSMUSG00000107300	Gm43279	13		ENSMUSG00000115095	Gm49311	18
ENSMUSG00000106749	Gm43281	20		ENSMUSG00000115771	Gm49312	20
ENSMUSG00000107290	Gm43282	19		ENSMUSG00000111462	Gm49317	20
ENSMUSG00000106490	Gm43283	18		ENSMUSG00000035370	Gm49322	20
ENSMUSG00000105562	Gm43287	18		ENSMUSG00000113294	Gm49326	20
ENSMUSG00000106220	Gm43288	18		ENSMUSG00000113536	Gm49327	1
ENSMUSG00000104917	Gm43289	20		ENSMUSG00000113642	Gm49329	19
ENSMUSG00000104641	Gm43290	1		ENSMUSG00000114025	Gm49331	20
ENSMUSG00000107094	Gm43294	9		ENSMUSG00000112035	Gm49335	20
ENSMUSG00000105572	Gm43300	9		ENSMUSG00000114797	Gm49336	2
ENSMUSG00000105703	Gm43305	12		ENSMUSG00000111128	Gm49338	20
ENSMUSG00000105990	Gm43307	20		ENSMUSG00000110723	Gm49353	20
ENSMUSG00000107197	Gm43312	20		ENSMUSG00000113450	Gm49359	20

ENSMUSG00000107083	Gm43313	20		ENSMUSG00000114479	Gm49375	1
ENSMUSG00000105332	Gm43316	19		ENSMUSG00000112368	Gm49376	20
ENSMUSG00000104655	Gm43317	19		ENSMUSG00000114529	Gm4939	20
ENSMUSG00000105974	Gm43318	19		ENSMUSG00000109378	Gm49396	1
ENSMUSG00000105958	Gm43319	19		ENSMUSG00000024869	Gm49405	9
ENSMUSG00000106037	Gm4332	20		ENSMUSG00000116185	Gm49411	20
ENSMUSG00000106414	Gm43320	20		ENSMUSG00000116238	Gm49413	20
ENSMUSG00000106408	Gm43321	19		ENSMUSG00000115186	Gm49417	20
ENSMUSG00000105939	Gm43322	20		ENSMUSG00000116280	Gm49435	20
ENSMUSG00000107390	Gm43323	20		ENSMUSG00000116493	Gm49445	20
ENSMUSG00000104782	Gm43326	18		ENSMUSG00000116494	Gm49449	20
ENSMUSG00000105695	Gm43327	19		ENSMUSG00000062472	Gm4945	19
ENSMUSG00000105677	Gm43328	20		ENSMUSG00000116358	Gm49450	20
ENSMUSG00000105842	Gm43329	20		ENSMUSG00000116508	Gm49463	20
ENSMUSG00000106341	Gm43330	20		ENSMUSG00000118140	Gm4949	20
ENSMUSG00000104910	Gm43331	20		ENSMUSG00000116180	Gm49492	20
ENSMUSG00000105261	Gm43333	20		ENSMUSG00000108878	Gm49493	20
ENSMUSG00000105071	Gm43336	9		ENSMUSG00000116518	Gm49494	20
ENSMUSG00000105382	Gm43339	20		ENSMUSG00000069379	Gm4950	20
ENSMUSG00000105228	Gm43340	20		ENSMUSG00000116226	Gm49502	20
ENSMUSG00000105791	Gm43341	3		ENSMUSG00000073555	Gm4951	20
ENSMUSG00000107091	Gm43343	20		ENSMUSG00000116480	Gm49512	20
ENSMUSG00000097989	Gm4335	20		ENSMUSG00000115756	Gm49519	20
ENSMUSG00000106369	Gm43355	20		ENSMUSG00000115923	Gm49521	20
ENSMUSG00000105434	Gm43359	20		ENSMUSG00000116321	Gm49522	20
ENSMUSG00000105970	Gm43360	20		ENSMUSG00000115898	Gm49524	20
ENSMUSG00000105201	Gm43362	20		ENSMUSG00000116397	Gm49525	20
ENSMUSG00000105084	Gm43365	20		ENSMUSG00000115975	Gm49526	20
ENSMUSG00000107234	Gm43371	20		ENSMUSG00000061992	Gm4953	12
ENSMUSG00000107335	Gm43372	18		ENSMUSG00000116004	Gm49539	20
ENSMUSG00000107363	Gm43373	7		ENSMUSG00000116272	Gm49540	18
ENSMUSG00000106673	Gm43374	7		ENSMUSG00000115190	Gm49542	20
ENSMUSG00000106262	Gm43375	20		ENSMUSG00000116262	Gm49544	20
ENSMUSG00000106543	Gm43378	20		ENSMUSG00000116243	Gm49552	20
ENSMUSG00000105449	Gm43379	20		ENSMUSG00000116735	Gm49555	20
ENSMUSG00000104874	Gm43387	20		ENSMUSG00000116913	Gm49566	20
ENSMUSG00000106665	Gm43389	18		ENSMUSG00000105536	Gm4959	1
ENSMUSG00000105594	Gm43398	18		ENSMUSG00000116081	Gm49593	9
ENSMUSG00000106542	Gm43410	9		ENSMUSG00000116835	Gm49594	20
ENSMUSG00000105979	Gm43411	20		ENSMUSG00000116908	Gm49599	20
ENSMUSG00000105107	Gm43412	20		ENSMUSG00000116795	Gm49600	20
ENSMUSG00000104951	Gm43413	9		ENSMUSG00000116594	Gm49601	20
ENSMUSG00000106993	Gm43417	20		ENSMUSG00000113831	Gm49602	20
ENSMUSG00000106115	Gm43420	16		ENSMUSG00000116882	Gm49614	18
ENSMUSG00000104706	Gm43421	20		ENSMUSG00000110869	Gm49628	20
ENSMUSG00000106643	Gm43422	9		ENSMUSG00000116904	Gm49640	20
ENSMUSG00000105033	Gm43423	20		ENSMUSG00000113184	Gm49654	18
ENSMUSG00000106214	Gm43426	20		ENSMUSG00000116832	Gm49658	10
ENSMUSG00000105681	Gm43428	20		ENSMUSG00000116740	Gm49659	18
ENSMUSG00000106290	Gm43429	20		ENSMUSG00000116902	Gm49660	20
ENSMUSG00000106149	Gm43430	20		ENSMUSG00000116881	Gm49674	7
ENSMUSG00000104965	Gm43437	20		ENSMUSG00000116867	Gm49677	1
ENSMUSG00000104969	Gm43445	9		ENSMUSG00000116610	Gm49678	20
ENSMUSG00000106678	Gm43457	20		ENSMUSG00000115122	Gm49685	20

ENSMUSG00000105561	Gm43462	20		ENSMUSG00000085601	Gm4969	20
ENSMUSG00000106184	Gm43463	20		ENSMUSG00000116697	Gm49694	9
ENSMUSG00000105717	Gm43465	9		ENSMUSG00000116572	Gm49695	20
ENSMUSG00000105824	Gm43466	20		ENSMUSG00000116635	Gm49699	7
ENSMUSG00000105397	Gm43471	20		ENSMUSG00000116883	Gm49700	20
ENSMUSG00000107076	Gm43480	20		ENSMUSG00000116766	Gm49702	9
ENSMUSG00000107000	Gm43481	20		ENSMUSG00000113687	Gm49703	6
ENSMUSG00000106757	Gm43482	2		ENSMUSG00000116626	Gm49705	6
ENSMUSG00000106166	Gm43484	20		ENSMUSG00000116624	Gm49706	20
ENSMUSG00000106448	Gm43486	20		ENSMUSG00000109609	Gm4972	20
ENSMUSG00000105063	Gm43488	20		ENSMUSG00000116876	Gm49721	20
ENSMUSG00000097532	Gm4349	1		ENSMUSG00000116534	Gm49731	20
ENSMUSG00000105526	Gm43490	3		ENSMUSG00000116571	Gm49741	20
ENSMUSG00000106537	Gm43502	9		ENSMUSG00000116604	Gm49745	20
ENSMUSG00000105347	Gm43503	20		ENSMUSG00000115222	Gm49747	20
ENSMUSG00000104990	Gm43504	20		ENSMUSG00000116764	Gm49748	20
ENSMUSG00000106332	Gm43506	20		ENSMUSG00000111394	Gm49759	9
ENSMUSG00000105861	Gm43508	20		ENSMUSG00000116815	Gm49760	1
ENSMUSG00000106603	Gm43509	20		ENSMUSG00000116617	Gm49767	20
ENSMUSG00000104761	Gm43511	20		ENSMUSG00000115220	Gm49768	20
ENSMUSG00000105607	Gm43513	20		ENSMUSG00000111613	Gm4977	20
ENSMUSG00000089922	Gm43517	9		ENSMUSG00000111740	Gm49783	20
ENSMUSG00000105528	Gm43519	20		ENSMUSG00000116898	Gm49785	20
ENSMUSG00000106310	Gm43523	18		ENSMUSG00000116568	Gm49790	20
ENSMUSG00000105803	Gm43526	20		ENSMUSG00000116858	Gm49797	20
ENSMUSG00000106018	Gm43527	20		ENSMUSG00000117130	Gm49802	20
ENSMUSG00000105290	Gm43528	20		ENSMUSG00000117313	Gm49838	20
ENSMUSG00000091900	Gm4353	9		ENSMUSG00000117294	Gm49839	20
ENSMUSG00000106822	Gm43533	3		ENSMUSG00000117255	Gm49864	18
ENSMUSG00000105549	Gm43540	20		ENSMUSG00000117114	Gm49867	17
ENSMUSG00000105936	Gm43544	3		ENSMUSG00000117335	Gm49870	20
ENSMUSG00000106035	Gm43545	2		ENSMUSG00000117250	Gm49871	1
ENSMUSG00000105019	Gm43547	20		ENSMUSG00000117228	Gm49877	10
ENSMUSG00000107033	Gm43553	9		ENSMUSG00000117113	Gm49883	20
ENSMUSG00000105977	Gm43555	18		ENSMUSG00000117123	Gm49890	20
ENSMUSG00000117145	Gm4356	3		ENSMUSG00000117069	Gm49894	20
ENSMUSG00000106057	Gm43560	20		ENSMUSG00000117222	Gm49906	9
ENSMUSG00000105565	Gm43566	3		ENSMUSG00000117098	Gm49909	11
ENSMUSG00000105945	Gm43570	19		ENSMUSG00000117247	Gm49923	20
ENSMUSG00000106110	Gm43571	9		ENSMUSG00000117126	Gm49924	20
ENSMUSG00000105876	Gm43572	20		ENSMUSG00000117010	Gm49933	20
ENSMUSG00000104786	Gm43573	20		ENSMUSG00000117003	Gm49937	20
ENSMUSG00000105287	Gm43577	3		ENSMUSG00000117332	Gm49942	20
ENSMUSG00000106836	Gm43578	20		ENSMUSG00000117093	Gm49949	20
ENSMUSG00000105199	Gm43581	19		ENSMUSG00000117430	Gm49968	20
ENSMUSG00000106565	Gm43582	20		ENSMUSG00000117647	Gm49969	20
ENSMUSG00000105195	Gm43584	20		ENSMUSG00000117494	Gm49970	20
ENSMUSG00000106666	Gm43587	18		ENSMUSG00000117599	Gm49971	20
ENSMUSG00000107370	Gm43588	20		ENSMUSG00000117372	Gm49972	20
ENSMUSG00000104550	Gm43589	20		ENSMUSG00000117350	Gm49975	20
ENSMUSG00000106288	Gm43593	20		ENSMUSG00000117465	Gm49980	20
ENSMUSG00000107226	Gm43594	20		ENSMUSG00000106738	Gm5	20
ENSMUSG00000107096	Gm43597	8		ENSMUSG00000117538	Gm50008	20
ENSMUSG00000106927	Gm43598	20		ENSMUSG00000117653	Gm50009	2

ENSMUSG00000105762	Gm43605	20		ENSMUSG00000117499	Gm50010	9
ENSMUSG00000105663	Gm43606	20		ENSMUSG00000117628	Gm50012	20
ENSMUSG00000106087	Gm43609	20		ENSMUSG00000117579	Gm50023	20
ENSMUSG00000106054	Gm43623	20		ENSMUSG00000117521	Gm50024	9
ENSMUSG00000105636	Gm43625	20		ENSMUSG00000117507	Gm50045	20
ENSMUSG00000105935	Gm43628	12		ENSMUSG00000117541	Gm50048	20
ENSMUSG00000106962	Gm43633	3		ENSMUSG00000117448	Gm50055	1
ENSMUSG00000107045	Gm43636	20		ENSMUSG00000117634	Gm50069	20
ENSMUSG00000107225	Gm43637	20		ENSMUSG00000117440	Gm50087	20
ENSMUSG00000105574	Gm43648	20		ENSMUSG00000117600	Gm50094	20
ENSMUSG00000105992	Gm43651	20		ENSMUSG00000117818	Gm50105	20
ENSMUSG00000105447	Gm43653	20		ENSMUSG00000118239	Gm50107	20
ENSMUSG00000105804	Gm43654	20		ENSMUSG00000118234	Gm50115	20
ENSMUSG00000106815	Gm43658	20		ENSMUSG00000118242	Gm50141	20
ENSMUSG00000107383	Gm4366	5		ENSMUSG00000118184	Gm50144	20
ENSMUSG00000105571	Gm43662	20		ENSMUSG00000118030	Gm50163	13
ENSMUSG00000105578	Gm43663	20		ENSMUSG00000117757	Gm50169	20
ENSMUSG00000104672	Gm43665	20		ENSMUSG00000118274	Gm50204	20
ENSMUSG00000105135	Gm43667	20		ENSMUSG00000117947	Gm50226	20
ENSMUSG00000105176	Gm43668	20		ENSMUSG00000117725	Gm50240	12
ENSMUSG00000106019	Gm43672	20		ENSMUSG00000117819	Gm50253	20
ENSMUSG00000106153	Gm43677	20		ENSMUSG00000118310	Gm50268	9
ENSMUSG00000105950	Gm43679	20		ENSMUSG00000118287	Gm50300	20
ENSMUSG00000106870	Gm43681	20		ENSMUSG00000118340	Gm50301	18
ENSMUSG00000107019	Gm43682	20		ENSMUSG00000117410	Gm50304	20
ENSMUSG00000105613	Gm43684	20		ENSMUSG00000118077	Gm50315	20
ENSMUSG00000104974	Gm43686	10		ENSMUSG00000118138	Gm50322	20
ENSMUSG00000106980	Gm43690	20		ENSMUSG00000117900	Gm50323	1
ENSMUSG00000105429	Gm43692	20		ENSMUSG00000118026	Gm50335	1
ENSMUSG00000105929	Gm43693	20		ENSMUSG00000118100	Gm50340	20
ENSMUSG00000105304	Gm43696	20		ENSMUSG00000117951	Gm50357	20
ENSMUSG00000105453	Gm43702	20		ENSMUSG00000117732	Gm50364	20
ENSMUSG00000104903	Gm43707	20		ENSMUSG00000118198	Gm50370	1
ENSMUSG00000104649	Gm43712	9		ENSMUSG00000118125	Gm50387	20
ENSMUSG00000105079	Gm43715	20		ENSMUSG00000117789	Gm50388	20
ENSMUSG00000105366	Gm43719	1		ENSMUSG00000118171	Gm50390	9
ENSMUSG00000106202	Gm43727	20		ENSMUSG00000118210	Gm50394	18
ENSMUSG00000104867	Gm43728	20		ENSMUSG00000117943	Gm50403	9
ENSMUSG00000105692	Gm43737	18		ENSMUSG00000107028	Gm5043	20
ENSMUSG00000107639	Gm4374	20		ENSMUSG00000117814	Gm50431	20
ENSMUSG00000107165	Gm43747	20		ENSMUSG00000118052	Gm50432	20
ENSMUSG00000106612	Gm43750	20		ENSMUSG00000117037	Gm50433	20
ENSMUSG00000105322	Gm43751	20		ENSMUSG00000117930	Gm50436	20
ENSMUSG00000105707	Gm43753	20		ENSMUSG00000025922	Gm5045	20
ENSMUSG00000105368	Gm43759	2		ENSMUSG00000111515	Gm5055	20
ENSMUSG00000107106	Gm43760	20		ENSMUSG00000117416	Gm5064	20
ENSMUSG00000105119	Gm43765	20		ENSMUSG00000090671	Gm5067	20
ENSMUSG00000105868	Gm43766	20		ENSMUSG00000055676	Gm5069	20
ENSMUSG00000105412	Gm43768	12		ENSMUSG00000110235	Gm5086	20
ENSMUSG00000106061	Gm43773	20		ENSMUSG00000115263	Gm5089	20
ENSMUSG00000104631	Gm43774	20		ENSMUSG00000064052	Gm5089	20
ENSMUSG00000107304	Gm43775	20		ENSMUSG00000091742	Gm5093	1
ENSMUSG00000105946	Gm43776	20		ENSMUSG00000097279	Gm5106	20
ENSMUSG00000105761	Gm43787	3		ENSMUSG00000052730	Gm5111	20

ENSMUSG00000107286	Gm43788	20		ENSMUSG00000107868	Gm5112	20
ENSMUSG00000105947	Gm43789	20		ENSMUSG00000066647	Gm5113	1
ENSMUSG00000106818	Gm43790	20		ENSMUSG00000093862	Gm5117	20
ENSMUSG00000106732	Gm43792	20		ENSMUSG00000111151	Gm5120	12
ENSMUSG00000107155	Gm43793	12		ENSMUSG00000066626	Gm5121	12
ENSMUSG00000106772	Gm43794	20		ENSMUSG00000051537	Gm5124	20
ENSMUSG00000106720	Gm43795	20		ENSMUSG00000112039	Gm5136	20
ENSMUSG00000106867	Gm43800	13		ENSMUSG00000091183	Gm5141	14
ENSMUSG00000104615	Gm43804	20		ENSMUSG00000071273	Gm5145	10
ENSMUSG00000105971	Gm43805	9		ENSMUSG00000058174	Gm5148	20
ENSMUSG00000107048	Gm43807	20		ENSMUSG00000055795	Gm5160	20
ENSMUSG00000107140	Gm43811	20		ENSMUSG00000116802	Gm5165	10
ENSMUSG00000106636	Gm43813	2		ENSMUSG00000110744	Gm5171	20
ENSMUSG00000105510	Gm43815	20		ENSMUSG00000112621	Gm5173	20
ENSMUSG00000105341	Gm43817	20		ENSMUSG00000111997	Gm5176	20
ENSMUSG00000106224	Gm43823	20		ENSMUSG00000111942	Gm5182	20
ENSMUSG00000106071	Gm43826	1		ENSMUSG00000113412	Gm5191	20
ENSMUSG00000105530	Gm43829	19		ENSMUSG00000117729	Gm5242	1
ENSMUSG00000105454	Gm43830	20		ENSMUSG00000047227	Gm527	13
ENSMUSG00000105653	Gm43831	10		ENSMUSG00000105452	Gm5276	20
ENSMUSG00000106944	Gm43843	20		ENSMUSG00000105124	Gm5297	20
ENSMUSG00000106858	Gm43844	3		ENSMUSG00000107259	Gm5298	20
ENSMUSG00000105753	Gm43847	20		ENSMUSG00000108107	Gm5312	12
ENSMUSG00000106317	Gm43848	20		ENSMUSG00000098050	Gm5345	19
ENSMUSG00000106680	Gm43852	20		ENSMUSG00000087321	Gm5353	7
ENSMUSG00000104548	Gm43857	20		ENSMUSG00000116597	Gm536	20
ENSMUSG00000104546	Gm43858	20		ENSMUSG00000080715	Gm5406	9
ENSMUSG00000105710	Gm43859	20		ENSMUSG00000049414	Gm5417	20
ENSMUSG00000106928	Gm43860	20		ENSMUSG00000097245	Gm5421	20
ENSMUSG00000108168	Gm43864	20		ENSMUSG00000039684	Gm5422	10
ENSMUSG00000108008	Gm43869	20		ENSMUSG00000046687	Gm5424	1
ENSMUSG00000107712	Gm43870	2		ENSMUSG00000111875	Gm5425	20
ENSMUSG00000107913	Gm43871	20		ENSMUSG00000094374	Gm5435	20
ENSMUSG00000108070	Gm43872	20		ENSMUSG00000042962	Gm5436	20
ENSMUSG00000107606	Gm43873	7		ENSMUSG00000101930	Gm5441	20
ENSMUSG00000107779	Gm43874	20		ENSMUSG00000094185	Gm5445	20
ENSMUSG00000107454	Gm43875	20		ENSMUSG00000050243	Gm5446	20
ENSMUSG00000108055	Gm43876	20		ENSMUSG00000063166	Gm5449	20
ENSMUSG00000107571	Gm43877	20		ENSMUSG00000095847	Gm5451	20
ENSMUSG00000107858	Gm43879	9		ENSMUSG00000114687	Gm5452	10
ENSMUSG00000107886	Gm43880	7		ENSMUSG00000062461	Gm5453	3
ENSMUSG00000107442	Gm43881	20		ENSMUSG00000047643	Gm5454	3
ENSMUSG00000107433	Gm43882	20		ENSMUSG00000021741	Gm5457	20
ENSMUSG00000108217	Gm43883	20		ENSMUSG00000075553	Gm5464	20
ENSMUSG00000108123	Gm43884	20		ENSMUSG00000083397	Gm5466	18
ENSMUSG00000107643	Gm43885	20		ENSMUSG00000116083	Gm5469	20
ENSMUSG00000107515	Gm43886	20		ENSMUSG00000116215	Gm5470	9
ENSMUSG00000108189	Gm43887	20		ENSMUSG00000115882	Gm5481	20
ENSMUSG00000107782	Gm43888	20		ENSMUSG00000116912	Gm5487	20
ENSMUSG00000108146	Gm43889	20		ENSMUSG00000098164	Gm5493	9
ENSMUSG00000108053	Gm43890	20		ENSMUSG00000071035	Gm5499	20
ENSMUSG00000108246	Gm43896	20		ENSMUSG00000117484	Gm5500	20
ENSMUSG00000107757	Gm43898	20		ENSMUSG00000117730	Gm5503	9
ENSMUSG00000108040	Gm43900	20		ENSMUSG00000069376	Gm5507	12

ENSMUSG00000107680	Gm43903	20		ENSMUSG00000117669	Gm5509	20
ENSMUSG00000108126	Gm43909	20		ENSMUSG00000100615	Gm5511	20
ENSMUSG00000108024	Gm43912	9		ENSMUSG00000112693	Gm5512	20
ENSMUSG00000107534	Gm43920	20		ENSMUSG00000063586	Gm5513	20
ENSMUSG00000108256	Gm43923	20		ENSMUSG00000045104	Gm5514	20
ENSMUSG00000108200	Gm43931	20		ENSMUSG00000068466	Gm5518	20
ENSMUSG00000107877	Gm43951	20		ENSMUSG00000118252	Gm5521	20
ENSMUSG00000108067	Gm43953	20		ENSMUSG00000099492	Gm5525	20
ENSMUSG00000108169	Gm43958	1		ENSMUSG00000084817	Gm5526	19
ENSMUSG00000108249	Gm43960	20		ENSMUSG00000100007	Gm5527	20
ENSMUSG00000107549	Gm43961	20		ENSMUSG00000067106	Gm5529	20
ENSMUSG00000107962	Gm43980	20		ENSMUSG00000073535	Gm5532	20
ENSMUSG00000108214	Gm43982	20		ENSMUSG00000074758	Gm5535	9
ENSMUSG00000108238	Gm43984	20		ENSMUSG00000069008	Gm5537	20
ENSMUSG00000107835	Gm43999	20		ENSMUSG00000103986	Gm5539	12
ENSMUSG00000107966	Gm44001	20		ENSMUSG00000048916	Gm5540	20
ENSMUSG00000107624	Gm44005	9		ENSMUSG00000106419	Gm5550	20
ENSMUSG00000108035	Gm44021	6		ENSMUSG00000083477	Gm5555	20
ENSMUSG00000107706	Gm44022	2		ENSMUSG00000096726	Gm5558	12
ENSMUSG00000107955	Gm44027	20		ENSMUSG00000096617	Gm5559	20
ENSMUSG00000108154	Gm44033	20		ENSMUSG00000067161	Gm5560	20
ENSMUSG00000107726	Gm44037	20		ENSMUSG00000096474	Gm5561	19
ENSMUSG00000107458	Gm44040	20		ENSMUSG00000046440	Gm5564	18
ENSMUSG00000107604	Gm44041	20		ENSMUSG00000080002	Gm5566	20
ENSMUSG00000107976	Gm44043	2		ENSMUSG00000108045	Gm5576	20
ENSMUSG00000107690	Gm44044	1		ENSMUSG00000084950	Gm5577	20
ENSMUSG00000107461	Gm44045	5		ENSMUSG00000029633	Gm5578	20
ENSMUSG00000108137	Gm44053	20		ENSMUSG00000107906	Gm5580	20
ENSMUSG00000107447	Gm44054	20		ENSMUSG00000108366	Gm5586	20
ENSMUSG00000108095	Gm44067	3		ENSMUSG00000108518	Gm5587	12
ENSMUSG00000107796	Gm44068	20		ENSMUSG00000086108	Gm5602	20
ENSMUSG00000108004	Gm44080	10		ENSMUSG00000109181	Gm5605	20
ENSMUSG00000107801	Gm44086	20		ENSMUSG00000090602	Gm5611	20
ENSMUSG00000107927	Gm44090	20		ENSMUSG00000063543	Gm5616	19
ENSMUSG00000107688	Gm44091	20		ENSMUSG00000042293	Gm5617	3
ENSMUSG00000107576	Gm44093	20		ENSMUSG00000099980	Gm5619	20
ENSMUSG00000107967	Gm44094	9		ENSMUSG00000056904	Gm5620	20
ENSMUSG00000108219	Gm44101	19		ENSMUSG00000113660	Gm5626	20
ENSMUSG00000108093	Gm44102	9		ENSMUSG00000113750	Gm5628	20
ENSMUSG00000107583	Gm44104	3		ENSMUSG00000046993	Gm5637	20
ENSMUSG00000107795	Gm44105	20		ENSMUSG00000069014	Gm5641	10
ENSMUSG00000108122	Gm44116	12		ENSMUSG00000083311	Gm5643	16
ENSMUSG00000107605	Gm44117	20		ENSMUSG00000100104	Gm5644	12
ENSMUSG00000107999	Gm44123	20		ENSMUSG00000080866	Gm5687	20
ENSMUSG00000107770	Gm44126	20		ENSMUSG00000081557	Gm5697	20
ENSMUSG00000108211	Gm44130	3		ENSMUSG00000048600	Gm5763	18
ENSMUSG00000107884	Gm44144	20		ENSMUSG00000099647	Gm5776	12
ENSMUSG00000108152	Gm44152	20		ENSMUSG00000091825	Gm5778	20
ENSMUSG00000108216	Gm44153	20		ENSMUSG00000112471	Gm5779	20
ENSMUSG00000107756	Gm44164	1		ENSMUSG00000112140	Gm5780	20
ENSMUSG00000108297	Gm44167	20		ENSMUSG00000093908	Gm5784	20
ENSMUSG00000108049	Gm44168	20		ENSMUSG00000066487	Gm5786	12
ENSMUSG00000108132	Gm44175	20		ENSMUSG00000114858	Gm5790	20
ENSMUSG00000107676	Gm44178	20		ENSMUSG00000058581	Gm5801	20

ENSMUSG00000108268	Gm44187	20		ENSMUSG00000114488	Gm5802	20
ENSMUSG00000108092	Gm44189	16		ENSMUSG00000061848	Gm5805	12
ENSMUSG00000097055	Gm4419	20		ENSMUSG00000051639	Gm5812	20
ENSMUSG00000108267	Gm44190	20		ENSMUSG00000046952	Gm5815	1
ENSMUSG00000107937	Gm44200	20		ENSMUSG00000117994	Gm5820	20
ENSMUSG00000108079	Gm44210	1		ENSMUSG00000089993	Gm5822	9
ENSMUSG00000108197	Gm44214	20		ENSMUSG00000117813	Gm5823	20
ENSMUSG00000107655	Gm44220	6		ENSMUSG00000117785	Gm5827	20
ENSMUSG00000108286	Gm44224	3		ENSMUSG00000072978	Gm5830	20
ENSMUSG00000107996	Gm44228	16		ENSMUSG00000111133	Gm5831	20
ENSMUSG00000108088	Gm44229	20		ENSMUSG00000101795	Gm5835	1
ENSMUSG00000107905	Gm44230	4		ENSMUSG00000104496	Gm5837	20
ENSMUSG00000108173	Gm44231	2		ENSMUSG00000103921	Gm5841	20
ENSMUSG00000108057	Gm44234	20		ENSMUSG00000103293	Gm5842	9
ENSMUSG00000107917	Gm44235	2		ENSMUSG00000082896	Gm5844	20
ENSMUSG00000107556	Gm44237	20		ENSMUSG00000104309	Gm5846	20
ENSMUSG00000107498	Gm44240	1		ENSMUSG00000082280	Gm5847	20
ENSMUSG00000107840	Gm44241	20		ENSMUSG00000105641	Gm5853	20
ENSMUSG00000107826	Gm44242	1		ENSMUSG00000105558	Gm5855	12
ENSMUSG00000107997	Gm44243	20		ENSMUSG00000106416	Gm5857	4
ENSMUSG00000108199	Gm44249	12		ENSMUSG00000082927	Gm5863	20
ENSMUSG00000107881	Gm44250	13		ENSMUSG00000104923	Gm5865	20
ENSMUSG00000107971	Gm44260	9		ENSMUSG00000043484	Gm5867	20
ENSMUSG00000107472	Gm44265	12		ENSMUSG00000104802	Gm5869	20
ENSMUSG00000107553	Gm44266	20		ENSMUSG00000093651	Gm5873	20
ENSMUSG00000108108	Gm44270	20		ENSMUSG00000071568	Gm5874	9
ENSMUSG00000107586	Gm44283	20		ENSMUSG00000068262	Gm5879	20
ENSMUSG00000108033	Gm44284	20		ENSMUSG00000107747	Gm5881	20
ENSMUSG00000107707	Gm44286	20		ENSMUSG00000037827	Gm5884	20
ENSMUSG00000108109	Gm44287	20		ENSMUSG00000081788	Gm5898	20
ENSMUSG00000107483	Gm44288	20		ENSMUSG00000094685	Gm5900	20
ENSMUSG00000107529	Gm44291	20		ENSMUSG00000082988	Gm5903	20
ENSMUSG00000108291	Gm44292	20		ENSMUSG00000109894	Gm5904	7
ENSMUSG00000103532	Gm4430	20		ENSMUSG00000110275	Gm5905	12
ENSMUSG00000103009	Gm4430	5		ENSMUSG00000082491	Gm5909	20
ENSMUSG00000106175	Gm44313	20		ENSMUSG00000110617	Gm5910	20
ENSMUSG00000106021	Gm44316	20		ENSMUSG00000081436	Gm5912	12
ENSMUSG00000107749	Gm44321	20		ENSMUSG00000081111	Gm5913	20
ENSMUSG00000105998	Gm44324	20		ENSMUSG00000098120	Gm5914	12
ENSMUSG00000107395	Gm44331	20		ENSMUSG00000080021	Gm5915	20
ENSMUSG00000106670	Gm44347	20		ENSMUSG00000068200	Gm5931	20
ENSMUSG00000105100	Gm44349	20		ENSMUSG00000081622	Gm5937	20
ENSMUSG00000106130	Gm44361	20		ENSMUSG00000083258	Gm5939	20
ENSMUSG00000105415	Gm44364	20		ENSMUSG00000048592	Gm5946	4
ENSMUSG00000107689	Gm44386	20		ENSMUSG00000052192	Gm5963	12
ENSMUSG00000104771	Gm44394	20		ENSMUSG00000048411	Gm597	18
ENSMUSG00000108143	Gm44414	2		ENSMUSG00000110701	Gm6013	20
ENSMUSG00000108195	Gm44415	20		ENSMUSG00000081159	Gm6023	20
ENSMUSG00000107436	Gm44416	20		ENSMUSG00000101875	Gm6028	20
ENSMUSG00000107865	Gm44417	20		ENSMUSG00000062284	Gm6030	20
ENSMUSG00000107968	Gm44419	20		ENSMUSG00000114551	Gm6035	20
ENSMUSG00000107451	Gm44421	12		ENSMUSG00000083736	Gm6039	20
ENSMUSG00000108064	Gm44423	20		ENSMUSG00000075245	Gm6043	20
ENSMUSG00000107698	Gm44430	20		ENSMUSG00000057157	Gm6054	20

ENSMUSG00000107932	Gm44432	20		ENSMUSG00000062081	Gm6055	20
ENSMUSG00000107512	Gm44433	20		ENSMUSG00000087632	Gm6058	20
ENSMUSG00000107813	Gm44434	20		ENSMUSG00000093392	Gm6061	20
ENSMUSG00000108005	Gm44435	2		ENSMUSG00000108772	Gm6063	12
ENSMUSG00000107823	Gm44436	20		ENSMUSG00000098104	Gm6085	20
ENSMUSG00000108102	Gm44437	3		ENSMUSG00000115684	Gm6087	9
ENSMUSG00000107947	Gm44438	2		ENSMUSG00000105422	Gm6089	1
ENSMUSG00000107804	Gm44439	1		ENSMUSG00000053182	Gm609	20
ENSMUSG00000107511	Gm44440	20		ENSMUSG00000062588	Gm6104	20
ENSMUSG00000107595	Gm44441	20		ENSMUSG00000113237	Gm6109	12
ENSMUSG00000108640	Gm44508	20		ENSMUSG00000103922	Gm6123	20
ENSMUSG00000108358	Gm44509	20		ENSMUSG00000113843	Gm6129	20
ENSMUSG00000108443	Gm44510	20		ENSMUSG00000084106	Gm6136	12
ENSMUSG00000109385	Gm44518	19		ENSMUSG00000084304	Gm6142	20
ENSMUSG00000109286	Gm44541	19		ENSMUSG00000110080	Gm6145	20
ENSMUSG00000109040	Gm44542	18		ENSMUSG00000105687	Gm6157	20
ENSMUSG00000109379	Gm44550	1		ENSMUSG00000090381	Gm6158	20
ENSMUSG00000108319	Gm44552	20		ENSMUSG00000099377	Gm6159	20
ENSMUSG00000108741	Gm44553	20		ENSMUSG00000074280	Gm6166	20
ENSMUSG00000108973	Gm44555	9		ENSMUSG00000100078	Gm6170	20
ENSMUSG00000108943	Gm44559	16		ENSMUSG00000053038	Gm6180	20
ENSMUSG00000108880	Gm44560	3		ENSMUSG00000113861	Gm6190	20
ENSMUSG00000109362	Gm44562	9		ENSMUSG00000118220	Gm6192	20
ENSMUSG00000108447	Gm44567	20		ENSMUSG00000104295	Gm6197	20
ENSMUSG00000109456	Gm44584	9		ENSMUSG00000105879	Gm6204	12
ENSMUSG00000109430	Gm44585	20		ENSMUSG00000048949	Gm6206	20
ENSMUSG00000109082	Gm44586	9		ENSMUSG00000107951	Gm6210	1
ENSMUSG00000109094	Gm44587	9		ENSMUSG00000091577	Gm6211	18
ENSMUSG00000083626	Gm4459	20		ENSMUSG00000115196	Gm6212	20
ENSMUSG00000109088	Gm44593	9		ENSMUSG00000112145	Gm6218	13
ENSMUSG00000108037	Gm44597	20		ENSMUSG00000083019	Gm6222	9
ENSMUSG00000108630	Gm44607	20		ENSMUSG00000096647	Gm6223	20
ENSMUSG00000108701	Gm44616	9		ENSMUSG00000097746	Gm6225	20
ENSMUSG00000090319	Gm4462	20		ENSMUSG00000086926	Gm6226	20
ENSMUSG00000109570	Gm44624	20		ENSMUSG00000082996	Gm6238	20
ENSMUSG00000108910	Gm44625	20		ENSMUSG00000090475	Gm6245	1
ENSMUSG00000108521	Gm44639	2		ENSMUSG00000117804	Gm6252	12
ENSMUSG00000108867	Gm44643	20		ENSMUSG00000066538	Gm6254	20
ENSMUSG00000109121	Gm44644	20		ENSMUSG00000091866	Gm6257	20
ENSMUSG00000108601	Gm44645	20		ENSMUSG00000080966	Gm6263	1
ENSMUSG00000108607	Gm44646	20		ENSMUSG00000081281	Gm6274	20
ENSMUSG00000108317	Gm44663	9		ENSMUSG00000097440	Gm6277	20
ENSMUSG00000108522	Gm44664	9		ENSMUSG00000116972	Gm6278	20
ENSMUSG00000109539	Gm44667	20		ENSMUSG00000081703	Gm6285	20
ENSMUSG00000109363	Gm44668	20		ENSMUSG00000086925	Gm6286	20
ENSMUSG00000108388	Gm44673	5		ENSMUSG00000071867	Gm6304	20
ENSMUSG00000109370	Gm44675	7		ENSMUSG00000061833	Gm6311	12
ENSMUSG00000109217	Gm44676	7		ENSMUSG00000078370	Gm6316	20
ENSMUSG00000109505	Gm44677	7		ENSMUSG00000083392	Gm6335	20
ENSMUSG00000108920	Gm44678	6		ENSMUSG00000096964	Gm6345	20
ENSMUSG00000109320	Gm44679	9		ENSMUSG00000107952	Gm6352	20
ENSMUSG00000109044	Gm44680	9		ENSMUSG00000114993	Gm6363	20
ENSMUSG00000109256	Gm44681	20		ENSMUSG00000051548	Gm6365	20
ENSMUSG00000108633	Gm44694	18		ENSMUSG00000080775	Gm6368	20

ENSMUSG00000108560	Gm44695	20		ENSMUSG00000107950	Gm6375	20
ENSMUSG00000107875	Gm44696	1		ENSMUSG00000082675	Gm6382	20
ENSMUSG00000108959	Gm44697	20		ENSMUSG00000104623	Gm6394	1
ENSMUSG00000109366	Gm44698	1		ENSMUSG00000091905	Gm6395	20
ENSMUSG00000109314	Gm44699	20		ENSMUSG00000117962	Gm6402	5
ENSMUSG00000109576	Gm44704	14		ENSMUSG00000072407	Gm6419	20
ENSMUSG00000108597	Gm44708	20		ENSMUSG00000114540	Gm6421	5
ENSMUSG00000108721	Gm44721	20		ENSMUSG00000044757	Gm6430	20
ENSMUSG00000108806	Gm44729	20		ENSMUSG00000116694	Gm6440	20
ENSMUSG00000108543	Gm44735	20		ENSMUSG00000053740	Gm6457	20
ENSMUSG00000108473	Gm44739	20		ENSMUSG00000117327	Gm6467	12
ENSMUSG00000109057	Gm44741	20		ENSMUSG00000095597	Gm6472	12
ENSMUSG00000109332	Gm44742	3		ENSMUSG00000100261	Gm6473	20
ENSMUSG00000108455	Gm44745	9		ENSMUSG00000116838	Gm6475	20
ENSMUSG00000108341	Gm44746	7		ENSMUSG00000111877	Gm6477	20
ENSMUSG00000108750	Gm44750	20		ENSMUSG00000087153	Gm6483	20
ENSMUSG00000108678	Gm44758	20		ENSMUSG00000089999	Gm6485	20
ENSMUSG00000109517	Gm44763	20		ENSMUSG00000090021	Gm6493	18
ENSMUSG00000108802	Gm44769	20		ENSMUSG00000099998	Gm6501	20
ENSMUSG00000108594	Gm44770	20		ENSMUSG00000070522	Gm6505	20
ENSMUSG00000109374	Gm44773	20		ENSMUSG00000082420	Gm6517	20
ENSMUSG00000108955	Gm44775	20		ENSMUSG00000104862	Gm6520	18
ENSMUSG00000108738	Gm44777	20		ENSMUSG00000115160	Gm6532	20
ENSMUSG00000108338	Gm44794	20		ENSMUSG00000115193	Gm6533	20
ENSMUSG00000109359	Gm44797	3		ENSMUSG00000117049	Gm6540	20
ENSMUSG00000109139	Gm44798	19		ENSMUSG00000085172	Gm6542	20
ENSMUSG00000109095	Gm44799	19		ENSMUSG00000106135	Gm6543	20
ENSMUSG00000109418	Gm44800	3		ENSMUSG00000111118	Gm6545	18
ENSMUSG00000109529	Gm44801	19		ENSMUSG00000091549	Gm6548	12
ENSMUSG00000108918	Gm44802	3		ENSMUSG00000117458	Gm6552	20
ENSMUSG00000108790	Gm44806	2		ENSMUSG00000097806	Gm6556	9
ENSMUSG00000109021	Gm44812	18		ENSMUSG00000104913	Gm6560	20
ENSMUSG00000107066	Gm4482	20		ENSMUSG00000051255	Gm6563	20
ENSMUSG00000109154	Gm44822	20		ENSMUSG00000079224	Gm6565	20
ENSMUSG00000109157	Gm44829	1		ENSMUSG00000113918	Gm6566	20
ENSMUSG00000108886	Gm44830	1		ENSMUSG00000083169	Gm6580	20
ENSMUSG00000109575	Gm44831	3		ENSMUSG00000111446	Gm6581	10
ENSMUSG00000108832	Gm44832	20		ENSMUSG00000107143	Gm6598	20
ENSMUSG00000108542	Gm44834	20		ENSMUSG00000091997	Gm6611	20
ENSMUSG00000108557	Gm44835	20		ENSMUSG00000112550	Gm6627	20
ENSMUSG00000108486	Gm44836	20		ENSMUSG00000083681	Gm6640	20
ENSMUSG00000108993	Gm44846	9		ENSMUSG00000100309	Gm6644	12
ENSMUSG00000108890	Gm44847	18		ENSMUSG00000105645	Gm6649	20
ENSMUSG00000109461	Gm44848	9		ENSMUSG00000099858	Gm6652	20
ENSMUSG00000109245	Gm44860	20		ENSMUSG00000084291	Gm6654	12
ENSMUSG00000108897	Gm44861	20		ENSMUSG00000074171	Gm6658	20
ENSMUSG00000109131	Gm44862	20		ENSMUSG00000117929	Gm6663	20
ENSMUSG00000109416	Gm44863	20		ENSMUSG00000107713	Gm6681	20
ENSMUSG00000108376	Gm44874	9		ENSMUSG00000091269	Gm6682	4
ENSMUSG00000108477	Gm44878	20		ENSMUSG00000032889	Gm6685	20
ENSMUSG00000108572	Gm44883	20		ENSMUSG00000116958	Gm6705	20
ENSMUSG00000109555	Gm44891	20		ENSMUSG00000078887	Gm6710	20
ENSMUSG00000108756	Gm44894	20		ENSMUSG00000072761	Gm6712	20
ENSMUSG00000108474	Gm44895	20		ENSMUSG00000111609	Gm6713	1

ENSMUSG00000108665	Gm44897	20		ENSMUSG00000112318	Gm6721	1
ENSMUSG00000108325	Gm44898	20		ENSMUSG00000091408	Gm6728	12
ENSMUSG00000109008	Gm44899	20		ENSMUSG00000084329	Gm6733	20
ENSMUSG00000108954	Gm44901	20		ENSMUSG00000071414	Gm6736	20
ENSMUSG00000109216	Gm44907	20		ENSMUSG00000043346	Gm6741	20
ENSMUSG00000089840	Gm4491	1		ENSMUSG00000105866	Gm6745	20
ENSMUSG00000108852	Gm44911	20		ENSMUSG00000081118	Gm6758	20
ENSMUSG00000109051	Gm44913	20		ENSMUSG00000081453	Gm6767	12
ENSMUSG00000108984	Gm44916	20		ENSMUSG00000021908	Gm6768	20
ENSMUSG00000108983	Gm44926	9		ENSMUSG00000047509	Gm6776	20
ENSMUSG00000108893	Gm44927	20		ENSMUSG00000035129	Gm6781	20
ENSMUSG00000108643	Gm44931	20		ENSMUSG00000117875	Gm6789	20
ENSMUSG00000109393	Gm44936	20		ENSMUSG00000092086	Gm6793	20
ENSMUSG00000109434	Gm44937	20		ENSMUSG00000057605	Gm6807	18
ENSMUSG00000108655	Gm44949	20		ENSMUSG00000117972	Gm6813	20
ENSMUSG00000108350	Gm44950	20		ENSMUSG00000109488	Gm6828	20
ENSMUSG00000108365	Gm44951	12		ENSMUSG00000083670	Gm6829	20
ENSMUSG00000109109	Gm44953	20		ENSMUSG00000056836	Gm6851	20
ENSMUSG00000109205	Gm44954	20		ENSMUSG00000043483	Gm6863	20
ENSMUSG00000107497	Gm44957	20		ENSMUSG00000083557	Gm6877	20
ENSMUSG00000109302	Gm44967	9		ENSMUSG00000117515	Gm6883	20
ENSMUSG00000109073	Gm44975	20		ENSMUSG00000093826	Gm6900	20
ENSMUSG00000108586	Gm44976	20		ENSMUSG00000090074	Gm6921	20
ENSMUSG00000108450	Gm44977	20		ENSMUSG00000103753	Gm6934	9
ENSMUSG00000109399	Gm44982	20		ENSMUSG00000117856	Gm6937	20
ENSMUSG00000108573	Gm44986	20		ENSMUSG00000082922	Gm6939	20
ENSMUSG00000108511	Gm44987	20		ENSMUSG00000098141	Gm6944	20
ENSMUSG00000109199	Gm44996	12		ENSMUSG00000066553	Gm6969	20
ENSMUSG00000108446	Gm44997	20		ENSMUSG00000091230	Gm6970	20
ENSMUSG00000109128	Gm45022	20		ENSMUSG00000117673	Gm6974	20
ENSMUSG00000109010	Gm45027	20		ENSMUSG00000098076	Gm6981	1
ENSMUSG00000108500	Gm45033	20		ENSMUSG00000111671	Gm6983	10
ENSMUSG00000109317	Gm45041	20		ENSMUSG00000113198	Gm6988	10
ENSMUSG00000109337	Gm45047	20		ENSMUSG00000115743	Gm7004	20
ENSMUSG00000108368	Gm45053	19		ENSMUSG00000035983	Gm7008	20
ENSMUSG00000109438	Gm45073	18		ENSMUSG00000067121	Gm7027	7
ENSMUSG00000109568	Gm45074	9		ENSMUSG00000113473	Gm7045	20
ENSMUSG00000108384	Gm45082	20		ENSMUSG00000113637	Gm7049	20
ENSMUSG00000109045	Gm45084	1		ENSMUSG00000114217	Gm7054	20
ENSMUSG00000109090	Gm45088	20		ENSMUSG00000117468	Gm7059	20
ENSMUSG00000108753	Gm45094	20		ENSMUSG00000082776	Gm7061	20
ENSMUSG00000108871	Gm45102	1		ENSMUSG00000117284	Gm7072	20
ENSMUSG00000109209	Gm45104	20		ENSMUSG00000096375	Gm7094	20
ENSMUSG00000108465	Gm45110	20		ENSMUSG00000060647	Gm7099	20
ENSMUSG00000109397	Gm45117	20		ENSMUSG00000118345	Gm7105	20
ENSMUSG00000109038	Gm45120	20		ENSMUSG00000115280	Gm7107	20
ENSMUSG00000108372	Gm45124	20		ENSMUSG00000100481	Gm7114	20
ENSMUSG00000108953	Gm45129	9		ENSMUSG00000080914	Gm7129	20
ENSMUSG00000109481	Gm45130	9		ENSMUSG00000114184	Gm7143	20
ENSMUSG00000109191	Gm45131	20		ENSMUSG00000099843	Gm7160	20
ENSMUSG00000109274	Gm45133	20		ENSMUSG00000112926	Gm7172	20
ENSMUSG00000109206	Gm45137	6		ENSMUSG00000083774	Gm7180	20
ENSMUSG00000109165	Gm45148	20		ENSMUSG00000106813	Gm7181	20
ENSMUSG00000108736	Gm45151	20		ENSMUSG00000082475	Gm7206	20

ENSMUSG00000108693	Gm45153	20		ENSMUSG00000115662	Gm7232	20
ENSMUSG00000109125	Gm45159	2		ENSMUSG00000094151	Gm7233	20
ENSMUSG00000109419	Gm45163	20		ENSMUSG00000113626	Gm7240	9
ENSMUSG00000109413	Gm45165	18		ENSMUSG00000091003	Gm7244	20
ENSMUSG00000108776	Gm45169	20		ENSMUSG00000069236	Gm7251	20
ENSMUSG00000109422	Gm45174	20		ENSMUSG00000114135	Gm7252	20
ENSMUSG00000109239	Gm45176	6		ENSMUSG00000117060	Gm7253	20
ENSMUSG00000108819	Gm45177	6		ENSMUSG00000102573	Gm7265	20
ENSMUSG00000109562	Gm45178	6		ENSMUSG00000073532	Gm7276	20
ENSMUSG00000108956	Gm45179	7		ENSMUSG00000080950	Gm7278	20
ENSMUSG00000109387	Gm45182	6		ENSMUSG00000105408	Gm7285	20
ENSMUSG00000109353	Gm45183	2		ENSMUSG00000104222	Gm7292	20
ENSMUSG00000108752	Gm45191	9		ENSMUSG00000111162	Gm7293	20
ENSMUSG00000109257	Gm45200	8		ENSMUSG00000081113	Gm7308	20
ENSMUSG00000109031	Gm45201	9		ENSMUSG00000049235	Gm7324	19
ENSMUSG00000108494	Gm45203	9		ENSMUSG00000050900	Gm7327	20
ENSMUSG00000108808	Gm45204	20		ENSMUSG00000110043	Gm7328	4
ENSMUSG00000108353	Gm45205	20		ENSMUSG00000059461	Gm7331	12
ENSMUSG00000108389	Gm45206	20		ENSMUSG00000080875	Gm7332	20
ENSMUSG00000108584	Gm45216	12		ENSMUSG00000044645	Gm7334	20
ENSMUSG00000109279	Gm45220	20		ENSMUSG00000067189	Gm7335	20
ENSMUSG00000109005	Gm45221	20		ENSMUSG00000059645	Gm7361	20
ENSMUSG00000109498	Gm45222	20		ENSMUSG00000083344	Gm7363	20
ENSMUSG00000109429	Gm45223	2		ENSMUSG00000047370	Gm7367	20
ENSMUSG00000109448	Gm45224	12		ENSMUSG00000097657	Gm7389	1
ENSMUSG00000090257	Gm4524	20		ENSMUSG00000110644	Gm7390	9
ENSMUSG00000110317	Gm45250	18		ENSMUSG00000112908	Gm7392	20
ENSMUSG00000110084	Gm45257	20		ENSMUSG00000091941	Gm7399	16
ENSMUSG00000110269	Gm45258	20		ENSMUSG00000089975	Gm7420	9
ENSMUSG00000109942	Gm45262	20		ENSMUSG00000082260	Gm7429	18
ENSMUSG00000110342	Gm45263	20		ENSMUSG00000109241	Gm7434	20
ENSMUSG00000109927	Gm45264	20		ENSMUSG00000111662	Gm7435	20
ENSMUSG00000109725	Gm45265	6		ENSMUSG00000113258	Gm7446	20
ENSMUSG00000109787	Gm45286	20		ENSMUSG00000109489	Gm7451	20
ENSMUSG00000109642	Gm45289	20		ENSMUSG00000107310	Gm7452	18
ENSMUSG00000110079	Gm45292	9		ENSMUSG00000115730	Gm7459	20
ENSMUSG00000110325	Gm45293	12		ENSMUSG00000117425	Gm7464	20
ENSMUSG00000110068	Gm45294	20		ENSMUSG00000098449	Gm7467	20
ENSMUSG00000109775	Gm45297	7		ENSMUSG00000112605	Gm7476	5
ENSMUSG00000110329	Gm45304	20		ENSMUSG00000039617	Gm7488	1
ENSMUSG00000110087	Gm45309	20		ENSMUSG00000066693	Gm7493	10
ENSMUSG00000110272	Gm45310	20		ENSMUSG00000098142	Gm7507	20
ENSMUSG00000110316	Gm45311	20		ENSMUSG00000113137	Gm7511	20
ENSMUSG00000109925	Gm45315	20		ENSMUSG00000115700	Gm7517	20
ENSMUSG00000110375	Gm45323	20		ENSMUSG00000117503	Gm7527	20
ENSMUSG00000109783	Gm45338	20		ENSMUSG00000090957	Gm7535	20
ENSMUSG00000109838	Gm45341	20		ENSMUSG00000057036	Gm7536	12
ENSMUSG00000109679	Gm45342	3		ENSMUSG00000115738	Gm7543	20
ENSMUSG00000110088	Gm45343	20		ENSMUSG00000101784	Gm7553	20
ENSMUSG00000110099	Gm45344	12		ENSMUSG00000114349	Gm7591	20
ENSMUSG00000109957	Gm45353	1		ENSMUSG00000087390	Gm7598	20
ENSMUSG00000108689	Gm45354	20		ENSMUSG00000109933	Gm7600	20
ENSMUSG00000110162	Gm45356	20		ENSMUSG00000095288	Gm7618	9
ENSMUSG00000109585	Gm45358	20		ENSMUSG00000118283	Gm7623	20

ENSMUSG00000109728	Gm45359	20		ENSMUSG00000025644	Gm7628	20
ENSMUSG00000109852	Gm45360	20		ENSMUSG00000090667	Gm765	20
ENSMUSG00000110377	Gm45363	20		ENSMUSG00000097991	Gm7656	18
ENSMUSG00000110326	Gm45378	20		ENSMUSG00000082935	Gm7658	20
ENSMUSG00000110360	Gm45380	9		ENSMUSG00000113428	Gm7664	20
ENSMUSG00000109940	Gm45399	20		ENSMUSG00000067547	Gm7666	20
ENSMUSG00000092072	Gm4540	12		ENSMUSG00000097086	Gm7672	20
ENSMUSG00000109873	Gm45407	9		ENSMUSG00000102752	Gm7694	20
ENSMUSG00000109640	Gm45413	12		ENSMUSG00000091363	Gm7701	20
ENSMUSG00000110400	Gm45416	1		ENSMUSG00000046636	Gm7729	20
ENSMUSG00000110341	Gm45424	20		ENSMUSG00000110545	Gm7730	20
ENSMUSG00000109792	Gm45426	20		ENSMUSG00000105189	Gm7774	20
ENSMUSG00000109956	Gm45428	20		ENSMUSG00000083064	Gm7785	20
ENSMUSG00000110020	Gm45441	9		ENSMUSG00000091460	Gm7808	12
ENSMUSG00000109780	Gm45447	5		ENSMUSG00000106201	Gm7815	20
ENSMUSG00000109741	Gm45455	20		ENSMUSG00000083261	Gm7816	20
ENSMUSG00000110411	Gm45457	20		ENSMUSG00000117234	Gm7818	20
ENSMUSG00000109744	Gm45461	9		ENSMUSG00000106917	Gm7832	20
ENSMUSG00000109727	Gm45464	20		ENSMUSG00000086240	Gm7846	20
ENSMUSG00000109674	Gm45470	20		ENSMUSG00000085720	Gm7854	1
ENSMUSG00000108394	Gm45477	3		ENSMUSG00000116089	Gm7859	20
ENSMUSG00000109693	Gm45483	9		ENSMUSG00000046580	Gm7862	20
ENSMUSG00000110046	Gm45484	20		ENSMUSG00000100351	Gm7867	20
ENSMUSG00000109784	Gm45493	5		ENSMUSG00000113600	Gm7868	20
ENSMUSG00000106981	Gm45495	20		ENSMUSG00000044211	Gm7887	20
ENSMUSG00000110187	Gm45496	20		ENSMUSG00000100347	Gm7895	20
ENSMUSG00000110159	Gm45501	20		ENSMUSG00000103823	Gm7899	10
ENSMUSG00000110308	Gm45516	7		ENSMUSG00000101431	Gm7901	16
ENSMUSG00000109622	Gm45517	9		ENSMUSG00000106067	Gm7902	20
ENSMUSG00000109982	Gm45520	20		ENSMUSG00000115602	Gm7908	20
ENSMUSG00000109618	Gm45527	20		ENSMUSG00000098068	Gm7909	20
ENSMUSG00000110167	Gm45532	20		ENSMUSG00000117345	Gm7926	20
ENSMUSG00000110296	Gm45533	12		ENSMUSG00000067321	Gm7931	20
ENSMUSG00000110405	Gm45534	20		ENSMUSG00000108084	Gm7932	19
ENSMUSG00000109606	Gm45537	20		ENSMUSG00000075609	Gm7935	20
ENSMUSG00000110397	Gm45540	20		ENSMUSG00000055771	Gm7936	20
ENSMUSG00000110279	Gm45552	20		ENSMUSG00000114999	Gm7962	20
ENSMUSG00000109652	Gm45555	2		ENSMUSG00000063902	Gm7964	20
ENSMUSG00000110160	Gm45560	20		ENSMUSG00000100009	Gm7967	20
ENSMUSG00000110038	Gm45570	9		ENSMUSG00000115144	Gm7968	20
ENSMUSG00000110137	Gm45577	20		ENSMUSG00000109032	Gm7972	1
ENSMUSG00000110389	Gm45579	20		ENSMUSG00000049891	Gm7984	20
ENSMUSG00000109460	Gm45591	19		ENSMUSG00000107092	Gm7993	20
ENSMUSG00000109810	Gm45592	20		ENSMUSG00000102493	Gm8010	1
ENSMUSG00000109608	Gm45597	20		ENSMUSG00000094281	Gm8034	20
ENSMUSG00000109945	Gm45601	9		ENSMUSG00000069986	Gm8054	20
ENSMUSG00000109829	Gm45605	20		ENSMUSG00000096171	Gm8055	20
ENSMUSG00000109715	Gm45606	1		ENSMUSG00000106237	Gm8066	9
ENSMUSG00000109877	Gm45609	20		ENSMUSG00000105143	Gm8069	9
ENSMUSG00000110070	Gm45619	20		ENSMUSG00000097848	Gm807	20
ENSMUSG00000110086	Gm45623	16		ENSMUSG00000073073	Gm8098	9
ENSMUSG00000110010	Gm45629	20		ENSMUSG00000105787	Gm8099	20
ENSMUSG00000109638	Gm45630	20		ENSMUSG00000059422	Gm8116	20
ENSMUSG00000110243	Gm45631	20		ENSMUSG00000051116	Gm8121	19

ENSMUSG00000110077	Gm45632	20		ENSMUSG00000059159	Gm8129	20
ENSMUSG00000110227	Gm45634	13		ENSMUSG00000116637	Gm8130	20
ENSMUSG00000109791	Gm45636	13		ENSMUSG00000100025	Gm8141	20
ENSMUSG00000109812	Gm45640	20		ENSMUSG00000103260	Gm8146	1
ENSMUSG00000109863	Gm45643	9		ENSMUSG00000100534	Gm816	20
ENSMUSG00000109636	Gm45644	20		ENSMUSG00000112381	Gm8170	10
ENSMUSG00000109849	Gm45645	20		ENSMUSG00000082926	Gm8172	12
ENSMUSG00000110263	Gm45652	20		ENSMUSG00000100555	Gm8173	1
ENSMUSG00000109828	Gm45662	20		ENSMUSG00000048188	Gm8181	20
ENSMUSG00000109735	Gm45667	20		ENSMUSG00000117877	Gm8184	20
ENSMUSG00000109917	Gm45671	9		ENSMUSG00000090119	Gm8185	20
ENSMUSG00000110529	Gm45694	9		ENSMUSG00000049124	Gm8186	20
ENSMUSG00000110564	Gm45697	20		ENSMUSG00000112163	Gm8188	20
ENSMUSG00000109428	Gm45698	20		ENSMUSG00000101878	Gm8203	9
ENSMUSG00000110483	Gm45702	1		ENSMUSG00000084303	Gm8216	9
ENSMUSG00000110696	Gm45706	3		ENSMUSG00000099927	Gm8226	20
ENSMUSG00000110534	Gm45708	1		ENSMUSG00000099779	Gm8228	20
ENSMUSG00000110706	Gm45709	20		ENSMUSG00000105021	Gm8234	20
ENSMUSG00000110344	Gm45716	20		ENSMUSG00000090707	Gm8237	20
ENSMUSG00000109390	Gm45718	20		ENSMUSG00000102827	Gm8242	20
ENSMUSG00000108940	Gm45719	20		ENSMUSG00000116861	Gm8253	12
ENSMUSG00000110546	Gm45734	20		ENSMUSG00000048334	Gm8258	20
ENSMUSG00000109231	Gm45737	20		ENSMUSG00000081718	Gm8261	20
ENSMUSG00000108101	Gm45738	20		ENSMUSG00000102550	Gm8276	20
ENSMUSG00000110540	Gm45743	20		ENSMUSG00000095681	Gm8281	20
ENSMUSG00000098240	Gm4575	20		ENSMUSG00000110361	Gm8291	20
ENSMUSG00000110711	Gm45760	20		ENSMUSG00000100215	Gm8292	20
ENSMUSG00000110556	Gm45762	20		ENSMUSG00000098222	Gm8318	20
ENSMUSG00000110702	Gm45767	20		ENSMUSG00000108528	Gm8319	20
ENSMUSG00000107838	Gm45769	20		ENSMUSG00000027694	Gm8325	20
ENSMUSG00000110588	Gm45774	20		ENSMUSG00000101262	Gm8326	20
ENSMUSG00000110558	Gm45779	20		ENSMUSG00000100033	Gm8337	20
ENSMUSG00000110136	Gm45785	20		ENSMUSG00000093798	Gm8355	20
ENSMUSG00000110672	Gm45786	20		ENSMUSG00000090722	Gm8378	20
ENSMUSG00000109150	Gm45793	20		ENSMUSG00000050490	Gm8394	20
ENSMUSG00000110504	Gm45797	20		ENSMUSG00000043889	Gm8399	1
ENSMUSG00000110442	Gm45804	20		ENSMUSG00000103964	Gm8407	20
ENSMUSG00000108991	Gm45807	20		ENSMUSG00000055093	Gm8430	12
ENSMUSG00000109708	Gm45809	3		ENSMUSG00000118295	Gm8437	20
ENSMUSG00000110471	Gm45823	20		ENSMUSG00000099471	Gm8451	20
ENSMUSG00000108526	Gm45828	9		ENSMUSG00000106028	Gm8493	20
ENSMUSG00000110465	Gm45832	20		ENSMUSG00000094090	Gm8494	20
ENSMUSG00000110673	Gm45833	20		ENSMUSG00000049477	Gm8508	20
ENSMUSG00000108825	Gm45838	20		ENSMUSG00000114765	Gm8514	20
ENSMUSG00000110105	Gm45844	20		ENSMUSG00000103206	Gm8515	20
ENSMUSG00000109427	Gm45845	20		ENSMUSG00000115312	Gm8518	20
ENSMUSG00000109814	Gm45847	20		ENSMUSG00000106294	Gm8539	20
ENSMUSG00000110635	Gm45853	20		ENSMUSG00000090535	Gm8540	20
ENSMUSG00000110598	Gm45854	20		ENSMUSG00000111684	Gm8543	20
ENSMUSG00000110686	Gm45855	12		ENSMUSG00000094463	Gm8546	20
ENSMUSG00000109243	Gm45867	20		ENSMUSG00000047905	Gm8566	20
ENSMUSG00000110390	Gm45869	20		ENSMUSG00000024437	Gm8615	20
ENSMUSG00000110277	Gm45871	20		ENSMUSG00000083705	Gm8624	12
ENSMUSG00000110142	Gm45872	12		ENSMUSG00000048709	Gm8666	20

ENSMUSG00000110499	Gm45873	19		ENSMUSG00000050157	Gm867	20
ENSMUSG00000110486	Gm45877	20		ENSMUSG00000083586	Gm8688	18
ENSMUSG00000110366	Gm45885	20		ENSMUSG00000113743	Gm8712	20
ENSMUSG00000110615	Gm45890	20		ENSMUSG00000117935	Gm8717	20
ENSMUSG00000110525	Gm45893	16		ENSMUSG00000081164	Gm8722	20
ENSMUSG00000110440	Gm45894	20		ENSMUSG00000063696	Gm8730	20
ENSMUSG00000051396	Gm45902	20		ENSMUSG00000092540	Gm8738	19
ENSMUSG00000082394	Gm4596	20		ENSMUSG00000091655	Gm8741	20
ENSMUSG00000116529	Gm4600	12		ENSMUSG00000098238	Gm8756	20
ENSMUSG00000078592	Gm4609	12		ENSMUSG00000104360	Gm8762	20
ENSMUSG00000111061	Gm46102	20		ENSMUSG00000091223	Gm8775	20
ENSMUSG00000111443	Gm46123	20		ENSMUSG00000094388	Gm8783	20
ENSMUSG00000111163	Gm46136	20		ENSMUSG00000113936	Gm8784	20
ENSMUSG00000096544	Gm4617	12		ENSMUSG00000103034	Gm8797	12
ENSMUSG00000112146	Gm46210	20		ENSMUSG00000083367	Gm8806	18
ENSMUSG00000110830	Gm46218	20		ENSMUSG00000085625	Gm8813	20
ENSMUSG00000112994	Gm46312	20		ENSMUSG00000082319	Gm8822	18
ENSMUSG00000113959	Gm46339	20		ENSMUSG00000098028	Gm8824	20
ENSMUSG00000112606	Gm46364	20		ENSMUSG00000104181	Gm8850	20
ENSMUSG00000113427	Gm46378	20		ENSMUSG00000084029	Gm8855	20
ENSMUSG00000113440	Gm46404	20		ENSMUSG00000081892	Gm8864	20
ENSMUSG00000114851	Gm46409	20		ENSMUSG00000060680	Gm8894	20
ENSMUSG00000114903	Gm46419	2		ENSMUSG00000111847	Gm8899	20
ENSMUSG00000113204	Gm46430	20		ENSMUSG00000092464	Gm8902	20
ENSMUSG00000114827	Gm46432	20		ENSMUSG00000098187	Gm8927	20
ENSMUSG00000113379	Gm46436	20		ENSMUSG00000117810	Gm8934	12
ENSMUSG00000114516	Gm46440	20		ENSMUSG00000111964	Gm8942	2
ENSMUSG00000115813	Gm46447	20		ENSMUSG00000112099	Gm8960	6
ENSMUSG00000098111	Gm4654	20		ENSMUSG00000114321	Gm8971	20
ENSMUSG00000116845	Gm46546	20		ENSMUSG00000114500	Gm8983	20
ENSMUSG00000116581	Gm46565	20		ENSMUSG00000114607	Gm8990	20
ENSMUSG00000087636	Gm4660	20		ENSMUSG00000059179	Gm8991	12
ENSMUSG00000118012	Gm46620	20		ENSMUSG00000063286	Gm8995	20
ENSMUSG00000117356	Gm46633	20		ENSMUSG00000080824	Gm9001	20
ENSMUSG00000117713	Gm46637	20		ENSMUSG00000087286	Gm9013	20
ENSMUSG00000097750	Gm4673	1		ENSMUSG00000089685	Gm9017	20
ENSMUSG00000115146	Gm4681	20		ENSMUSG00000112614	Gm9030	20
ENSMUSG00000097979	Gm4691	20		ENSMUSG00000114409	Gm9042	20
ENSMUSG00000112836	Gm47006	12		ENSMUSG00000105156	Gm9057	20
ENSMUSG00000113981	Gm47011	5		ENSMUSG00000117939	Gm9067	20
ENSMUSG00000112340	Gm47016	9		ENSMUSG00000098138	Gm9081	20
ENSMUSG00000112295	Gm47017	20		ENSMUSG00000061242	Gm9104	20
ENSMUSG00000112103	Gm47018	20		ENSMUSG00000081370	Gm9105	20
ENSMUSG00000112142	Gm47022	20		ENSMUSG00000112825	Gm9118	20
ENSMUSG00000111935	Gm47023	20		ENSMUSG00000104218	Gm9134	20
ENSMUSG00000112287	Gm47024	2		ENSMUSG00000073233	Gm9144	20
ENSMUSG00000112106	Gm47026	20		ENSMUSG00000109326	Gm9165	19
ENSMUSG00000111931	Gm47032	2		ENSMUSG00000098198	Gm9169	20
ENSMUSG00000111990	Gm47033	1		ENSMUSG00000112850	Gm9176	20
ENSMUSG00000093686	Gm4705	20		ENSMUSG00000083595	Gm9200	20
ENSMUSG00000111498	Gm47059	9		ENSMUSG00000110562	Gm9204	20
ENSMUSG00000091831	Gm4707	2		ENSMUSG00000104642	Gm9207	20
ENSMUSG00000114493	Gm47071	20		ENSMUSG00000058050	Gm9234	12
ENSMUSG00000114169	Gm47075	10		ENSMUSG00000105599	Gm9238	20

ENSMUSG00000110790	Gm47079	20		ENSMUSG00000115505	Gm9247	20
ENSMUSG00000117187	Gm4708	20		ENSMUSG00000105928	Gm9256	20
ENSMUSG00000111212	Gm47087	2		ENSMUSG00000118377	Gm9276	20
ENSMUSG00000113076	Gm47088	9		ENSMUSG00000044609	Gm9294	20
ENSMUSG00000111223	Gm47089	20		ENSMUSG00000108381	Gm9299	20
ENSMUSG00000110738	Gm47092	20		ENSMUSG00000100865	Gm9320	20
ENSMUSG00000111844	Gm47097	2		ENSMUSG00000108702	Gm9333	20
ENSMUSG00000112838	Gm47098	20		ENSMUSG00000110126	Gm9347	20
ENSMUSG00000111341	Gm47111	20		ENSMUSG00000106619	Gm9353	20
ENSMUSG00000111079	Gm47112	20		ENSMUSG00000109059	Gm9354	20
ENSMUSG00000111465	Gm47113	20		ENSMUSG00000080848	Gm9385	12
ENSMUSG00000114244	Gm47119	20		ENSMUSG00000074236	Gm9387	20
ENSMUSG00000111434	Gm47121	12		ENSMUSG00000087298	Gm9392	20
ENSMUSG00000111003	Gm47122	8		ENSMUSG00000063328	Gm9396	20
ENSMUSG00000113610	Gm47132	20		ENSMUSG00000106499	Gm9403	1
ENSMUSG00000111837	Gm47135	18		ENSMUSG00000104241	Gm9442	20
ENSMUSG00000113003	Gm47138	20		ENSMUSG00000105006	Gm9484	20
ENSMUSG00000113106	Gm47139	3		ENSMUSG00000111530	Gm9487	20
ENSMUSG00000111325	Gm47140	20		ENSMUSG00000044424	Gm9493	12
ENSMUSG00000114019	Gm47155	9		ENSMUSG00000113389	Gm9512	3
ENSMUSG00000111490	Gm47159	20		ENSMUSG00000117252	Gm9514	20
ENSMUSG00000111977	Gm47163	20		ENSMUSG00000104103	Gm9517	20
ENSMUSG00000113067	Gm47166	20		ENSMUSG00000098021	Gm9522	20
ENSMUSG00000111340	Gm47171	20		ENSMUSG00000106181	Gm9523	20
ENSMUSG00000111343	Gm47175	20		ENSMUSG00000116580	Gm9525	20
ENSMUSG00000113664	Gm47184	20		ENSMUSG00000096950	Gm9530	20
ENSMUSG00000110959	Gm47198	9		ENSMUSG00000079225	Gm9531	12
ENSMUSG00000113706	Gm47201	20		ENSMUSG00000114558	Gm9570	20
ENSMUSG00000110980	Gm47204	20		ENSMUSG00000083692	Gm9575	20
ENSMUSG00000111312	Gm47205	20		ENSMUSG00000071691	Gm960	20
ENSMUSG00000112190	Gm47209	20		ENSMUSG00000114003	Gm9616	20
ENSMUSG00000113647	Gm47210	20		ENSMUSG00000097906	Gm9625	9
ENSMUSG00000112008	Gm47218	20		ENSMUSG00000098002	Gm9658	20
ENSMUSG00000111030	Gm47234	20		ENSMUSG00000082383	Gm9670	5
ENSMUSG00000114535	Gm47246	20		ENSMUSG00000063001	Gm9701	9
ENSMUSG00000114016	Gm47258	3		ENSMUSG00000082741	Gm9703	19
ENSMUSG00000113328	Gm47260	5		ENSMUSG00000037982	Gm9725	20
ENSMUSG00000110826	Gm47262	20		ENSMUSG00000094935	Gm9726	20
ENSMUSG00000111722	Gm47270	20		ENSMUSG00000089628	Gm9727	20
ENSMUSG00000096768	Gm47283	20		ENSMUSG00000047361	Gm973	20
ENSMUSG00000113789	Gm47322	20		ENSMUSG00000115483	Gm9732	20
ENSMUSG00000111425	Gm47324	20		ENSMUSG00000024658	Gm9750	20
ENSMUSG00000112556	Gm47339	20		ENSMUSG00000029360	Gm9754	1
ENSMUSG00000106208	Gm4734	20		ENSMUSG00000030735	Gm9755	20
ENSMUSG00000112595	Gm47340	20		ENSMUSG00000034437	Gm9761	20
ENSMUSG00000112741	Gm47341	20		ENSMUSG00000037096	Gm9762	20
ENSMUSG00000112243	Gm47342	20		ENSMUSG00000108391	Gm9768	9
ENSMUSG00000064193	Gm4735	20		ENSMUSG00000040540	Gm9770	20
ENSMUSG00000111435	Gm47358	9		ENSMUSG00000042165	Gm9774	20
ENSMUSG00000048087	Gm4737	20		ENSMUSG00000042857	Gm9776	10
ENSMUSG00000113656	Gm47370	20		ENSMUSG00000044330	Gm9790	12
ENSMUSG00000113766	Gm47374	20		ENSMUSG00000044434	Gm9791	20
ENSMUSG00000112808	Gm4739	20		ENSMUSG00000107176	Gm9794	3
ENSMUSG00000113680	Gm47405	20		ENSMUSG00000045075	Gm9796	20

ENSMUSG00000113502	Gm47411	20		ENSMUSG00000045455	Gm9797	20
ENSMUSG00000114424	Gm47414	20		ENSMUSG00000045799	Gm9800	2
ENSMUSG00000105461	Gm4742	16		ENSMUSG00000045813	Gm9801	12
ENSMUSG00000114095	Gm47424	20		ENSMUSG00000045886	Gm9803	20
ENSMUSG00000110877	Gm47427	20		ENSMUSG00000085783	Gm9816	20
ENSMUSG00000111947	Gm47438	20		ENSMUSG00000095478	Gm9824	20
ENSMUSG00000113606	Gm47441	20		ENSMUSG00000096403	Gm9825	20
ENSMUSG00000111361	Gm47445	20		ENSMUSG00000048538	Gm9826	20
ENSMUSG00000113094	Gm47447	20		ENSMUSG00000048603	Gm9828	15
ENSMUSG00000113047	Gm47469	20		ENSMUSG00000094437	Gm9830	20
ENSMUSG00000112905	Gm47477	7		ENSMUSG00000049230	Gm9833	20
ENSMUSG00000111496	Gm47483	9		ENSMUSG00000081071	Gm9836	20
ENSMUSG00000113529	Gm47484	9		ENSMUSG00000050299	Gm9843	12
ENSMUSG00000112947	Gm47493	9		ENSMUSG00000091955	Gm9844	12
ENSMUSG00000113269	Gm47494	20		ENSMUSG00000050533	Gm9845	20
ENSMUSG00000110840	Gm47496	20		ENSMUSG00000085666	Gm9855	20
ENSMUSG00000087235	Gm4750	20		ENSMUSG00000110945	Gm9856	20
ENSMUSG00000111491	Gm47503	20		ENSMUSG00000094002	Gm9866	19
ENSMUSG00000111466	Gm47504	20		ENSMUSG00000104516	Gm9884	20
ENSMUSG00000114440	Gm47505	5		ENSMUSG00000052629	Gm9885	20
ENSMUSG00000110791	Gm47508	20		ENSMUSG00000052673	Gm9887	20
ENSMUSG00000097274	Gm47512	20		ENSMUSG00000052825	Gm9892	20
ENSMUSG00000113320	Gm47515	20		ENSMUSG00000118038	Gm9895	16
ENSMUSG00000106895	Gm4754	20		ENSMUSG00000053214	Gm9899	20
ENSMUSG00000113519	Gm47541	1		ENSMUSG00000097271	Gm9903	20
ENSMUSG00000114707	Gm47558	20		ENSMUSG00000053358	Gm9905	20
ENSMUSG00000112922	Gm47572	16		ENSMUSG00000103469	Gm9910	20
ENSMUSG00000112847	Gm47573	10		ENSMUSG00000109874	Gm9911	20
ENSMUSG00000112317	Gm47580	1		ENSMUSG00000104178	Gm9916	18
ENSMUSG00000113853	Gm47583	20		ENSMUSG00000097099	Gm9917	20
ENSMUSG00000113708	Gm47584	3		ENSMUSG00000053830	Gm9923	20
ENSMUSG00000114104	Gm47585	12		ENSMUSG00000053861	Gm9925	20
ENSMUSG00000113326	Gm47586	10		ENSMUSG00000053925	Gm9929	1
ENSMUSG00000113126	Gm47588	20		ENSMUSG00000053980	Gm9930	20
ENSMUSG00000112601	Gm47590	7		ENSMUSG00000054061	Gm9934	20
ENSMUSG00000112153	Gm47591	9		ENSMUSG00000054247	Gm9939	20
ENSMUSG00000112015	Gm47592	8		ENSMUSG00000054450	Gm9945	20
ENSMUSG00000112255	Gm47594	20		ENSMUSG00000054488	Gm9946	20
ENSMUSG00000112759	Gm47615	20		ENSMUSG00000054493	Gm9947	20
ENSMUSG00000112576	Gm47621	20		ENSMUSG00000104868	Gm9954	20
ENSMUSG00000112681	Gm47622	20		ENSMUSG00000054945	Gm9958	10
ENSMUSG00000112257	Gm47623	20		ENSMUSG00000055048	Gm9962	20
ENSMUSG00000111918	Gm47624	20		ENSMUSG00000055323	Gm9967	20
ENSMUSG00000111421	Gm47643	20		ENSMUSG00000055497	Gm9974	20
ENSMUSG00000112734	Gm47644	20		ENSMUSG00000055958	Gm9987	20
ENSMUSG00000111481	Gm47652	20		ENSMUSG00000056023	Gm9989	12
ENSMUSG00000113980	Gm47655	20		ENSMUSG00000117505	Gm9993	20
ENSMUSG00000113557	Gm47657	9		ENSMUSG00000056316	Gm9996	20
ENSMUSG00000113993	Gm47659	20		ENSMUSG00000001157	Gmcl1	20
ENSMUSG00000113183	Gm47664	20		ENSMUSG00000038372	Gmds	20
ENSMUSG00000113649	Gm47665	9		ENSMUSG00000028901	Gmeb1	20
ENSMUSG00000111065	Gm47676	20		ENSMUSG00000038705	Gmeb2	12
ENSMUSG00000112254	Gm47690	12		ENSMUSG00000062014	Gmfb	20
ENSMUSG00000112338	Gm47693	13		ENSMUSG00000060791	Gmfg	20

ENSMUSG00000114584	Gm47694	20		ENSMUSG00000036246	Gmip	20
ENSMUSG00000112075	Gm47700	20		ENSMUSG00000068428	Gmnc	13
ENSMUSG00000114148	Gm47701	20		ENSMUSG00000006715	Gmnn	20
ENSMUSG00000112914	Gm47702	20		ENSMUSG00000033021	Gmppa	20
ENSMUSG00000112928	Gm47710	19		ENSMUSG00000070284	Gmppb	20
ENSMUSG00000112468	Gm47720	20		ENSMUSG00000000253	Gmpr	9
ENSMUSG00000112879	Gm47726	20		ENSMUSG00000002326	Gmpr2	20
ENSMUSG00000114121	Gm47728	20		ENSMUSG00000027823	Gmps	3
ENSMUSG00000112830	Gm47765	20		ENSMUSG00000034781	Gna11	20
ENSMUSG00000114285	Gm47773	20		ENSMUSG00000000149	Gna12	16
ENSMUSG00000114907	Gm47794	13		ENSMUSG00000020611	Gna13	16
ENSMUSG00000114253	Gm47798	6		ENSMUSG00000024697	Gna14	20
ENSMUSG00000112036	Gm47801	5		ENSMUSG00000034792	Gna15	20
ENSMUSG00000112070	Gm47809	20		ENSMUSG00000057614	Gnai1	1
ENSMUSG00000114575	Gm47814	20		ENSMUSG00000032562	Gnai2	7
ENSMUSG00000113752	Gm47817	2		ENSMUSG00000000001	Gnai3	20
ENSMUSG00000113072	Gm47820	20		ENSMUSG00000024524	Gnal	9
ENSMUSG00000113650	Gm47826	20		ENSMUSG00000031748	Gnao1	9
ENSMUSG00000078308	Gm47854	20		ENSMUSG00000024639	Gnaq	2
ENSMUSG00000110772	Gm47856	3		ENSMUSG00000027523	Gnas	6
ENSMUSG00000110903	Gm47857	20		ENSMUSG00000086537	Gnasas1	20
ENSMUSG00000114083	Gm47860	3		ENSMUSG00000040009	Gnaz	2
ENSMUSG00000111943	Gm47862	20		ENSMUSG00000029064	Gnb1	20
ENSMUSG00000113165	Gm47863	20		ENSMUSG00000000884	Gnb1l	20
ENSMUSG00000072974	Gm4787	20		ENSMUSG00000029713	Gnb2	3
ENSMUSG00000112248	Gm47910	20		ENSMUSG00000023439	Gnb3	20
ENSMUSG00000112369	Gm47911	1		ENSMUSG00000027669	Gnb4	1
ENSMUSG00000114731	Gm47914	20		ENSMUSG00000032192	Gnb5	9
ENSMUSG00000114709	Gm47920	20		ENSMUSG00000028479	Gne	20
ENSMUSG00000054412	Gm4793	18		ENSMUSG00000038607	Gng10	12
ENSMUSG00000114721	Gm47938	20		ENSMUSG00000032766	Gng11	20
ENSMUSG00000111793	Gm47950	20		ENSMUSG00000036402	Gng12	2
ENSMUSG00000112000	Gm47956	3		ENSMUSG00000025739	Gng13	12
ENSMUSG00000110993	Gm47963	3		ENSMUSG00000043004	Gng2	19
ENSMUSG00000112300	Gm47976	20		ENSMUSG00000071658	Gng3	8
ENSMUSG00000112714	Gm4798	20		ENSMUSG00000021303	Gng4	16
ENSMUSG00000113966	Gm47980	20		ENSMUSG00000068523	Gng5	9
ENSMUSG00000113288	Gm47982	20		ENSMUSG00000048240	Gng7	9
ENSMUSG00000113698	Gm47987	20		ENSMUSG00000063594	Gng8	20
ENSMUSG00000113808	Gm47988	20		ENSMUSG00000038811	Gngt2	12
ENSMUSG00000071151	Gm4799	20		ENSMUSG00000024429	Gnl1	20
ENSMUSG00000114399	Gm47994	20		ENSMUSG00000028869	Gnl2	20
ENSMUSG00000112756	Gm48014	20		ENSMUSG00000042354	Gnl3	20
ENSMUSG00000112067	Gm48015	20		ENSMUSG00000025266	Gnl3l	19
ENSMUSG00000112745	Gm48025	20		ENSMUSG00000002769	Gnmt	6
ENSMUSG00000113775	Gm48027	20		ENSMUSG00000031985	Gnpat	1
ENSMUSG00000098158	Gm4804	20		ENSMUSG00000052102	Gnpda1	20
ENSMUSG00000111905	Gm48045	20		ENSMUSG00000029209	Gnpda2	20
ENSMUSG00000114102	Gm48048	20		ENSMUSG00000037722	Gnpnat1	20
ENSMUSG00000111098	Gm48057	9		ENSMUSG00000035311	Gnptab	20
ENSMUSG00000113852	Gm48062	20		ENSMUSG00000035521	Gnptg	3
ENSMUSG00000113020	Gm48063	20		ENSMUSG00000015812	Gnrh1	20
ENSMUSG00000114254	Gm48067	20		ENSMUSG00000034707	Gns	20
ENSMUSG00000111547	Gm48069	20		ENSMUSG00000026754	Golga1	20

ENSMUSG00000112875	Gm48074	20		ENSMUSG00000002546	Golga2	20
ENSMUSG00000114293	Gm48078	20		ENSMUSG00000029502	Golga3	20
ENSMUSG00000113527	Gm48079	20		ENSMUSG00000038708	Golga4	20
ENSMUSG00000111928	Gm48082	20		ENSMUSG00000021192	Golga5	20
ENSMUSG00000112229	Gm48086	19		ENSMUSG00000015341	Golga7	16
ENSMUSG00000110758	Gm48092	20		ENSMUSG00000042532	Golga7b	20
ENSMUSG00000113263	Gm4811	20		ENSMUSG00000034243	Golgb1	20
ENSMUSG00000110928	Gm48114	3		ENSMUSG00000034109	Golim4	18
ENSMUSG00000111934	Gm48123	20		ENSMUSG00000021556	Golm1	5
ENSMUSG00000110832	Gm48125	20		ENSMUSG00000022200	Golph3	2
ENSMUSG00000111271	Gm48127	20		ENSMUSG00000046519	Golph3l	20
ENSMUSG00000111323	Gm48129	12		ENSMUSG00000030245	Golt1b	20
ENSMUSG00000114822	Gm4813	20		ENSMUSG00000054199	Gon4l	9
ENSMUSG00000113571	Gm48137	20		ENSMUSG00000091931	Gon7	20
ENSMUSG00000113842	Gm48138	1		ENSMUSG00000019861	Gopc	20
ENSMUSG00000114193	Gm48139	20		ENSMUSG00000040124	Gorab	20
ENSMUSG00000112806	Gm48146	20		ENSMUSG00000032513	Gorasp1	20
ENSMUSG00000113223	Gm48147	20		ENSMUSG00000014959	Gorasp2	1
ENSMUSG00000112056	Gm48181	20		ENSMUSG00000010392	Gosr1	20
ENSMUSG00000112448	Gm48182	20		ENSMUSG00000020946	Gosr2	3
ENSMUSG00000111756	Gm48183	4		ENSMUSG00000025190	Got1	20
ENSMUSG00000114835	Gm48194	20		ENSMUSG00000039720	Got1l1	20
ENSMUSG00000112512	Gm48203	20		ENSMUSG00000031672	Got2	9
ENSMUSG00000112900	Gm48204	20		ENSMUSG00000080935	Got2-ps1	9
ENSMUSG00000112571	Gm48207	20		ENSMUSG00000050761	Gp1bb	20
ENSMUSG00000112637	Gm48225	20		ENSMUSG00000022561	Gpaa1	20
ENSMUSG00000112302	Gm48226	20		ENSMUSG00000022008	Gpalpp1	20
ENSMUSG00000112391	Gm48230	20		ENSMUSG00000024978	Gpam	20
ENSMUSG00000112090	Gm48231	2		ENSMUSG00000092417	Gpank1	2
ENSMUSG00000111211	Gm48233	20		ENSMUSG00000046338	Gpat2	6
ENSMUSG00000114230	Gm48239	20		ENSMUSG00000029314	Gpat3	20
ENSMUSG00000115593	Gm4824	12		ENSMUSG00000031545	Gpat4	1
ENSMUSG00000111447	Gm48249	20		ENSMUSG00000063808	Gpatch1	9
ENSMUSG00000114812	Gm48254	20		ENSMUSG00000050668	Gpatch11	20
ENSMUSG00000113810	Gm48259	19		ENSMUSG00000039210	Gpatch2	2
ENSMUSG00000113073	Gm48261	19		ENSMUSG00000021254	Gpatch2l	20
ENSMUSG00000113742	Gm48262	19		ENSMUSG00000028850	Gpatch3	20
ENSMUSG00000114626	Gm48266	20		ENSMUSG00000028069	Gpatch4	20
ENSMUSG00000114624	Gm48267	5		ENSMUSG00000034621	Gpatch8	20
ENSMUSG00000112074	Gm48278	20		ENSMUSG00000032745	Gpbp1	20
ENSMUSG00000112060	Gm48279	20		ENSMUSG00000034042	Gpbp1l1	12
ENSMUSG00000116620	Gm4828	12		ENSMUSG00000034220	Gpc1	9
ENSMUSG00000112589	Gm48280	20		ENSMUSG00000029510	Gpc2	20
ENSMUSG00000112059	Gm48282	20		ENSMUSG00000055653	Gpc3	6
ENSMUSG00000111504	Gm48284	2		ENSMUSG00000031119	Gpc4	5
ENSMUSG00000113910	Gm48286	20		ENSMUSG00000022112	Gpc5	1
ENSMUSG00000116779	Gm4829	20		ENSMUSG00000058571	Gpc6	1
ENSMUSG00000111794	Gm48294	20		ENSMUSG00000027346	Gpcpd1	8
ENSMUSG00000114309	Gm48295	20		ENSMUSG00000023019	Gpd1	20
ENSMUSG00000112820	Gm48298	20		ENSMUSG00000050627	Gpd1l	1
ENSMUSG00000113041	Gm48302	20		ENSMUSG00000026827	Gpd2	3
ENSMUSG00000111017	Gm48314	1		ENSMUSG00000053647	Gper1	20
ENSMUSG00000113391	Gm48327	20		ENSMUSG00000047454	Gphn	20
ENSMUSG00000113588	Gm48328	20		ENSMUSG00000036427	Gpi1	8

ENSMUSG00000117428	Gm4833	20		ENSMUSG00000031148	Gpkow	20
ENSMUSG00000112927	Gm48332	20		ENSMUSG00000021340	Gpld1	9
ENSMUSG00000111442	Gm48334	20		ENSMUSG00000031517	Gpm6a	4
ENSMUSG00000110894	Gm48335	1		ENSMUSG00000031342	Gpm6b	9
ENSMUSG00000114934	Gm48342	20		ENSMUSG00000064037	Gpn1	20
ENSMUSG00000113191	Gm48343	20		ENSMUSG00000028848	Gpn2	20
ENSMUSG00000113871	Gm48349	20		ENSMUSG00000029464	Gpn3	20
ENSMUSG00000043223	Gm4835	20		ENSMUSG00000029816	Gpnmb	1
ENSMUSG00000114014	Gm48350	20		ENSMUSG00000046856	Gpr1	20
ENSMUSG00000112309	Gm48353	13		ENSMUSG00000036357	Gpr101	20
ENSMUSG00000114524	Gm48357	20		ENSMUSG00000000194	Gpr107	20
ENSMUSG00000113395	Gm48365	12		ENSMUSG00000005823	Gpr108	20
ENSMUSG00000113349	Gm48369	20		ENSMUSG00000041468	Gpr12	20
ENSMUSG00000111539	Gm48372	20		ENSMUSG00000043398	Gpr135	1
ENSMUSG00000114094	Gm48375	20		ENSMUSG00000024958	Gpr137	18
ENSMUSG00000113864	Gm48381	20		ENSMUSG00000021306	Gpr137b	20
ENSMUSG00000113917	Gm48382	20		ENSMUSG00000097715	Gpr137b-ps	20
ENSMUSG00000110901	Gm48393	20		ENSMUSG00000049092	Gpr137c	18
ENSMUSG00000118282	Gm4840	20		ENSMUSG00000066197	Gpr139	8
ENSMUSG00000113385	Gm48405	20		ENSMUSG00000044197	Gpr146	20
ENSMUSG00000113309	Gm48408	20		ENSMUSG00000043441	Gpr149	20
ENSMUSG00000113070	Gm48420	20		ENSMUSG00000045509	Gpr150	20
ENSMUSG00000114749	Gm48442	20		ENSMUSG00000042804	Gpr153	18
ENSMUSG00000111485	Gm48443	6		ENSMUSG00000041762	Gpr155	9
ENSMUSG00000111424	Gm48478	20		ENSMUSG00000046961	Gpr156	18
ENSMUSG00000113167	Gm48482	20		ENSMUSG00000047875	Gpr157	20
ENSMUSG00000112666	Gm48485	1		ENSMUSG00000045967	Gpr158	9
ENSMUSG00000100204	Gm4849	20		ENSMUSG00000037661	Gpr160	20
ENSMUSG00000113889	Gm48501	20		ENSMUSG00000040836	Gpr161	5
ENSMUSG00000112112	Gm48508	1		ENSMUSG00000038390	Gpr162	9
ENSMUSG00000112655	Gm48511	9		ENSMUSG00000031210	Gpr165	9
ENSMUSG00000081185	Gm4852	20		ENSMUSG00000052229	Gpr17	9
ENSMUSG00000111521	Gm48529	20		ENSMUSG00000056679	Gpr173	20
ENSMUSG00000112020	Gm48532	20		ENSMUSG00000073008	Gpr174	20
ENSMUSG00000112169	Gm48536	20		ENSMUSG00000040133	Gpr176	20
ENSMUSG00000112483	Gm48542	9		ENSMUSG00000070337	Gpr179	9
ENSMUSG00000112420	Gm48543	20		ENSMUSG00000022131	Gpr180	20
ENSMUSG00000113262	Gm48551	20		ENSMUSG00000058396	Gpr182	20
ENSMUSG00000113222	Gm48557	20		ENSMUSG00000051212	Gpr183	20
ENSMUSG00000111345	Gm48562	20		ENSMUSG00000032641	Gpr19	20
ENSMUSG00000114773	Gm48568	1		ENSMUSG00000053164	Gpr21	1
ENSMUSG00000114501	Gm48582	2		ENSMUSG00000044067	Gpr22	5
ENSMUSG00000114277	Gm48583	20		ENSMUSG00000052759	Gpr25	20
ENSMUSG00000113250	Gm48585	7		ENSMUSG00000040125	Gpr26	9
ENSMUSG00000113195	Gm48586	6		ENSMUSG00000072875	Gpr27	20
ENSMUSG00000113505	Gm48593	20		ENSMUSG00000049649	Gpr3	20
ENSMUSG00000114267	Gm48600	20		ENSMUSG00000040229	Gpr34	6
ENSMUSG00000114431	Gm48601	12		ENSMUSG00000026271	Gpr35	20
ENSMUSG00000114310	Gm48602	20		ENSMUSG00000039904	Gpr37	16
ENSMUSG00000111291	Gm48604	20		ENSMUSG00000026424	Gpr37l1	20
ENSMUSG00000110914	Gm48611	20		ENSMUSG00000026343	Gpr39	9
ENSMUSG00000113045	Gm48621	20		ENSMUSG00000044317	Gpr4	18
ENSMUSG00000113404	Gm48622	20		ENSMUSG00000041907	Gpr45	1
ENSMUSG00000112941	Gm48623	17		ENSMUSG00000118401	Gpr52	9

ENSMUSG00000113140	Gm48624	20		ENSMUSG00000046922	Gpr6	9
ENSMUSG00000111292	Gm48627	20		ENSMUSG00000046793	Gpr61	20
ENSMUSG00000113200	Gm48632	20		ENSMUSG00000091735	Gpr62	18
ENSMUSG00000114664	Gm48639	20		ENSMUSG00000040372	Gpr63	20
ENSMUSG00000111069	Gm48646	20		ENSMUSG00000021886	Gpr65	20
ENSMUSG00000104777	Gm4865	20		ENSMUSG00000047415	Gpr68	19
ENSMUSG00000113425	Gm48653	20		ENSMUSG00000043999	Gpr75	20
ENSMUSG00000112393	Gm48655	12		ENSMUSG00000031932	Gpr83	9
ENSMUSG00000111505	Gm48673	20		ENSMUSG00000063234	Gpr84	20
ENSMUSG00000111971	Gm48678	9		ENSMUSG00000048216	Gpr85	1
ENSMUSG00000113623	Gm48691	10		ENSMUSG00000068696	Gpr88	9
ENSMUSG00000114153	Gm48692	7		ENSMUSG00000028096	Gpr89	20
ENSMUSG00000113812	Gm48699	19		ENSMUSG00000043384	Gprasp1	9
ENSMUSG00000113055	Gm48700	1		ENSMUSG00000072966	Gprasp2	9
ENSMUSG00000113841	Gm48701	20		ENSMUSG00000008734	Gprc5b	16
ENSMUSG00000114555	Gm48706	3		ENSMUSG00000051043	Gprc5c	13
ENSMUSG00000113154	Gm48713	19		ENSMUSG00000069227	Gprin1	5
ENSMUSG00000111067	Gm48714	20		ENSMUSG00000071531	Gprin2	20
ENSMUSG00000111254	Gm48717	1		ENSMUSG00000045441	Gprin3	9
ENSMUSG00000112736	Gm48718	20		ENSMUSG00000025156	Gps1	3
ENSMUSG00000111652	Gm48726	20		ENSMUSG00000023170	Gps2	19
ENSMUSG00000113078	Gm48734	20		ENSMUSG00000026930	Gpsm1	2
ENSMUSG00000113601	Gm48735	20		ENSMUSG00000027883	Gpsm2	18
ENSMUSG00000111107	Gm48737	20		ENSMUSG00000034786	Gpsm3	20
ENSMUSG00000111626	Gm48738	20		ENSMUSG00000022546	Gpt	2
ENSMUSG00000110885	Gm48739	20		ENSMUSG00000031700	Gpt2	20
ENSMUSG00000111077	Gm48743	20		ENSMUSG00000063856	Gpx1	5
ENSMUSG00000112543	Gm48744	20		ENSMUSG00000018339	Gpx3	6
ENSMUSG00000112019	Gm48748	20		ENSMUSG00000075706	Gpx4	12
ENSMUSG00000082791	Gm4875	20		ENSMUSG00000110841	Gpx4-ps2	12
ENSMUSG00000112307	Gm48751	20		ENSMUSG00000004341	Gpx6	1
ENSMUSG00000111951	Gm48755	18		ENSMUSG00000028597	Gpx7	20
ENSMUSG00000112162	Gm48758	20		ENSMUSG00000021760	Gpx8	13
ENSMUSG00000054556	Gm4876	20		ENSMUSG00000001248	Gramd1a	12
ENSMUSG00000114617	Gm48766	20		ENSMUSG00000040111	Gramd1b	12
ENSMUSG00000112346	Gm48768	20		ENSMUSG00000036292	Gramd1c	20
ENSMUSG00000113159	Gm48771	9		ENSMUSG00000074259	Gramd2	20
ENSMUSG00000111929	Gm48780	20		ENSMUSG00000001700	Gramd3	20
ENSMUSG00000110727	Gm48784	20		ENSMUSG00000035900	Gramd4	20
ENSMUSG00000111228	Gm48789	20		ENSMUSG00000004837	Grap	12
ENSMUSG00000111390	Gm48796	20		ENSMUSG00000042351	Grap2	9
ENSMUSG00000112816	Gm48798	20		ENSMUSG00000000531	Grasp	6
ENSMUSG00000112596	Gm48804	7		ENSMUSG00000020176	Grb10	17
ENSMUSG00000112033	Gm48808	20		ENSMUSG00000026888	Grb14	9
ENSMUSG00000110895	Gm48822	20		ENSMUSG00000059923	Grb2	18
ENSMUSG00000114863	Gm48824	20		ENSMUSG00000019312	Grb7	20
ENSMUSG00000113894	Gm48838	9		ENSMUSG00000072772	Grcc10	12
ENSMUSG00000111664	Gm48865	20		ENSMUSG00000036523	Greb1	9
ENSMUSG00000113869	Gm48869	20		ENSMUSG00000042942	Greb1l	9
ENSMUSG00000086229	Gm4887	20		ENSMUSG00000074934	Grem1	2
ENSMUSG00000113065	Gm48870	20		ENSMUSG00000050069	Grem2	20
ENSMUSG00000112948	Gm48871	20		ENSMUSG00000020656	Grhl1	5
ENSMUSG00000113905	Gm48872	20		ENSMUSG00000037188	Grhl3	20
ENSMUSG00000112744	Gm48880	20		ENSMUSG00000035637	Grhpr	7

ENSMUSG00000113119	Gm48883	20		ENSMUSG00000020524	Gria1	18
ENSMUSG00000112944	Gm48885	20		ENSMUSG00000033981	Gria2	18
ENSMUSG00000113922	Gm48887	4		ENSMUSG00000001986	Gria3	9
ENSMUSG00000112955	Gm48889	20		ENSMUSG00000025892	Gria4	18
ENSMUSG00000113984	Gm48890	18		ENSMUSG00000041078	Grid1	1
ENSMUSG00000111950	Gm48893	20		ENSMUSG00000071424	Grid2	2
ENSMUSG00000114887	Gm48894	19		ENSMUSG00000010825	Grid2ip	18
ENSMUSG00000112441	Gm48898	20		ENSMUSG00000022935	Grik1	6
ENSMUSG00000097174	Gm4890	20		ENSMUSG00000056073	Grik2	9
ENSMUSG00000114081	Gm48904	20		ENSMUSG00000001985	Grik3	9
ENSMUSG00000113886	Gm48905	20		ENSMUSG00000032017	Grik4	5
ENSMUSG00000115545	Gm48908	20		ENSMUSG00000003378	Grik5	9
ENSMUSG00000115194	Gm48909	20		ENSMUSG00000026959	Grin1	9
ENSMUSG00000115339	Gm48913	20		ENSMUSG00000085830	Grin1os	20
ENSMUSG00000113080	Gm48928	20		ENSMUSG00000059003	Grin2a	5
ENSMUSG00000115360	Gm48932	1		ENSMUSG00000030209	Grin2b	9
ENSMUSG00000115772	Gm48935	20		ENSMUSG00000020734	Grin2c	16
ENSMUSG00000115389	Gm48936	10		ENSMUSG00000002771	Grin2d	18
ENSMUSG00000115041	Gm48939	20		ENSMUSG00000039579	Grin3a	18
ENSMUSG00000115228	Gm48948	20		ENSMUSG00000035745	Grin3b	20
ENSMUSG00000115417	Gm48949	20		ENSMUSG00000022564	Grina	20
ENSMUSG00000044268	Gm4895	20		ENSMUSG00000034813	Grip1	20
ENSMUSG00000115742	Gm48950	3		ENSMUSG00000030098	Grip2	18
ENSMUSG00000115729	Gm48957	12		ENSMUSG00000031153	Gripap1	9
ENSMUSG00000114996	Gm48958	3		ENSMUSG00000024858	Grk2	9
ENSMUSG00000115793	Gm48961	20		ENSMUSG00000042249	Grk3	19
ENSMUSG00000115340	Gm48965	20		ENSMUSG00000052783	Grk4	20
ENSMUSG00000115314	Gm48966	20		ENSMUSG00000003228	Grk5	20
ENSMUSG00000115023	Gm48967	20		ENSMUSG00000074886	Grk6	20
ENSMUSG00000115044	Gm48978	20		ENSMUSG00000019828	Grm1	18
ENSMUSG00000115570	Gm48986	20		ENSMUSG00000023192	Grm2	5
ENSMUSG00000115650	Gm48992	20		ENSMUSG00000003974	Grm3	9
ENSMUSG00000115755	Gm48993	20		ENSMUSG00000063239	Grm4	1
ENSMUSG00000115252	Gm48996	19		ENSMUSG00000049583	Grm5	9
ENSMUSG00000115087	Gm48998	20		ENSMUSG00000056755	Grm7	7
ENSMUSG00000115509	Gm49012	20		ENSMUSG00000024211	Grm8	20
ENSMUSG00000114278	Gm49027	20		ENSMUSG00000034708	Grn	20
ENSMUSG00000115248	Gm49037	20		ENSMUSG00000024517	Grp	20
ENSMUSG00000107970	Gm49058	20		ENSMUSG00000029198	Grpel1	20
ENSMUSG00000114771	Gm49064	2		ENSMUSG00000024580	Grpel2	20
ENSMUSG00000115810	Gm49066	20		ENSMUSG00000031364	Grpr	20
ENSMUSG00000114970	Gm49069	9		ENSMUSG00000050105	Grrp1	18
ENSMUSG00000044221	Grsf1	16				
ENSMUSG00000038515	Grtp1	20		ENSMUSG00000003283	Hck	20
ENSMUSG00000053801	Grwd1	20		ENSMUSG00000022831	Hcls1	20
ENSMUSG00000039934	Gsap	20		ENSMUSG00000021730	Hcn1	20
ENSMUSG00000022575	Gsdmd	20		ENSMUSG00000020331	Hcn2	18
ENSMUSG00000029821	Gsdme	20		ENSMUSG00000028051	Hcn3	20
ENSMUSG00000031822	Gse1	5		ENSMUSG00000032338	Hcn4	18
ENSMUSG00000046182	Gsg1l	9		ENSMUSG00000028778	Hcrtr1	20
ENSMUSG00000057177	Gsk3a	1		ENSMUSG00000032360	Hcrtr2	20
ENSMUSG00000022812	Gsk3b	8		ENSMUSG00000028800	Hdac1	9
ENSMUSG00000044715	Gskip	20		ENSMUSG00000062906	Hdac10	20
ENSMUSG00000026879	Gsn	16		ENSMUSG00000034245	Hdac11	2

ENSMUSG00000062203	Gspt1	20		ENSMUSG00000019777	Hdac2	18
ENSMUSG00000071723	Gspt2	16		ENSMUSG00000024454	Hdac3	20
ENSMUSG00000031584	Gsr	20		ENSMUSG00000026313	Hdac4	2
ENSMUSG00000027610	Gss	9		ENSMUSG00000008855	Hdac5	12
ENSMUSG00000085317	Gssos2	20		ENSMUSG00000031161	Hdac6	20
ENSMUSG00000025934	Gsta3	20		ENSMUSG00000022475	Hdac7	20
ENSMUSG00000032348	Gsta4	20		ENSMUSG00000067567	Hdac8	20
ENSMUSG00000028018	Gstcd	20		ENSMUSG00000004698	Hdac9	18
ENSMUSG00000029864	Gstk1	20		ENSMUSG00000027360	Hdc	6
ENSMUSG00000058135	Gstm1	1		ENSMUSG00000000295	Hddc2	20
ENSMUSG00000040562	Gstm2	20		ENSMUSG00000030532	Hddc3	20
ENSMUSG00000107369	Gstm2-ps1	20		ENSMUSG00000004897	Hdgf	20
ENSMUSG00000027890	Gstm4	20		ENSMUSG00000002833	Hdgfl2	3
ENSMUSG00000004032	Gstm5	3		ENSMUSG00000025104	Hdgfl3	20
ENSMUSG00000068762	Gstm6	6		ENSMUSG00000025421	Hdhd2	20
ENSMUSG00000004035	Gstm7	2		ENSMUSG00000038422	Hdhd3	10
ENSMUSG00000025068	Gsto1	1		ENSMUSG00000058979	Hdhd5	20
ENSMUSG00000025069	Gsto2	9		ENSMUSG00000034088	Hdlbp	12
ENSMUSG00000001663	Gstt1	20		ENSMUSG00000034551	Hdx	3
ENSMUSG00000033318	Gstt2	9		ENSMUSG00000050244	Heatr1	3
ENSMUSG00000001665	Gstt3	20		ENSMUSG00000031657	Heatr3	20
ENSMUSG00000021033	Gstz1	9		ENSMUSG00000035181	Heatr5a	3
ENSMUSG00000086429	Gt(ROSA)26Sor	20		ENSMUSG00000039414	Heatr5b	20
ENSMUSG00000036890	Gtdc1	5		ENSMUSG00000000976	Heatr6	9
ENSMUSG00000020962	Gtf2a1	1		ENSMUSG00000042770	Hebp1	19
ENSMUSG00000033543	Gtf2a2	20		ENSMUSG00000019853	Hebp2	20
ENSMUSG00000028271	Gtf2b	18		ENSMUSG00000039879	Heca	20
ENSMUSG00000022828	Gtf2e1	20		ENSMUSG00000035247	Hectd1	2
ENSMUSG00000031585	Gtf2e2	3		ENSMUSG00000041180	Hectd2	5
ENSMUSG00000002658	Gtf2f1	20		ENSMUSG00000087579	Hectd2os	9
ENSMUSG00000067995	Gtf2f2	20		ENSMUSG00000046861	Hectd3	20
ENSMUSG00000006599	Gtf2h1	20		ENSMUSG00000042744	Hectd4	19
ENSMUSG00000021639	Gtf2h2	9		ENSMUSG00000021301	Hecw1	19
ENSMUSG00000029387	Gtf2h3	20		ENSMUSG00000042807	Hecw2	7
ENSMUSG00000001524	Gtf2h4	2		ENSMUSG00000075254	Heg1	19
ENSMUSG00000034345	Gtf2h5	20		ENSMUSG00000020228	Helb	20
ENSMUSG00000060261	Gtf2i	3		ENSMUSG00000025001	Hells	20
ENSMUSG00000023079	Gtf2ird1	20		ENSMUSG00000035266	Helq	20
ENSMUSG00000015942	Gtf2ird2	20		ENSMUSG00000020721	Helz	20
ENSMUSG00000016503	Gtf3a	20		ENSMUSG00000027580	Helz2	20
ENSMUSG00000032777	Gtf3c1	7		ENSMUSG00000032579	Hemk1	13
ENSMUSG00000106864	Gtf3c2	20		ENSMUSG00000045662	Henmt1	9
ENSMUSG00000041303	Gtf3c3	12		ENSMUSG00000046240	Hepacam	16
ENSMUSG00000035666	Gtf3c4	20		ENSMUSG00000031209	Heph	20
ENSMUSG00000026816	Gtf3c5	20		ENSMUSG00000038664	Herc1	19
ENSMUSG00000019837	Gtf3c6	20		ENSMUSG00000030451	Herc2	3
ENSMUSG00000042535	Gtpbp1	20		ENSMUSG00000029804	Herc3	9
ENSMUSG00000040464	Gtpbp10	12		ENSMUSG00000020064	Herc4	20
ENSMUSG00000023952	Gtpbp2	4		ENSMUSG00000029798	Herc6	20
ENSMUSG00000007610	Gtpbp3	20		ENSMUSG00000031770	Herpud1	6
ENSMUSG00000021149	Gtpbp4	20		ENSMUSG00000008429	Herpud2	20
ENSMUSG00000033434	Gtpbp6	20		ENSMUSG00000022528	Hes1	20
ENSMUSG00000022668	Gtpbp8	20		ENSMUSG00000048001	Hes5	20
ENSMUSG00000033416	Gucd1	13		ENSMUSG00000067071	Hes6	12

ENSMUSG00000033910	Gucy1a1	9		ENSMUSG00000023781	Hes7	20
ENSMUSG00000041624	Gucy1a2	12		ENSMUSG00000025232	Hexa	20
ENSMUSG00000028005	Gucy1b1	6		ENSMUSG00000021665	Hexb	19
ENSMUSG00000042638	Gucy2c	6		ENSMUSG00000039307	Hexdc	3
ENSMUSG00000020890	Gucy2e	20		ENSMUSG00000048878	Hexim1	3
ENSMUSG00000042282	Gucy2f	9		ENSMUSG00000043372	Hexim2	9
ENSMUSG00000055523	Gucy2g	20		ENSMUSG00000040289	Hey1	20
ENSMUSG00000029208	Guf1	20		ENSMUSG00000019789	Hey2	13
ENSMUSG00000020444	Guk1	12		ENSMUSG00000032744	Heyl	20
ENSMUSG00000056870	Gulp1	20		ENSMUSG00000006611	Hfe	1
ENSMUSG00000025534	Gusb	20		ENSMUSG00000043410	Hfm1	20
ENSMUSG00000036197	Gxylt1	9		ENSMUSG00000028864	Hgf	1
ENSMUSG00000030074	Gxylt2	1		ENSMUSG00000022554	Hgh1	20
ENSMUSG00000019528	Gyg	20		ENSMUSG00000025793	Hgs	20
ENSMUSG00000003865	Gys1	18		ENSMUSG00000037260	Hgsnat	20
ENSMUSG00000027439	Gzf1	8		ENSMUSG00000037375	Hhat	20
ENSMUSG00000042385	Gzmk	20		ENSMUSG00000032523	Hhatl	20
ENSMUSG00000054206	Gzmm	20		ENSMUSG00000064325	Hhip	1
ENSMUSG00000019188	H13	20		ENSMUSG00000021260	Hhipl1	1
ENSMUSG00000096210	H1f0	6		ENSMUSG00000029776	Hibadh	20
ENSMUSG00000044927	H1fx	20		ENSMUSG00000041426	Hibch	20
ENSMUSG00000036594	H2-Aa	20		ENSMUSG00000043099	Hic1	20
ENSMUSG00000073421	H2-Ab1	20		ENSMUSG00000050240	Hic2	1
ENSMUSG00000073406	H2-Bl	6		ENSMUSG00000034586	Hid1	20
ENSMUSG00000073411	H2-D1	20		ENSMUSG00000021109	Hif1a	19
ENSMUSG00000037649	H2-DMa	20		ENSMUSG00000036450	Hif1an	20
ENSMUSG00000079547	H2-DMb1	20		ENSMUSG00000004328	Hif3a	20
ENSMUSG00000037548	H2-DMb2	20		ENSMUSG00000038412	Higd1a	11
ENSMUSG00000060586	H2-Eb1	2		ENSMUSG00000020928	Higd1b	20
ENSMUSG00000061232	H2-K1	11		ENSMUSG00000025868	Higd2a	12
ENSMUSG00000067203	H2-K2	20		ENSMUSG00000062797	Hikeshi	20
ENSMUSG00000073422	H2-Ke6	9		ENSMUSG00000043421	Hilpda	20
ENSMUSG00000016206	H2-M3	20		ENSMUSG00000032119	Hinfp	16
ENSMUSG00000024459	H2-M5	20		ENSMUSG00000020267	Hint1	12
ENSMUSG00000041538	H2-Ob	20		ENSMUSG00000028470	Hint2	3
ENSMUSG00000091705	H2-Q2	20		ENSMUSG00000019791	Hint3	9
ENSMUSG00000035929	H2-Q4	20		ENSMUSG00000039959	Hip1	20
ENSMUSG00000073409	H2-Q6	11		ENSMUSG00000000915	Hip1r	20
ENSMUSG00000060550	H2-Q7	20		ENSMUSG00000008730	Hipk1	20
ENSMUSG00000079491	H2-T10	20		ENSMUSG00000061436	Hipk2	15
ENSMUSG00000056116	H2-T22	11		ENSMUSG00000027177	Hipk3	2
ENSMUSG00000067212	H2-T23	11		ENSMUSG00000022702	Hira	20
ENSMUSG00000053835	H2-T24	20		ENSMUSG00000042606	Hirip3	19
ENSMUSG00000060032	H2afj	12		ENSMUSG00000049539	Hist1h1a	20
ENSMUSG00000041126	H2afv	1		ENSMUSG00000058773	Hist1h1b	20
ENSMUSG00000049932	H2afx	20		ENSMUSG00000036181	Hist1h1c	20
ENSMUSG00000015937	H2afy	20		ENSMUSG00000052565	Hist1h1d	20
ENSMUSG00000020086	H2afy2	20		ENSMUSG00000051627	Hist1h1e	20
ENSMUSG00000037894	H2afz	12		ENSMUSG00000061615	Hist1h2ab	20
ENSMUSG00000048994	H2al3	9		ENSMUSG00000069270	Hist1h2ac	20
ENSMUSG00000060743	H3f3a	12		ENSMUSG00000069272	Hist1h2ae	20
ENSMUSG00000073485	H3f3aos	20		ENSMUSG00000061991	Hist1h2af	1
ENSMUSG00000016559	H3f3b	20		ENSMUSG00000069301	Hist1h2ag	20
ENSMUSG00000082029	H3f3c	20		ENSMUSG00000071516	Hist1h2ai	20

ENSMUSG00000028980	H6pd	20		ENSMUSG00000063021	Hist1h2ak	20
ENSMUSG00000021476	Habp4	6		ENSMUSG00000091383	Hist1h2al	20
ENSMUSG00000063275	Hacd1	20		ENSMUSG00000094248	Hist1h2ao	20
ENSMUSG00000035376	Hacd2	1		ENSMUSG00000075031	Hist1h2bb	20
ENSMUSG00000033629	Hacd3	20		ENSMUSG00000018102	Hist1h2bc	19
ENSMUSG00000028497	Hacd4	9		ENSMUSG00000047246	Hist1h2be	20
ENSMUSG00000038822	Hace1	9		ENSMUSG00000058385	Hist1h2bg	20
ENSMUSG00000021884	Hacl1	3		ENSMUSG00000069300	Hist1h2bj	1
ENSMUSG00000027984	Hadh	12		ENSMUSG00000114279	Hist1h2bm	2
ENSMUSG00000025745	Hadha	9		ENSMUSG00000069265	Hist1h3a	20
ENSMUSG00000059447	Hadhb	20		ENSMUSG00000099583	Hist1h3d	20
ENSMUSG00000024158	Hagh	2		ENSMUSG00000069273	Hist1h3e	20
ENSMUSG00000061046	Haghl	2		ENSMUSG00000100210	Hist1h3f	20
ENSMUSG00000075277	Haglr	9		ENSMUSG00000099517	Hist1h3g	9
ENSMUSG00000006930	Hap1	20		ENSMUSG00000101355	Hist1h3h	20
ENSMUSG00000021613	Hapln1	20		ENSMUSG00000101972	Hist1h3i	1
ENSMUSG00000004894	Hapln2	16		ENSMUSG00000060093	Hist1h4a	20
ENSMUSG00000030606	Hapln3	20		ENSMUSG00000069266	Hist1h4b	20
ENSMUSG00000007594	Hapln4	18		ENSMUSG00000060678	Hist1h4c	19
ENSMUSG00000027243	Harbi1	20		ENSMUSG00000061482	Hist1h4d	20
ENSMUSG00000001380	Hars	9		ENSMUSG00000069274	Hist1h4f	20
ENSMUSG00000019143	Hars2	20		ENSMUSG00000060981	Hist1h4h	10
ENSMUSG00000003665	Has1	20		ENSMUSG00000060639	Hist1h4i	20
ENSMUSG00000031910	Has3	9		ENSMUSG00000067455	Hist1h4j	20
ENSMUSG00000027018	Hat1	20		ENSMUSG00000064288	Hist1h4k	20
ENSMUSG00000027285	Haus2	1		ENSMUSG00000069306	Hist1h4m	20
ENSMUSG00000079555	Haus3	20		ENSMUSG00000069305	Hist1h4n	20
ENSMUSG00000022177	Haus4	20		ENSMUSG00000063954	Hist2h2aa2	18
ENSMUSG00000078762	Haus5	12		ENSMUSG00000063689	Hist2h2ab	20
ENSMUSG00000038047	Haus6	20		ENSMUSG00000068855	Hist2h2ac	19
ENSMUSG00000031371	Haus7	20		ENSMUSG00000105827	Hist2h2bb	20
ENSMUSG00000035439	Haus8	20		ENSMUSG00000068854	Hist2h2be	20
ENSMUSG00000020399	Havcr2	20		ENSMUSG00000074403	Hist2h3b	18
ENSMUSG00000027944	Hax1	20		ENSMUSG00000081058	Hist2h3c2	20
ENSMUSG00000069919	Hba-a1	20		ENSMUSG00000091405	Hist2h4	9
ENSMUSG00000069917	Hba-a2	20		ENSMUSG00000078851	Hist3h2a	20
ENSMUSG00000052305	Hbb-bs	20		ENSMUSG00000056895	Hist3h2ba	20
ENSMUSG00000024486	Hbegf	9		ENSMUSG00000080712	Hist3h2bb-ps	20
ENSMUSG00000002996	Hbp1	20		ENSMUSG00000096010	Hist4h4	20
ENSMUSG00000019977	Hbs1l	2		ENSMUSG00000021366	Hivep1	18
ENSMUSG00000031352	Hccs	20		ENSMUSG00000015501	Hivep2	2
ENSMUSG00000031386	Hcfc1	20		ENSMUSG00000028634	Hivep3	3
ENSMUSG00000023904	Hcfc1r1	20		ENSMUSG00000044783	Hjurp	20
ENSMUSG00000020246	Hcfc2	20		ENSMUSG00000037012	Hk1	3
ENSMUSG00000006127	Inpp5k	20		ENSMUSG00000085347	Hk1os	20
ENSMUSG00000032737	Inppl1	16		ENSMUSG00000000628	Hk2	20
ENSMUSG00000048782	Insc	20		ENSMUSG00000025877	Hk3	20
ENSMUSG00000045294	Insig1	1		ENSMUSG00000020080	Hkdc1	20
ENSMUSG00000003721	Insig2	20		ENSMUSG00000040820	Hlcs	20
ENSMUSG00000066090	Insl5	12		ENSMUSG00000003949	Hlf	18
ENSMUSG00000050957	Insl6	12		ENSMUSG00000002428	Hltf	10
ENSMUSG00000068154	Insm1	20		ENSMUSG00000039377	Hlx	20
ENSMUSG00000005534	Insr	20		ENSMUSG00000098439	Hm629797	20
ENSMUSG00000005640	Insrr	20		ENSMUSG00000021972	Hmbox1	12

ENSMUSG00000066607	Insyn1	9		ENSMUSG00000032126	Hmbs	1
ENSMUSG00000073805	Insyn2a	20		ENSMUSG00000030060	Hmces	20
ENSMUSG00000069911	Insyn2b	9		ENSMUSG00000066842	Hmcn1	12
ENSMUSG00000029547	Ints1	12		ENSMUSG00000055632	Hmcn2	20
ENSMUSG00000031864	Ints10	20		ENSMUSG00000032329	Hmg20a	20
ENSMUSG00000029034	Ints11	20		ENSMUSG00000020232	Hmg20b	20
ENSMUSG00000028016	Ints12	20		ENSMUSG00000046711	Hmga1	18
ENSMUSG00000040250	Ints13	20		ENSMUSG00000078249	Hmga1b	11
ENSMUSG00000034263	Ints14	20		ENSMUSG00000066551	Hmgb1	20
ENSMUSG00000018068	Ints2	20		ENSMUSG00000084129	Hmgb1-ps1	5
ENSMUSG00000027933	Ints3	20		ENSMUSG00000081738	Hmgb1-ps2	20
ENSMUSG00000025133	Ints4	20		ENSMUSG00000083822	Hmgb1-ps5	20
ENSMUSG00000071652	Ints5	20		ENSMUSG00000080928	Hmgb1-ps6	20
ENSMUSG00000035161	Ints6	1		ENSMUSG00000092281	Hmgb1-ps7	9
ENSMUSG00000035967	Ints6l	3		ENSMUSG00000097295	Hmgb1-ps8	1
ENSMUSG00000037461	Ints7	9		ENSMUSG00000096722	Hmgb1-ps9	20
ENSMUSG00000040738	Ints8	1		ENSMUSG00000099773	Hmgb1-rs16	20
ENSMUSG00000021975	Ints9	13		ENSMUSG00000109750	Hmgb1-rs17	20
ENSMUSG00000028344	Invs	12		ENSMUSG00000054717	Hmgb2	20
ENSMUSG00000032594	Ip6k1	20		ENSMUSG00000015217	Hmgb3	20
ENSMUSG00000032599	Ip6k2	20		ENSMUSG00000085717	Hmgb4os	20
ENSMUSG00000024210	Ip6k3	20		ENSMUSG00000028672	Hmgcl	12
ENSMUSG00000064065	Ipcef1	1		ENSMUSG00000007908	Hmgcll1	20
ENSMUSG00000060733	Ipmk	10		ENSMUSG00000021670	Hmgcr	20
ENSMUSG00000042590	Ipo11	7		ENSMUSG00000093930	Hmgcs1	19
ENSMUSG00000033365	Ipo13	20		ENSMUSG00000027875	Hmgcs2	19
ENSMUSG00000002319	Ipo4	3		ENSMUSG00000040681	Hmgn1	9
ENSMUSG00000030662	Ipo5	12		ENSMUSG00000003038	Hmgn2	19
ENSMUSG00000066232	Ipo7	3		ENSMUSG00000079067	Hmgn2-ps1	20
ENSMUSG00000040029	Ipo8	2		ENSMUSG00000066456	Hmgn3	20
ENSMUSG00000041879	Ipo9	9		ENSMUSG00000031245	Hmgn5	20
ENSMUSG00000028696	Ipp	20		ENSMUSG00000024622	Hmgxb3	7
ENSMUSG00000021385	Ippk	9		ENSMUSG00000034518	Hmgxb4	12
ENSMUSG00000102018	Iqank1	20		ENSMUSG00000020330	Hmmr	13
ENSMUSG00000026301	Iqca	14		ENSMUSG00000005413	Hmox1	20
ENSMUSG00000022837	Iqcb1	20		ENSMUSG00000004070	Hmox2	3
ENSMUSG00000040795	Iqcc	20		ENSMUSG00000020679	Hnf1b	20
ENSMUSG00000029601	Iqcd	14		ENSMUSG00000026986	Hnmt	20
ENSMUSG00000036555	Iqce	20		ENSMUSG00000007836	Hnrnpa0	20
ENSMUSG00000035578	Iqcg	14		ENSMUSG00000046434	Hnrnpa1	9
ENSMUSG00000051777	Iqcj	20		ENSMUSG00000004980	Hnrnpa2b1	20
ENSMUSG00000073856	Iqck	20		ENSMUSG00000059005	Hnrnpa3	20
ENSMUSG00000031620	Iqcm	12		ENSMUSG00000020358	Hnrnpab	20
ENSMUSG00000030536	Iqgap1	12		ENSMUSG00000060373	Hnrnpc	18
ENSMUSG00000021676	Iqgap2	9		ENSMUSG00000000568	Hnrnpd	20
ENSMUSG00000028068	Iqgap3	20		ENSMUSG00000029328	Hnrnpdl	20
ENSMUSG00000034312	Iqsec1	16		ENSMUSG00000042079	Hnrnpf	20
ENSMUSG00000041115	Iqsec2	9		ENSMUSG00000007850	Hnrnph1	18
ENSMUSG00000040797	Iqsec3	12		ENSMUSG00000045427	Hnrnph2	1
ENSMUSG00000046192	Iqub	1		ENSMUSG00000020069	Hnrnph3	12
ENSMUSG00000031392	Irak1	20		ENSMUSG00000021546	Hnrnpk	20
ENSMUSG00000032251	Irak1bp1	18		ENSMUSG00000015165	Hnrnpl	20
ENSMUSG00000060477	Irak2	20		ENSMUSG00000024095	Hnrnpll	7
ENSMUSG00000020227	Irak3	20		ENSMUSG00000059208	Hnrnpm	18

ENSMUSG00000059883	Irak4	20		ENSMUSG00000066037	Hnrnpr	9
ENSMUSG00000032293	Ireb2	20		ENSMUSG00000039630	Hnrnpu	20
ENSMUSG00000018899	Irf1	20		ENSMUSG00000040725	Hnrnpul1	20
ENSMUSG00000031627	Irf2	20		ENSMUSG00000071659	Hnrnpul2	20
ENSMUSG00000044030	Irf2bp1	20		ENSMUSG00000025176	Hoga1	20
ENSMUSG00000051495	Irf2bp2	20		ENSMUSG00000007617	Homer1	9
ENSMUSG00000034168	Irf2bpl	12		ENSMUSG00000025813	Homer2	19
ENSMUSG00000003184	Irf3	20		ENSMUSG00000003573	Homer3	5
ENSMUSG00000029771	Irf5	20		ENSMUSG00000057156	Homez	20
ENSMUSG00000026638	Irf6	20		ENSMUSG00000028572	Hook1	15
ENSMUSG00000025498	Irf7	9		ENSMUSG00000052566	Hook2	9
ENSMUSG00000041515	Irf8	20		ENSMUSG00000037234	Hook3	19
ENSMUSG00000002325	Irf9	20		ENSMUSG00000059325	Hopx	3
ENSMUSG00000046879	Irgm1	20		ENSMUSG00000087658	Hotairm1	1
ENSMUSG00000069874	Irgm2	20		ENSMUSG00000022484	Hoxc10	1
ENSMUSG00000041037	Irgq	3		ENSMUSG00000075394	Hoxc4	1
ENSMUSG00000055980	Irs1	9		ENSMUSG00000022485	Hoxc5	1
ENSMUSG00000038894	Irs2	18		ENSMUSG00000001661	Hoxc6	1
ENSMUSG00000054667	Irs4	17		ENSMUSG00000001657	Hoxc8	1
ENSMUSG00000060969	Irx1	6		ENSMUSG00000036139	Hoxc9	1
ENSMUSG00000001504	Irx2	18		ENSMUSG00000027102	Hoxd8	1
ENSMUSG00000031734	Irx3	18		ENSMUSG00000028759	Hp1bp3	18
ENSMUSG00000031737	Irx5	3		ENSMUSG00000028785	Hpca	9
ENSMUSG00000044792	Isca1	1		ENSMUSG00000071379	Hpcal1	2
ENSMUSG00000021241	Isca2	2		ENSMUSG00000046093	Hpcal4	18
ENSMUSG00000025825	Iscu	12		ENSMUSG00000043155	Hpdl	20
ENSMUSG00000035692	Isg15	20		ENSMUSG00000038005	Hpf1	20
ENSMUSG00000039236	Isg20	20		ENSMUSG00000031613	Hpgd	20
ENSMUSG00000048039	Isg20l2	20		ENSMUSG00000029919	Hpgds	20
ENSMUSG00000042258	Isl1	20		ENSMUSG00000001249	Hpn	1
ENSMUSG00000037206	Islr	13		ENSMUSG00000025630	Hprt	20
ENSMUSG00000051243	Islr2	18		ENSMUSG00000025188	Hps1	20
ENSMUSG00000074766	Ism1	20		ENSMUSG00000027615	Hps3	20
ENSMUSG00000024601	Isoc1	20		ENSMUSG00000042328	Hps4	20
ENSMUSG00000086784	Isoc2a	20		ENSMUSG00000014418	Hps5	6
ENSMUSG00000052605	Isoc2b	20		ENSMUSG00000074811	Hps6	9
ENSMUSG00000031729	Ist1	20		ENSMUSG00000022096	Hr	18
ENSMUSG00000030056	Isy1	20		ENSMUSG00000025499	Hras	9
ENSMUSG00000019139	Isyna1	20		ENSMUSG00000071001	Hrct1	20
ENSMUSG00000027598	Itch	2		ENSMUSG00000053004	Hrh1	9
ENSMUSG00000031703	Itfg1	9		ENSMUSG00000034987	Hrh2	9
ENSMUSG00000001518	Itfg2	9		ENSMUSG00000039059	Hrh3	1
ENSMUSG00000042284	Itga1	20		ENSMUSG00000046607	Hrk	9
ENSMUSG00000090210	Itga10	1		ENSMUSG00000020605	Hs1bp3	20
ENSMUSG00000032243	Itga11	20		ENSMUSG00000040151	Hs2st1	7
ENSMUSG00000015533	Itga2	20		ENSMUSG00000051022	Hs3st1	9
ENSMUSG00000034664	Itga2b	20		ENSMUSG00000046321	Hs3st2	9
ENSMUSG00000001507	Itga3	18		ENSMUSG00000047759	Hs3st3a1	20
ENSMUSG00000027009	Itga4	19		ENSMUSG00000070407	Hs3st3b1	20
ENSMUSG00000000555	Itga5	9		ENSMUSG00000078591	Hs3st4	20
ENSMUSG00000027111	Itga6	20		ENSMUSG00000044499	Hs3st5	9
ENSMUSG00000025348	Itga7	5		ENSMUSG00000045216	Hs6st1	3
ENSMUSG00000026768	Itga8	5		ENSMUSG00000062184	Hs6st2	20
ENSMUSG00000039115	Itga9	1		ENSMUSG00000053465	Hs6st3	20

ENSMUSG00000070369	Itgad	20		ENSMUSG00000031839	Hsbp1	12
ENSMUSG00000005947	Itgae	20		ENSMUSG00000078963	Hsbp1l1	20
ENSMUSG00000030830	Itgal	20		ENSMUSG00000043510	Hscb	20
ENSMUSG00000030786	Itgam	10		ENSMUSG00000016194	Hsd11b1	19
ENSMUSG00000027087	Itgav	2		ENSMUSG00000025260	Hsd17b10	3
ENSMUSG00000030789	Itgax	20		ENSMUSG00000029311	Hsd17b11	20
ENSMUSG00000025809	Itgb1	20		ENSMUSG00000027195	Hsd17b12	9
ENSMUSG00000062352	Itgb1bp1	19		ENSMUSG00000024507	Hsd17b4	20
ENSMUSG00000031312	Itgb1bp2	20		ENSMUSG00000026675	Hsd17b7	1
ENSMUSG00000000290	Itgb2	20		ENSMUSG00000042289	Hsd3b7	20
ENSMUSG00000020689	Itgb3	20		ENSMUSG00000034189	Hsdl1	3
ENSMUSG00000020758	Itgb4	16		ENSMUSG00000028383	Hsdl2	20
ENSMUSG00000022817	Itgb5	20		ENSMUSG00000022556	Hsf1	5
ENSMUSG00000001281	Itgb7	20		ENSMUSG00000019878	Hsf2	2
ENSMUSG00000025321	Itgb8	20		ENSMUSG00000033249	Hsf4	18
ENSMUSG00000032925	Itgbl1	1		ENSMUSG00000070345	Hsf5	20
ENSMUSG00000037254	Itih2	20		ENSMUSG00000078915	Hsp25-ps1	19
ENSMUSG00000006522	Itih3	20		ENSMUSG00000021270	Hsp90aa1	9
ENSMUSG00000025780	Itih5	18		ENSMUSG00000023944	Hsp90ab1	20
ENSMUSG00000020395	Itk	12		ENSMUSG00000020048	Hsp90b1	1
ENSMUSG00000031239	Itm2a	20		ENSMUSG00000025092	Hspa12a	19
ENSMUSG00000022108	Itm2b	12		ENSMUSG00000074793	Hspa12b	20
ENSMUSG00000026223	Itm2c	3		ENSMUSG00000032932	Hspa13	1
ENSMUSG00000074797	Itpa	1		ENSMUSG00000109865	Hspa14	20
ENSMUSG00000081476	Itpa-ps1	13		ENSMUSG00000091971	Hspa1a	20
ENSMUSG00000057963	Itpk1	20		ENSMUSG00000090877	Hspa1b	11
ENSMUSG00000027296	Itpka	3		ENSMUSG00000059970	Hspa2	4
ENSMUSG00000038855	Itpkb	18		ENSMUSG00000020361	Hspa4	20
ENSMUSG00000003752	Itpkc	20		ENSMUSG00000025757	Hspa4l	20
ENSMUSG00000030102	Itpr1	9		ENSMUSG00000026864	Hspa5	20
ENSMUSG00000030287	Itpr2	3		ENSMUSG00000015656	Hspa8	1
ENSMUSG00000042644	Itpr3	18		ENSMUSG00000024359	Hspa9	20
ENSMUSG00000027007	Itprid2	20		ENSMUSG00000004951	Hspb1	20
ENSMUSG00000117975	Itprip	20		ENSMUSG00000063172	Hspb11	12
ENSMUSG00000074825	Itpripl1	20		ENSMUSG00000036854	Hspb6	20
ENSMUSG00000095115	Itpripl2	20		ENSMUSG00000041548	Hspb8	3
ENSMUSG00000022957	Itsn1	18		ENSMUSG00000022849	Hspbap1	20
ENSMUSG00000020640	Itsn2	2		ENSMUSG00000063802	Hspbp1	3
ENSMUSG00000027332	Ivd	2		ENSMUSG00000025980	Hspd1	20
ENSMUSG00000023150	Ivns1abp	9		ENSMUSG00000058809	Hspd1-ps3	18
ENSMUSG00000024384	Iws1	20		ENSMUSG00000073676	Hspe1	12
ENSMUSG00000019762	Iyd	18		ENSMUSG00000117621	Hspe1-rs1	20
ENSMUSG00000055862	Izumo4	20		ENSMUSG00000028763	Hspg2	13
ENSMUSG00000025764	Jade1	20		ENSMUSG00000029657	Hsph1	20
ENSMUSG00000020387	Jade2	1		ENSMUSG00000039745	Htatip2	18
ENSMUSG00000037315	Jade3	12		ENSMUSG00000067873	Htatsf1	9
ENSMUSG00000027276	Jag1	1		ENSMUSG00000021721	Htr1a	5
ENSMUSG00000002799	Jag2	20		ENSMUSG00000049511	Htr1b	9
ENSMUSG00000051256	Jagn1	20		ENSMUSG00000070687	Htr1d	9
ENSMUSG00000028530	Jak1	20		ENSMUSG00000050783	Htr1f	1
ENSMUSG00000024789	Jak2	9		ENSMUSG00000034997	Htr2a	9
ENSMUSG00000031805	Jak3	20		ENSMUSG00000041380	Htr2c	20
ENSMUSG00000063646	Jakmip1	9		ENSMUSG00000032269	Htr3a	20
ENSMUSG00000024502	Jakmip2	20		ENSMUSG00000026322	Htr4	9

ENSMUSG00000056856	Jakmip3	20		ENSMUSG00000039106	Htr5a	20
ENSMUSG00000053062	Jam2	18		ENSMUSG00000050534	Htr5b	20
ENSMUSG00000031990	Jam3	9		ENSMUSG00000028747	Htr6	9
ENSMUSG00000038518	Jarid2	20		ENSMUSG00000024798	Htr7	17
ENSMUSG00000063568	Jazf1	20		ENSMUSG00000006205	Htra1	1
ENSMUSG00000033960	Jcad	9		ENSMUSG00000068329	Htra2	12
ENSMUSG00000067149	Jchain	19		ENSMUSG00000029096	Htra3	20
ENSMUSG00000034271	Jdp2	9		ENSMUSG00000029104	Htt	8
ENSMUSG00000032023	Jhy	9		ENSMUSG00000053414	Hunk	5
ENSMUSG00000005078	Jkamp	20		ENSMUSG00000020413	Hus1	20
ENSMUSG00000037876	Jmjd1c	2		ENSMUSG00000076430	Hus1b	20
ENSMUSG00000036819	Jmjd4	16		ENSMUSG00000025261	Huwe1	3
ENSMUSG00000056962	Jmjd6	20		ENSMUSG00000064267	Hvcn1	9
ENSMUSG00000098789	Jmjd7	9		ENSMUSG00000010051	Hyal1	20
ENSMUSG00000025736	Jmjd8	20		ENSMUSG00000010047	Hyal2	20
ENSMUSG00000021690	Jmy	2		ENSMUSG00000059854	Hydin	14
ENSMUSG00000022426	Josd1	20		ENSMUSG00000006395	Hyi	20
ENSMUSG00000038695	Josd2	2		ENSMUSG00000035878	Hykk	20
ENSMUSG00000042686	Jph1	9		ENSMUSG00000050555	Hyls1	20
ENSMUSG00000017817	Jph2	20		ENSMUSG00000032115	Hyou1	12
ENSMUSG00000074896	Ifit3	20		ENSMUSG00000027245	Hypk	20
ENSMUSG00000062488	Ifit3b	20		ENSMUSG00000040456	Hypm	9
ENSMUSG00000045777	Ifitm10	20		ENSMUSG00000116504	I730030J21Ri	20
ENSMUSG00000060591	Ifitm2	20		ENSMUSG00000074342	I830077J02Ri	20
ENSMUSG00000025492	Ifitm3	13		ENSMUSG00000062054	Iah1	20
ENSMUSG00000065968	Ifitm7	20		ENSMUSG00000037851	Iars	18
ENSMUSG00000022967	Ifnar1	1		ENSMUSG00000026618	Iars2	9
ENSMUSG00000022971	Ifnar2	20		ENSMUSG00000049287	Iba57	20
ENSMUSG00000020009	Ifngr1	20		ENSMUSG00000035941	Ibtk	20
ENSMUSG00000022965	Ifngr2	1		ENSMUSG00000062995	Ica1	20
ENSMUSG00000062157	Ifnlr1	20		ENSMUSG00000026018	Ica1l	20
ENSMUSG00000001627	Ifrd1	20		ENSMUSG00000037405	Icam1	20
ENSMUSG00000010048	Ifrd2	20		ENSMUSG00000001029	Icam2	20
ENSMUSG00000030323	Ift122	20		ENSMUSG00000032174	Icam5	18
ENSMUSG00000024169	Ift140	20		ENSMUSG00000034525	Ice1	1
ENSMUSG00000038564	Ift172	20		ENSMUSG00000032235	Ice2	20
ENSMUSG00000001105	Ift20	20		ENSMUSG00000009828	Ick	18
ENSMUSG00000007987	Ift22	20		ENSMUSG00000039662	Icmt	20
ENSMUSG00000016637	Ift27	2		ENSMUSG00000000732	Icosl	20
ENSMUSG00000007867	Ift43	20		ENSMUSG00000042745	Id1	20
ENSMUSG00000002031	Ift46	20		ENSMUSG00000020644	Id2	3
ENSMUSG00000017858	Ift52	20		ENSMUSG00000007872	Id3	20
ENSMUSG00000032965	Ift57	20		ENSMUSG00000021379	Id4	1
ENSMUSG00000028576	Ift74	20		ENSMUSG00000025950	Idh1	20
ENSMUSG00000027778	Ift80	20		ENSMUSG00000030541	Idh2	3
ENSMUSG00000029469	Ift81	1		ENSMUSG00000032279	Idh3a	9
ENSMUSG00000040040	Ift88	20		ENSMUSG00000027406	Idh3b	2
ENSMUSG00000086486	Ift88os	20		ENSMUSG00000002010	Idh3g	20
ENSMUSG00000031221	Igbp1	20		ENSMUSG00000058258	Idi1	19
ENSMUSG00000032394	Igdcc3	20		ENSMUSG00000050002	Idnk	9
ENSMUSG00000032816	Igdcc4	18		ENSMUSG00000031551	Ido1	3
ENSMUSG00000020053	Igf1	18		ENSMUSG00000031549	Ido2	20
ENSMUSG00000005533	Igf1r	20		ENSMUSG00000035847	Ids	1
ENSMUSG00000048583	Igf2	13		ENSMUSG00000033540	Idua	1

ENSMUSG00000033581	Igf2bp2	20		ENSMUSG00000053560	Ier2	20
ENSMUSG00000029814	Igf2bp3	20		ENSMUSG00000003541	Ier3	20
ENSMUSG00000023830	Igf2r	12		ENSMUSG00000090000	Ier3ip1	12
ENSMUSG00000039323	Igfbp2	13		ENSMUSG00000056708	Ier5	9
ENSMUSG00000020427	Igfbp3	20		ENSMUSG00000089762	Ier5l	1
ENSMUSG00000017493	Igfbp4	9		ENSMUSG00000038271	Iffo1	20
ENSMUSG00000026185	Igfbp5	6		ENSMUSG00000041025	Iffo2	20
ENSMUSG00000023046	Igfbp6	19		ENSMUSG00000039997	Ifi203	20
ENSMUSG00000036256	Igfbp7	13		ENSMUSG00000073489	Ifi204	20
ENSMUSG00000035551	Igfbpl1	20		ENSMUSG00000043263	Ifi209	20
ENSMUSG00000051985	Igfn1	13		ENSMUSG00000064215	Ifi27	13
ENSMUSG00000076617	Ighm	9		ENSMUSG00000079017	Ifi27l2a	12
ENSMUSG00000024831	Ighmbp2	20		ENSMUSG00000021208	Ifi27l2b	1
ENSMUSG00000110185	Igip	20		ENSMUSG00000010358	Ifi35	20
ENSMUSG00000013367	Iglon5	18		ENSMUSG00000028037	Ifi44	20
ENSMUSG00000031111	Igsf1	6		ENSMUSG00000026896	Ifih1	20
ENSMUSG00000036334	Igsf10	17		ENSMUSG00000034459	Ifit1	20
ENSMUSG00000022790	Igsf11	9		ENSMUSG00000079339	Ifit1bl1	20
ENSMUSG00000040972	Igsf21	20		ENSMUSG00000067297	Ifit1bl2	20
ENSMUSG00000042035	Igsf3	20		ENSMUSG00000045932	Ifit2	20
ENSMUSG00000000159	Igsf5	20		ENSMUSG00000037679	Inf2	9
ENSMUSG00000035004	Igsf6	20		ENSMUSG00000045969	Ing1	20
ENSMUSG00000038034	Igsf8	2		ENSMUSG00000063049	Ing2	20
ENSMUSG00000037995	Igsf9	1		ENSMUSG00000029670	Ing3	20
ENSMUSG00000034275	Igsf9b	18		ENSMUSG00000030330	Ing4	20
ENSMUSG00000078853	Igtp	20		ENSMUSG00000026283	Ing5	20
ENSMUSG00000054072	Iigp1	20		ENSMUSG00000032968	Inha	3
ENSMUSG00000024474	Ik	20		ENSMUSG00000041324	Inhba	9
ENSMUSG00000019975	Ikbip	20		ENSMUSG00000037035	Inhbb	20
ENSMUSG00000031537	Ikbkb	20		ENSMUSG00000038544	Inip	20
ENSMUSG00000042349	Ikbke	20		ENSMUSG00000042106	Inka1	20
ENSMUSG00000004221	Ikbkg	20		ENSMUSG00000048458	Inka2	9
ENSMUSG00000018654	Ikzf1	20		ENSMUSG00000003477	Inmt	20
ENSMUSG00000025997	Ikzf2	20		ENSMUSG00000034154	Ino80	20
ENSMUSG00000018168	Ikzf3	19		ENSMUSG00000030034	Ino80b	3
ENSMUSG00000002578	Ikzf4	20		ENSMUSG00000047989	Ino80c	12
ENSMUSG00000040167	Ikzf5	20		ENSMUSG00000040865	Ino80d	2
ENSMUSG00000032089	Il10ra	20		ENSMUSG00000084799	Ino80dos	20
ENSMUSG00000022969	Il10rb	20		ENSMUSG00000030689	Ino80e	20
ENSMUSG00000073889	Il11ra1	1		ENSMUSG00000026102	Inpp1	19
ENSMUSG00000018341	Il12rb2	20		ENSMUSG00000026113	Inpp4a	2
ENSMUSG00000017057	Il13ra1	1		ENSMUSG00000037940	Inpp4b	18
ENSMUSG00000031712	Il15	20		ENSMUSG00000025477	Inpp5a	20
ENSMUSG00000023206	Il15ra	20		ENSMUSG00000028894	Inpp5b	9
ENSMUSG00000001741	Il16	5		ENSMUSG00000026288	Inpp5d	20
ENSMUSG00000024578	Il17b	20		ENSMUSG00000026925	Inpp5e	20
ENSMUSG00000050222	Il17d	20		ENSMUSG00000042105	Inpp5f	9
ENSMUSG00000002897	Il17ra	20		ENSMUSG00000032288	Imp3	19
ENSMUSG00000015966	Il17rb	3		ENSMUSG00000026127	Imp4	20
ENSMUSG00000030281	Il17rc	1		ENSMUSG00000027531	Impa1	20
ENSMUSG00000040717	Il17rd	20		ENSMUSG00000024525	Impa2	20
ENSMUSG00000039217	Il18	20		ENSMUSG00000024423	Impact	17
ENSMUSG00000070427	Il18bp	20		ENSMUSG00000066324	Impad1	3
ENSMUSG00000027399	Il1a	20		ENSMUSG00000003500	Impdh1	20

ENSMUSG00000026072	Il1r1	20		ENSMUSG00000062867	Impdh2	20
ENSMUSG00000022514	Il1rap	19		ENSMUSG00000071041	Impdh2-ps	20
ENSMUSG00000052372	Il1rapl1	3		ENSMUSG00000032343	Impg1	9
ENSMUSG00000059203	Il1rapl2	9		ENSMUSG00000035270	Impg2	20
ENSMUSG00000026069	Il1rl1	20		ENSMUSG00000034336	Ina	18
ENSMUSG00000070942	Il1rl2	19		ENSMUSG00000091811	Inafm1	20
ENSMUSG00000020007	Il20ra	9		ENSMUSG00000074918	Inafm2	20
ENSMUSG00000044244	Il20rb	20		ENSMUSG00000001016	Ilf2	20
ENSMUSG00000030745	Il21r	2		ENSMUSG00000032178	Ilf3	20
ENSMUSG00000005465	Il27ra	20		ENSMUSG00000030890	Ilk	3
ENSMUSG00000068227	Il2rb	1		ENSMUSG00000026309	Ilkap	20
ENSMUSG00000031304	Il2rg	20		ENSMUSG00000056692	Ilrun	8
ENSMUSG00000024810	Il33	16		ENSMUSG00000032763	Ilvbl	20
ENSMUSG00000031750	Il34	9		ENSMUSG00000042670	Immp1l	20
ENSMUSG00000068758	Il3ra	20		ENSMUSG00000056899	Immp2l	20
ENSMUSG00000074141	Il4i1	20		ENSMUSG00000052337	Immt	9
ENSMUSG00000030748	Il4ra	9		ENSMUSG00000041605	Inava	20
ENSMUSG00000025746	Il6	20		ENSMUSG00000057054	Inca1	20
ENSMUSG00000027947	Il6ra	20		ENSMUSG00000024660	Incenp	20
ENSMUSG00000021756	Il6st	12		ENSMUSG00000034570	Inpp5j	16
ENSMUSG00000003882	Il7r	20		ENSMUSG00000025318	Jph3	20
ENSMUSG00000040612	Ildr2	19				
