# Supplementary material for: CK2 alpha prime and alpha-synuclein pathogenic functional interaction mediates synaptic dysregulation in huntington’s disease
Source: Acta Neuropathol Commun. 2022 Jun 3;10:83. doi: 10.1186/s40478-022-01379-8 (PMC9164558; doi:10.1186/s40478-022-01379-8)
Supplement: Supplementary file 7 — Additional file 7. WGCNA module names and number of genes per module. [file 40478_2022_1379_MOESM7_ESM.pdf]

Table S2. WGCNA module names and number of genes per module

ModuleName	ModuleID	NoGene	Pval(HD-HD;CK2a'+/-)	Pval(HD-WT)
Blue	1	1165	0.9168	0.0472
Black	2	432	0.3472	0.0163
Green	3	711	0.4647	0.0090
Cyan	4	107	0.6015	0.1172
Greenyellow	5	255	0.0090	0.0090
Salmon	6	211	0.1745	0.4647
Tan	7	235	0.7540	0.1745
Grey60	8	66	0.7540	0.9168
Turquoise	9	2016	0.9168	0.7540
Pink	10	404	0.6015	0.3472
Lightyellow	11	52	0.9168	0.0472
Yellow	12	994	0.3472	0.0472
Magenta	13	324	0.9168	0.7540
Midnightblue	14	93	0.7540	0.9168
Lightgreen	15	53	0.1172	0.6015
Purple	16	268	0.2506	0.0472
Lightcyan	17	82	0.1745	0.9168
Brown	18	1067	0.9168	0.6015
Red	19	639	0.0472	0.0163
Grey	20	11926	0.0758	0.0163
