# Supplementary material for: CK2 alpha prime and alpha-synuclein pathogenic functional interaction mediates synaptic dysregulation in huntington’s disease
Source: Acta Neuropathol Commun. 2022 Jun 3;10:83. doi: 10.1186/s40478-022-01379-8 (PMC9164558; doi:10.1186/s40478-022-01379-8)
Supplement: Supplementary file 11 — Additional file 11. Expression analyses for astrocyte markers of A1, A2 and pan-reactive (from Liddelow et al., 2017 (40)) in WT, zQ175, CK2α’(+/-) and zQ175:CK2α’(+/-). [file 40478_2022_1379_MOESM11_ESM.pdf]

Table S4. Expression analyses for astrocyte markers of A1, A2 and pan reactive (from Liddelow et al., 2017)

GENES ASSOCIATED WITH PAN REACTIVE, A1 AND A2 ASTROCYTES

EnsemblID	FC_HD.WT	P_HD.WT	Q_HD.WT	FC_HDHET	P_HDHET.\	Q_HDHET.	FC_HD.HD	P_HD.HDH	Q_HD.HDH	FC_HD.HE	P_HD.HET	Q_HD.HET	FC_HDHET	P_HDHET.I	Q_HDHET.	FC_HET.W	P_HET.WT	Q_HET.WT	GeneSymbol	
ENSMUSG	-1.14609	0.160577	NA	-0.51943	0.515094	NA	-0.62666	0.451644	0.996027	0.276807	0.780398	NA	0.903464	0.355314	NA	-1.42289	0.139949	1	Lcn2	PAN REACTIVE
ENSMUSG	1.790505	0.232362	NA	2.560314	0.085282	NA	-0.76981	0.587928	1	-0.60378	0.712229	NA	0.166024	0.918696	NA	2.394289	0.157896	1	Steap4	
ENSMUSG	-0.12823	0.649538	0.867518	-0.24247	0.393998	0.869917	0.114237	0.690479	1	-0.03872	0.905648	0.978208	-0.15296	0.641602	0.939904	-0.08952	0.782683	1	S1pr3	
ENSMUSG	-1.08231	0.473498	NA	-0.35739	0.807911	NA	-0.72493	0.636129	1	-2.99215	0.073848	NA	-2.26723	0.1663	NA	1.909843	0.237589	1	Timp1	
ENSMUSG	-0.15143	0.14569	0.491222	-0.1656	0.111857	0.631925	0.014173	0.891967	1	-0.12115	0.31343	0.712446	-0.13532	0.260442	0.774228	-0.03029	0.800766	1	Hsbp1	
ENSMUSG	-1.41815	0.256904	NA	-0.72026	0.550822	NA	-0.69789	0.587972	1	-0.2923	0.844921	NA	0.405597	0.780891	NA	-1.12586	0.429587	1	Cxcl10	
ENSMUSG	-0.04361	0.898373	0.967005	0.130886	0.701001	0.961868	-0.17449	0.609707	1	-0.20838	0.595865	0.881176	-0.03388	0.931193	0.988925	0.164768	0.67431	1	Cd44	
ENSMUSG	0.108797	0.72898	0.901614	0.58812	0.059632	0.523315	-0.47932	0.124813	0.965883	0.311288	0.392855	0.77016	0.790611	0.029318	0.368578	-0.20249	0.578302	1	Osmr	
ENSMUSG	0.099363	0.508843	0.797025	0.048157	0.749735	0.970313	0.051206	0.734593	1	-0.2197	0.201718	0.606144	-0.27091	0.116477	0.627492	0.319065	0.06363	1	Cp	
ENSMUSG	-4.40929	0.030442	NA	-4.32644	0.033701	NA	0	1	1	-2.6608	0.264432	NA	-2.57795	0.279595	NA	-1.74849	0.431987	1	Serpina3g	
ENSMUSG	3.967647	0.077223	NA	3.433971	0.129329	NA	0.533676	0.800316	1	1.226066	0.620646	NA	0.692389	0.781315	NA	2.741582	0.293479	1	Serpina3h	
ENSMUSG	-0.79751	0.759147	NA	-0.36524	0.887488	NA	-0.43228	0.86915	1	2.37176	0.451349	NA	2.804037	0.370761	NA	-3.16927	0.308748	1	Serpina3i	
ENSMUSG	2.512734	0.340487	NA	3.575762	0.168249	NA	-1.06303	0.672804	1	0.82375	0.785652	NA	1.886778	0.528486	NA	1.688984	0.585142	1	Serpina3m	
ENSMUSG	0.933112	0.067569	0.348361	0.796007	0.119073	0.643816	0.137105	0.787956	1	-0.31337	0.594029	0.880253	-0.45048	0.44369	0.878418	1.246486	0.034246	1	Serpina3n	
ENSMUSG	-0.0678	0.624355	0.857131	0.139295	0.313399	0.832195	-0.2071	0.135094	0.967737	0.205383	0.201412	0.605698	0.412482	0.010177	0.231486	-0.27319	0.088546	1	Vim	
ENSMUSG	-0.4955	0.07811	0.371649	0.055645	0.843119	0.981536	-0.55115	0.050059	0.885412	-0.50628	0.118986	0.490899	0.044869	0.890081	0.981797	0.010776	0.973521	1	Gfap	
ENSMUSG	-0.72189	0.368618	0.70618	-0.902	0.262302	0.798596	0.180102	0.823713	1	-1.07199	0.246299	0.655329	-1.25209	0.17638	0.701112	0.350098	0.703942	1	H2-T23	1 ASTROCYTES
ENSMUSG	0.12642	0.48241	0.782747	0.455609	0.011137	0.299313	-0.32919	0.066615	0.935832	-0.06982	0.736268	0.930761	0.259366	0.209981	0.737619	0.196244	0.34389	1	H2-D1	
ENSMUSG	-0.20162	0.467756	0.774615	-0.18011	0.517461	0.912907	-0.02151	0.939009	1	-0.14373	0.654056	0.903021	-0.12222	0.703608	0.95044	-0.05789	0.855663	1	Ggta1	
ENSMUSG	0.069998	0.839507	0.946987	0.560035	0.101912	0.610901	-0.49004	0.15288	0.97929	-0.40636	0.302386	0.703934	0.083673	0.830628	0.975732	0.476361	0.226233	1	Serping1	
ENSMUSG	0.266222	0.421759	0.746781	0.123029	0.712521	0.964926	0.143193	0.666518	1	-0.07797	0.836705	0.958365	-0.22116	0.561076	0.915483	0.344194	0.364691	1	Gbp2	
ENSMUSG	-0.62343	0.045665	0.289343	-0.20975	0.497504	0.908343	-0.41368	0.188491	0.979658	-0.98433	0.005721	0.109284	-0.57065	0.10674	0.613553	0.360896	0.304481	1	Fbln5	
ENSMUSG	0.043275	0.771781	0.919025	0.111505	0.454911	0.896838	-0.06823	0.647742	1	-0.07519	0.66209	0.906065	-0.00696	0.967742	0.995398	0.118463	0.490854	1	Fkbp5	
ENSMUSG	-0.84054	0.030038	0.245223	0.114671	0.762051	0.972927	-0.95521	0.013821	0.648683	-1.0298	0.020132	0.214269	-0.07459	0.864031	0.978111	0.189256	0.663533	1	Psmb8	
ENSMUSG	-0.30531	0.160581	0.51444	-0.10883	0.614997	0.939091	-0.19649	0.372701	0.996027	-0.29768	0.234559	0.642354	-0.1012	0.684883	0.945541	-0.00763	0.975339	1	Srgn	
ENSMUSG	0.115763	0.697483	0.888776	-0.42375	0.158708	0.693481	0.53951	0.07274	0.938721	0.39397	0.254363	0.663391	-0.14554	0.675875	0.943916	-0.27821	0.420878	1	Amigo2	
ENSMUSG	-0.17675	0.694637	0.888085	-0.69539	0.126893	0.655866	0.518645	0.256878	0.990064	-0.94119	0.067278	0.388265	-1.45984	0.004914	0.158852	0.764449	0.135948	1	C3	
ENSMUSG	-0.26417	0.773744	NA	0.71946	0.414296	NA	-0.98363	0.274642	0.99181	-1.76392	0.077039	NA	-0.78029	0.417828	NA	1.499753	0.126091	1	Tgm1	2 ASTROCYTES
ENSMUSG	-1.58113	0.455925	NA	-1.21898	0.560591	NA	-0.36214	0.868959	1	-1.08744	0.656814	NA	-0.7253	0.764859	NA	-0.49368	0.834147	1	Ptx3	
**ENSMUSG**	**-1.04664**	**2.68E-05**	**0.005563**	**-1.08878**	**1.34E-05**	**0.017165**	**0.042138**	**0.86894**	**1**	**-1.39026**	**1.08E-06**	**0.000357**	**-1.4324**	**5.39E-07**	**0.000446**	**0.343614**	**0.220356**	**1**	**S100a10**	
ENSMUSG	-0.09771	0.90033	NA	0.512506	0.505334	0.910563	-0.61021	0.430625	0.996027	-0.24498	0.784491	NA	0.365235	0.68033	NA	0.14727	0.868806	1	Sphk1	
ENSMUSG	0.314958	0.502937	0.7939	-0.12158	0.798852	0.978381	0.436535	0.357835	0.995411	-0.07592	0.887681	0.973916	-0.51246	0.345832	0.831106	0.39088	0.468833	1	Cd109	
**ENSMUSG**	**1.260279**	**0.002191**	**0.06471**	**0.362463**	**0.386982**	**0.866418**	**0.897816**	**0.028387**	**0.787307**	**1.362371**	**0.004538**	**0.094917**	**0.464556**	**0.339624**	**0.826888**	**-0.10209**	**0.834304**	**1**	**Ptgs2**	
ENSMUSG	0.353648	0.406305	0.73449	0.11733	0.785357	0.976151	0.236318	0.580209	1	0.638384	0.202357	0.607107	0.402066	0.425894	0.871336	-0.28474	0.571929	1	Emp1	
ENSMUSG	-0.11974	0.621289	0.855328	-0.14423	0.553079	0.925041	0.02449	0.920283	1	-0.65889	0.01682	0.196558	-0.68338	0.013387	0.265386	0.53915	0.049306	1	Tm4sf1	
ENSMUSG	-0.27627	0.747238	NA	0.124371	0.883473	NA	-0.40064	0.640922	1	1.136303	0.285882	NA	1.536942	0.146228	NA	-1.41257	0.181152	1	B3gnt5	
ENSMUSG	0.240867	0.594653	0.843206	0.749193	0.093403	0.592613	-0.50833	0.252724	0.988916	-0.52061	0.305617	0.706311	-0.01229	0.980505	0.997073	0.761478	0.135441	1	Cd14	
