# Supplementary material for: CK2 alpha prime and alpha-synuclein pathogenic functional interaction mediates synaptic dysregulation in huntington’s disease
Source: Acta Neuropathol Commun. 2022 Jun 3;10:83. doi: 10.1186/s40478-022-01379-8 (PMC9164558; doi:10.1186/s40478-022-01379-8)
Supplement: Supplementary file 12 — Additional file 12. Expression analyses for microglia markers of A1 inducing and reactive microglia (from Liddelow et al., 2017 (40)) in WT, zQ175, CK2α’(+/-) and zQ175:CK2α’(+/-). [file 40478_2022_1379_MOESM12_ESM.pdf]

Table S5. Expression analyses for microglia markers of A1 inducing and reactive microglia (from Liddelow et al., 2017)

EnsemblID	FC_HD.WT	P_HD.WT	Q_HD.WT	FC_HDHET	P_HDHET.\	Q_HDHET.	FC_HD.HD	P_HD.HDH	Q_HD.HDH	FC_HD.HE	P_HD.HET	Q_HD.HET	FC_HDHET	P_HDHET.I	Q_HDHET.	FC_HET.W	P_HET.WT	Q_HET.WT	GeneSymbol	
ENSMUSG	-0.04167	0.949115	NA	1.018271	0.105641	0.618342	-1.05994	0.094797	0.951321	-0.45668	0.535965	NA	0.603263	0.400121	NA	0.415008	0.571497	1	Il1a	PRO-A1
ENSMUSG	**-0.63462**	**4.71E-05**	**0.007512**	-0.17335	0.263496	0.79861	-0.46128	0.00323	0.437304	-0.42755	0.017655	0.200436	0.033725	0.850884	0.976156	-0.20707	0.24681	1	C1qa	
ENSMUSG	**-0.61367**	**0.000213**	**0.018214**	-0.27581	0.095037	0.592613	-0.33787	0.042444	0.853397	-0.4178	0.02911	0.259242	-0.07993	0.675618	0.943916	-0.19588	0.303464	1	C1qb	
ENSMUSG	**-0.62004**	**9.93E-05**	**0.012198**	-0.15161	0.338879	0.849425	-0.46843	0.003383	0.437834	-0.29917	0.104589	0.472687	0.169265	0.356721	0.838338	-0.32087	0.079842	1	C1qc	
ENSMUSG	1.975231	0.505055	NA	-1.4665	0.632712	NA	3.441735	0.249047	0.987901	3.62898	0.296796	NA	0	1	NA	-1.65375	0.641279	1	Tnf	
ENSMUSG	-0.03634	0.885623	0.964622	-0.19444	0.444071	0.89254	0.158098	0.535008	0.998825	0.105689	0.71806	0.928412	-0.05241	0.858488	0.977027	-0.14203	0.626729	1	Aif1	ACTIVE MICROGLIA
ENSMUSG	-0.3124	0.080804	0.377034	0.102733	0.561606	0.926814	-0.41513	0.020665	0.73565	-0.35579	0.083774	0.427952	0.059347	0.771196	0.964767	0.043386	0.831295	1	Cd68	
ENSMUSG	-0.02103	0.910504	0.971056	0.370591	0.045799	0.480457	-0.39162	0.035542	0.841866	0.082792	0.702574	0.92183	0.474417	0.027675	0.361059	-0.10383	0.631028	1	Itgam	
ENSMUSG	-0.11382	0.823587	0.939818	0.721624	0.149407	0.685753	-0.83545	0.097543	0.951321	-0.3233	0.580398	0.875162	0.512145	0.374033	0.848688	0.209479	0.718751	1	Tlr2	
ENSMUSG	0.381135	0.478002	0.781033	0.323282	0.548561	0.924202	0.057853	0.913968	1	0.694728	0.269213	0.677468	0.636875	0.312242	0.810517	-0.31359	0.619569	1	Tlr4	
ENSMUSG	0.347073	0.798252	NA	-0.53898	0.697575	NA	0.886049	0.521357	0.998804	1.727011	0.295975	NA	0.840962	0.615941	NA	-1.37994	0.404923	1	Il6	
ENSMUSG	-1.34545	0.332724	NA	-1.48827	0.292862	NA	0.142823	0.924581	1	-2.90993	0.055534	NA	-3.05276	0.047949	NA	1.564483	0.272829	1	Ccl2	
ENSMUSG	-1.08583	0.335813	NA	-0.23632	0.83223	0.980936	-0.84952	0.453301	0.996027	-0.00411	0.997518	NA	0.845411	0.518303	NA	-1.08173	0.407066	1	H2-Aa	
ENSMUSG	-0.80635	0.240393	NA	-1.00322	0.146874	0.683459	0.196874	0.779119	1	0.08161	0.919236	NA	-0.11526	0.886689	NA	-0.88796	0.264514	1	H2-Ab1	
ENSMUSG	-0.23805	0.245373	0.607058	0.05548	0.78518	0.976151	-0.29353	0.153676	0.97929	-0.38802	0.098727	0.4611	-0.09449	0.686104	0.94605	0.149974	0.519948	1	H2-DMa	
ENSMUSG	-1.10964	0.165044	NA	0.250172	0.743437	NA	-1.35982	0.088538	0.943855	-0.73899	0.424087	NA	0.620821	0.487635	NA	-0.37065	0.678857	1	H2-DMb1	
ENSMUSG	-2.83206	0.001213	NA	-0.6804	0.367829	NA	-2.15165	0.015746	0.683595	-1.32128	0.19756	NA	0.830377	0.369534	NA	-1.51078	0.096926	1	H2-DMb2	
ENSMUSG	-1.4041	0.150192	NA	-1.95448	0.049806	NA	0.550381	0.58954	1	-0.36475	0.749058	NA	-0.91513	0.429316	NA	-1.03935	0.352986	1	H2-Eb1	
ENSMUSG	-1.41217	0.113848	NA	-2.24249	0.020591	NA	0.830315	0.416026	0.996027	-0.8705	0.400639	NA	-1.70081	0.122522	NA	-0.54167	0.582039	1	H2-Oa	
ENSMUSG	-0.59732	0.371041	NA	-0.13365	0.839062	0.981536	-0.46367	0.491657	0.996027	-0.2387	0.758777	NA	0.224978	0.769856	NA	-0.35863	0.638466	1	H2-Ob	
