# Supplementary material for: CK2 alpha prime and alpha-synuclein pathogenic functional interaction mediates synaptic dysregulation in huntington’s disease
Source: Acta Neuropathol Commun. 2022 Jun 3;10:83. doi: 10.1186/s40478-022-01379-8 (PMC9164558; doi:10.1186/s40478-022-01379-8)
Supplement: Supplementary file 14 — Additional file 14. Marker genes and their mean log2 fold change between zQ175 and zQ175:CK2α’(+/-) mice compared to WT. [file 40478_2022_1379_MOESM14_ESM.pdf]

**Table S6. Marker genes and their mean log2 fold change between zQ175 and zQ175:$CK2\alpha'^{(+/-)}$ mice compared to WT**

| GeneSymbol | FC_HD.HDHE | P_HD.HDH | Q_HD.HDHET | Module WGNA | Synaptic Function | Gene description | Link Genecards |
|---|---|---|---|---|---|---|---|
| **Gm30085** | 4.7648641 | 8.54E-05 | 0.081847 | | | Unknown | |
| **Grm2** | 3.2378454 | 2.45E-05 | 0.039975 | 5 | YES | Glutamate Metabotropic Receptor 2. Involved in synaptogenesis and/or synaptic stabilization | https://www.genecards.org/cgi-bin/carddisp.pl?gene=GRM2 |
| **Slc30a3** | 3.091492 | 0.000123 | 0.09839 | 5 | YES | Solute carrier family 30 member 3 (ZnT-3 family membrane transport protein) | https://www.genecards.org/cgi-bin/carddisp.pl?gene=SLC30A3 |
| **Gm42598** | 2.8563671 | 7.11E-05 | 0.078997 | 20 | | Unknown | |
| **Gm2824** | 2.2739586 | 9.18E-05 | 0.084999 | 19 | | Unknown | |
| **Slc17a7** | 2.2325005 | 0.000138 | 0.09839 | 5 | YES | Vesicular Glutamate transporte VGlut1 | https://www.genecards.org/cgi-bin/carddisp.pl?gene=SLC17A7&keywords=Slc17a7 |
| **Nr4a2** | 2.2250106 | 1.00E-10 | 9.26E-07 | 20 | YES | Nuclear receptor related 1 protein (NURR1), is a member of the nuclear receptor family of intracellular t | https://www.genecards.org/cgi-bin/carddisp.pl?gene=NR4A2&keywords=Nr4a2 |
| **C1ql3** | 1.7864526 | 3.99E-07 | 0.001865 | 5 | | C1q/TNF-Related Protein 3, May regulate the number of excitatory synapses that are formed on hippoc | https://www.genecards.org/cgi-bin/carddisp.pl?gene=C1QL3&keywords=C1ql3 |
| **Iqcj** | 1.7861791 | 6.69E-05 | 0.077457 | 20 | YES | Calmoduling binding motif protein | https://www.genecards.org/cgi-bin/carddisp.pl?gene=IQCJ&keywords=Iqcj |
| **C230012O17Rik** | 1.7844816 | 0.000134 | 0.09839 | 20 | | Unknown | |
| **Gm17807** | 1.4859631 | 8.06E-05 | 0.081402 | 20 | | Unknown | |
| **Tbr1** | 1.4502627 | 8.61E-06 | 0.022609 | 5 | YES | Transcription factor protein important in vertebrate embryo development | https://www.genecards.org/cgi-bin/carddisp.pl?gene=TBR1&keywords=Tbr1 |
| **Cckbr** | 1.3961687 | 3.71E-06 | 0.012901 | 20 | YES | G protein-coupled receptor for gastrin and cholecystokinin, influence neurotransmission in the brain, re | https://www.genecards.org/cgi-bin/carddisp.pl?gene=CCKBR&keywords=Cckbr |
| **Vxn** | 1.3540086 | 1.01E-07 | 0.000699 | 19 | YES | Vexin, required for neurogenesis in the neural plate and retina. Strongly cooperates with neural bHLH f | https://www.genecards.org/cgi-bin/carddisp.pl?gene=VXN&keywords=Vxn |
| **Col5a1** | 1.3206734 | 0.000117 | 0.09839 | 16 | | Collagen Type V Alpha 1 Chain, provides instructions for making a component of type V collagen | https://www.genecards.org/cgi-bin/carddisp.pl?gene=COL5A1&keywords=col5a1 |
| **Nrp2** | 1.10989 | 5.99E-05 | 0.075648 | 5 | | Neuropilin 2 interacts with vascular endothelial growth factor (VEGF). This protein may play a role in ca | https://www.genecards.org/cgi-bin/carddisp.pl?gene=NRP2&keywords=Nrp2 |
| **Fbln2** | 1.0300568 | 4.03E-07 | 0.001865 | 19 | YES | This gene encodes an extracellular matrix protein, which belongs to the fibulin family. This protein binds | https://www.genecards.org/cgi-bin/carddisp.pl?gene=FBLN2&keywords=Fbln2 |
| **Csnk2a2** | 0.9857398 | 1.13E-23 | 3.15E-19 | 20 | | Protein kinase CK2 alpha prime. Catalytic subunit of CK2 holoenzyme | https://www.genecards.org/cgi-bin/carddisp.pl?gene=CSNK2A2&keywords=Csnk2a2 |
| **Pde1a** | 0.9491691 | 1.06E-05 | 0.022609 | 20 | YES | Calcium/calmodulin-dependent 3',5'-cyclic nucleotide phosphodiesterase 1A | https://www.genecards.org/cgi-bin/carddisp.pl?gene=PDE1A&keywords=Pde1a |
| **Cpne4** | 0.815418 | 2.35E-05 | 0.039975 | 19 | YES | Calcium-dependent membrane-binding proteins may regulate molecular events at the interface of the c | https://www.genecards.org/cgi-bin/carddisp.pl?gene=CPNE4&keywords=Cpne4 |
| **Gm45640** | 0.7899246 | 0.00013 | 0.09839 | 20 | | Unknown | |
| **Ncald** | 0.7531203 | 3.29E-05 | 0.050717 | 5 | YES | Neuronal calcium-binding protein that belongs to the neuronal calcium sensor (NCS) family of proteins. | https://www.genecards.org/cgi-bin/carddisp.pl?gene=NCALD&keywords=Ncald |
| **Basp1** | 0.5603242 | 5.57E-05 | 0.073668 | 19 | YES | Brain Abundant Membrane Attached Signal Protein 1 | https://www.genecards.org/cgi-bin/carddisp.pl?gene=BASP1&keywords=Basp1 |
| **Pgm2l1** | 0.5560045 | 2.08E-05 | 0.038578 | 5 | | PGM2L1 (Phosphoglucomutase 2 Like 1) is a Protein Coding gene. Among its related pathways are Me | https://www.genecards.org/cgi-bin/carddisp.pl?gene=PGM2L1&keywords=Pgm2l1 |
| **Erc1** | 0.399286+D3 | 1.02E-05 | 0.022609 | 5 | YES | RIM-binding protein. RIMs are active zone proteins that regulate neurotransmitter release. | https://www.genecards.org/cgi-bin/carddisp.pl?gene=ERC1&keywords=Erc1 |
| **Ctsd** | -0.4584743 | 9.26E-06 | 0.022609 | 19 | | CTSD gene provides instructions for making an enzyme called cathepsin D. | https://www.genecards.org/cgi-bin/carddisp.pl?gene=CTSD&keywords=Ctsd |
| **Npc2** | -0.4653114 | 4.68E-05 | 0.068443 | 5 | | NPC Intracellular Cholesterol Transporter 2, transports cholesterol through the late endosomal/lysosom | https://www.genecards.org/cgi-bin/carddisp.pl?gene=NPC2&keywords=Npc2 |
| **Elovl7** | -0.7269098 | 0.000135 | 0.09839 | 20 | | Fatty Acid Elongase 7. Among its related pathways are Fatty Acyl-CoA Biosynthesis and Metabolism | https://www.genecards.org/cgi-bin/carddisp.pl?gene=ELOVL7&keywords=Elovl7 |
| **Tcn2** | -0.7635684 | 6.45E-05 | 0.077457 | 19 | | Transcobalamin II (TC II), a plasma globulin that acts as the primary transport protein for vitamin B12 | https://www.genecards.org/cgi-bin/carddisp.pl?gene=TCN2&keywords=Tcn2 |
| **Rtraf-ps** | -0.8885925 | 0.000132 | 0.09839 | 20 | | RNA transcription, translation and transport factor, pseudogene | |
| **BC067074** | -0.9301093 | 1.78E-06 | 0.00706 | 20 | | Unknown | |
| **Lyz2** | -1.0403831 | 5.40E-05 | 0.073668 | 20 | | antibacterial enzyme lysozyme M (LysM) | https://www.genecards.org/cgi-bin/carddisp.pl?gene=LYZ&keywords=Lyz2 |
| **Klf4** | -1.136359 | 7.75E-05 | 0.081402 | 20 | | Kruppel-like factor 4 is a member of the KLF family of zinc finger transcription factors, which belongs to | https://www.genecards.org/cgi-bin/carddisp.pl?gene=KLF4&keywords=Klf4 |
| **Slc18a3** | -1.1761844 | 1.89E-05 | 0.037482 | 20 | YES | Solute Carrier Family 18 (Vesicular Acetylcholine Transporter), Member 3 | https://www.genecards.org/cgi-bin/carddisp.pl?gene=SLC18A3&keywords=Slc18a3 |
| **Gm2991** | -1.6784161 | 0.00012 | 0.09839 | 20 | | Unknown | |
| **Gm42047** | -2.5858568 | 6.38E-06 | 0.019685 | 20 | | lncRNA | |
| **Tmem88** | -3.1280924 | 8.20E-05 | 0.081402 | 9 | YES | Transmembrane protein 88 Inhibits the Wnt/beta-catenin signaling pathway | https://www.genecards.org/cgi-bin/carddisp.pl?gene=TMEM88&keywords=Tmem88 |
| **4933406B17Rik** | -3.7325491 | 9.85E-11 | 9.26E-07 | 20 | | Long non coding RNA associated with Csnk2a2 promoter regulation | http://useast.ensembl.org/Mus_musculus/Gene/Summary?db=core;g=ENSMUSG00000110427;r=8:95496430-9 |
| **Ttr** | -6.9107612 | 9.50E-05 | 0.085158 | 13 | | Transthyretin. This protein transports vitamin A (retinol) and a hormone called thyroxine throughout the | https://www.genecards.org/cgi-bin/carddisp.pl?gene=TTR&keywords=Ttr |
